# A change point analysis protocol for comparing intracellular transport by different molecular motor combinations

**DOI:** 10.3934/mbe.2021442

**Published:** 2021-10-18

**Authors:** Melanie A. Jensen, Qingzhou Feng, William O. Hancock, Scott A. McKinley

**Affiliations:** 1 Department of Mathematics, Tulane University, New Orleans, LA 70118, USA; 2 Department of Biomedical Engineering, Pennsylvania State University, University Park, PA 16802; 3 Molecular Cellular and Integrative Biological Sciences Program, Huck Institute of Life Sciences, Pennsylvania State University, University Park, PA 16802; 4 Schlumberger, 1 Hampshire St Ste 1, Cambridge, MA, 02319 USA; 5 Department of Cell Biology, Yale School of Medicine, Yale University, New Haven, CT 06520

**Keywords:** intracellular transport, molecular motors, change point analysis

## Abstract

Intracellular transport by microtubule-based molecular motors is marked by qualitatively different behaviors. It is a long-standing and still-open challenge to accurately quantify the various individual-cargo behaviors and how they are affected by the presence or absence of particular motor families. In this work we introduce a protocol for analyzing change points in cargo trajectories that can be faithfully projected along the length of a (mostly) straight microtubule. Our protocol consists of automated identification of velocity change points, estimation of velocities during the behavior segments, and extrapolation to motor-specific velocity distributions. Using simulated data we show that our method compares favorably with existing methods. We then apply the technique to data sets in which quantum dots are transported by Kinesin-1, by Dynein-Dynactin-BicD2 (DDB), and by Kinesin-1/DDB pairs. In the end, we identify pausing behavior that is consistent with some tug-of-war model predictions, but also demonstrate that the simultaneous presence of antagonistic motors can lead to long processive runs that could contribute favorably to population-wide transport.

## 1. Introduction

**Figure 1.**
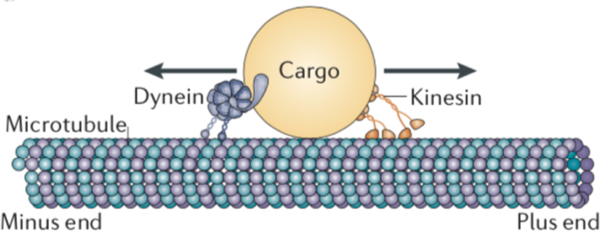
Anterograde moving motors, such as kinesin, transport cargo towards the cell periphery (plus end) by stepping along the microtubule, while retrograde moving motors, such as dynein, carry the cargo towards the cell nucleus (negative end). Figure copied with permission from [10].

Intracellular transport of vesicles, organelles and other biomolecular cargo is commonly carried about by two families of motor proteins (kinesin and dynein) that tether cargoes to microtubules in the cell and move along these microtubules in a directed fashion. Microtubules have a structural polarization, termed the plus-end and a minus-end. Motors in the kinesin family generally move in the plus-end (anterograde) direction, while cytoplasmic dynein moves in the minus-end (retrograde) direction. Typically, more than one motor will be attached to a cargo at a time, and it has been a long-term theoretical challenge to understand how multiple motors cooperate [1, 2, 3, 4] and/or compete [5, 6, 7, 8] in the course of cargo transport. See Figure 1 for a schematic depiction. Indeed, particle tracking experiments for live cells have consistently revealed cargo trajectories that are bidirectional, marked by periods of both anterograde and retrograde motion, while featuring periods when transport appears to be “paused” [9, 10]. The causes of the pauses [11, 12] and the manner in which the motors exchange dominance to give rise to bidirectionality remain the focus of extensive investigation [10, 13, 14].

**Figure 2.**
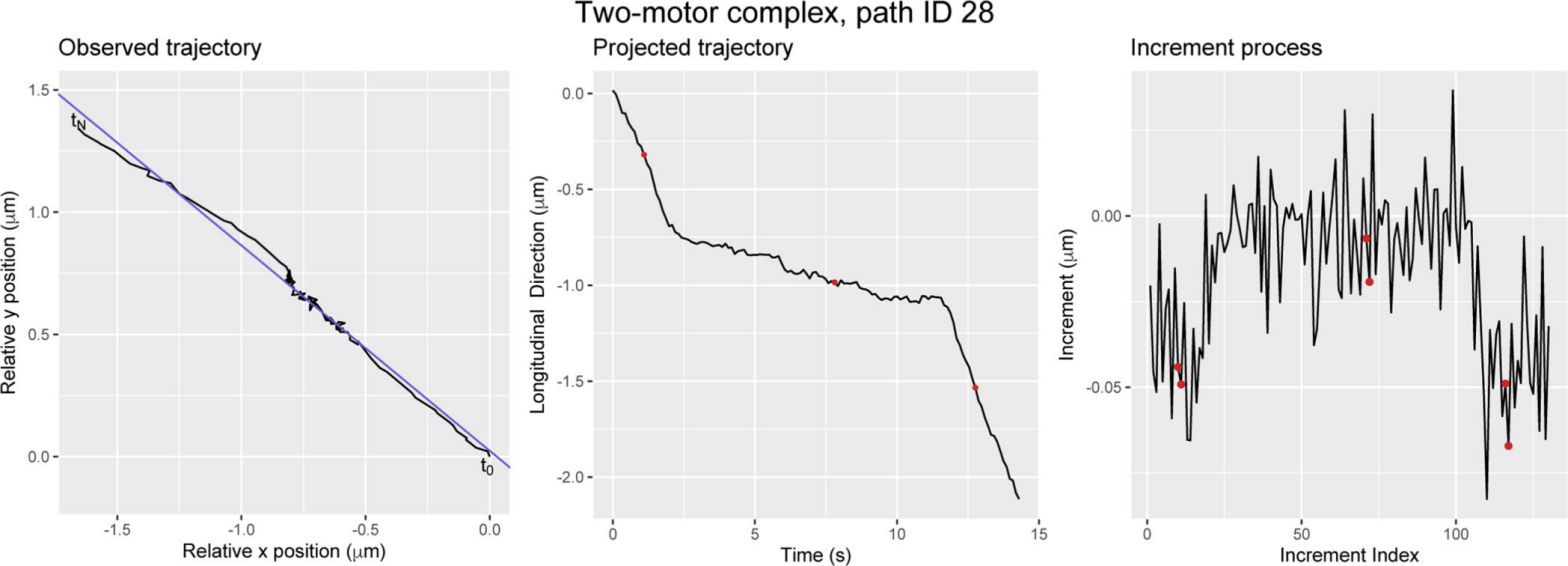
**Left:** The tracked cargo tethered to a two-motor complex trajectory (black curve) in the (x,y) plane, where *t*_0_ denotes the starting position and $t_{N}$ denotes the ending position with respect to time. The purple line denotes the estimated position of the microtubule. **Center:** The trajectory (black curve) in the time, longitudinal distances coordinate system, where the red circles correspond to missing observations that have been estimated. **Right:** The increment process of the trajectory, where the red circles indicate the increments calculated from the missing observations that were estimated.

Initially, in vitro experiments of motor-driven cargo transport by motors were in one of two categories: (1) transport by a single motor, achieved by holding the motor concentration very low compared to cargo concentration thereby assuring at most one motor is attached; or (2) multi-motor transport with an uncertain number of motors being involved in the transport [15, 16, 17]. More recently, DNA origami techniques have been developed in order to observe transport by exactly two motors, which may or may not be of the same type [18, 19, 20, 21]. The effort to make comparisons between single-and multi-motor transport has raised a number of methodological challenges. Two such challenges that we seek to address here are: (1) how to identify when a motor-cargo complex switches from one biophysical state to another; and (2) how to characterize and quantify the distribution of velocities exhibited by transport mediated by various motors and motor-motor combinations. A typical path of interest is displayed in Figure 2. The cargo is initially being transported by the motor and then comes to a stop. It pauses temporarily (with some continued progress) and then it begins stepping again until the end of the observed time.

A variety of methods to address questions like this exist in the literature. Some have been developed specifically for particle tracking data, while others (change point detection algorithms in particular) have been developed with other applications in mind. In Section 1.1 we survey some relevant methods and in this work we develop a particle-tracking specific algorithm that is informed by some of these “best practices” that have been explored in the statistics literature. One collection of work that was particularly influential for us is that generated by the KymoAnalyzer software developed by the Encalada lab [22]. This tool for quantifying behavior observed in kymograph data allows a user to partition individual paths into segments, and then estimate the velocity of each segment. The collection of observed velocities can then be binned in a histogram or displayed as a cumulative distribution function, either of which can be sufficiently detailed to capture qualitative differences in behavior observed in various experiments. The two major drawbacks to using KymoAnalyzer (or any similar tool) stem from the “use of the human hand” in the analysis. Because the segments must be marked individually, user-time is a limiting factor, preventing analysis of massive data sets. Moreover, different users can have different habits in determining when segments occur, leading to biases that are difficult to quantify.

In the work that follows, we take a step toward developing a protocol for finding change points and reporting velocity distributions that is fully automated, once some initial settings are put in place. In Section 1.2 we summarize the method that we have implemented, which is Bayesian in the overall framework, but relies on a number of approximations to be computationally feasible. Subsequently, in Sections 3 and 4 we provide a detailed development of our approach to modeling both the data and the underlying biophysical process for the purposes of quantifying the data and simulating artificial data, respectively. We then describe our procedure for validating the statistical methods we employ. In Section 5 we report how various change point methods perform on the simulated data and then ultimately we apply our method to data sets featuring quantum dots being transported by a single kinesin-1 (kin1) motor, a single dynein-dynactin-BicD2 (DDB) motor, and a kin1-DDB pair. We are able to quantify differences in velocity distributions and run lengths and reveal a pattern of behavior that, at least from this preliminary perspective, is neither completely consistent with tug-of-war hypothesis predictions, nor with the in vivo observations of co-dependence among antogonistic motors.

### 1.1. Existing methods in change point detection

The change point detection problem is well-studied, dating back at least to the work of Page in the 1950s. After laying down a framework for detecting a change in parameter using cumulative sum schemes for general distributions [23, 24], Page specifically addressed a problem much like what we study here in 1957, looking at the change in mean of independent normally distributed observations [25]. In 1964, Chernoff and Zacks [26] derived a Bayesian estimator for segment means under the assumption that there is at most one change. The problem becomes considerably more difficult when the number and location of the change points is not known. The literature on this topic is extensive, but we would like to highlight a few prominent methods that we include in our numerical analysis.

Essentially all change point detection schemes rely on proposing a number of changes, and the locations of those changes, and then using a probability model to evaluate a likelihood score. Since a model with more change points can always be made to fit the data better, there is a risk of overfitting a trajectory. There must be some penalty in the score for how many change points are proposed. These two components are very explicit in algorithms that construct a piecewise-linear approximation of the data and compute the score based on the sum of squares of the residuals (RSS). One such algorithm, proposed by Bai and Perron [27, 28], employs a dynamic programming algorithm to minimize RSS. This has been implemented in the R package strucchange, in which the function breakpoints uses the Bayesian Information Criterion (BIC) to penalize larger numbers of change points.

Bayesian methods take a random sampling approach to finding good parameter sets. In these algorithms, a sequence of location vectors and associated means are proposed. At each step a likelihood score is evaluated and the proposed parameter set is accepted or rejected in accordance with the Metropolis-Hastings algorithm. Here the penalty is encoded in prior information about the anticipated probability that any given observation might be the location of a change point. Once a mathematical model is specified, innovations come about through proposing more efficient methods of exploring the parameter space. To our knowledge, the first Bayesian method for detecting multiple change points with unknown number was proposed by Barry and Hartigan [29]. They called their mathematical model the Product Partition Model (PPM), which encompasses all models that satisfy a product condition on partitions (the change points) and an independence condition for the observations given the partition. Barry and Hartigan construct a PPM in [30] that performs well in detecting sharp short-lived changes in the means of independently normally distributed observations. The PPM specified in [30] has been implemented as an R package bcp by Erdman et al. [31].

Within the particle tracking community, in 2018 Yin et al. [32] proposed using a likelihood ratio test (LRT) to classify a time as a change point. To identify all the change points in a single trajectory, the authors utilized a recursive binary segmentation algorithm. Such a method is feasible for the case of a sequence of Normal random variables with change in mean parameter and common variance because the log likelihood ratio is a well studied object and estimates of the critical value exist, see [33]. We refer to this method as LRT in our analysis.

### 1.2. Comparison of our method to existing Bayesian methods

In this work, we construct a PPM that differs from [30] in the modeling of the segment means and segment durations. Barry and Hartigan employ a hierarchical model for the segment means. Each segment mean is assumed to be drawn from a Normal distribution that has common mean, *μ*_0_, and variance $\sigma_{0}^{2}/|I|$ that is inversely proportional to the segment length, |I|. One of the objectives of this work is to infer the parameters $\mu_{0}$ and $\sigma_{0}^{2}$. Such a model does well for sequences with one typical behavior and short departures from this behavior. This model structure may not be well-suited for many motor-cargo trajectories, which commonly involve a switch between two prolonged states. Moreover, the model prescribes the shape for the segment mean distribution that does not allow for important qualitative features that might exists in motor data, in particular multimodality. The method we propose takes advantage of Bayesian sampling techniques but does impose a model on the velocities. For this reason, we remove the hierarchical structure on the segment means and treat each segment velocity as a distinct parameter with a uniform prior with bounds determined by existing knowledge about motor speeds. Additionally, we modify the geometric distribution prior on the segment lengths used in [30] to guard against short unrealistic segments that commonly arise from tracker errors.

In theory, one could implement a fully Bayesian non-parametric method to infer the change points, velocities, and velocity distribution structure all at once. However, this approach is computationally intractable for most desktop settings. In fact, despite efforts to minimize computational cost, the protocol we propose is itself computationally intensive. With these computational barriers in mind, we split our inference method into multiple stages. In the first stage, we introduce a likelihood approximation, utilizing the intuition underlying Bayes Factors, that reduces the dimension of the parameter space and allows us to estimate the most likely number of change points. Then given a number of change points, in the second stage, we use MCMC methods to sample from the posterior distribution of the position of the change points and the segment velocities. We then construct an estimated velocity distribution using samples from the stage 2 posterior distributions that are weighted by segment length. We refer to the method as *Number of Change points - Conditional Inference (NC-CI).*

## 2. Experimental methods

### 2.1. Collection of in-vitro data

Kinesin-1 motors were bacterially expressed and purified by Ni column chromatography, as described previously [20]. Dynein-dynactin-BicD2 (DDB) complexes were purified by adding bacterially expressed BicD2 to bovine brain lysate and purifying DDB complexes by column chromatography, as described previously [21]. Both motors contained a C-terminal Green Fluorescent Protein (GFP). Isolated kinesin-1 and DDB were visualized by attaching streptavidin-coated Quantum dots (Qdots) to the motors through a biotinylated anti-GFP nanobody (GFP Binding Protein, or GBP). Kinesin-DDB complexes were formed by attaching single-stranded DNA-functionalized GBP to each motor and connecting them through a complementary single-stranded oligonucleotide labeled with a Qdot. All of the methods for forming complexes are described in Feng et al. [21]. The Qdot label has a diameter of approximately 0.03*μ*m and estimated diffusivity of 10*μ*m^2^/*s* (based on Stokes-Einstein equation). Motors were tracked by visualizing the Qdots with Total Internal Reflection Fluorescence Microscopy on a Nikon TE-2000 microscope, as previously described [21].

Transport by Kinesin-1 has been well studied since the 1990s. Kinesin-1 has been shown to be an extremely processive motor taking approximately 100 steps, each of size ≈ 0.008*μ*m, before detaching from the microtubule. The unloaded velocity is roughly 100 steps/s, or 0.8 *μ*m/s [34, 35, 36], and the motor is able to sustain loads of roughly 6 pN [15, 37, 38].

Due to this consistent processive behavior, we treated kinesin data as a control group, in which we did not expect to detect many change points. There were 34 paths within the Kinesin-1 data set, in which observations were taken at 20 frames per second (fps) (Δ = 0.05*s*). By contrast, activated dynein in a DDB complex has been shown to display diverse motitily behavior in vitro, including processive runs, pauses, and diffusive behavior [19, 21, 39]. There were 42 tracked paths in this group with observations taken 22 fps (Δ = 0.043s). In our analysis, we considered a subset of 41 DDB motor-cargo paths with approximately linear microtubules.

#### 2.1.1. Data for cargo simultaneously bound to one Kinsein-1 and one DDB motor

Less is known about how a single cargo is transported by a multimotor complex comprised of antagonistic motors such as Kinesin-1 and DDB [10]. The last data set we studied consists of individual Qdots being transported by both a Kinesin-1 and a DDB motor, referred to as Kin1-DDB. These complexes have been assembled previously using DNA origami and shown to display tug-of-war behavior. There were 101 tracked particles and observations are taken 9fps (Δ ≈ 0.11*s*). Because our technique is restricted to paths that are approximately linear, the excluded set of paths was larger for this data set than the others. We included 92 out of 101 paths. It is not immediately clear why there are more “bent” paths, but one likely explanation is that Kin1-DDB complexes may be more likely to switch between different microtubules before detaching.

## 3. Statistical methods and algorithms

In this section, we present the mathematical model and algorithm used in this work. In Section 3.1 we present two models for the motor-cargo system, the first is a biophysical model based on Langevin dynamics, and the second is an approximation to the biophysical model that yields explicit solutions that can be used for statistical inference. In Section 3.2, we construct a Bayesian approach to estimating the number of change points that exist within a path. This is the first step in our two-step method. Then in Section 3.3, we provide the sampling algorithm to infer change point locations and segment velocities for a given number of change points, which constitutes the second step of our method. We give definitions of quantities of interest estimated from the outputs of the two proposed sampling algorithms in Section 3.4. Lastly, in Section 3.5 we describe the statistical methods used to clean and pre-process the molecular motor data described in Section 2.

### 3.1. Mathematical models for motor cargo-system

#### 3.1.1. Biophysical model

The motor-cargo process is intrinsically stochastic due to motor-stepping and the fluctuations of the associated cargo. We assume that the various motor configuration states (for example, a single kinesin motor transporting the cargo, or a single DDB motor, or a kinesin-DDB pair) can be modeled effectively through assigning a mean stepping rate for each state. To this end, suppose there are *k* changes in motor interactions at times $\tau =\left(\tau_{1},\ldots ,\tau_{k}\right)$ with the additional notational convention that $\tau_{0}=0$. Within the *j*th time segment, $\tau_{j-1}\leq t<\tau_{j}$, the motor configuration is characterized by a distinct stepping rate, $\rho_{j}$, which can be either a deterministic value, or drawn from some probability distribution. We then model the motor step times within a segment as a Poisson arrival process with rate parameter $\rho_{j}$. Assuming that each motor step is of size *δ*, it follows that the position of the motor configuration center, *Z*(*t*), is a scaled Poisson process,

(3.1) $$(Z(t)-Z(s))/\delta ∼\;\text{Poisson}\;\left((t-s)\rho_{j}\right)\;\text{for}\;\tau_{j-1}\leq s<t\leq \tau_{j}$$

for j=1,…k.

As for the position of the cargo at time *t*, denoted *X*(*t*), we consider a Langevin Stochastic Differential Equation (SDE) framework in the overdamped (zero mass) limit. To be specific, the position of the cargo is governed by an Ornstein-Uhlenbeck process centered at *Z*(*t*):

(3.2) $$\text{d}X(t)=-\frac{\kappa }{\gamma }(X(t)-Z(t))\;\text{d}t+\sqrt{2D}\;\text{d}W(t),\;X(0)=x_{0},$$

where *κ* is the spring constant of the tether, *γ* is the viscous drag coefficient of the cargo, $D=k_{B}T/\gamma$ is the diffusivity of the cargo in the absence of the tether (with *k*_*B*_*T* being Boltzmann’s constant times the fluid temperature), and *W*(*t*) is a standard Brownian motion.

#### 3.1.2. Statistical Model: Brownian motion with drift

In experimental settings, the position of the motor *Z*(*t*) is usually unknown. It is possible to estimate motor positions from cargo observation, but since the motor model is phenomenological to begin with, and since the quantities of interest are the various velocity states of the motor-cargo complex, we introduce a simplification that is used primarily for statistical inference purposes. The statistical model is expressed in terms of two non-physical parameters: the state velocity *ν*_*j*_, and the state diffusivity 1/η. In this statistical model, the (observed) position of the cargo $\hat{X}(t)$ with *k* changes in velocities is described by Brownian motion with changes in drift parameter:

(3.3) $$\text{d}\hat{X}(t)=v_{j}\text{d}t+\sqrt{1/\eta }\text{d}W(t)\;\text{for}\;\tau_{j-1}<t\leq \tau_{j},\;\hat{X}(0)=x_{0}.$$

The drift parameter $v_{j}(j\in \{1,2,\ldots ,k+1\})$ is the velocity of the cargo during the *j*th state (roughly $\rho_{j}\delta$). The common diffusion parameter 1/η is treated as a nuisance parameter (its exact value is not of primary concern in this work) since it is an amalgamation of multiple sources of noise in the system: fluctuations in motor-stepping, fluctuations of the cargo about the motor positions, and observation error. This statistical model has the same form as a standard diffusion approximation for a Poisson process [40] (which would have the form $1/\eta =v_{j}$) but assuming this strict form would not take into account other contributions to the noise.

Because single particle tracking data is collected at discrete times, we further assume that observations are made on a uniform grid of size $\text{Δ}=t_{n}-t_{n-1}$ for n=1,…N and that Δ is small enough so the *j*th change point can be approximated by an observation time,

(3.4) $$\tau_{j}=t_{M_{j}}\;\text{where}\;M_{j}:=\left\lfloor \tau_{j}/\text{Δ}\right\rfloor >0$$

for all j=1,…,k, with the convention that $\tau_{0}=0$ and $\tau_{k+1}=N\text{Δ}$. Here $M_{j}$ denotes the index of the *j*th change whereas *τ*_*j*_ denotes the time of the *j*th change. Under these assumptions for times $t\in \left[t_{M_{j}},t_{M_{j+1}}\right)$, the position of the observed cargo conditioned on the position of the *j*th change, ${\hat{X}}_{M_{j}}=\hat{X}\left(\text{Δ}M_{j}\right)$, is given by

(3.5) $${\hat{X}(t)|}_{{\hat{X}}_{M_{j}}}={\hat{X}}_{M_{j}}+v_{j+1}\left(t-t_{M_{j}}\right)+\sqrt{1/\eta }.$$

From Equation 3.5, the increment process of $\hat{X}$, that is ${\left\{{\text{Ξ}}_{n}:={\hat{X}}_{n+1}-{\hat{X}}_{n}\right\}}_{n=1}^{N}$, has the following distribution

(3.6) $${\text{Ξ}}_{n}∼N\left(v_{j}\text{Δ},\eta^{-1}\text{Δ}\right)\;\text{if}\;\tau_{j-1}<t_{n}\leq \tau_{j},$$

for 1≤j≤k+1, n=1,…N. Denoting a realization of the increment process by $\xi =\left(\xi_{1},\ldots ,\xi_{N}\right)$, our statistical model assuming *k* change points has an approximate likelihood function

(3.7) $$L_{N,k}\left(\xi ;\theta_{k}\right)={\left(\frac{\eta }{2\pi \text{Δ}}\right)}^{N/2}\exp\left(\frac{-\eta }{2\text{Δ}}\sum_{j=1}^{k+1}\sum_{i=M_{j-1}+1}^{M_{j}}{\left(\xi_{i}-v_{j}\text{Δ}\right)}^{2}\right)$$

where $\theta_{k}=\left(\tau_{1},\ldots \tau_{k},v_{1},\ldots v_{k+1},\eta \right)$ denotes the model parameters with *k* change points. We treat the vector of change point times, $\tau_{k}$, as model parameters rather than the vector of change point indices, $M_{k}=\left(M_{1},\ldots M_{k}\right)$, since the latter is computable from $\tau_{k}$ and Δ. We refer to the *j*th segment as the increments between $M_{j-1}+1$ and $M_{j}$. In the special case when all the change points occur at observation times, Equation 3.7 is exact.

Under this approximation our change point problem for molecular motors can be written as a sequence of independent normal random variables with changes in mean parameter. From this point forward, our inference technique assumes the statistical model with *k* changes,

(3.8) $${𝓜}_{k}:\xi_{n}∼\;\text{Normal}\left(v_{j}\text{Δ},\eta^{-1}\text{Δ}\right),\;\text{for}\;M_{j-1}<n\leq M_{j},j\in \{1,\ldots k+1\},$$

where $M_{0}=0$ and $M_{k+1}=N$. The unknown parameters of interest are *k*, ***τ***, ***ν***, and *η*.

### 3.2. Posterior approximation for selecting number of change points

In this section, we provide our approach to approximating the marginal posterior probability distribution of the number of change points. We constructed a Metropolis-Hastings (MH) sampling algorithm that utilizes a switch point process to infer the number of change points, *k*. In Lavielle et al. [41], the switch point process was defined as an (*N* − 1)-dimensional vector of independent Bernoulli random variables, denoted $r=\left(r_{1},\ldots ,r_{N-1}\right)$, where $r_{n}=1$ indicates a change point occurred at time observation *n* with some probability that is known a priori. By contrast, we take *λ*, the rate of changes among states, to be unknown. So, the parameters to be inferred in our model are the segment mean velocities, ***ν***, the common precision, *η*, the switch point process, ${r|}_{\lambda }$, and the rate of switches, λ. Assuming independence among the parameters *η*, ***ν***, and ${r|}_{\lambda }$, our model results in a joint posterior distribution of the form

(3.9) $$\begin{array}{l} p(r,\lambda ,v,\eta |\xi )\overset{C}{=}L_{N,K_{r}}(\xi ;r,v,\eta )p(r,\lambda ,v,\eta )\\ \;=L_{N,K_{r}}(\xi ;r,v,\eta )\left(\prod_{j=1}^{K_{r}+1}p\left(v_{j}\right)\right)p(\eta )p(r|\lambda )p(\lambda ) \end{array}$$

where $\overset{C}{=}$ denotes equality up to a constant, *K*_*r*_ is the number of of change points in switch point process $r,L_{N,K_{r}}(\xi ;r,v,\eta )$ is given in Equation 3.7, and p(•) denotes the prior distribution of unknown model parameters. Estimating all of these parameters simultaneously is computationally prohibitive. As such we have taken the approach to integrate over all possible segment velocities, weighted by their prior distribution, and then we infer the remaining parameters ***r***, *λ*, and *η*. This marginalization of the velocities is similar to the prior-weighted averaging that takes place in computing Bayes factors for model selection [48]. Under this model reduction strategy, the target distribution becomes

(3.10) $$p(r,\lambda ,\eta |\xi )\overset{C}{=}\left(\int_{\mathbb{R}}\ldots \int_{\mathbb{R}}L_{N,K_{r}}(\xi ;r,v,\eta )p\left(v_{1}\right)\ldots p\left(v_{K_{r}+1}\right)dv_{1}\ldots dv_{K_{r}+1}\right)p(\eta )p(r|\lambda )p(\lambda ).$$

We provide explicit forms of Equation 3.10 with our choices of prior distributions at the end of this subsection.

Our target distribution includes the locations of the changepoints, the switch rate, and the common precision. This is different from existing Bayesian methods. For the bcp algorithm of Barry & Hartigan [30] and Erdman & Emerson [31], the posterior distribution is the joint distribution of the change points (partitions) and the segment means, while the switch rate, *λ* and the common variance (1/*η*) are assumed to be known. In the work by Lavielle & Lebarbier [43], two posterior distributions are considered; the switch point process and the joint distribution of the switch point process and segment means. The former is obtained by integrating out the segment means. Both posterior distributions are feasible because the switch rate and the common variance 1/*η* are estimated from the data and then fixed.

#### 3.2.1. Prior and hyper-prior selection

The following discussion concerns both model parameters to be inferred and hyperparameters that define the associated prior distributions. To distinguish between them, the hyperparameters are under-lined.

Our prior for the switch point process is motivated by interpreting the switch point process, *r*(*t*), as a continuous-time counting process where change points occur with rate *λ*, *r*(*t*) ∼ Poisson(*λt*). However, the times between events in a Poisson process can be arbitrarily small and this is inconsistent with the idea that biophysical states must persist for some period of time to be meaningful. We therefore introduced a minimum length for allowable segment durations, which is at least ${\underline{d}}_{r}$ observations. This means that the posterior distribution of switch point processes is restricted to the set of all switch point processes that have all change points separated by at least ${\underline{d}}_{r}$ steps. We denote this set ${𝓣}_{N,{\underline{d}}_{r}}$. We refer to ${\underline{d}}_{r}$ as the *minimum segment duration* and we select it according to the following reasoning. Suppose a molecular motor needs to step at least ten times in order for the segment to have significance and the motor is assumed to step on average once every 0.01 seconds. Then ${\underline{d}}_{r}$ would be chosen to be ${\underline{d}}_{r}=\left\lceil (10*0.01)/\text{Δ}\right\rceil$, where Δ is the time between observations.

Under this modeling assumption, the prior distribution on the switch point process is

(3.11) $$p\left(r;\lambda ,{\underline{d}}_{r}\right)=\{\begin{array}{ll} {\left(1-e^{-\lambda \text{Δ}}\right)}^{K_{r}}{\left(e^{-\lambda \text{Δ}}\right)}^{A_{r}} & \text{if}\;r\in {𝓣}_{N,{\underline{d}}_{r}}\\ 0 & \text{otherwise} \end{array}$$

where

(3.12) $$K_{r}=\sum_{n=1}^{N-1}r_{n}\;A_{r}=\sum_{j=1}^{K_{r}+1}\max\left\{0,M_{j}-\left(M_{j-1}+1\right)-2\left({\underline{d}}_{r}-1\right)\right\},\;M_{r}=\left\{n:r_{n}=1\right\}\cup \{0,N\},$$

and we adopt the convention that $M_{r}$ can be written as the ordered set $M_{r}=\left(M_{0},M_{1},\ldots ,M_{K_{r}+1}\right)$. Note that when ${\underline{d}}_{r}=1$, then $A_{r}=(N-1)-K_{r}$ which agrees with the prior when the switch point process is a discrete approximation of a Poisson process.

We capture uncertainty in the switch point rate, *λ*, by placing a prior distribution on *λ*. Specifically, we assume that

(3.13) $$\lambda ∼\text{Gamma}\left({\underline{a}}_{\lambda },{\underline{b}}_{\lambda }\right)$$

where ${\underline{a}}_{\lambda }$ and ${\underline{b}}_{\lambda }$ are hyper-parameters chosen to express prior belief about the rate at which change points occur. In the inference below, we set ${\underline{a}}_{\lambda }=(3/10)*50$ and ${\underline{b}}_{\lambda }=50$, so on average three changes occur within ten seconds.

We place an informative, compact prior on the segment speeds, $v_{j}$ for j=1,…k+1. Specifically, we set

(3.14) $$v_{j}∼\text{Uniform}\left(\left[-{\underline{\text{v}}}_{\max},{\underline{\text{v}}}_{\max}\right]\right)\;\text{for}\;j=1,\ldots k+1$$

where the hyper-parameter ${\underline{\text{V}}}_{\max}$ is the maximum speed a motor is believed to be able to travel within one time unit. Below we set this hyperparameter to be ${\underline{\text{V}}}_{\max}=2\;\mu \text{m}/\text{s}$. One advantage of such a prior is that we can obtain an explicit analytical expression for the marginal posterior distribution of (r,λ,η). There is also a conjugate prior for $v_{j}$, the Normal distribution, and it also yields an explicit analytical expression for p(r,λ,η∣ξ). However, when we used the non-compact prior for inference on experimental data, the result was an unrealistic number of inferred change points. This result is discussed in Section 5.1.

Because the common precision parameter *η* is phenomenological and there is little biophysical guidance to set a reasonable prior, we used an empirical prior. To this end, we constructed a Gamma prior from the uniformly minimum variance unbiased (UMVU) estimator of *η* assuming no change points,

(3.15) $$\eta ∼\text{Gamma}\left({\hat{\eta }}_{\text{umvu}}*0.15,0.1\right),$$

where

(3.16) $${\hat{\eta }}_{\text{umvu}}=\frac{\text{Δ}}{\text{Var}(\xi )},$$

and the values 0.15 and 0.1 were chosen so that expected common precision was slightly larger (1.5x) than the estimated value assuming no change points.

We provide a list of all the hyper-parameters for the sampling algorithm in Table 1.

#### 3.2.2. Target distribution for unknown number of change points

Before giving the explicit form of the posterior distribution for the given choices of priors, we introduce the following notation. The marginal likelihood of observing a vector ξ assuming uniform priors on the segment velocities is

(3.17) $$\begin{array}{l} L(\xi ;r,\eta )={\left(\frac{1}{2{\underline{\text{v}}}_{\max}}\right)}^{K_{r}}{\left(\frac{2\pi \text{Δ}}{\eta }\right)}^{-N/2}{\left(\frac{2\pi }{\eta }\right)}^{\frac{K_{r}}{2}}\exp\left(-\frac{\eta }{2\text{Δ}}S_{N}\left(M_{r}\right)\right)\\ \;\times \prod_{j=1}^{K_{r}}{\left(\frac{1}{\text{Δ}\left(M_{j}-M_{j-1}\right)}\right)}^{1/2}\left(\text{Φ}\left(\frac{{\underline{\text{v}}}_{\max}-{\bar{\xi }}_{\left(M_{j-1},M_{j}\right]}}{{\left(\eta \text{Δ}\left(M_{j}-M_{j-1}\right)\right)}^{-1/2}}\right)-\text{Φ}\left(\frac{-{\underline{\text{v}}}_{\max}-{\bar{\xi }}_{\left(M_{j-1},M_{j}\right]}}{{\left(\eta \text{Δ}\left(M_{j}-M_{j-1}\right)\right)}^{-1/2}}\right)\right), \end{array}$$

where *K*_*r*_ and $M_{r}$ are defined in Equation 3.12,

(3.18) $${\bar{\xi }}_{\left(M_{j-1},M_{j}\right]}=\frac{1}{M_{j}-M_{j-1}}\sum_{n=M_{j-1}+1}^{M_{j}}\xi_{n},\;S_{N}\left(M_{r}\right)=\sum_{j=1}^{K_{r}}\sum_{n=M_{j-1}+1}^{M_{j}}{\left(\xi_{n}-{\bar{\xi }}_{\left(M_{j-1},M_{j}\right]}\right)}^{2},$$

and Φ(z) is the cumulative distribution function of a standard normal random variable. The derivation of Equation 3.17 is provided in Appendix 7.1.

When $r\in {𝓣}_{N,{\underline{d}}_{r}}$, the model decisions above lead to the following posterior distribution

(3.19) $$p(r,\lambda ,\eta |\xi )\overset{C}{=}\left(\int_{\mathbb{R}}\ldots \int_{\mathbb{R}}L_{N,K_{r}}(\xi ;r,v,\eta )p\left(v_{1}\right)\ldots p\left(v_{K_{r}+1}\right)dv_{1}\ldots dv_{K_{r}+1}\right)p(\eta )p(r|\lambda )p(\lambda )\;\;\overset{C}{=}L_{N,K_{r}}(\xi ;r,\eta )\times \eta^{0.15{\hat{\eta }}_{\text{umvu}}-1}e^{-0.1\eta }\times {\left(1-e^{-\lambda \text{Δ}}\right)}^{K_{r}}{\left(e^{-\lambda \text{Δ}}\right)}^{A_{r}}\times \lambda^{{\underline{a}}_{\lambda }-1}e^{-{\underline{b}}_{\lambda }\lambda }$$

where *K*_*r*_ and *A*_*r*_ are given in Equation 3.12. When $r\notin {𝓣}_{N,{\underline{d}}_{r}}$ then p(r,λ,η∣ξ)=0.

#### 3.2.3. Initialization of inferred model parameters

In our analysis, both *λ* and *η* were randomly generated from their respective prior distributions. Due to the complex parameter landscape, we took more care in initializing the switch point process. To do this, we first sampled the number of change points, *k*_0_, from a Poisson distribution with parameter $\frac{{\underline{a}}_{\lambda }}{{\underline{b}}_{\lambda }}T_{\text{final}}$, where *T*_final_ is the duration of the tracked particle. Then assuming there are *k*_0_ change points, we initialized the change points to those estimates obtained by the existing change point detection method of constructing a piecewise-linear approximation to the data and minimizing the RSS.

#### 3.2.4. Sampling the unknown model parameters

For the Metropolis-Hastings (MH) sampling algorithm, we used different strategies for the different parameters. In this section we denote the proposed parameter value and the current parameter value by a superscript ‘prop’ and ‘cur’, respectively.

For the change point rate, *λ*, we employed an independence sampler, i.e., a proposal function that does not depend on the current value:

(3.20) $$q_{\lambda }\left(\lambda^{\text{prop}}|\lambda^{\text{cur}}\right)=q_{\lambda }\left(\lambda^{\text{prop}}\right)∼\text{Gamma}\left({\underline{a}}_{\text{prop}},{\underline{b}}_{\text{prop}}\right),$$

where ${\underline{a}}_{\text{prop}},{\underline{b}}_{\text{prop}}$ are hyper-parameters.

For the precision parameter, we used a Normal random walk proposal function:

(3.21) $$q_{\eta }\left(\eta^{\text{prop}}|\eta^{\text{cur}}\right)∼\text{Normal}\left(\eta^{\text{cur}},{\underline{\sigma }}_{\eta }^{2}\right),$$

where ${\underline{\sigma }}_{\eta }^{2}$ is a tuning parameter. Note that a negative proposal will be rejected with probability one because it is outside the support of the prior on *η*.

For the switch point process, we adopted the proposal function created by Green [44] and used in [41, 43]. The proposal function takes its values on the set ${\left\{0,1\right\}}^{N-d_{r}-1}$, where *N* is number of observations. At each step, one of three types of proposals might be made: (1) an independent switch point process; (2) a process generated by the creation or extinction of one of the change points; or (3) a location shift of a single change point. These proposal types occur with probability, ${\underline{u}}_{1}$, ${\underline{u}}_{2}$, and $1-{\underline{u}}_{1}-{\underline{u}}_{2}$, respectively. Details can be found in the SI Section 1. We provide a pseudo-code for our algorithm in SI Algorithm 1.

#### 3.2.5. Output of the algorithm for unknown number of change points

Our proposed MH sampler results in posterior samples of the switch point process, the switch point rate, and the common precision. We use the posterior samples to approximate the posterior distribution of the total number of change points. Suppose there are *M* posterior samples and let the number of change points in the *m*th switch point configuration be given by $K_{r}^{\left(m\right)}=\sum_{n=1}^{N-1}r_{n}^{\left(m\right)}$, then by ergodic theorem

(3.22) $$\frac{1}{M}\sum_{m=1}^{M}1\left(K_{r}^{\left(m\right)}=k\right)\overset{\text{a.s}}{\to }\mathbb{P}\left(K_{r}=k|\xi \right)$$

as *M* goes to infinity. Our posterior estimate for the number of change points is taken as the number with the highest posterior probability, denoted ${\hat{k}}_{\text{MAP}}$.

### 3.3. Bayesian inference for model parameters given a number of change points

In this section, we describe the MCMC sampling algorithm used to infer the change point times, the segment velocities, and the common precision when the number of change points, *k*, is known. Our model, Equation 3.6, naturally lends itself to the methodology proposed in [45, 46] to infer $\theta_{k}=\left(\tau_{k},v_{k+1},\eta \right)$. Namely, our assumption that the change points occurs at an observation time, or can be approximated by an observation time, yields conditionally independent segments and Normal data allows us to exploit the form of the full conditional posterior distributions through a Gibbs sampler for the following target distribution:

(3.23) $$p\left(\tau_{k},v_{k+1},\eta |\xi \right)\overset{C}{=}L_{N,k}\left(\xi ;\tau_{k},v_{k+1},\eta \right)p\left(\tau_{k},v_{k+1},\eta \right)$$

where $p\left(\tau_{k},v_{k+1},\eta \right)$ is the joint prior distribution of the model parameters and $\overset{C}{=}$ indicates equality up to a constant. Because we assume a model with *k* changes, we drop the subscript *k* and *k* + 1 on $\tau_{k}$ and $v_{k+1}$, respectively in the following section.

#### 3.3.1. Prior selection

For $v_{j}$ for j=1,…k+1, we employ the same priors as described in Section 3.2.1, Equation 3.14. We still model our prior belief in the common precision as a Gamma random variable as in Step 1, but update the hyper-parameters to reflect the information learned in Step 1. Let ${\hat{\eta }}_{\text{MAP},1}$ denote the maximum a posterior (MAP) estimator of *η* obtained from the posterior samples of Step 1, then the prior for *η* is given by

(3.24) $$\eta ∼\text{Gamma}\left(0.1*{\hat{\eta }}_{\text{MAP},1},0.1\right).$$

These choices in hyper-parameters result in Gamma prior with large spread and mean at the MAP estimator from Step 1.

Recall, in Step 1 we impose that each segment must have at least $\underline{d}$ observations, so that the change point times satisfy

(3.25) $$\begin{array}{c} \underline{d}\text{Δ}\leq \tau_{1}<\left(M_{2}+\underline{d}-1\right)\text{Δ}\\ \left(M_{j-1}+\underline{d}\right)\text{Δ}\leq \tau_{j}<\left(M_{j+1}+\underline{d}-1\right)\text{Δ}\\ \left(M_{k-1}+\underline{d}\right)\text{Δ}\leq \tau_{k}<(N-\underline{d}+1)\text{Δ} \end{array}$$

where $M_{j}=\left\lfloor \tau_{j}/\text{Δ}\right\rfloor$ is the change point index. To keep consistent between steps, we use a uniform prior constrained by the inequalities given in Equation 3.25 for the change point times. By choosing $\underline{d}=1$, we retrieve the uniform (non-informative) prior used in [46].

#### 3.3.2. Sampling the unknown model parameters

To obtain posterior samples for *ν* we perform component-wise sampling of $v_{j}|\left\{\xi ,\eta ,M_{j-1},M_{j}\right\}$ for j=1,…k+1. For the *j*th segment velocity, we use a normal random walk to propose a new value of *ν*_*j*_,

(3.26) $$q\left(v_{j}^{\text{prop}}|v_{j}^{\text{cur}}\right)∼\text{Normal}\left(v_{j}^{\text{cur}},{\underline{\epsilon }}_{v}^{2}\right)$$

where ${\underline{\epsilon }}_{v}$ is a tuning parameter. In the below inference, we used the value of ${\underline{\epsilon }}_{v}=1/2$.

For the precision parameter, our use of a conjugate prior enables direct sampling from the conditional posterior distribution,

(3.27) $${\eta |}_{\left\{\xi ,v,M\right\}}∼\text{Gamma}\left(0.1*{\hat{\eta }}_{\text{MAP},1}+\frac{N}{2},0.1+\frac{1}{2\text{Δ}}\sum_{j=1}^{k}\sum_{i=M_{j-1}+1}^{M_{j}}{\left(\xi_{i}-v_{j}\text{Δ}\right)}^{2}\right).$$

We obtain a sample of τ by component-wise sampling, $\tau_{j}|\left\{v_{j-1},v_{j},\eta ,\tau_{j-1},\tau_{j+1}\right\}$ for j=1,…k using the following proposal function for *τ*_*j*_, $q\left(•|\tau_{j}^{\text{curr}}\right)$,

(3.28) $$q\left(\tau_{j}^{\text{prop}}|\tau_{j}^{\text{curr}}\right)∼\text{Uniform}\left(\tau_{j}^{\text{curr}}-{\underline{\epsilon }}_{\tau },\tau_{j}^{\text{curr}}+{\underline{\epsilon }}_{\tau }\right)\;\text{for}\;j=1,\ldots k\text{,}$$

where ${\underline{\epsilon }}_{\tau }$ is a tuning parameter. In the inference below, we set ${\underline{\epsilon }}_{\tau }=\left\lceil {\left(1+k\right)}^{-2}*N\right\rceil *\text{Δ}$, where *N* is the path length. We note that our proposed values of the change point indices are continuous rather than discrete as to allow for better estimation of *τ*.

We assessed the convergence of this sampling algorithm to the target distribution using the potential scale reduction, $\hat{R}$, as given in [47] and the effective number of independent samples, ${\hat{n}}_{\text{eff}}$, as defined in [42]. One concludes that there is evidence for convergence if all inferred model parameters satisfy the two constraints

(3.29) $$\hat{R}<1.1\;\text{and}\;{\hat{n}}_{\text{eff}}>5\times (\text{\#}\;chains).$$

All the above hyper-parameters and tuning parameters can be found in Table 2 and a pseudo code for the above sampling algorithm is described in Algorithm SI-2. For brevity in Algorithm SI-2, we denote the posterior shape and rate of *η* given in Equations 3.27 by α(ξ,v,M), and β(ξ,v,M), respectively.

### 3.4. Posterior estimates for quantities of interest

As defined in [10], we use the term **run length** to denote “distance a molecular motor moves before detaching from the filament and diffusing away.” The **run time** is the time the molecular motor remains on the filament before detaching and diffusing away. When the cargo displays multiple physical states, we define the *j*th segment as the time between the $\tau_{j-1}$ and $\tau_{j}$ change point. We define a **segment duration** to be the time the motor stays in the *j*th state $\tau_{j}-\tau_{j-1}$, and **segment speed** to be the average speed of the motor during the segment.

#### 3.4.1. Posterior point estimates

We used a maximum a posteriori (MAP) estimator for our point estimates of the number of change points, the change points, the segment velocities, and common precision. To estimate the number of change points, we calculated an empirical marginal distribution from posterior distribution samples as described in Equation 3.22. The posterior samples were obtained by merging four independent runs of Step 1 samplers, each for 200,000 iterations with the first half discarded as burn-in and then thinned to every 100th sample. Then the posterior point estimate for the number of change points was set to the number of change points with the highest posterior probability, denoted ${\hat{k}}_{\text{MAP}}$. For the segment velocities, change points, and common precision, we set the posterior point estimates to the respective component of the MAP estimate over the full posterior distribution, Equation 3.23. Posterior quantities of the full distributions were obtained using the posterior samples of four independent runs of the Step 2 samplers, each of 40,000 iterations with the first half discarded and thinned to every 50th sample.

#### 3.4.2. Velocity distributions accounting for segment duration

To examine the heterogeneity of velocities within families of molecular motors and across families of molecular motors, we construct and compare posterior velocity distributions that account for the amount of time the motor spends in a given segment. For a given data set the velocity distribution is constructed as follows. For each tracked path, we set the duration of each segment using the posterior point estimate described in Section 3.4.1 for the change point times. Then, we draw posterior samples of the segment velocities, where the number of samples drawn equals the number of observations within each segment, i.e. if a path remains in the *j*th segment for ten observations, we draw ten samples from the posterior distribution of the *j*th velocity. Finally, to obtain the velocity distribution for the given data set, we pool together the posterior velocity samples from the previous step over all paths in the data set. Because we do not the polarity of the microtubules in practice, we take the absolute value of the velocity samples and construct speed distributions.

#### 3.4.3. Quantifying transitions between biological states

In order to quantify switches among biophysical states, we defined three categories: Initial, Paused, and Reversed, then assigned each segment to one of these categories. Then, using these assignments, we estimated the 1-step transition probabilities. We defined the three biological states in terms of the estimated segment velocity and the distance traveled in the segment, referred to as segment distance. Without the inclusion of the segment distance, a segment that has a slow but processive motor-cargo complex can be misclassified as paused. Because we did not have information concerning the direction of the plus- and minus- ends of the MT, we considered the initial direction as given by sign (${\hat{v}}_{0}$), where ${\hat{v}}_{0}$ is the MAP estimate of the first segment velocity that is considered processive, and the reverse direction as the opposing sign. The three state definitions are for segment *j* are:

- Initial: movement in the initial direction ($\text{sign}\left({\hat{v}}_{j}\right)=\text{sign}\left({\hat{v}}_{0}\right)$ and $\left|{\hat{v}}_{j}\right|\geq 0.1$) or ($\text{sign}\left({\hat{v}}_{j}\right)=\text{sign}\left({\hat{v}}_{0}\right)$ and $\left|{\hat{v}}_{j}\right|<0.1$ and segment distance ≥ 0.4*μ*m),
- Paused: no directed movement, ($\left|{\hat{v}}_{j}\right|<0.1$ and segment distance < 0.4*μ*m),
- Reversed: movement in the direction opposite of the initial segment, ($\text{sign}\left({\hat{v}}_{j}\right)\neq \text{sign}({\hat{v}}_{0})$ and $\left|{\hat{v}}_{j}\right|\geq 0.1$) or ($\text{sign}\left({\hat{v}}_{j}\right)\neq \text{sign}\left({\hat{v}}_{0}\right)$ and $\left|{\hat{v}}_{j}\right|<0.1$ and segment distance ≥ 0.4*μ*m).

The threshold values 0.1*μ*m/*s* and 0.4*μ*m were chosen based on an informal analysis of segment durations and velocities. A scatter plot for each of the data sets is shown in Figure SI-1. We found that for the single motor data sets, there were no segments with a velocity of less than 0.1*μ*m/*s* that nevertheless had an overall displacement of at least 0.4*μ*m. However, in the two-motor data set, there were multiple such segments, and to the eye, these appeared to show consistent slow movement over a long period of time. Because the behavior is not a statistical artifact, we included this type as an “active” segment. We conducted our analysis using 0.2*μ*m/*s* and 0.5*μ*m and all reported qualitative conclusions held for that choice as well.

### 3.5. Pre-processing of the molecular motor data

Before applying the described statistical methods, we pre-processed the data as follows.

#### 3.5.1. Change of coordinates to longitudinal position

The model we proposed for the position of the cargo in Section 3.1.2 and used in our Bayesian inference method is one dimensional, whereas the experimental cargo data is two dimensional, cartesian coordinates. Because we are interested in the position of cargo along (parallel to) the MT, we transformed the *x*,*y*-position data into longitudinal (motion parallel to the MT) position as follows. We assume the variation in the *x*- and *y*-directions are independent and identically distributed, and used orthogonal least squares regression to approximate the position of the MT. Then we consider the dynamics of the cargo in terms of its projection along the length of the MT.

To be specific, for orthogonal regression, the sum of square residuals is minimized to obtain the coefficients for the line of best fit, ${\hat{\beta }}_{0}$ and ${\hat{\beta }}_{1}$, and the predicted *x*-position, ${\hat{x}}_{n}$:

$${\hat{\beta }}_{0}=\frac{s_{yy}-s_{xx}+\sqrt{{\left(s_{yy}-s_{xx}\right)}^{2}+4s_{xy}^{2}}}{2s_{xy}}$$

$${\hat{\beta }}_{1}=\bar{y}-{\hat{\beta }}_{1}\bar{x}$$

$${\hat{x}}_{n}=x_{n}+\frac{{\hat{\beta }}_{1}}{{\hat{\beta }}_{1}^{2}+1}\left(y_{n}-{\hat{\beta }}_{0}-{\hat{\beta }}_{1}x_{n}\right)\;\text{for}\;n=1,\ldots N,$$

where $\bar{x},s_{xx}$ is the sample mean and sample standard deviation of *x*, respectively, $\bar{y},s_{yy}$ is the sample mean and sample standard deviation of *y*, respectively, and *s*_*xy*_ is the sample covariance of *x* and *y*. The predicted *y*-positions are ${\hat{y}}_{n}={\hat{\beta }}_{0}+{\hat{\beta }}_{1}{\hat{x}}_{n}$ for n=1,…N. Then, for n=1,…N, we rotate the predicted positions (${\hat{x}}_{n},{\hat{y}}_{n}$) onto the *x*-axis and set the longitudinal position to the rotated *x*-position

$$X_{n}^{∥}=\cos(\beta )\left({\hat{x}}_{n}-{\hat{x}}_{1}\right)-\sin(\beta )\left({\hat{y}}_{n}-{\hat{y}}_{1}\right)$$

where −*β* is the angle between the *x*-axis and the vector from the origin to $\left({\hat{x}}_{2}-{\hat{x}}_{1},{\hat{y}}_{2}-{\hat{y}}_{1}\right)$. For those tracked paths that appear to be stepping on a non-linear microtubule we remove the path from the following analysis.

This process is depicted in the the left and center frame of Figure 2. In the left frame the observed trajectory of a cargo tethered to a Kinesin1-DDB motor complex is shown in black and the estimated position of the microtubule is shown in purple. The black curve in the center frame represents the longitudinal position of the trajectory when projected onto the microtubule.

#### 3.5.2. Handling missing data points

An assumption in our inference method that is often violated in practice is observation at uniform time increments. One such cause is that the tagged cargo becomes too dim to track for a couple of frames due to a change in the *z*-position. We opt to fill in the missing data points using the longitudinal position for a maximum of twenty consecutive missed points, although the maximum number of consecutive missed points for the data presented above in Section 2 was eight. If more than the maximum allowed number of consecutive points are missing, we keep the trajectory up until the period of max missed number of observations.

In the case of *r* sequential missing positions we estimate the missing observations by the uniformly spaced positions between the previous and last observed position with some noise. For instance, if the longitudinal position at the (*n* + 1)th time observation, $t_{n+1}$, to the (*n* + *r*)th time observation, $t_{n+r}$ are missing, we estimate $X_{n+i}^{∥}$ for i=1,…r by

(3.30) $${\hat{X}}_{n+i}^{∥}=X_{n}^{∥}+\frac{i}{r+1}\left(X_{n+r+1}^{∥}-X_{n}^{∥}\right)+\epsilon Z$$

where *Z* ~ Normal(0,1) and is chosen so qualitatively the missing points looks similar to the rest of the path. In the below analysis, we set $\epsilon^{2}=var\left(X^{∥}\right)/10$, which in some cases is an underestimation of the true variance. This can potentially lead to a bias in the over-estimation of the length of states.

We display an example of this procedure in the center frame of Figure 2. The black line is an observed longitudinal trajectory of a tracked cargo tethered by a Kinesin1-DDB motor complex and the three red circles correspond to estimated missing observations as given by Equation 3.30. In the right frame of Figure 2, we display the increment process of the longitudinal trajectory in black and mark the increments that have been calculated with estimated missing observations with red circles.

## 4. Numerical methods for validation of the protocol

In this section, we provide an experimental design for validating inference methods for the number of change points in molecular motor data. This entails constructing simulated data sets of the observed cargo data in which the number of change points is known. First in Section 4.1, we describe the simulation technique for generating 1D molecular motor and cargo processes from the biophysical model, Equations 3.1 and 3.2. Section 4.2 gives a model for the experimental cargo data that arises from the data collection method. Lastly, Section 4.3 outlines and describes the construction of four simulated data sets that vary in complexity.

### 4.1. Simulating from the bio-physical model

We simulated motor-cargo processed from the biophysical model as follows. First, we obtained the motor paths using the Gillespie algorithm where the rate of the motor stepping changes at times $\tau =\left(\tau_{1},\tau_{2},\ldots \right)$. The simulation time grid for the motor positions, denoted ${\left\{s_{n}\right\}}_{n=1}$, is set to the ordered union of the uniform time mesh of size ${\text{Δ}}_{\text{sim}}$ and the change times ***τ***. Defining the time grid in this manner ensures that *Z*(*t*) only jumps at the right endpoint of ${\left\{s_{n}\right\}}_{n=1}$. After sampling the motor positions, the position of the cargo is determined for time *s*_*n*_ using the exact conditional solution to Equation 3.2

$${X\left(s_{n}\right)|}_{Z\left(s_{n-1}\right),X\left(s_{n-1}\right)}=e^{-\frac{\kappa }{\gamma }\left(s_{n}-s_{n-1}\right)}X_{n-1}+\left(1-e^{-\frac{\kappa }{\gamma }\left(s_{n}-s_{n-1}\right)}\right)Z_{n-1}\;\;+\sqrt{2D}\int_{s_{n-1}}^{s_{n}}e^{-\frac{\kappa }{\gamma }\left(s_{n}-s_{n-1}\right)}dW(u)$$

where the stochastic term is a normal random variable and using Ito’s Isometry

(4.1) $$\sqrt{2D}\int_{s_{n-1}}^{s_{n}}e^{-\frac{\kappa }{\gamma }\left(s_{n}-s_{n-1}\right)}dW(u)∼\text{Normal}\left(0,\frac{D}{\kappa /\gamma }\left(1-e^{-2\frac{\kappa }{\gamma }\left(s_{n}-s_{n-1}\right)}\right)\right).$$

The output of the algorithm is the trajectory of the motor position and the cargo position with respect to the simulation time grid ${\left\{s_{n}\right\}}_{n=1}$. Pseudo code for simulating the motor-cargo trajectory is given in Algorithm SI-3.

**Figure 3.**
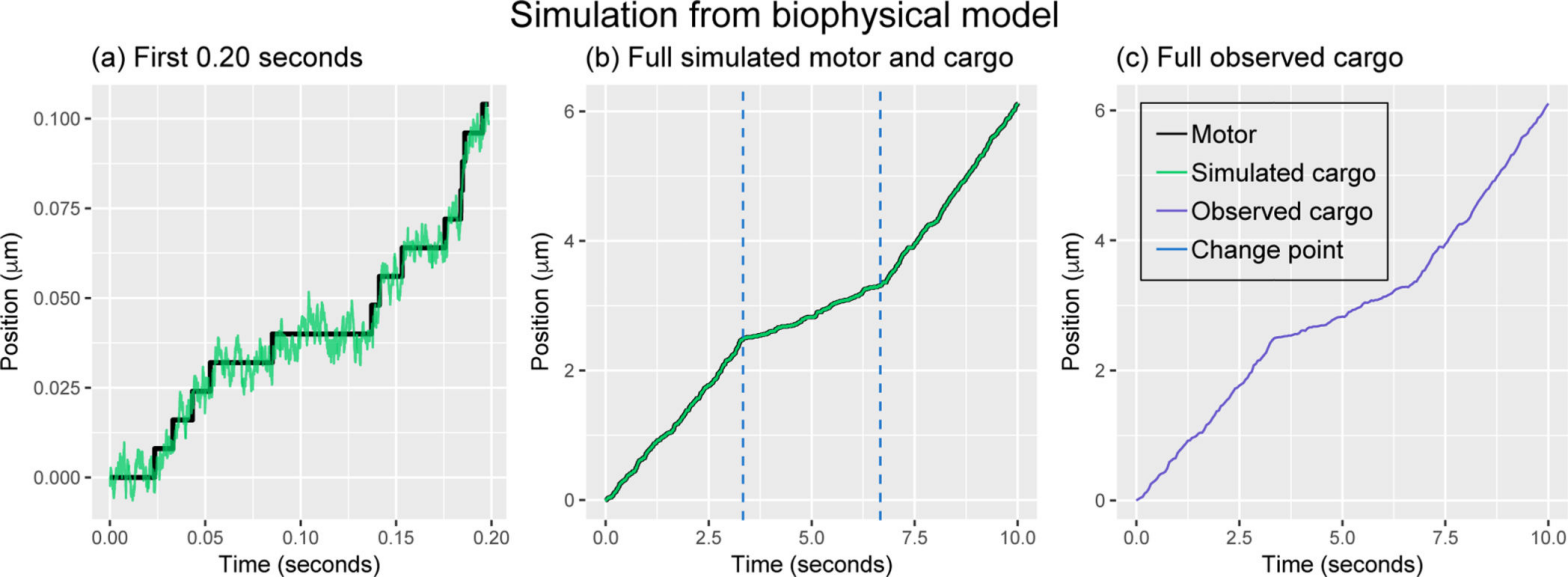
Simulated cargo-motor system from the biophysical model. **(a)**: Simulated motor and cargo process governed by the biophysical model presented in Section 3.1.1, on a short time scale of 0.2 seconds. The motor and simulated cargo position are colored in black, and green, respectively. **(b)**: The same simulated paths as in (a) but over the whole simulation time window of 10 second. The motors change stepping rate twice, marked by the dashed blue lines. **(c)**: The trajectory of the observable cargo over the whole simulation time window. Observations are assumed to occur once per every 0.05s.

In Figure 3(a),(b), we display a simulated motor (black) and cargo (green) process in which the stepping rate of the motor-cargo complex changes rate twice on a short time scale and long time scale, respectively. To generate the simulation in Figure 3, the simulation time grid was taken to be every ${\text{Δ}}_{\text{sim}}=10^{-4}s$, and the model parameters were set to

(4.2) $$\begin{array}{l} \text{Motor Parameters:}\;\rho =(100,30,100){\text{s}}^{-1},\delta_{\text{step}}=0.008\mu \text{m},\\ \text{Cargo Parameters:}\;\kappa /\gamma =1000,\;D=0.012\mu {\text{m}}^{2}/\text{s}. \end{array}$$

Alternatively, the average speed of the motor, |v| can be specified rather than the stepping rate, in which the stepping rate is computed as $\rho =|v|/\delta_{\text{step}}$.

### 4.2. Model for the observational error

We introduce a model for the experimental cargo data that arises from the data collection method. Position data of a tagged particle is collected at discrete time increments depend on the frame rate of the camera. Let Δ denote the time between observations, and ${\left\{t_{n}\right\}}_{n=0}^{N}$ be the collection of *N* + 1 observation times. For simplicity, we assume that observations are made on a uniform time grid, $t_{n}=n\text{Δ}$.

Due to limited spatial precision, the collected time-series data captures the position of the cargo plus some observational noise. Under the assumption that the observational error is independent at each measurement and normally distributed, our model for the observed process is given by

(4.3) $${\hat{X}}_{n}=X\left(t_{n}\right)+\sigma \epsilon_{n}$$

where $X\left(t_{n}\right)$ is the true position of the cargo, *σ* is the standard deviation of the observational error, and *ϵ*_*n*_ for *n* = 0,... *N* are i.i.d standard normal random variables.

The observed cargo process is obtained from the simulated cargo position, described in Section 4.1, by subsampling the simulated cargo position at times ${\left\{t_{n}\right\}}_{n=1}^{N}$, and adding Normal noise to each observation as given in Equation 4.3. For the simulated motor-cargo process in Figure 3(b) we depict the observed cargo process in Figure 3(c), where observations at taken uniformly every 0.05 seconds (Δ = 0.05) and σ = 0.003*μ*m.

### 4.3. Numerical experiment scenarios

To construct a simulated data set that emulates transport by multimotor complex Kin1-DDB, we work under the assumption that each simulated path is from the biophysical model, Equation 3.2, but the observed data is from Equation 4.3. We assume that a change in motor interaction does not result in a switch of direction, but rather the motor’s average velocity changes between a slow velocity, $v_{\text{slow}}$, distribution and fast velocity, $v_{\text{fast}}$, distribution. Equivalently, using the relation: $\lambda =|v|/\delta_{\text{step}}$, the motor changes between a distribution of low and high stepping rate, respectively. We further assume the number of changes follows a Poisson distribution.

In each simulation study, the strength of the change by altering the length of each segment or the relative difference in the segment velocities. For the former, the change points are either uniformly spaced in time so all segment durations are equal, or ordered uniformly distributed random variables so each segment duration is random. As for the latter, the amount of overlap, negligible or non-negligible, between the fast and slow distribution. The resulting four scenarios are:

- **Case 1:** change points are uniformly *spaced* and the distributions for the slow and fast velocities overlap on sets of negligible probability,
- **Case 2:** change points are uniformly *spaced* and the distributions for the slow and fast velocities are overlapping,
- **Case 3:** change points are uniformly *distributed* and the distributions for the slow and fast velocities overlap on sets of negligible probability,
- **Case 4:** change points are uniformly *distributed* and the distributions for the slow and fast velocities are overlapping.

Case 1 produces paths with the strongest change signal since each segment length is controlled and large velocity changes are probable, whereas Case 4 contains paths with the weakest signal since segments can be shorter than the frame rate and small velocity changes are more probable. Both Case 2 and 3 muddle change point detection, but ultimately the short segment durations (Case 3) makes changes more difficult to detect. We note there was no restriction that segments must be longer than the minimum segment cutoff *d*_*r*_ that appears in our NC-CI method. In Section 5.2, we report on the performance of the four methods in two ways. First, we looked at the accuracy of all methods directly on the simulated paths regardless of segment lengths. We do not expect that any of the methods should perform well in identifying extremely short segments. So, in the next analysis, we restricted our study to paths whose segments are all “meaningfully long” in the sense that they are longer than *d*_*r*_ observations.

#### 4.3.1. Selecting biological parameters for numerical experiments

For each case, we simulated 200 paths from Equation 4.3 with experimental parameters

$$\text{Δ}=0.05\;\text{s},T_{\text{final}}=10\;\text{s},\;\text{and}\;\sigma =0.003\mu \text{m,}$$

and physical parameters,

$$\delta_{\text{step}}=0.008\mu \text{m},D=0.01\mu {\text{m}}^{2}/s,\;\text{and}\;\kappa /\gamma =1000.$$

The remaining parameters: number of change points, time of change points and segment stepping rates varied from path to path.

For a single path, we first sampled the number of change points from Poisson (*λ* = 3). Then for Case 1 and 2 change points were uniformly spaced, while for Case 3 and 4 the change points were set to an ordered sample from Uniform((0, *T*_final_)). Lastly, we obtained the stepping rate for each segment through sampling the segment velocities and using the relation $\lambda =v/\delta_{\text{step}}$. For each segment velocity, we alternated drawing the velocity from two folded normal distributions with Case 1 and Case 3 parameters:

(4.4) $$v_{\text{slow}}∼\text{folded Normal}\left(0.1,0.05^{2}\right)\;v_{\text{fast}}∼\text{folded Normal}\left(0.6,0.1^{2}\right)$$

and Case 2 and Case 4 parameters:

(4.5) $$v_{\text{slow}}∼\text{folded Normal}\left(0.1,0.1^{2}\right)\;v_{\text{fast}}∼\text{folded Normal}\left(0.6,0.2^{2}\right),$$

as displayed in Figure SI-3.

#### 4.3.2. Selecting algorithm parameter for numerical experiments

We considered our proposed algorithm, NC-CI, along with the three other algorithms introduced in Section 1.1: (1) the PPM specified in [30] that has been implemented in R, referred to as BCP, (2) Bai and Perron’s dynamic programming algorithm minimizing the RSS, referred to as RSS, and (3) the likelihood ratio test method proposed by [32], referred to as LRT. For all four simulation studies, the hyperparameters for each considered method were kept the same.

For our proposed sampling scheme, the hyper-parameters were set to those given in the caption of Table 1. For each path we ran two independent Step 1 samplers with different initial values, each for 200,000 iterations, discarding the first 100,000 samples as the burn-in period, and keeping every 100th sample. The remaining posterior samples from each sampler were merged together and used for inference.

For BCP, there are two model parameters that need to be selected, the maximum probability of change occurring at an observation, and the maximum signal to noise ratio. While the default value for both parameters is 0.2, which has been shown to work well in general settings, [30, 49], we chose problem-specific values that increased the method’s accuracy. For the probability of a changepoint parameter, we used the form that emerges from modeling the switches as a Poisson process. We set the assumed the rate of changes to 4/10, which yields a hyper-parameter value of $1-e^{-(4/10)\text{Δ}}$. As for the maximum signal to noise ratio, we set the variance of the means in the PPM model to (1/2)^2^, which is consistent with when the Normal priors were tried in the NC-CI. We approximated the measurement variance by the assessing the residual error when fitting a spline to the time-longitudinal data. Note the latter results in an empirical prior rather than a true Bayesian prior. Such hyper-parameters are not necessarily optimal, but express our knowledge of the system and perform better then the default value. The default priors resulted in accuracy of 21.0, 18.5, 5.5, and 3.5% in the Case 1, 2, 3, and 4 respectively. We ran the sampler for a total of 30,000, the first 10,000 as the burn-in period and the latter 20,000 as posterior samples.

As for the RSS method, we modeled the increment process as an intercept-only linear model with multiple structural changes and set the minimum segment length to 5, which is consistent with the hyper-parameter for the NC-CI method. For the LRT method, we approximated the critical value using $\hat{\mu }$, see [33, 32] value for *α* = 0.05.

## 5. Results

In this section, we report our findings on change point detection on both simulated and experimental molecular motor data. In Section 5.1 we present the results on change point detection when the conjugate prior is placed on segment velocities that led us to use the compact uniform priors in NC-CI. Section 5.2 compares the accuracy of four change point detection methods on the simulated case studies presented in Section 4.3. The results of our prososed NC-CI on the experimental data discussed in Section 2 is given in Section 5.3. To run our proposed method on both the simulated and real data, the High Performance Computing system at Tulane University was used.

### 5.1. Compact prior for segment velocities is necessary for successful model selection

A natural alternative prior for each segment velocity is a Normal distribution because it is a conjugate prior for our choice in likelihood. We encode prior knowledge about motor proteins into this prior by setting the prior mean to 0 and standard deviation to 1/2 so that a motor speed less or equal 1*μ*m/s occurs with 95% probability. When placing such unbounded prior on each of the segment velocities, and enforcing each segment had a minimum of ${\underline{d}}_{r}=5$ observations, we found most paths had a MAP estimate for the number change points near or at the upper bound on the number of change points. For example, within the DDB data set, there was a tracked cargo path with 283 observations. Visually, it has two apparent change points, but the method with normal (and hence, unbounded) prior estimated it to have 47 change points, shown in Figure SI-4. Such an estimate corresponds to each segment either be 6 observations (0.258 seconds) or 7 observations long (0.301 seconds). By contrast, the same DDB path was estimated to have 2 change points with the compact uniform prior, Figure SI-5.

**Figure 4.**
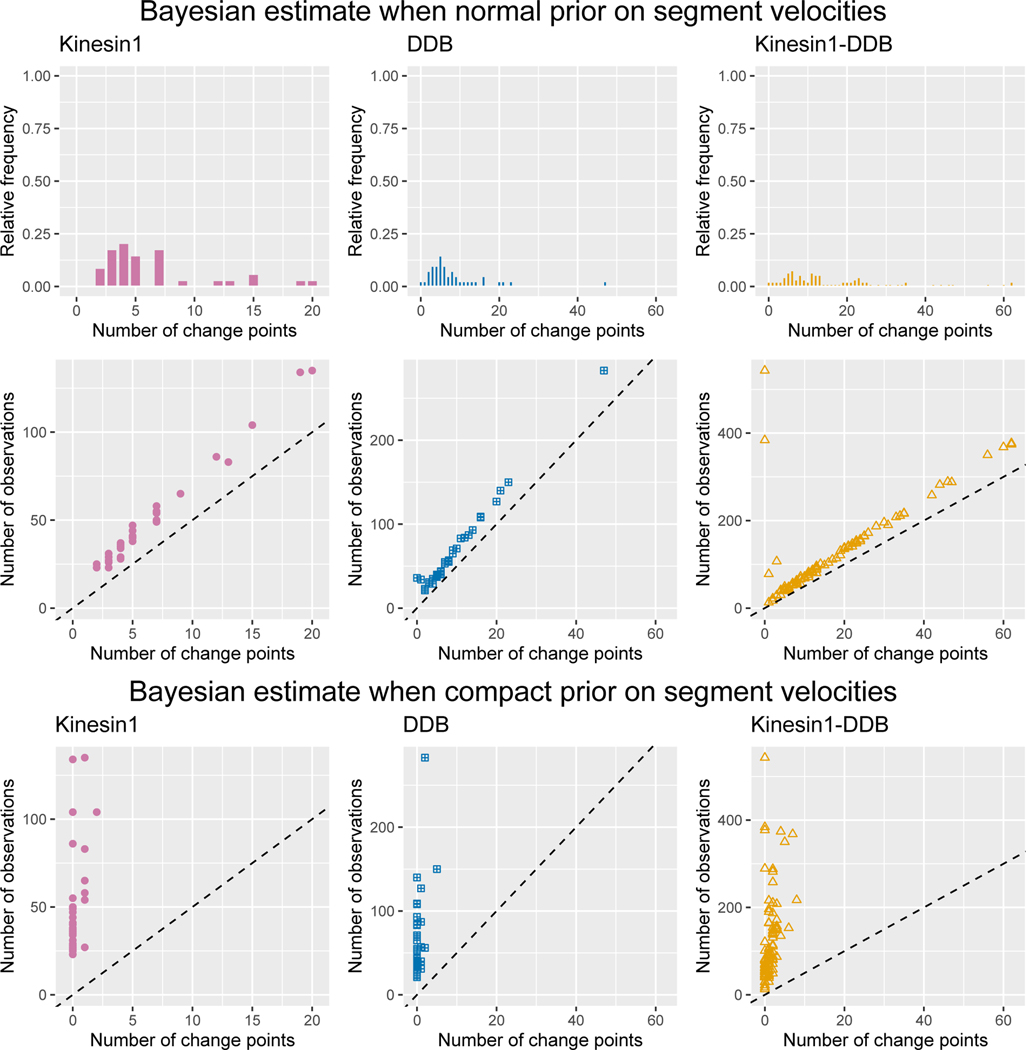
Unbounded (Normal) prior on segment velocities result in overestimation of number of change point. **Top row:** For Normal prior, the frequency of the MAP estimator for the number of change points within the Kinesin1, DDB, and Kinesin1-DDB data set from left to right, respectively. **Middle, bottom row:** For each tracked path, the number of observations versus the estimate for the number of change points (MAP) within the Kinesin1, DDB, and Kinesin1-DDB data set (left to right) when a Normal prior (middle row) and uniform prior (bottom row) is assumed. The black dashed line denotes the maximum number of allowed change points, which is equal to the number of observations/5).

In Figure 4, top row, for all three data sets we display the empirical distribution of the MAP estimator for the number of change points with a normal prior on the segment velocities. For comparison when a uniform prior on segment velocities is assumed see the first column in Figure 6. In the middle row of Figure 4 (left to right), we display the path length versus MAP estimate for the number of change point for each path in Kinesin-1, DDB, and Kinesin1-DDB data set, respectively. Within all data sets, a linear trend emerges between the number of change point estimate and the path length. The linear trend has a slope of roughly 6.3 for the Kin1 data set, 5.8 for the DDB data set, and 5.1 for the Kin1-DDB set, indicating each segment is closer to the minimum number of observations required by our choice in hyper-parameter, ${\underline{d}}_{r}=5$. These trends do not appear when a compact uniform prior is placed on the segment velocities. See the bottom row of Figure 4 for number of observations versus number of change points for each three data sets.

A possible explanation is that a large likelihood value arising from overfitting the data with many change points compensates for the low prior probability the Normal prior assigns to unrealistic segment speeds. This does not occur when using the uniform prior because it has finite support. We also conducted some preliminary analysis on simulated data assuming a Normal prior with mean zero and standard deviation 1/2 that we do not report here, which also resulted significant over-estimation.

### 5.2. Comparison of methods

We validated the first stage of our proposed method by comparing its accuracy with that obtained from using the three existing methods BCP, RSS, and LRT. First, we considered the accuracy of the four methods on the simulated molecular motor datasets described in Section 4.3. Then, we compared the estimates for the number of change points using the NC-CI and the BCP method on the three molecular motor data sets described in Section 2. The hyper-parameters of the alternative methods are given in Section 4.3.1.

#### 5.2.1. Results on simulated data sets

We estimated the accuracy of a method, with respect to the number of change points, as the fraction of paths with estimator for the number of change points equal to the true number of change points. For the Bayesian methods, NC-CI and BCP, the estimator is taken to be the MAP. Confidence intervals for the accuracy estimates are constructed assuming a normal approximation to the binomial distribution. We note that the analysis of the simulated data sets for the three existing methods can be run on a laptop, whereas the analysis by our proposed method is more computationally intensive and was run on a Tulane University’s high power computing system.

**Figure 5.**
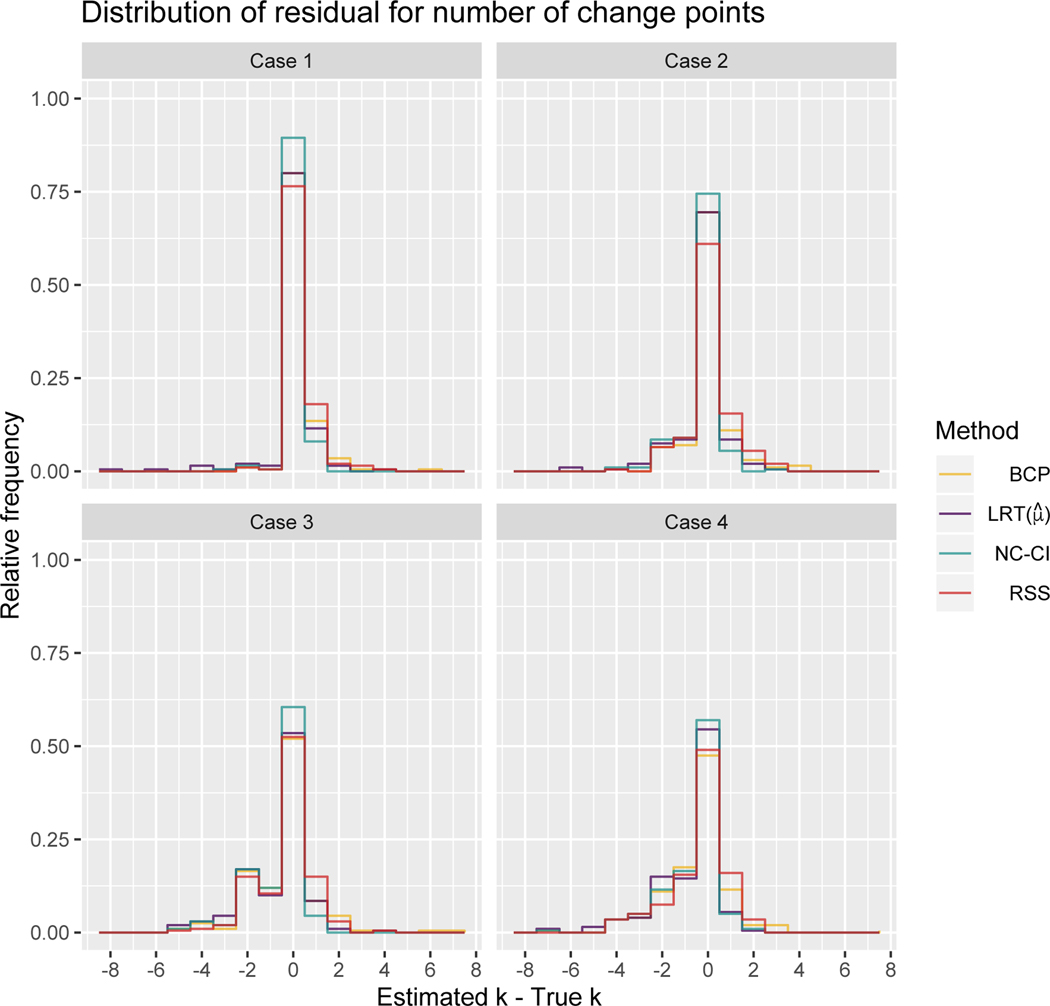
The distribution of the residual for the number of change points for the four simulation scenarios. Within a frame, the residual value is on the *x*-axis, the relative frequency of the residual is on the *y*-axis, and the method denoted by the color. The top (bottom) row consists of the cases with uniformly *spaced* (*distributed*) change points. The left (right) column consist of the cases with fast and slow velocity distributions that overlap with *negligible* (*significant*) probability.

For all four simulation studies, the NC-CI method had the highest accuracy, followed by the LRT method. Within increasing complexity of the the simulation study, less can be concluded about the significance of the improved accuracy when using the NC-CI method because the confidence intervals for all methods overlap. In Table 3, we give the accuracy and the 95% confidence intervals for the point estimate for each case and all four methods. A graphical display of the accuracy of each method is shown in Figure 5. Each frame displays the distribution of the residual for the number of change points, $\hat{k}-k_{\text{true}}$ with the residual value on the *x*-axis, the relative frequency on the *y*-axis, and the color denoting the method.

As the change point signal becomes harder to detect, going from Case 1 to Case 4, the accuracy of estimating the number of change points *k* decreases for all methods but the LRT, which has a similar accuracy for the hardest two cases, Case 3 and 4. For all methods, the frequency of under-estimation systematically increasing as to be expected due to weaker change point signals. This can be seen qualitatively in Figure 5, where the left tail of the residual distribution of the number of change points for all methods, grows as the case number increases.

Furthermore, for our choices in overlap between the fast and slow velocity distributions having uniformly distributed segment durations has a more negative impact on change point detection. This can be seen by comparing the residual distributions of each method across columns in Figure 5, as compared to those across the rows in Figure 5. There is a larger decrease in accuracy going from Case 1 to Case 3 as compared to Case 1 to Case 2, and going from Case 2 to Case 4 as compared to Case 3 to Case 4. Note the cases in the top row of Figure 5 have uniformly spaced change points whereas the bottom row has uniformly distributed change points, and the cases in the left column have negligibly overlapping velocity distributions, as opposed to the cases in the right column have overlapping velocity distributions.

#### 5.2.2. Analysis restricted to subset of simulated paths with ‘meaningful’ changes

The success of the NC-CI method is particularly strong when we consider only those paths whose segments are biophysically meaningful, in the sense that each true segment duration lasts at least 5 observations and true sequential velocity changes are at least 0.1*μ*m/*s* in magnitude, $\left|v_{i}-v_{i+1}\right|\geq 0.1$ for all i=1,…k. This is to be expected since, at this point, we are only considering those paths that have a segment duration greater than or equal to selected hyperparameter value for the minimum segment duration, *d*_*r*_ = 5. The increase in accuracy is larger for Case 3 and Case 4 as the former condition is violated more when the change points are uniformly distributed as opposed to uniformly spaced as in Case 1 and Case 2. Specifically, for Case 1 and Case 2 none of the paths violate the condition on the segment length, whereas 34/200 and 32/200 paths violate the condition on the segment length for Case 3 and Case 4 respectively. As for the condition on the magnitude of the velocity change, none of the paths in Case 3 violate the condition whereas 1/200, 14/200, and 19/200 paths violate the condition for Case 1, Case 2, and Case 4, respectively. The accuracy for the subset of meaningful paths are given in Table 4.

#### 5.2.3. Comparing estimates for the number of change points using NC-CI and BCP method

For the Kin-1 and DDB data sets, the estimate for number of change points using the NC-CI and BCP method were in good agreement, whereas the Kin1-DDB data set had more discrepancies between the two methods. In Table 5, we report the fraction of paths whose NC-CI estimate for the number of change points was equal, less than, and greater than that when using the BCP method.

For the data sets with a single motor, when there was a discrepancy between the two estimates, typically the NC-CI was higher than the BCP estimate. In about half the cases NC-CI detected a single change either towards the beginning or end of the path, whereas BCP detected no changes.

We found the converse results for the multi-motor data set: when the two estimates disagreed, the BCP estimate was typically larger that the NC-CI estimate. We attribute this to the Kin1-DDB data set having more paths with large spikes in the increment process only lasting for a few observations, either the stochasticity of the motor stepping, or tracker error. The BCP marked such short lived spikes as change points whereas the NC-CI did not due to the assumption of the minimum state duration of at least five observations.

### 5.3. Characterization of motor velocities within a family of motor proteins

We found that Kinesin-1 had more stereotypical velocities, whereas there was heterogeneity of velocity for the DDB and Kinesin1-DDB complexes.

#### 5.3.1. Kinesin-1 as a processive motor

**Figure 6.**
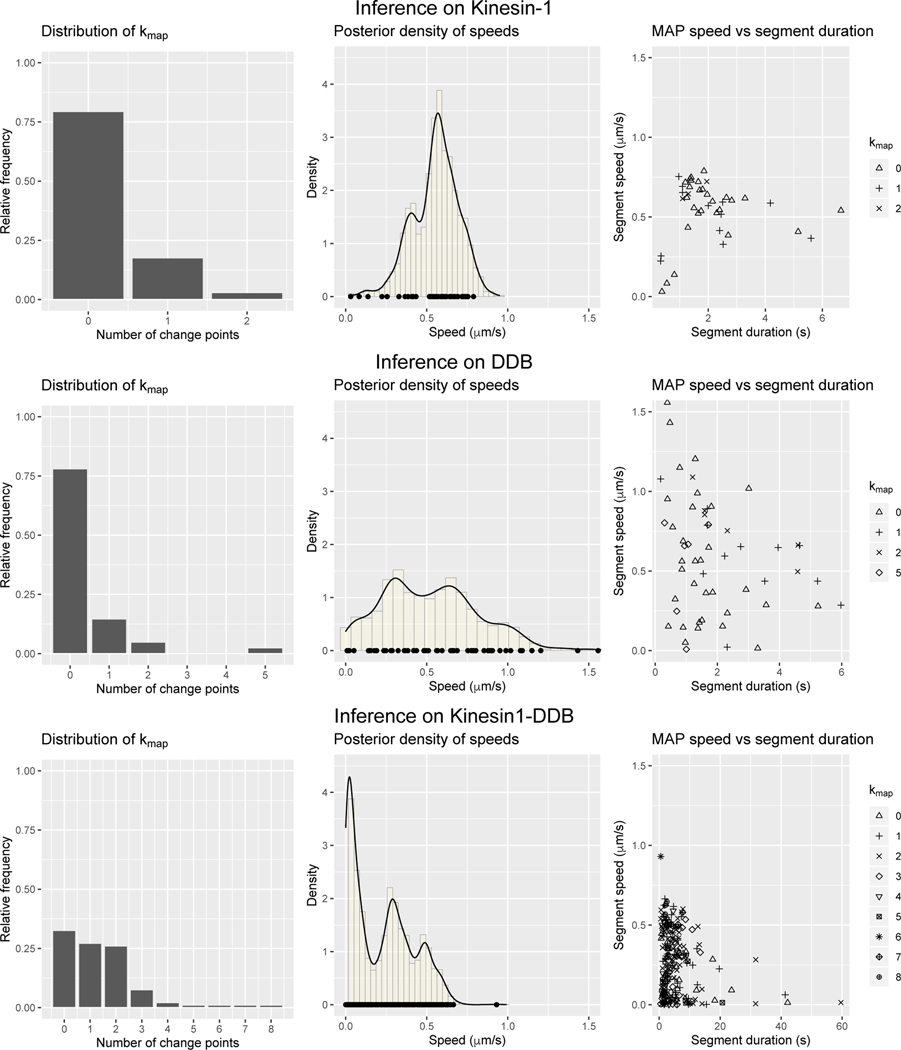
**Top, middle, bottom row:** Reported inference on the Kinesin-1, DDB, and Kin1-DDB data sets. **Left column:** The empirical distribution of the MAP estimator for the number of change points, ${\hat{k}}_{\text{MAP}}$. **Center:** The empirical posterior density of speed of all the tracked cargo weighted by the duration of each state described in Section 3.4.2. Points on the line *y* = 0 denote the posterior point estimate for each segment speed. **Right:** The MAP estimate for the *j*th speed versus that of the duration of the *j*th state, where the MAP is over the joint posterior distribution, Equation 3.23. The symbol corresponds to the number of changes points, ${\hat{k}}_{\text{MAP}}$, for the given path. Note that *x*-axis scales vary between rows for right column.

We found that 79.41% (27/34) of the tracked cargo had a MAP estimator for the number of change points as zero, top left frame of Figure 6. At first glance having 17.64% (6/34) of paths with ${\hat{k}}_{\text{MAP}}>0$ seems contradictory to Kinesin-1 being known as a processive motor [37, 15, 38]. Through path-bypath analysis of the paths with ${\hat{k}}_{\text{MAP}}=1$, we found four out of the six had a change point estimated towards the end of the trajectory in which the motor protein became less processive. A possible explanation is that the motor reached the end of the MT and remained there before detaching. Moreover for the single path with ${\hat{k}}_{\text{MAP}}=2$ visually appears to transition from a processive state to a paused state and back to a processive state, resulting in two change points.

While there was some variation in the posterior samples of the speed over all Kinesin-1 trajectories, the samples were concentrated ≈ 0.57*μ*m/s, top center frame of Figure 6. The MAP estimator for each speed segment is denoted by the black circle on the *x*-axis. There appears to be a slight a correlation between duration of each state and the speed of the cargo during the given state, top right frame in Figure 6. For states only lasting a short duration (bottom left points), there appears to be a decrease in the MAP estimator for the speed.

#### 5.3.2. Heterogeneity within DDB velocities

Overall, we found that a majority of the paths were estimated as having zero change points, 78.05% (32/41) of the paths. This finding agrees with the experimental results presented in [39], in which trajectories of cargo transported by DDB appeared as diagonal lines. However, there was a significant percentage, 21.95%, of paths with least one change point was detected, middle row left frame of Figure 6.

We found more variation in the posterior samples of the segment speed for those tracked cargo tethered to DDB, middle row center frame of Figure 6, as opposed to those tethered to Kinesin-1. The empirical posterior distribution appeared to be possibly be tri-modal with peaks around 0.4, 0.6, 0.1*μ*m/s from largest to smallest. More data is required to make a claim the multi-modality is statistically significant. Moreover, the empirical posterior distribution was supported on speeds larger than 1*μ*m/*s*, a region within the support of our uniform prior but a less biologically plausible region, and 7 of the 54 MAP estimates of the segment velocities were larger that 1*μ*m/s. In examining these paths, the segment with velocity larger than 1*μ*m/*s* in magnitude had a low number observations per state, ranging between 5 and 30, (see SI-DDB particle 1, 8, 18, 20, 24, and 39).

#### 5.3.3. Heterogeneity within Kinesin1-DDB velocities

The Kin1-DDB data had more challenging paths to analyze. We removed 9 out of 101 tracked particles because the trajectory was poorly captured by a single straight line. Either the microtubule was curved or bent, or the motor-cargo complex changed microtubule during the observation window, complicating tracking. There were fifteen tracked cargos with a low posterior probability of *k*, but after visual inspection, there were only two trajectories for which the number of change points and locations were unclear. These fifteen tracked paths are included in the below analysis. Furthermore, there were nine paths in which not all inferred parameters in Stage 2 had posterior samples satisfying the convergence criterion (SI-Kin1-DDB particle 8, 11,13, 26, 36, 41, 44, 74, 77). After visual inspection, we opted to include them in analysis below. Four of the nine paths had a large estimate for the number of change points (at least five). Whereas the remaining five had estimated number of changes between two and four, in which it is unclear if the number of changes is mis-estimated resulting in the convergence criterion not being satisfied.

We found 67.39% (62/92) of the paths were estimated as having at least one change, ${\hat{k}}_{\text{MAP}}>0$, as depicted in the empirical distribution of ${\hat{k}}_{\text{MAP}}$, bottom left frame of Figure 6. Qualitatively, the empirical posterior density of speed of the tracked Qdot-Kin1-DDB complexes weighted by the duration of each segment appeared to be tri-modal, with peaks around 0.05,0.3,0.5*μ*m/*s* (largest to smallest) bottom center frame of Figure 6. A possible explanation for the largest peak being ≈ 0.05*μ*m/*s* is the segments with longer duration had such a speed, bottom right frame of Figure 6. While there is a slight increase in run length and time tracked as ${\hat{k}}_{\text{MAP}}$ increases, there does not appear to be a correlation between run length nor time tracked and the MAP estimator for the segment speeds, bottom center and bottom right frame of Figure 6.

### 5.4. Comparison of motor velocities between motor families

Preliminary analysis qualitative showed that two motor complexes, Kinesin1-DDB complexes have longer run lengths and run time compared to the single motor complexes. Although there is an imbalance in the number of tracked particles for each type of motor, the median run length and amount of tracked time is largest for the Kin1-DDB data set. This suggests that cargo tethered to Kin1-DDB remain bound to the microtubule longer than when the cargo is only tethered to a single motor.

**Figure 7.**
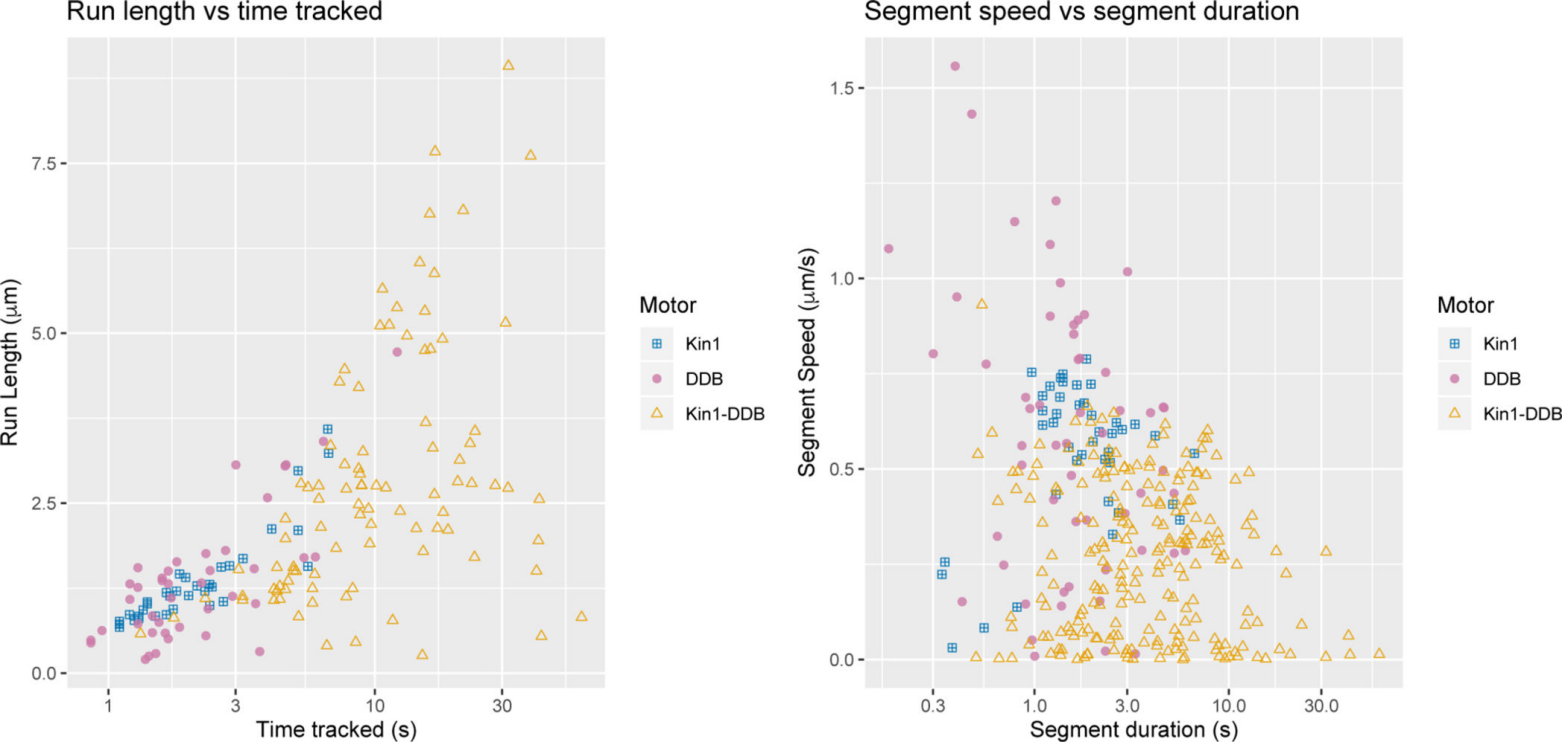
**Left:** Run length versus time tracked on a log 10 scale where the color and style of the point corresponds to the type of motor protein. **Right:** Segment speed versus segment duration on a log10 scale where the color and style of the point corresponds to the type of motor protein.

For all tracked cargo, we display the run length versus time tracked on a log10 scale for each cargo in the left panel of Figure 7, where a dot corresponds to a tracked cargo and the color and shape denotes the motor protein complex. Taking the corresponding MAP estimators obtained in Step 2 of our algorithm, we found on average the Kin1-DDB tracked particles had slower velocities but longer segment durations. We display the segment speed versus segment duration on a log10 scale for each cargo in the right panel of Figure 7, where a dot corresponds to a tracked cargo and the color and shape denotes the motor protein complex.

### 5.5. Transitions among biophysical states: reversals often involve paused states

There remain important biophysical questions concerning what motor-cargo configurations cause pauses in cargo trajectories [12] and whether reversal in direction is instantaneous or requires a paused tug-of-war intermediate state. As mentioned above, this data set is not sufficiently robust to fully answer these questions, but we do see evidence that paused states are more common when motor antagonism is present and paused states do occur between reverses in trajectories.

In agreement with our prior expectation that Kinesin-1 is a unidirectional and steadily-processive motor, we only observed 8 transitions among the 34 tracked paths (0.24 transitions per path, 0.09 transitions per second). Of these segments, none involved a reversal of direction. Similarly, for the DDB dataset there were 15 detected transitions among the 41 tracked paths (0.42 transitions per path, 0.16 transitions per second). Only one of these led to a reversed-direction state. However, individual inspection of the associated path indicates that this reversal might be an artifact of the first segment having a small number of data points, leading to a noisy estimation. See SI-DDB particle 16 for the path details. In contrast to the single motor data, state transitions were more commonly observed in for the antagonistic motor complex, Kin1-DDB. This was, in part, due the fact that that we could observe the paths for so much longer durations. Indeed, there were 128 detected transitions for the 92 tracked paths (1.39 transitions per path), but the transition rate was only slightly higher than that of Kinesin-1 (0.10 transitions per second).

**Figure 8.**
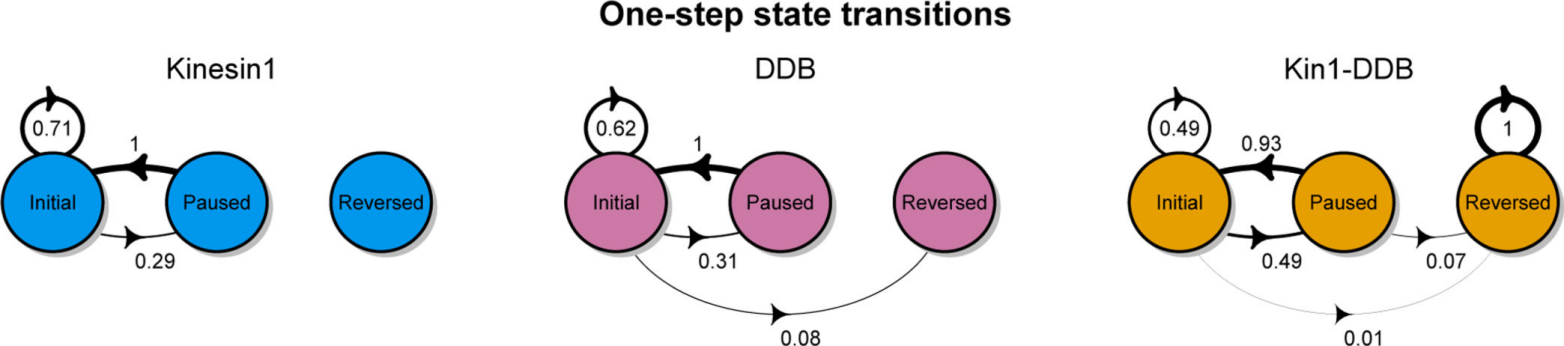
From left to right, the inferred transition diagram for the Kinesin-1, DDB, and Kin1-DDB data set. The nodes represent the three velocity states of motor-cargo complex’s velocity: movement in the initial direction (Initial), approximately no movement (Paused), and movement in the opposite direction (Reversed). The weights of each edge correspond to the observed transition probabilities. The transition probabilities of a state may not add to one due to rounding.

The methods we have described do allow for some insight into the transition properties of Kin1-DDB, but there are limitations in the experimental methods that prevent a full analysis. In particular, since we do not know the polarity of the microtubules in these experiments, we cannot know which motor is driving a particular state. Nevertheless, we can make some general observations about the role of the paused state. Most importantly, we see that direct reversals of direction are rare – usually there is an intermediate paused state, as is commonly predicted by tug-of-war models [10]. Using the method described in Section 3.4.3, we estimated the 1-step transition probabilities for each motor type, visually displayed in Figure 8. The node denotes a different state and the transition probability is given along the corresponding arrow where the thickness of the arrow corresponds to the magnitude of the transition probability. When a transition is marked from Initial to Initial, this means that there was a substantial change in velocity, where “substantial” is defined in Section 3.4.3. In the single motor experiments, the most common transition for both Kinesin-1 and DDB is Initial to Initial. While this is also the case for the for the Kinesin-1/DDB complex the transition from an Initial to a Paused state was slightly more probable than the single motor complexes (estimated probability 0.49). Due to the small sample size, the only statistically significant ordering we can make is the Reversed state is the least likely transition state from Initial. We caution the reader from taking too much from some of the transition probabilities. For example, there were only two transitions out of the reversed state for Kin1-DDB, both of which were to different Reverse-direction state.

## 6. Discussion

Identifying changes of means in time series of independent Normal random variables has a rich history. There are numerous techniques for this type on analysis, but few have been tailored for the specific application of intracellular transport by molecular motor data. The biological questions of interest here include i) what biophysical states the cargo is in, ii) how long it remains in each state, and iii) quantifying the characteristic parameters of each state. One important observation is that the distribution of velocities associated with active states are expected to be different for different molecular motor families. With this in mind, we have proposed a Bayesian change point method for detecting velocity changes in cargo trajectories that can faithfully be studied in a one-dimensional projection. We validated the method on simulated data in comparison to three other prominent techniques. Our method compared favorably in a set of simulated test cases meant to present realistic challenges presented by motor-transport. Moreover, we applied the method to three different experimental data sets: quantum dots being transported in vitro by a single Kinesin-1 motor, by a single DDB motor, and by a Kinesin-1/DDB pair. We saw substantial differences among the motor experiments that align with what might be expected in a molecular motor “folklore.” That is to say, Kinesin-1 steps processively with a stereotyped behavior while DDB shows more variation in its motility. When both motors are present, the velocity is typically smaller but the cargo stays attached for longer periods of time.

Our approach can be broken down into three basic steps. First, we identify the most likely number of switches; then conditioned on the number of switches we learn the segment velocities and change points; and lastly, we construct an estimated velocity distribution for the given set of trajectories. In each of these stages we sought to find a balance between statistical rigor and computational feasibility. For example, our approach to stage one is effective for one dimensional data but preliminary work indicates that our choice in likelihood might not be optimal for two dimensional data. Whether there exists an effective alternative to standard change point algorithms (bcp in particular) for 2D remains an open question. Moreover, it is not clear whether there is an optimal method for estimation of the velocity distribution for the purpose of characterizing motor-cargo interactions. Ultimately, the method we introduce here is sensitive enough to detect biophysically important differences in the behavior of different motor-cargo configurations.

With regard to biological implications, we see differences among the motor types, but more data and refined experimental techniques will be necessary to draw firm conclusions. For example, in this experimental framework it is not possible to know whether a motor run ended because it detached in the middle of a microtubule or at its end. Also, when Kinesin-1 and DDB are both present, we see bi-directional movement but we do not know the microtubule polarity, so we cannot yet attribute a particular state to a given motor or pair of motors. Our methods are readily adaptable to experimental data with higher resolution, and higher information content such as microtubule polarity, as well as to more complex complexes such as numerous kinesins and dyneins working against each other.

## Supplementary Material

supplemental material

## Figures and Tables

**Table 1.** Step 1 algorithm hyperparameters. In the following inference the hyperparameters are ${\underline{d}}_{r}=5$, ${\underline{u}}_{1}=0.25$, ${\underline{u}}_{2}=0.25$, ${\underline{\alpha }}_{\lambda }=(3/10)*50$, ${\underline{\beta }}_{\lambda }=50$, ${\underline{\alpha }}_{\text{prop}}=2.5$, ${\underline{\beta }}_{\text{prop}}=10$ and max velocity, $\underline{\text{v}}=2\mu \text{m}/\text{s}$.

Model Parameter	Prior	Prior Hyperparameters	Proposal kernel	Proposal Kernel Parameters
$v_{j}$	Uniform	$\underline{\text{v}}$: maximum allowed velocity	-	-
*η*	Gamma	${\underline{\alpha }}_{\eta }=0.15*{\hat{\eta }}_{\text{umvu}}$, ${\underline{\beta }}_{\eta }=0.1$	Normal random walk	$\mathrm{var}={\left({\hat{\eta }}_{\text{umvu}}/4\right)}^{2}$
***r***	Eq. 3.11	${\underline{d}}_{r}$: minimum segment duration	See [43]	${\underline{u}}_{1},{\underline{u}}_{2}$
*λ*	Gamma	${\underline{\alpha }}_{\lambda },{\underline{\beta }}_{\lambda }$	Independent Gamma	${\underline{a}}_{\text{prop}},{\underline{b}}_{\text{prop}}$

**Table 2.** Step 2 algorithm hyperparameters. In the following inference the hyperparameters are set to $\underline{d}=5$ and ${\underline{\text{v}}}_{\max}=2\mu \text{m}/s$ are considered. The parameters of the proposal distribution are set to ${\underline{\epsilon }}_{v}=1/2\mu \text{m}/s$ and ${\underline{\epsilon }}_{\tau }=\left\lceil {\left(1+k\right)}^{-2}*N\right\rceil *\text{Δ}$, where *k* is the assumed number of change points and *N* is the path length.

Model parameter	Prior	Prior Hyperparameters	Proposal kernel	Proposal Kernel Parameters
*v_j_*	Uniform	$\underline{\text{v}}$: maximum allowed velocity	Normal random walk	${\underline{\epsilon }}_{v}^{2}$
$\tau_{j}$	Eq. 3.25	$\underline{d}$: minimum state duration	Uniform random walk	${\underline{\epsilon }}_{\tau }$
η	Gamma	${\underline{\alpha }}_{v}=0.1*{\hat{\eta }}_{\text{MAP},1}$, ${\underline{\beta }}_{v}=0.1$	Gibbs sampling	-

**Table 3.** For each method and each case study consisting of 200 simulated paths, we give the accuracy of identifying the true number of change points along with 95% confidence interval. The accuracy fraction is estimated by the fraction of simulated paths with number of change points correctly estimated and confidence interval are obtained assuming a normal approximation to the binomial distribution.

% $\hat{k}=k_{\text{true}}$ for all simulated paths				

	Case 1	Case 2	Case 3	Case 4
NC-CI	0.90, [0.85, 0.94]	0.75, [0.68, 0.81]	0.61, [0.54, 0.67]	0.57, [0.50, 0.64]
BCP	0.80, [0.74, 0.86]	0.70, [0.63, 0.76]	0.52, [0.45, 0.59]	0.48, [0.40, 0.55]
LRT ($C_{0.95}=\hat{\mu }$)	0.80, [0.74, 0.86]	0.70, [0.63, 0.76]	0.54, [0.46, 0.61]	0.55, [0.48, 0.62]
RSS	0.77, [0.71, 0.83]	0.61, [0.54, 0.68]	0.53, [0.45, 0.60]	0.49, [0.42, 0.56]

**Table 4.** The accuracy of the estimate for the number of change points for the subset of paths with each segment duration lasting at least 5 observations and had subsequent velocity changes at least ±0.1*μ*m/*s*, $\left|v_{i}-v_{i+1}\right|\geq 0.1$ for all i=1,…k.

% $\hat{k}=k_{\text{true}}$ for the subset with “meaningful” changes				

	Case 1	Case 2	Case 3	Case 4
NC-CI	0.90	0.79	0.80	0.76
BCP	0.80	0.74	0.67	0.64
LRT ($C_{0.95}=\hat{\mu }$)	0.80	0.73	0.68	0.72
RSS	0.77	0.63	0.68	0.62

**Table 5.** The fraction of Kin1, DDB, and Kin1-DDB, paths whose NC-CI estimate number of change points, ${\hat{k}}_{\text{NC}-\text{CI}}$, was equal, less than, and greater than that when using the BCP method, ${\hat{k}}_{\text{BCP}}$.

	Kin1	DDB	Kin1-DDB
${\hat{k}}_{\text{NC-CI}}={\hat{k}}_{\text{BCP}}$	0.85	0.95	0.68
${\hat{k}}_{\text{NC-CI}}<{\hat{k}}_{\text{BCP}}$	0.00	0.02	0.21
${\hat{k}}_{\text{NC-CI}}>{\hat{k}}_{\text{BCP}}$	0.15	0.02	0.11

**Table 6.** The median run length and time tracked of the tracked cargo from the Kinesin-1, DDB, and Kin1-DDB data sets.

	Kinesin-1	DDB	Kin1-DDB
Median run length (*μ*m)	1.163	1.26	2.45
Median time tracked (s)	1.90	1.72	9.24

## 7. Appendix

### 7.1. Derivation of marginal likelihood in Section 3.2

For a fixed *η* and ***r*** the marginal likelihood of observing the data assuming uniform priors on the velocities is given by

(7.1)
$$\int_{\mathbb{R}}\ldots \int_{\mathbb{R}}L_{N,K_{r}}(\xi ;r,v,\eta )p\left(v_{1}\right)\ldots p\left(v_{K_{r}+1}\right)dv_{1}\ldots dv_{K_{r}+1}=\;\;\int_{\mathbb{R}}\cdots \int_{\mathbb{R}}{\left(\frac{\eta }{2\pi \text{Δ}}\right)}^{\frac{N}{2}}\exp\left(-\frac{\eta }{2\text{Δ}}\sum_{j=1}^{K_{r}+1}\sum_{n=M_{j-1}+1}^{M_{j}}{\left(\xi_{n}-v_{j}\text{Δ}\right)}^{2}\right)\;\;\times \frac{1}{2\underline{\text{v}}}1_{\left\{[-\underline{\text{v}},\underline{\text{v}}]\right\}}\left(v_{1}\right)\cdots \frac{1}{2\underline{\text{v}}}1_{\left\{[-\underline{\text{v}},\underline{\text{v}}]\right\}}\left(v_{K_{r}+1}\right)dv_{1}\ldots dv_{K_{r}+1}$$

Observing the integrand is integrable, employing Fubini’s theorem yields

(7.2)
$$={\left(\frac{\eta }{2\pi \text{Δ}}\right)}^{\frac{N}{2}}{\left(\frac{1}{2\underline{\text{v}}}\right)}^{K_{r}+1}\prod_{j=1}^{K_{r}+1}\int_{-\underline{\text{v}}}^{\underline{\text{v}}}\exp\left(-\frac{\eta }{2\text{Δ}}\sum_{n=M_{j-1}+1}^{M_{j}}{\left(\xi_{n}-\text{Δ}v_{j}\right)}^{2}\right)dv_{j}.$$

For the *j*th integral, the argument of the exponential can be expressed as

(7.3)
$$\sum_{n=M_{j-1}+1}^{M_{j}}{\left(\xi_{n}-\text{Δ}v_{j}\right)}^{2}=\sum_{n=M_{j-1}+1}^{M_{j}}{\left(\left(\xi_{n}-{\bar{\xi }}_{\left(M_{j-1},M_{j}\right]}\right)+\left({\bar{\xi }}_{\left(M_{j-1},M_{j}\right]}\text{Δ}-v_{j}\right)\right)}^{2}\;\;=\sum_{n=M_{j-1}+1}^{M_{j}}{\left(\xi_{n}-{\bar{\xi }}_{\left(M_{j-1},M_{j}\right]}\right)}^{2}+\left(M_{j}-M_{j-1}\right){\left({\bar{\xi }}_{\left(M_{j-1},M_{j}\right]}-\text{Δ}v_{j}\right)}^{2}.$$

Hence, *j*th integral can be written as

(7.4)
$$\int_{-\underline{\text{v}}}^{\underline{\text{v}}}\exp\left(-\frac{\eta }{2\text{Δ}}\sum_{n=M_{j-1}+1}^{M_{j}}{\left(\xi_{n}-\text{Δ}v_{j}\right)}^{2}\right)dv_{j}\;\;=\exp\left(-\frac{\eta }{2\text{Δ}}\sum_{n=M_{j-1}+1}^{M_{j}}{\left(\xi_{n}-{\bar{\xi }}_{\left(M_{j-1},M_{j}\right]}\right)}^{2}\right){\left(\frac{2\pi }{\left[\left(M_{j}-M_{j-1}\right)\text{Δ}\right]\eta }\right)}^{1/2}\;\;\times \int_{-\underline{v}}^{\underline{v}}{\left(\frac{\left[\left(M_{j}-M_{j-1}\right)\text{Δ}\right]\eta }{2\pi }\right)}^{1/2}\exp\left(-\frac{1}{2}\frac{{\left(v_{j}-{\bar{\xi }}_{\left(M_{j-1},M_{j}\right]}/\text{Δ}\right)}^{2}}{{\left(\left(M_{j}-M_{j-1}\right)\eta \text{Δ}\right)}^{-1}}\right)dv_{j}.$$

Viewing the integrand as the density function of a normal random variable with mean ${\bar{\xi }}_{\left(M_{j-1},M_{j}\right]}/\text{Δ}$ and variance ${\left(\left[\left(M_{j}-M_{j-1}\right)\text{Δ}\right]\eta \right)}^{-1}$, the *j* integral becomes

(7.5)
$$\exp\left(-\frac{\eta }{2\text{Δ}}\sum_{n=M_{j-1}+1}^{M_{j}}{\left(\xi_{n}-{\bar{\xi }}_{\left(M_{j-1},M_{j}\right]}\right)}^{2}\right){\left(\frac{2\pi }{\left[\left(M_{j}-M_{j-1}\right)\text{Δ}\right]\eta }\right)}^{1/2}\;\;\times \left(\text{Φ}\left(\frac{\underline{\text{v}}-{\bar{\xi }}_{\left(M_{j-1},M_{j}\right]}}{{\left(\eta \text{Δ}\left(M_{j}-M_{j-1}\right)\right)}^{-1/2}}\right)-\text{Φ}\left(\frac{-\underline{\text{v}}-{\bar{\xi }}_{\left(M_{j-1},M_{j}\right]}}{{\left(\eta \text{Δ}\left(M_{j}-M_{j-1}\right)\right)}^{-1/2}}\right)\right)$$

Φ(z) is the cumulative distribution function of a standard normal random variable. Thus for all *k* + 1 integrals we obtain Equation 3.17.
