# Supplementary material for: A change point analysis protocol for comparing intracellular transport by different molecular motor combinations
Source: Math Biosci Eng. Author manuscript; Available in PMC 2023 Jan 6. (PMC9817212; doi:10.3934/mbe.2021442)
Supplement: supplemental material [file NIHMS1850682-supplement-supplemental_material.zip › mbe-18-06-442 supplementary/Methods_SI.pdf]

1 Sampling the switch point process

The proposal function for the switch point process is a random function supported on $[0, 1]$ that proposes (1) a independent switch point process, (2) the creation or extinction of a change point or (3) a location shift of a single change point. Let $\boldsymbol{r}^{\text{prop}}$ denote the proposed switch point process and $\boldsymbol{r}^{\text{cur}}$ denote the current switch point process. The first type of proposal, denoted q_{new}, allows for large jumps in the switch point process aiding in escaping local maxima and allows for the number of change points to vary by proposing an independent switch point process:

$$q_{\text{new}}(\boldsymbol{r}^{\text{prop}}|\boldsymbol{r}^{\text{cur}}) = q_{\text{new}}(\boldsymbol{r}^{\text{prop}}) \sim p(\boldsymbol{r}^{\text{prop}}; \underline{\lambda}_{\text{r}}, \underline{d}_{\boldsymbol{r}}) \tag{1}$$

where $\underline{\lambda}_{\text{r}}$ is a hyper-prior chosen by the practitioner. For such proposal, the acceptance function has the form

$$\alpha_{\text{new}}(\boldsymbol{r}^{\text{prop}}, \boldsymbol{r}^{\text{cur}}) = \frac{p(\boldsymbol{r}^{\text{prop}}|\lambda^{\text{cur}}, \underline{d}_{\boldsymbol{r}})}{p(\boldsymbol{r}^{\text{cur}}|\lambda^{\text{cur}}, \underline{d}_{\boldsymbol{r}})} \frac{L(\boldsymbol{\xi}; \boldsymbol{r}^{\text{prop}}, \eta^{\text{cur}})}{L(\boldsymbol{\xi}; \boldsymbol{r}^{\text{cur}}, \eta^{\text{cur}})} \frac{p(\boldsymbol{r}^{\text{cur}}|\underline{\lambda}_{\text{r}}, \underline{d}_{\boldsymbol{r}})}{p(\boldsymbol{r}^{\text{prop}}|\underline{\lambda}_{\text{r}}, \underline{d}_{\boldsymbol{r}})}. \tag{2}$$

The second type, $q_{\text{bd}}(\boldsymbol{r}^{\text{prop}}|\boldsymbol{r}^{\text{cur}})$, provides another way for the number of change points to vary from iteration to iteration. A proposal of the second type is constructed by randomly sampling a component of the switch point that indicates a switch or a component that can be a switch

$$\begin{aligned} s \sim \text{Uniform}(S_{\boldsymbol{r}^{\text{cur}}}) := \{n : r_n^{\text{cur}} = 1\} \cup \\ \{m : \text{all the indices that are } d \text{ obs away from the switches}\} \end{aligned} \tag{3}$$

and then setting the proposal switch point process to

$$r_i^{\text{prop}} = \begin{cases} 1 - r_i^{\text{cur}} & \text{if } i = s \\ r_i^{\text{cur}} & \text{if } i \neq s. \end{cases} \tag{4}$$

For such type of proposal, the proposal function equals $1/|S_{\boldsymbol{r}^{\text{cur}}}|$, where $|S|$ denotes the cardinality of set S. This yields an acceptance function of the form

$$\alpha_{\text{bd}}(\boldsymbol{r}^{\text{prop}}, \boldsymbol{r}^{\text{cur}}) = \frac{p(\boldsymbol{r}^{\text{prop}}|\lambda^{\text{cur}}, \underline{d}_{\boldsymbol{r}})}{p(\boldsymbol{r}^{\text{cur}}|\lambda^{\text{cur}}, \underline{d}_{\boldsymbol{r}})} \frac{L(\boldsymbol{\xi}; \boldsymbol{r}^{\text{prop}}, \eta^{\text{cur}})}{L(\boldsymbol{\xi}; \boldsymbol{r}^{\text{cur}}, \eta^{\text{cur}})} \frac{|S_{\boldsymbol{r}^{\text{cur}}}|}{|S_{\boldsymbol{r}^{\text{prop}}}|}. \tag{5}$$

The last type, $q_{\text{shift}}(\boldsymbol{r}^{\text{prop}}|\boldsymbol{r}^{\text{cur}})$, allows for exploration of the best combination of change points for a fixed number of change points. The proposed switch point process is obtained by randomly sampling two components of the current switch

point process: one from the set of all current change points, τ_j for $j = 1, \ldots, k_r = \sum_{i=1}^{N-1} r_i$, and the other from the complement such that $\boldsymbol{r}^{\text{prop}} \in \mathcal{T}_{N,\underline{d}_r}$:

$$\begin{aligned} s &\sim \text{Uniform}(\{\tau_1, \tau_2, \ldots, \tau_{k_r}\}) \\ s' &\sim \text{Uniform}(\{m : \text{at least } \boldsymbol{d_r} \text{ entries away from } \tau_j \;\; \forall j = 1, \ldots k_r\}). \end{aligned} \tag{6}$$

The proposal of this type is defined as

$$r_i^{\text{prop}} = \begin{cases} 1 - r_i^{\text{cur}} & \text{if } i = s, s' \\ r_i^{\text{cur}} & \text{if } i \neq s, s'. \end{cases} \tag{7}$$

Although both switch point process have the same number of change points, the prior ratio does not cancel as the prior depends on the placement of switch points (unless $\underline{d}_r = 1$). The resulting acceptance function is of the form

$$\alpha_{\text{shift}}(\boldsymbol{r}^{\text{prop}}, \boldsymbol{r}^{\text{cur}}) = \frac{p(\boldsymbol{r}^{\text{prop}}|\lambda^{\text{cur}}, \underline{d}_r)}{p(\boldsymbol{r}^{\text{cur}}|\lambda^{\text{cur}}, \underline{d}_r)} \frac{L(\boldsymbol{\xi}; \boldsymbol{r}^{\text{prop}}, \eta^{\text{cur}})}{L(\boldsymbol{\xi}; \boldsymbol{r}^{\text{cur}}, \eta^{\text{cur}})} \frac{q_{\text{shift}}(\boldsymbol{r}^{\text{cur}}|\boldsymbol{r}^{\text{prop}})}{q_{\text{shift}}(\boldsymbol{r}^{\text{prop}}|\boldsymbol{r}^{\text{cur}})} \tag{8}$$

The ratio of the proposal should be

$$\frac{\left|\{n : r_n^{\text{cur}} = 0, n \; d_r \text{away from any changepoint}\}\right|}{\left|\{n : r_n^{\text{prop}} = 0, n \; d_r \text{away from any changepoint}\}\right|}. \tag{9}$$

For hyper-parameter $\underline{\boldsymbol{u}} = (\underline{u}_1, \underline{u}_2, 1 - \underline{u}_1 - \underline{u}_2)$ such that $0 < \underline{u_i} < 1$ for $i = 1, 2$ and $\underline{u}_1 + \underline{u}_2 = 1 - \underline{u}_1 - \underline{u}$, the proposal function for the switch point process is given by

$$q_r(\boldsymbol{r}^{\text{prop}}|\boldsymbol{r}^{\text{cur}}; u) = \begin{cases} q_{\text{new}}(\boldsymbol{r}^{\text{prop}}) & \text{if } 0 \leq u \leq \underline{u}_1 \\ q_{\text{bd}}(\boldsymbol{r}^{\text{prop}}|\boldsymbol{r}^{\text{cur}}) & \text{if } \underline{u}_1 < u \leq \underline{u}_2 \\ q_{\text{shift}}(\boldsymbol{r}^{\text{prop}}|\boldsymbol{r}^{\text{cur}}) & \text{if } \underline{u}_2 < u \leq 1. \end{cases} \tag{10}$$

When the current switch point process is in one of the extreme configuration, no switches or all switches, the last possible transition $q_{\text{shift}}(\boldsymbol{r}^{\text{prop}}|\boldsymbol{r}^{\text{cur}})$ is impossible. In this situation, the hyper parameters $\boldsymbol{u}$ is rescaled to

$$\underline{\boldsymbol{u}'} = \left(\frac{u_1}{\underline{u}_1 + \underline{u}_2}, \frac{u_2}{\underline{u}_1 + \underline{u}_2} \right)$$

2 Pseudo-code for sampling algorithms

Algorithm 1 Gibbs/MH Algorithm: Unknown number of change points

Initialize:
$\boldsymbol{r}^0, \quad \lambda^{(0)}, \quad \eta^{(0)}$

for iterations $i = 1, 2, \ldots, i_{\max}$ **do**
  $\lambda^{(\text{cand})} \sim q_\lambda(\bullet|\lambda^{(\text{i-1})})$

  if $\text{unif}(0,1) \leq \alpha(\lambda^{\text{cand}}, \lambda^{(i-1)})$ **then**
    $\lambda^{(i)} = \lambda^{\text{cand}}$
  else
    $\lambda^{(i)} = \lambda^{(i-1)}$

  $\eta^{(\text{cand})} \sim q_\eta(\bullet|\eta^{(\text{i})})$

  if $\text{unif}(0,1) \leq \alpha(\eta^{\text{cand}}, \eta^{(i-1)})$ **then**
    $\eta^{(i)} = \eta^{\text{cand}}$
  else
    $\eta^{(i)} = \eta^{(i-1)}$

  $u_{\boldsymbol{r}} \sim \text{unif}(0,1)$; $\boldsymbol{r}^{\text{cand}} = q_{\boldsymbol{r}}(\bullet|\boldsymbol{r}^{(i-1)}, u_{\boldsymbol{r}})$

  if $\text{unif}(0,1) \leq \alpha(\boldsymbol{r}^{\text{cand}}, \boldsymbol{r}^{(i-1)})$ **then**
    $\boldsymbol{r}^{(i)} = \boldsymbol{r}^{\text{cand}}$
  else
    $\boldsymbol{r}^{(i-1)} = \boldsymbol{r}^{(i)}$

  i =i+1
end for
Output: Set of $i_{\max}$ posterior samples of $\lambda, \eta, \boldsymbol{r}$.

Algorithm 2 Gibbs/MH Algorithm: Known number of change points

Initialize:
$\boldsymbol{\nu}^0, \quad \eta^{(0)}, \quad \boldsymbol{\tau}^{(0)}$
for iterations $i = 1, 2, \ldots, i_{\max}$ **do**
  $\eta^{(i)} \sim \text{Gamma}(\alpha(\boldsymbol{\xi}, \boldsymbol{\nu}^{(i-1)}, \boldsymbol{\tau}^{(i-1)}), \beta(\boldsymbol{\xi}, \boldsymbol{\nu}^{(i-1)}, \boldsymbol{\tau}^{(i-1)}))$
  for $k = 1, \ldots, K+1$ **do**
    $\nu_j^{\text{cand}} \sim q(\bullet | \nu_j^{(i-1)})$
    if $\text{unif}(0,1) \leq \alpha(\nu_j^{\text{cand}}, \nu_j^{(i-1)})$ **then**
      $\nu_j^{(i)} = \nu_j^{\text{cand}}$
    else
      $\nu_j^{(i)} = \nu_j^{(i-1)}$
  end for
  for k = 1, . . . , K **do**
    $\tau_j^{\text{cand}} \sim q(\bullet | \tau_j^{(\text{i})})$, $M_j^{\text{cand}} = \lfloor \tau_j^{\text{cand}} \Delta \rfloor$
    if $\text{unif}(0,1) \leq \alpha(M_j^{\text{cand}}, M_j^{(i-1)})$ **then**
      $\tau_j^{(i)} = \tau_j^{\text{cand}}$
    else
      $\tau_j^{(i)} = \tau_j^{(i-1)}$
  end for
  i =i+1
end for
Output: Set of $i_{\max}$ posterior samples of $\boldsymbol{\nu}, \eta, \boldsymbol{\tau}$.

3 Details about quantifying transitions between biological states

In order to quantify switches among biophysical states, we defined three categories based on threshold values for the segment speed and the segment distance as defined in the main text in Section 3.4.3. We only observed long, slow segments for paths with the two-motor complex, Kin-DDB, not for the paths with a single motor complex, Kin1 or DDB. Below in Figure 1, we display the segment speed versus segment distance for all inferred segments, where each frame corresponds to a different motor protein, DDB, Kin1, and Kin1-DDB (from left to right). The color of each point denotes the classified state using the cut stated in Section 3.4.3 and the red dash lines denote the cutoff values, either max speed = 0.1 $\mu m/s$ or minimum distance = 0.4 μm. Note the absence of segments in the upper left quadrant for Kinesin-1 and DDB, indicating that for these single motor experiments, there were not any paths with slow speed ($< 0.1 \mu m s$) that cover a large distance ($\geq 0.4 \mu m$). This is not true for the two-motor complex, Kin1-DDB, see the rightmost frame.

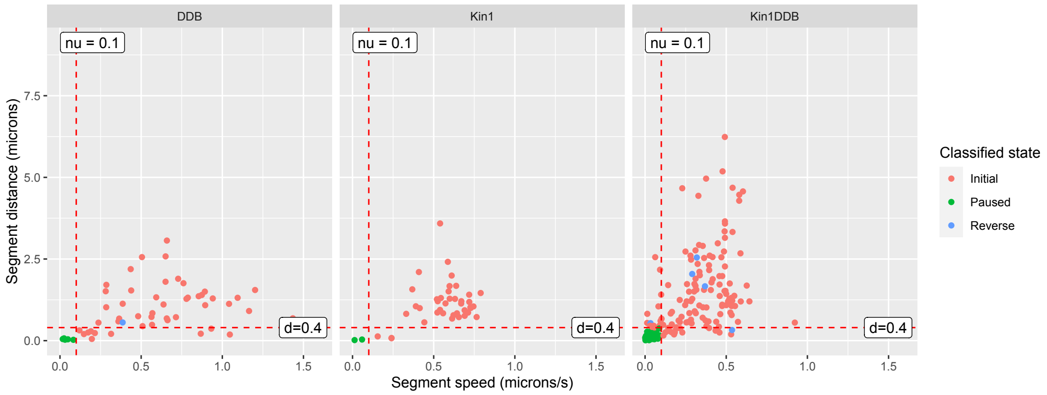

Figure 1: The inferred segment speed versus the segment distance for all paths with motor-proteins DDB(left), Kin1(center), and Kin1-DDB (right). The color of the point corresponds to the classified biological state as defined in main text in Section 3.4.3 and the red dashed lines correspond to the threshold values used to define the biological states.

We further investigated the effects of our choice in threshold values had on our analysis of whether reversal in direction is instantaneous or requires a paused tug-of-war intermediate state. To do this, we modified our threshold values for segment speed to $0.2 \mu m/s$ and for segment distance to $0.5 \mu m$. While this did alter the inferred transition rates, we found the same overall result, that reversal of direction often involves a paused state. In Figure 2, we provide the inferred transition diagram for the threshold values given in the main text (top) and for

the alternative threshold values given above (bottom).

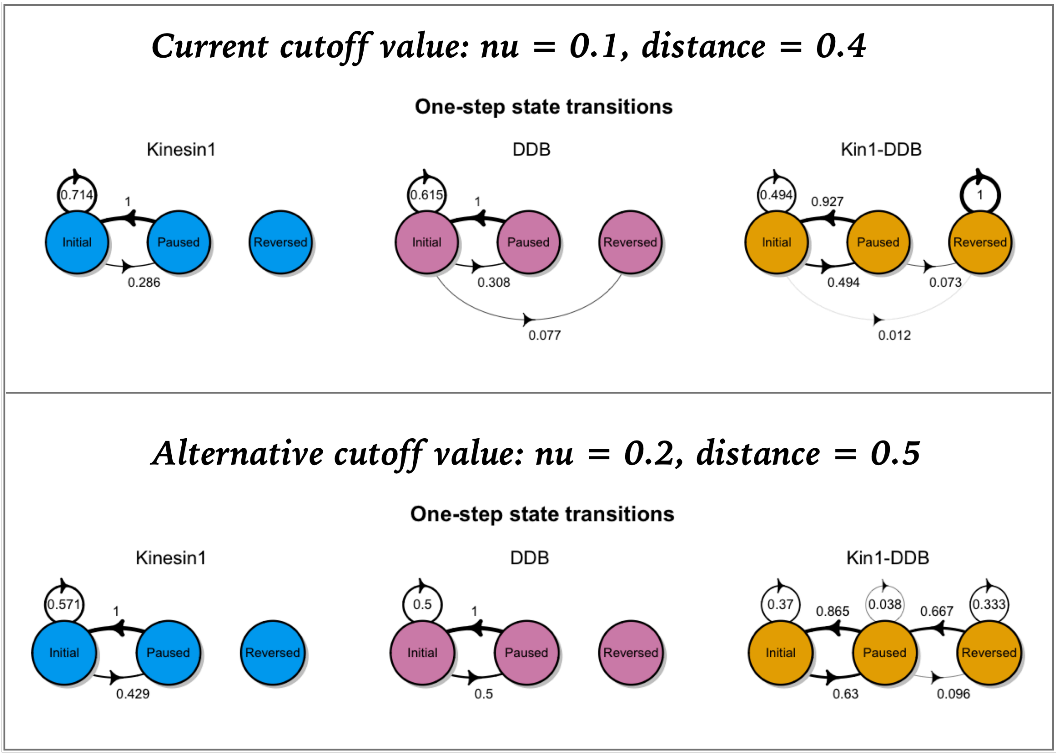

Figure 2: From left to right, the inferred transition diagram for the Kinesin-1, DDB, and Kin1-DDB data set for the threshold values given in the main text (top) and the an alternate set proposed above (bottom). The nodes represent the three velocity states of motor-cargo complex's velocity: movement in the initial direction (Initial), approximately no movement (Paused), and movement in the opposite direction (Reversed). The weights of each edge correspond to the observed transition probabilities.

4 Details for numerical experiments

Algorithm 3 Simulating a cargo-motor complex that changes stepping rate k times

Initialize:
$j = 1$; $t_{\text{curr}} = 0$; $\lambda_{\text{curr}} = \lambda_j$; $Z_0 = 0$; $X_0 = 0$; $N = T_{\text{final}}/\Delta$

for $t_{\text{curr}} < T_{\text{final}}$ **do**
  $s \sim \exp(\lambda_{\text{curr}})$
  $t_{\text{curr}} = t_{\text{curr}} + s$; $\boldsymbol{s'} = (\boldsymbol{s'}, t_{\text{curr}})$

  if $t_{\text{curr}} \geq \tau_{\text{curr}}$ **then**
    $\tau_j = t_{\text{curr}}$
    $\lambda_{\text{curr}} = \lambda_{j+1}$
end for

$\{s_n\}_{n=1} := \{s'_n\}_{n=1} \cup \{\tau_n\}_{n=1}^{k} \cup \{n\Delta_{\text{sim}}\}_{n=1}^{N}$
$j = 1$

for $n = 1, \dots |\boldsymbol{s}|$ **do**

  if s_n equals τ_j **then**
    $Z_n = Z_{n-1} + \delta_{\text{step}}$
    $j = j + 1$
  else
    $Z_n = Z_{n-1}$

  $\rho_n = e^{-\frac{\kappa}{\gamma}(s_n - s_{n-1})}$
  $X_n = \rho_n X_{n-1} + (1 - \rho_n) Z_{n-1} + \sqrt{\frac{D}{\kappa/\gamma}(1 - \rho_n^2)} * \text{Normal(0,1)}$
end for
Output: $\boldsymbol{Z} = (Z_1, \dots, Z_{|\boldsymbol{s}|}), \boldsymbol{X} = (X_1, \dots, X_{|\boldsymbol{s}|})$

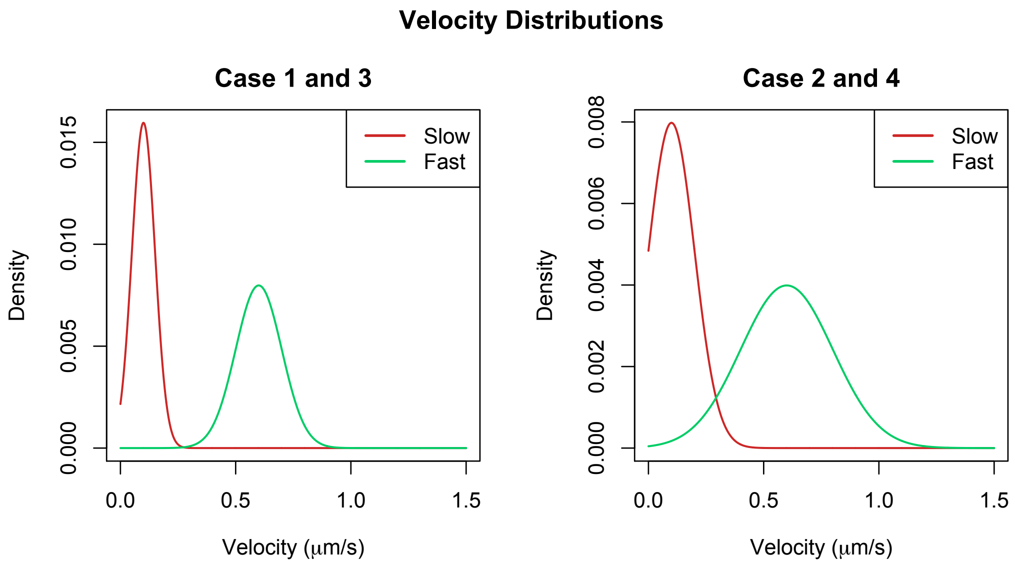

Figure 3: **(a),(b):** Assumed velocity distributions, folded normal, used in the simulations presented in the main manuscript for Cases 1 & 3, and Case 2 & 4, respectively. The fast and slow velocity distribution are denoted in green and red, respectively. There is more overlap between distributions in right frame.

5 Compact vs non-compact prior on segment velocity

For comparison of the inference on the number of change points, when assuming a non-compact prior versus a compact prior on the segment velocities, we provide the inference results for path 34 in the DDB data set in Figure 4 assuming a normal prior and in Figure 5 for a compact uniform prior. The bottom right of both figures displays the estimated posterior distribution for the number of change points. When a normal prior is assumed, Figure 4, the MAP estimate is 47, whereas assuming a compact uniform prior yields a MAP estimate of 2, Figure 5 bottom right.

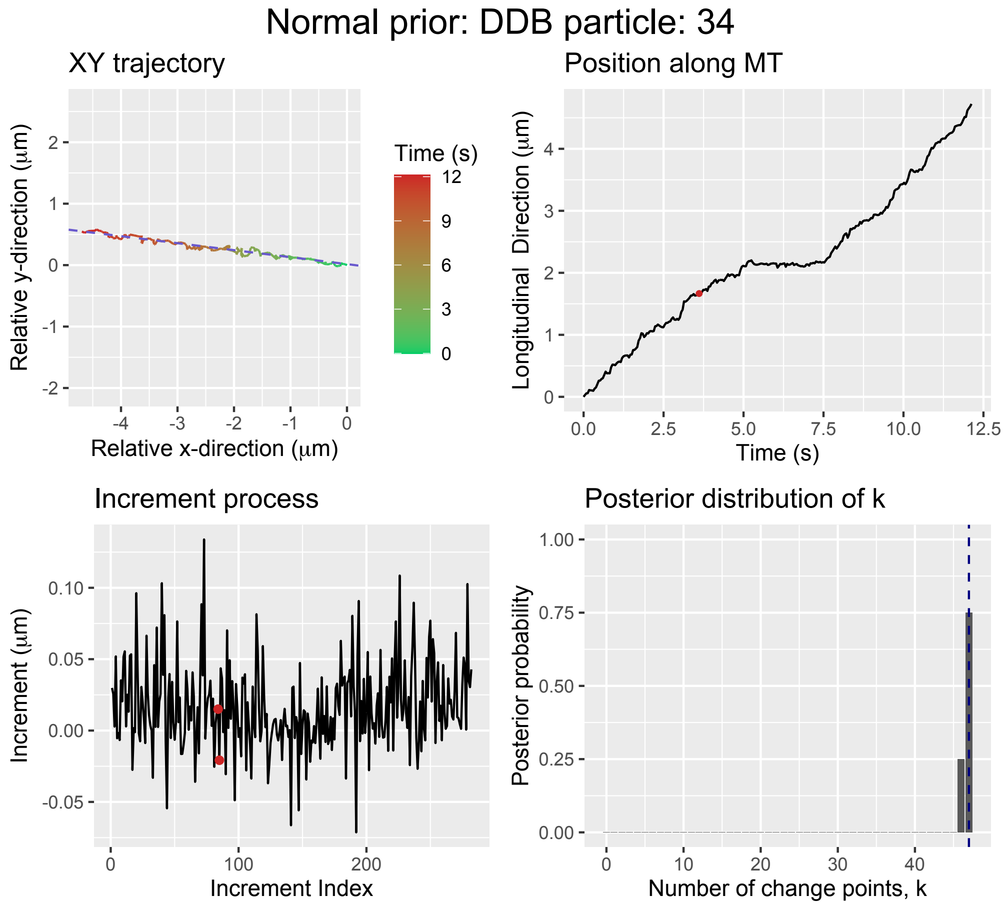

Figure 4: Inference performed on DDB path 34 when a normal prior is assumed on the segment velocities. The path of the cargo in the relative xy plane (top left) where the purple dashed line is the estimated MT and the color denoted the time. The longitudinal direction of the cargo versus time (top right) and the increment process of the longitudinal position (bottom left), where the red points denote missing observations. The inferred posterior distribution for the number of change points when normal priors are assumed on the segment velocities (bottom right) where the dashed blue vertical lines denotes the MAP, 47 change points.

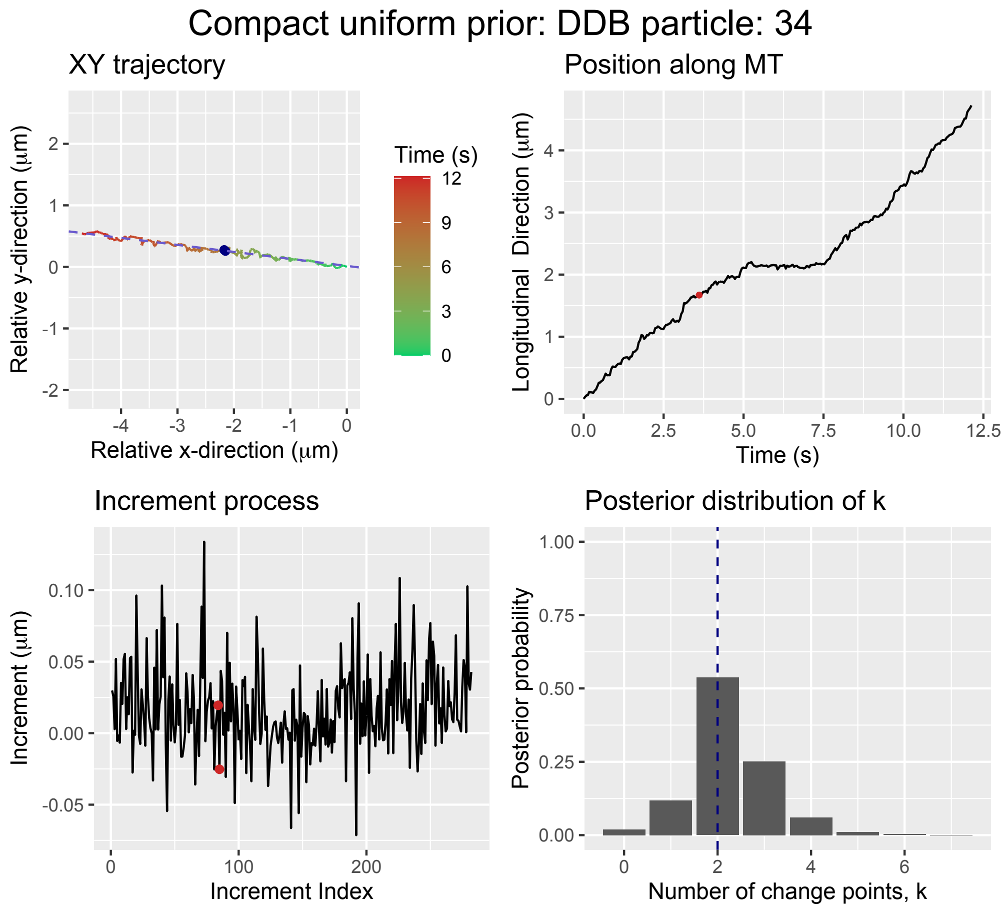

Figure 5: Inference performed on DDB path 34 when a compact uniform prior is assumed on the segment velocities. The path of the cargo in the relative xy plane (top left) where the purple dashed line is the estimated MT, the color denoted the time, and blue points denote the MAP estimate for the two change points. The longitudinal direction of the cargo versus time (top right) and the increment process of the longitudinal position (bottom left). Red points denote missing observations and dashed blue vertical lines denote the MAP estimates for the change points. The inferred posterior distribution for the number of change points when normal priors are assumed on the segment velocities (bottom right) where the dashed blue vertical lines denotes the MAP, 2 change points.
