# Supplementary material for: A change point analysis protocol for comparing intracellular transport by different molecular motor combinations
Source: Math Biosci Eng. Author manuscript; Available in PMC 2023 Jan 6. (PMC9817212; doi:10.3934/mbe.2021442)
Supplement: supplemental material [file NIHMS1850682-supplement-supplemental_material.zip › mbe-18-06-442 supplementary/SI_DDB.pdf]

DDB particle: 1

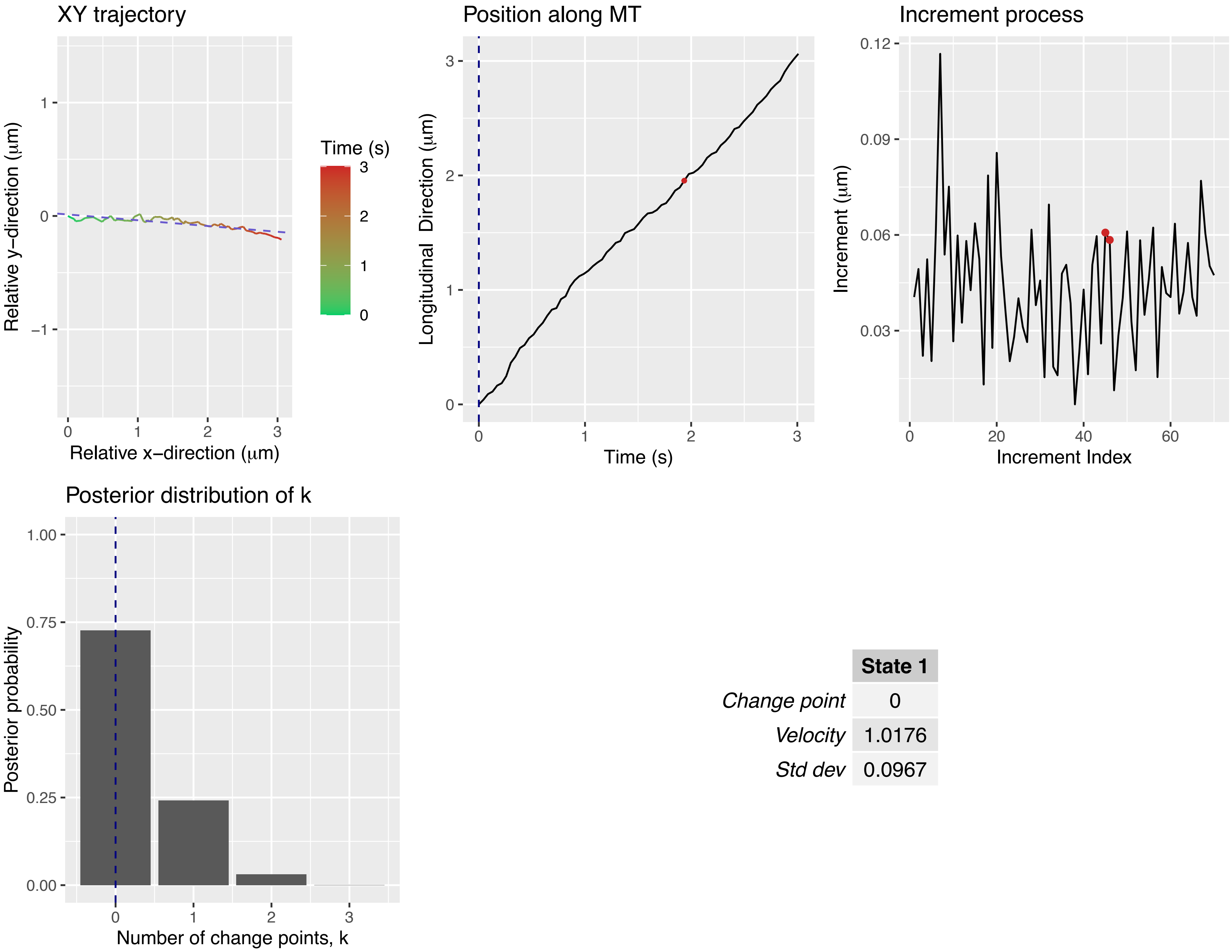

	State 1
Change point	0
Velocity	1.0176
Std dev	0.0967

DDB particle: 2

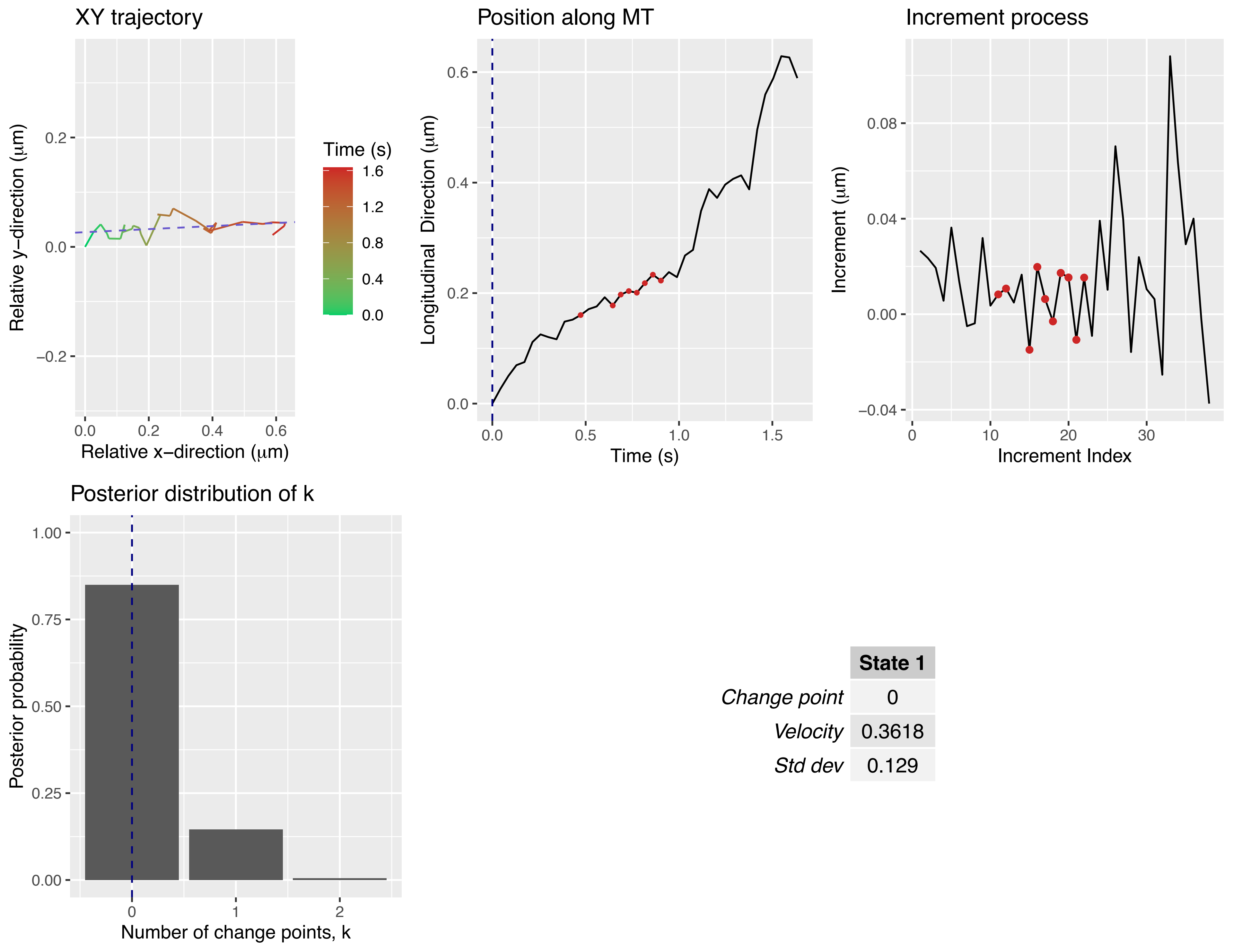

	State 1
Change point	0
Velocity	0.3618
Std dev	0.129

DDB particle: 3

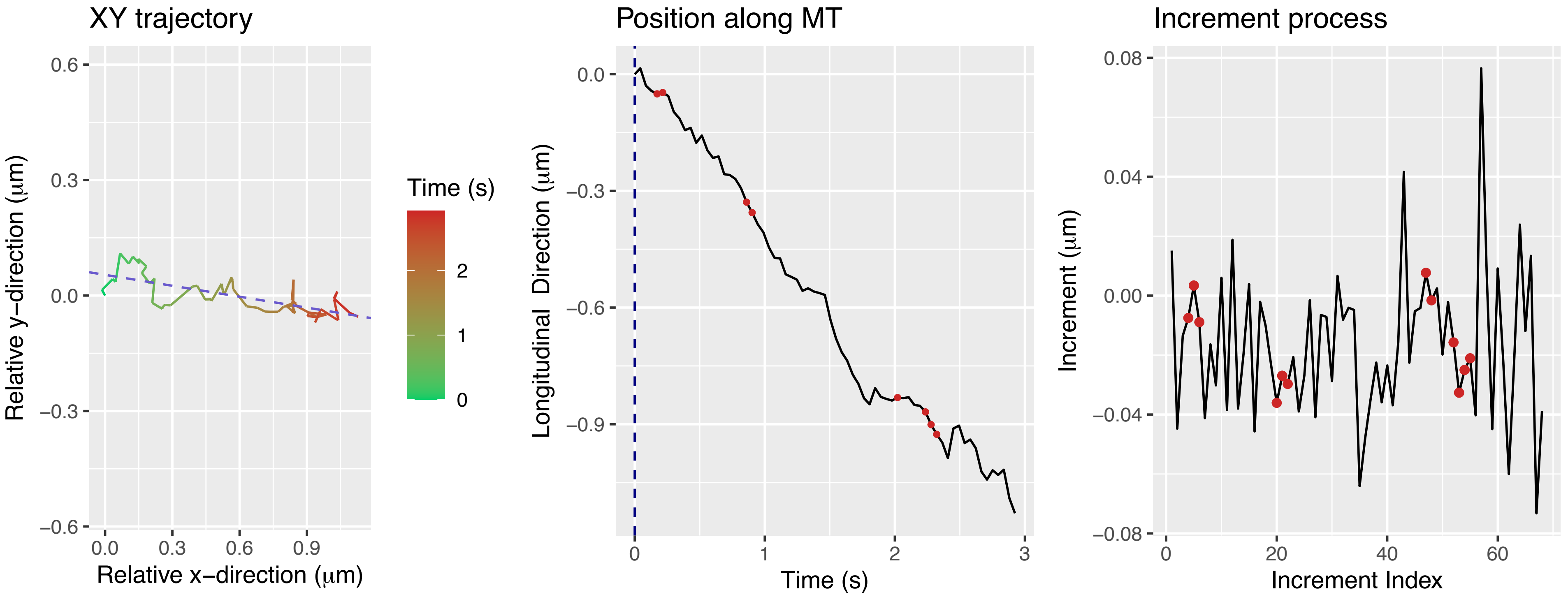

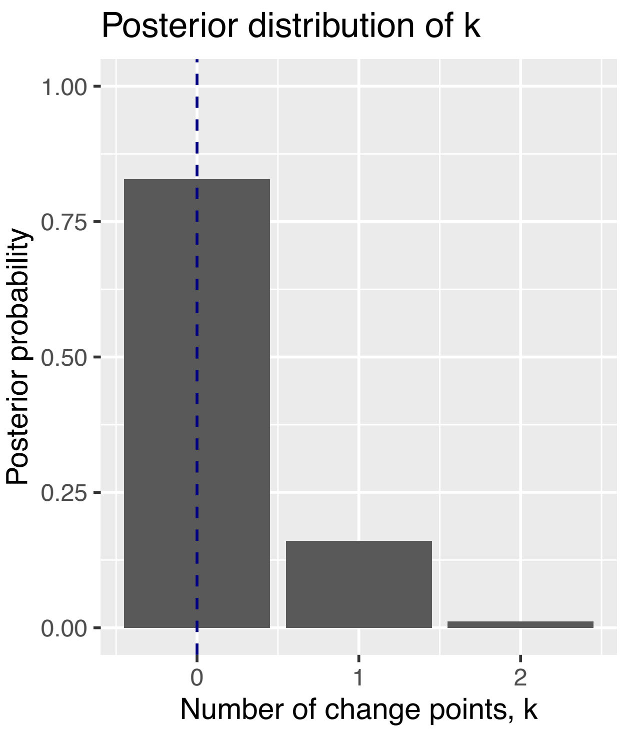

	State 1
Change point	0
Velocity	−0.3831
Std dev	0.118

DDB particle: 4

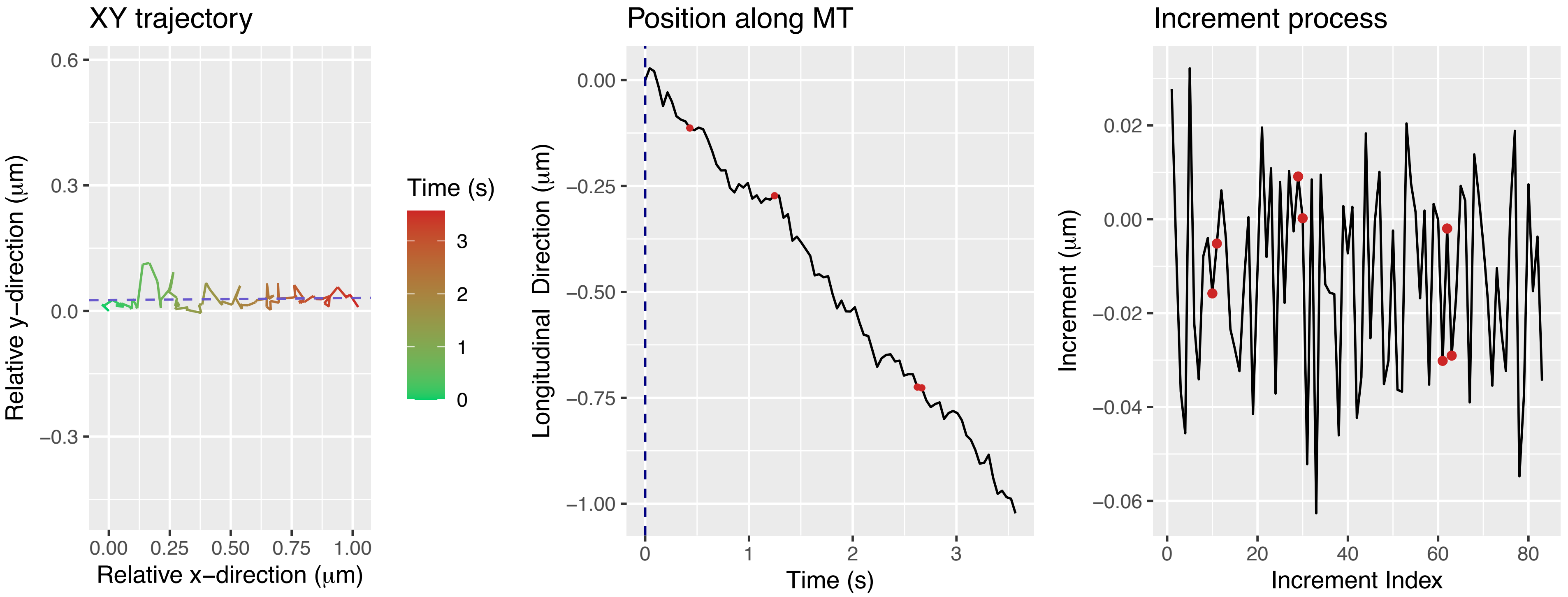

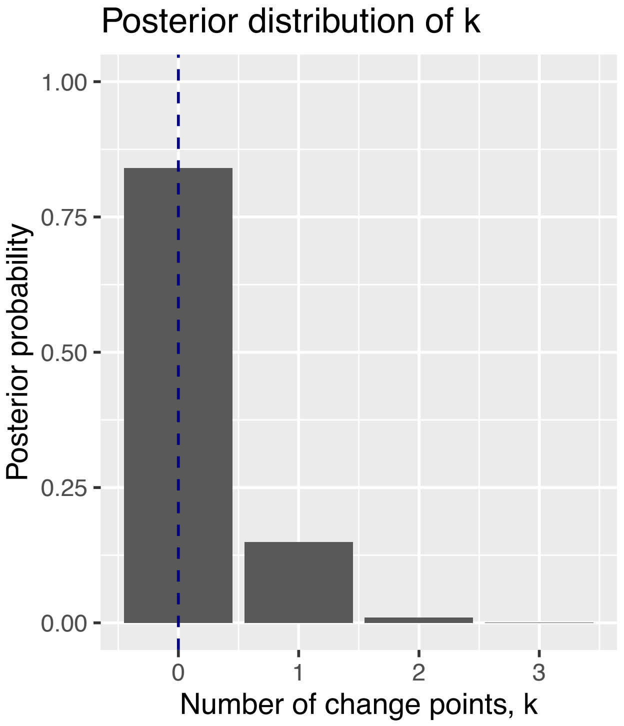

	State 1
Change point	0
Velocity	–0.2866
Std dev	0.1

DDB particle: 5

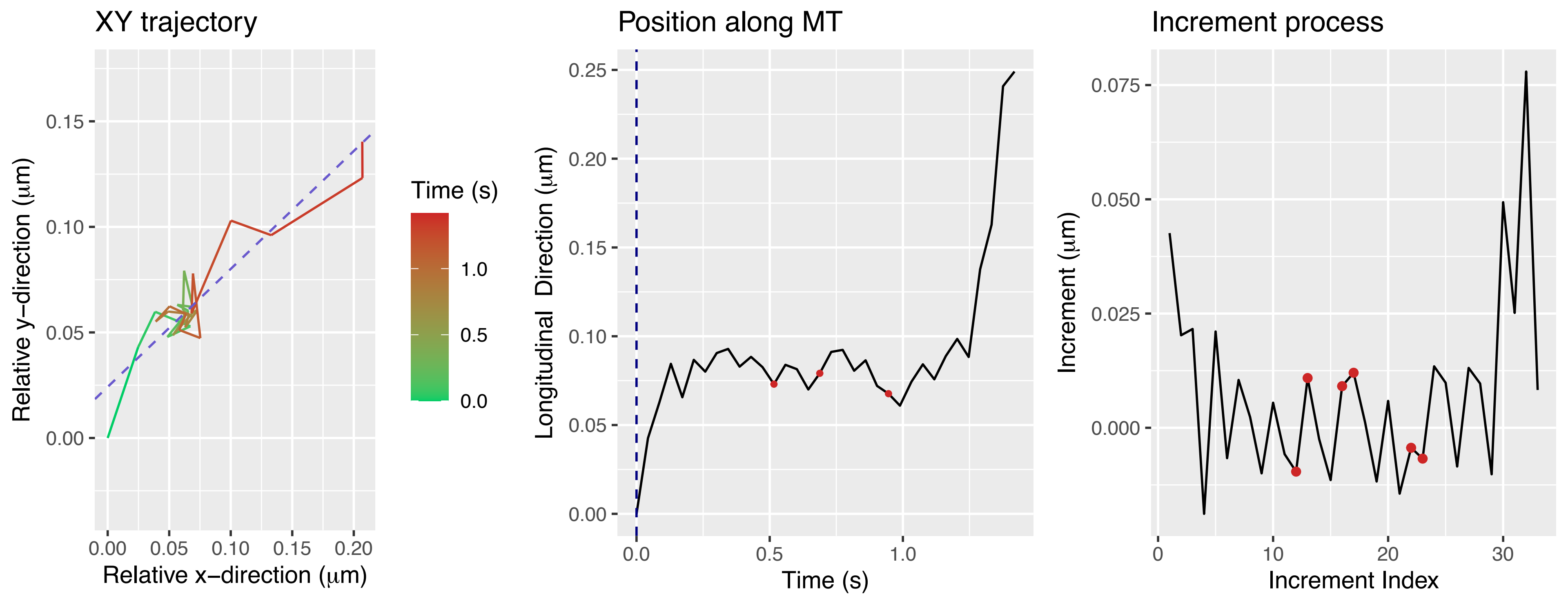

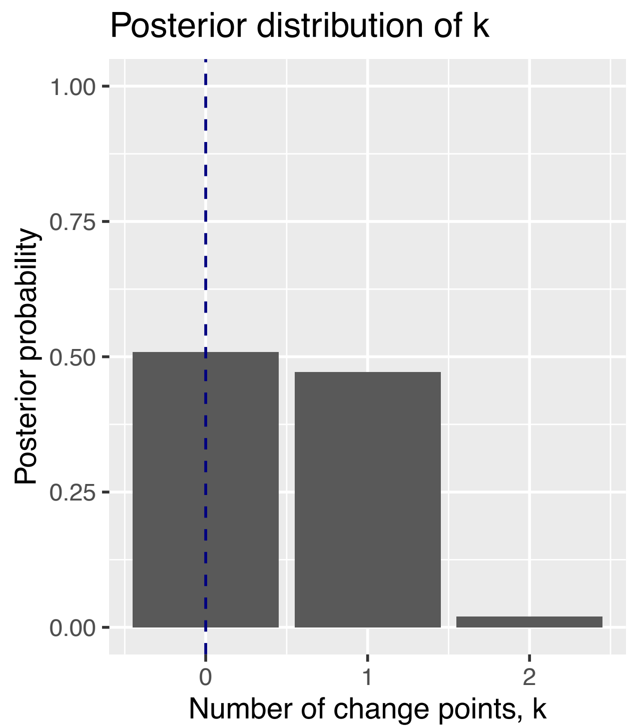

	**State 1**
Change point	0
Velocity	0.1771
Std dev	0.0937

DDB particle: 7

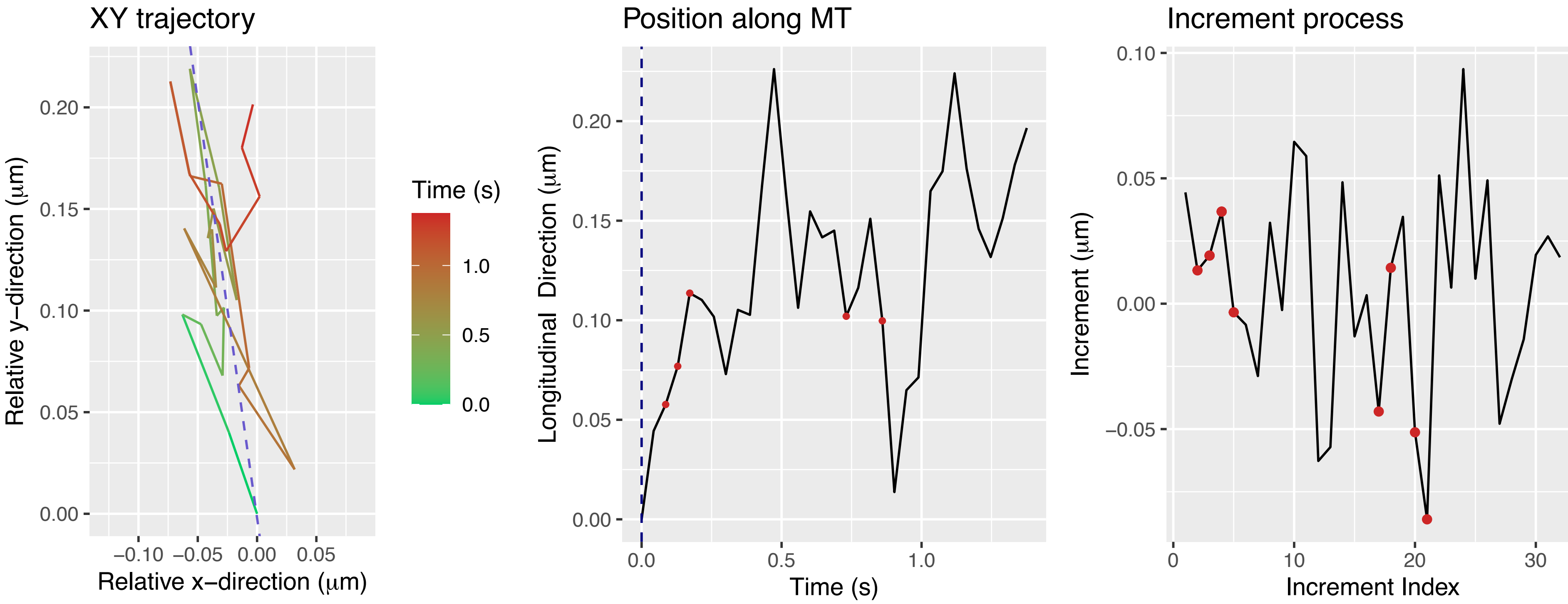

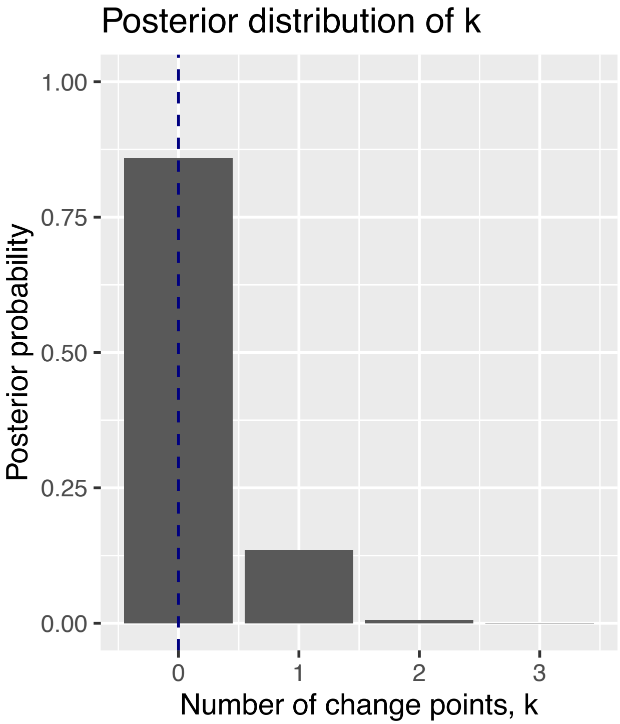

	State 1
Change point	0
Velocity	0.1404
Std dev	0.2031

DDB particle: 8

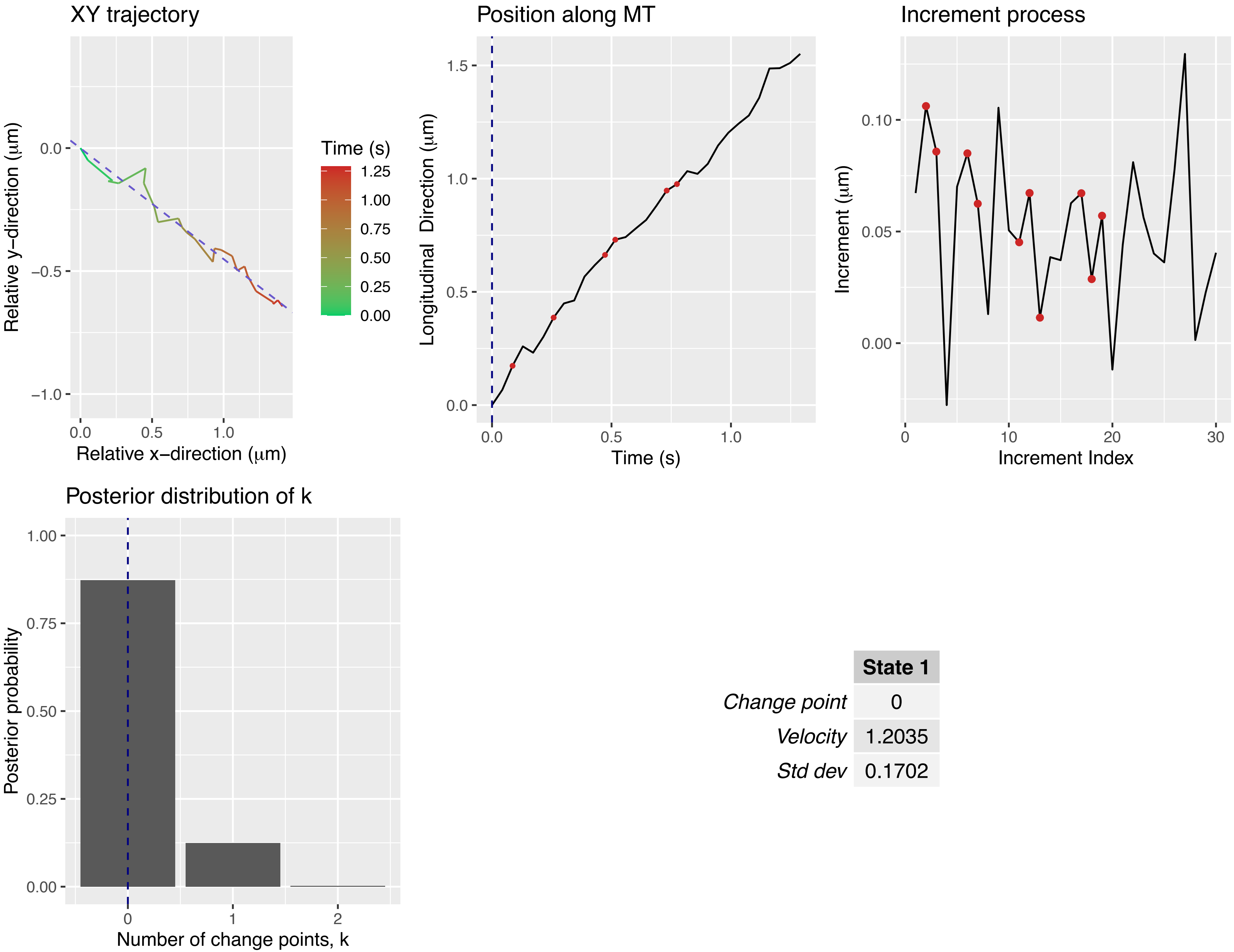

	State 1
Change point	0
Velocity	1.2035
Std dev	0.1702

DDB particle: 9

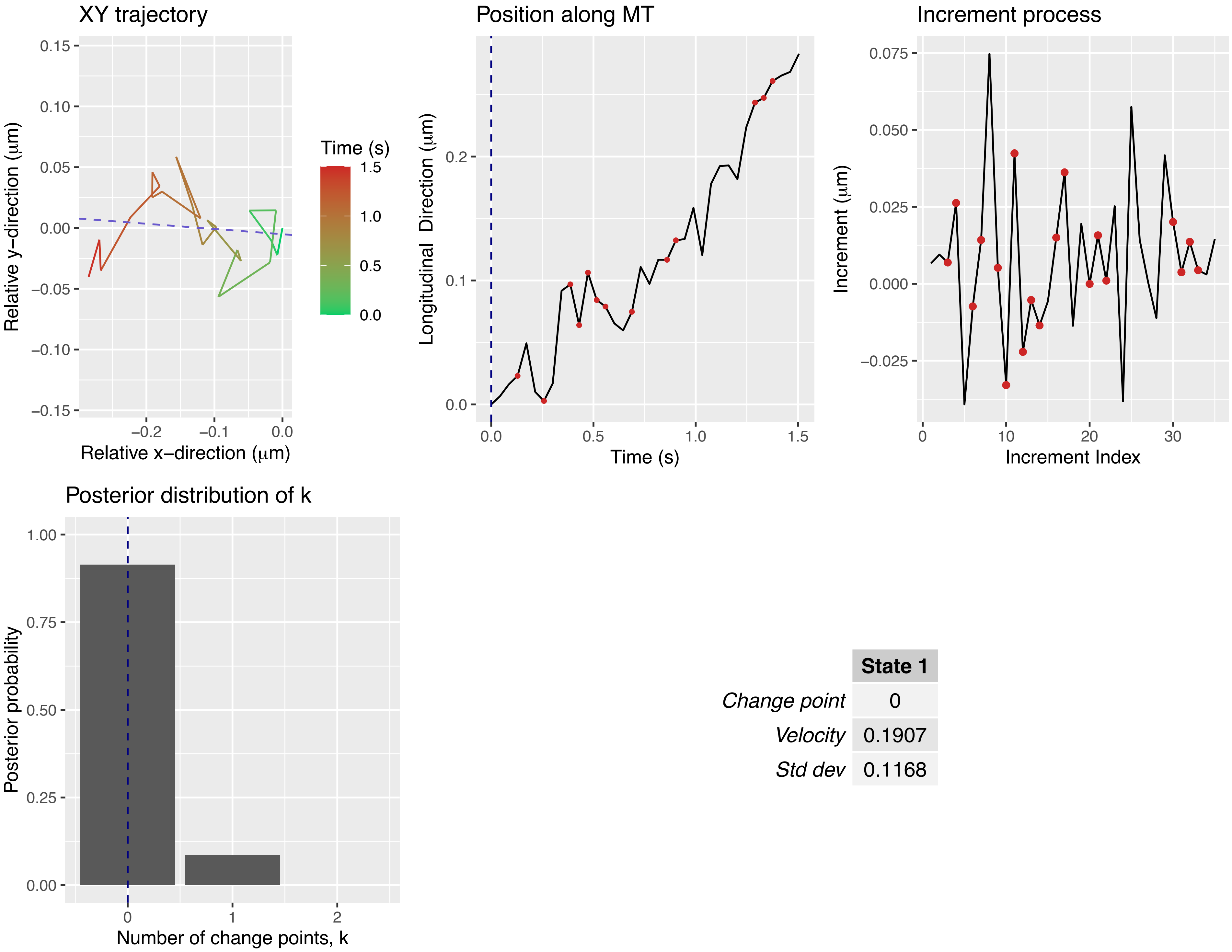

	State 1
Change point	0
Velocity	0.1907
Std dev	0.1168

DDB particle: 10

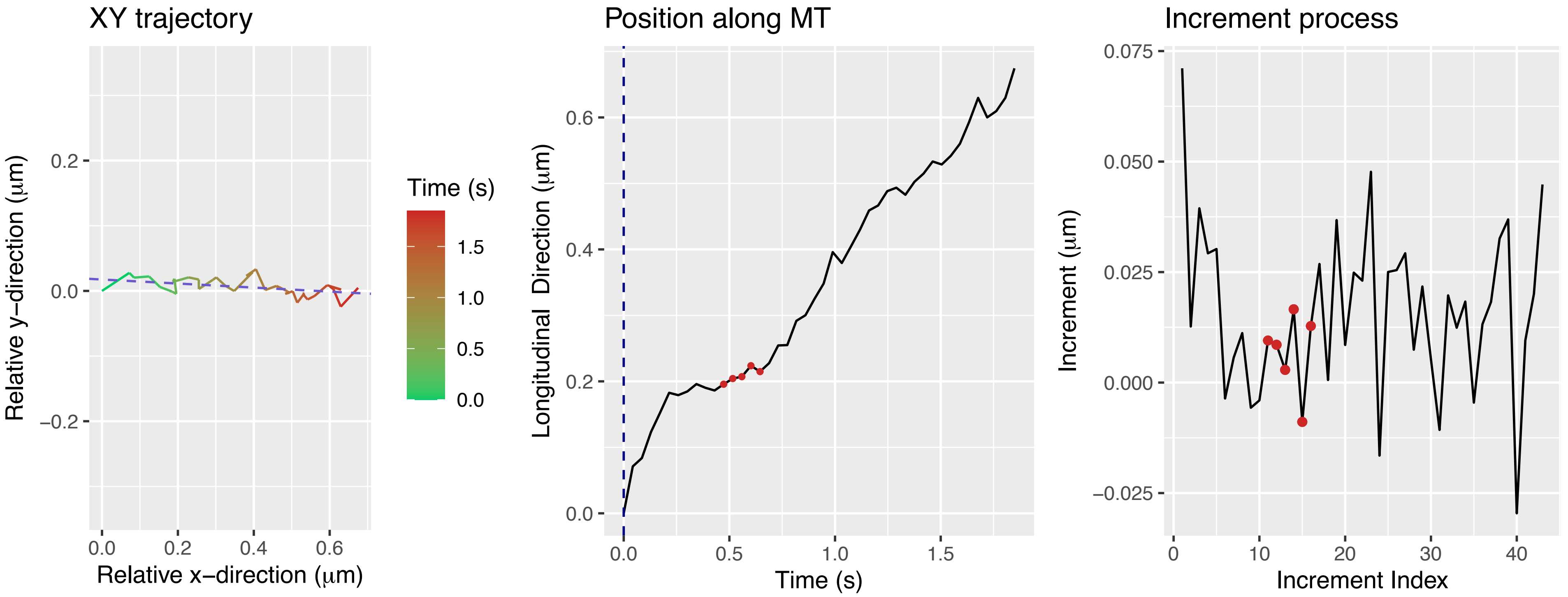

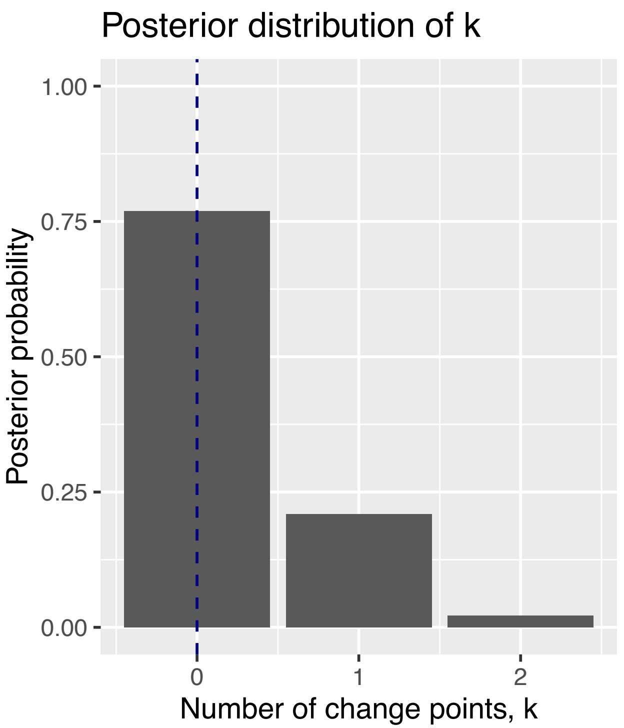

	State 1
Change point	0
Velocity	0.3659
Std dev	0.0887

DDB particle: 11

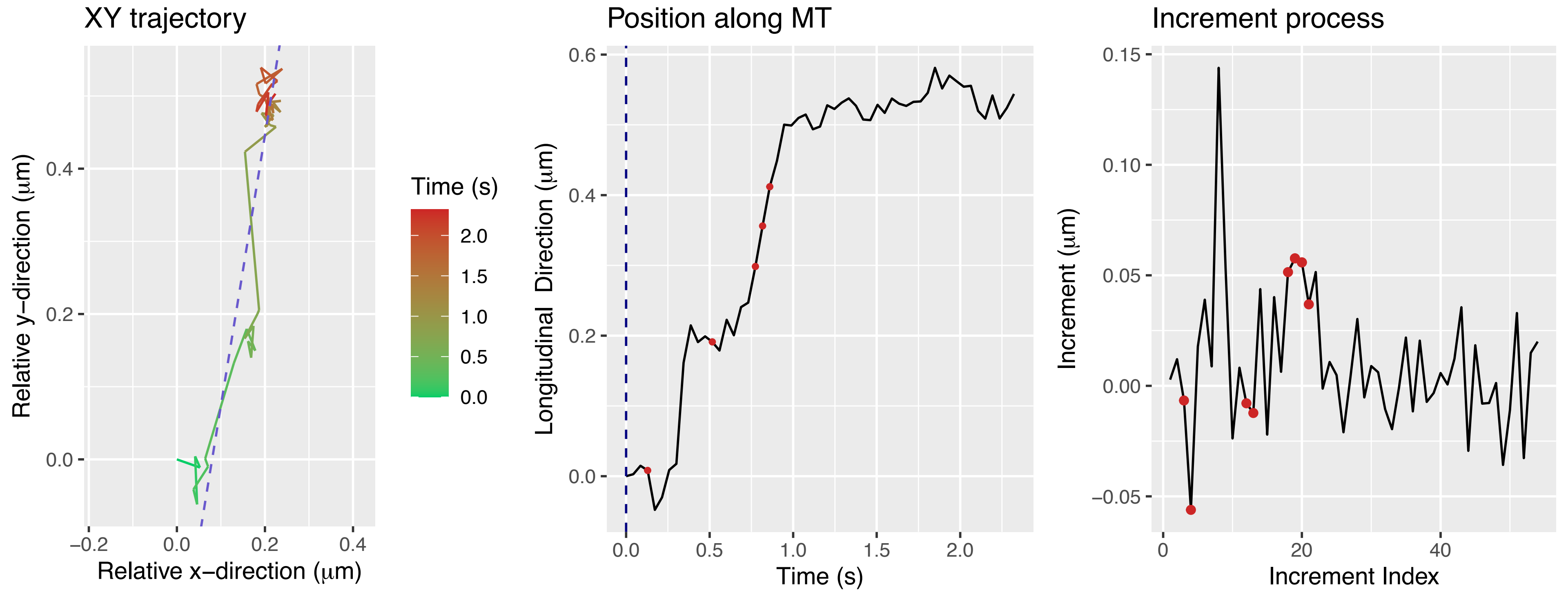

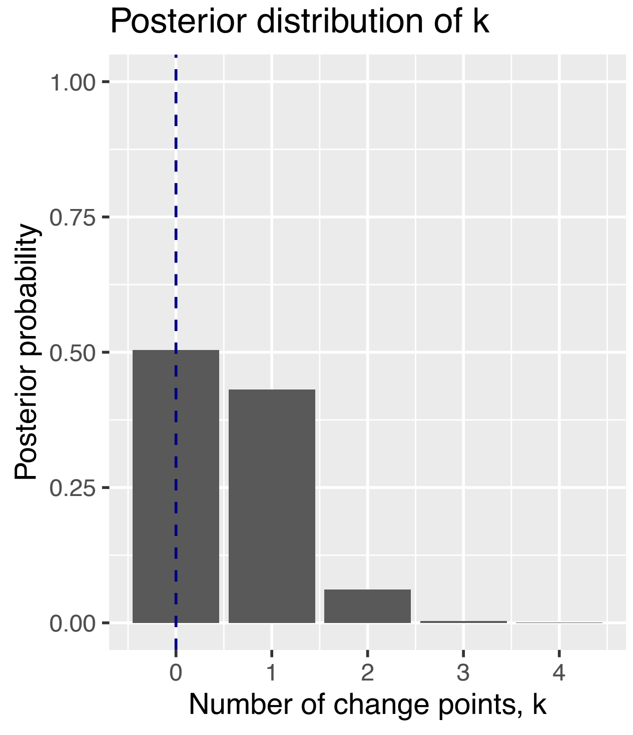

	State 1
Change point	0
Velocity	0.2353
Std dev	0.1513

DDB particle: 12

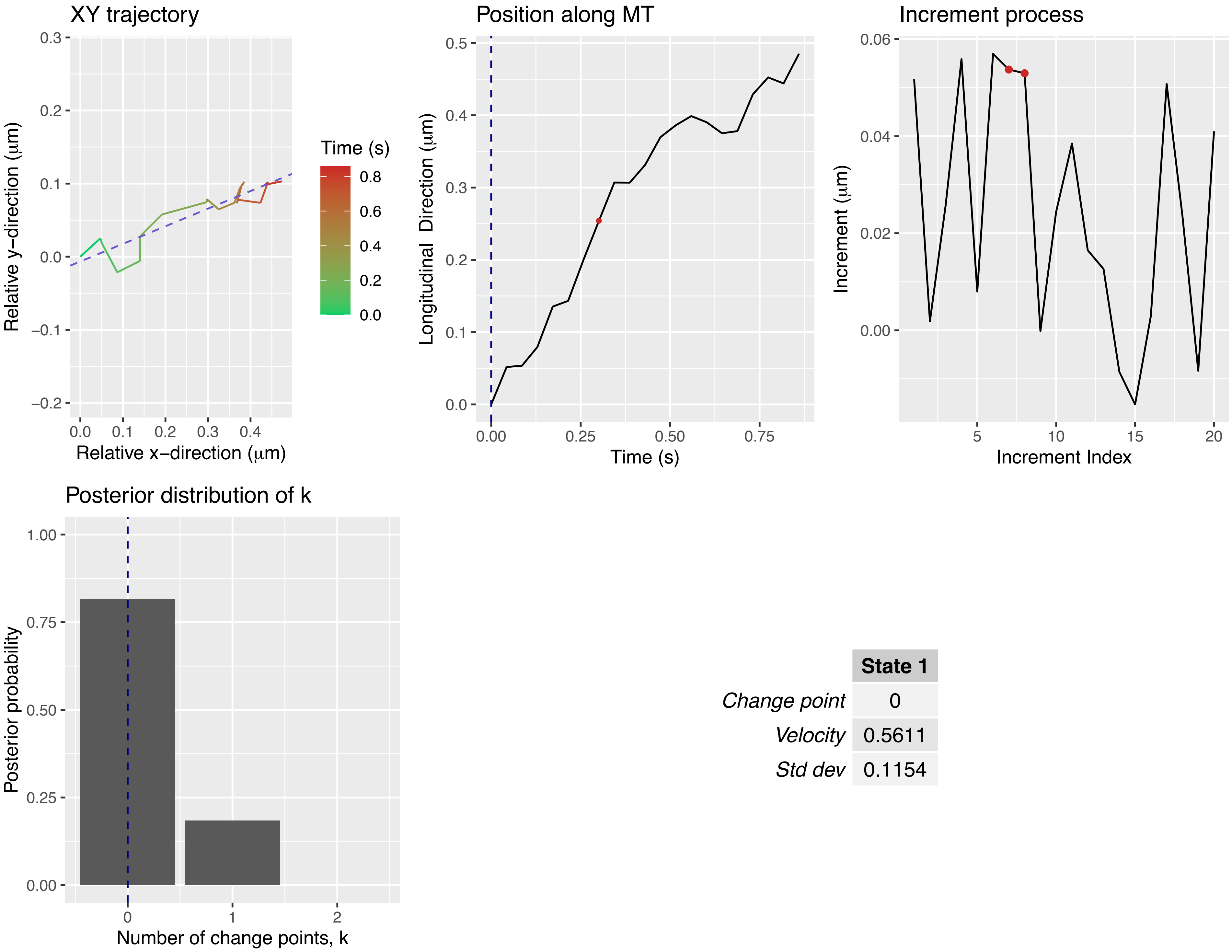

	State 1
Change point	0
Velocity	0.5611
Std dev	0.1154

DDB particle: 13

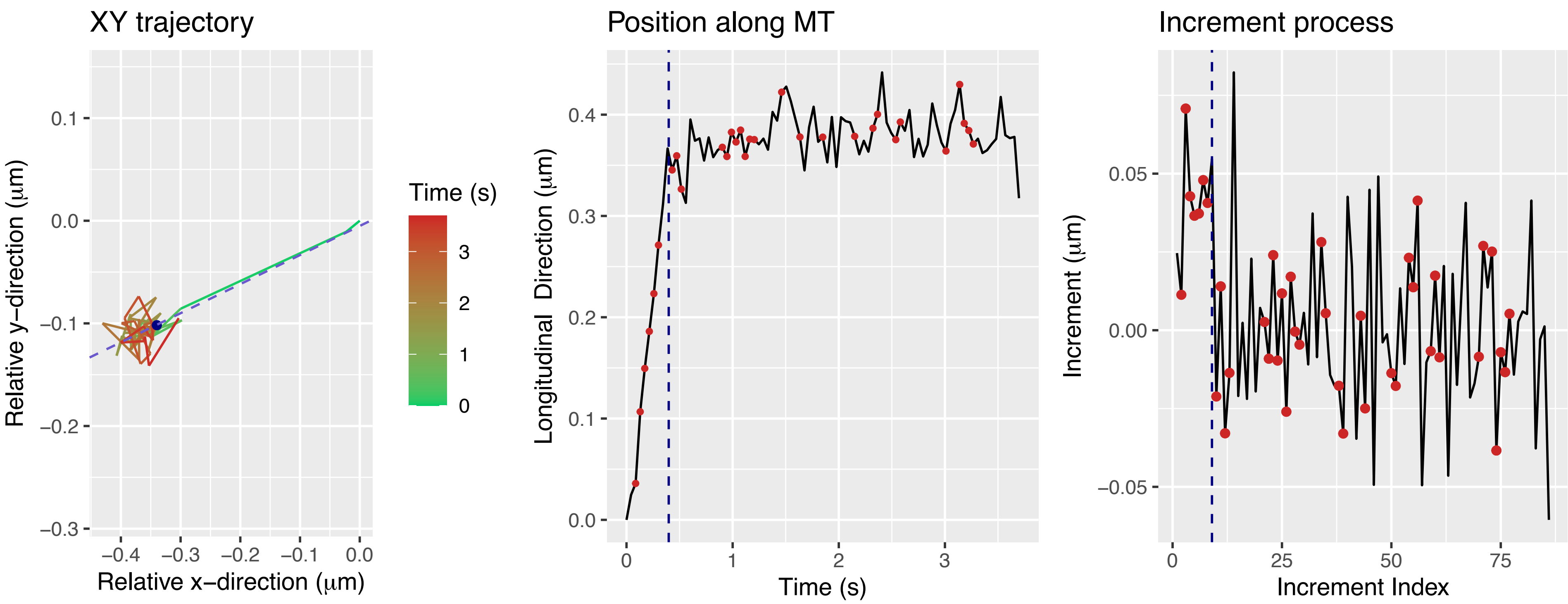

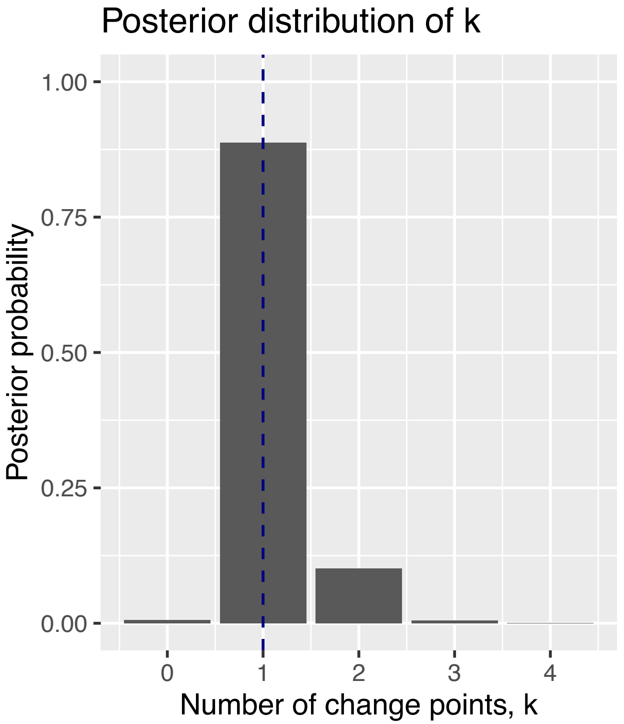

	State 1	State 2
Change point	0.3977	NA
Velocity	0.9516	−0.0147
Std dev	0.1209	NA

DDB particle: 14

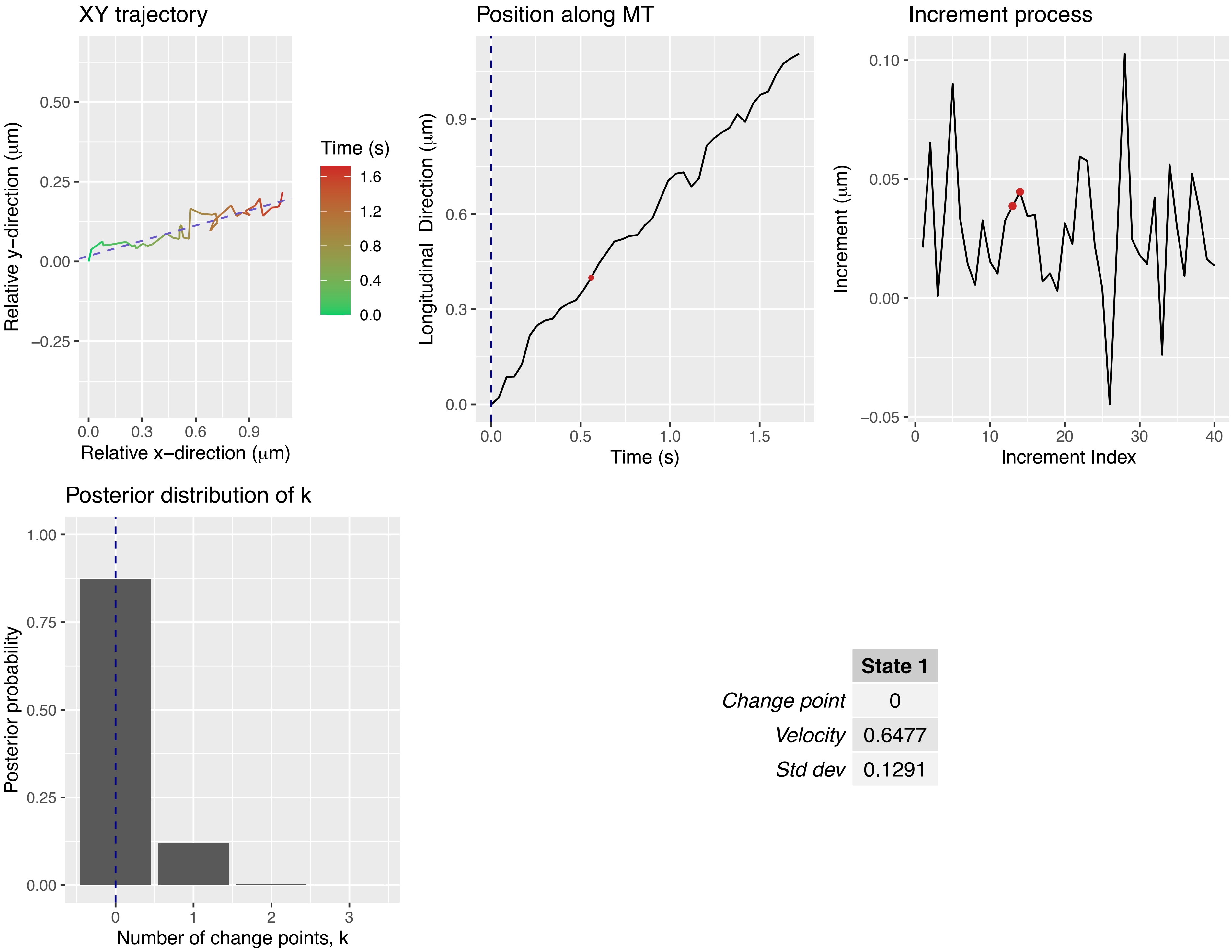

	**State 1**
Change point	0
Velocity	0.6477
Std dev	0.1291

DDB particle: 15

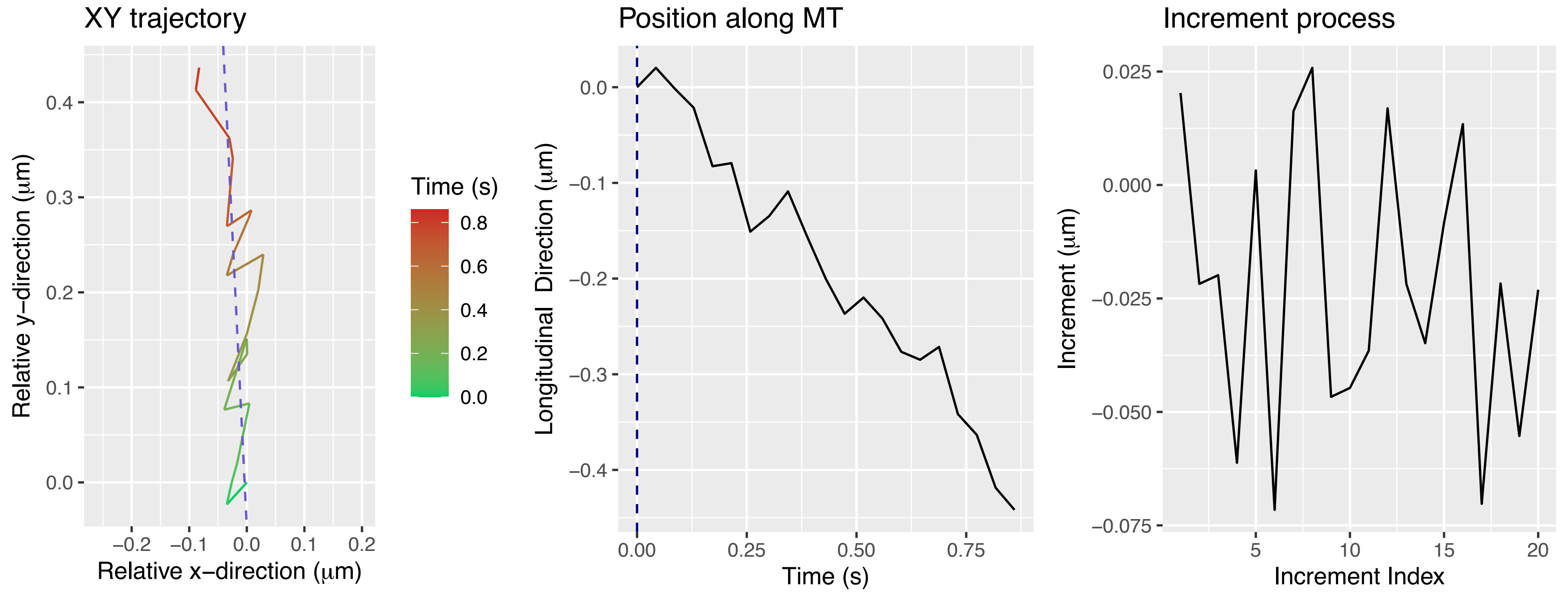

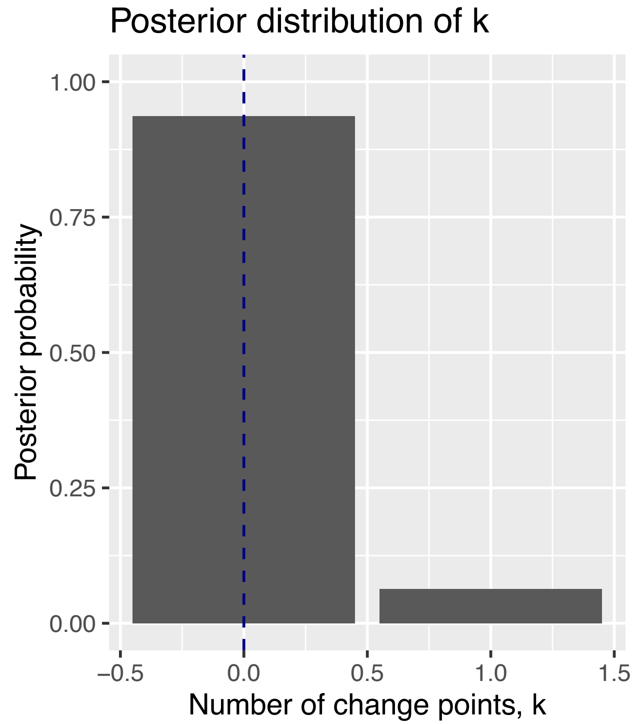

	State 1
Change point	0
Velocity	−0.5102
Std dev	0.1502

DDB particle: 16

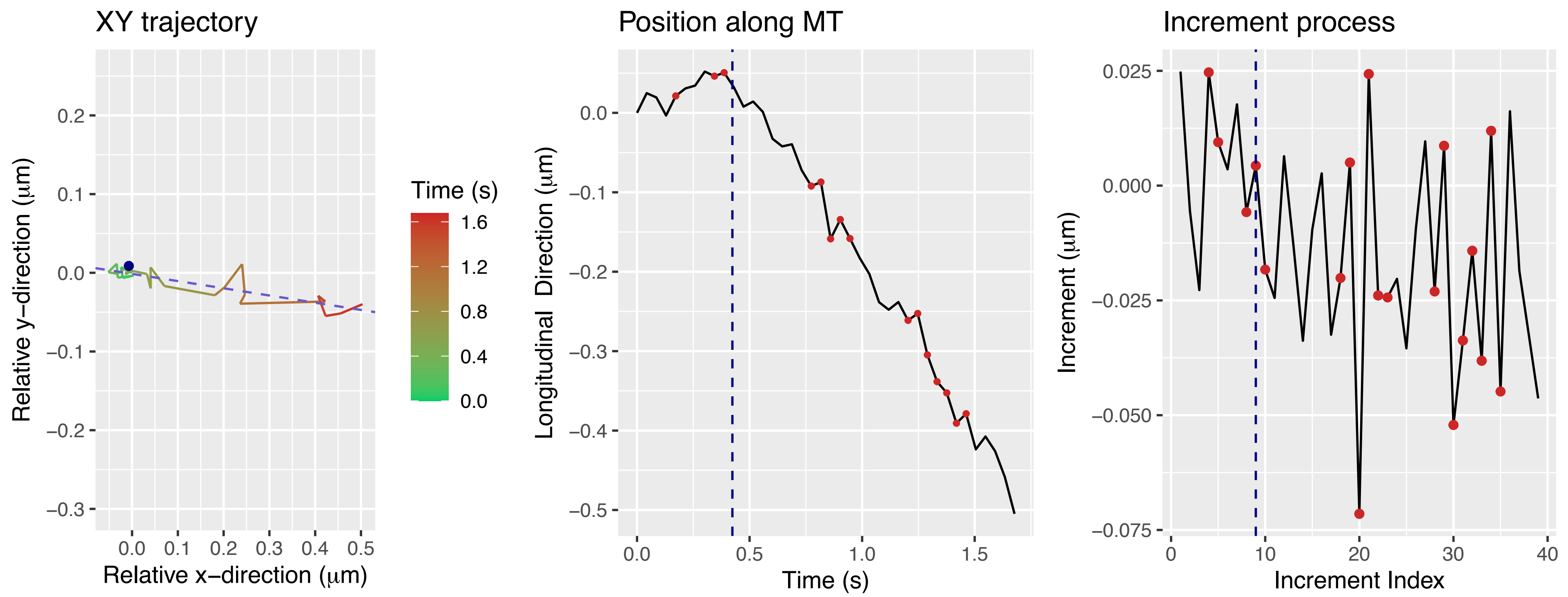

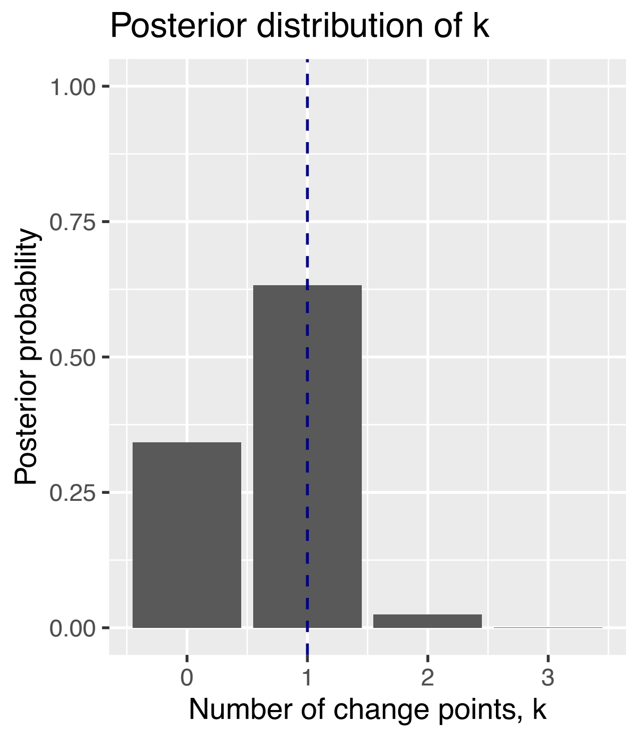

	State 1	State 2
Change point	0.4236	NA
Velocity	0.1518	−0.4193
Std dev	0.1028	NA

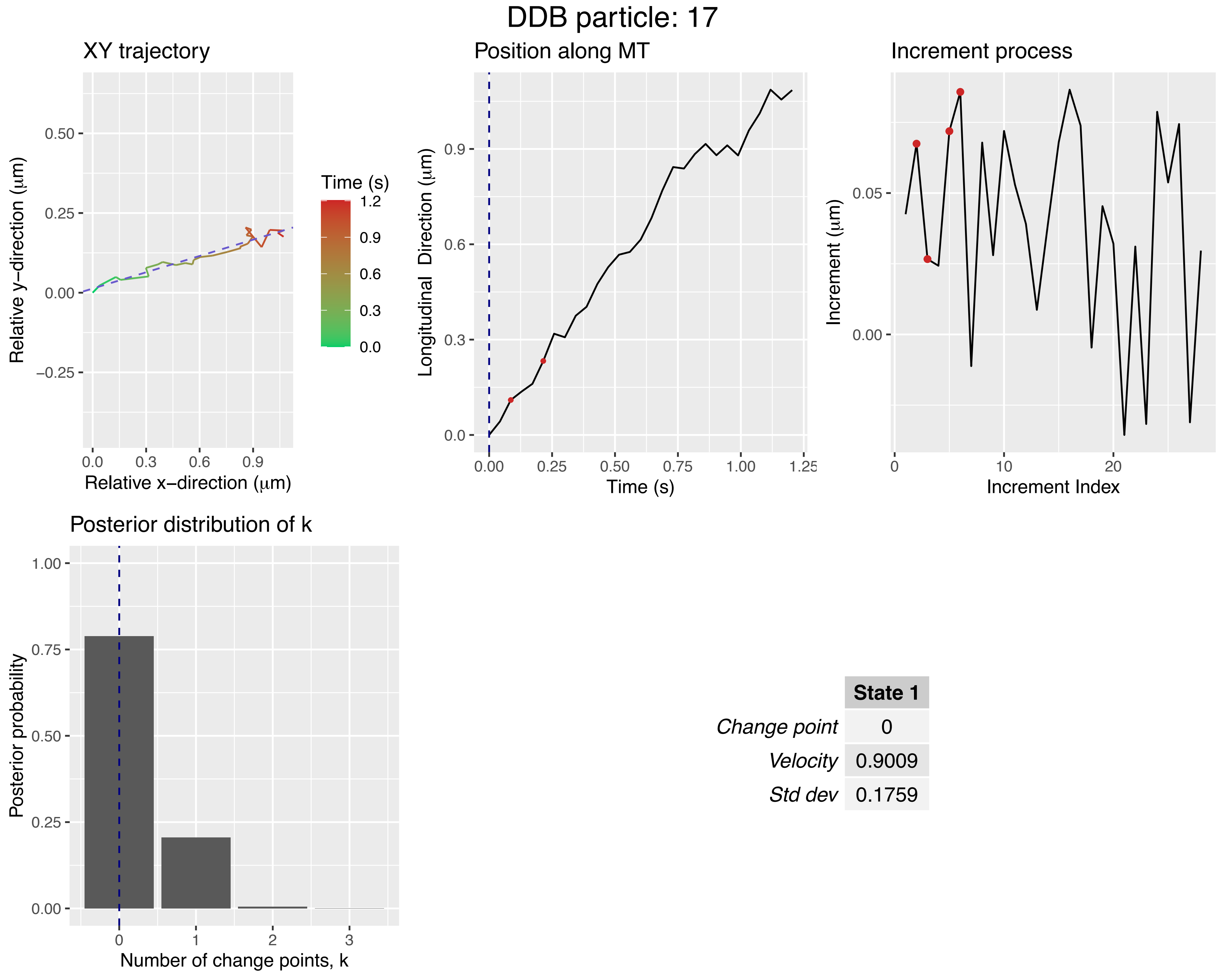

	State 1
Change point	0
Velocity	0.9009
Std dev	0.1759

DDB particle: 18

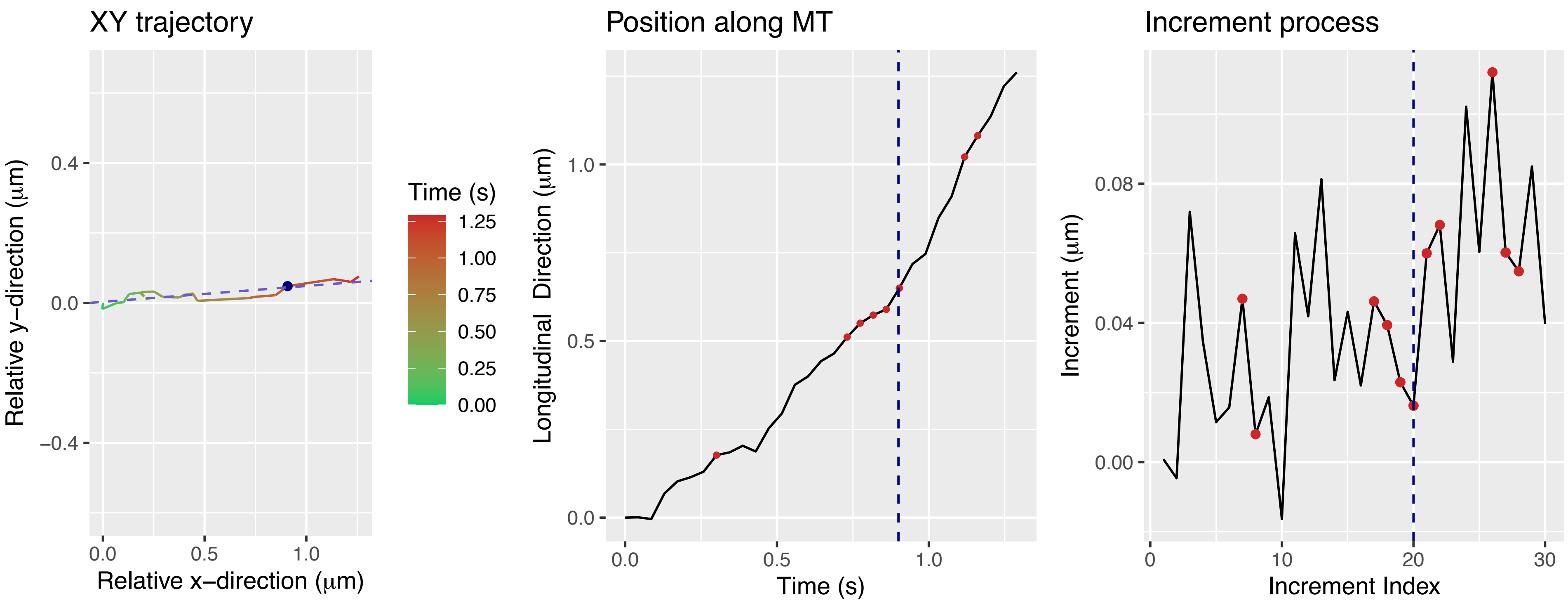

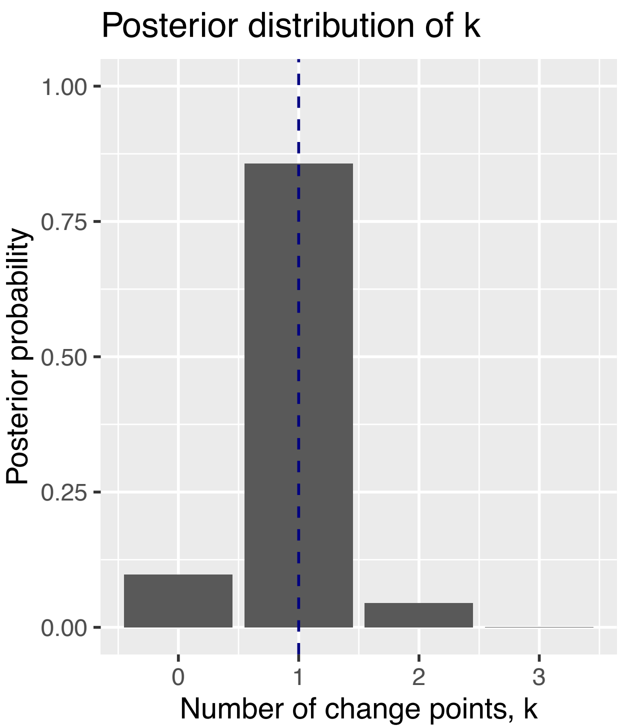

	State 1	State 2
Change point	0.9	NA
Velocity	0.6878	1.5572
Std dev	0.1289	NA

DDB particle: 19

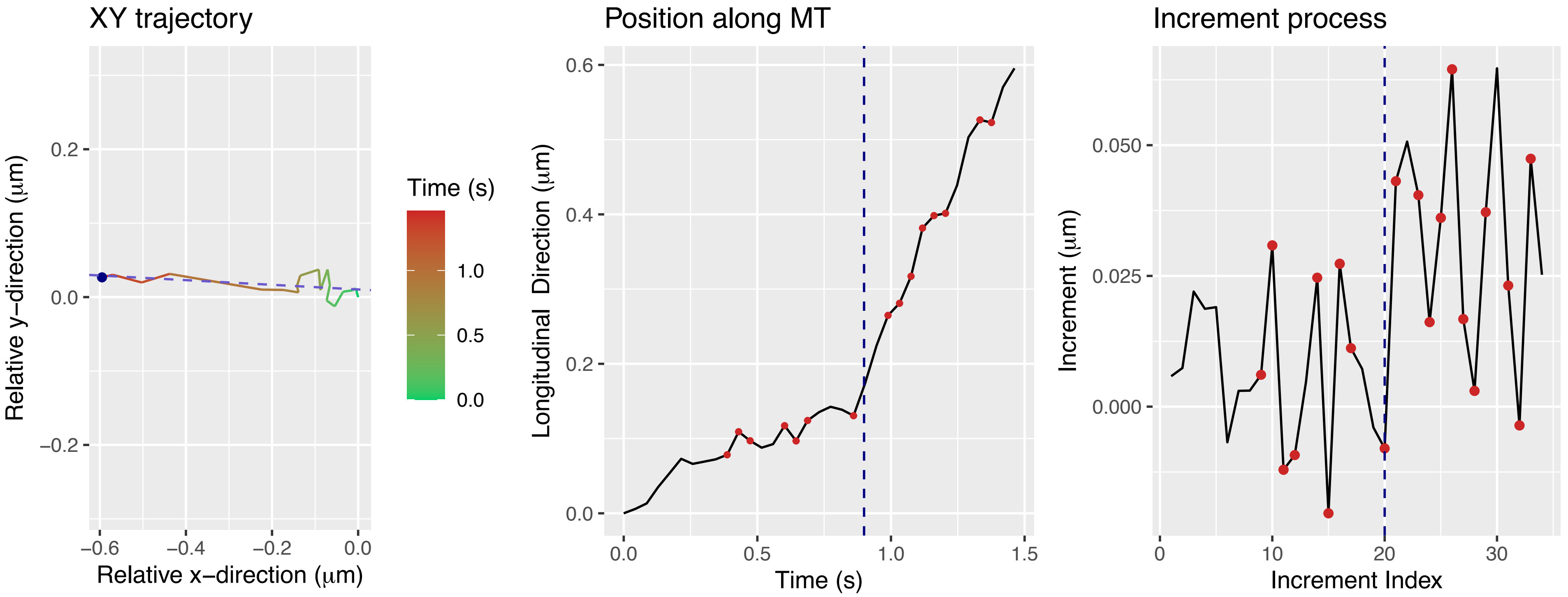

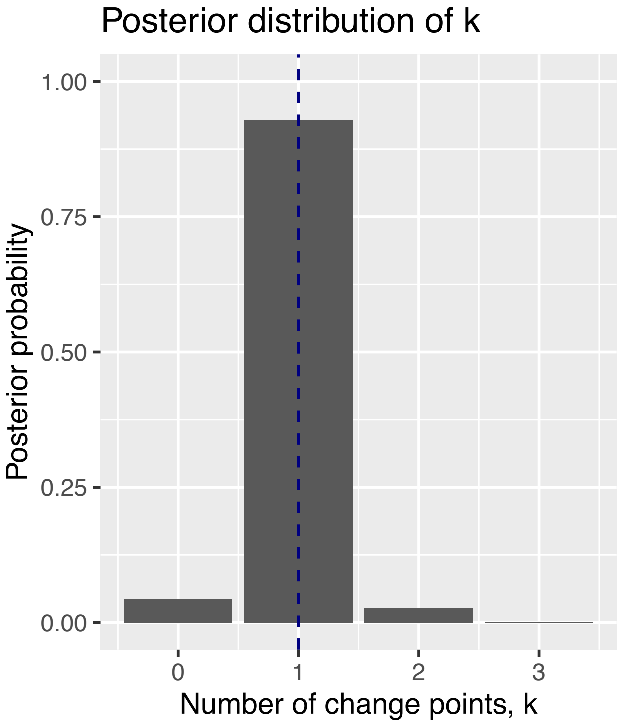

	State 1	State 2
Change point	0.8993	NA
Velocity	0.1456	0.7752
Std dev	0.0847	NA

DDB particle: 20

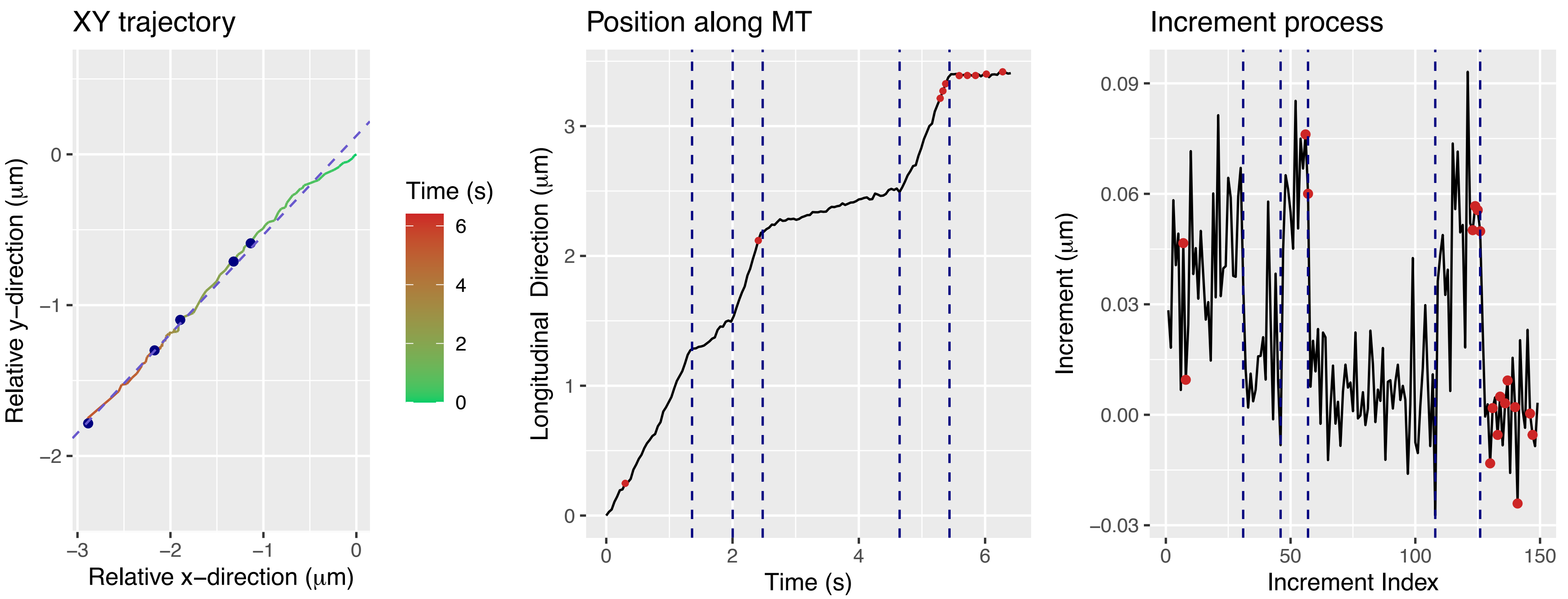

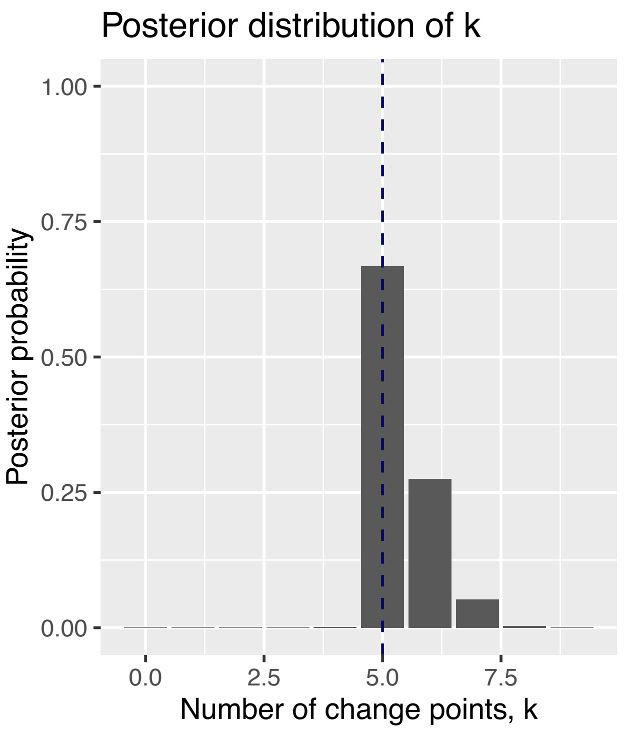

	State 1	State 2	State 3	State 4	State 5	State 6
Change point	1.3593	2.0025	2.4774	4.6464	5.4362	NA
Velocity	0.9885	0.3233	1.4314	0.1533	1.1489	0.0509
Std dev	0.072	NA	NA	NA	NA	NA

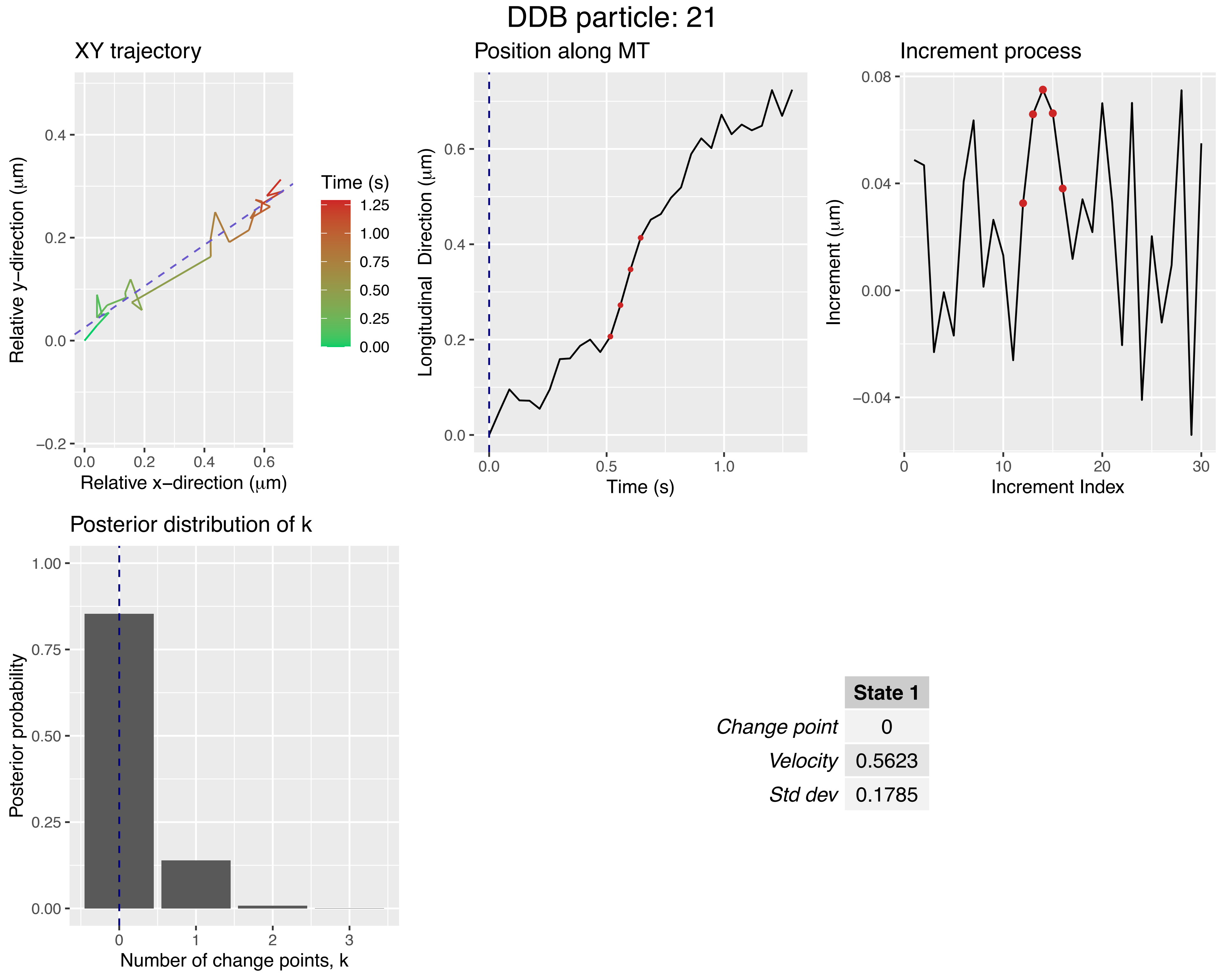

	State 1
Change point	0
Velocity	0.5623
Std dev	0.1785

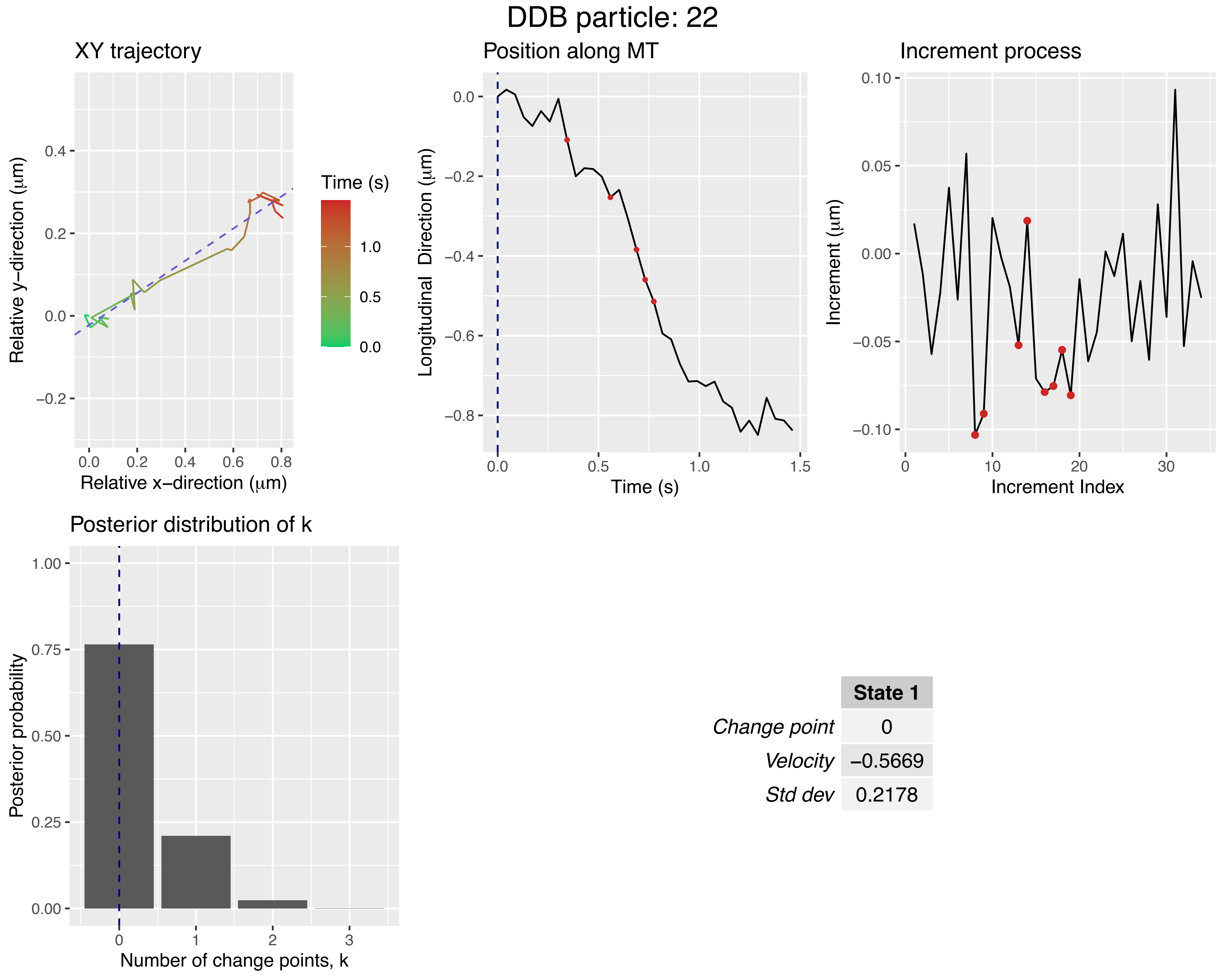

	State 1
Change point	0
Velocity	−0.5669
Std dev	0.2178

DDB particle: 23

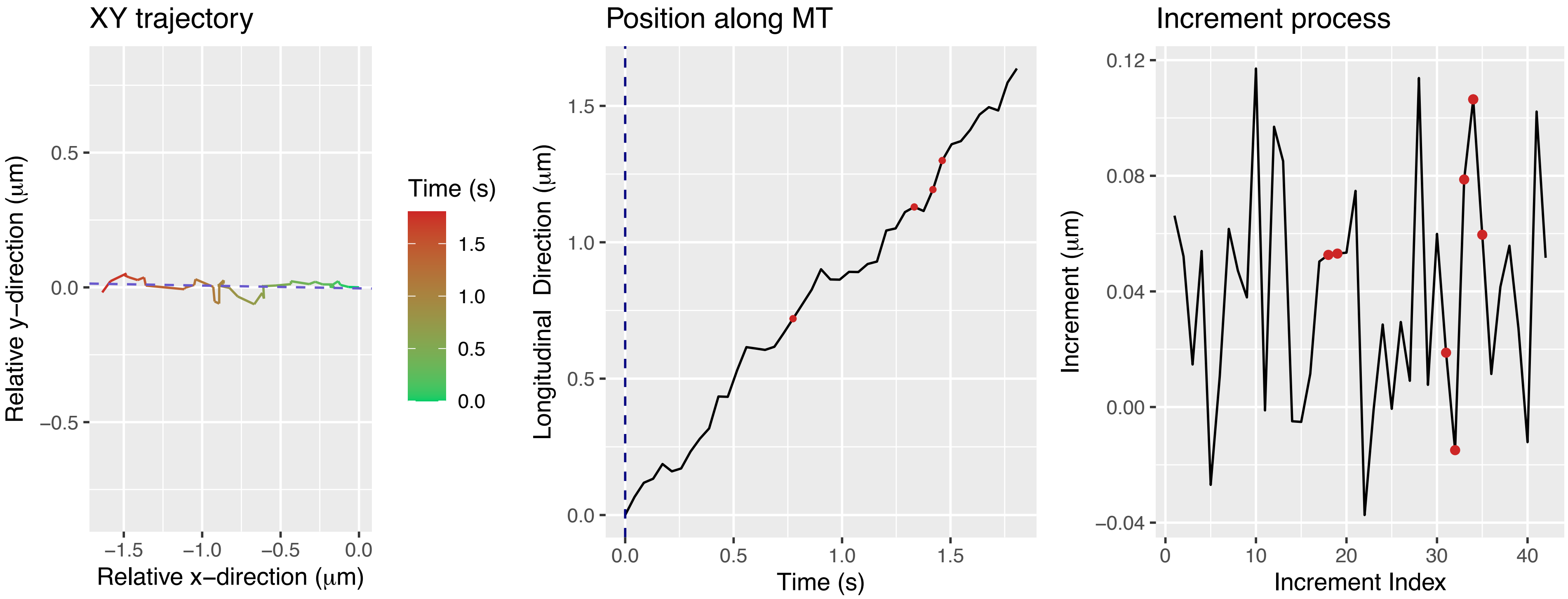

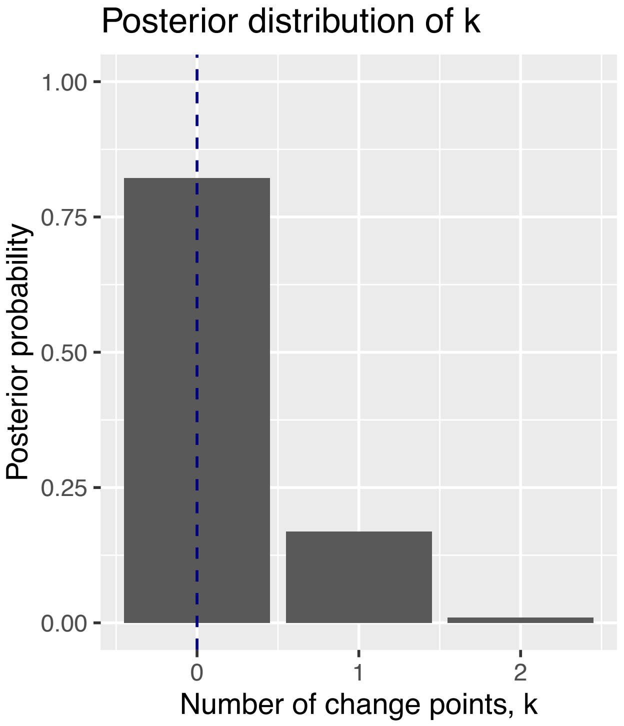

	State 1
Change point	0
Velocity	0.9048
Std dev	0.1894

DDB particle: 24

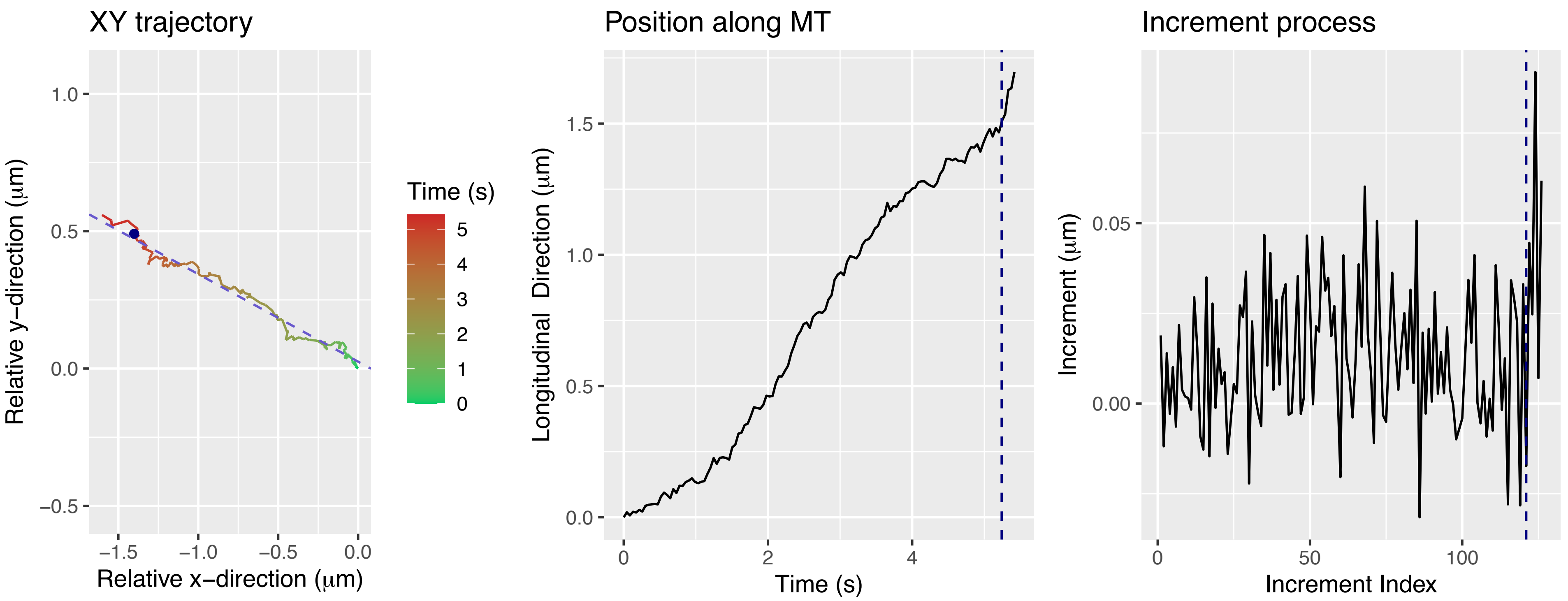

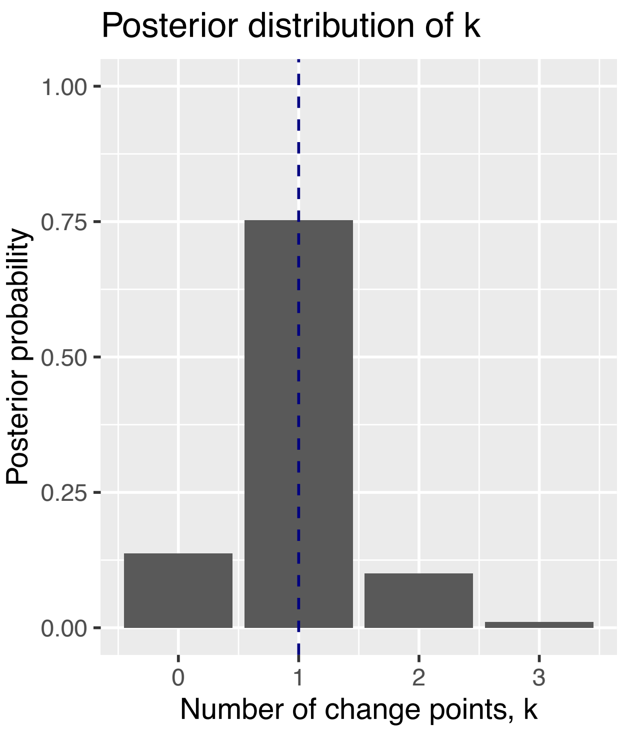

	State 1	State 2
Change point	5.2408	NA
Velocity	0.2789	1.078
Std dev	0.0933	NA

DDB particle: 25

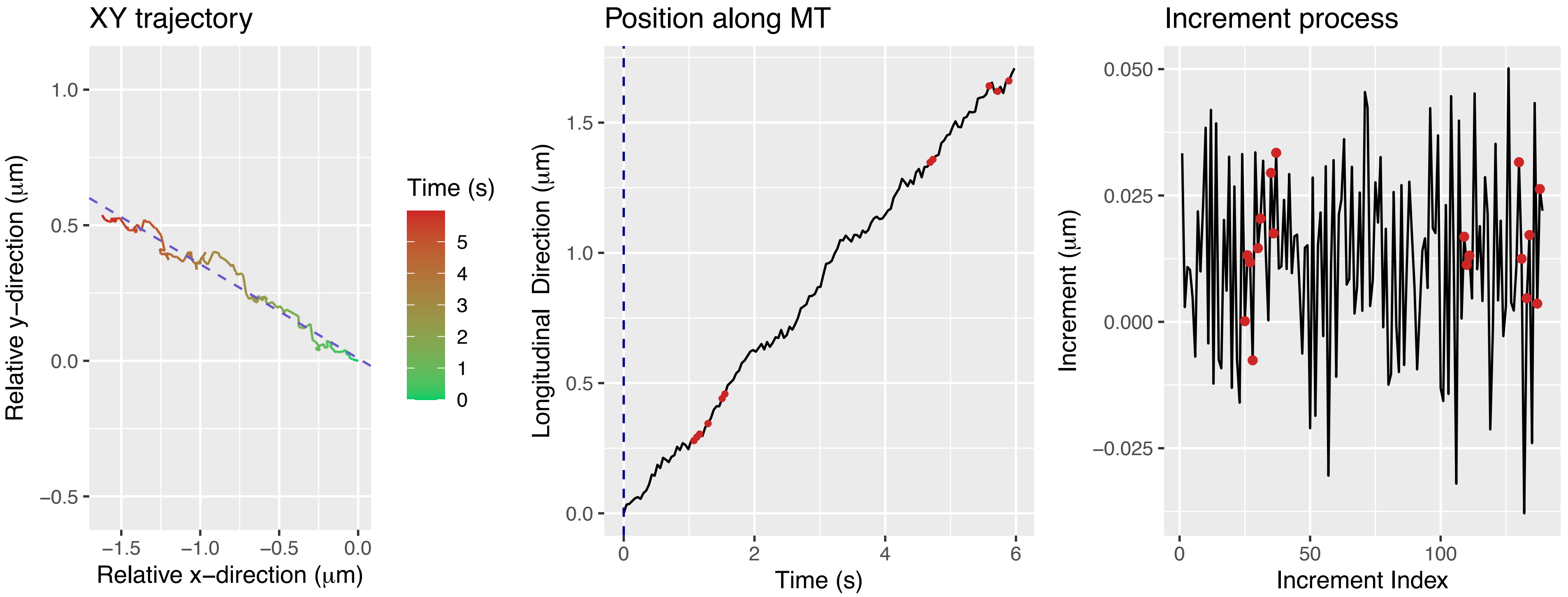

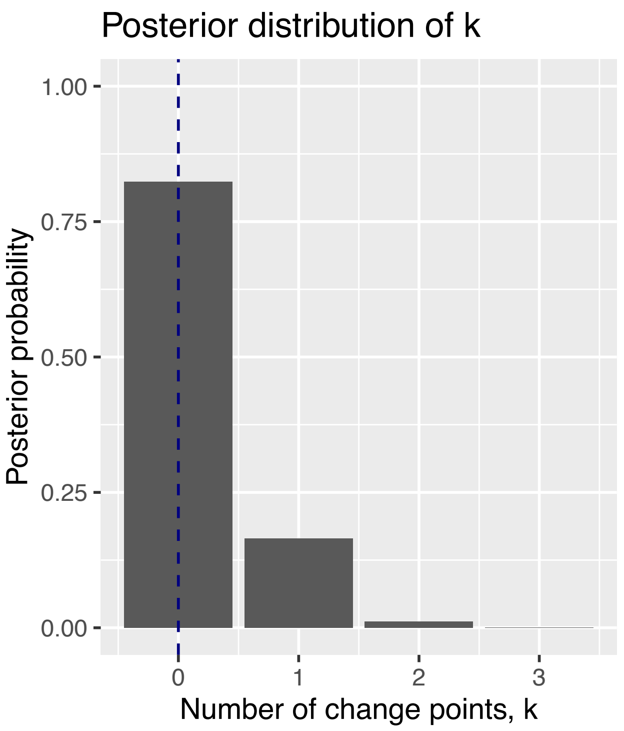

	State 1
Change point	0
Velocity	0.2855
Std dev	0.086

DDB particle: 26

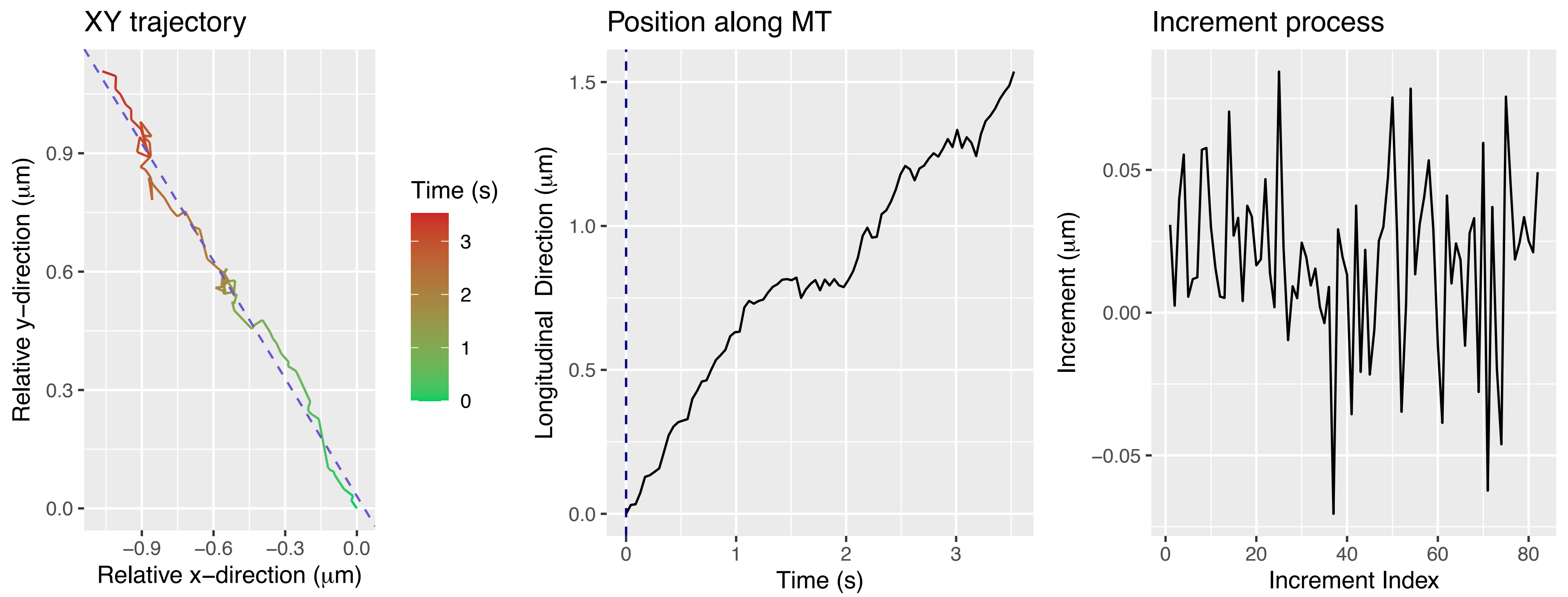

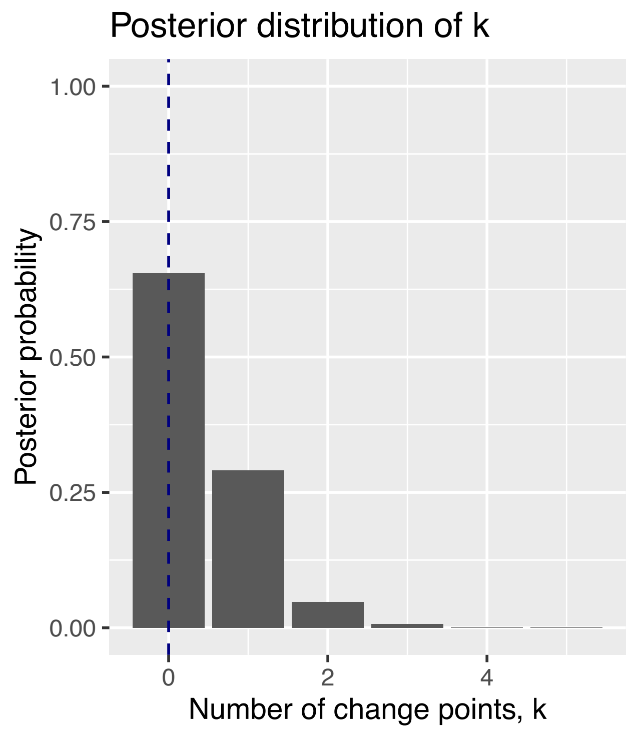

	State 1
Change point	0
Velocity	0.4365
Std dev	0.1453

DDB particle: 27

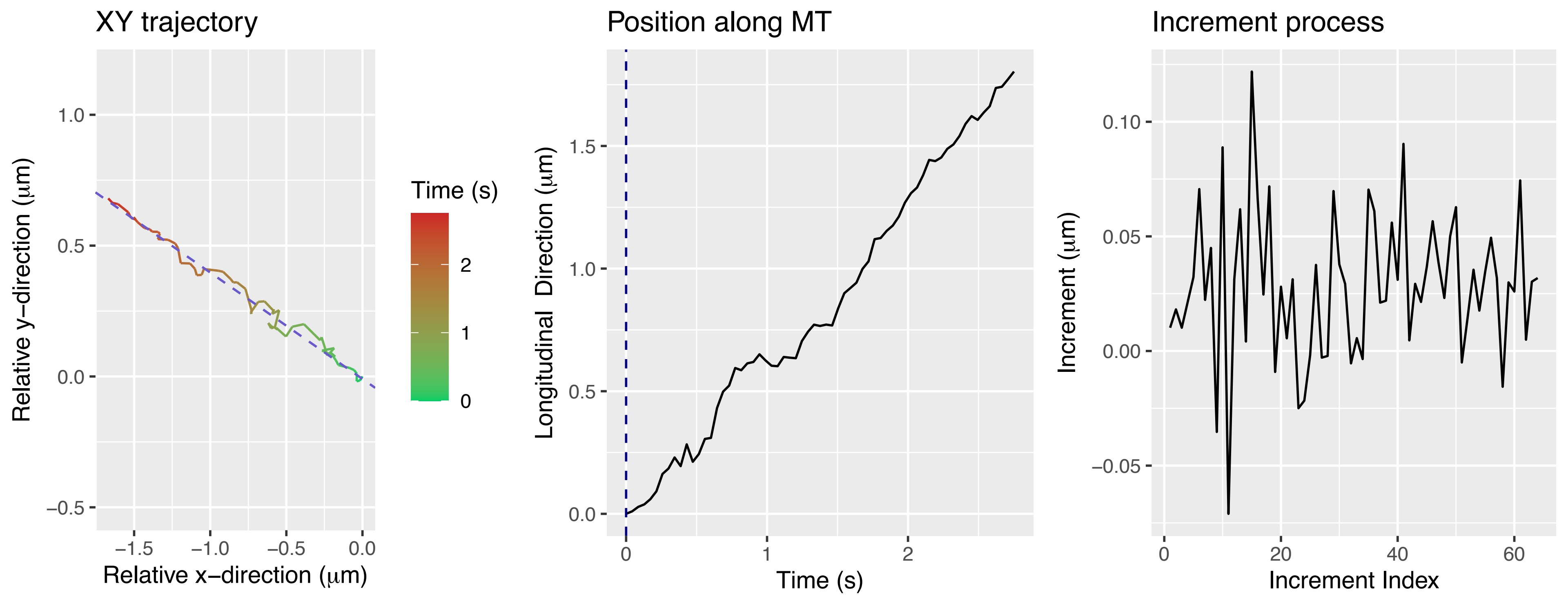

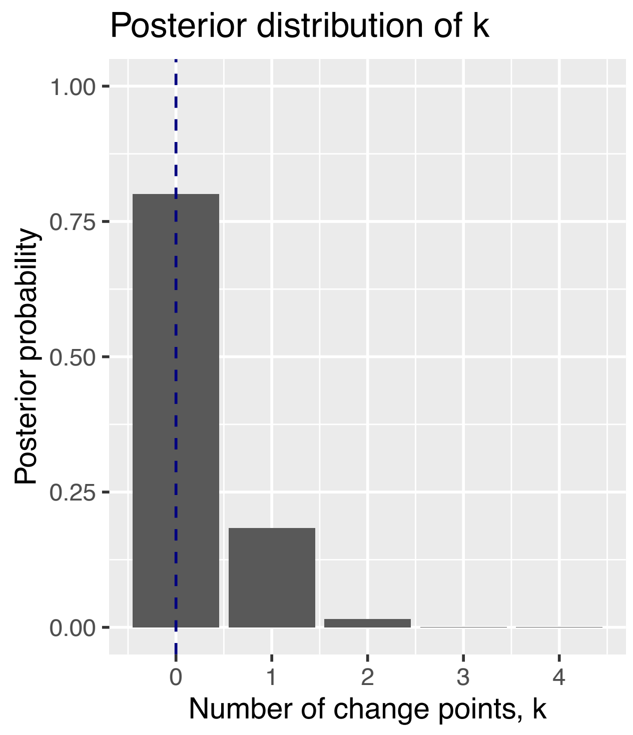

	State 1
Change point	0
Velocity	0.6532
Std dev	0.1582

DDB particle: 28

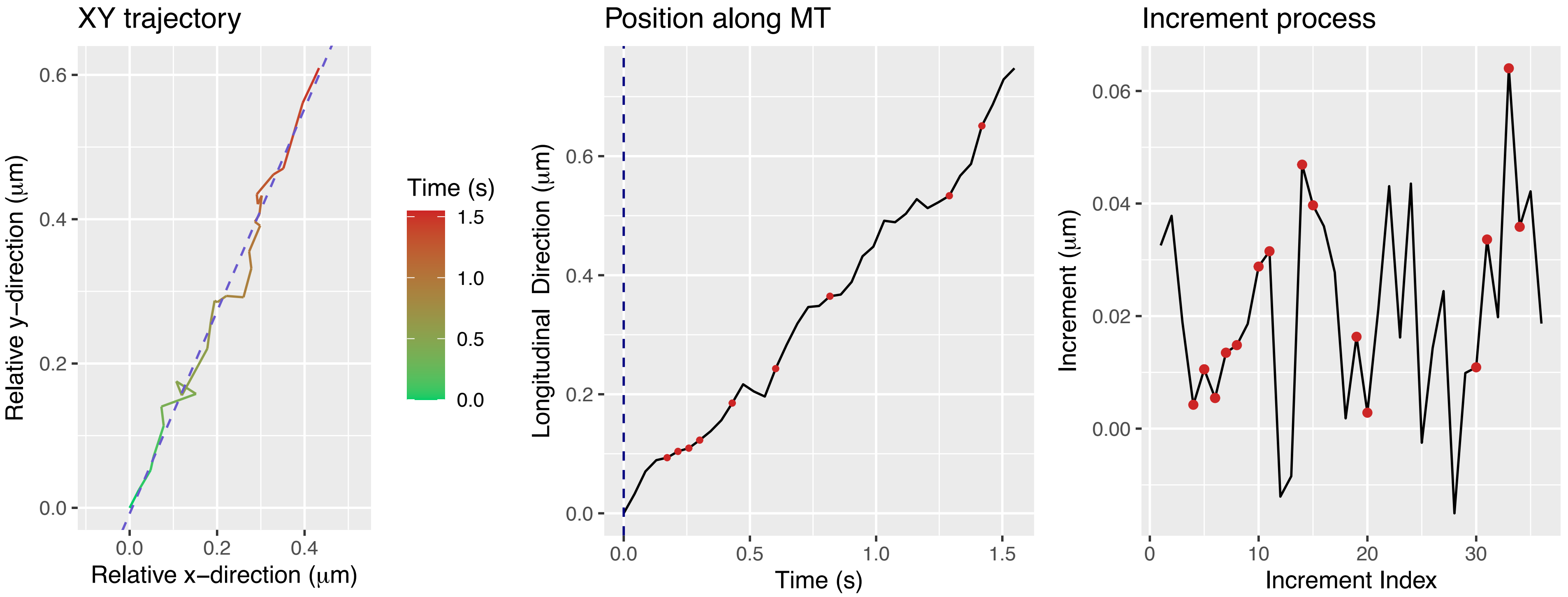

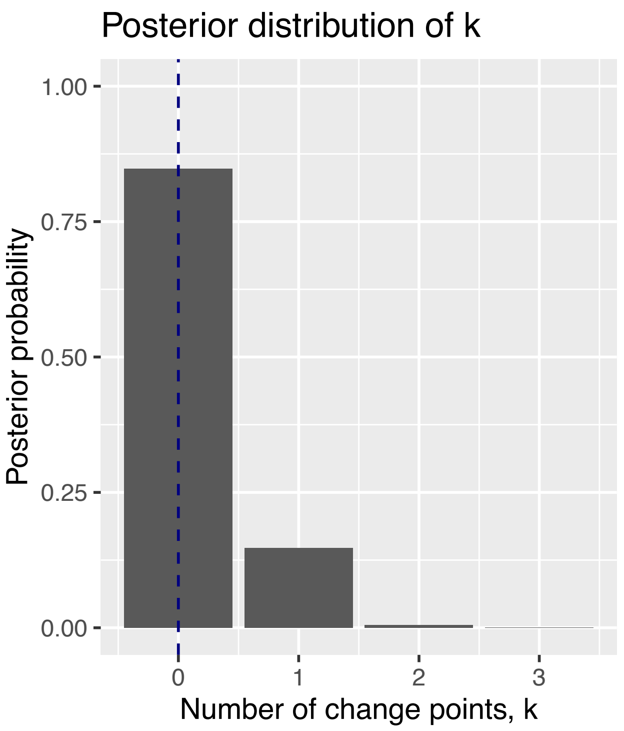

	State 1
Change point	0
Velocity	0.4829
Std dev	0.083

DDB particle: 29

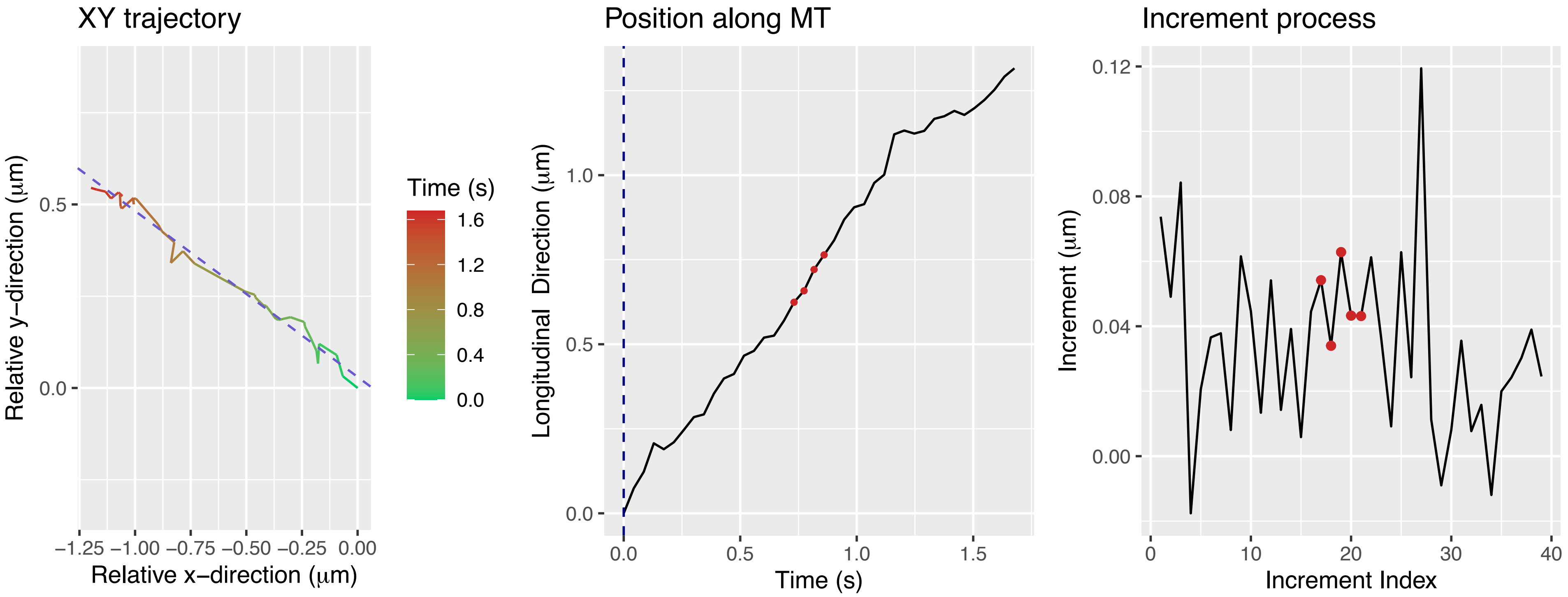

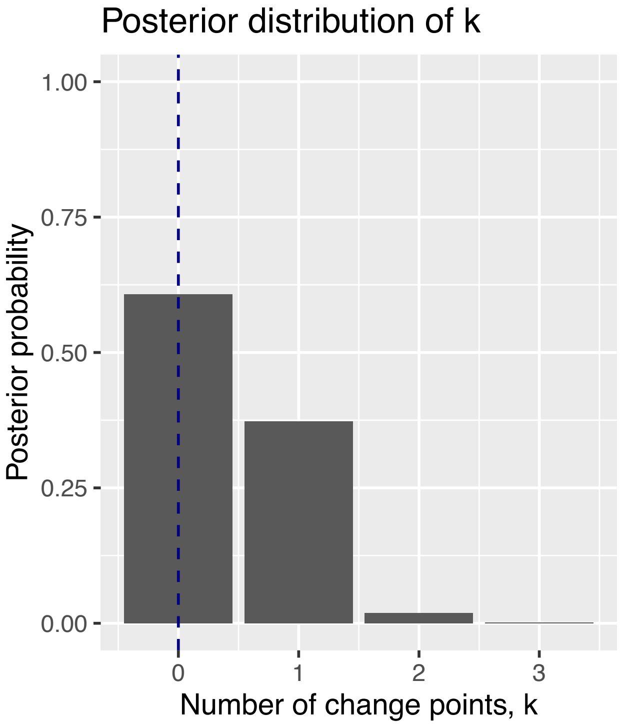

	State 1
Change point	0
Velocity	0.7873
Std dev	0.132

DDB particle: 30

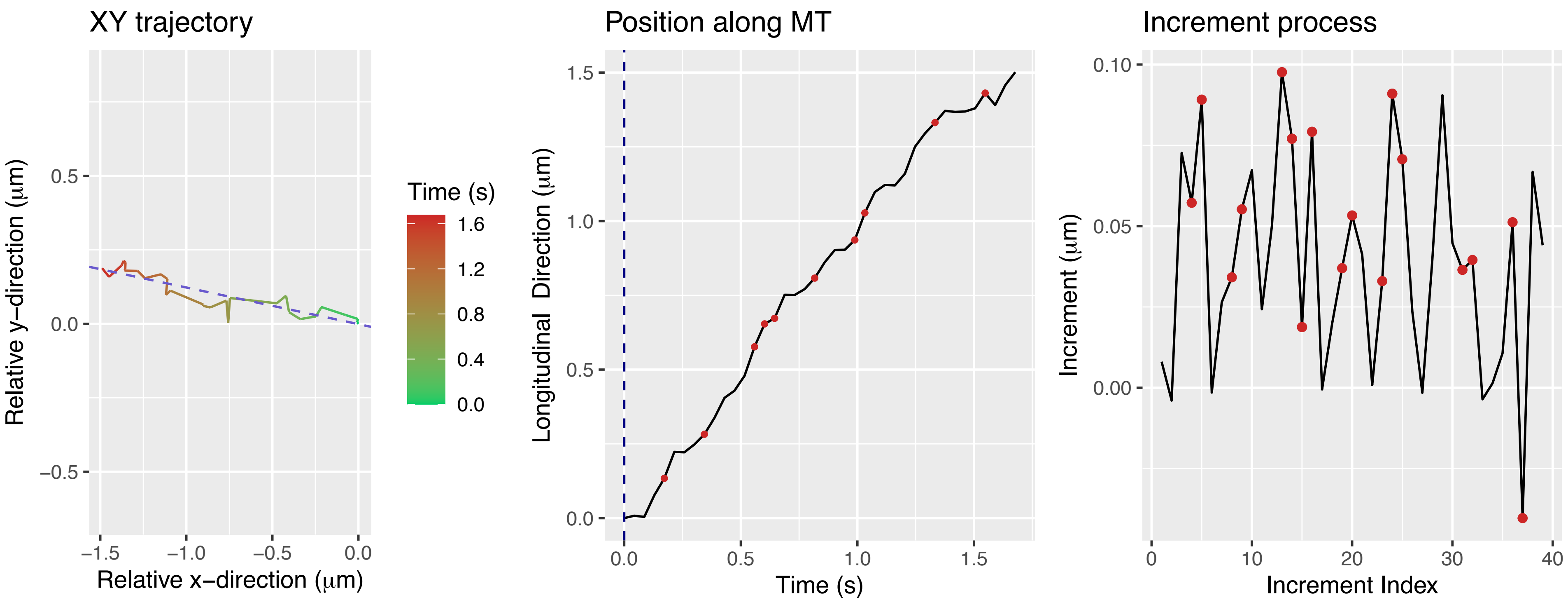

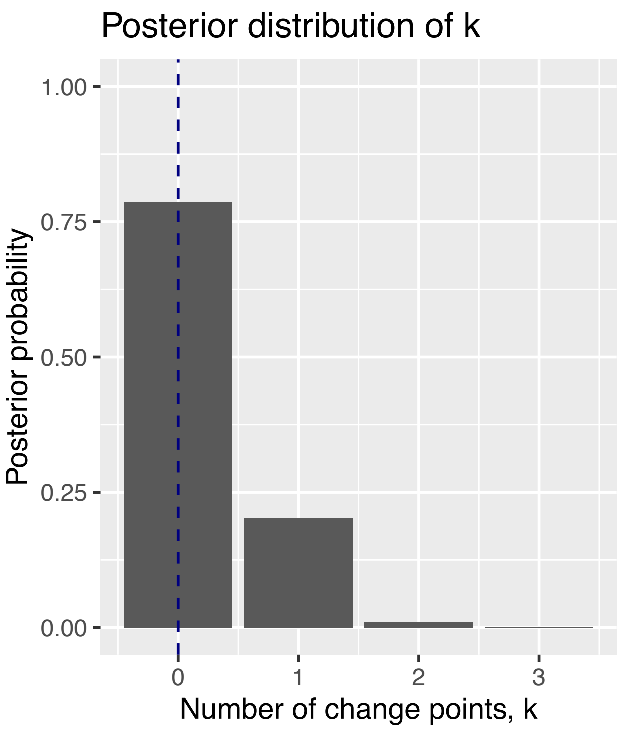

	State 1
Change point	0
Velocity	0.8911
Std dev	0.1594

DDB particle: 31

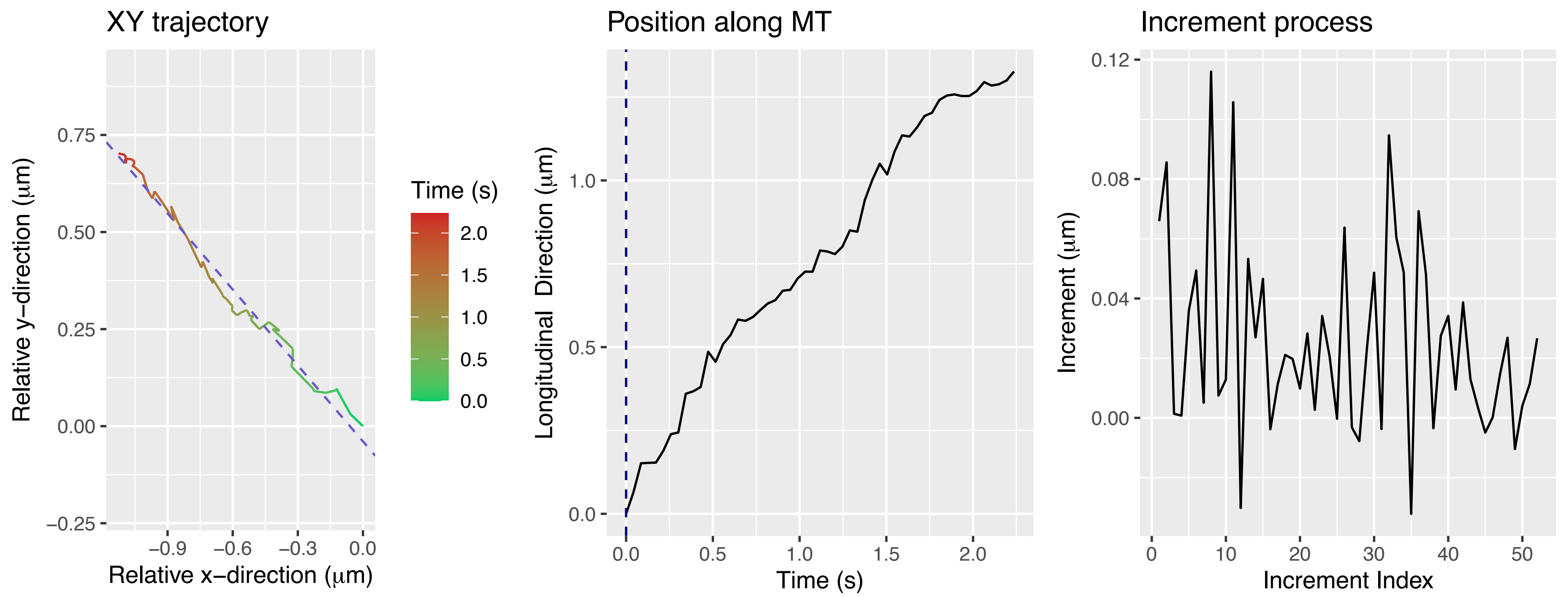

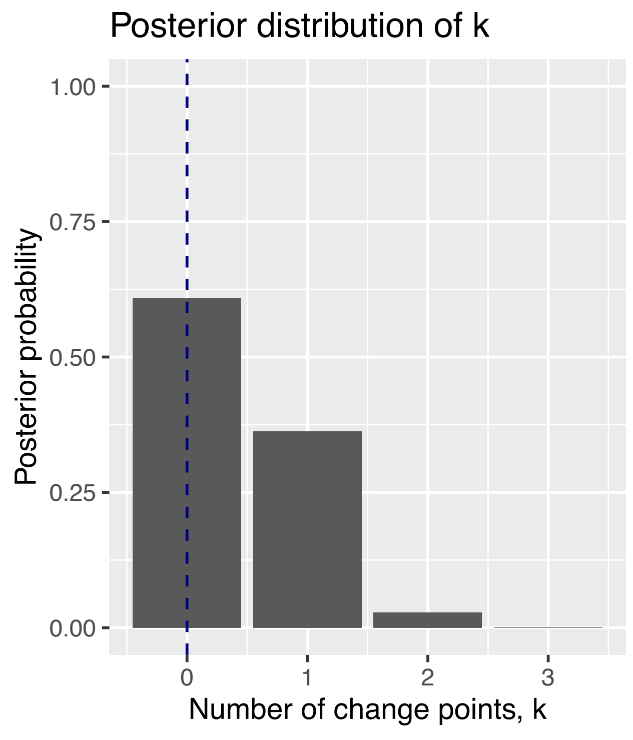

	State 1
Change point	0
Velocity	0.5943
Std dev	0.1553

DDB particle: 32

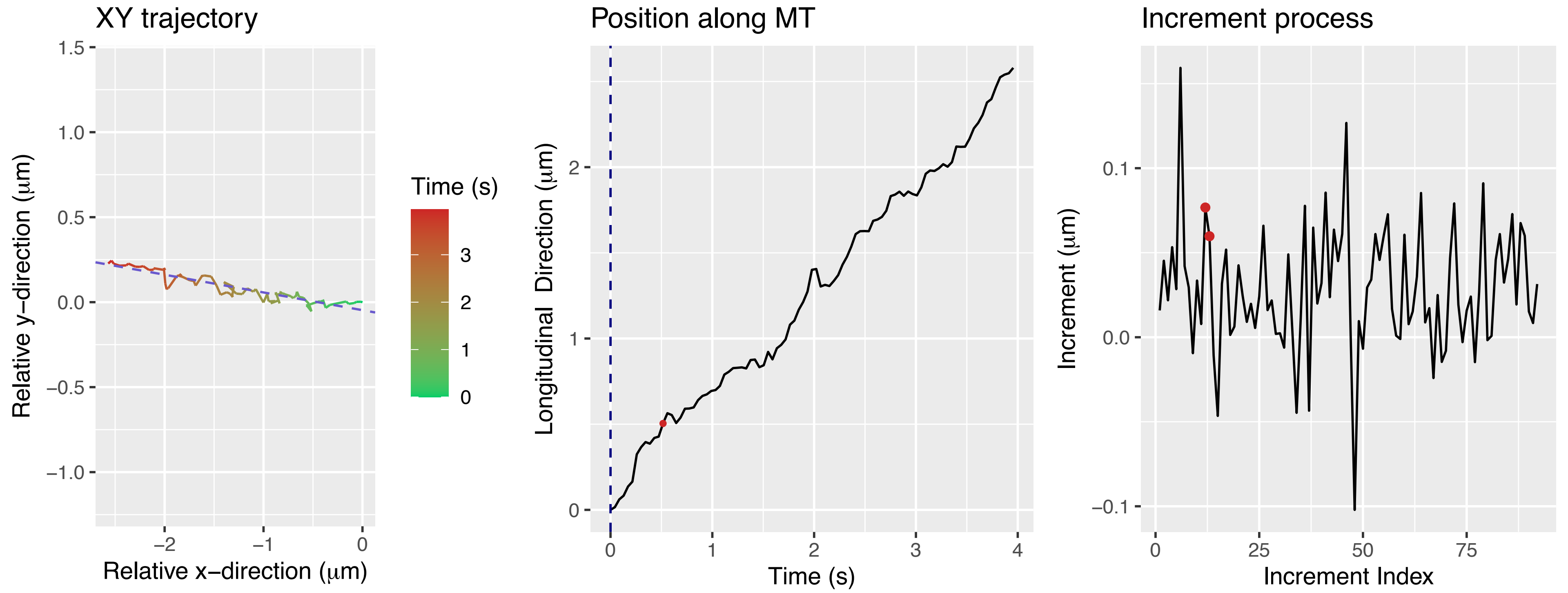

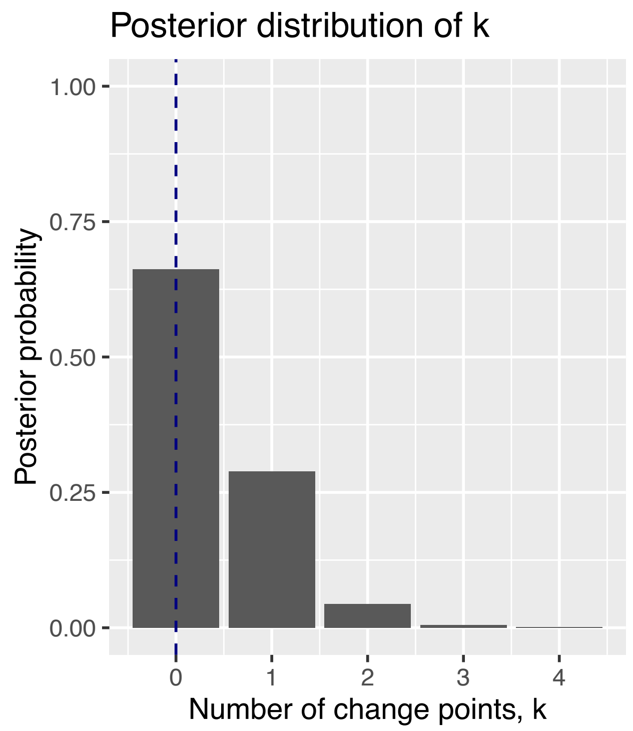

	State 1
Change point	0
Velocity	0.6476
Std dev	0.1788

DDB particle: 33

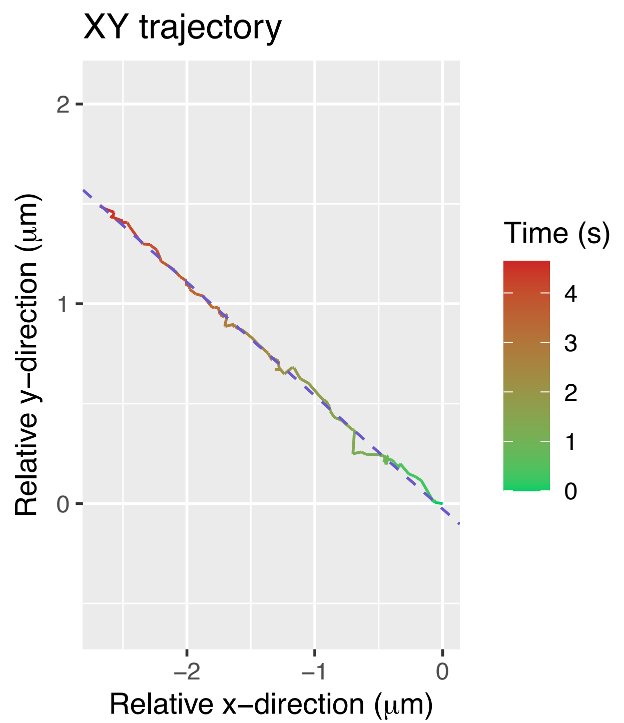

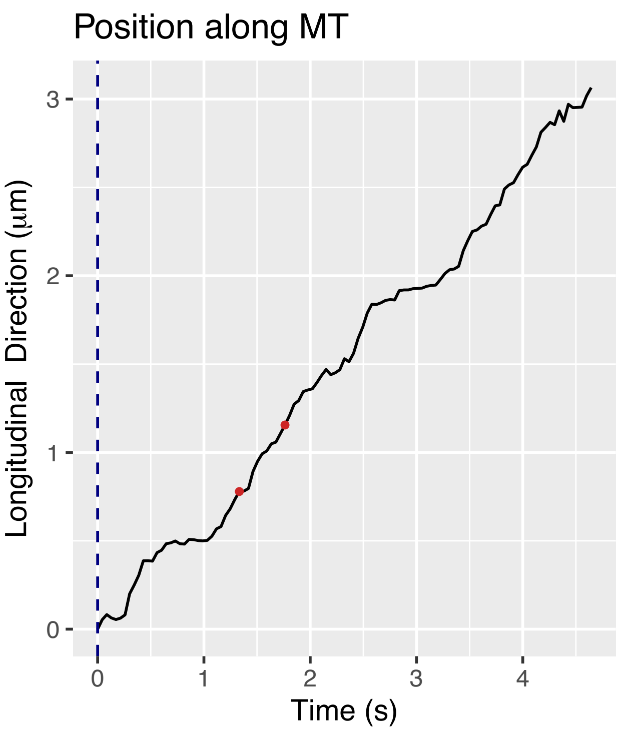

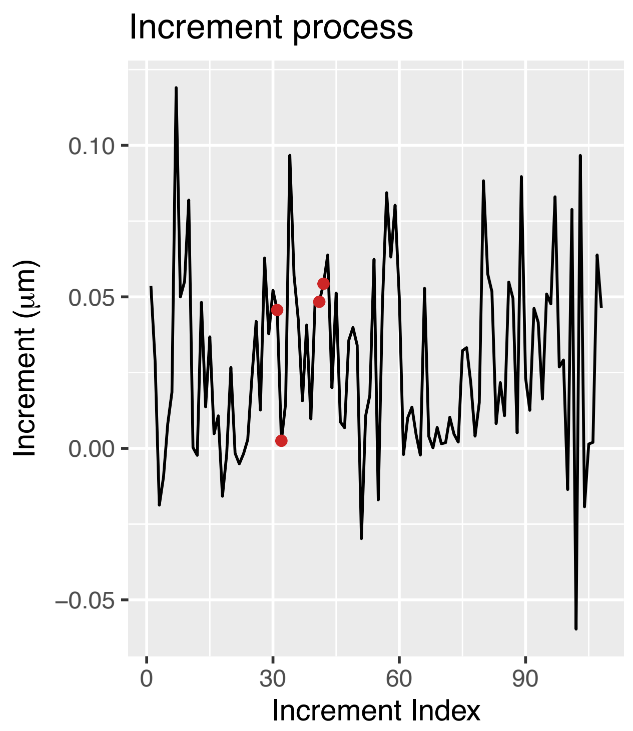

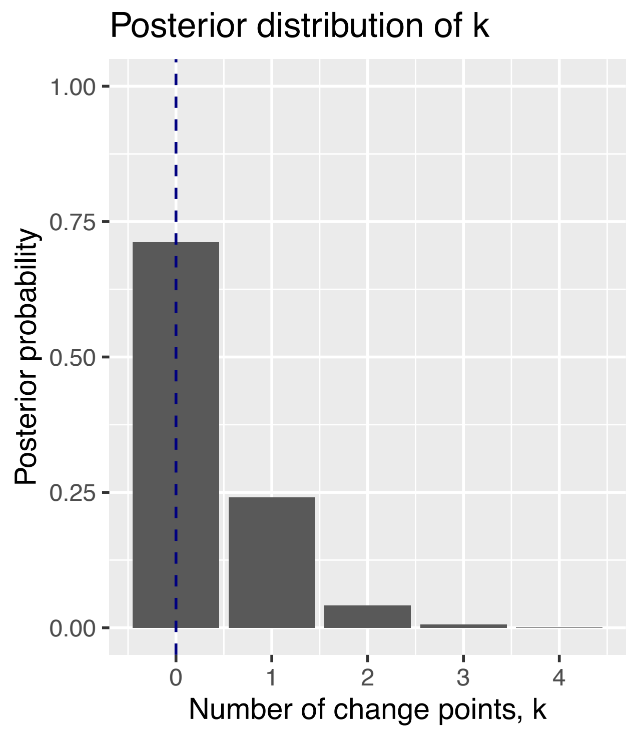

	**State 1**
Change point	0
Velocity	0.6604
Std dev	0.1494

DDB particle: 34

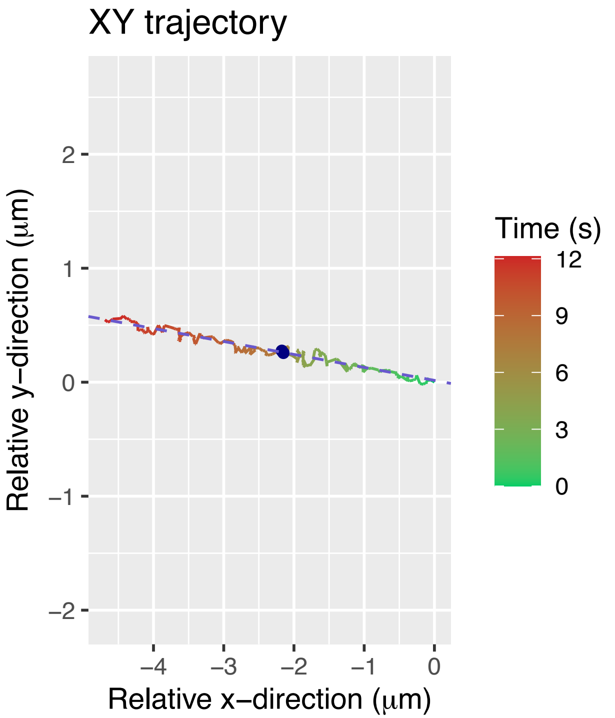

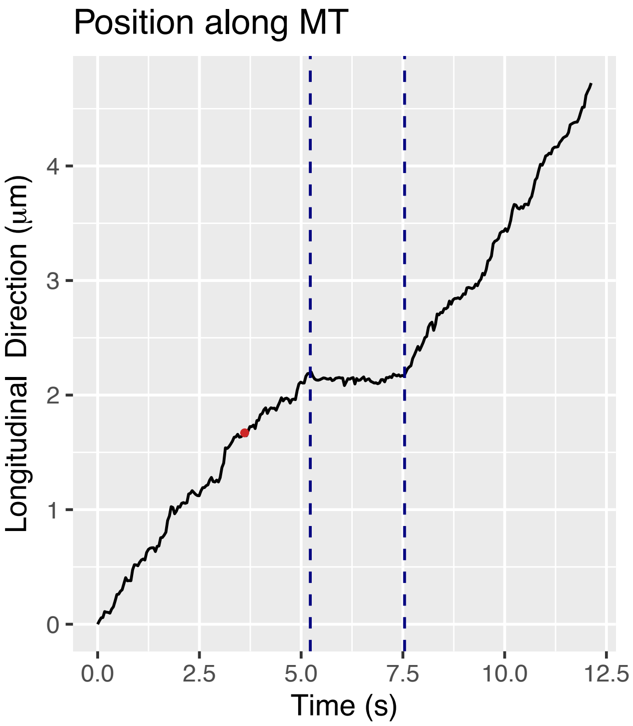

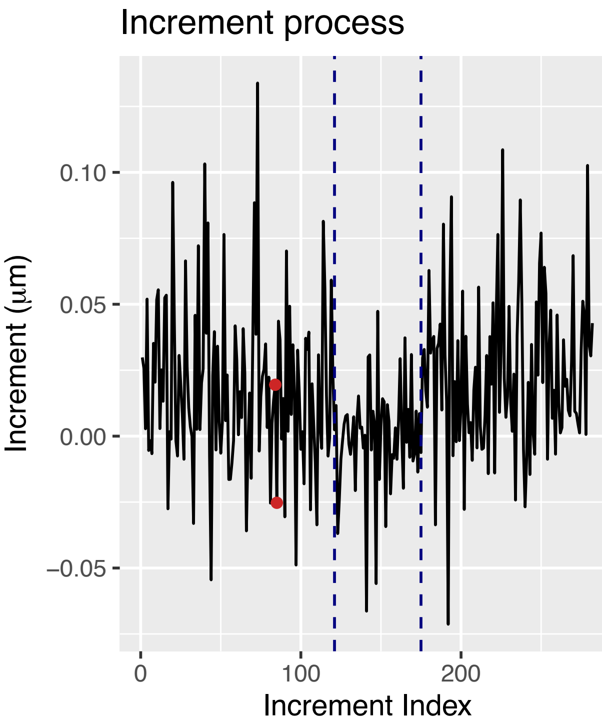

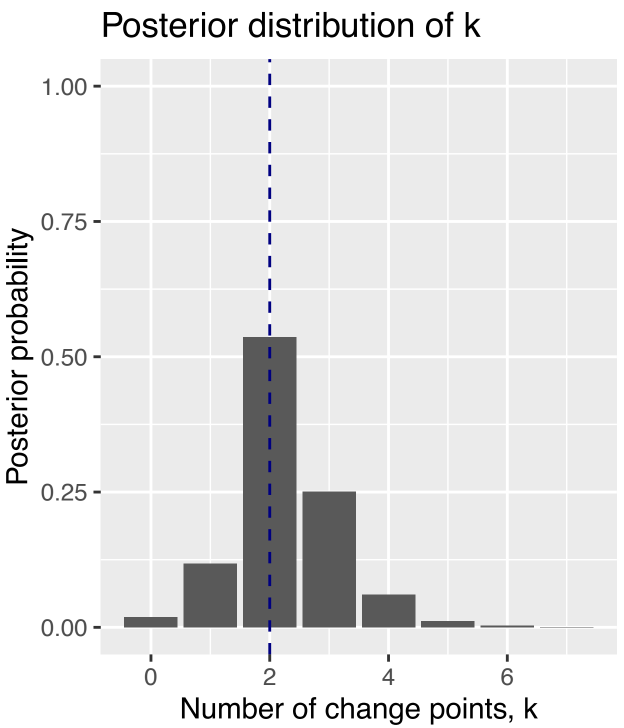

	State 1	State 2	State 3
Change point	5.2245	7.5412	NA
Velocity	0.4363	0.0219	0.4964
Std dev	0.1389	NA	NA

DDB particle: 35

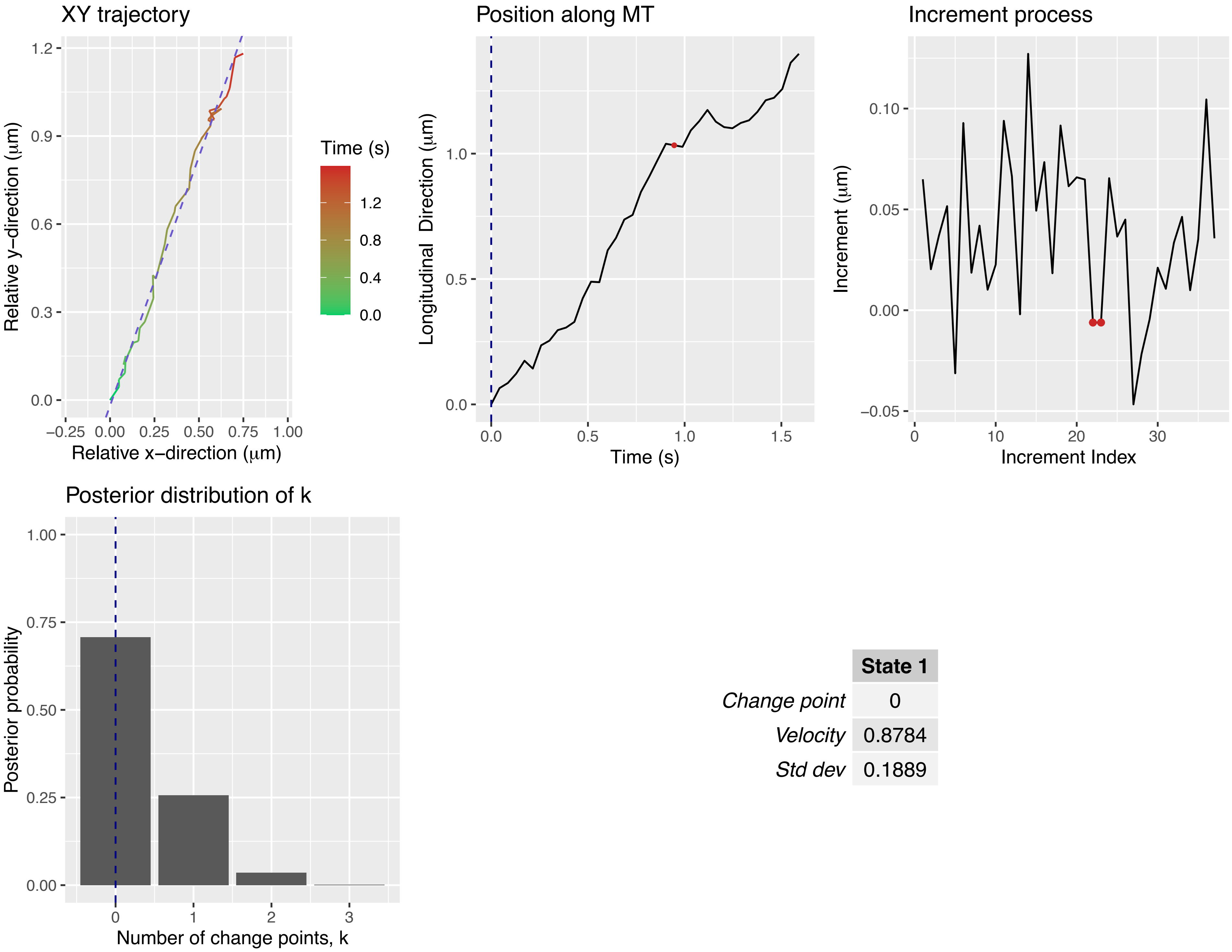

	State 1
Change point	0
Velocity	0.8784
Std dev	0.1889

DDB particle: 36

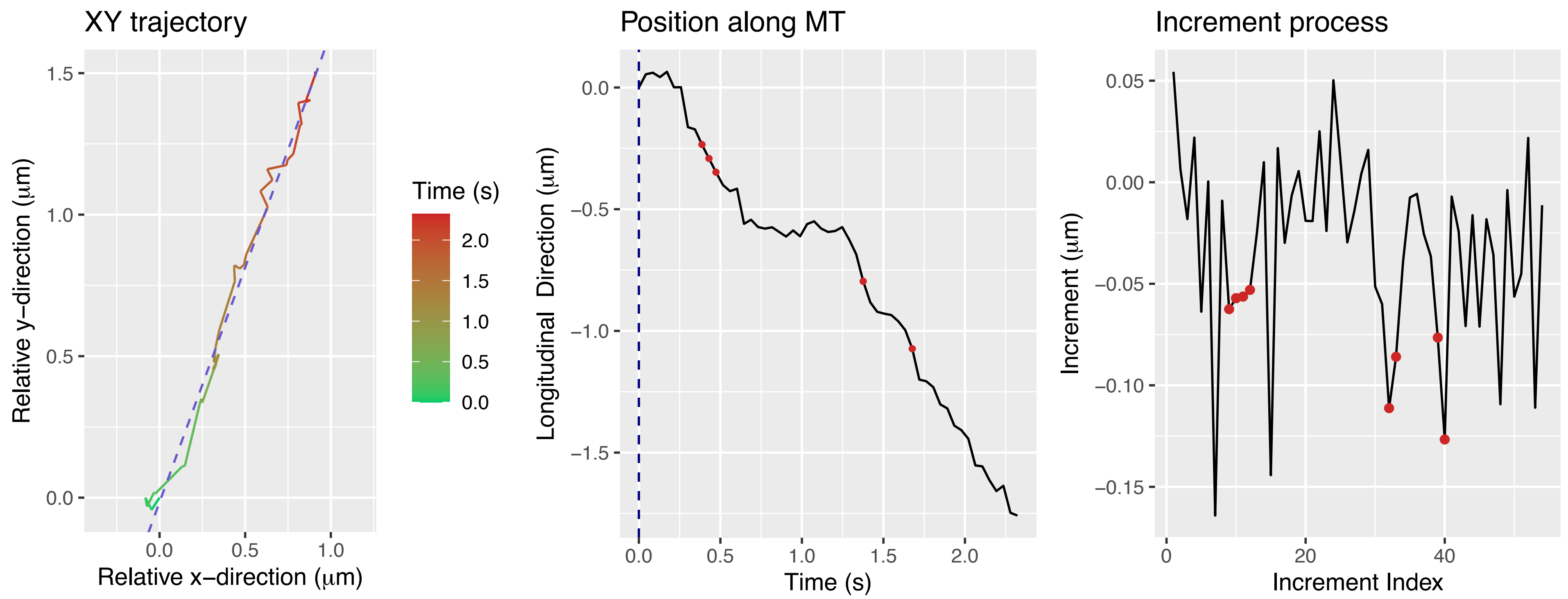

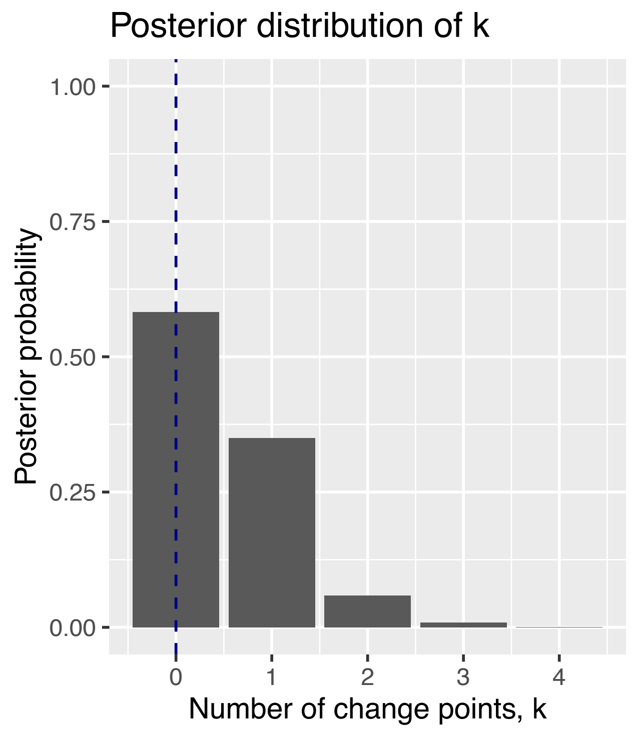

	State 1
Change point	0
Velocity	−0.7534
Std dev	0.2247

DDB particle: 37

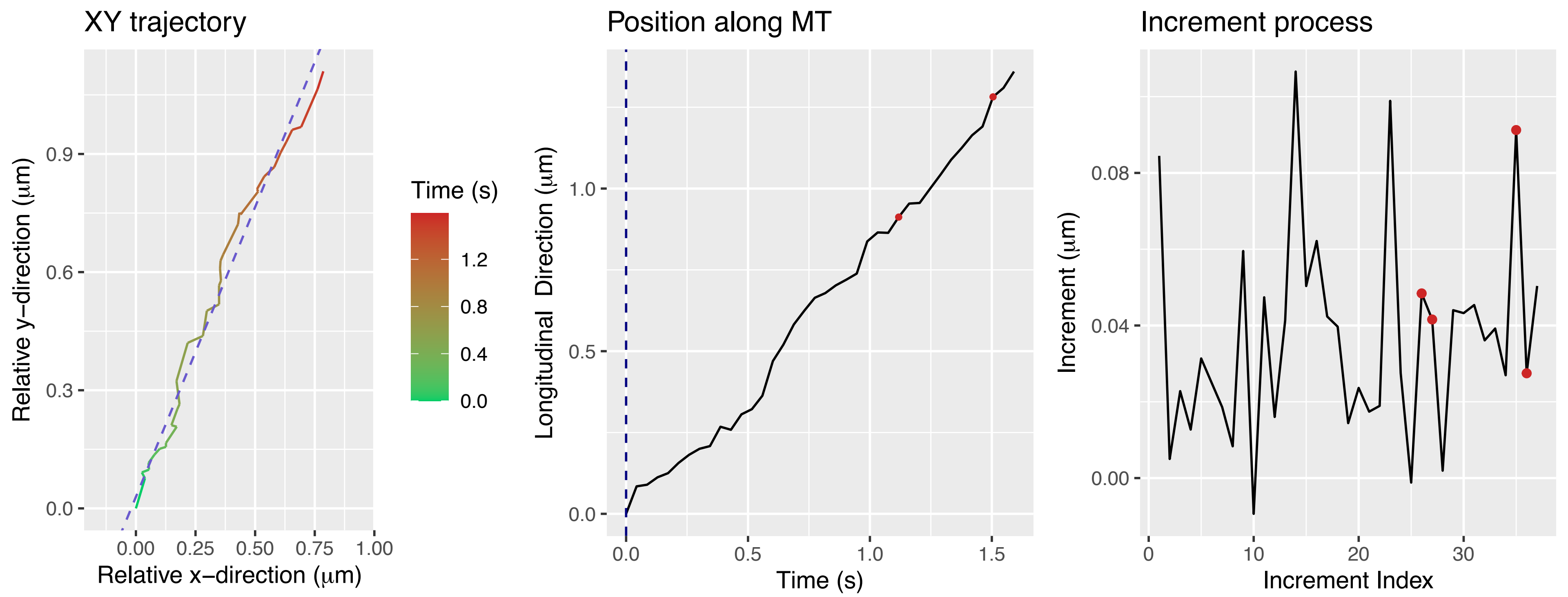

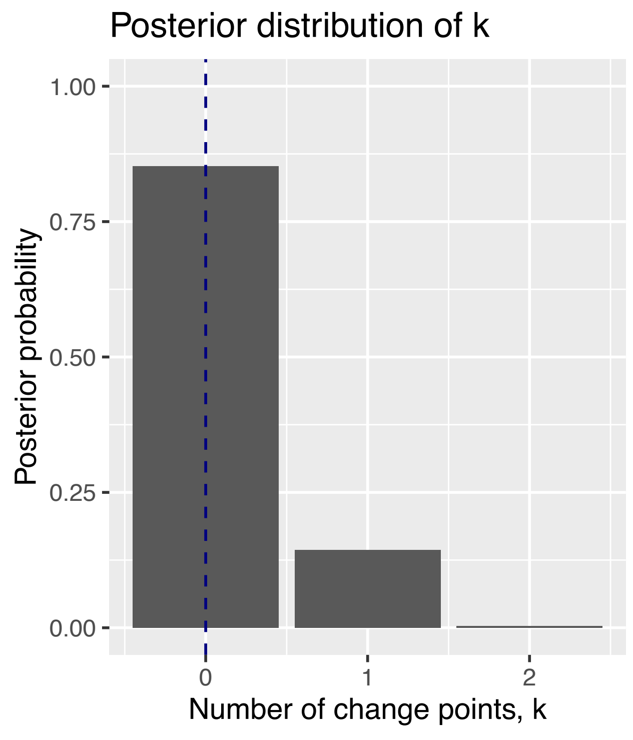

	State 1
Change point	0
Velocity	0.8537
Std dev	0.1294

DDB particle: 38

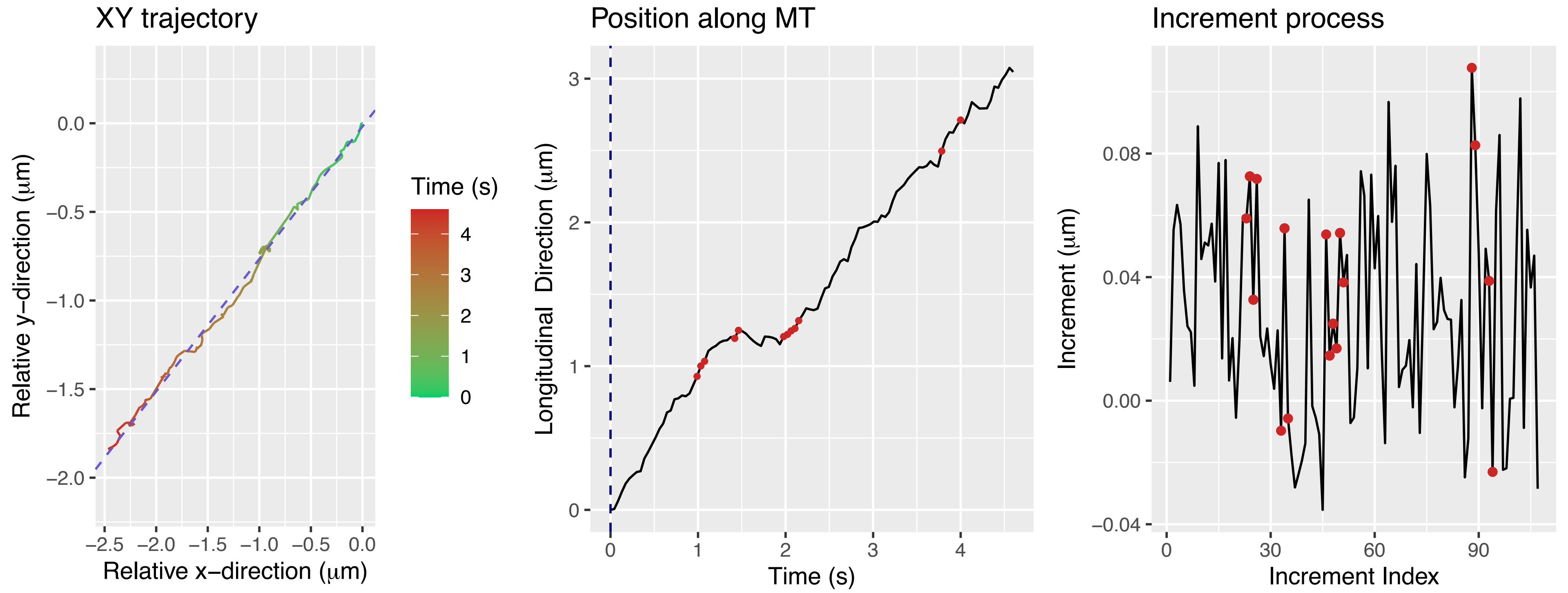

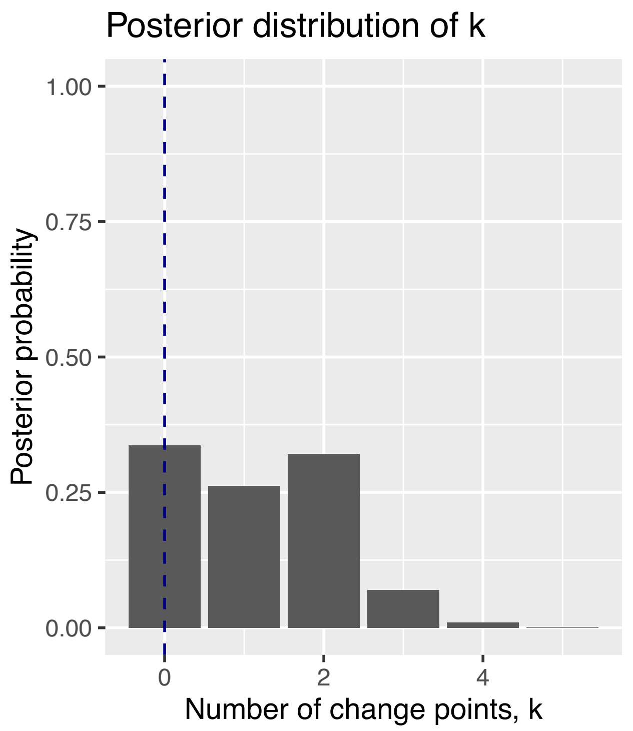

	**State 1**
Change point	0
Velocity	0.6625
Std dev	0.1622

DDB particle: 39

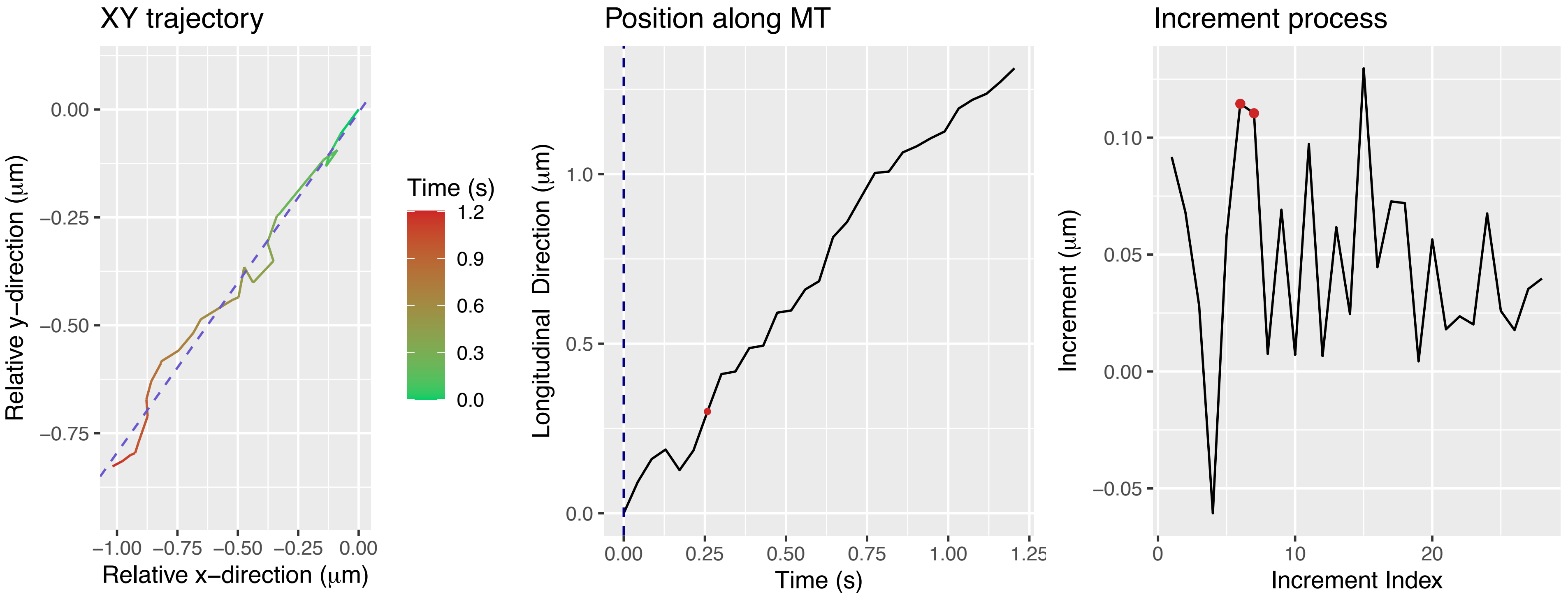

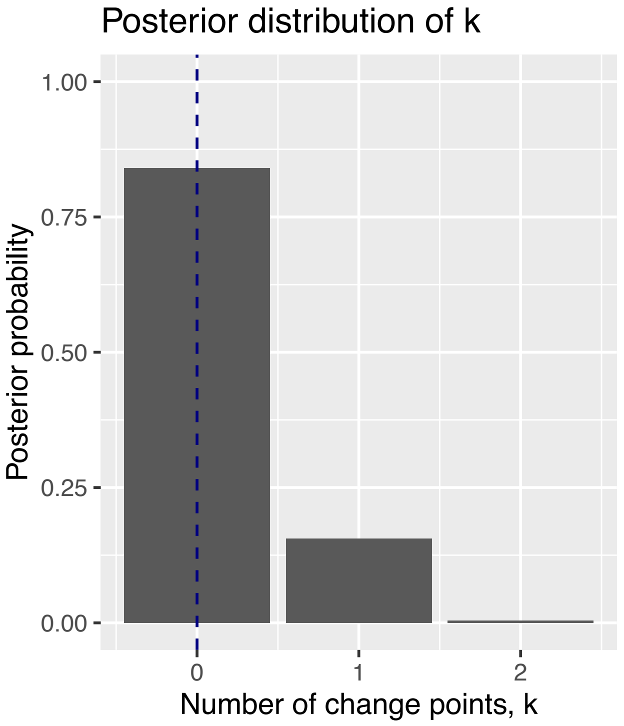

	State 1
Change point	0
Velocity	1.0891
Std dev	0.2031

DDB particle: 40

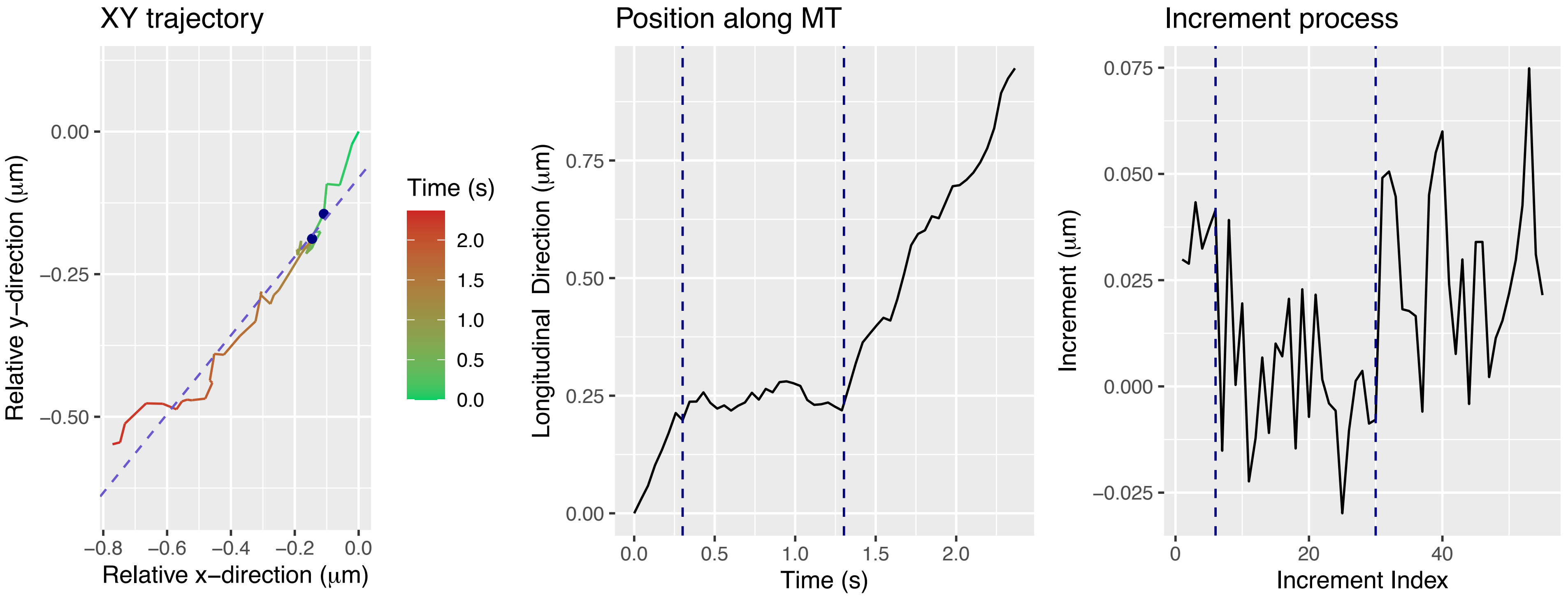

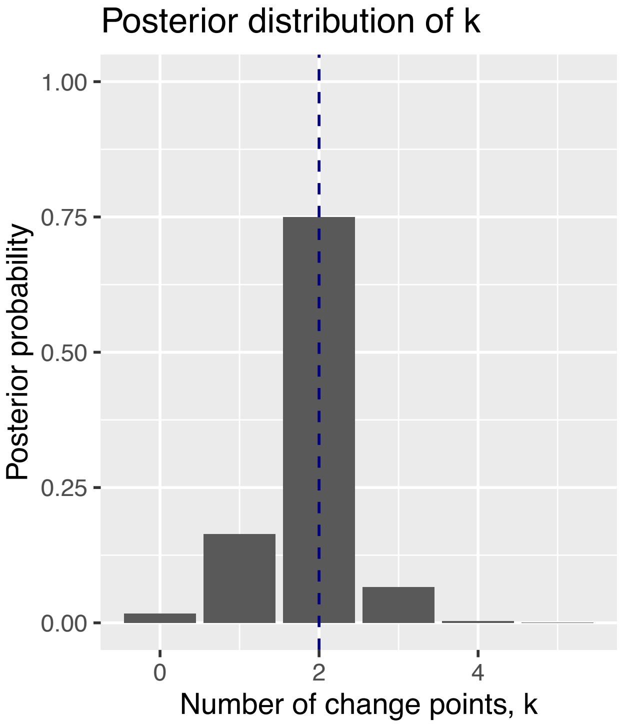

	State 1	State 2	State 3
Change point	0.3007	1.3028	NA
Velocity	0.8024	−0.0086	0.6676
Std dev	0.0842	NA	NA

DDB particle: 41

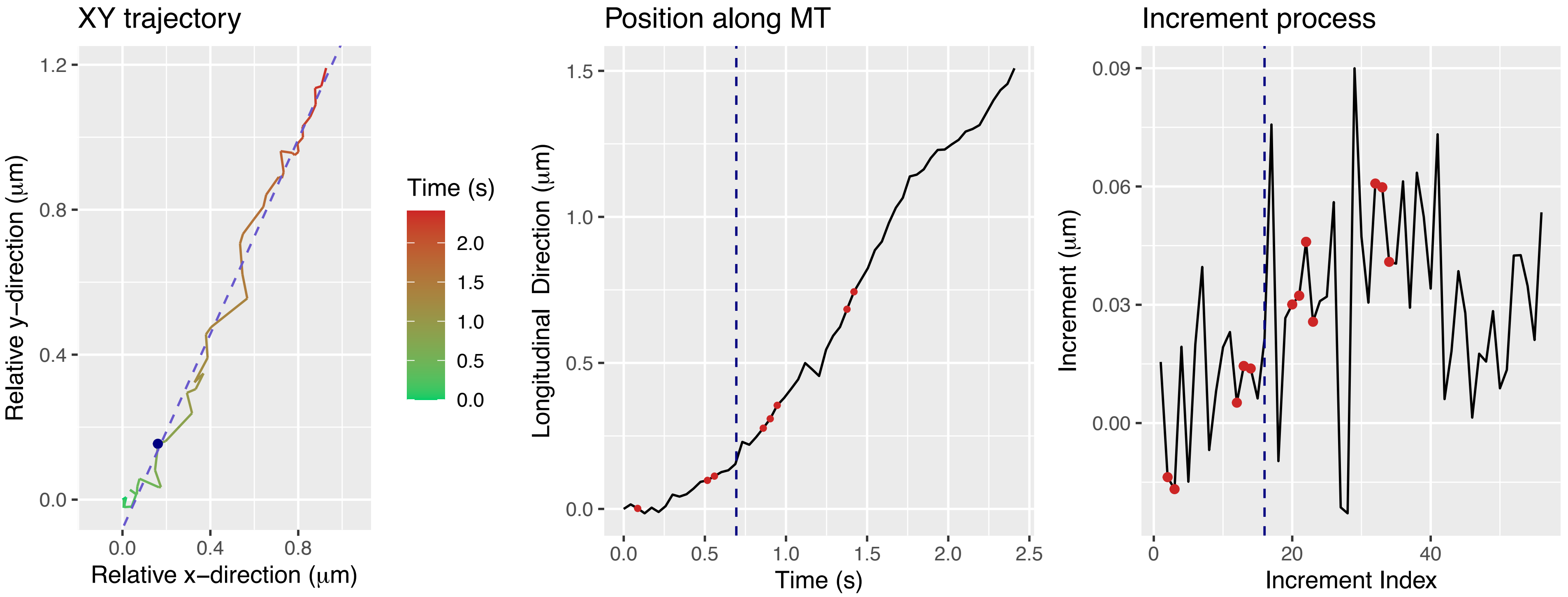

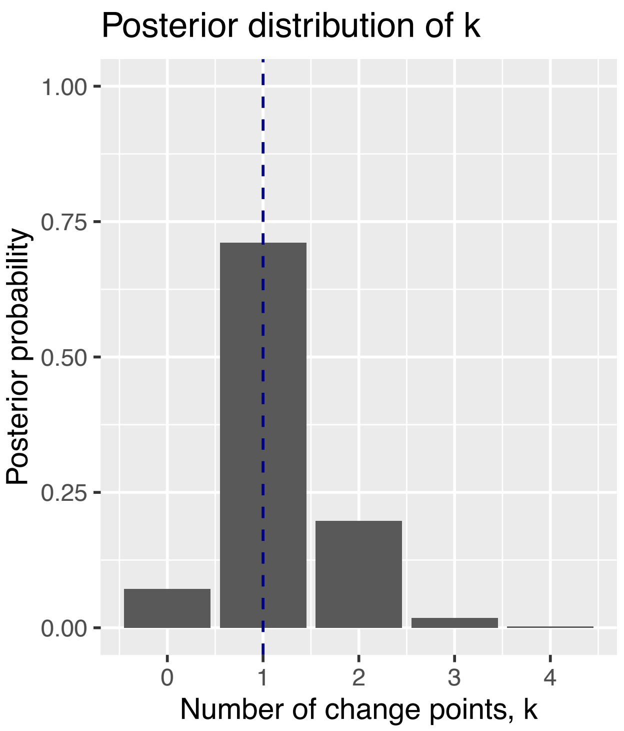

	State 1	State 2
Change point	0.6939	NA
Velocity	0.2478	0.7906
Std dev	0.1085	NA

DDB particle: 42

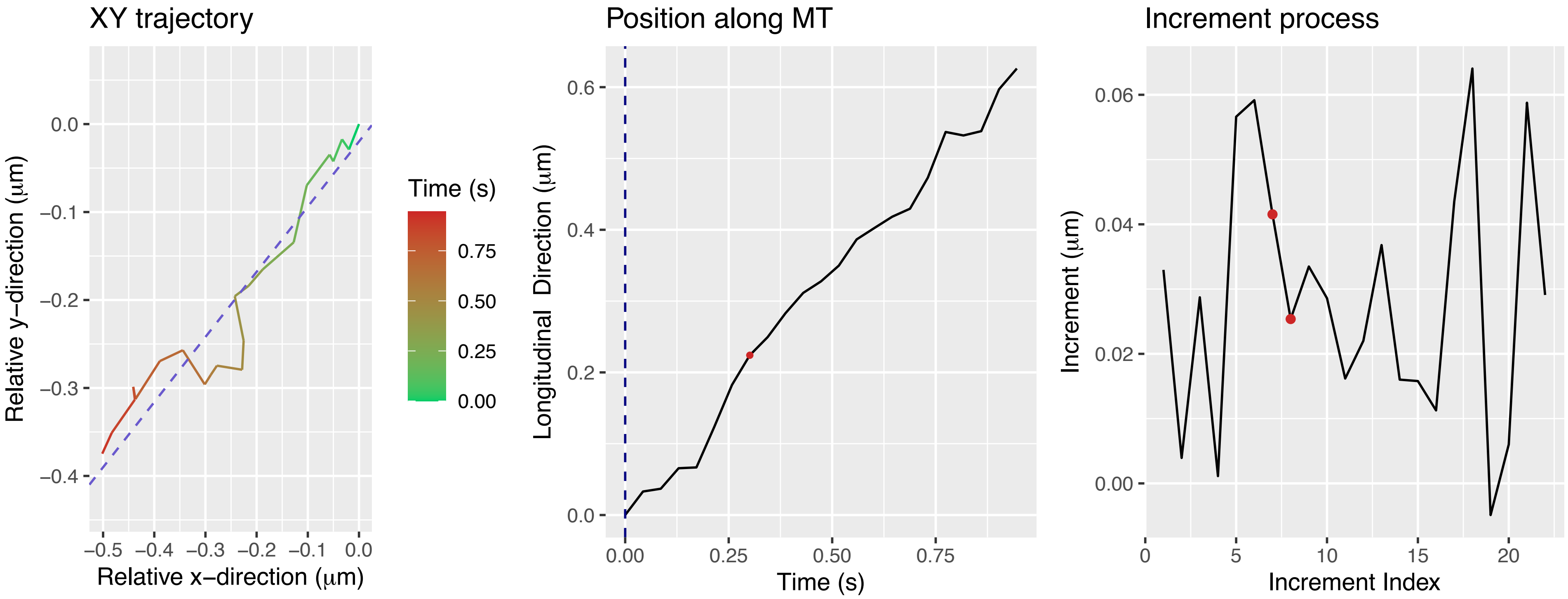

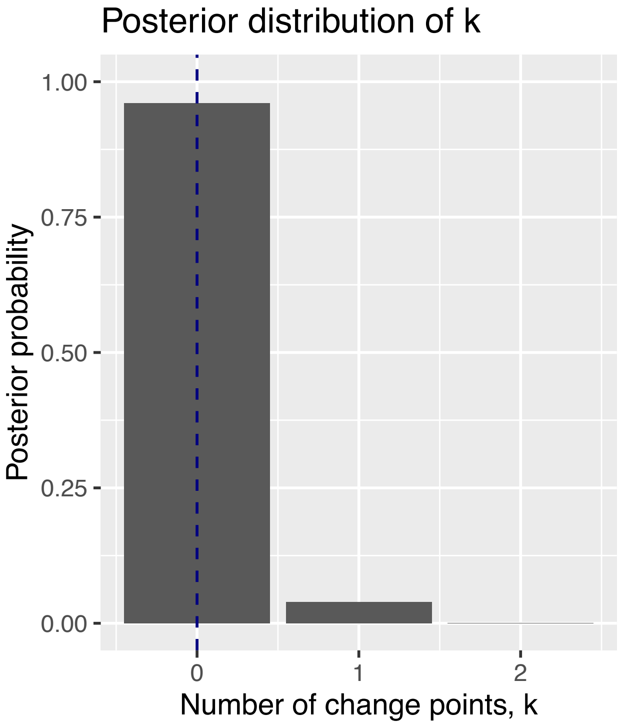

	State 1
Change point	0
Velocity	0.6585
Std dev	0.0918
