# Supplementary material for: A change point analysis protocol for comparing intracellular transport by different molecular motor combinations
Source: Math Biosci Eng. Author manuscript; Available in PMC 2023 Jan 6. (PMC9817212; doi:10.3934/mbe.2021442)
Supplement: supplemental material [file NIHMS1850682-supplement-supplemental_material.zip › mbe-18-06-442 supplementary/SI_Kin1.pdf]

Kin1 particle: 7

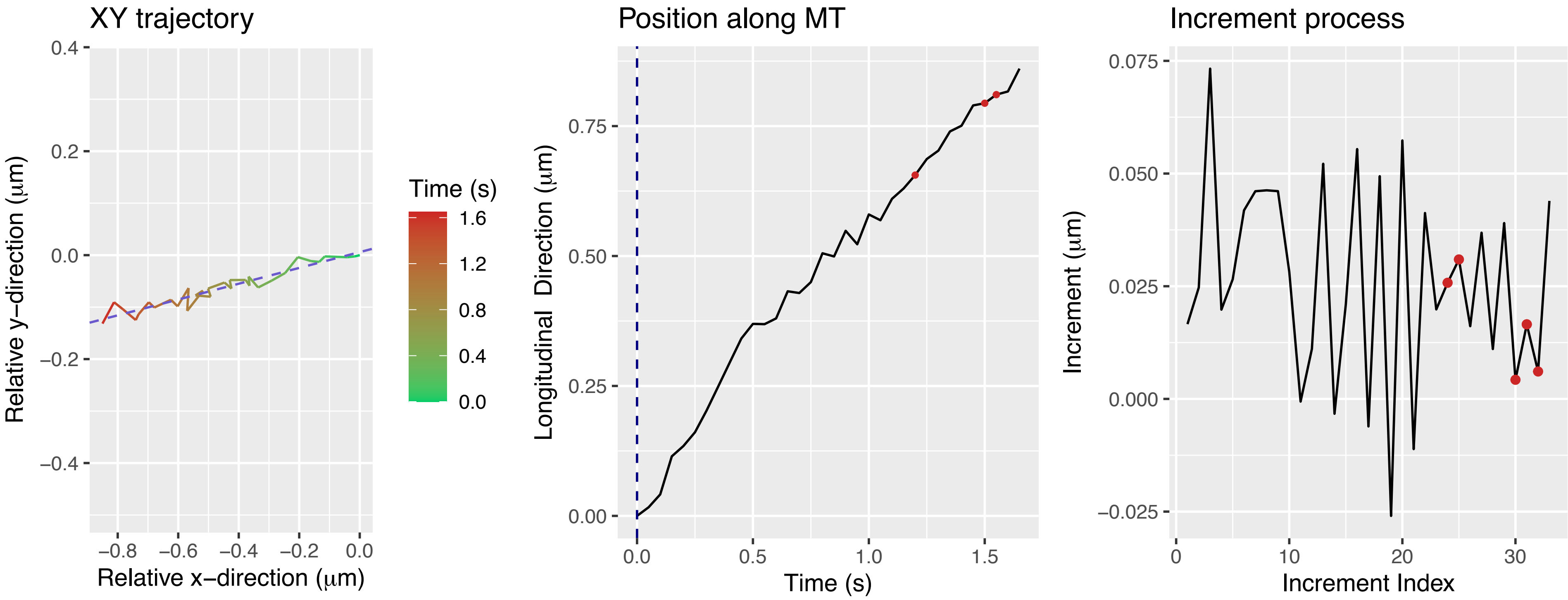

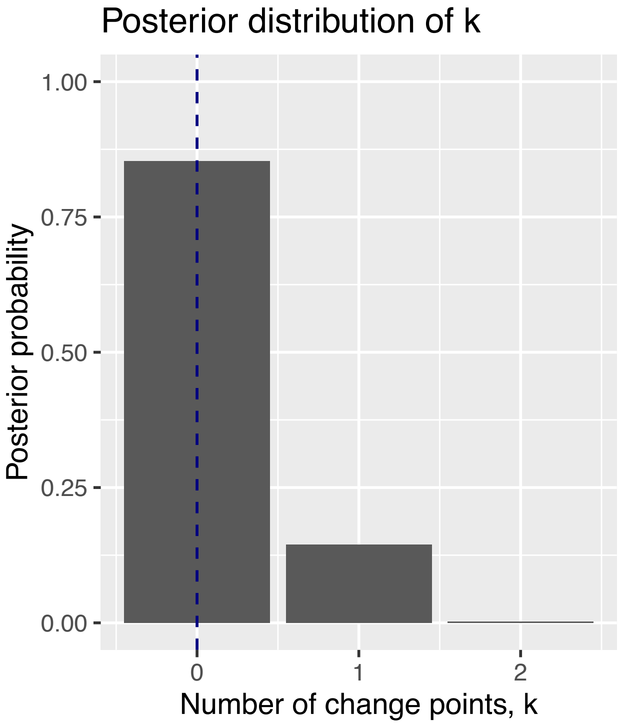

	**State 1**
Change point	0
Velocity	0.5224
Std dev	0.0982

Kin1 particle: 8

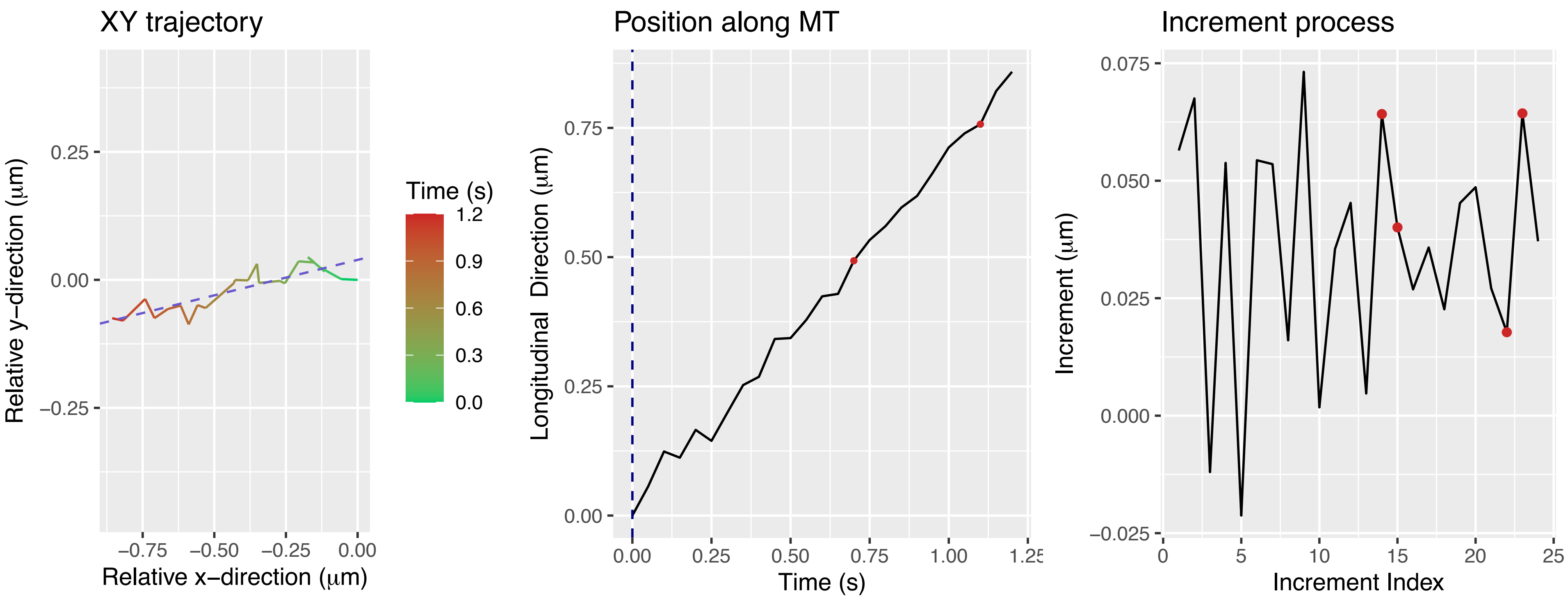

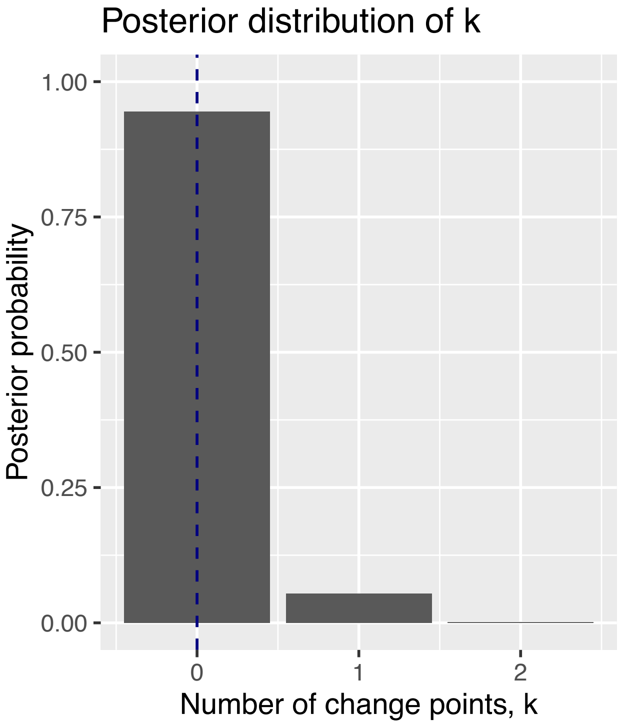

	State 1
Change point	0
Velocity	0.7173
Std dev	0.11

Kin1 particle: 9

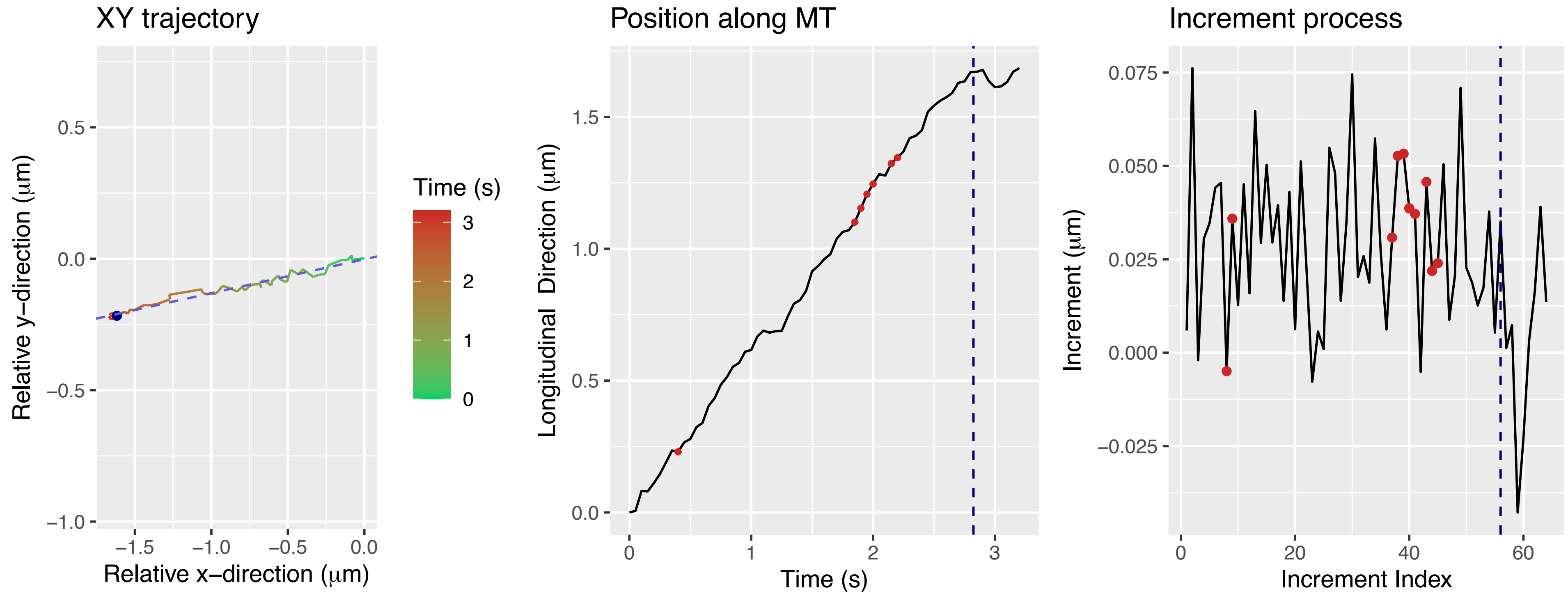

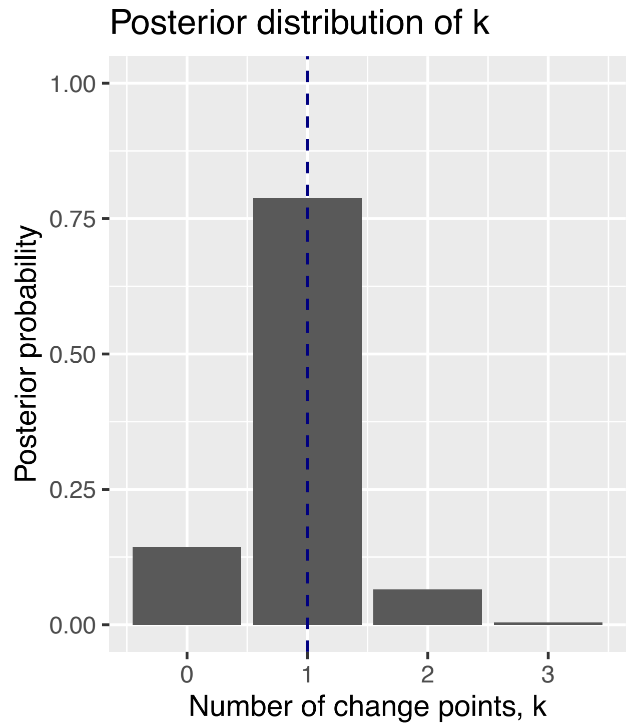

	State 1	State 2
Change point	2.8234	NA
Velocity	0.6036	0.0309
Std dev	0.0943	NA

Kin1 particle: 10

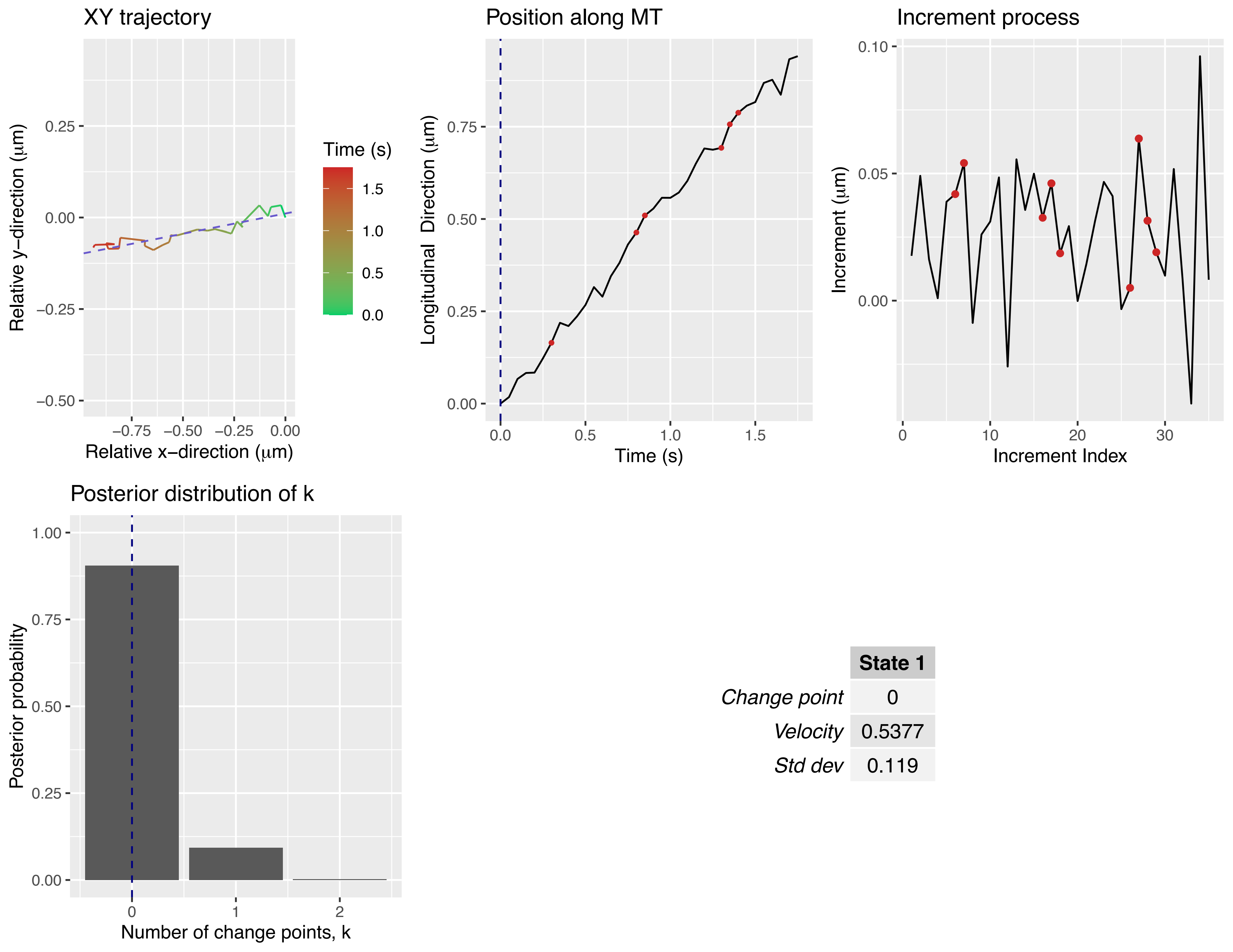

	State 1
Change point	0
Velocity	0.5377
Std dev	0.119

Kin1 particle: 11

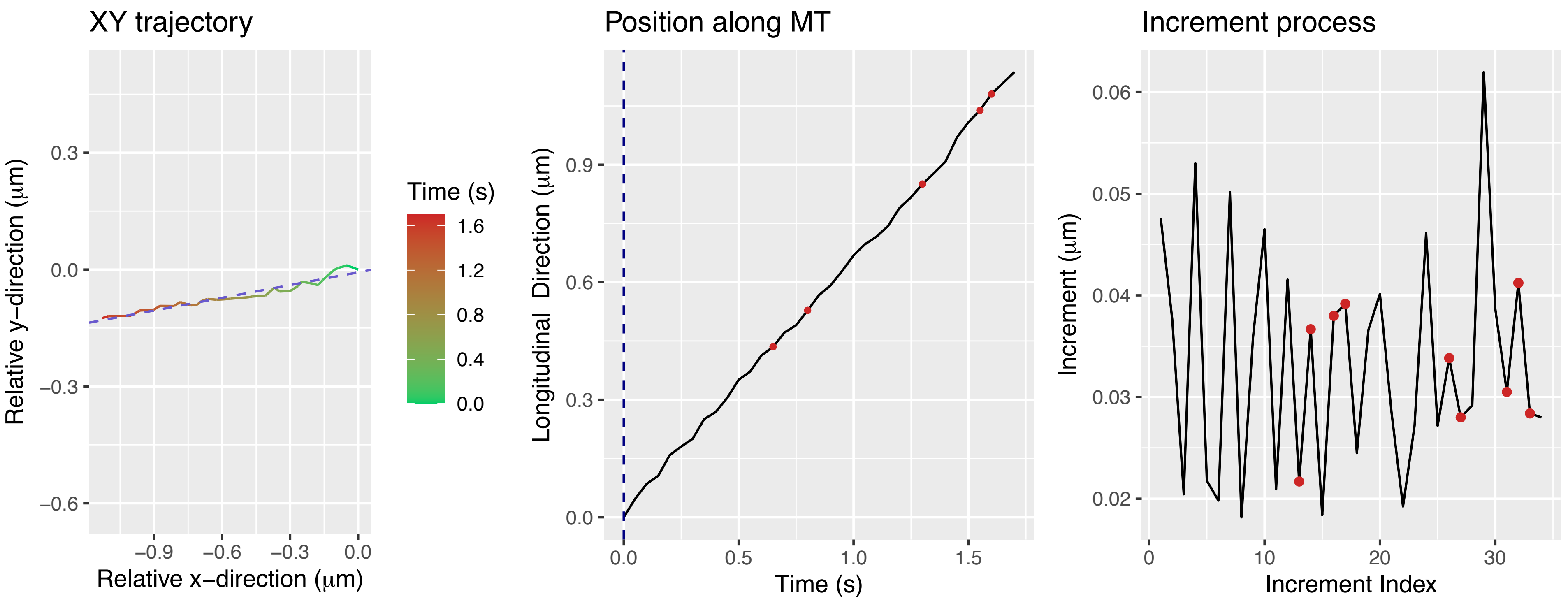

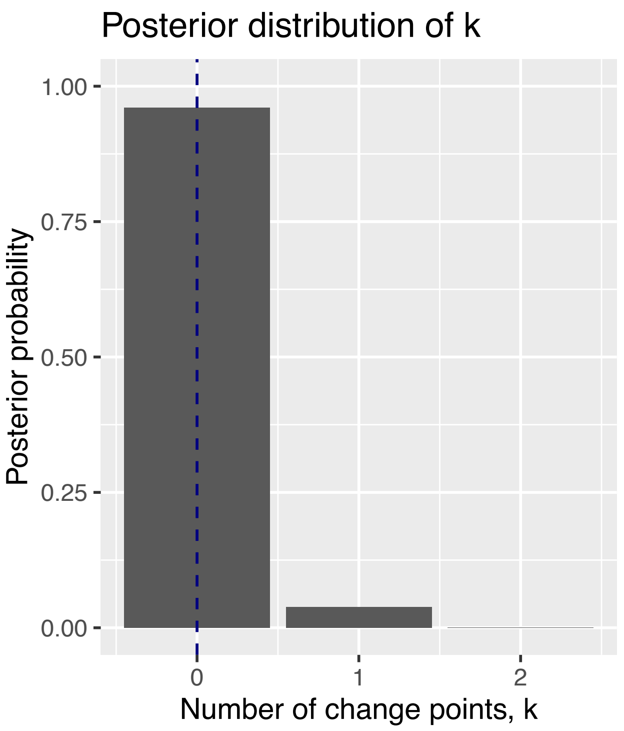

	State 1
Change point	0
Velocity	0.6679
Std dev	0.0446

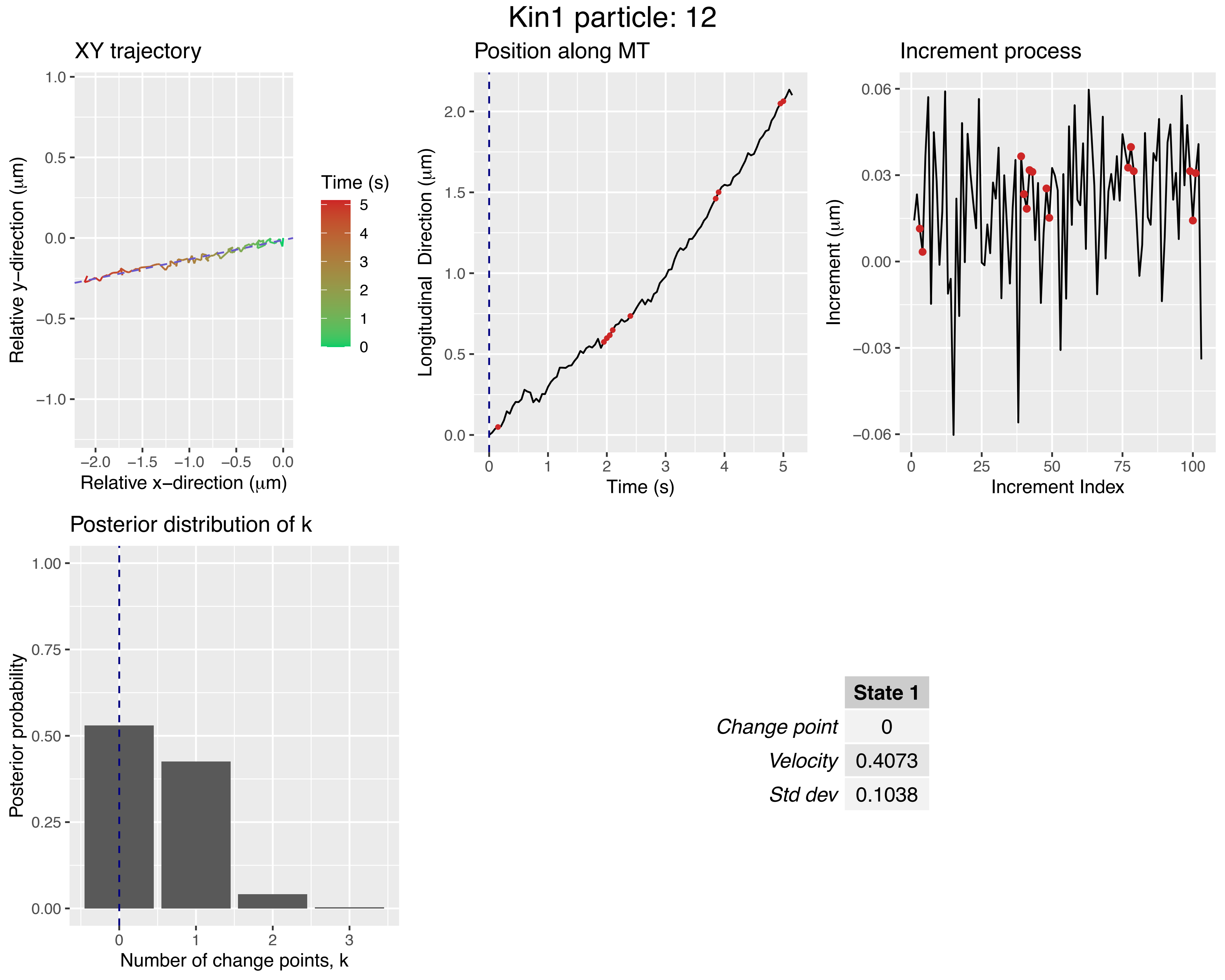

	State 1
Change point	0
Velocity	0.4073
Std dev	0.1038

Kin1 particle: 13

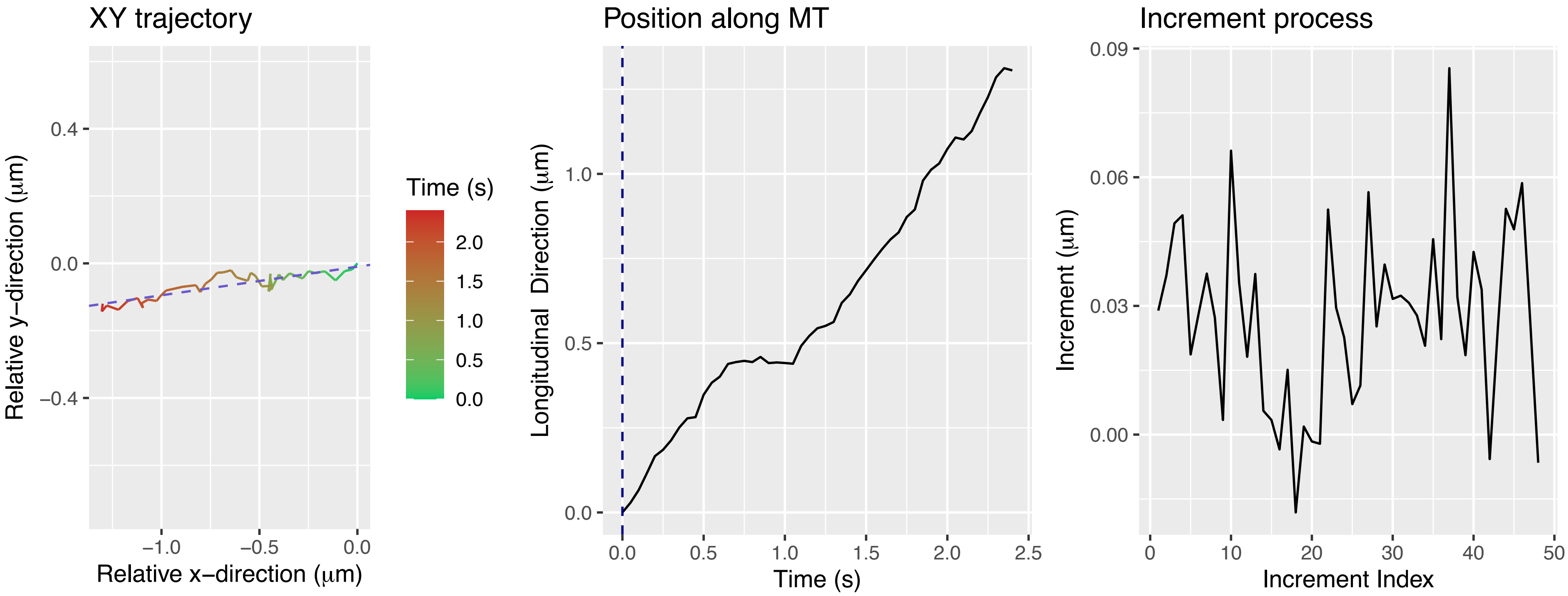

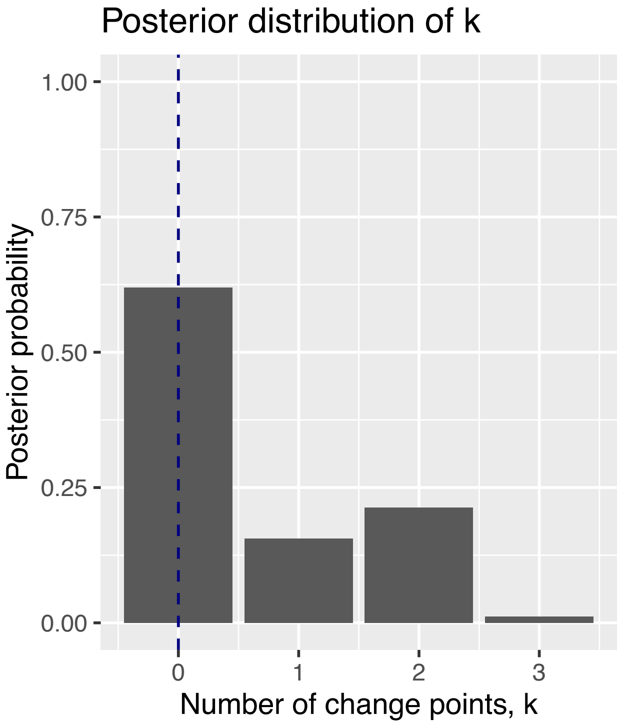

	State 1
Change point	0
Velocity	0.5443
Std dev	0.0929

Kin1 particle: 14

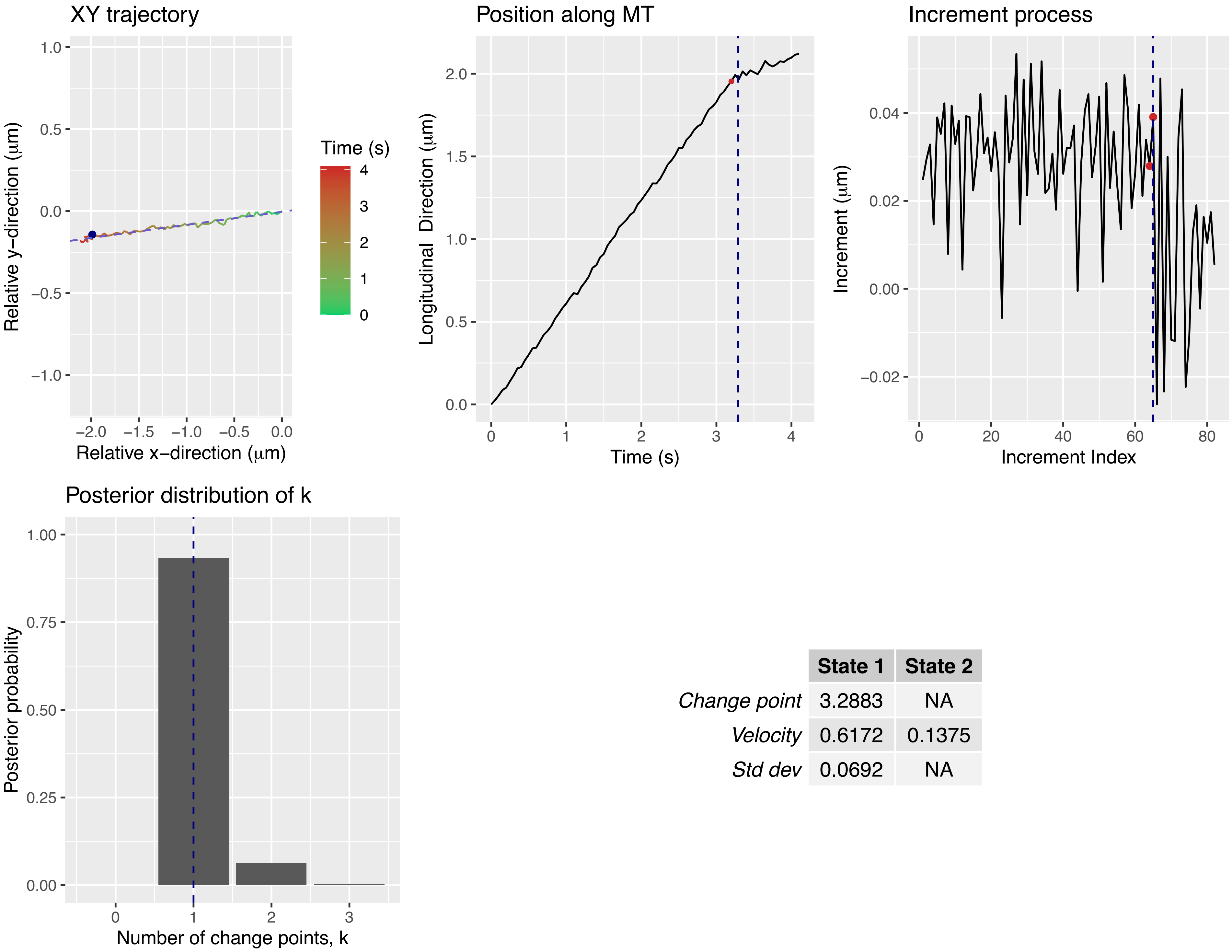

	State 1	State 2
Change point	3.2883	NA
Velocity	0.6172	0.1375
Std dev	0.0692	NA

Kin1 particle: 15

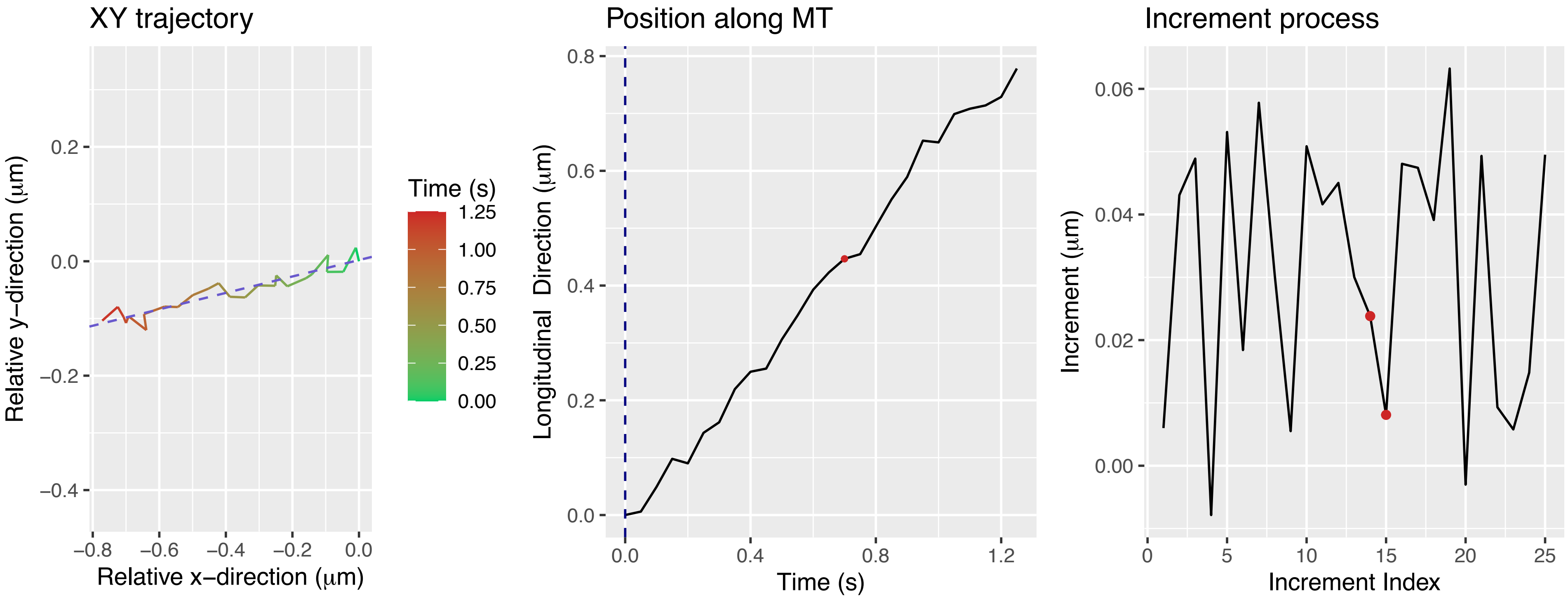

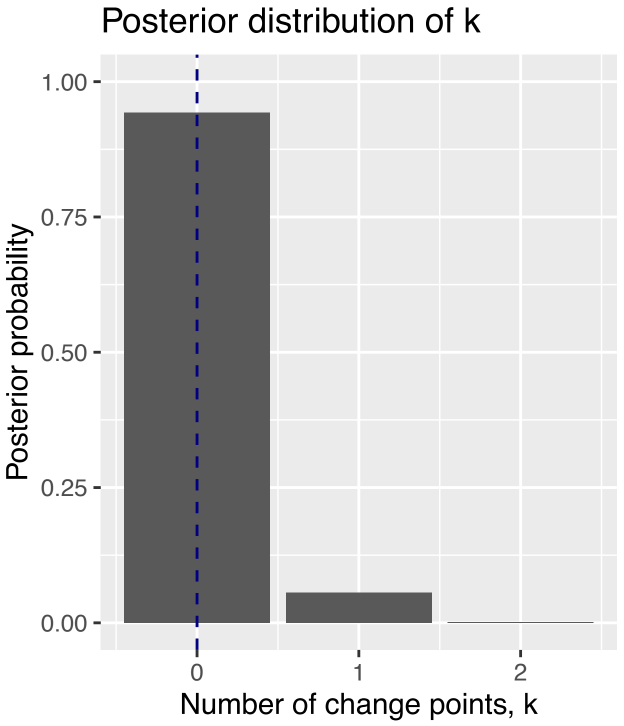

	State 1
Change point	0
Velocity	0.6217
Std dev	0.091

Kin1 particle: 16

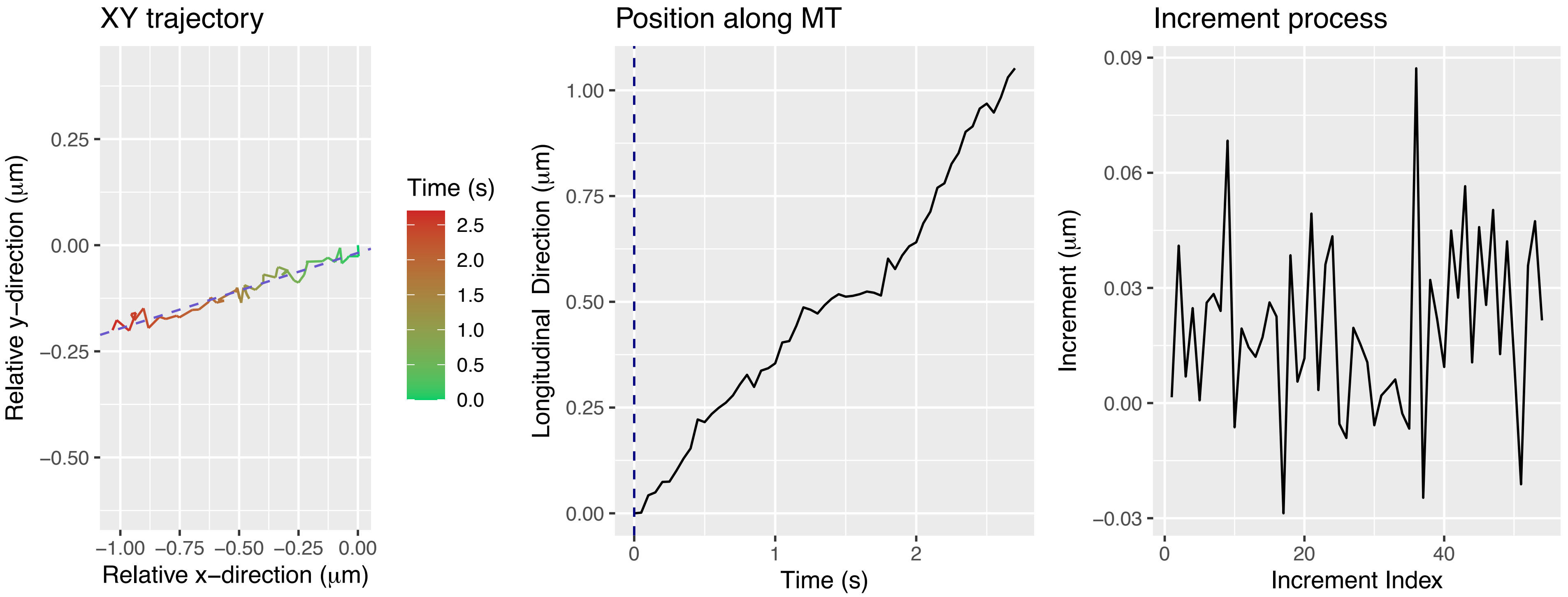

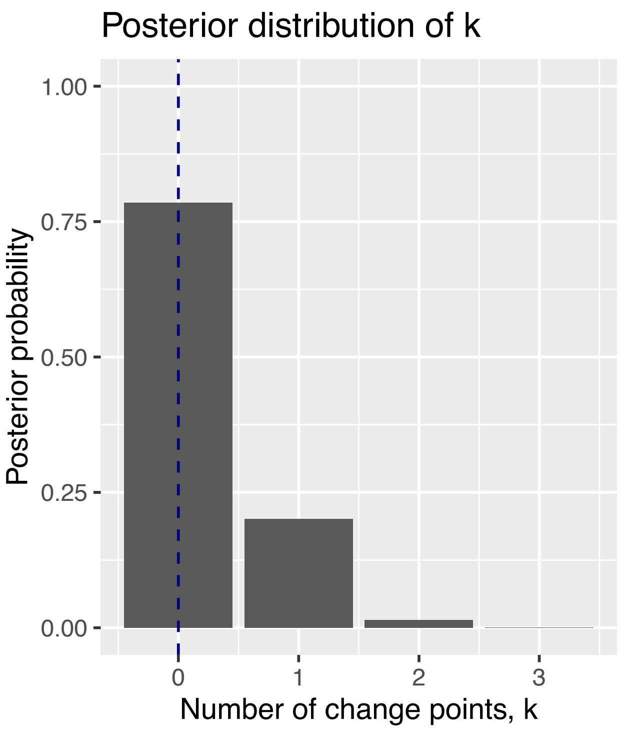

	State 1
Change point	0
Velocity	0.385
Std dev	0.1011

Kin1 particle: 17

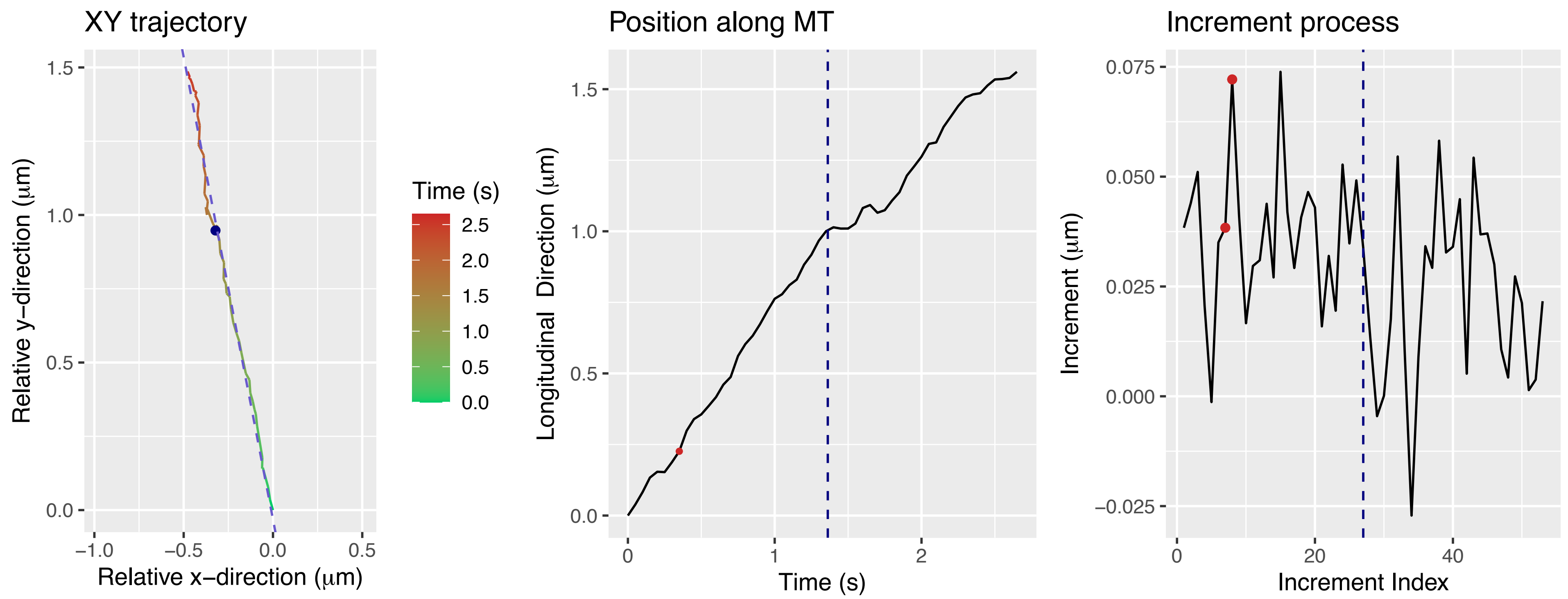

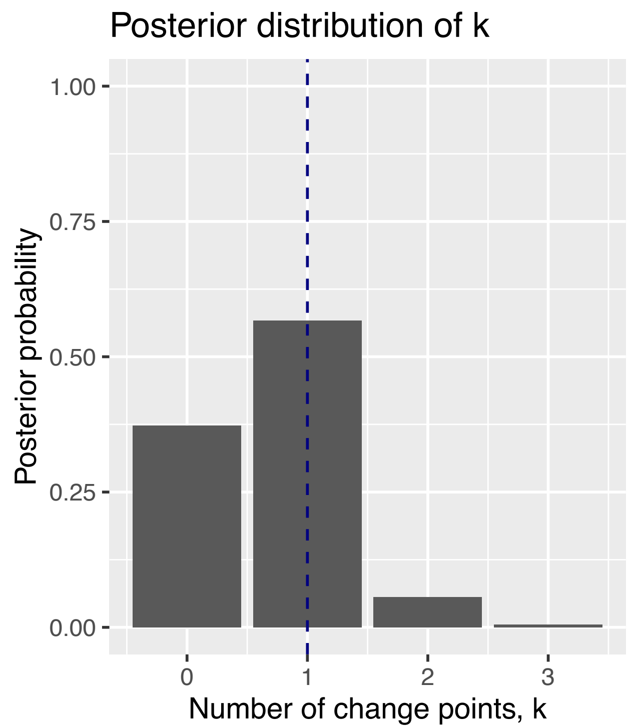

	State 1	State 2
Change point	1.3616	NA
Velocity	0.7393	0.4335
Std dev	0.0815	NA

Kin1 particle: 18

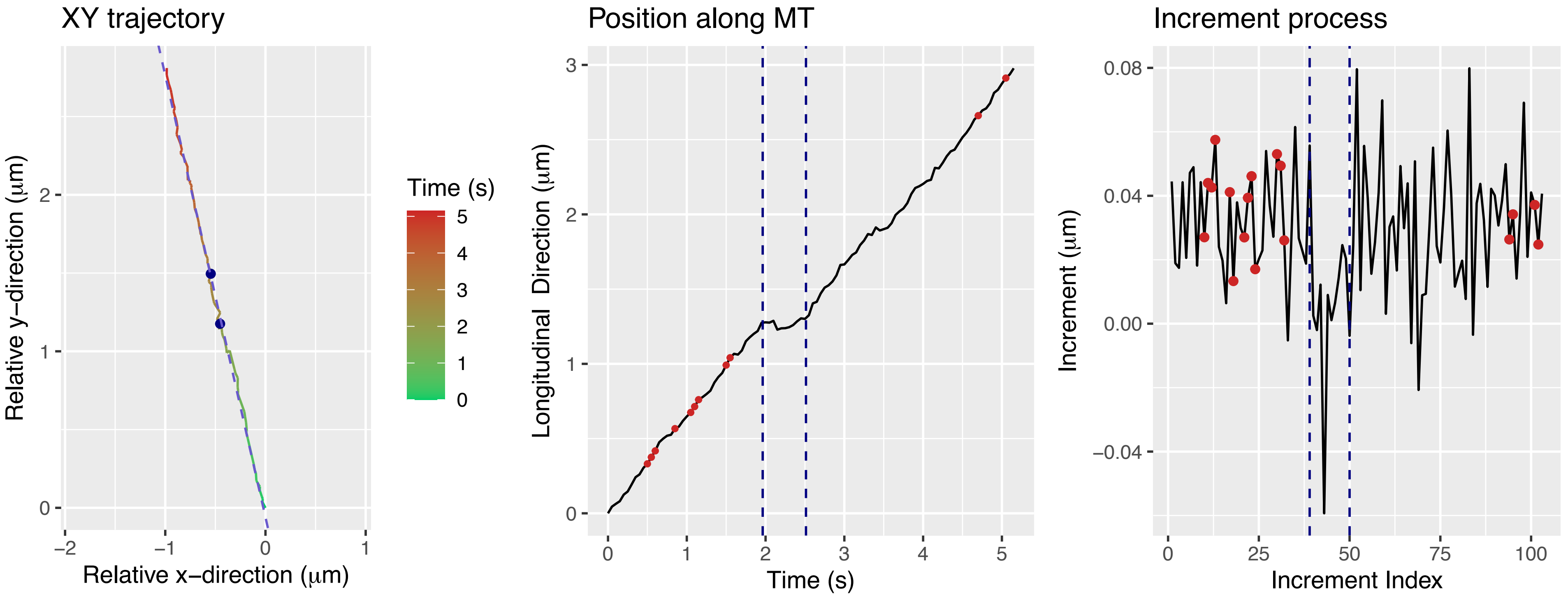

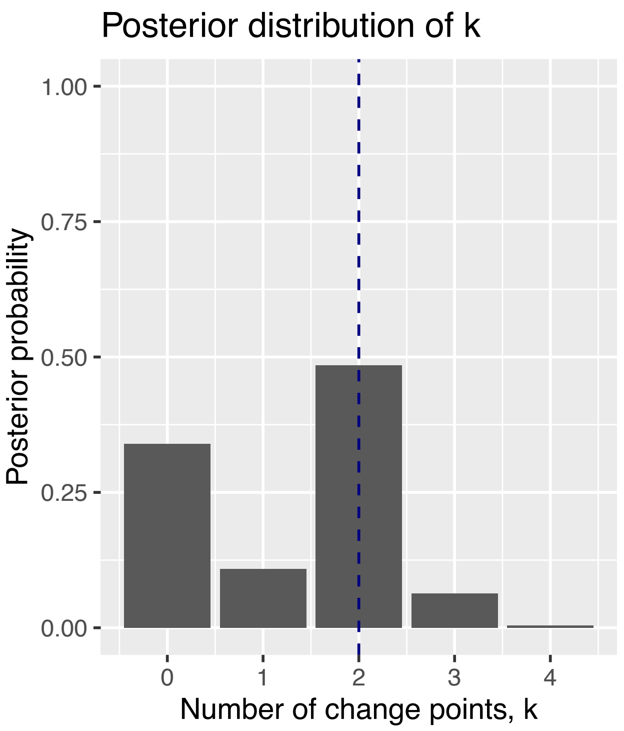

	State 1	State 2	State 3
Change point	1.9636	2.513	NA
Velocity	0.6408	0.083	0.6216
Std dev	0.0861	NA	NA

Kin1 particle: 19

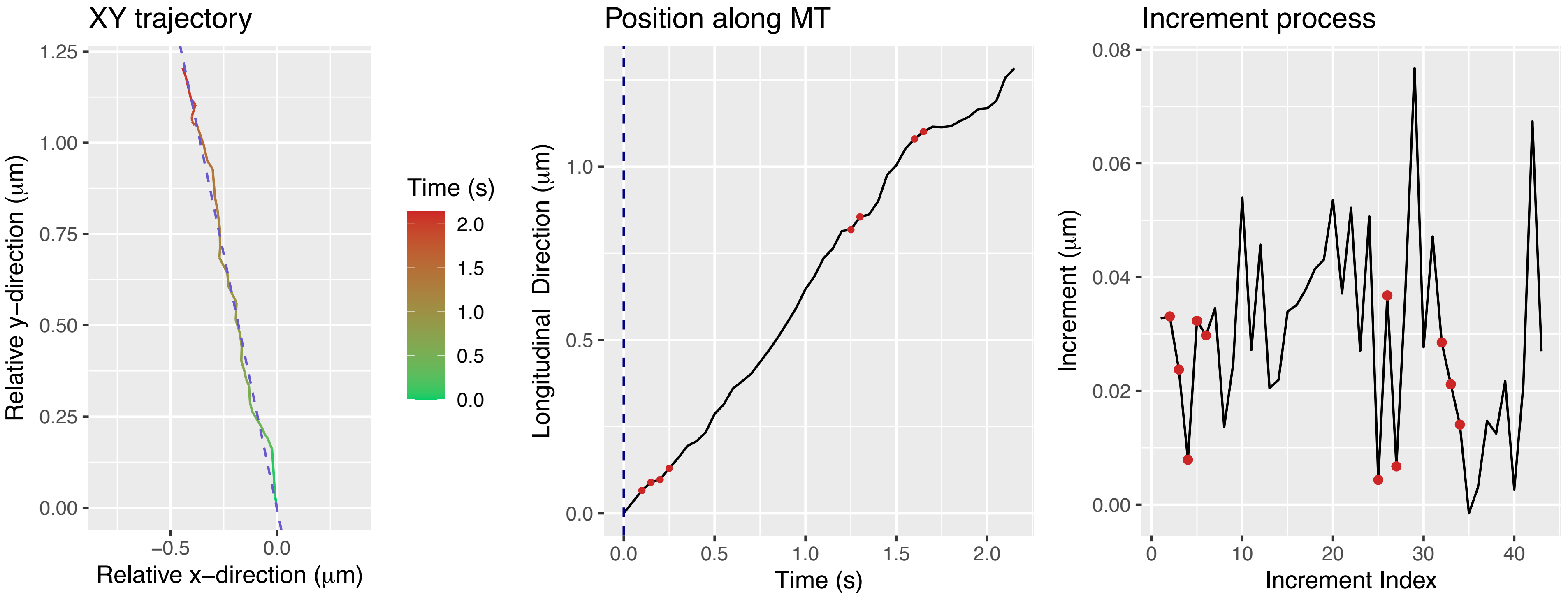

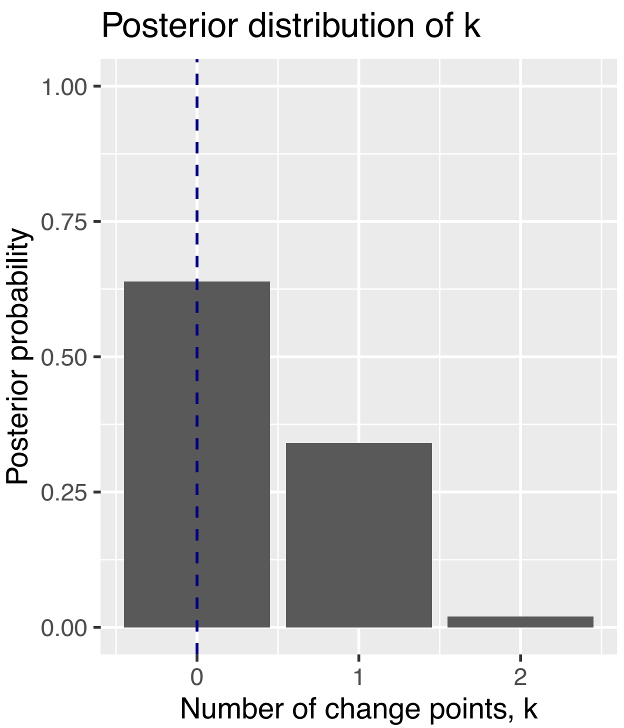

	State 1
Change point	0
Velocity	0.5975
Std dev	0.0745

Kin1 particle: 20

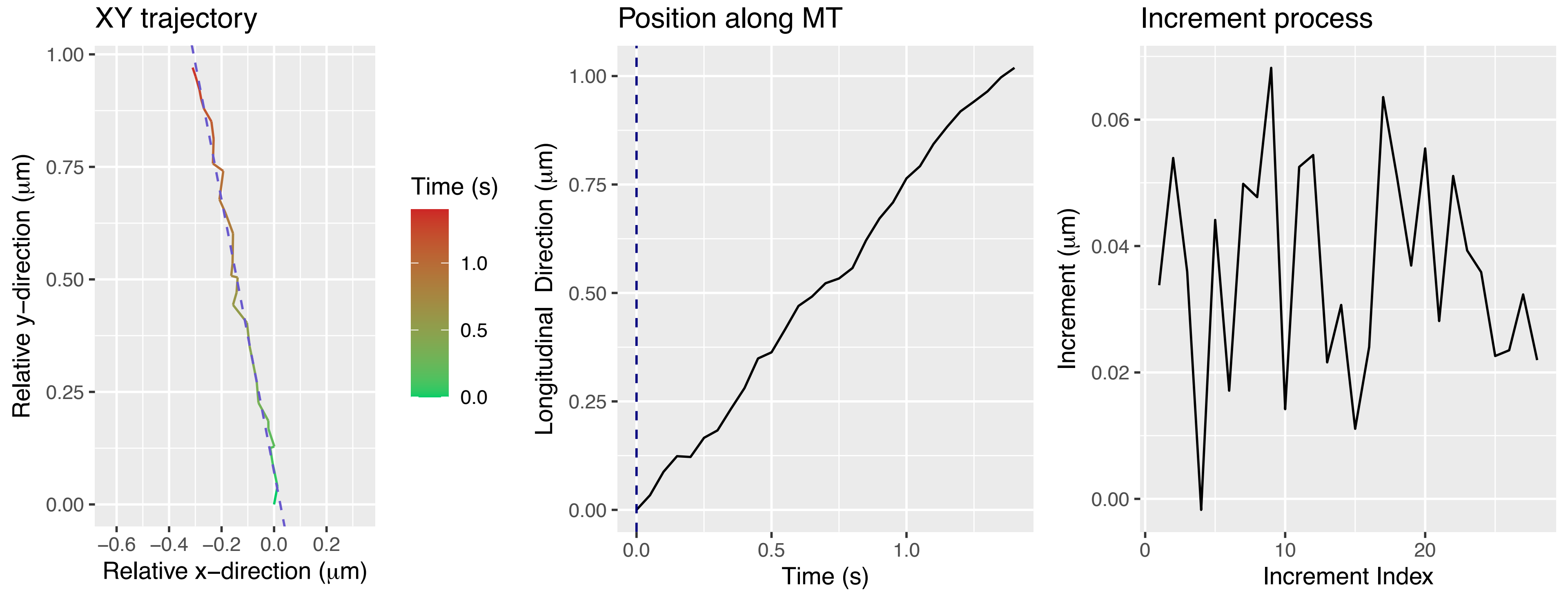

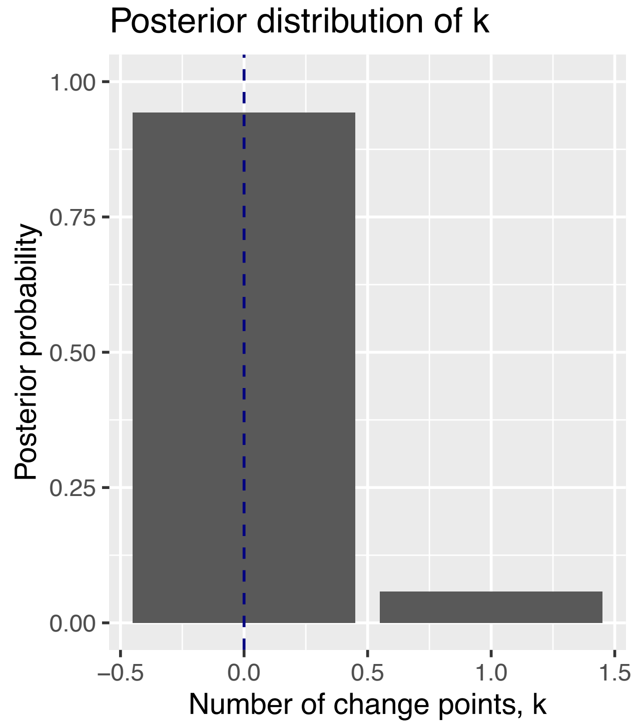

	State 1
Change point	0
Velocity	0.7289
Std dev	0.0717

Kin1 particle: 21

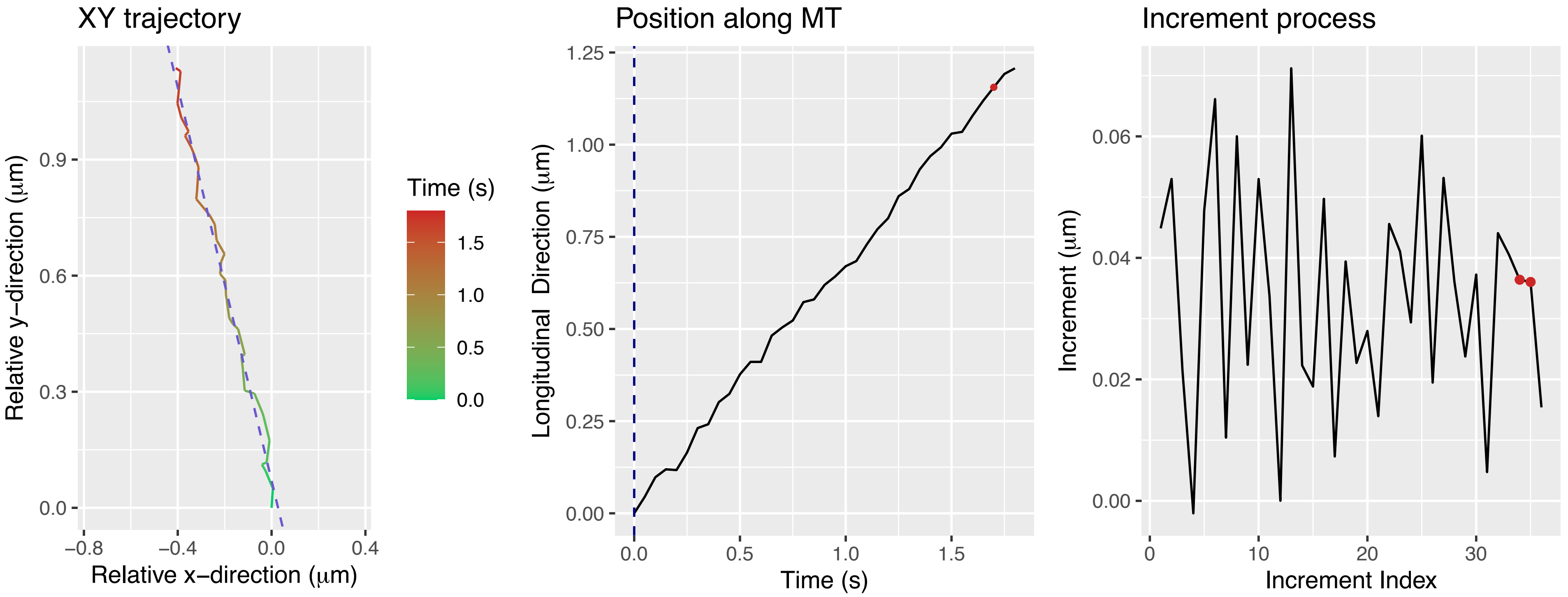

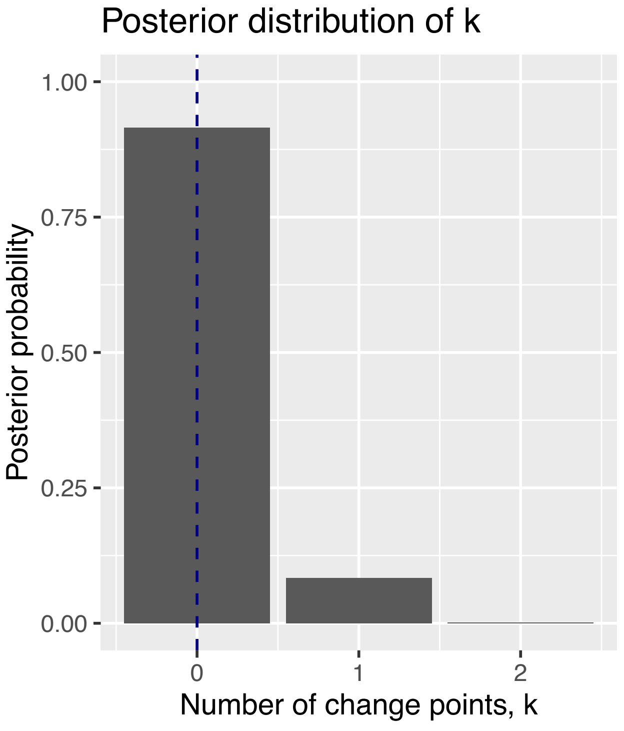

	State 1
Change point	0
Velocity	0.6731
Std dev	0.0809

Kin1 particle: 22

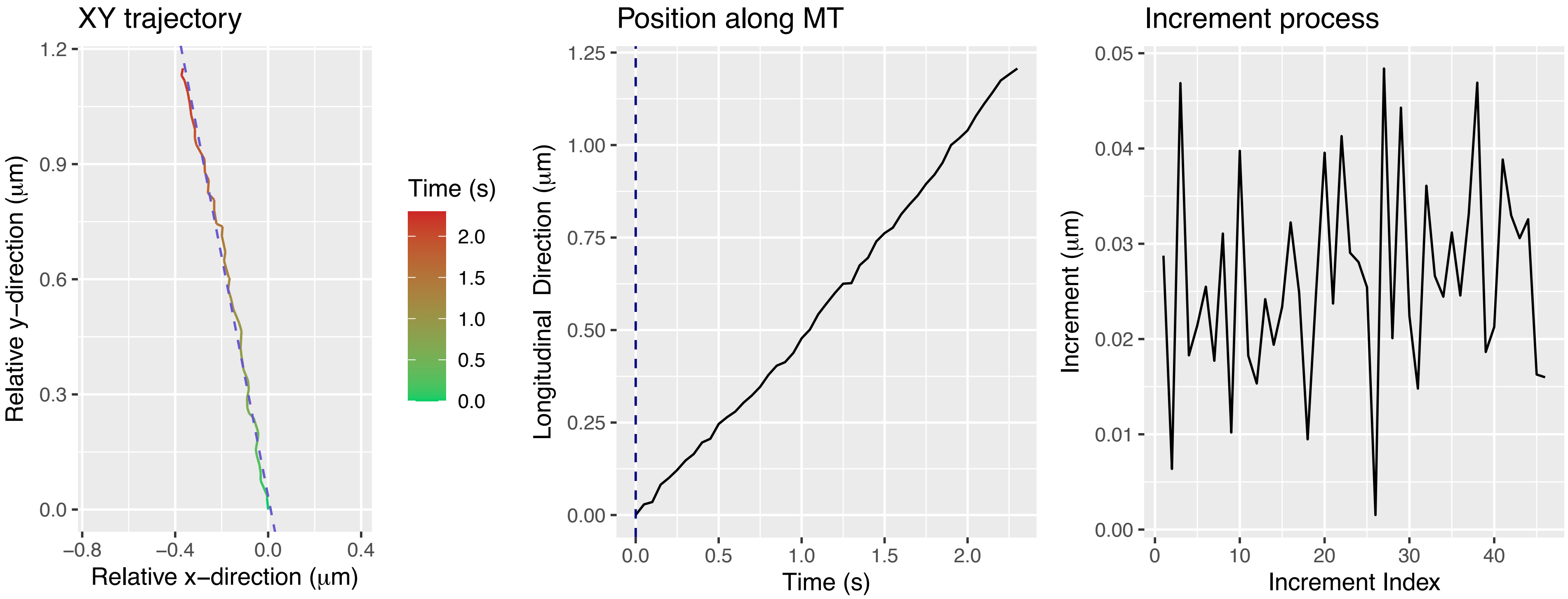

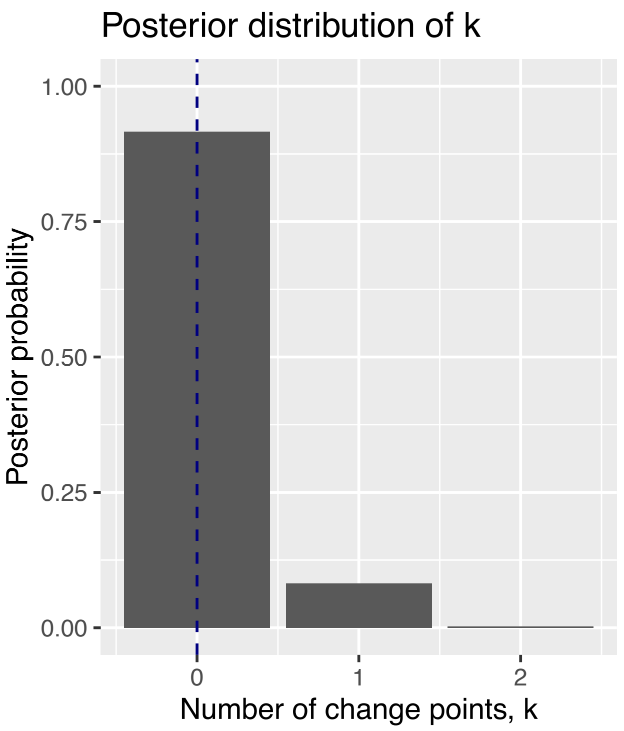

	State 1
Change point	0
Velocity	0.5253
Std dev	0.0435

Kin1 particle: 23

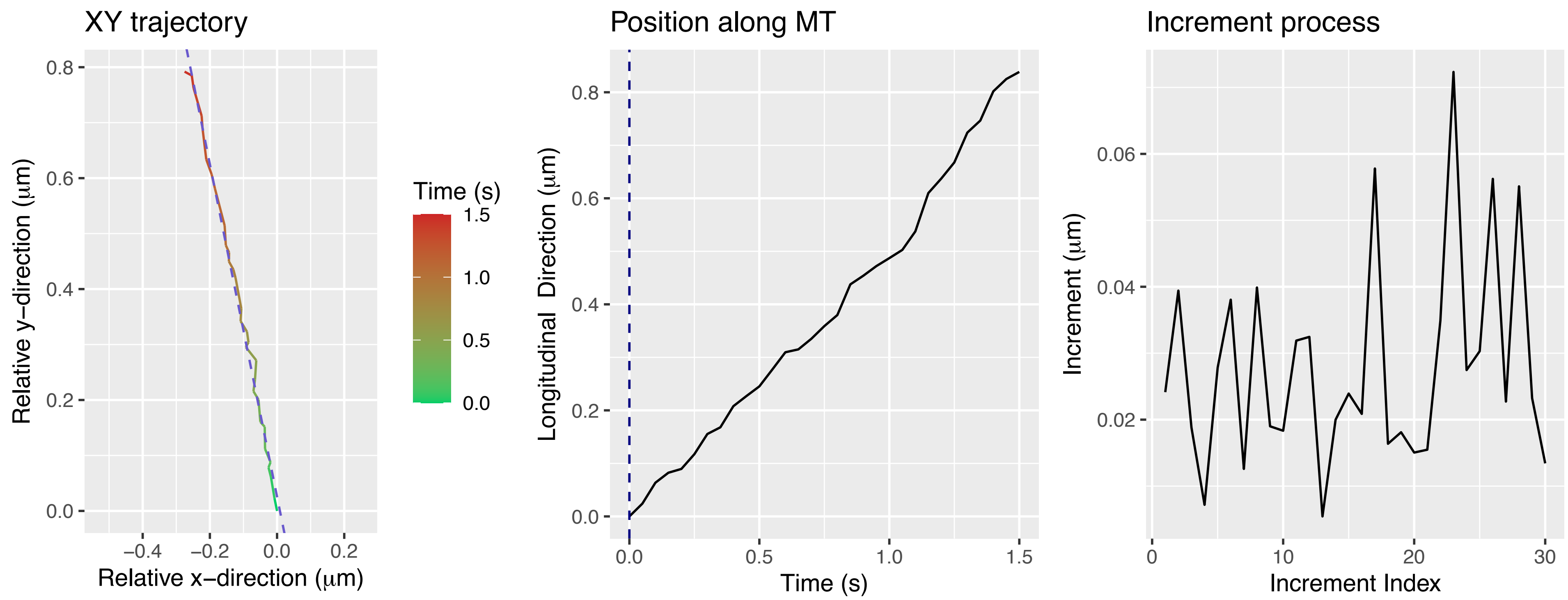

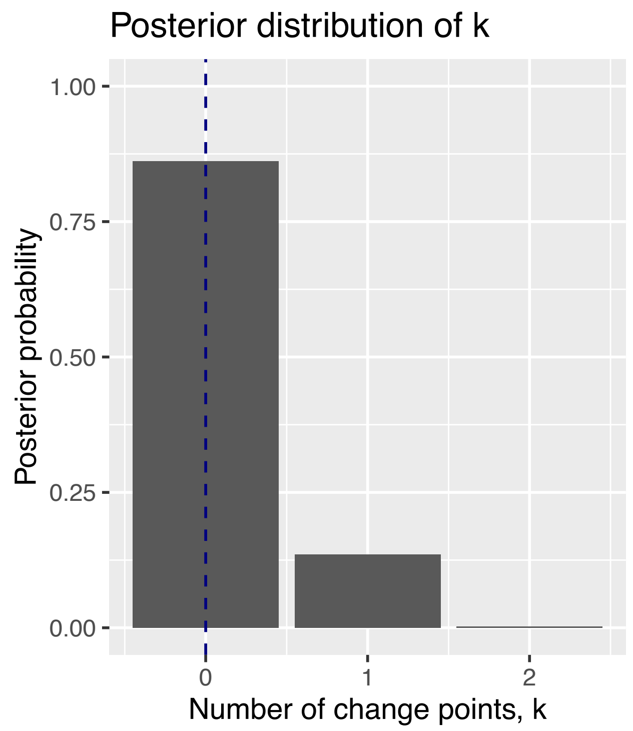

	State 1
Change point	0
Velocity	0.5568
Std dev	0.0665

Kin1 particle: 24

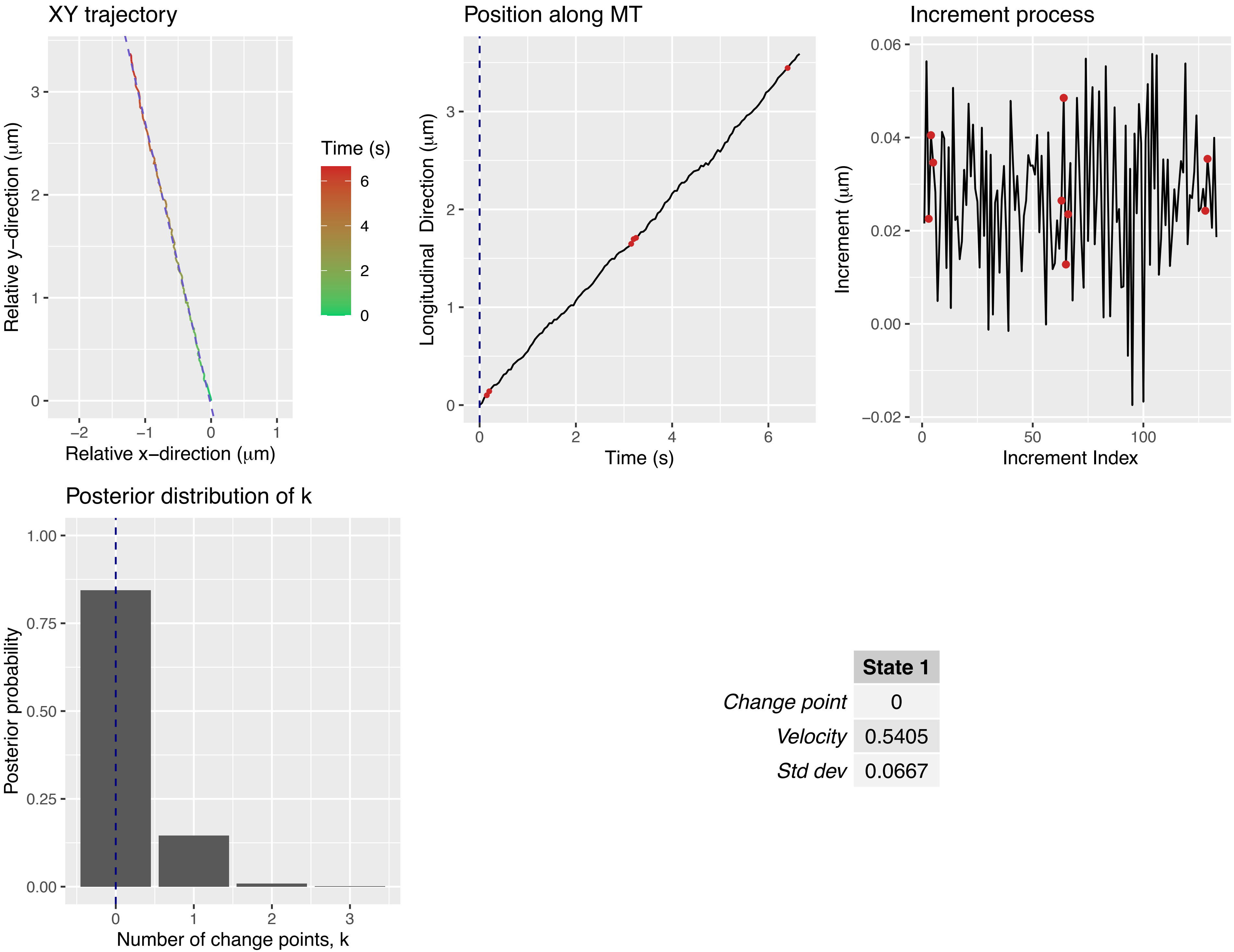

	State 1
Change point	0
Velocity	0.5405
Std dev	0.0667

Kin1 particle: 25

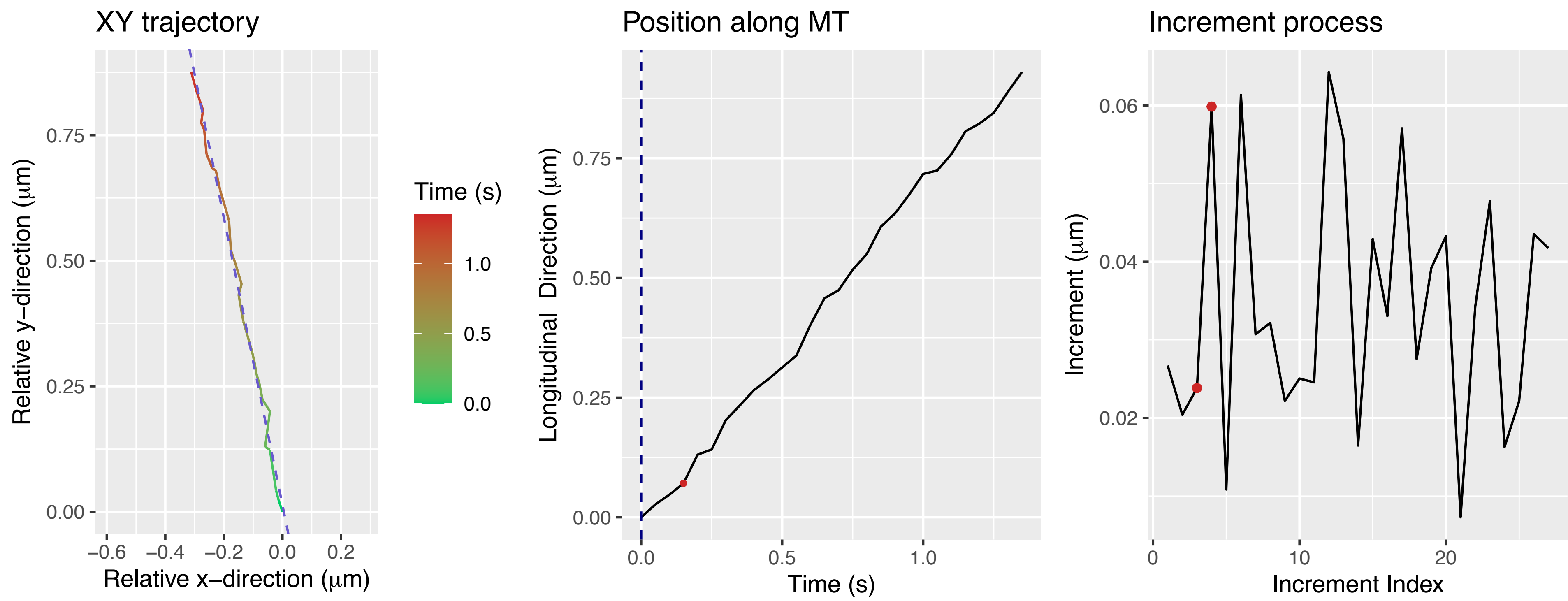

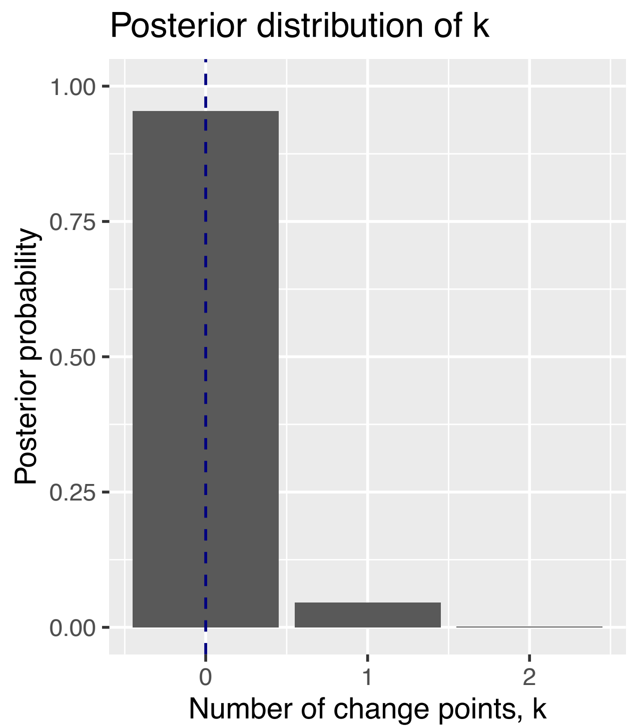

	State 1
Change point	0
Velocity	0.6887
Std dev	0.0664

Kin1 particle: 26

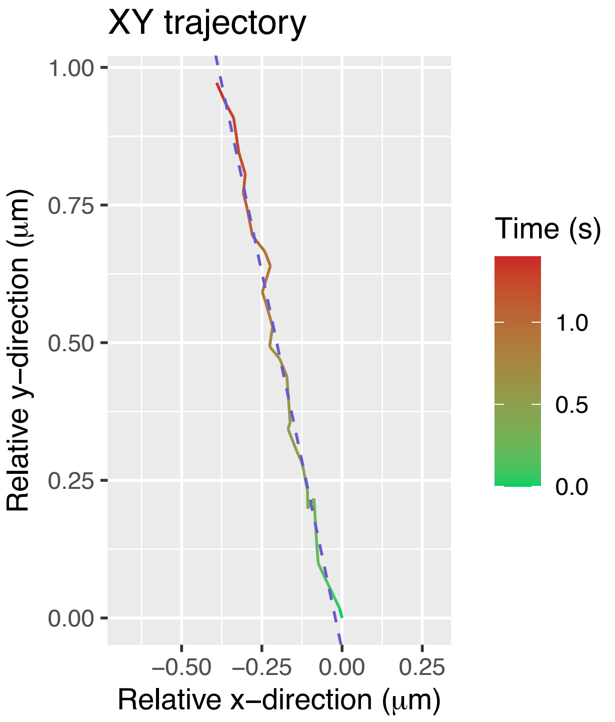

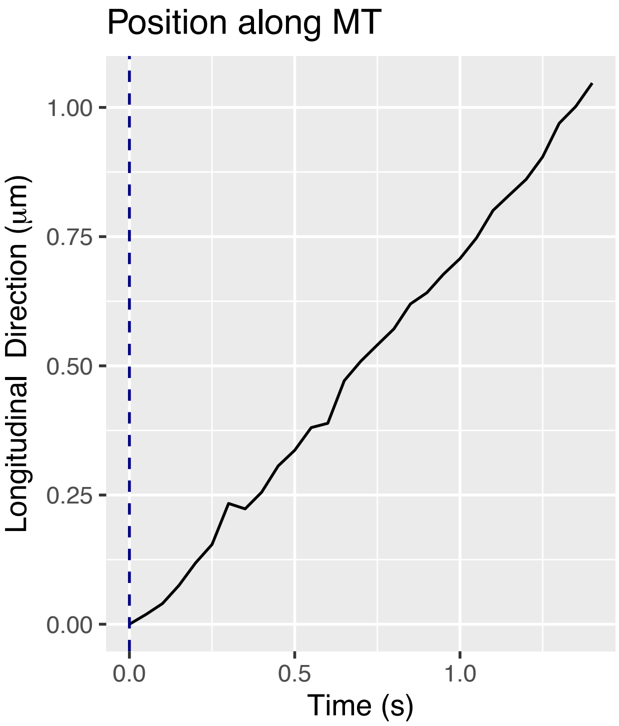

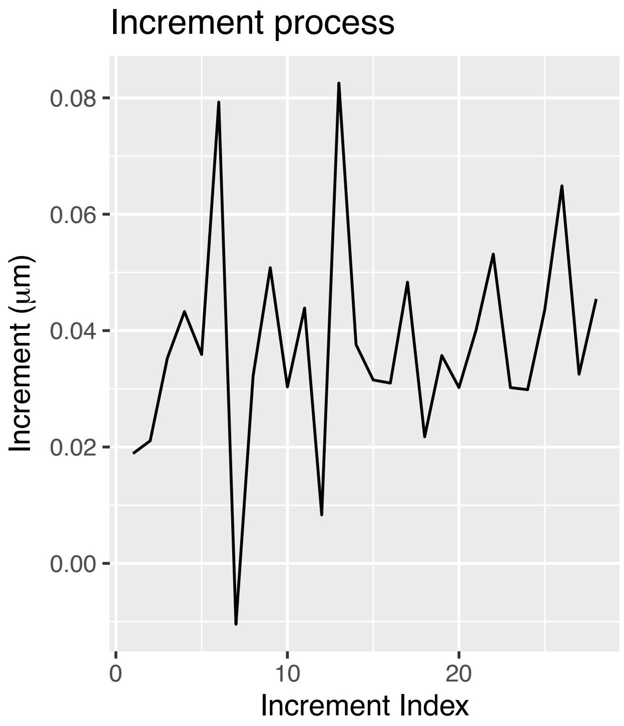

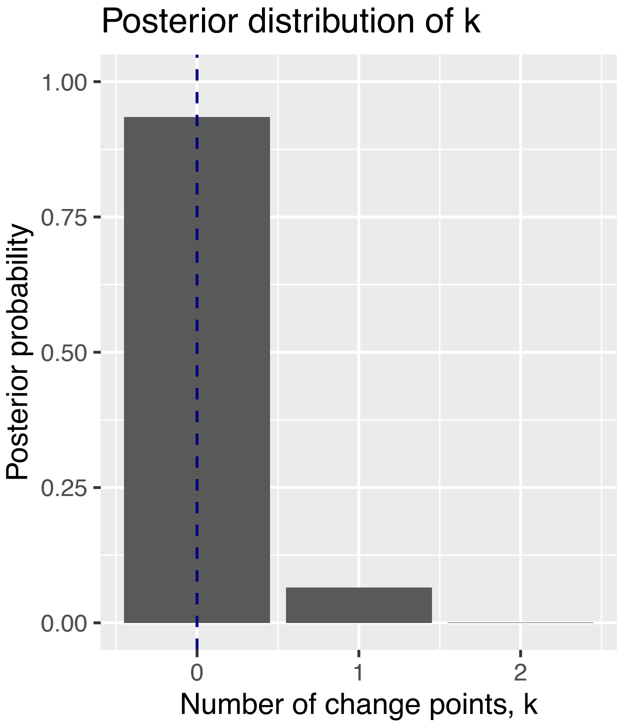

	**State 1**
Change point	0
Velocity	0.7486
Std dev	0.0802

Kin1 particle: 27

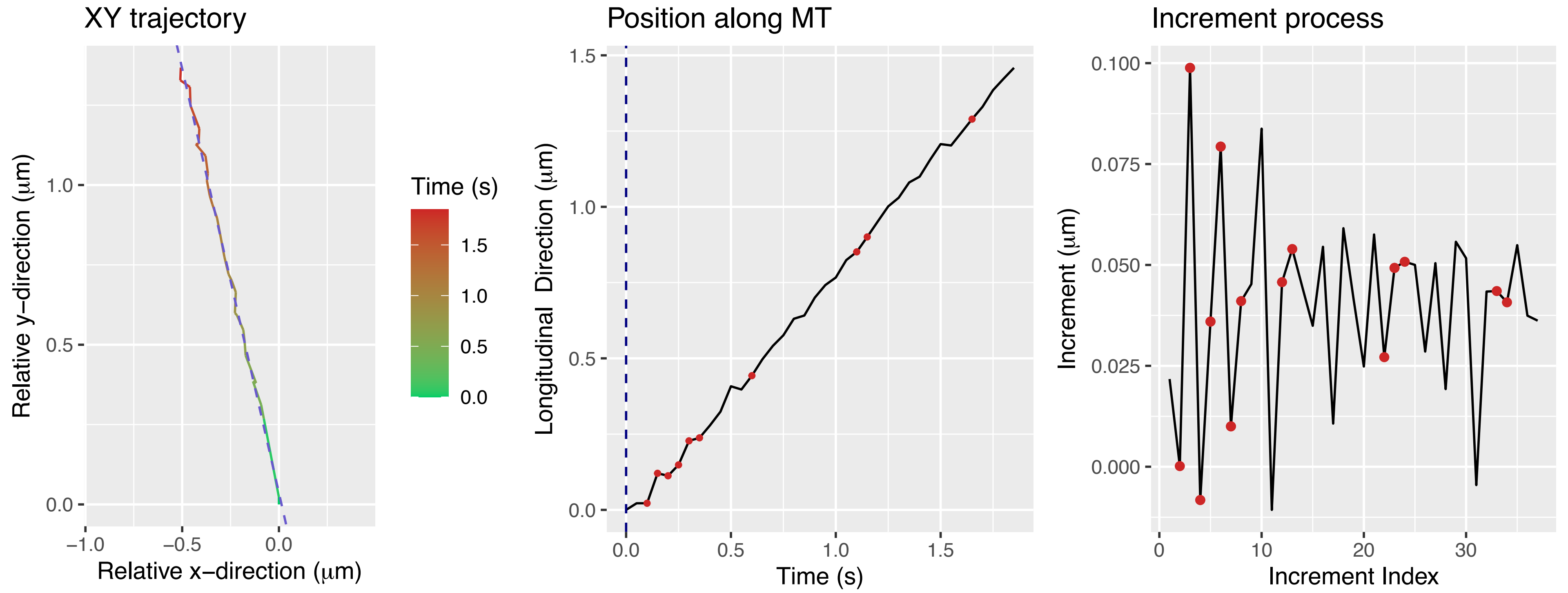

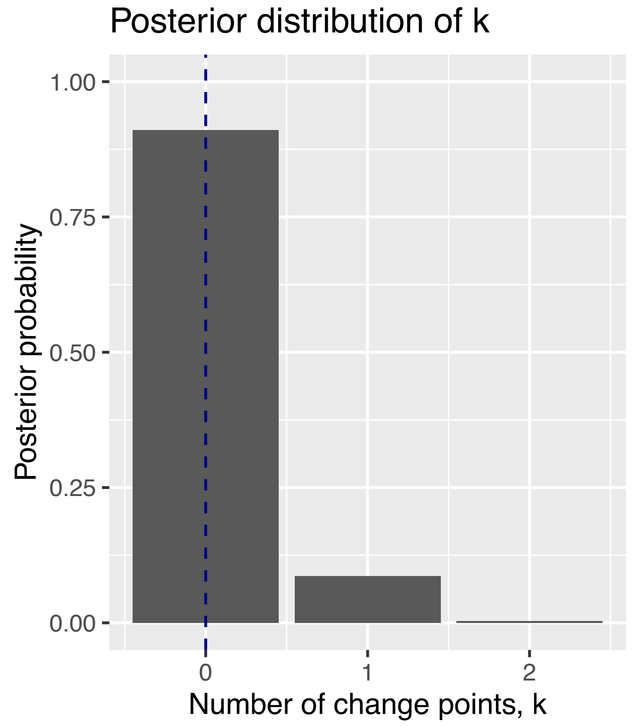

	State 1
Change point	0
Velocity	0.7884
Std dev	0.1059

Kin1 particle: 28

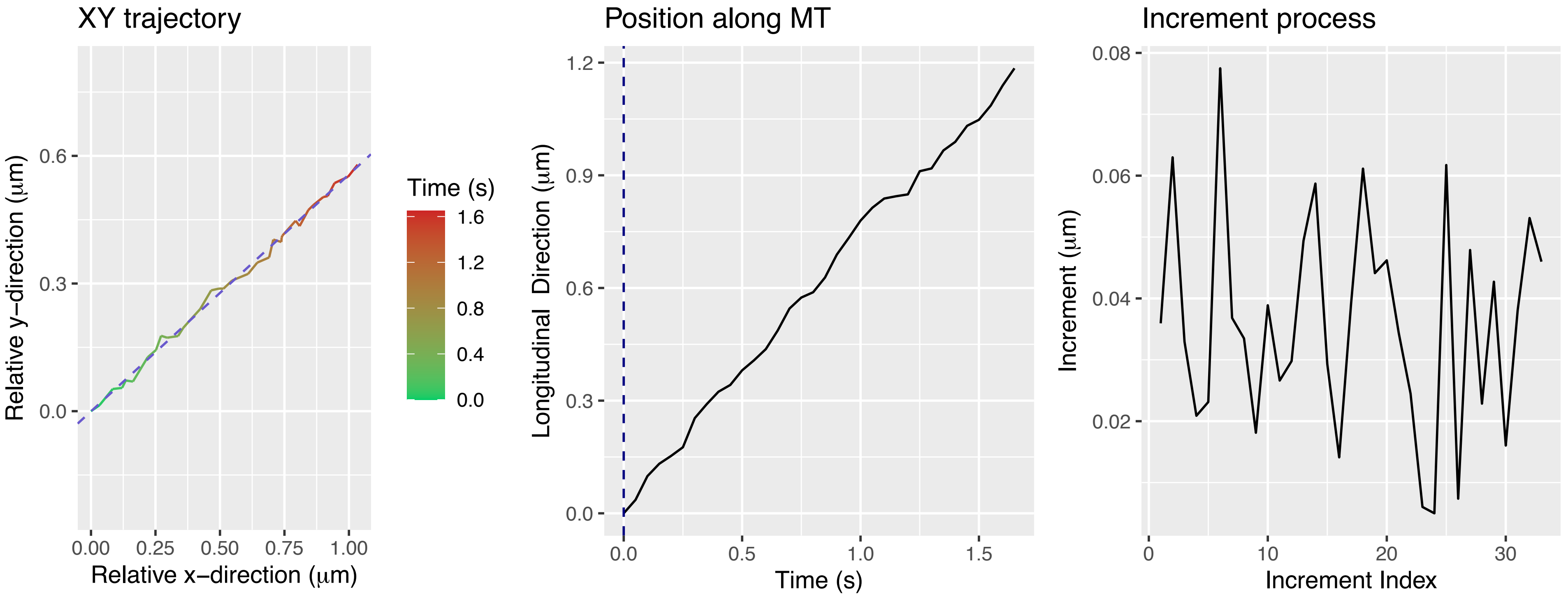

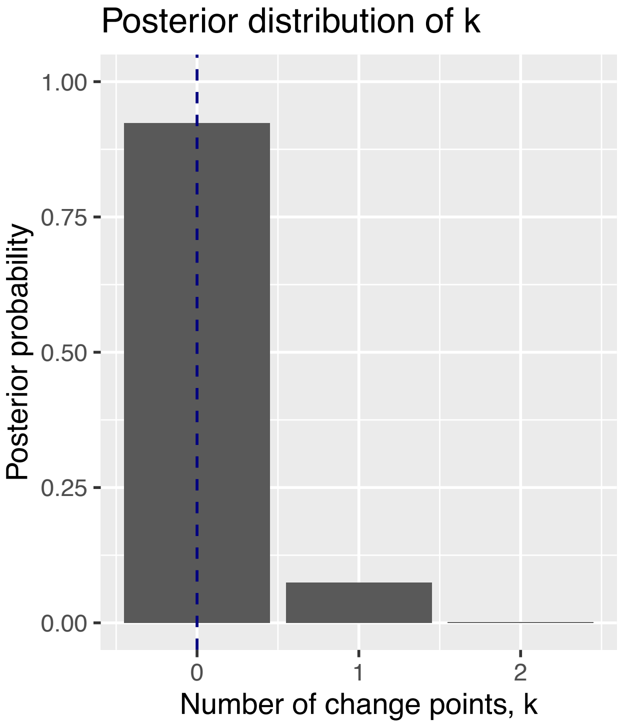

	State 1
Change point	0
Velocity	0.7204
Std dev	0.0761

Kin1 particle: 29

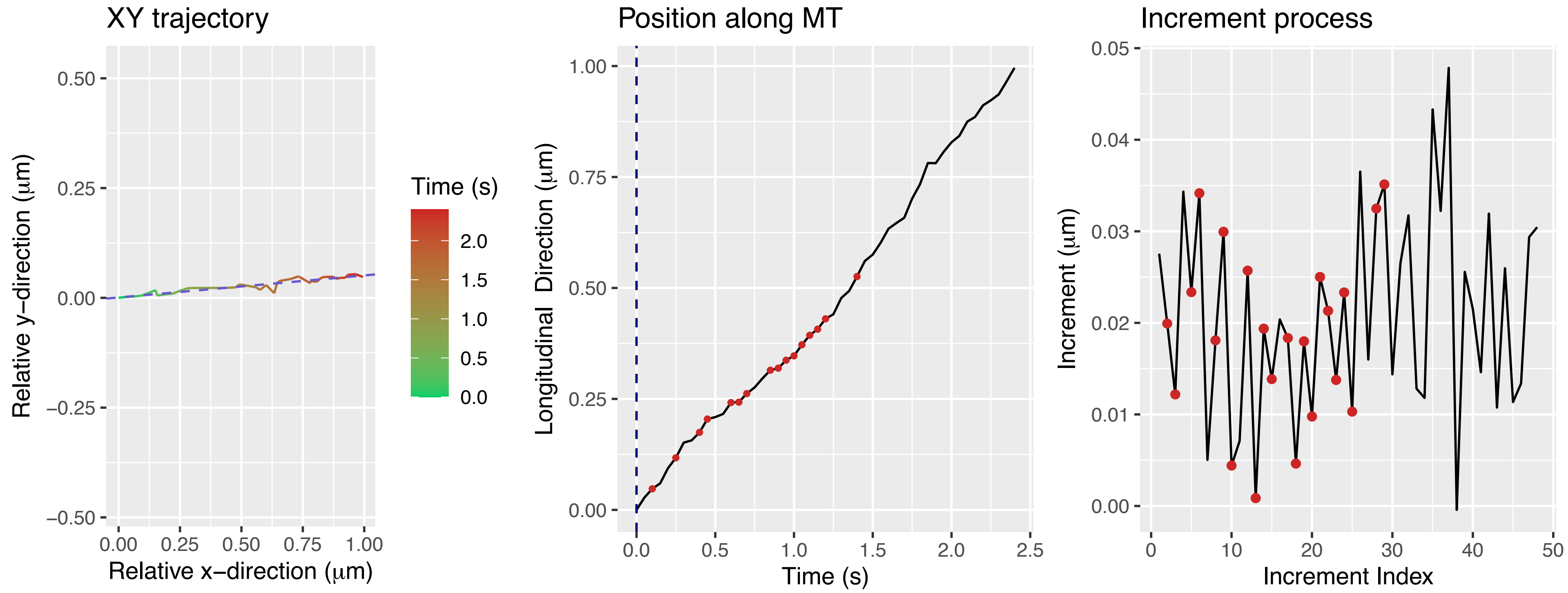

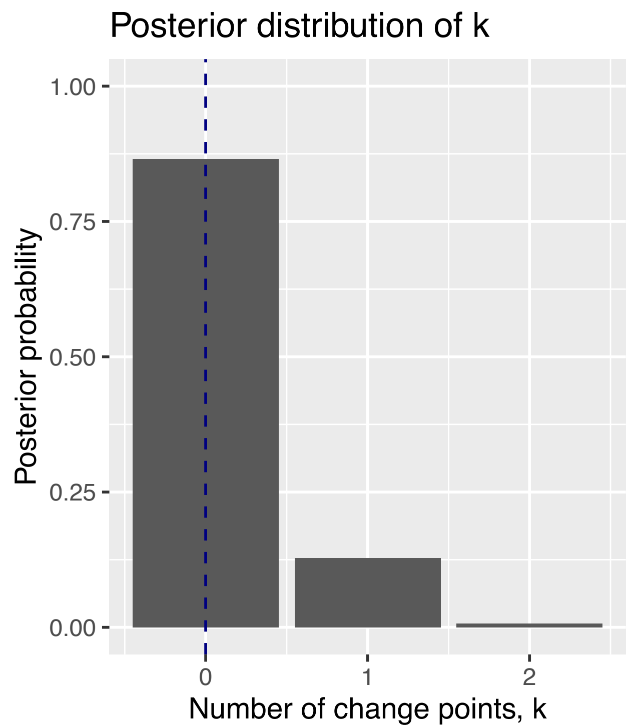

	State 1
Change point	0
Velocity	0.4147
Std dev	0.0455

Kin1 particle: 30

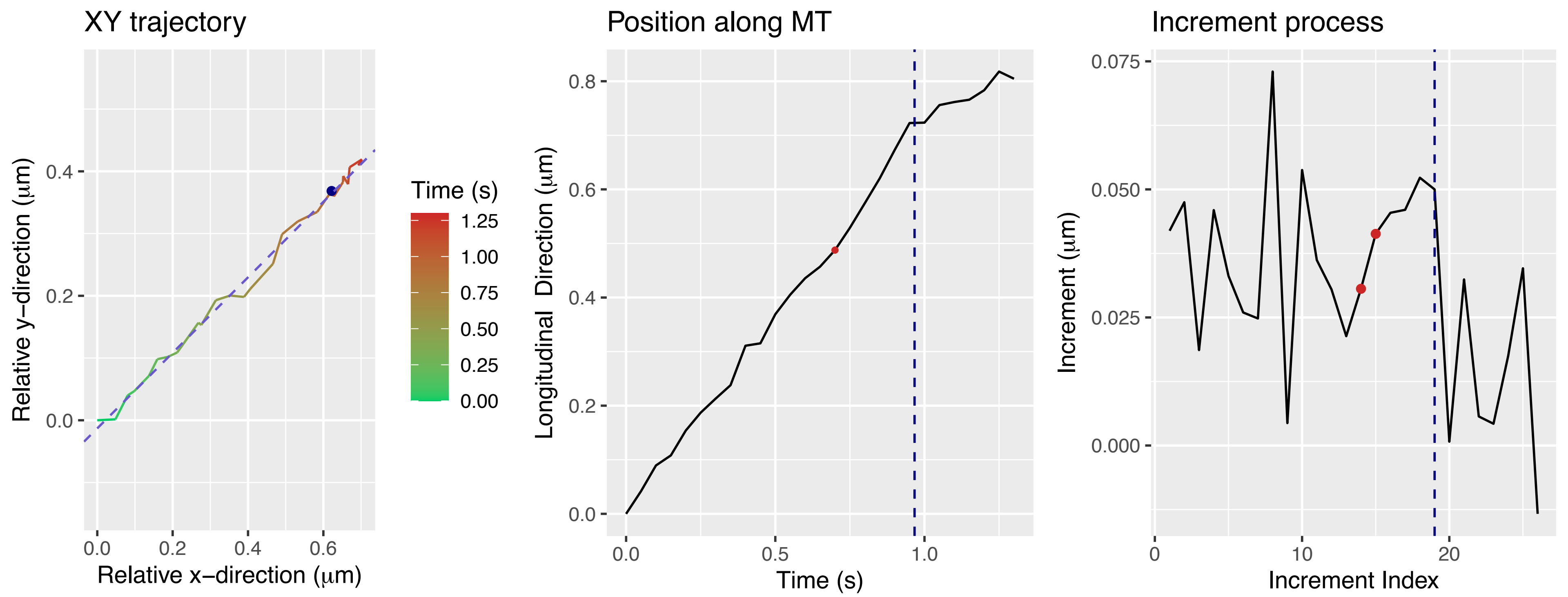

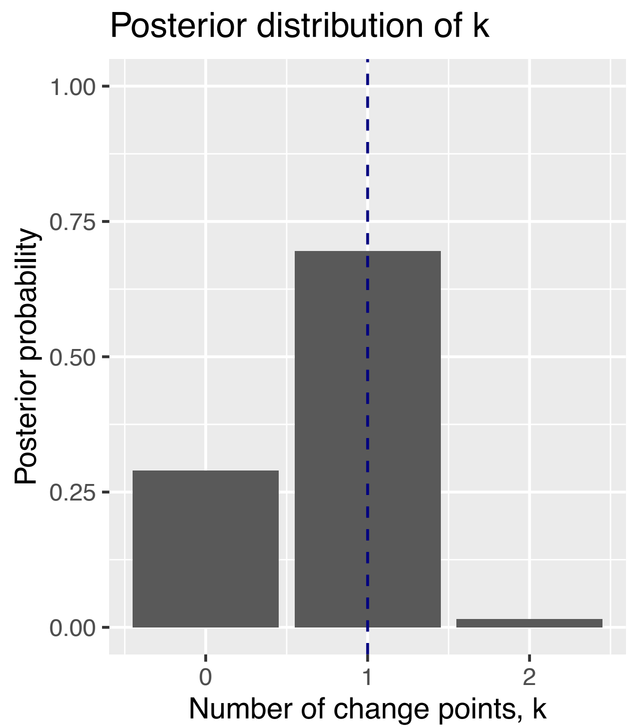

	State 1	State 2
Change point	0.9667	NA
Velocity	0.7538	0.2235
Std dev	0.0744	NA

Kin1 particle: 31

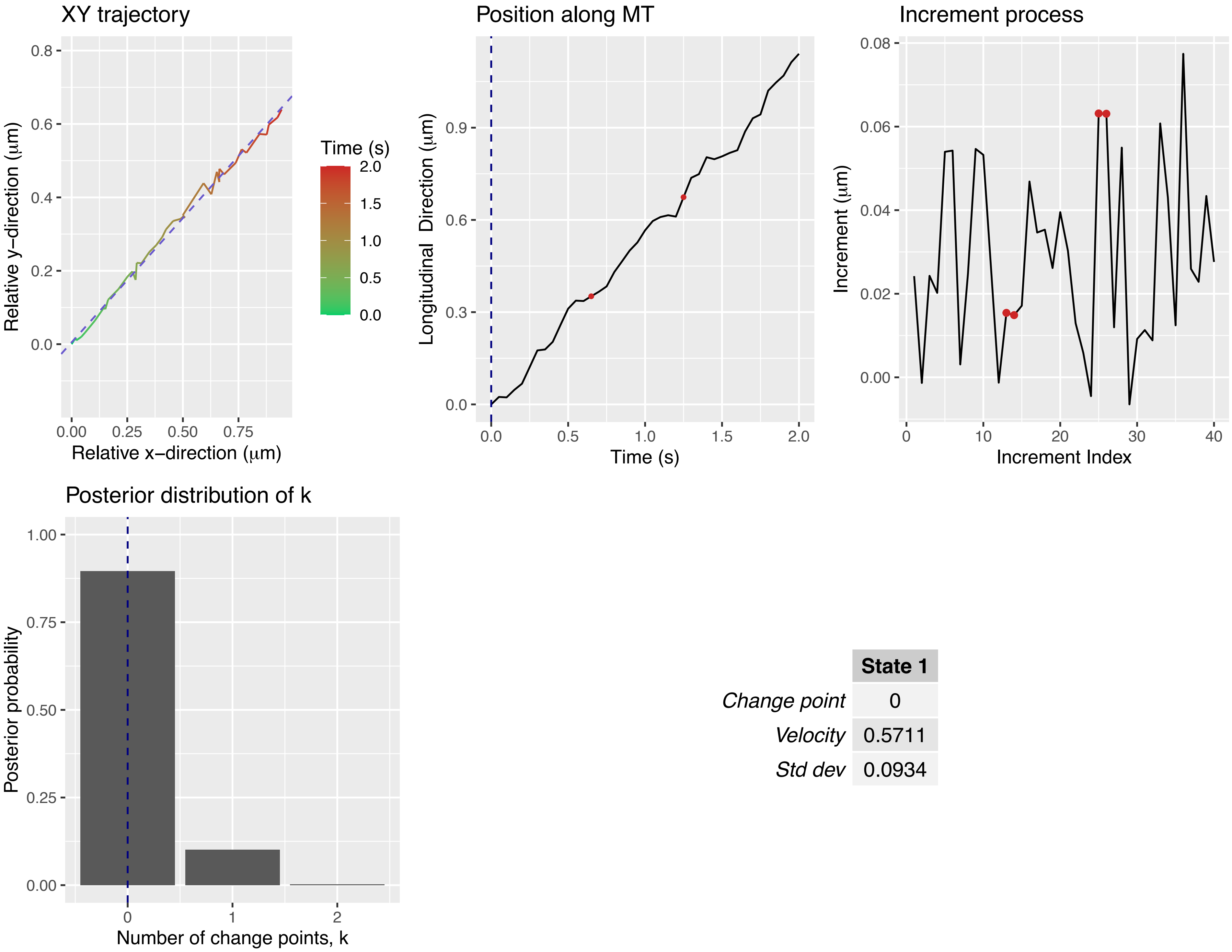

	State 1
Change point	0
Velocity	0.5711
Std dev	0.0934

Kin1 particle: 32

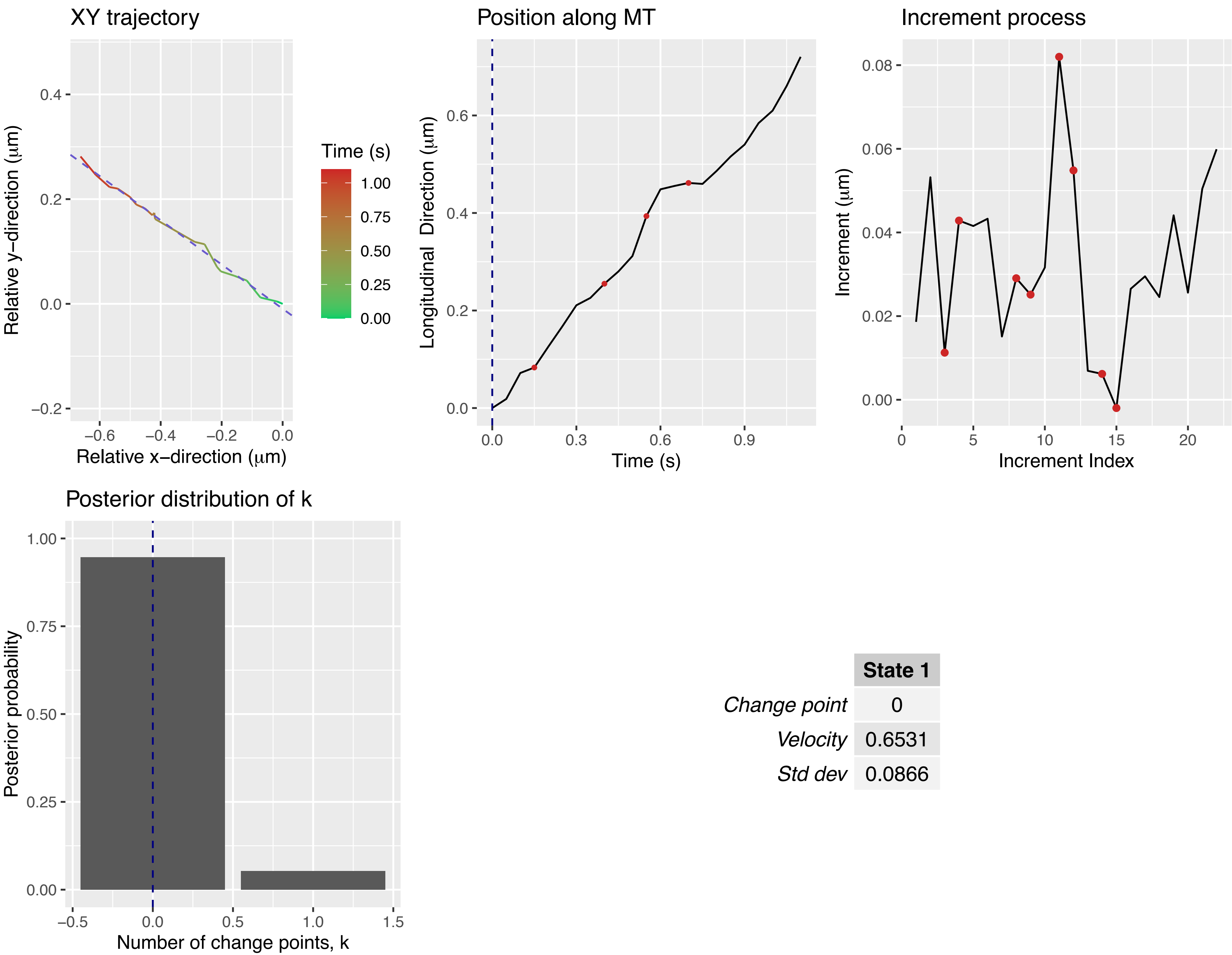

	State 1
Change point	0
Velocity	0.6531
Std dev	0.0866

Kin1 particle: 33

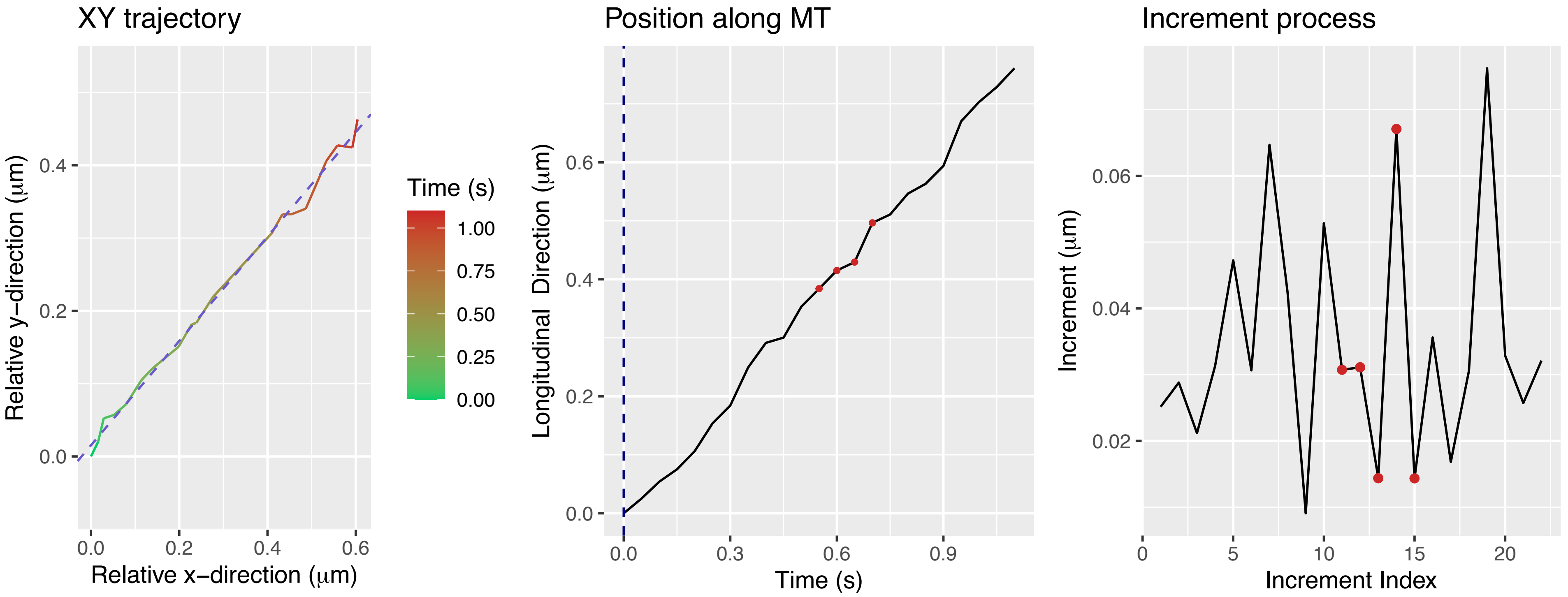

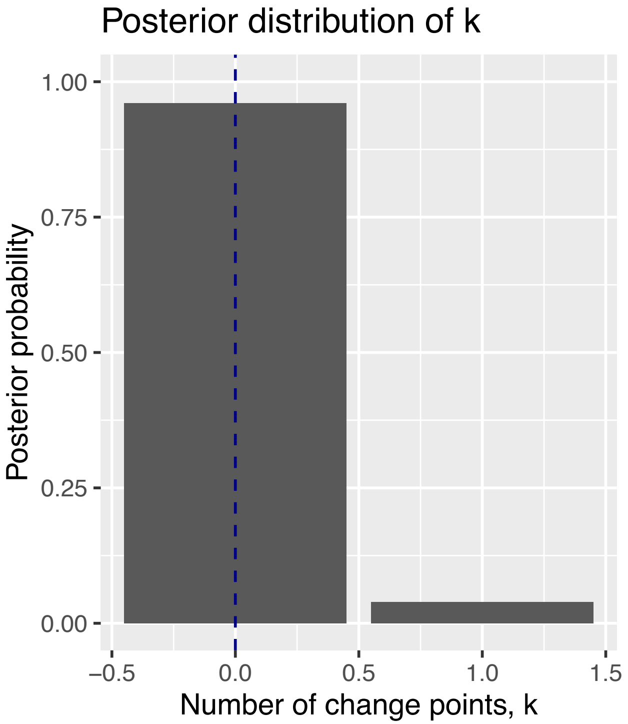

	State 1
Change point	0
Velocity	0.692
Std dev	0.0728

Kin1 particle: 34

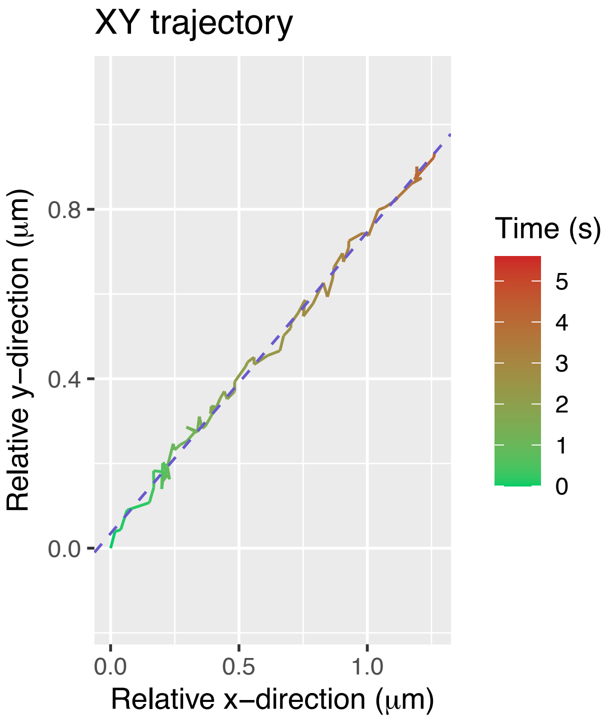

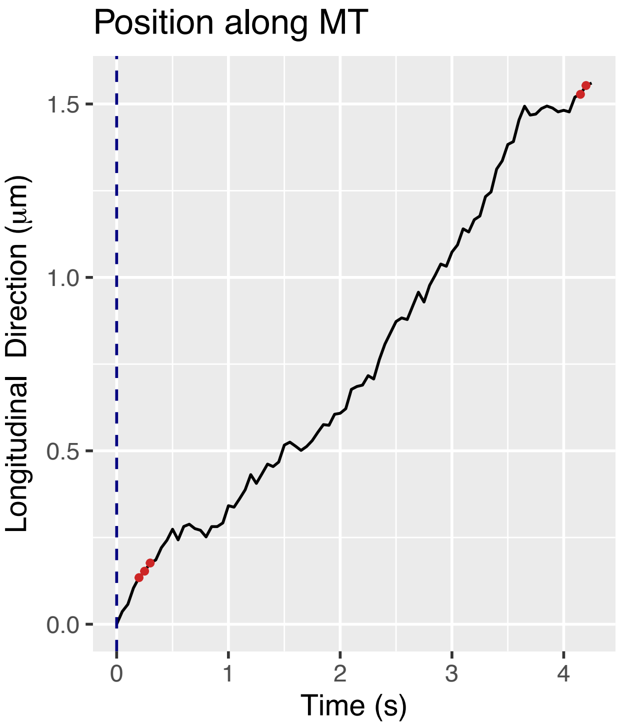

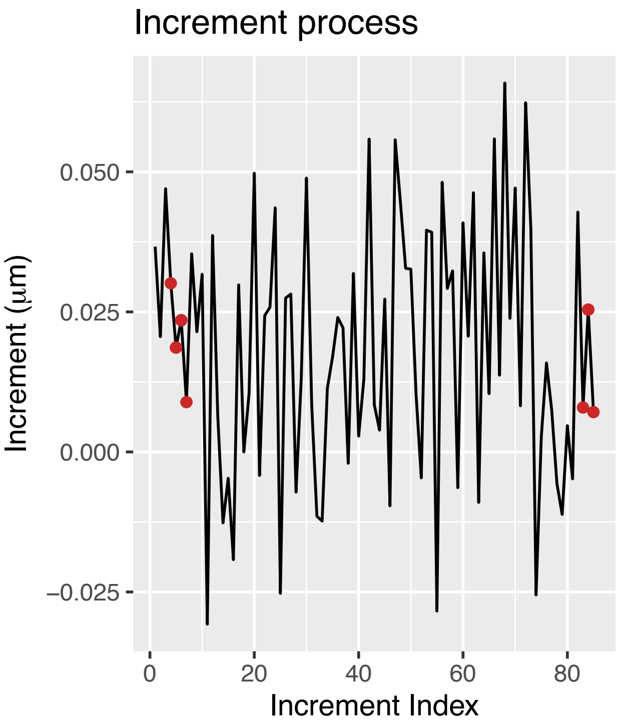

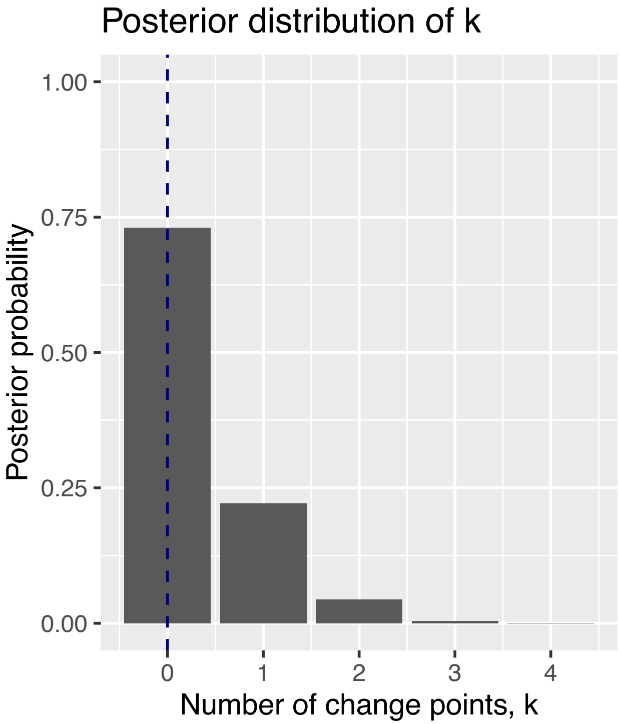

	**State 1**
Change point	0
Velocity	0.3661
Std dev	0.1003

Kin1 particle: 35

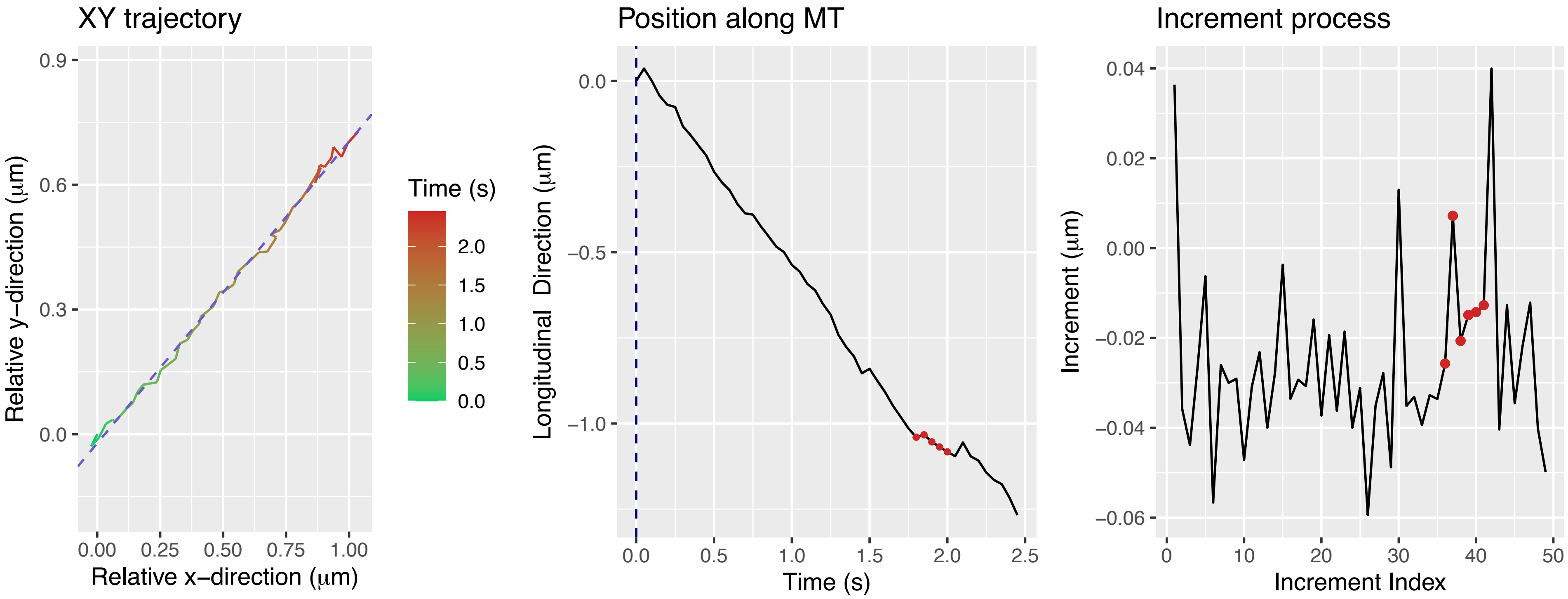

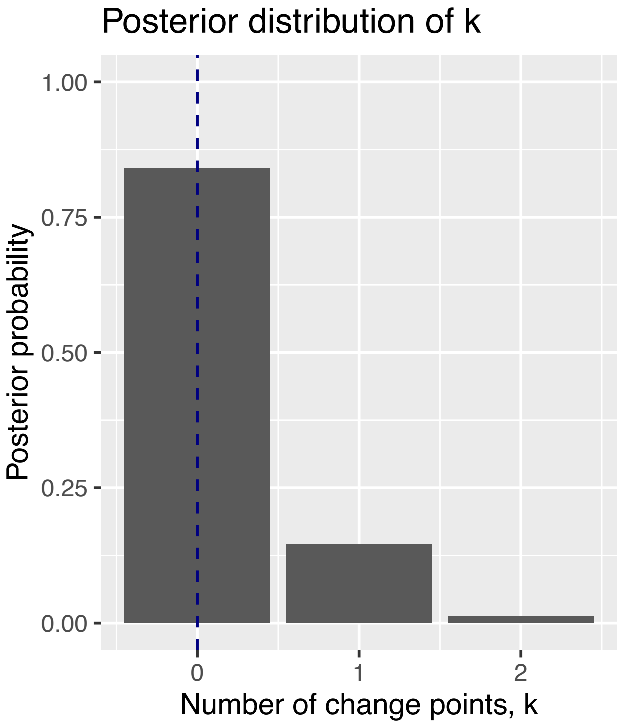

	State 1
Change point	0
Velocity	–0.5167
Std dev	0.086

Kin1 particle: 36

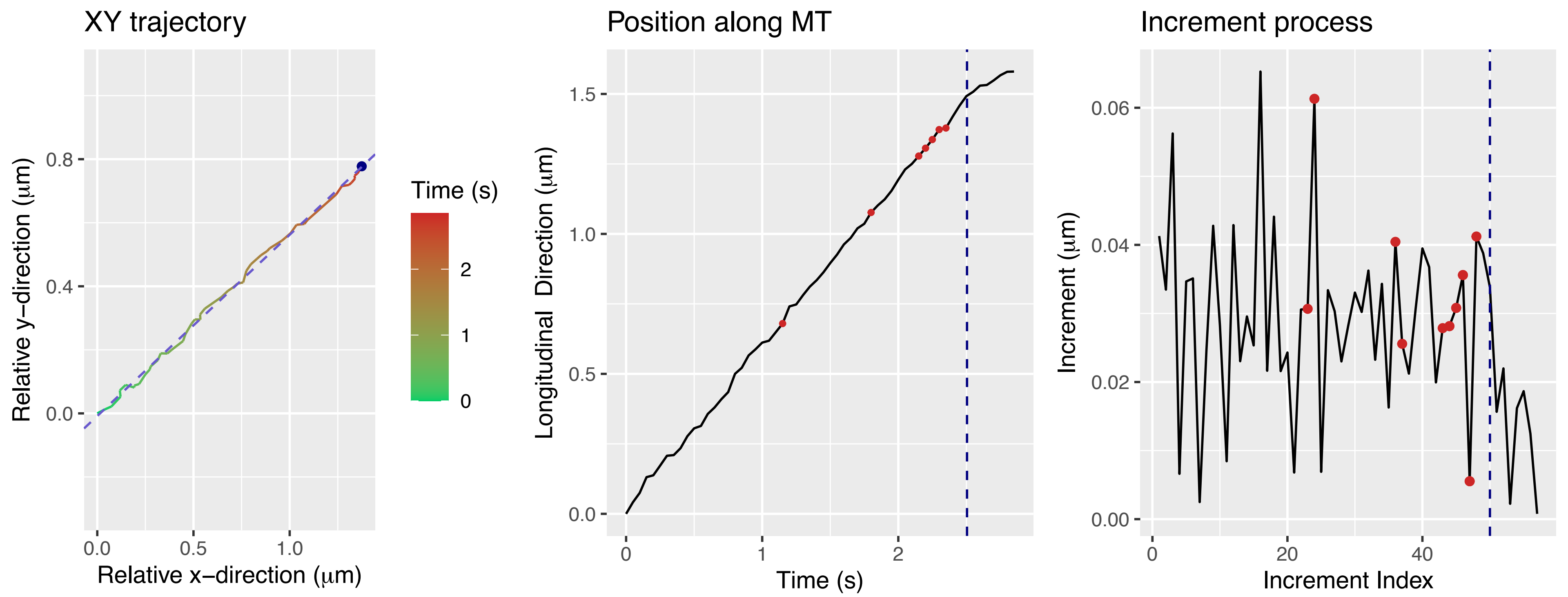

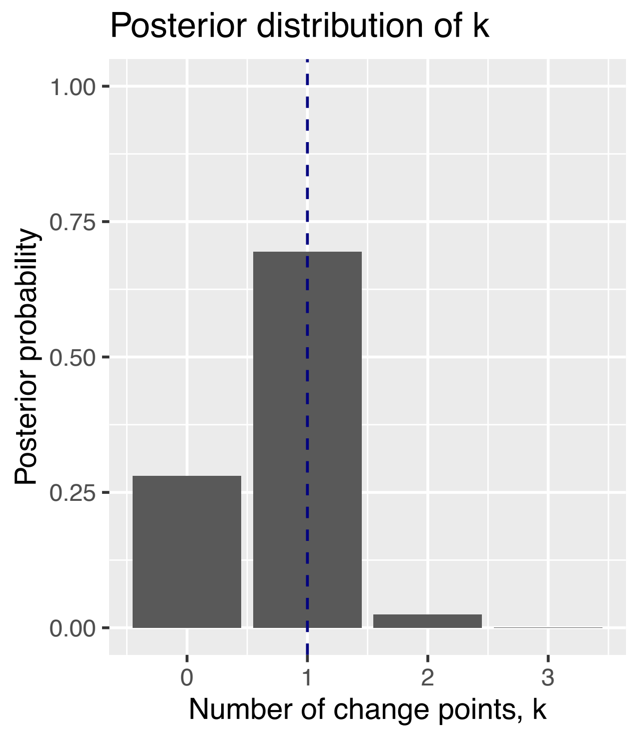

	State 1	State 2
Change point	2.5048	NA
Velocity	0.5933	0.2556
Std dev	0.056	NA

Kin1 particle: 37

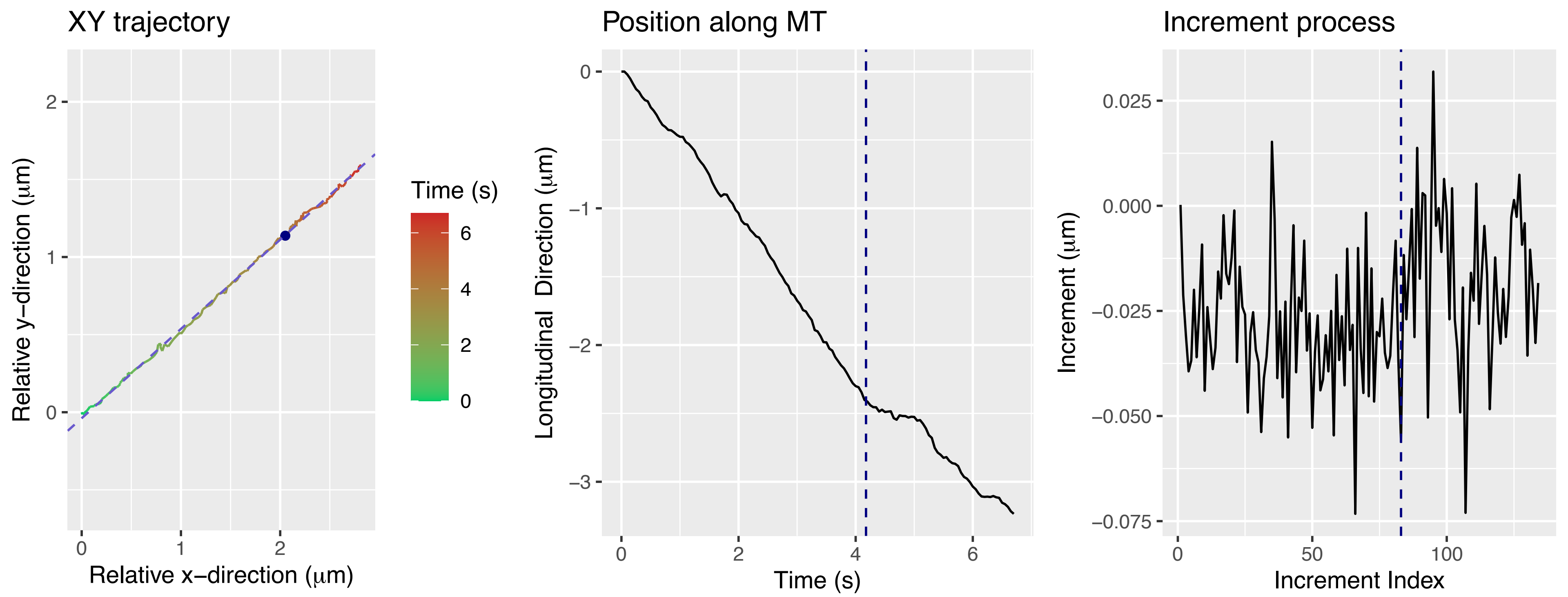

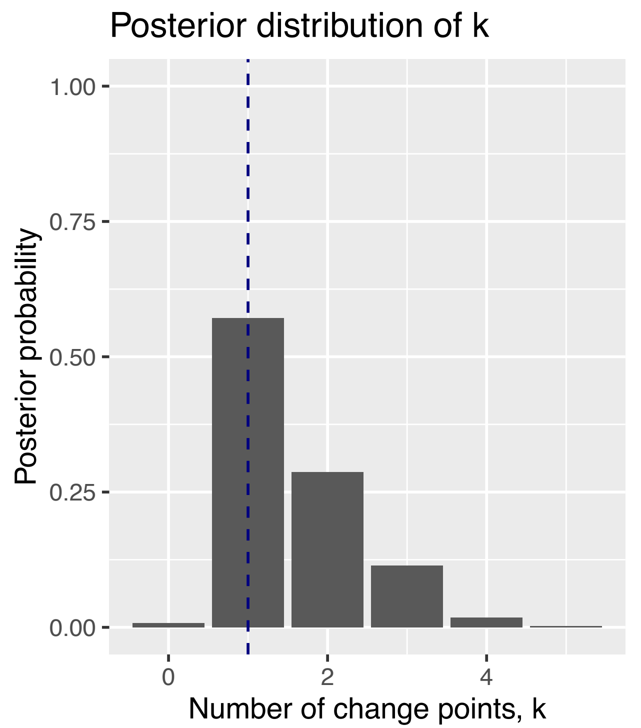

	State 1	State 2
Change point	4.1765	NA
Velocity	−0.5873	−0.3283
Std dev	0.0735	NA

Kin1 particle: 38

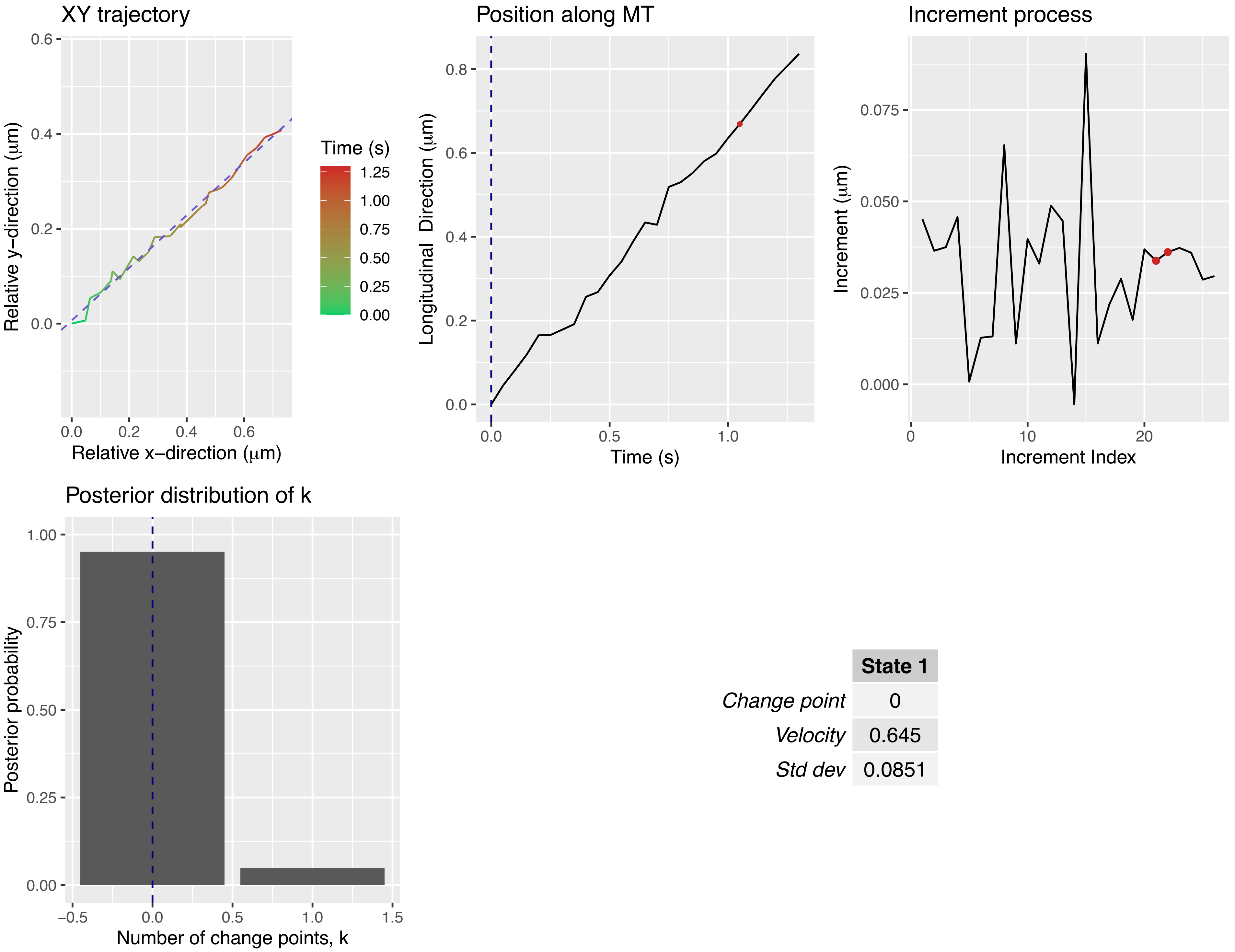

	State 1
Change point	0
Velocity	0.645
Std dev	0.0851

Kin1 particle: 39

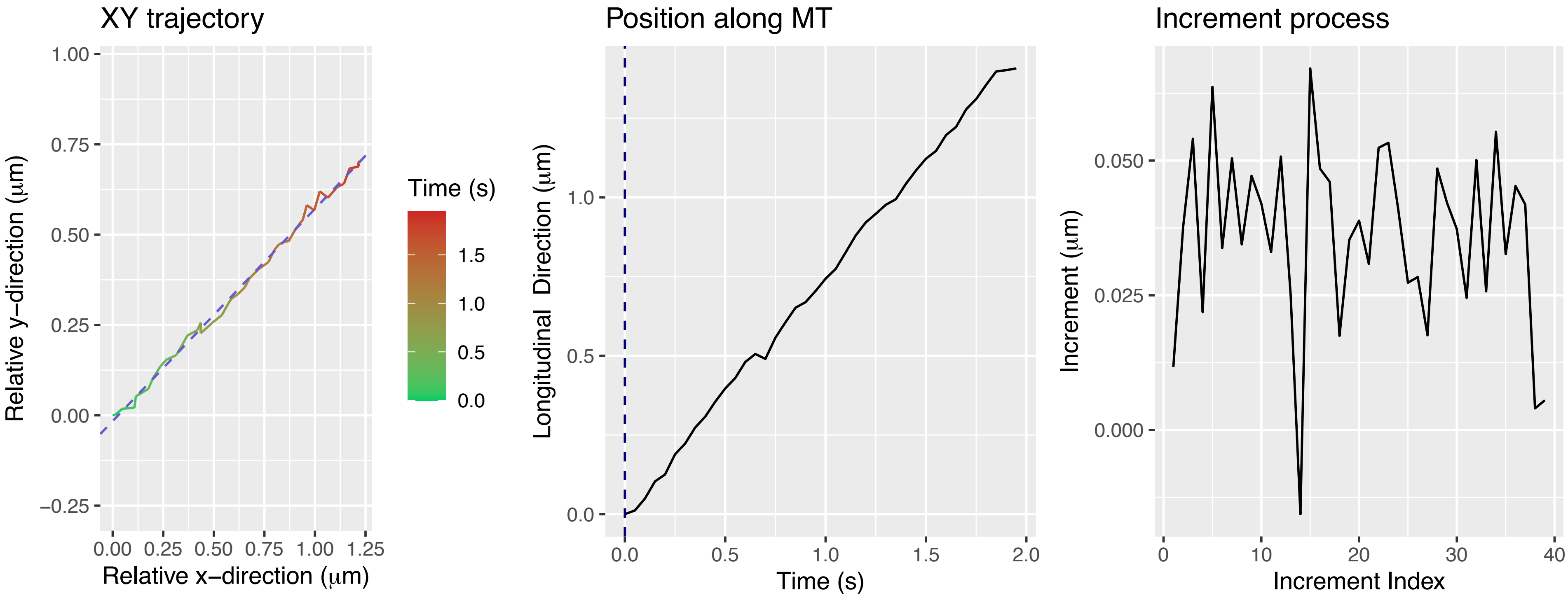

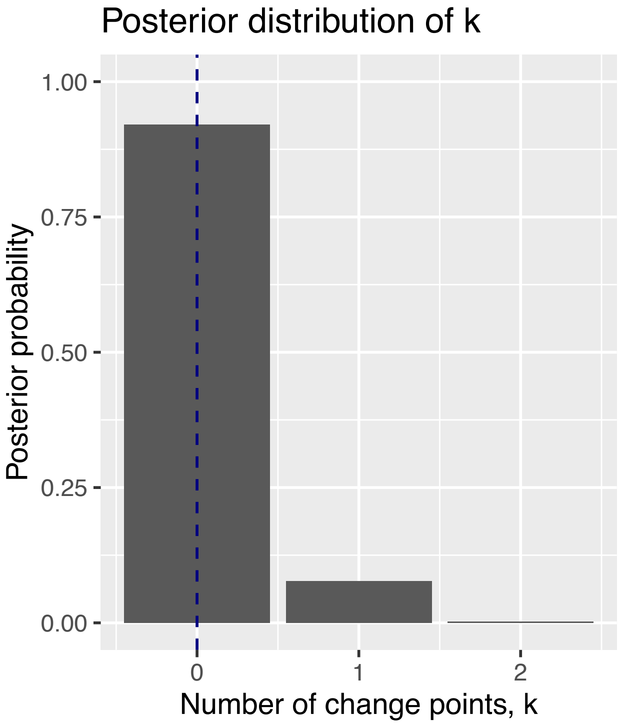

	State 1
Change point	0
Velocity	0.7226
Std dev	0.0732

Kin1 particle: 40

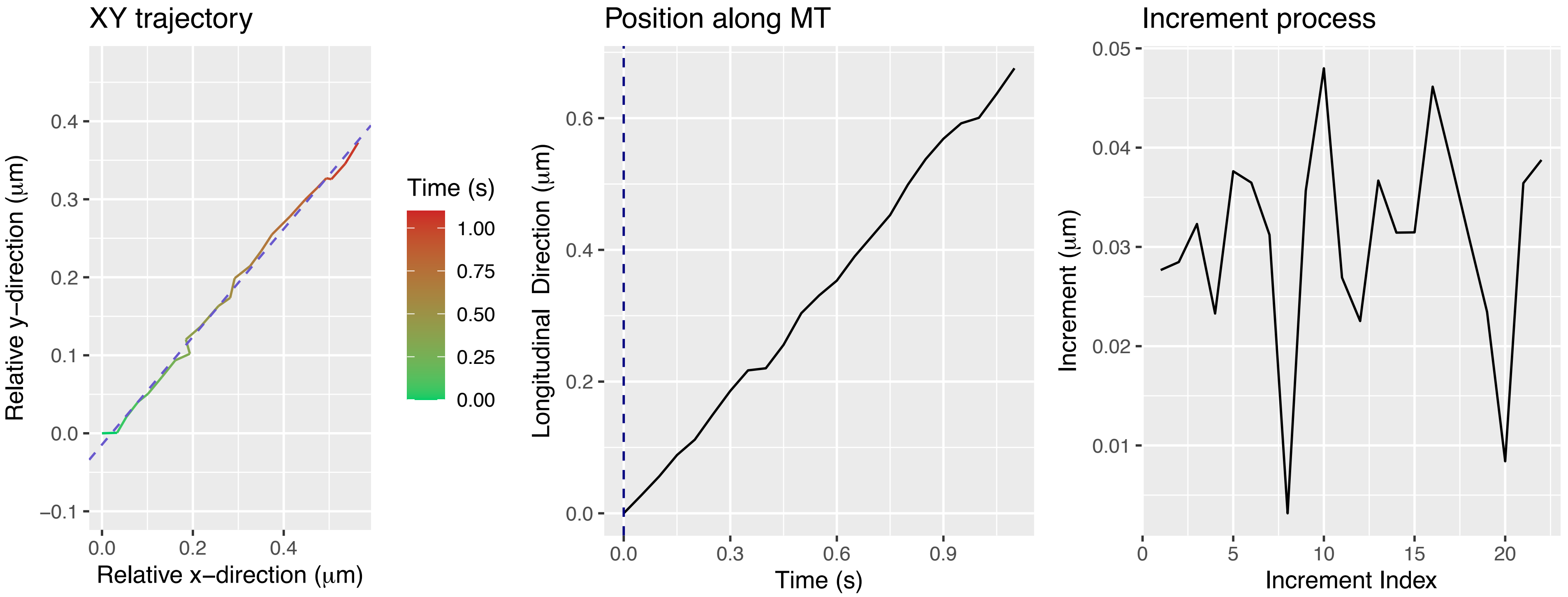

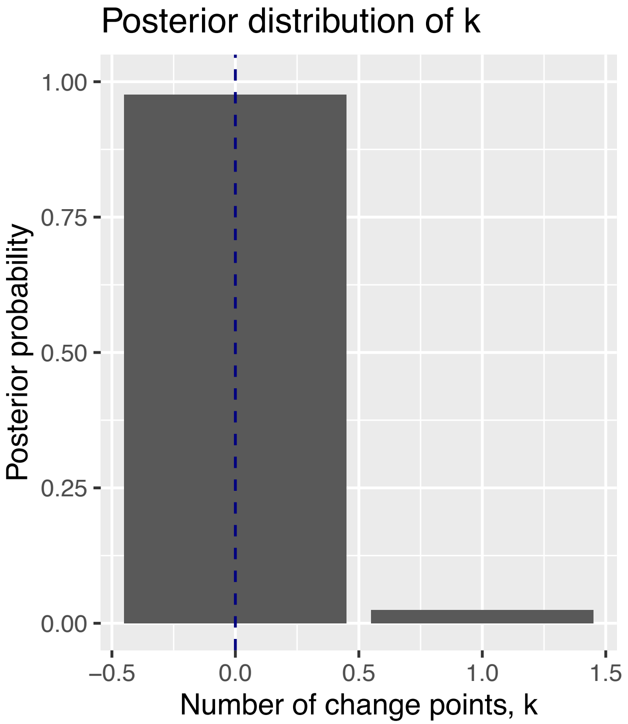

	State 1
Change point	0
Velocity	0.6151
Std dev	0.0407
