# Supplementary material for: A change point analysis protocol for comparing intracellular transport by different molecular motor combinations
Source: Math Biosci Eng. Author manuscript; Available in PMC 2023 Jan 6. (PMC9817212; doi:10.3934/mbe.2021442)
Supplement: supplemental material [file NIHMS1850682-supplement-supplemental_material.zip › mbe-18-06-442 supplementary/SI_Kin1DDB.pdf]

Kin1DDB particle: 1

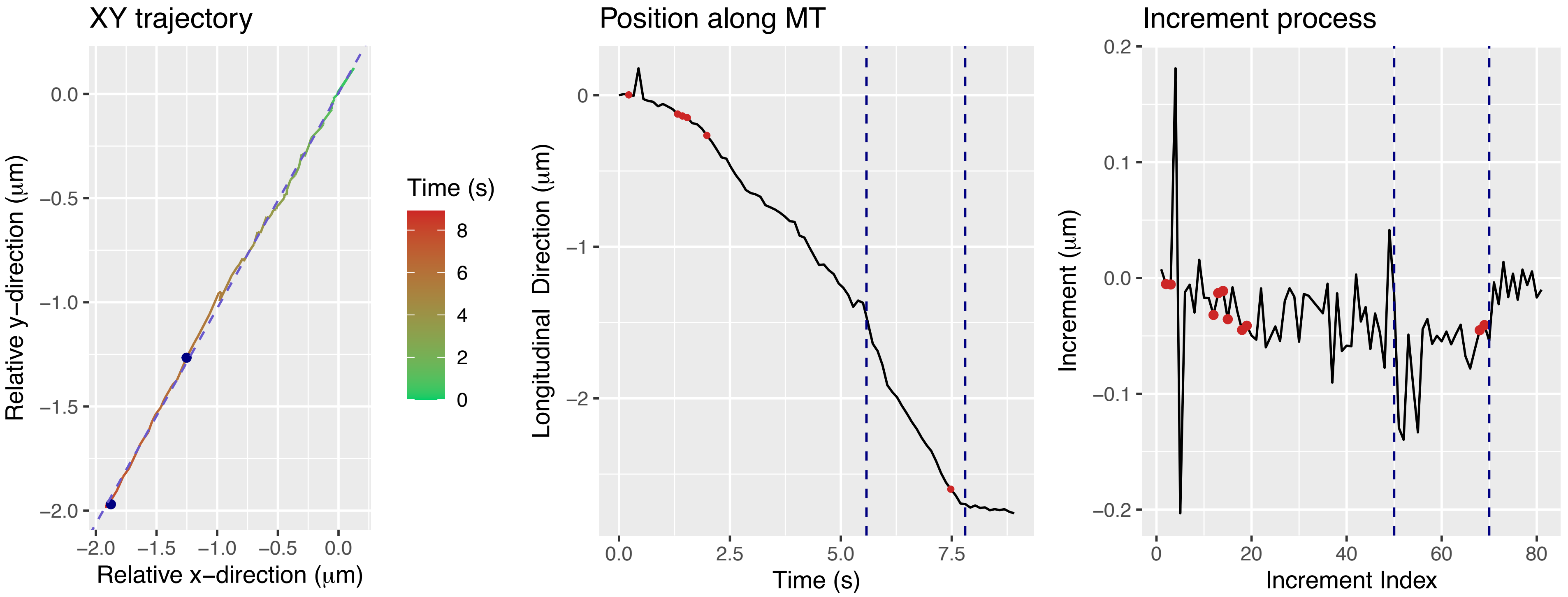

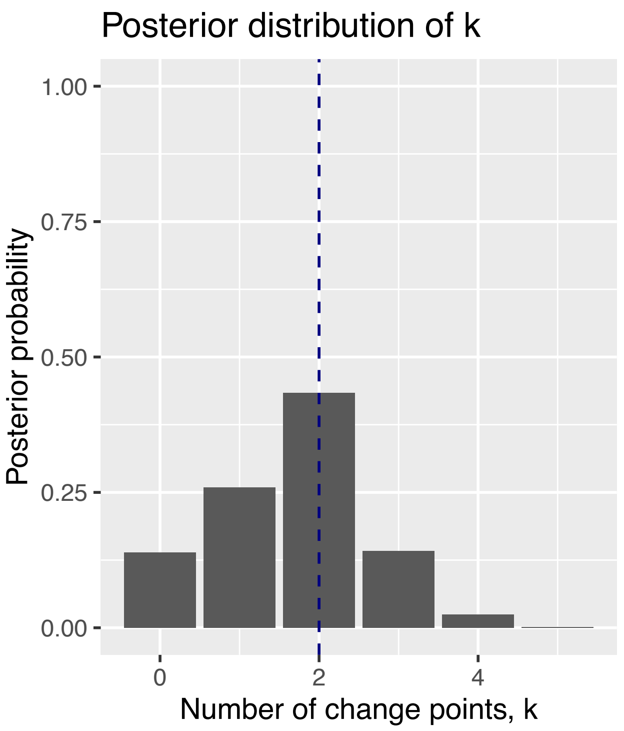

	State 1	State 2	State 3
Change point	5.5785	7.8032	NA
Velocity	−0.2387	−0.6308	−0.0593
Std dev	0.1229	NA	NA

Kin1DDB particle: 2

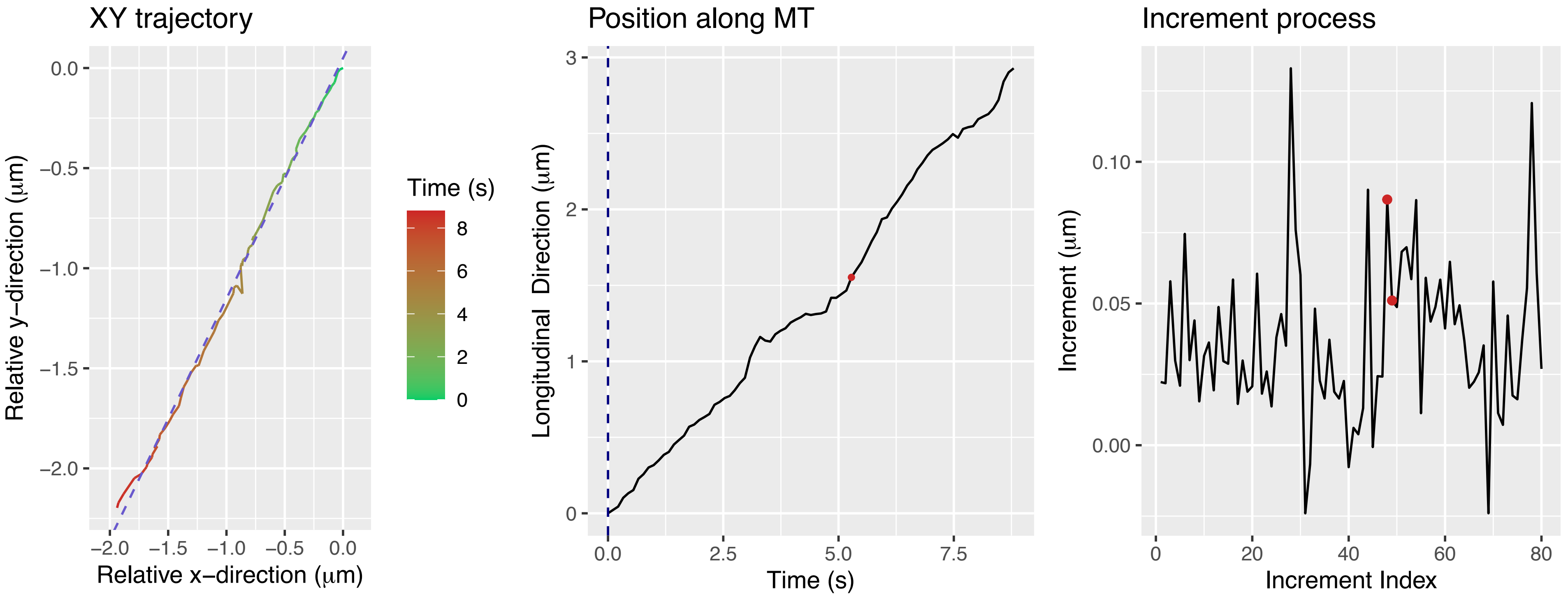

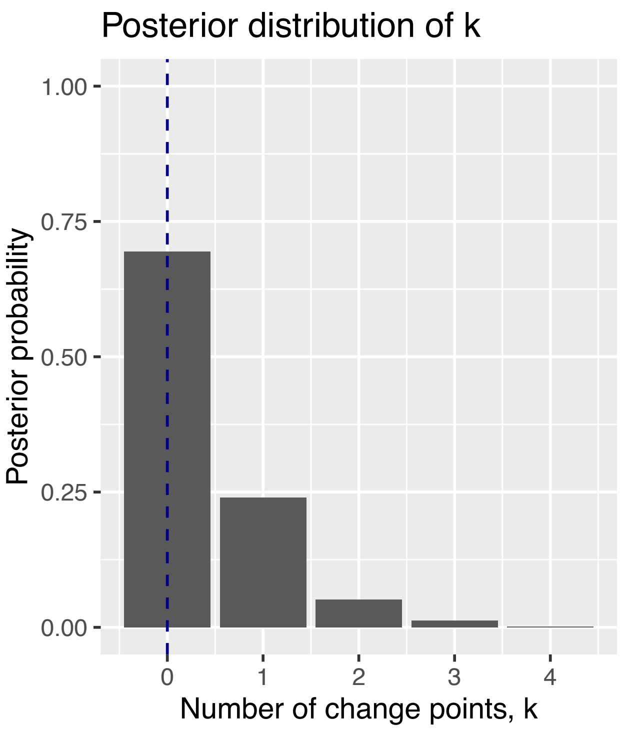

	State 1
Change point	0
Velocity	0.3321
Std dev	0.0822

Kin1DDB particle: 3

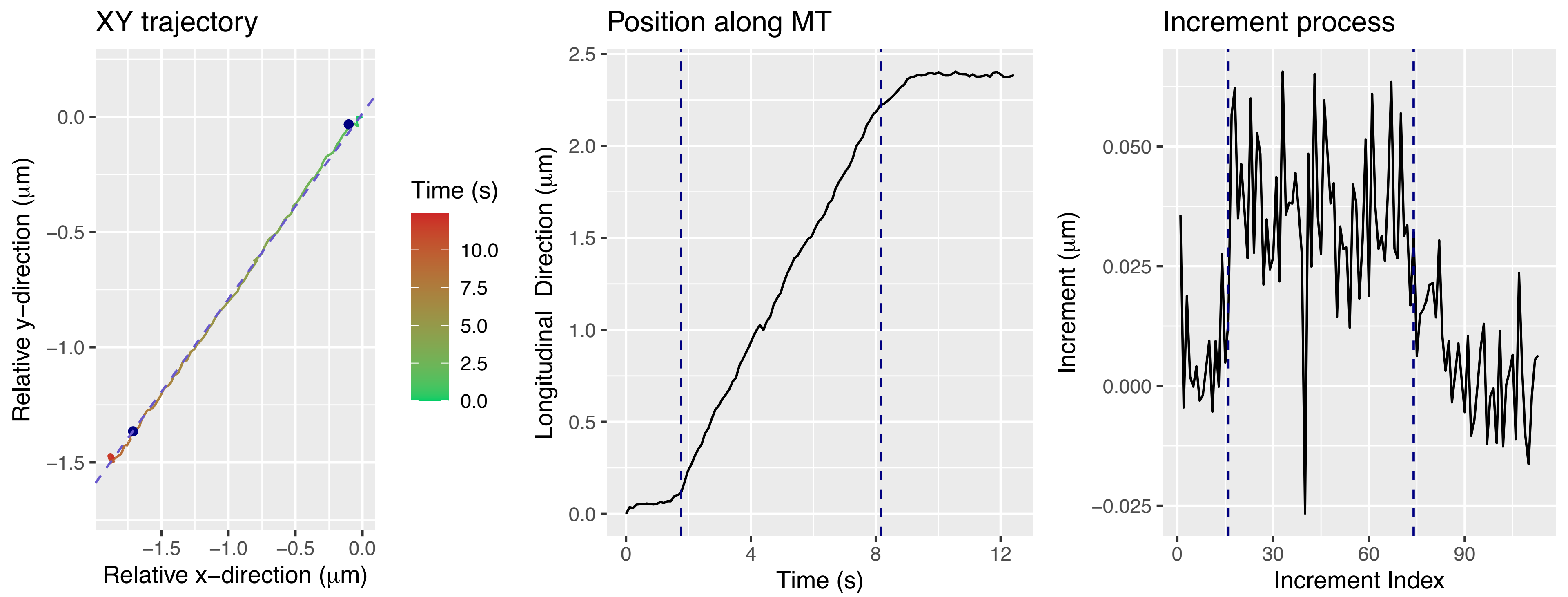

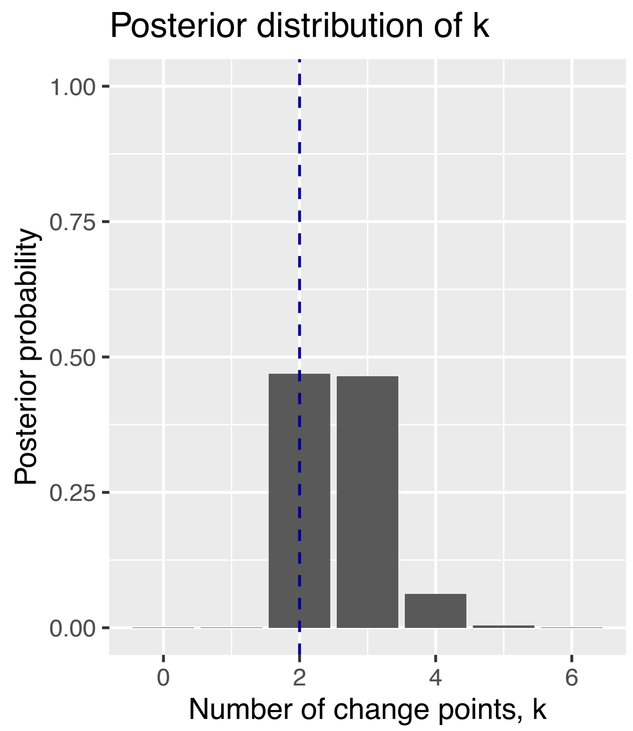

	State 1	State 2	State 3
Change point	1.7632	8.1645	NA
Velocity	0.0701	0.3254	0.0357
Std dev	0.0441	NA	NA

Kin1DDB particle: 4

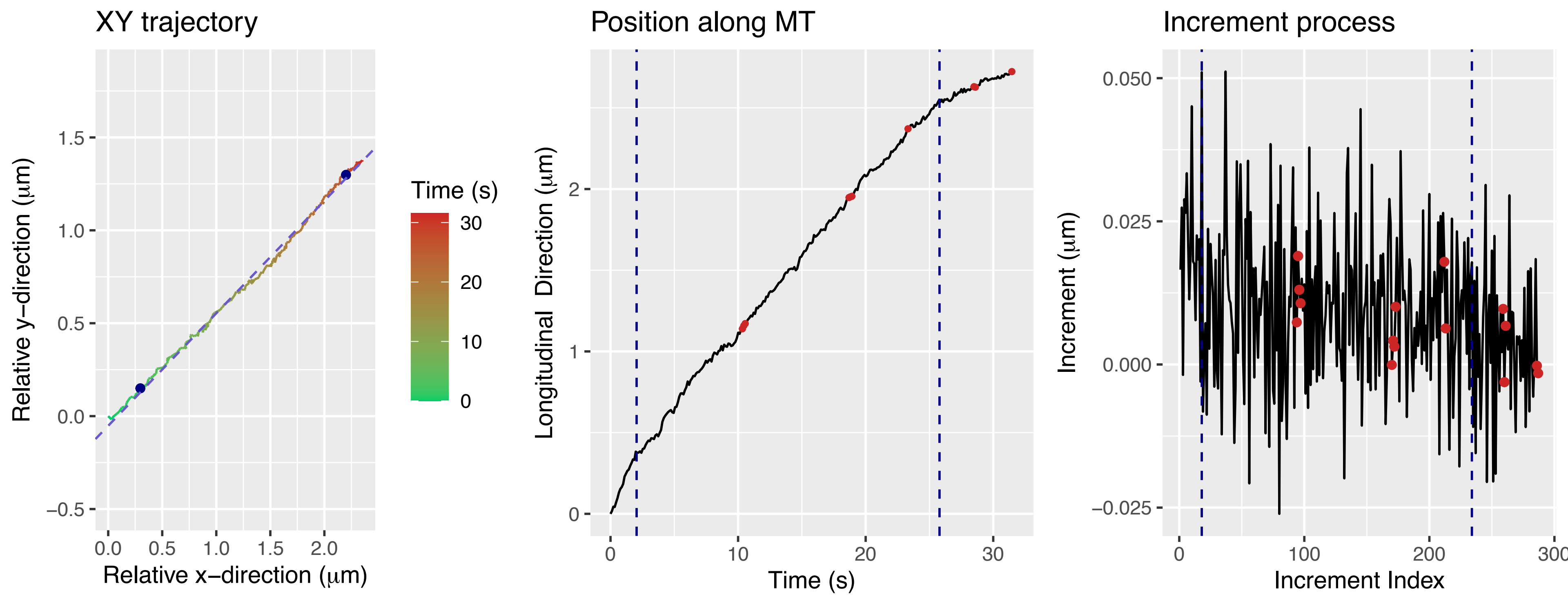

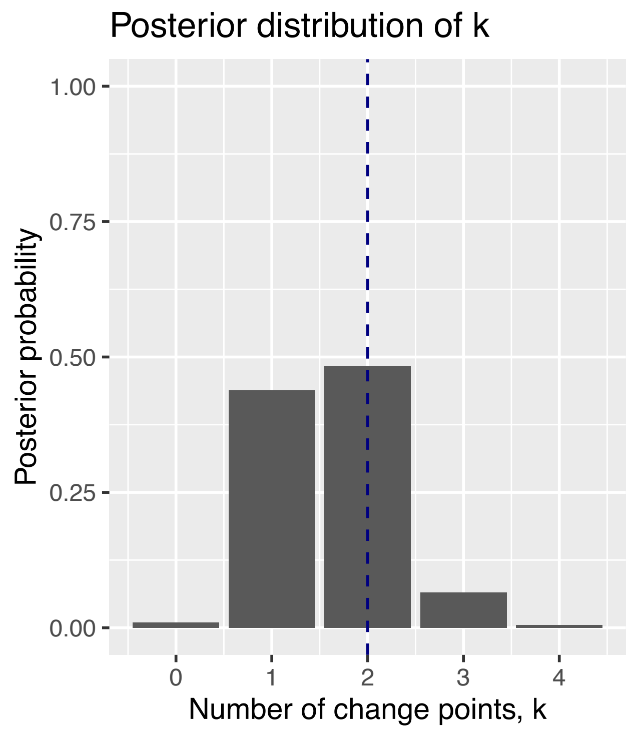

	State 1	State 2	State 3
Change point	2.0447	25.7894	NA
Velocity	0.1915	0.0907	0.0331
Std dev	0.0375	NA	NA

Kin1DDB particle: 5

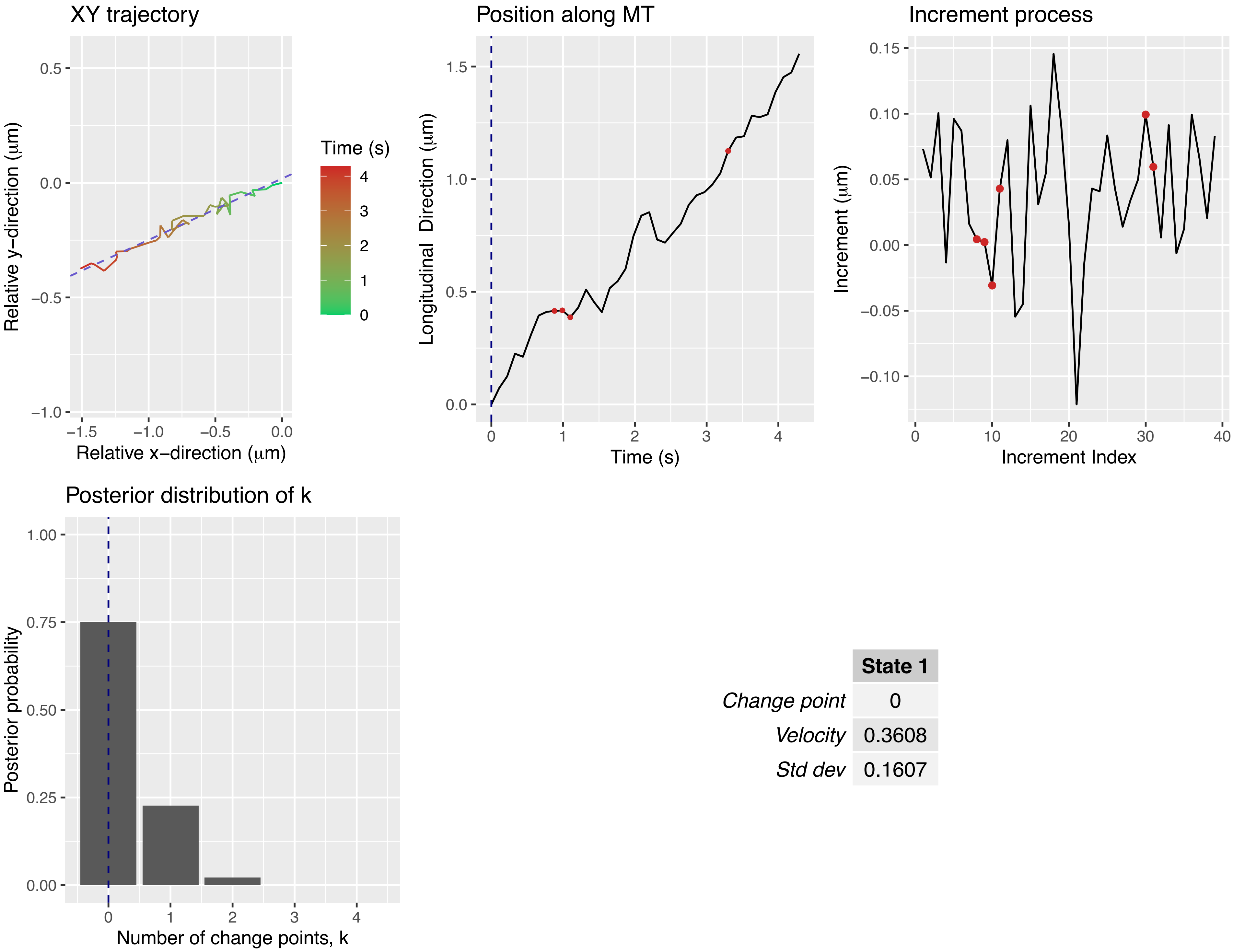

	State 1
Change point	0
Velocity	0.3608
Std dev	0.1607

Kin1DDB particle: 6

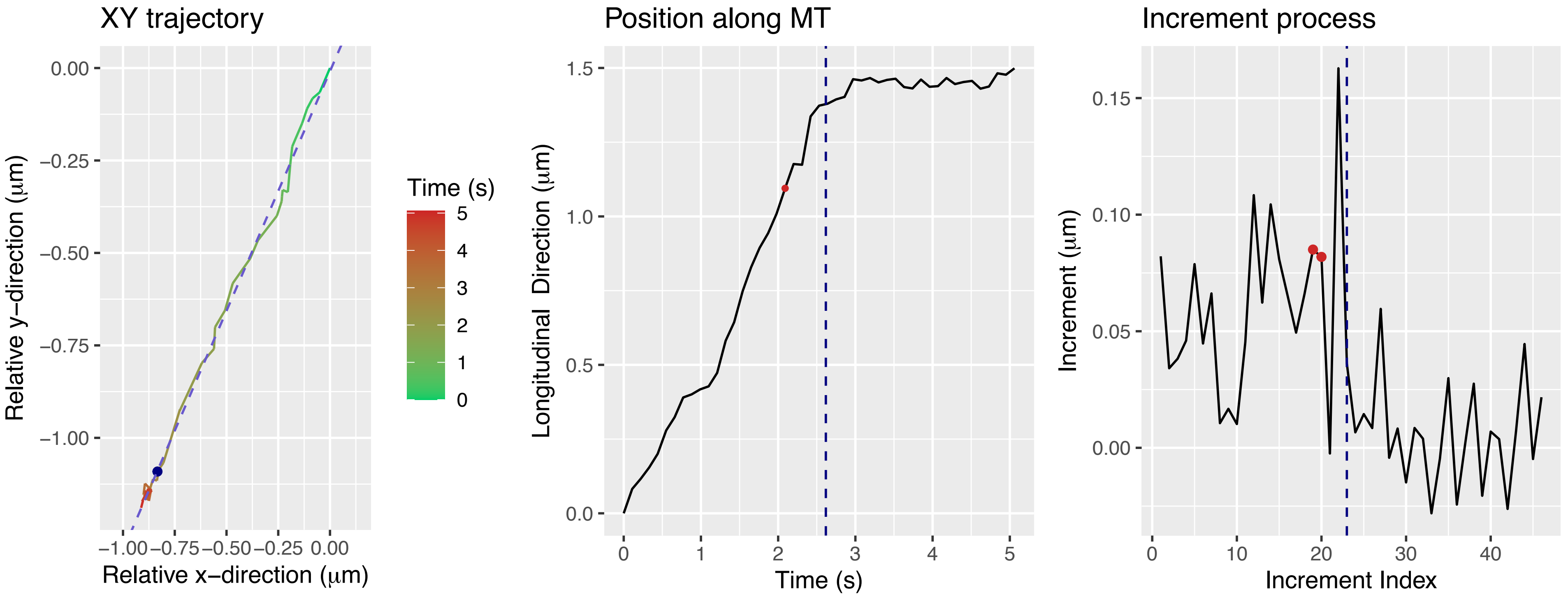

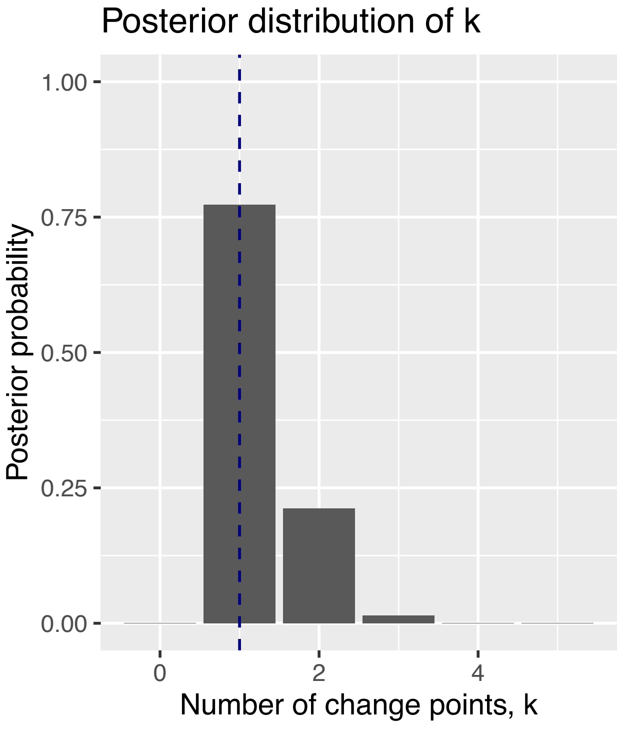

	State 1	State 2
Change point	2.6175	NA
Velocity	0.5411	0.0522
Std dev	0.0941	NA

Kin1DDB particle: 7

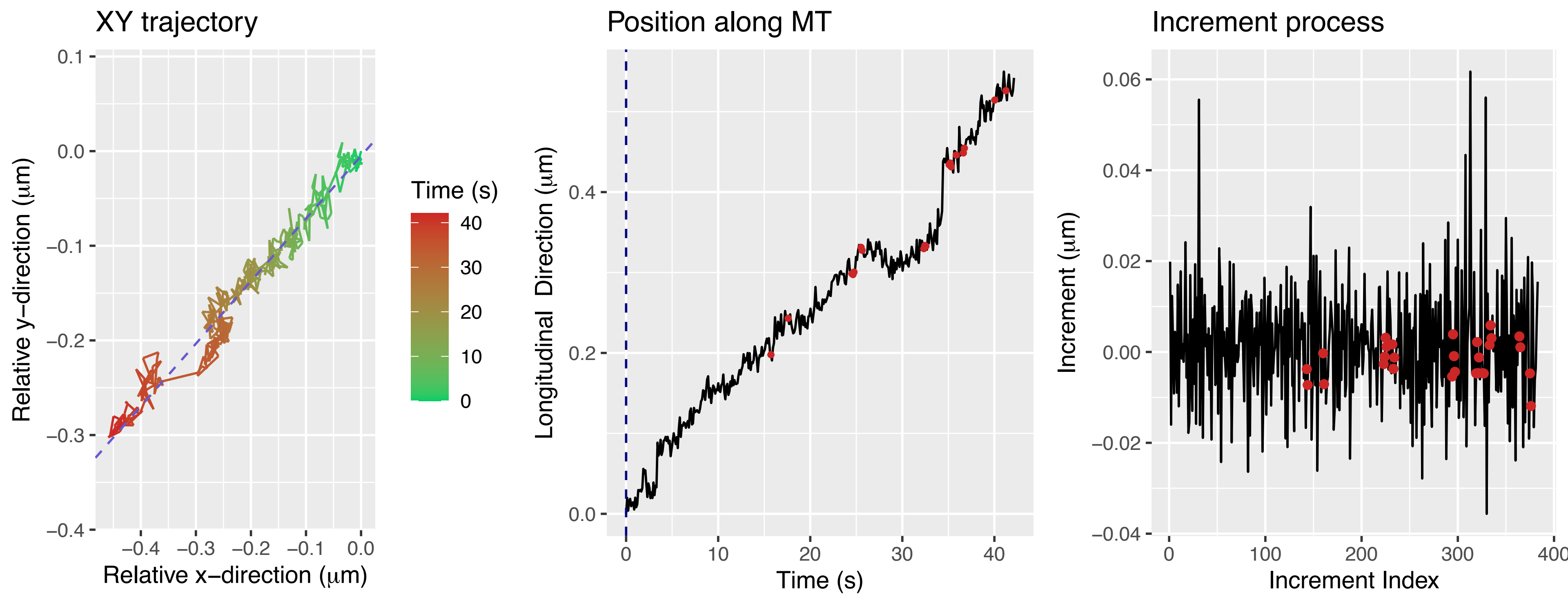

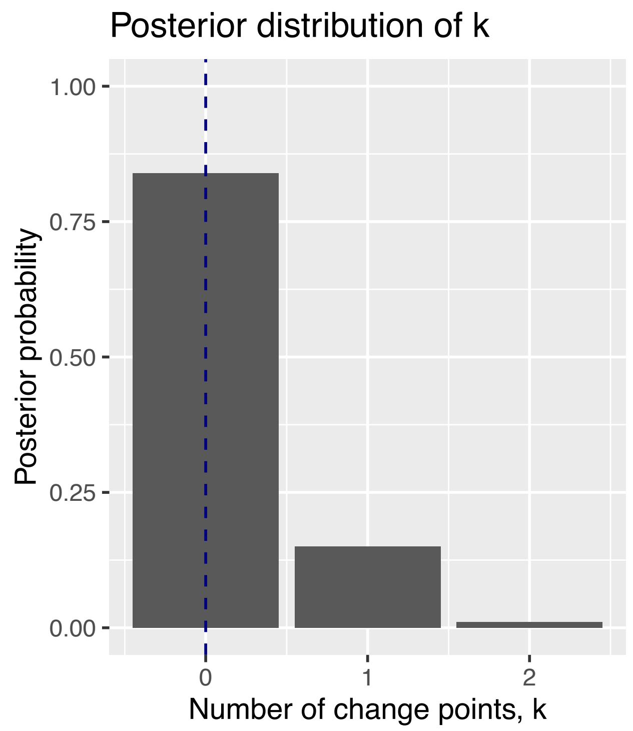

	**State 1**
Change point	0
Velocity	0.0125
Std dev	0.0377

Kin1DDB particle: 8

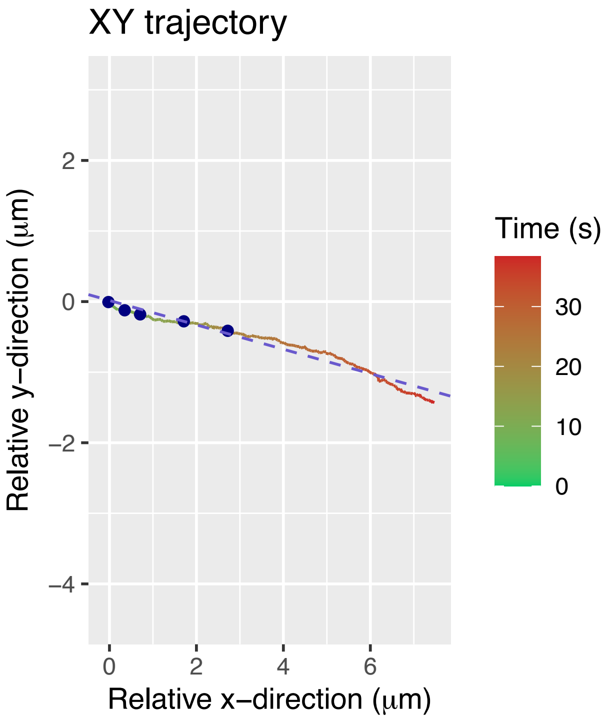

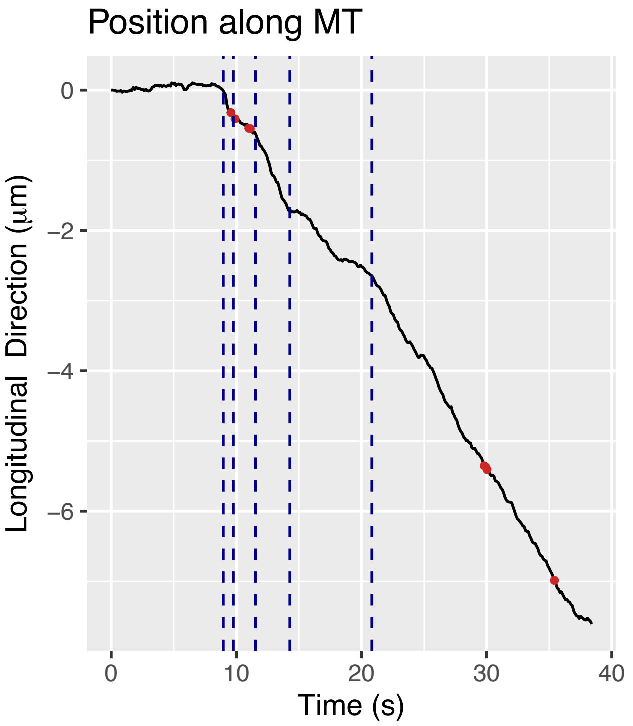

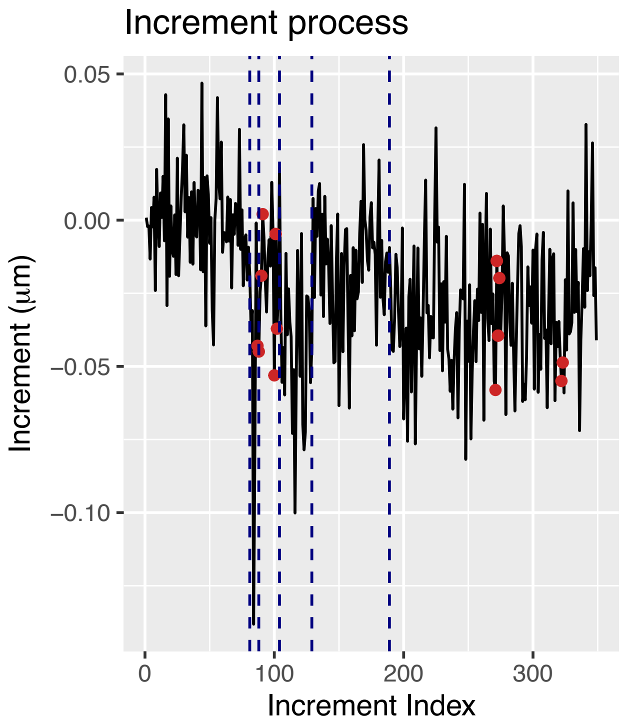

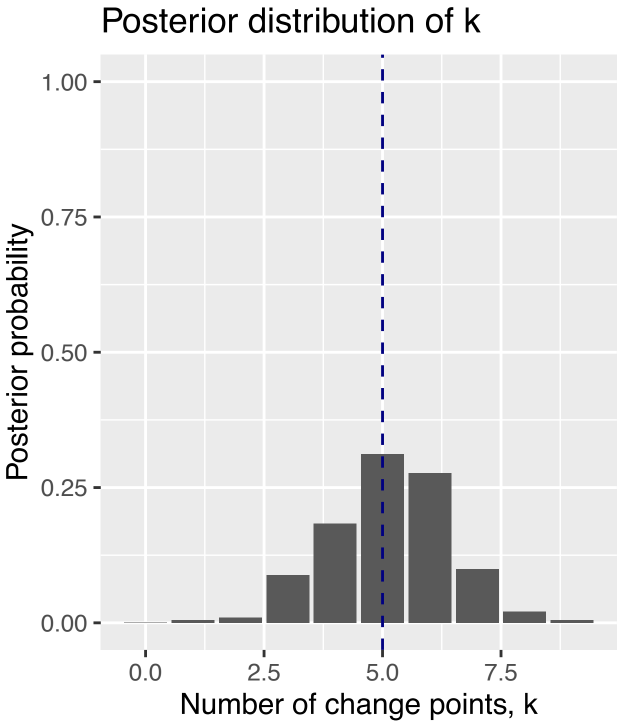

	State 1	State 2	State 3	State 4	State 5	State 6
Change point	8.9479	9.7528	11.5171	14.2766	20.8306	NA
Velocity	0.0077	−0.4464	−0.1295	−0.3941	−0.1551	−0.2841
Std dev	0.0628	NA	NA	NA	NA	NA

Kin1DDB particle: 9

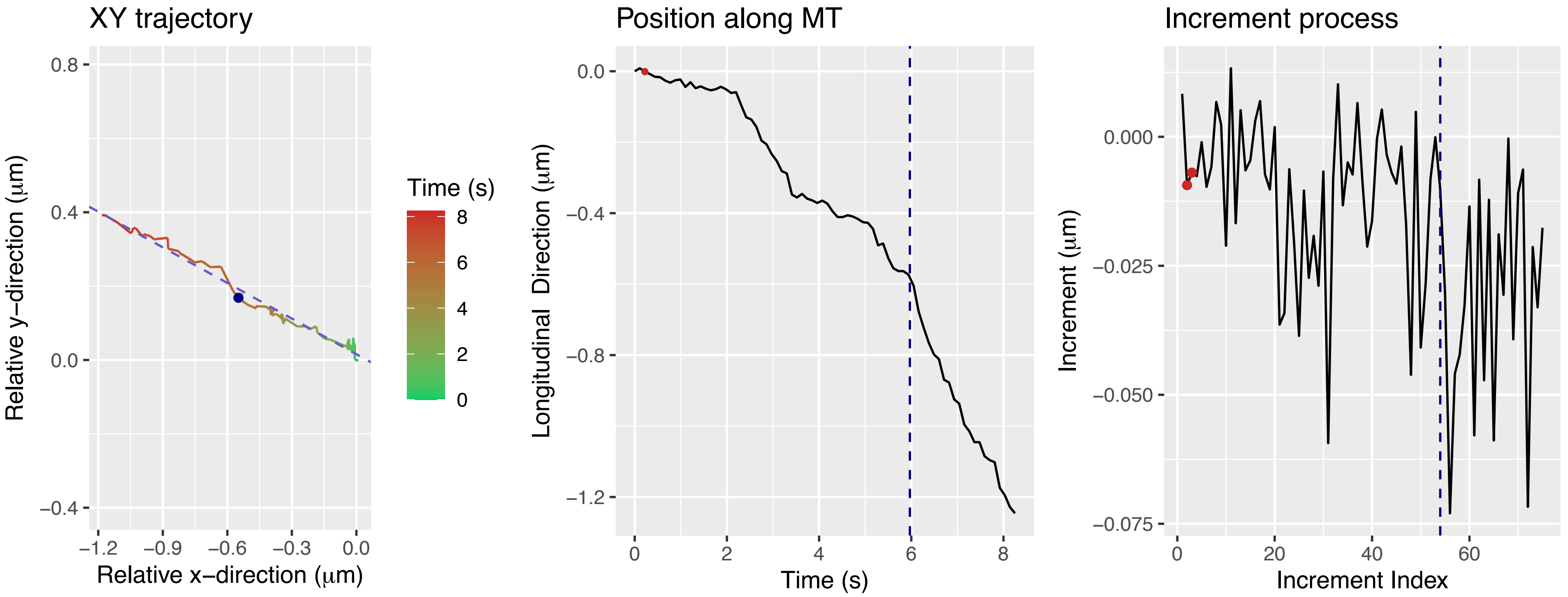

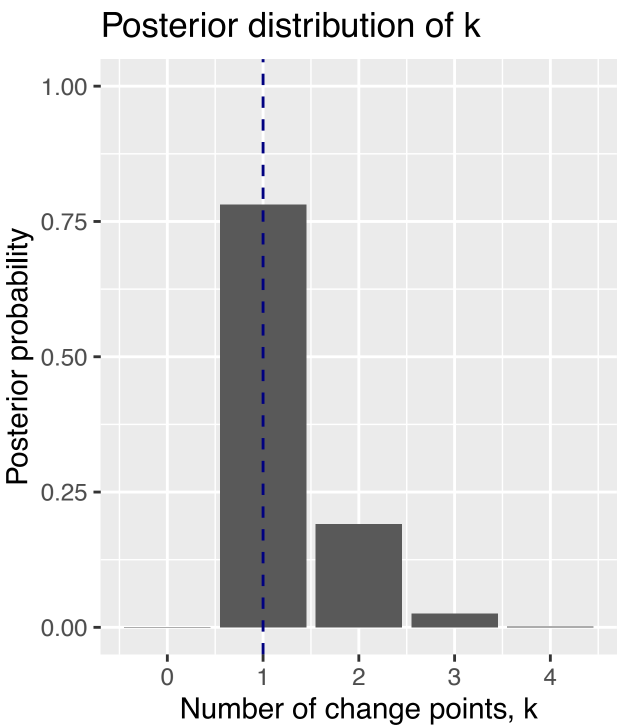

	**State 1**	**State 2**
Change point	5.97	NA
Velocity	−0.0988	−0.2934
Std dev	0.051	NA

Kin1DDB particle: 12

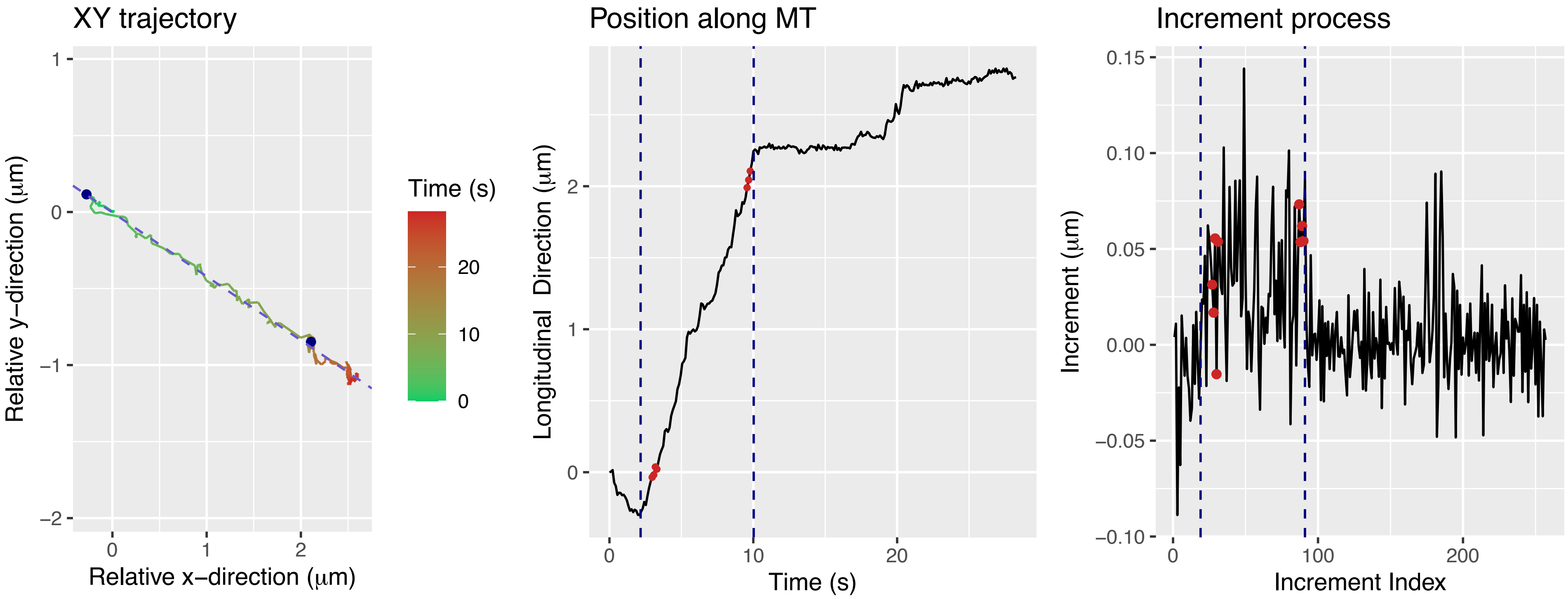

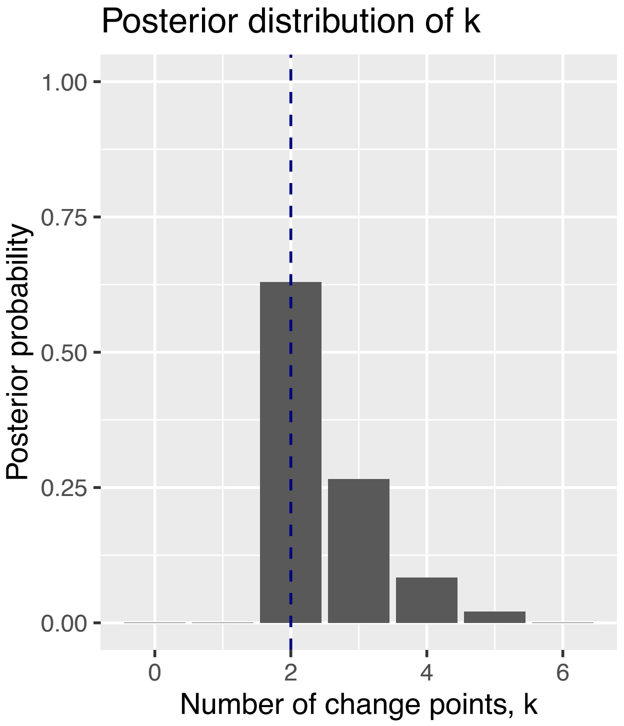

	State 1	State 2	State 3
Change point	2.1662	10.0277	NA
Velocity	−0.1429	0.3208	0.0264
Std dev	0.0807	NA	NA

Kin1DDB particle: 13

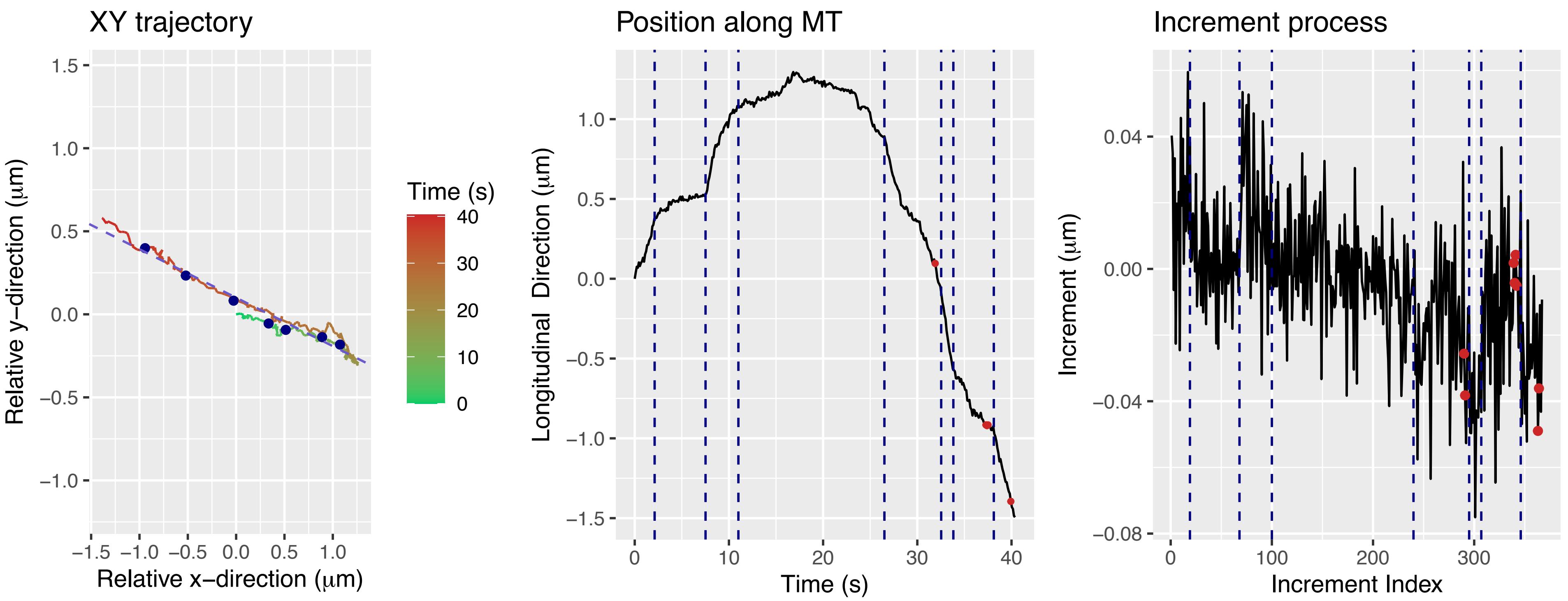

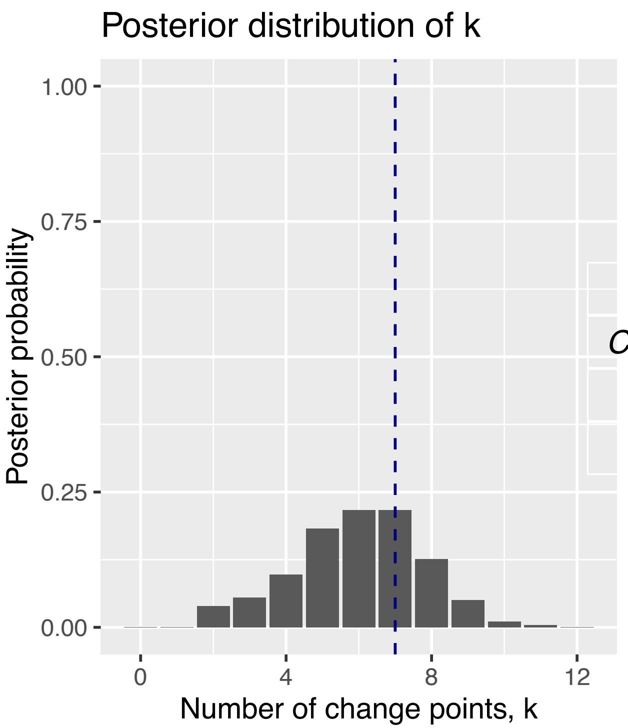

	State 1	State 2	State 3	State 4	State 5	State 6	State 7	State
Change point	2.1115	7.5181	11.0038	26.5057	32.5367	33.8257	38.1211	NA
Velocity	0.1835	0.0441	0.1582	−0.0016	−0.134	−0.4492	−0.047	−0.25[illegible]
Std dev	0.0511	NA	NA	NA	NA	NA	NA	NA

Kin1DDB particle: 14

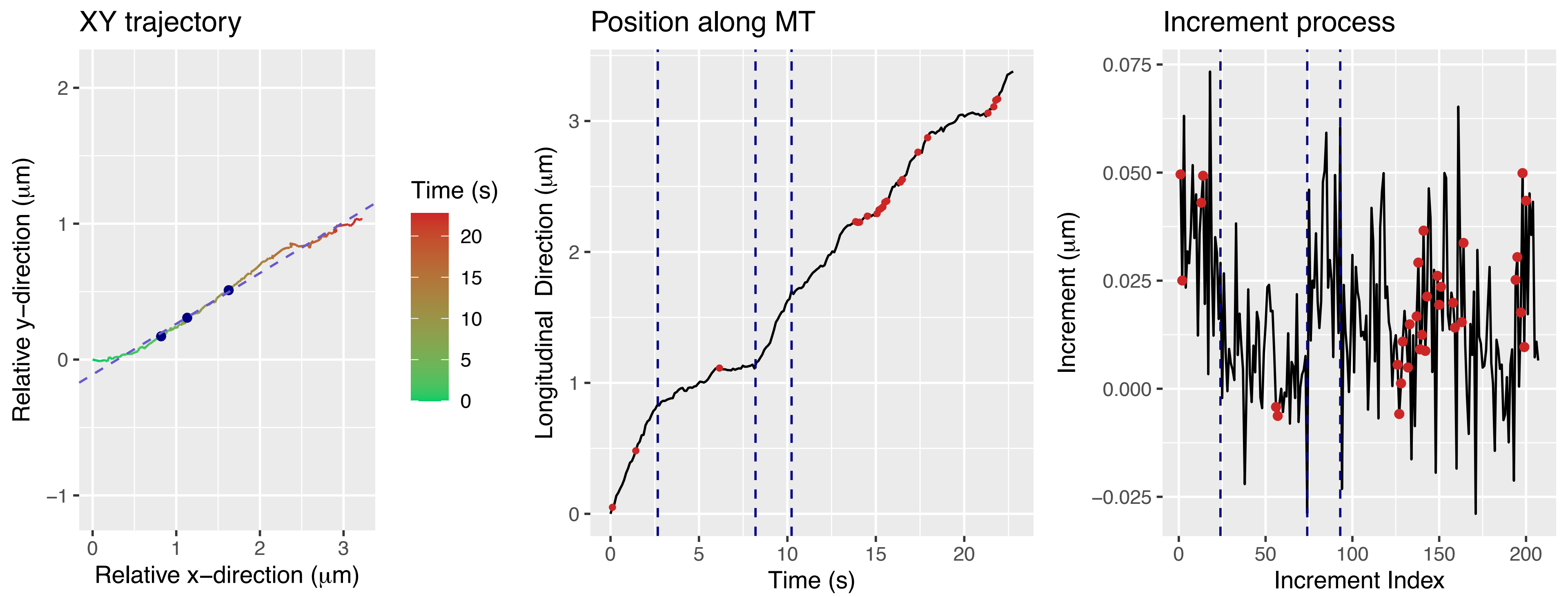

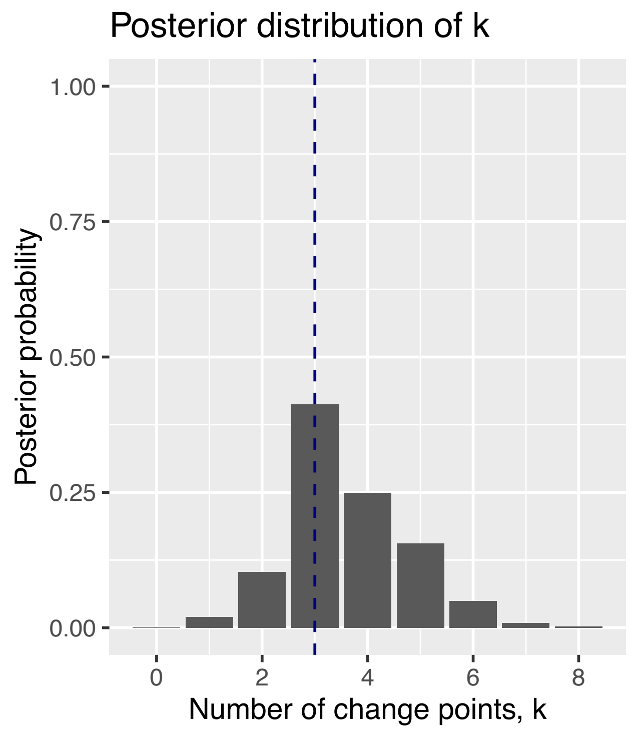

	State 1	State 2	State 3	State 4
Change point	2.6719	8.1973	10.237	NA
Velocity	0.3183	0.0619	0.2883	0.1254
Std dev	0.049	NA	NA	NA

Kin1DDB particle: 15

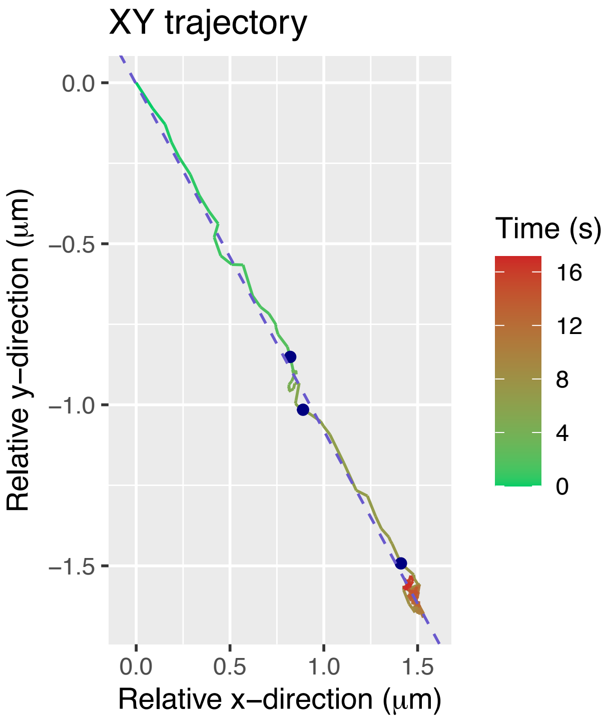

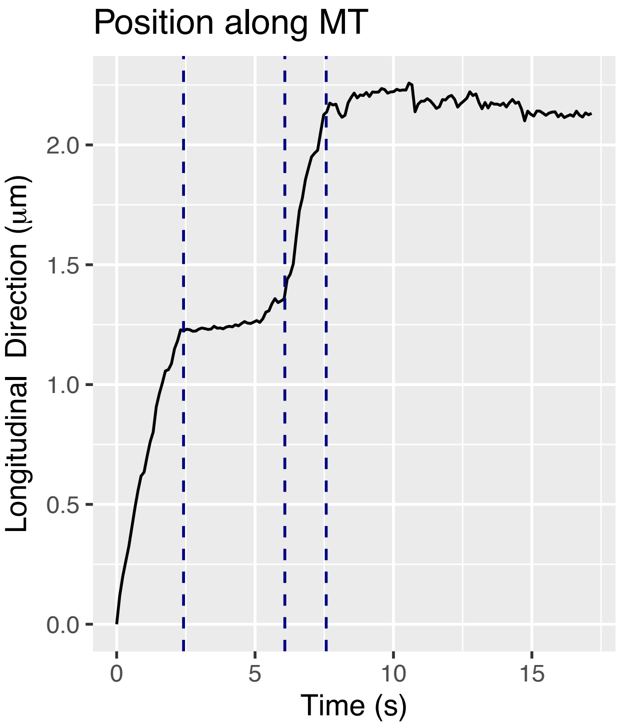

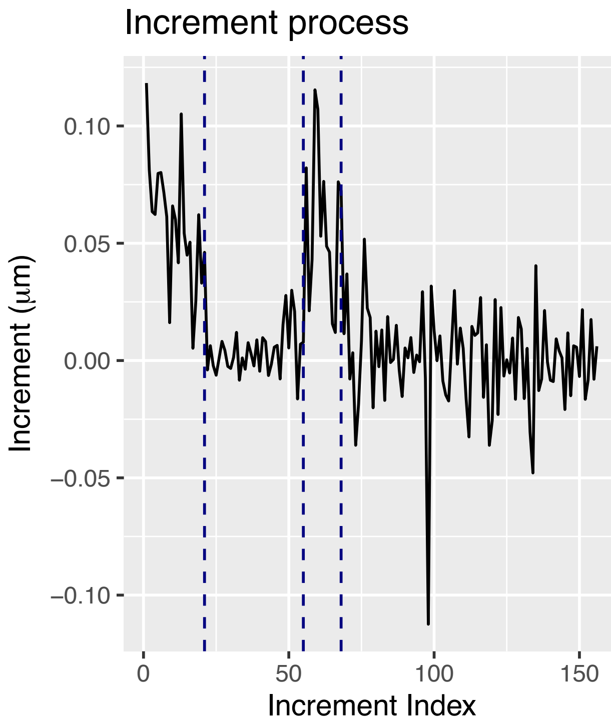

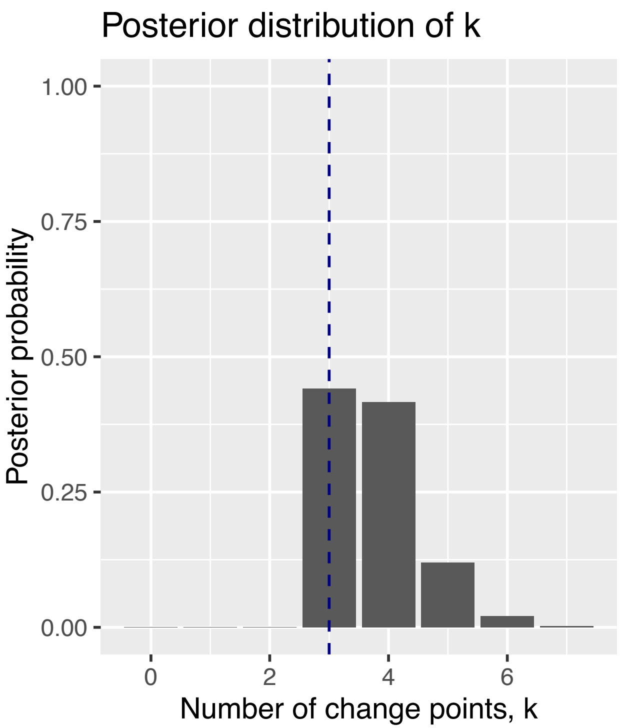

	State 1	State 2	State 3	State 4
Change point	2.4156	6.0754	7.5705	NA
Velocity	0.5486	0.044	0.5538	0.0025
Std dev	0.0664	NA	NA	NA

Kin1DDB particle: 16

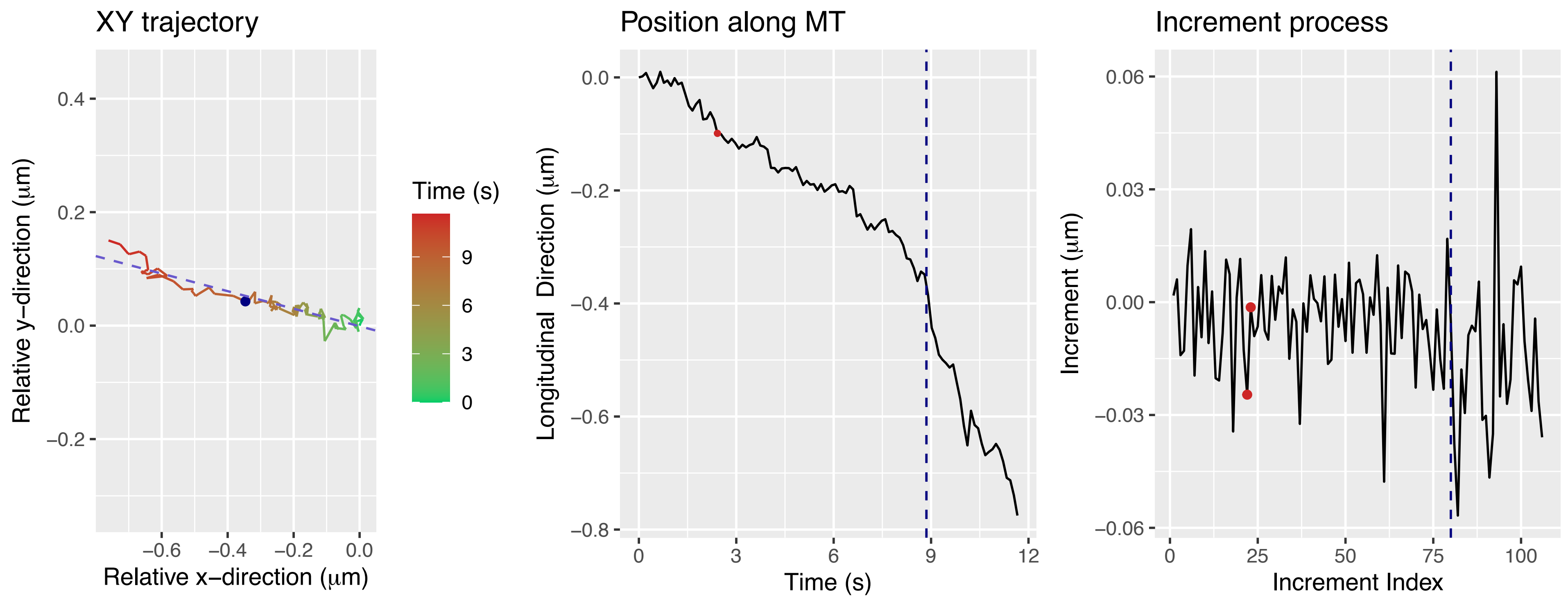

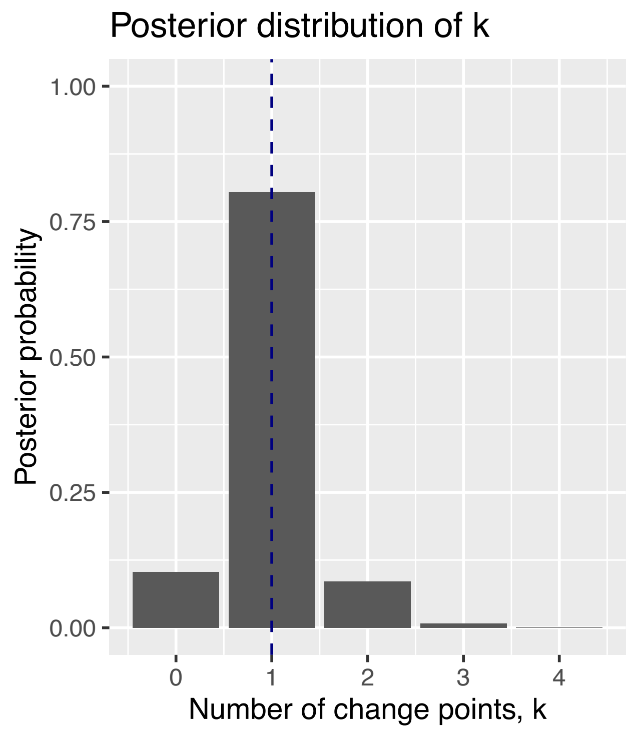

	State 1	State 2
Change point	8.8566	NA
Velocity	−0.0363	−0.1466
Std dev	0.0463	NA

Kin1DDB particle: 17

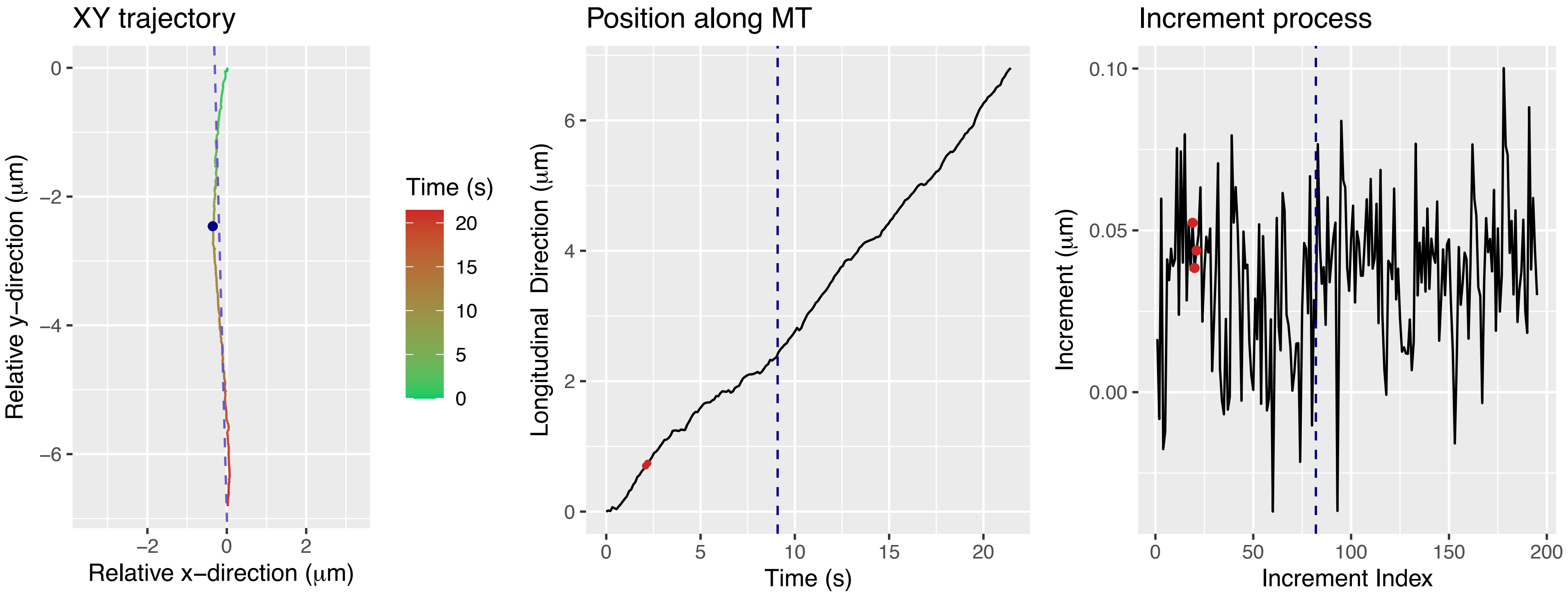

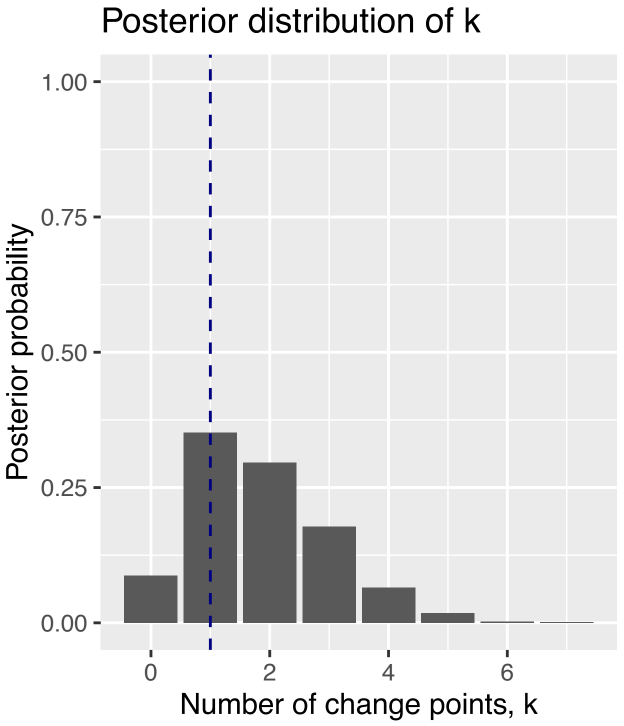

	State 1	State 2
Change point	9.0878	NA
Velocity	0.2625	0.3521
Std dev	0.0686	NA

Kin1DDB particle: 18

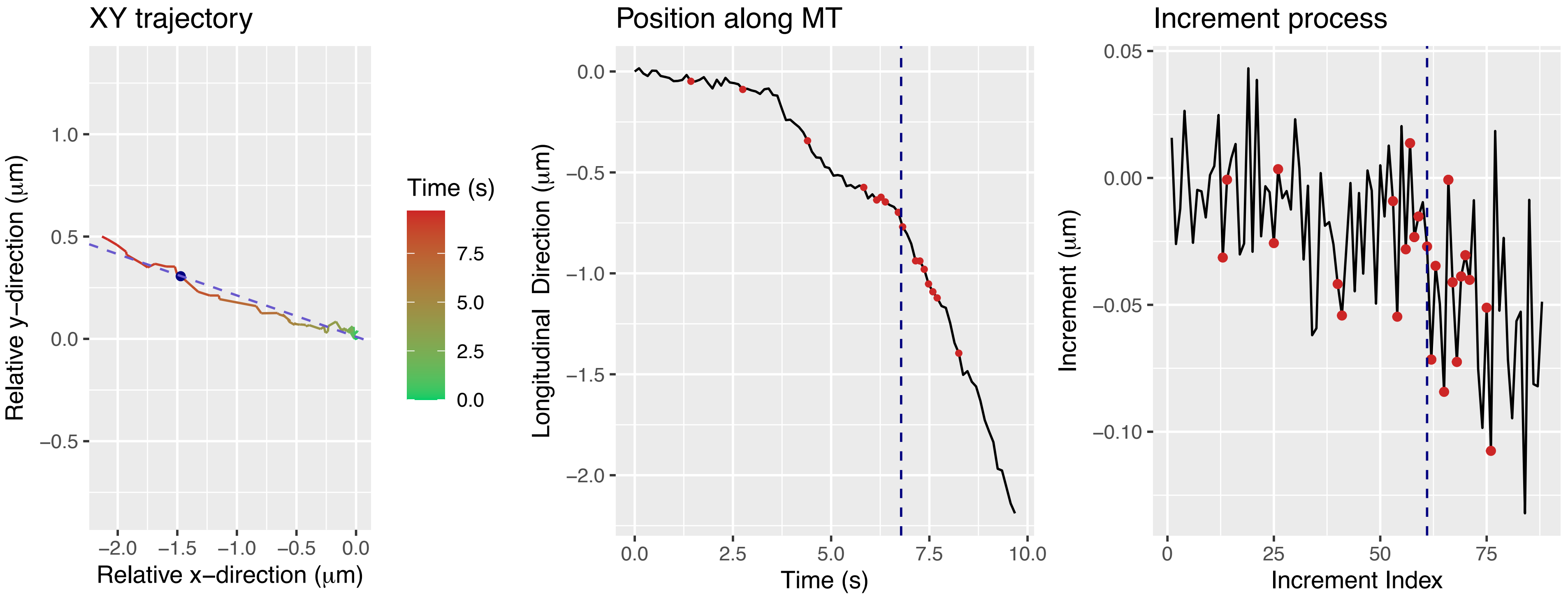

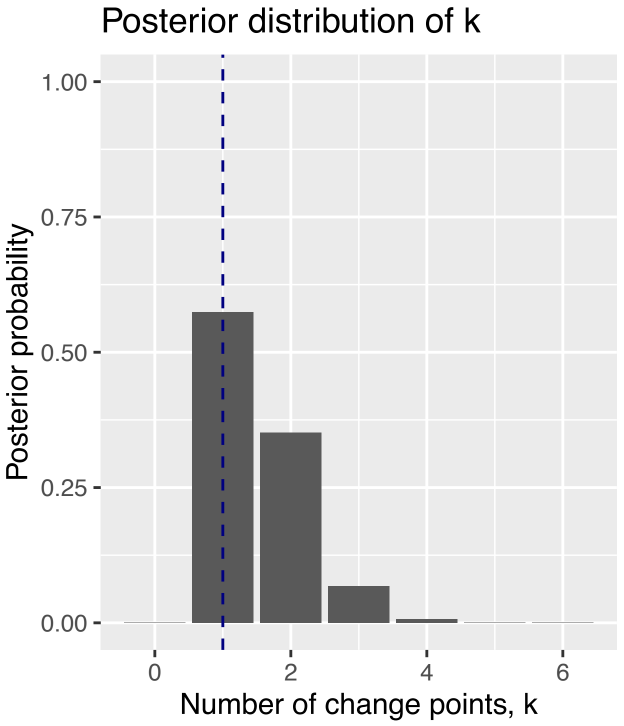

	State 1	State 2
Change point	6.782	NA
Velocity	−0.1072	−0.5029
Std dev	0.0817	NA

Kin1DDB particle: 19

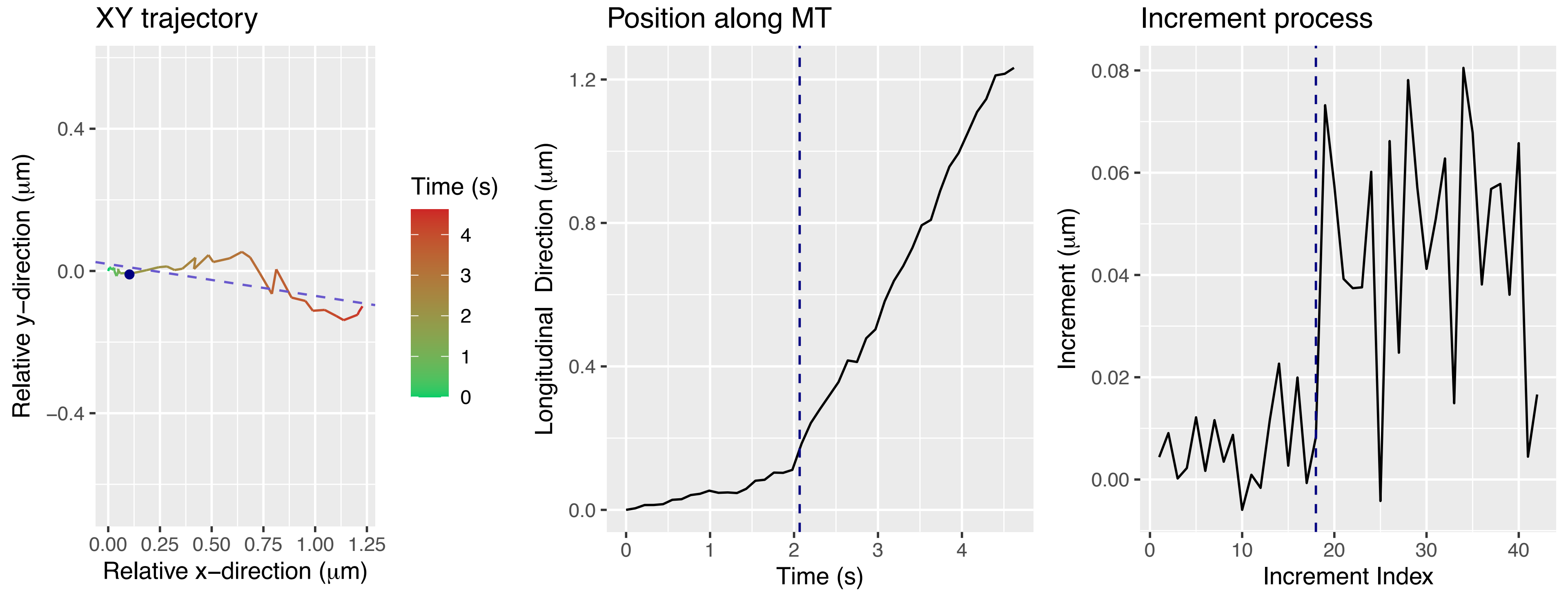

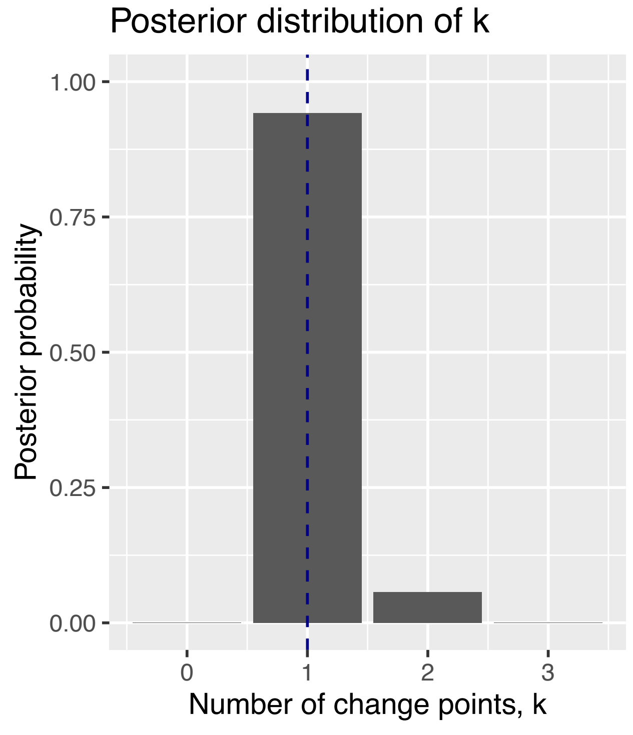

	State 1	State 2
Change point	2.0672	NA
Velocity	0.0552	0.4261
Std dev	0.0585	NA

Kin1DDB particle: 20

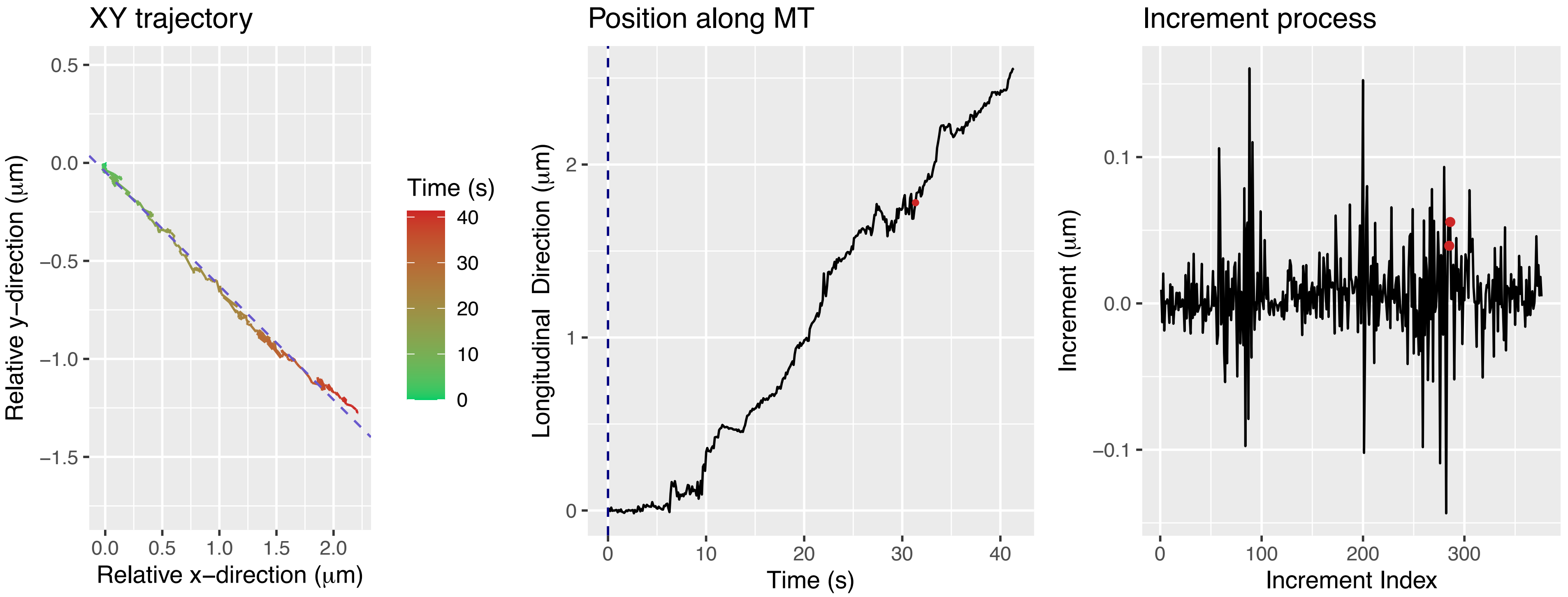

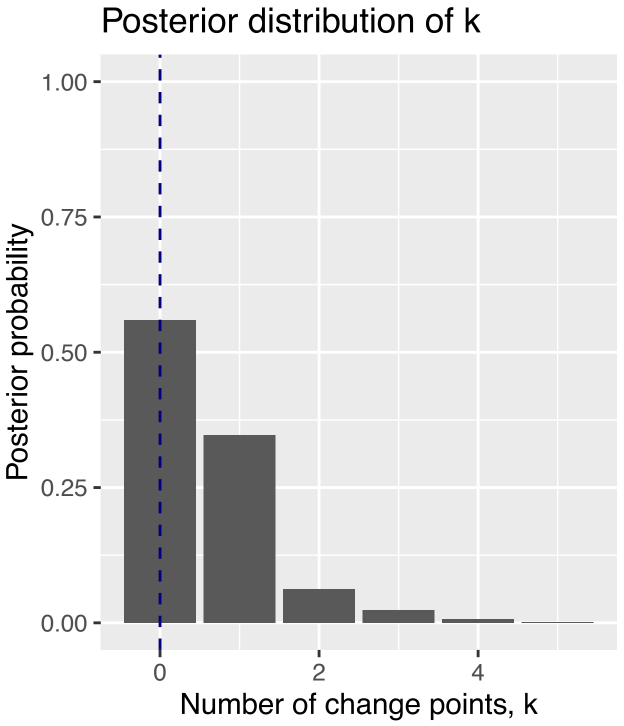

	**State 1**
Change point	0
Velocity	0.0622
Std dev	0.091

Kin1DDB particle: 21

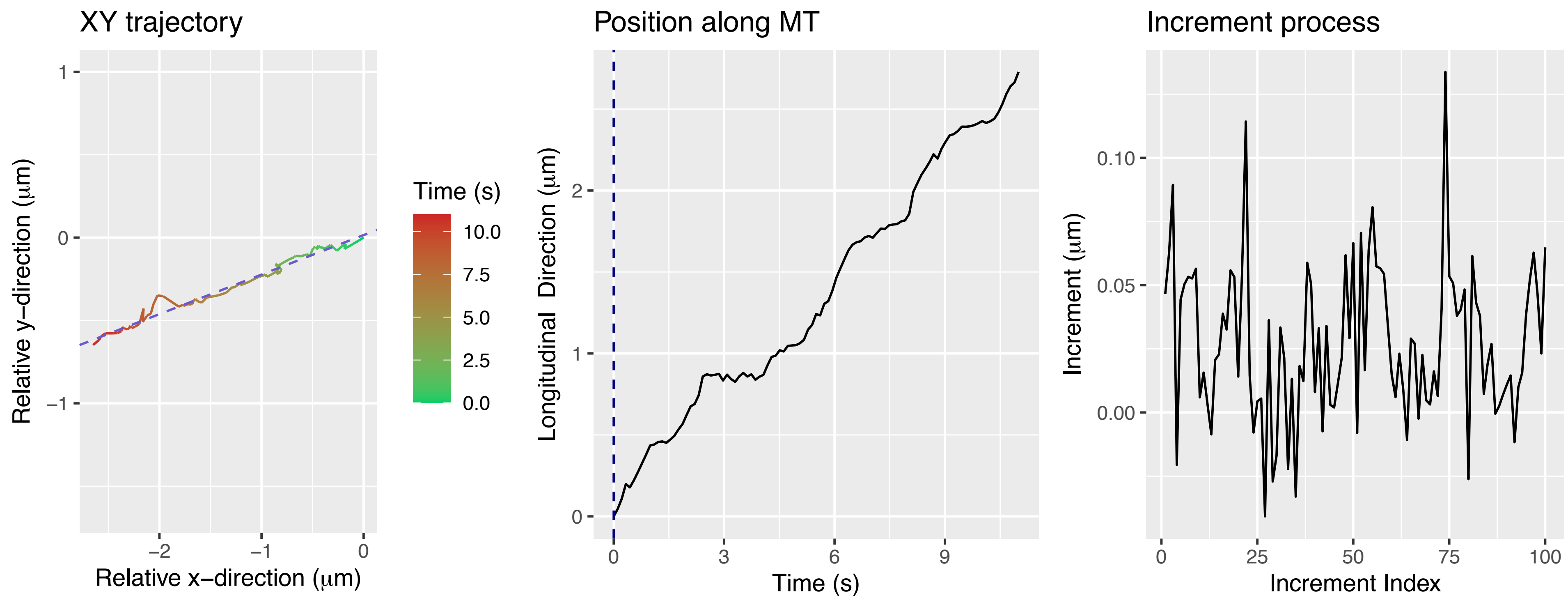

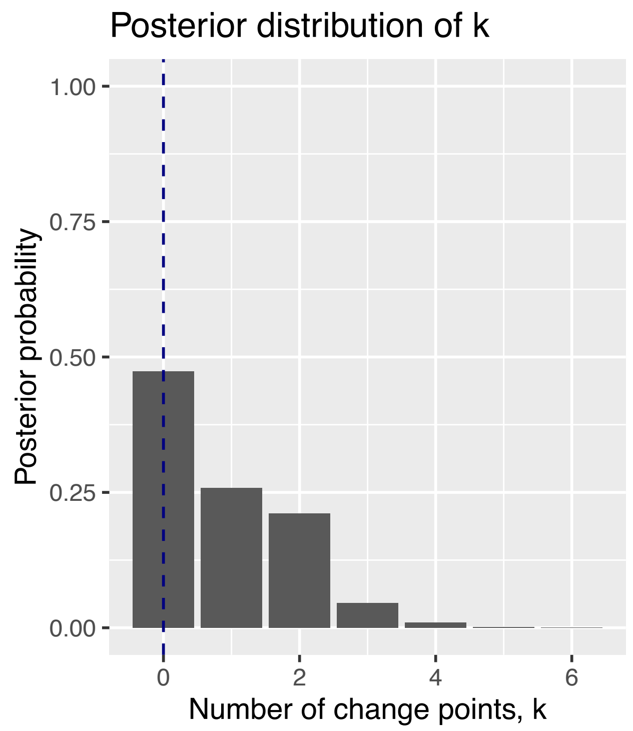

	State 1
Change point	0
Velocity	0.2482
Std dev	0.0904

Kin1DDB particle: 22

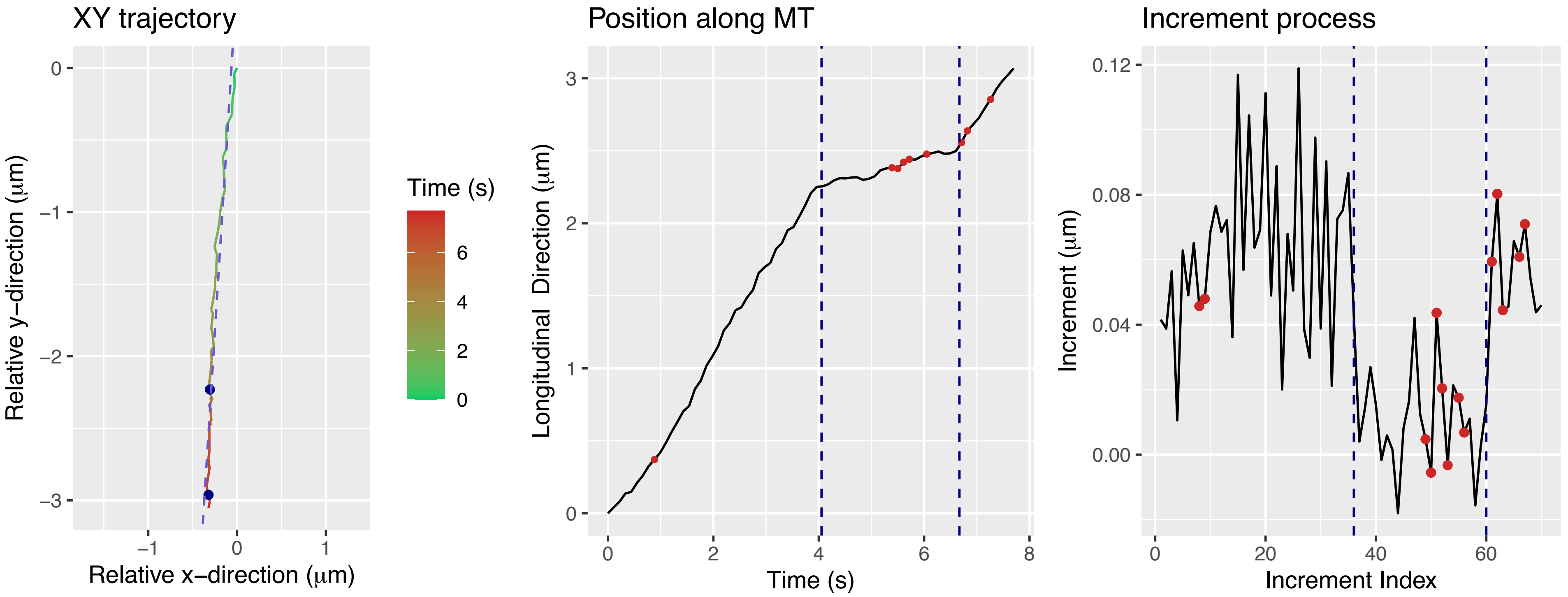

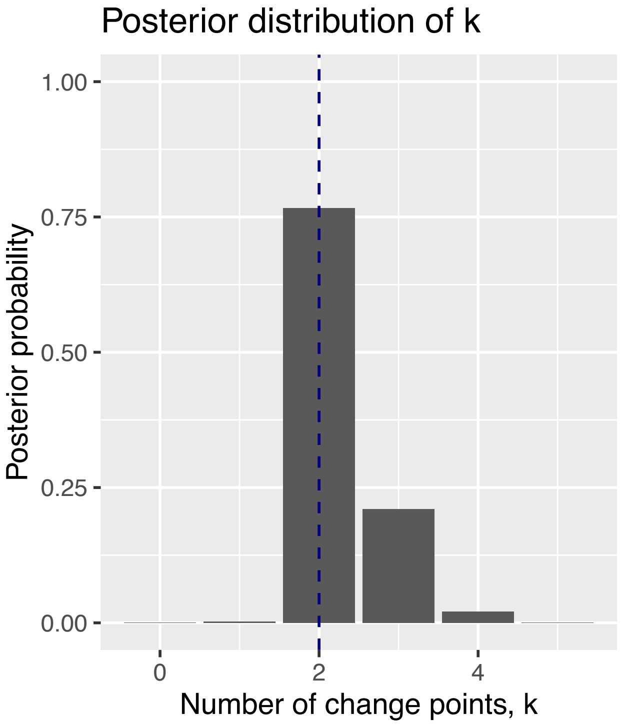

	State 1	State 2	State 3
Change point	4.053	6.6698	NA
Velocity	0.5652	0.095	0.5119
Std dev	0.0698	NA	NA

Kin1DDB particle: 23

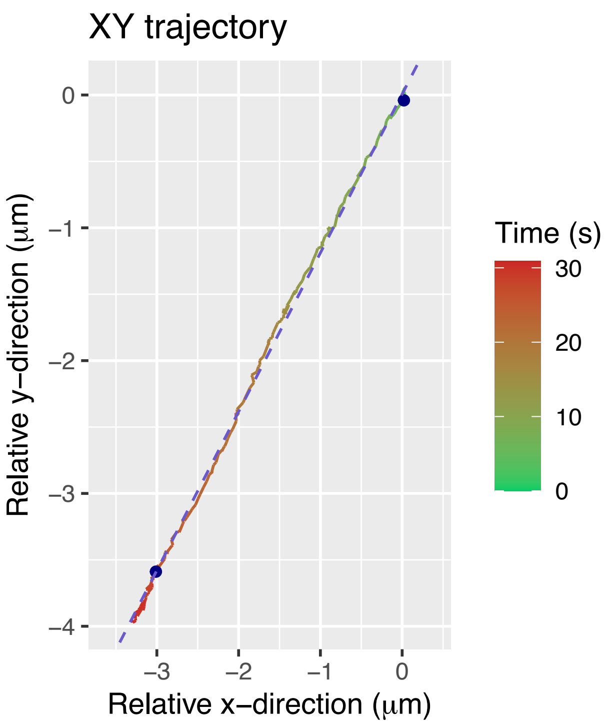

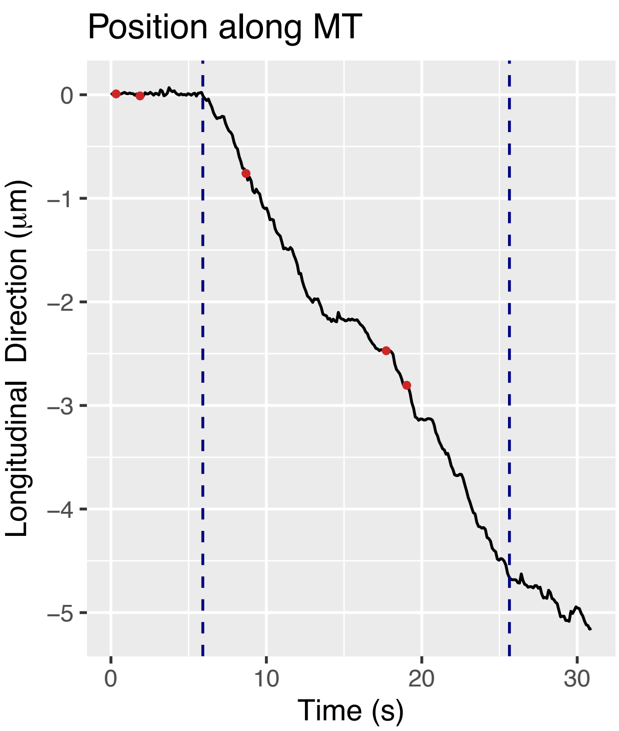

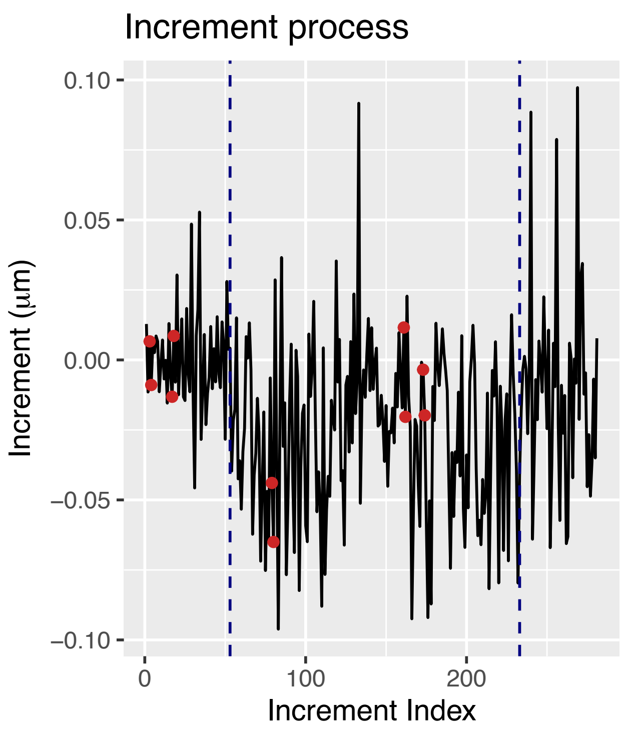

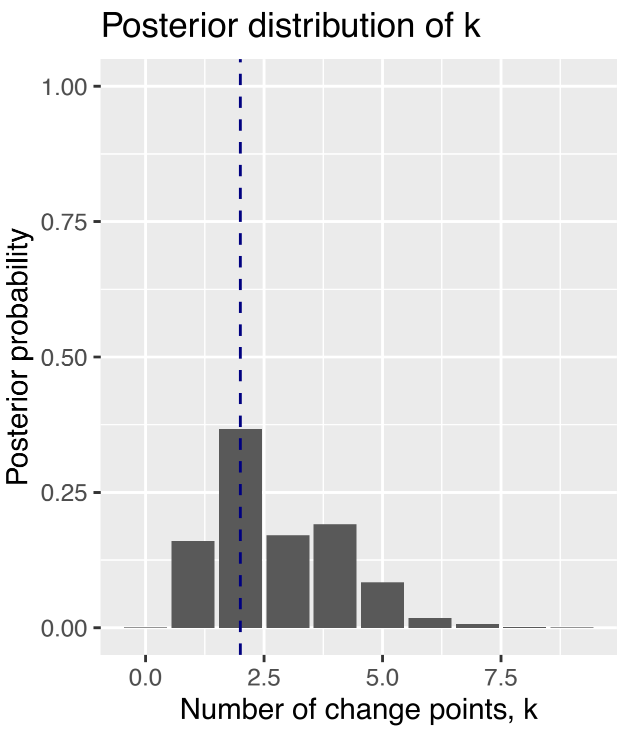

	State 1	State 2	State 3
Change point	5.9104	25.6389	NA
Velocity	−0.0051	−0.2254	−0.0776
Std dev	0.0885	NA	NA

Kin1DDB particle: 24

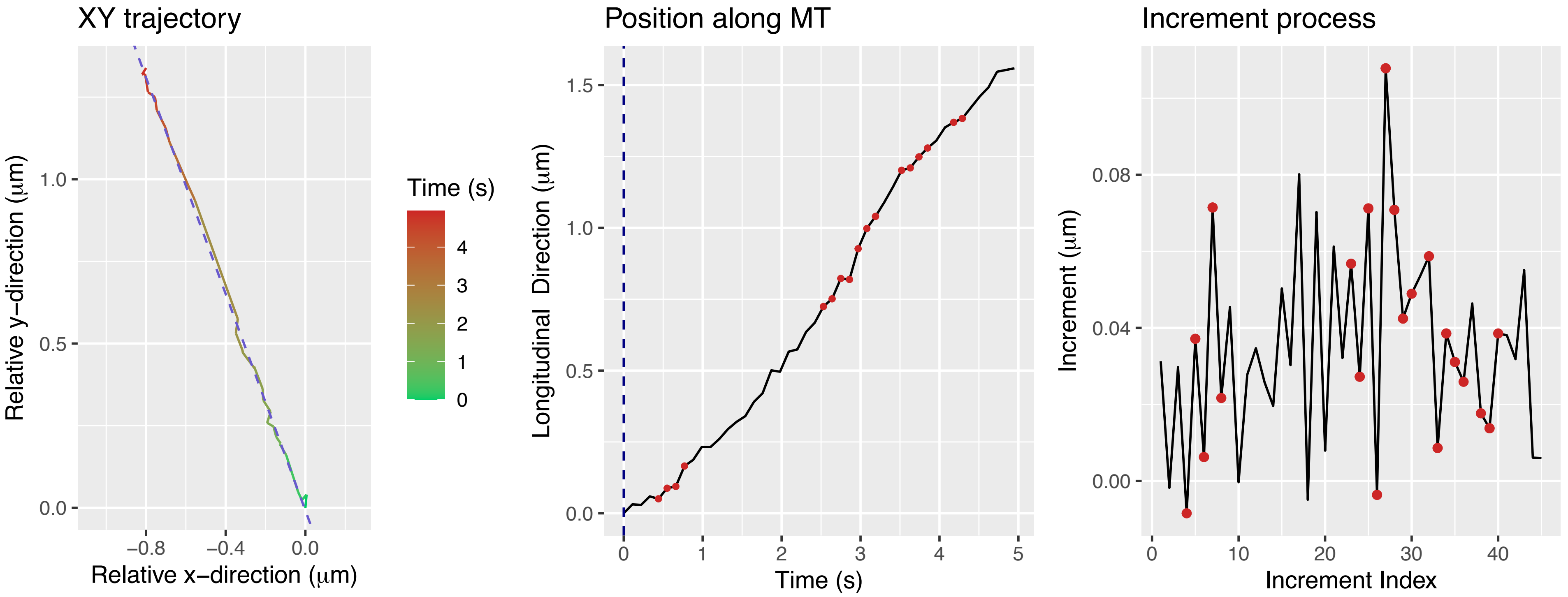

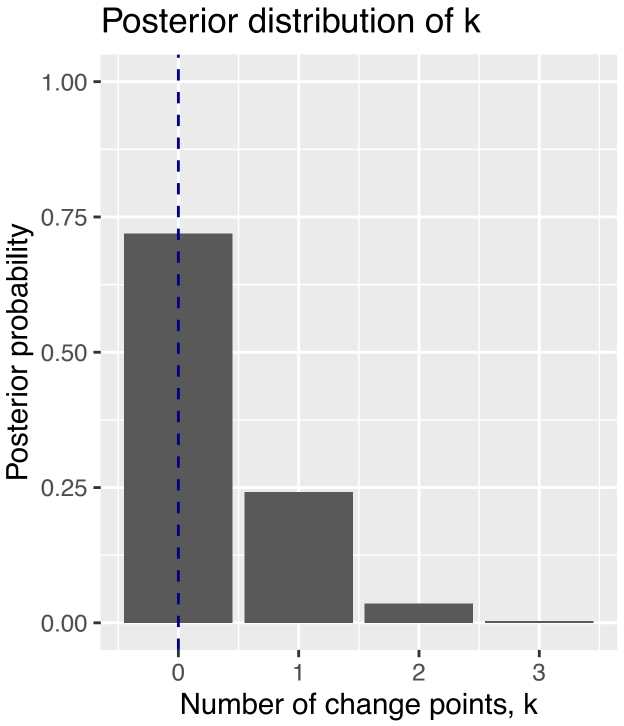

	State 1
Change point	0
Velocity	0.3151
Std dev	0.0743

Kin1DDB particle: 25

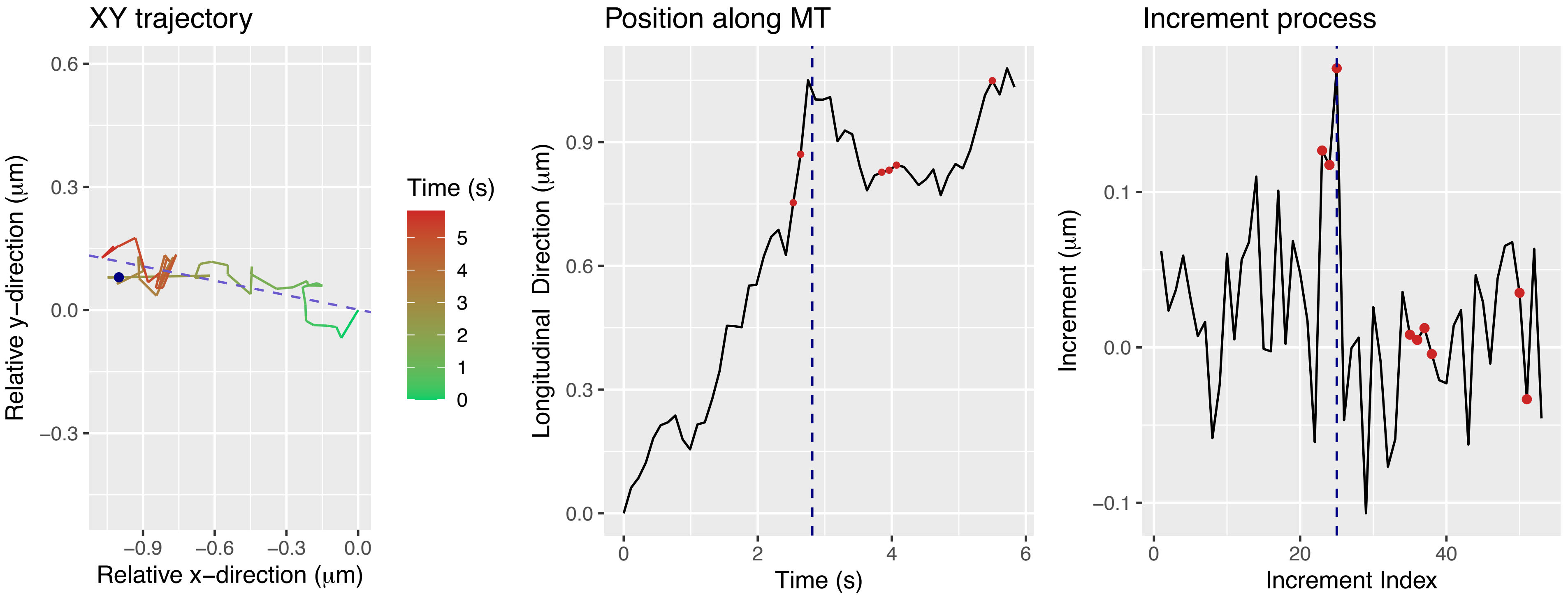

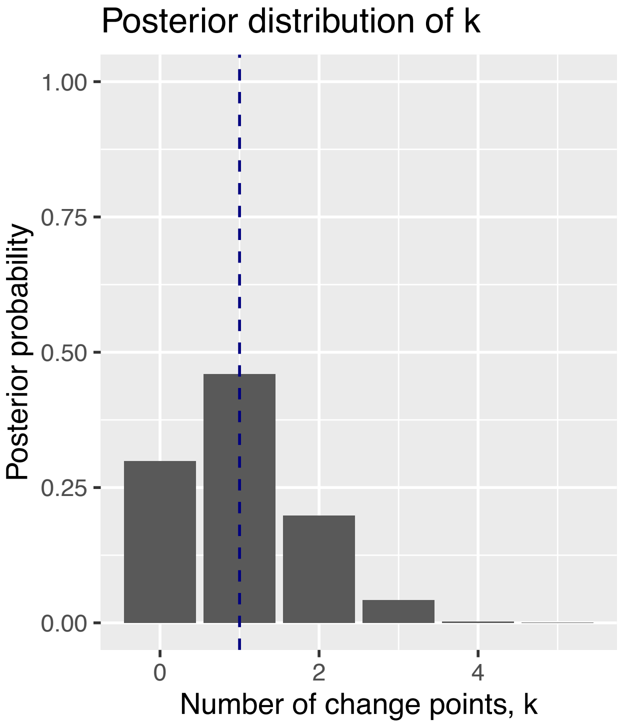

	State 1	State 2
Change point	2.8128	NA
Velocity	0.3822	−0.0047
Std dev	0.1506	NA

Kin1DDB particle: 26

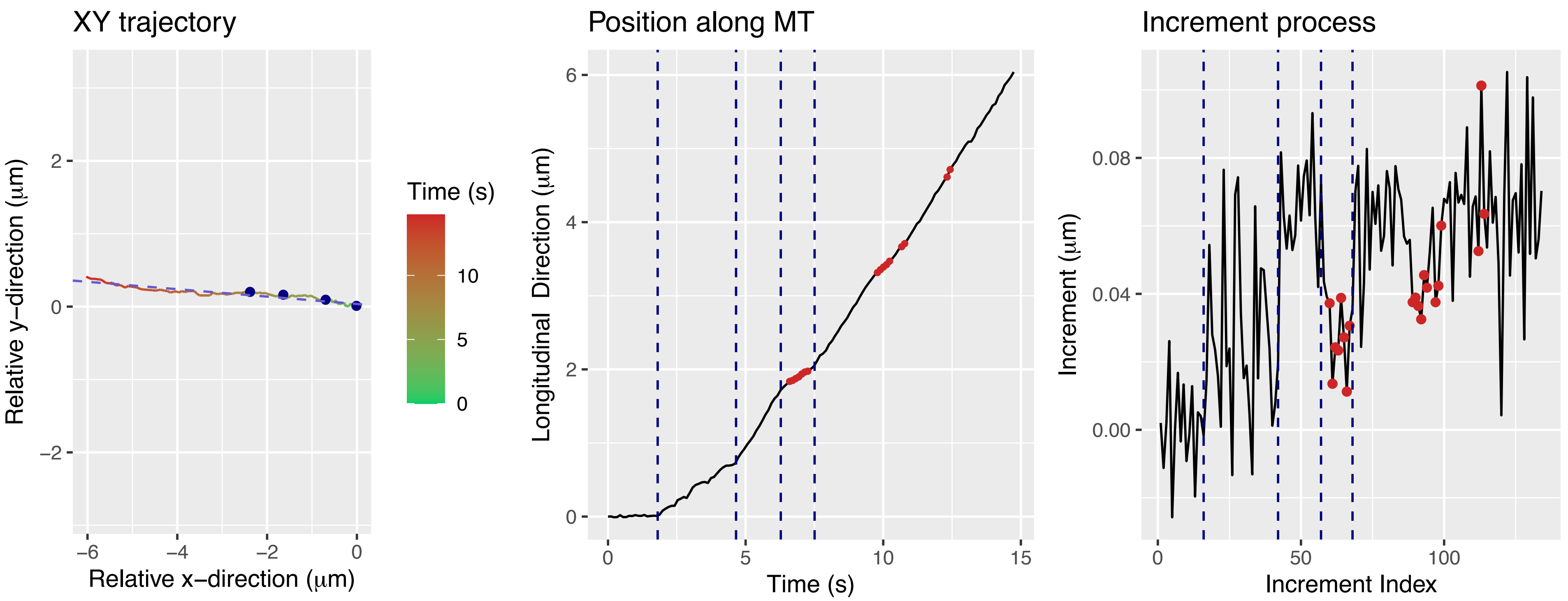

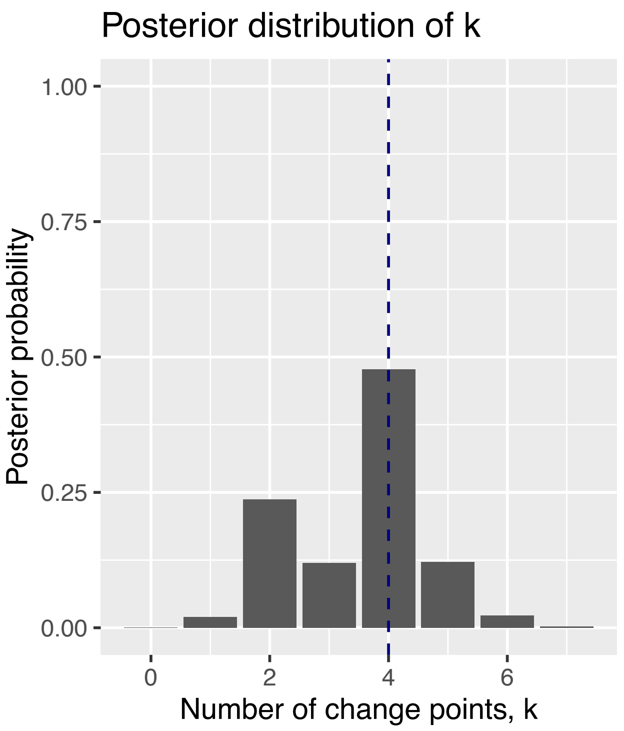

	State 1	State 2	State 3	State 4	State 5
Change point	1.8047	4.6522	6.2778	7.5061	NA
Velocity	−0.0059	0.2461	0.6247	0.2725	0.5531
Std dev	0.0555	NA	NA	NA	NA

Kin1DDB particle: 27

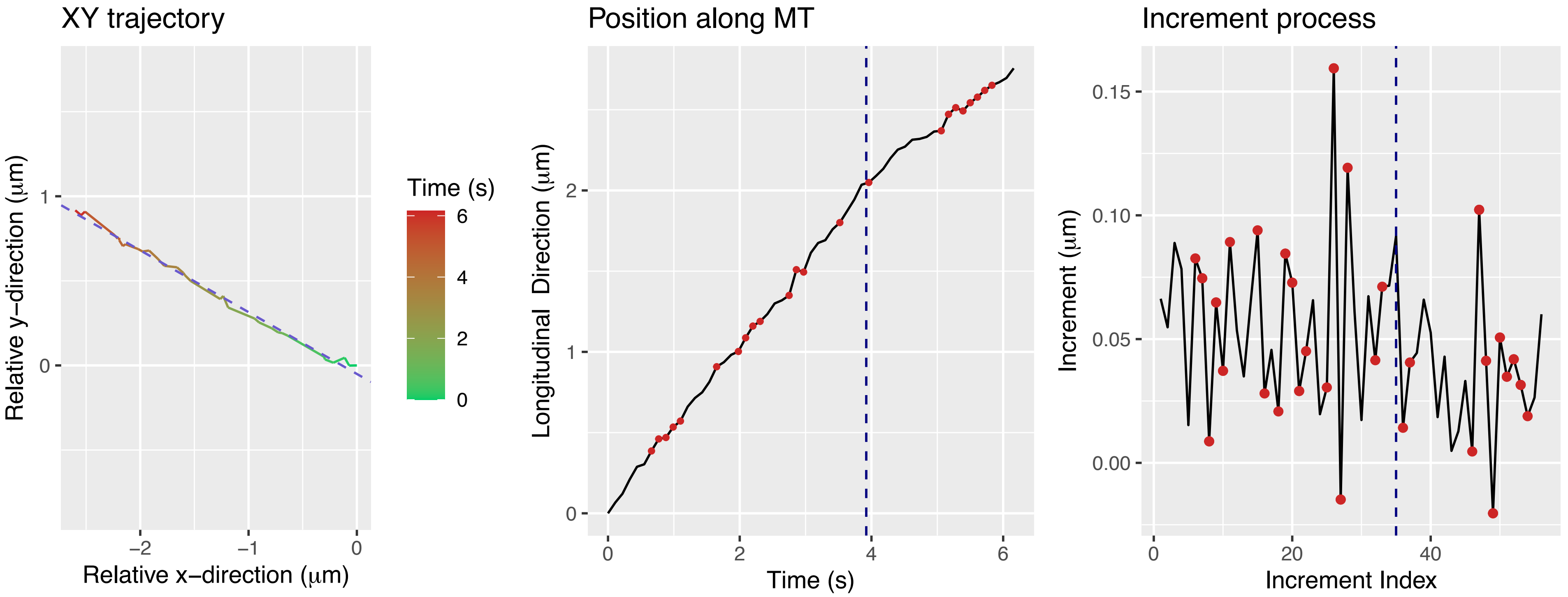

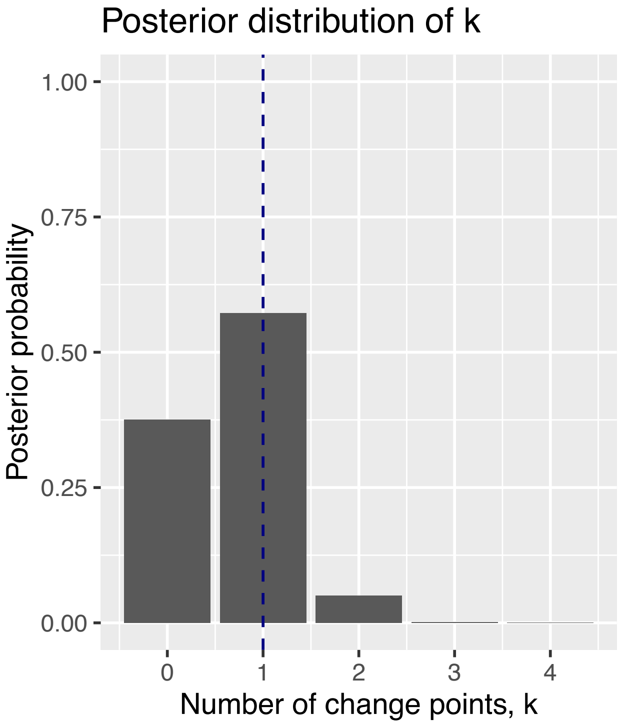

	State 1	State 2
Change point	3.9213	NA
Velocity	0.5195	0.306
Std dev	0.0946	NA

Kin1DDB particle: 28

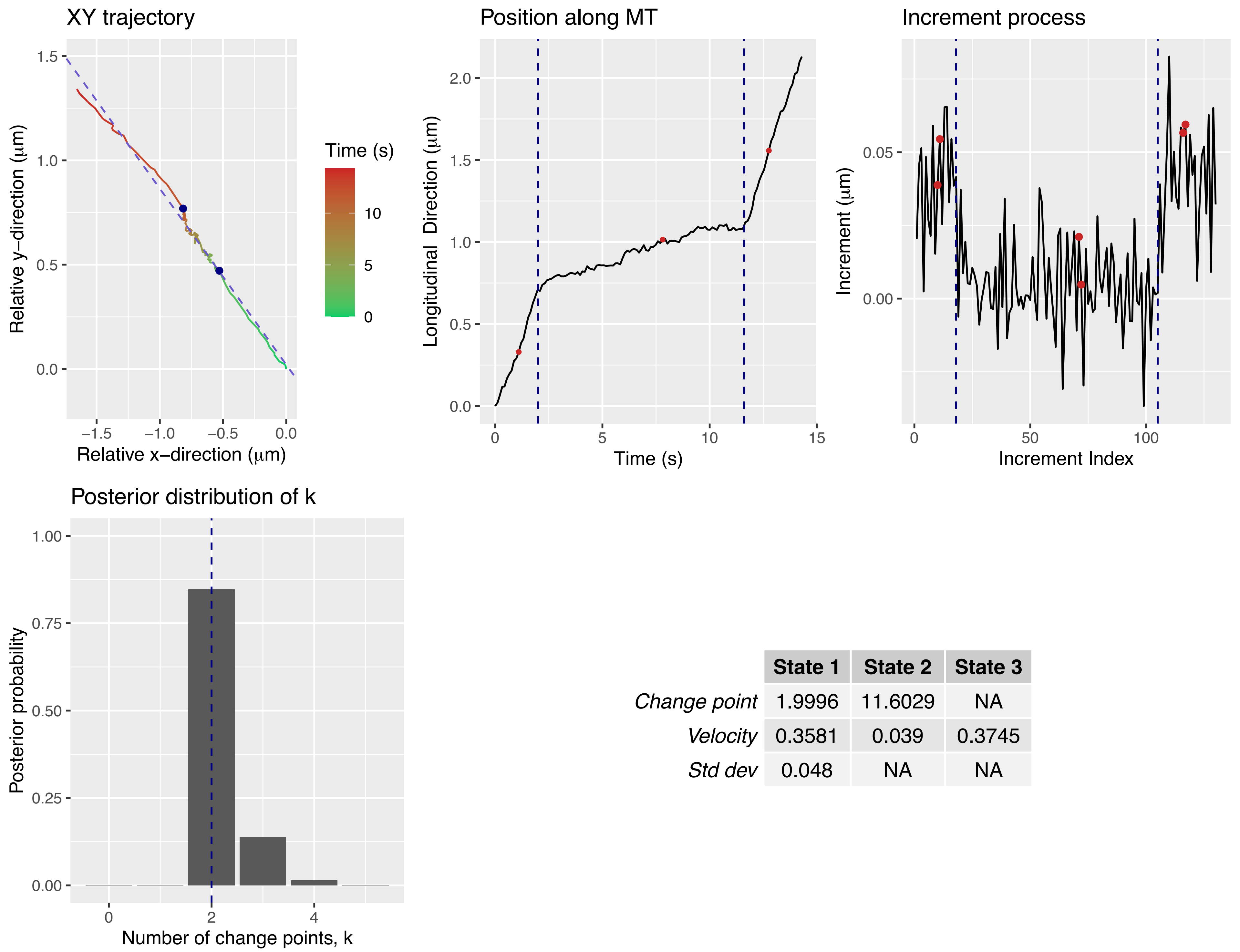

	State 1	State 2	State 3
Change point	1.9996	11.6029	NA
Velocity	0.3581	0.039	0.3745
Std dev	0.048	NA	NA

Kin1DDB particle: 29

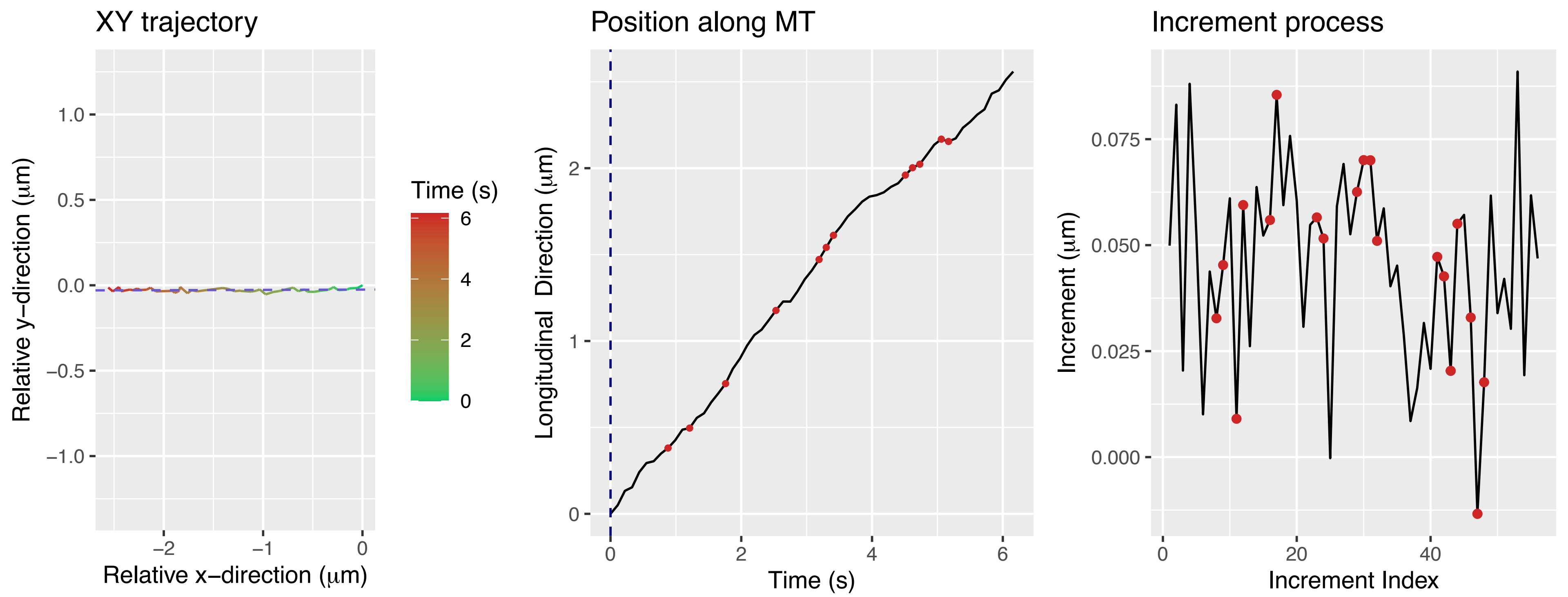

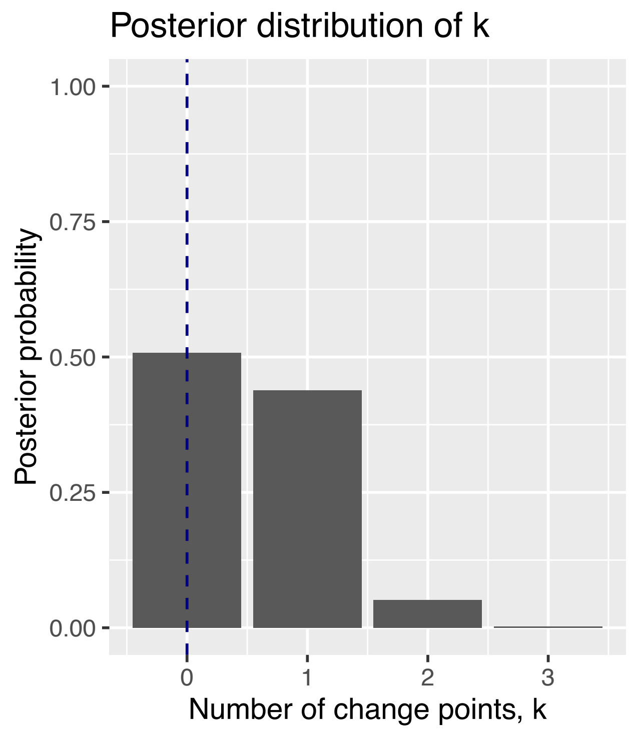

	State 1
Change point	0
Velocity	0.4153
Std dev	0.0657

Kin1DDB particle: 30

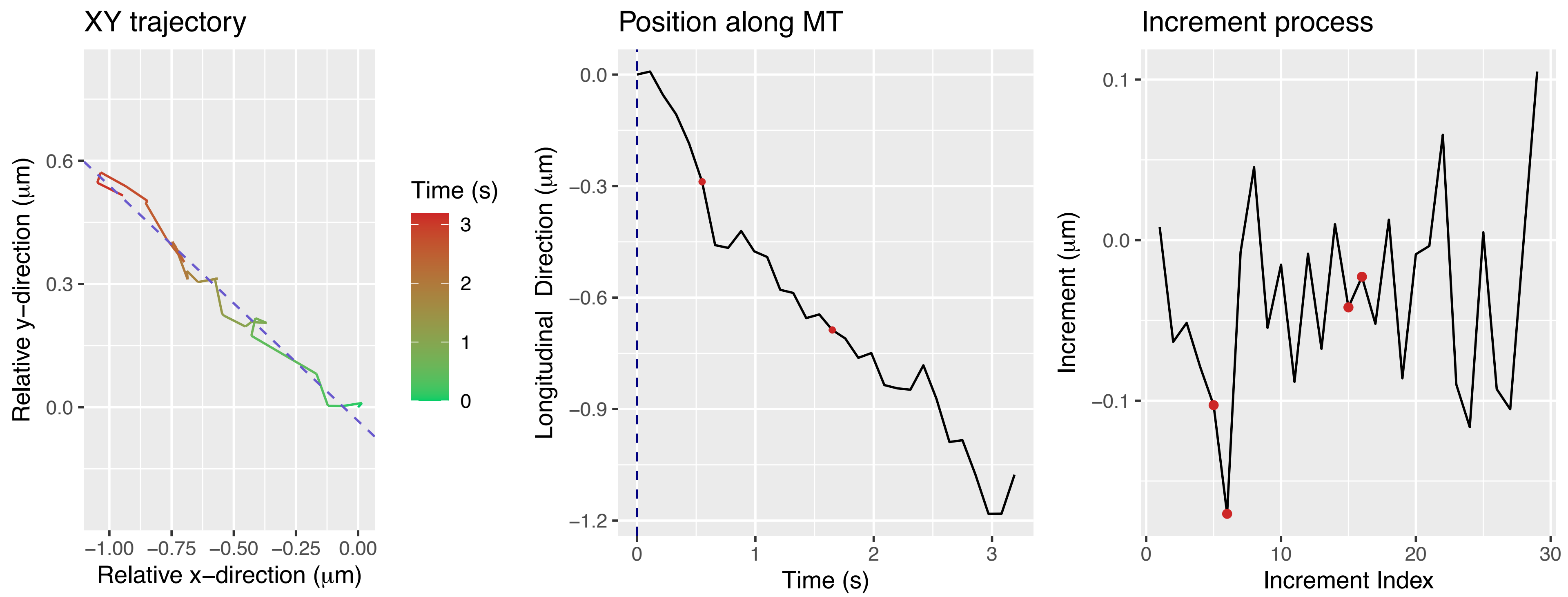

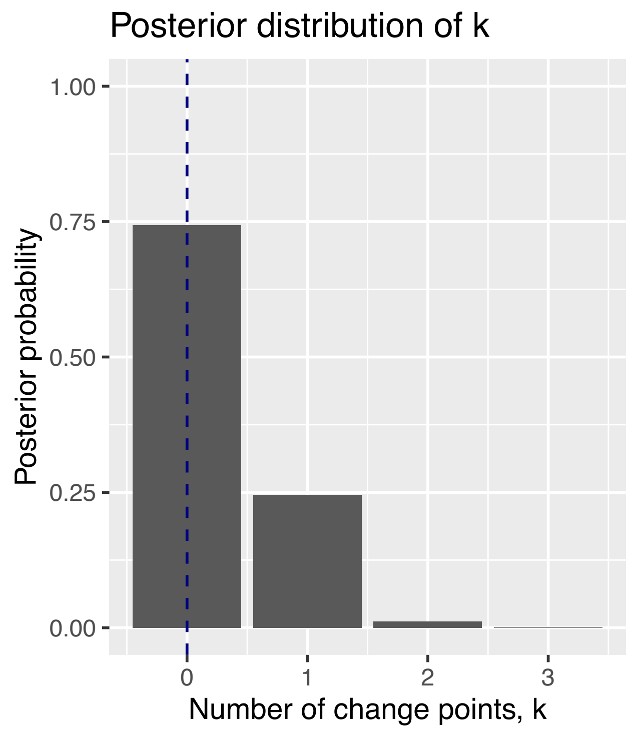

	State 1
Change point	0
Velocity	−0.3384
Std dev	0.1823

Kin1DDB particle: 31

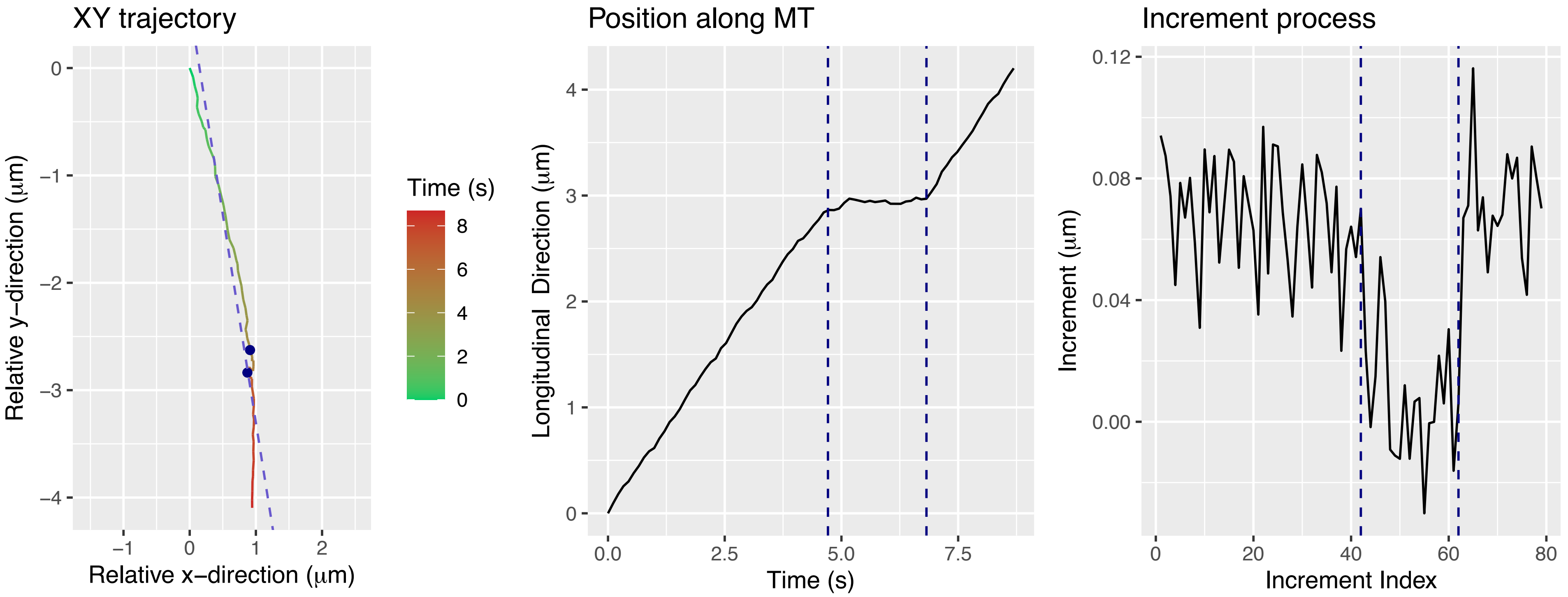

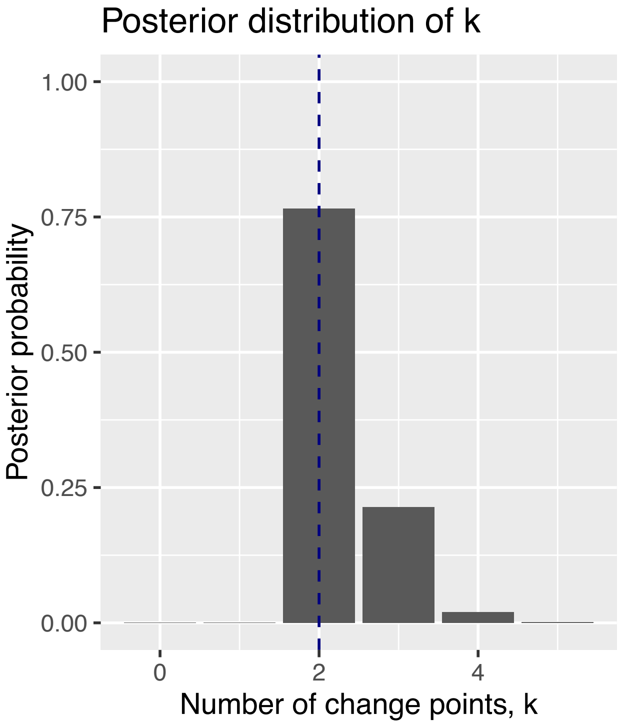

	State 1	State 2	State 3
Change point	4.7113	6.8209	NA
Velocity	0.6168	0.0546	0.6645
Std dev	0.0597	NA	NA

Kin1DDB particle: 33

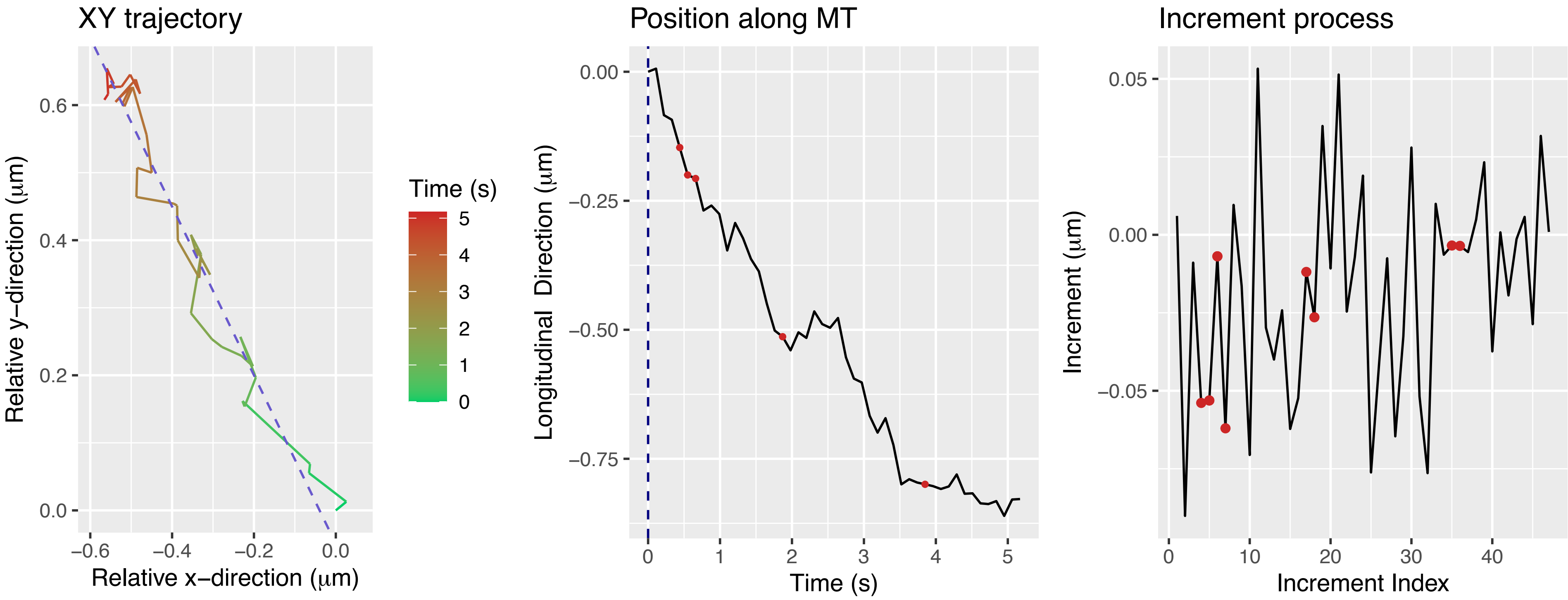

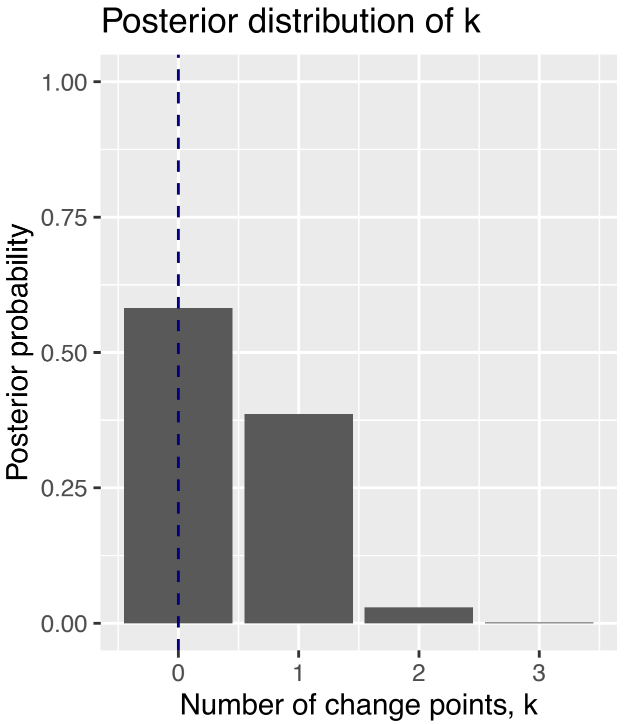

	State 1
Change point	0
Velocity	–0.1617
Std dev	0.1014

Kin1DDB particle: 34

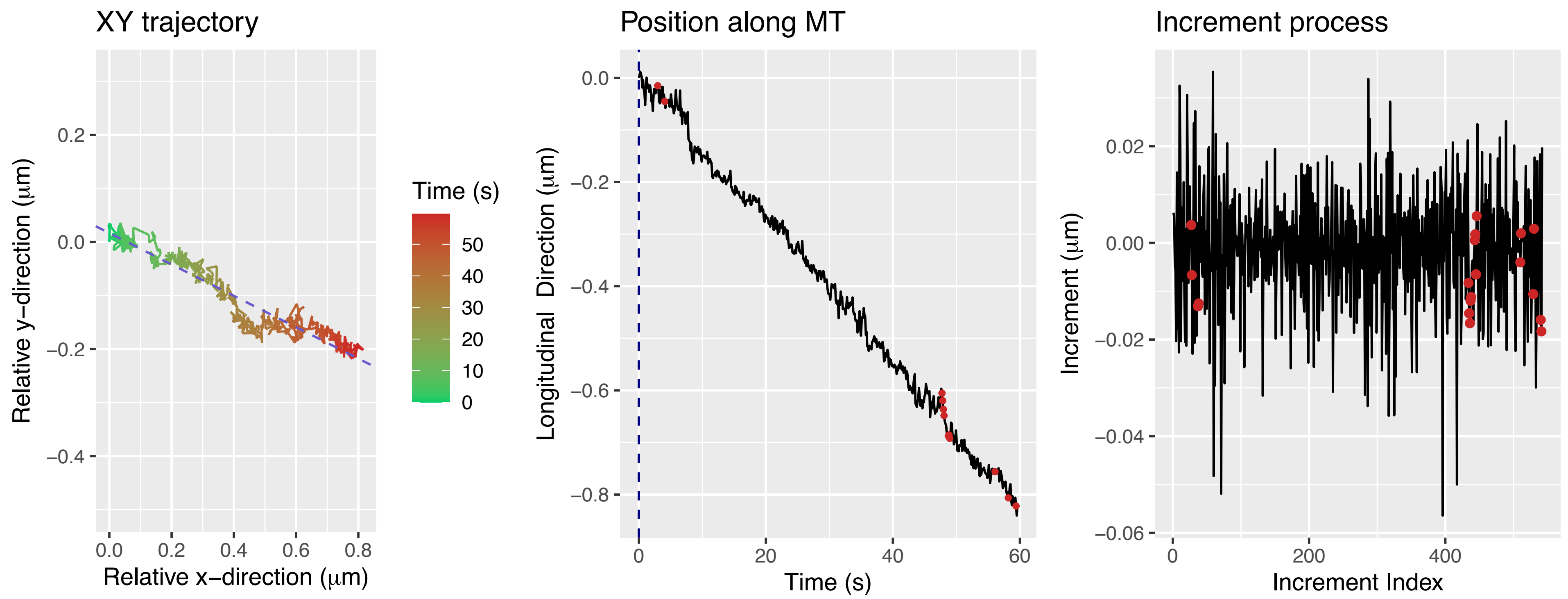

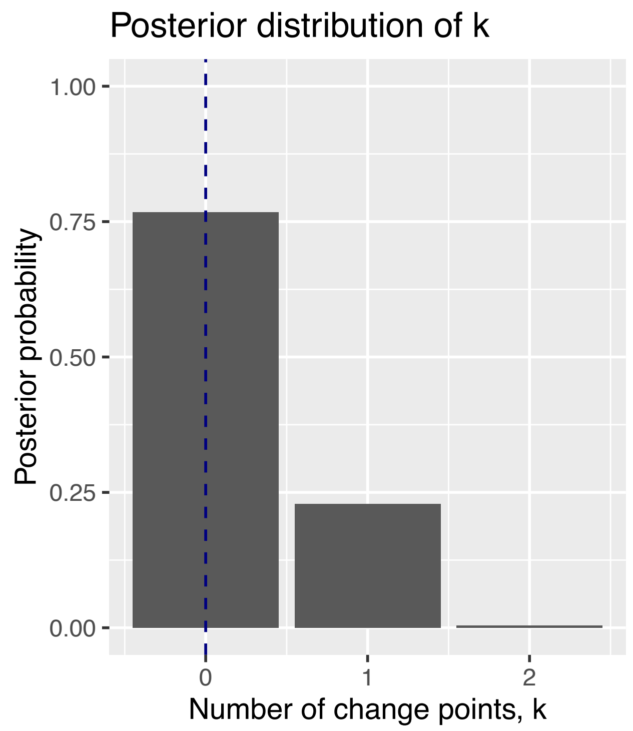

	State 1
Change point	0
Velocity	–0.0136
Std dev	0.0377

Kin1DDB particle: 35

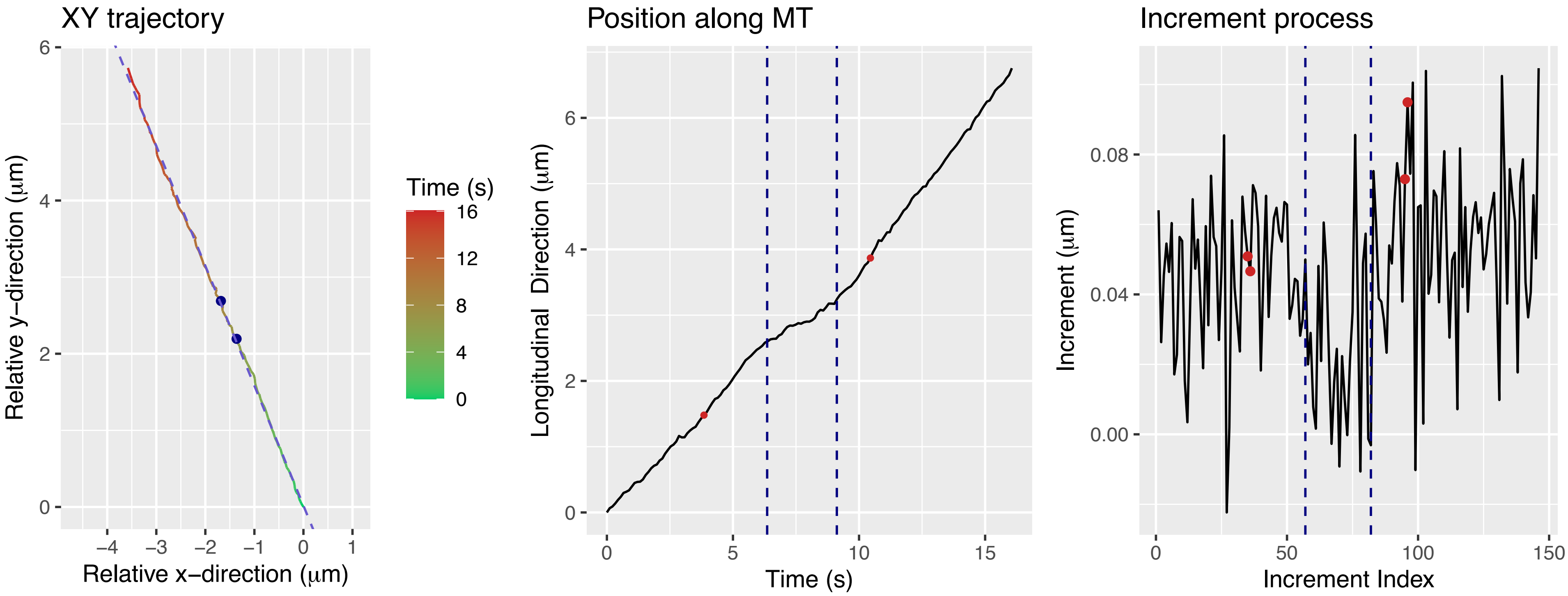

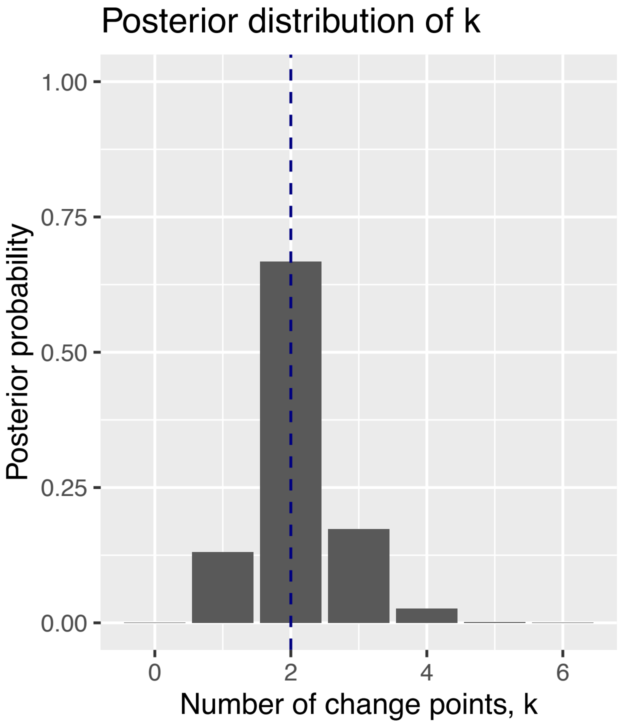

	State 1	State 2	State 3
Change point	6.3563	9.1197	NA
Velocity	0.418	0.2225	0.4893
Std dev	0.0693	NA	NA

Kin1DDB particle: 36

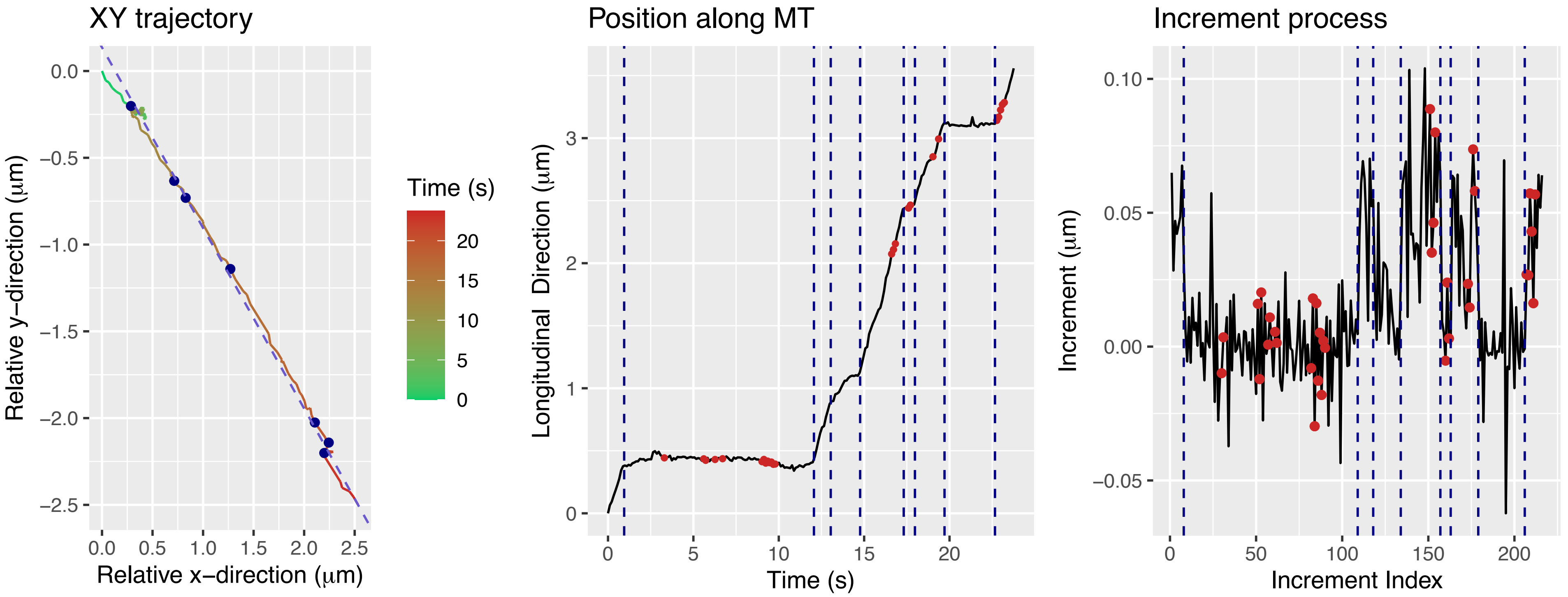

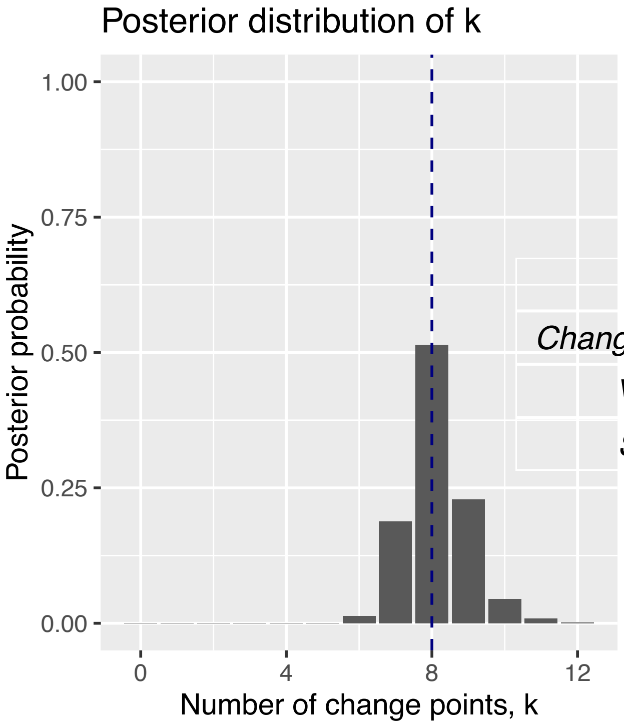

	State 1	State 2	State 3	State 4	State 5	State 6	State 7	State 8	[illegible]
Change point	0.9466	12.0596	13.0438	14.7648	17.3176	17.9742	19.7058	22.6616	[illegible]
Velocity	0.4215	−0.008	0.4812	0.1167	0.4938	0.0025	0.3708	−0.0147	[illegible]
Std dev	0.0535	NA	NA	NA	NA	NA	NA	NA	[illegible]

Kin1DDB particle: 37

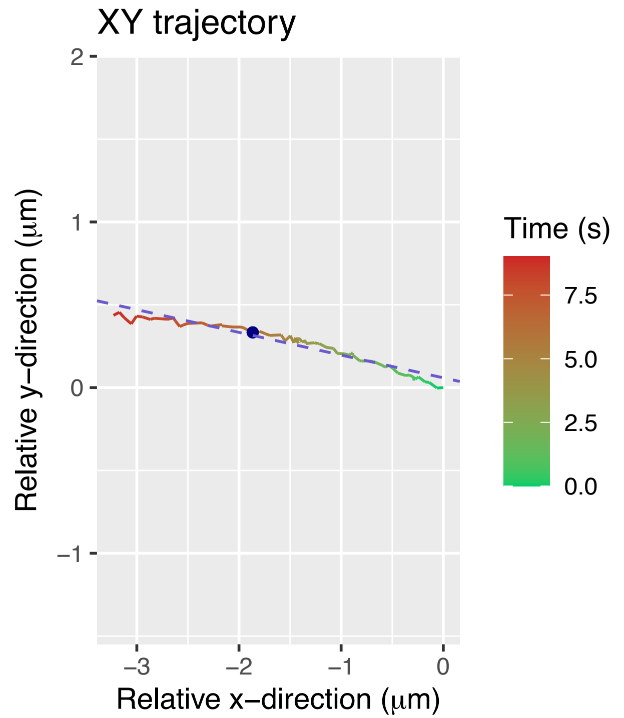

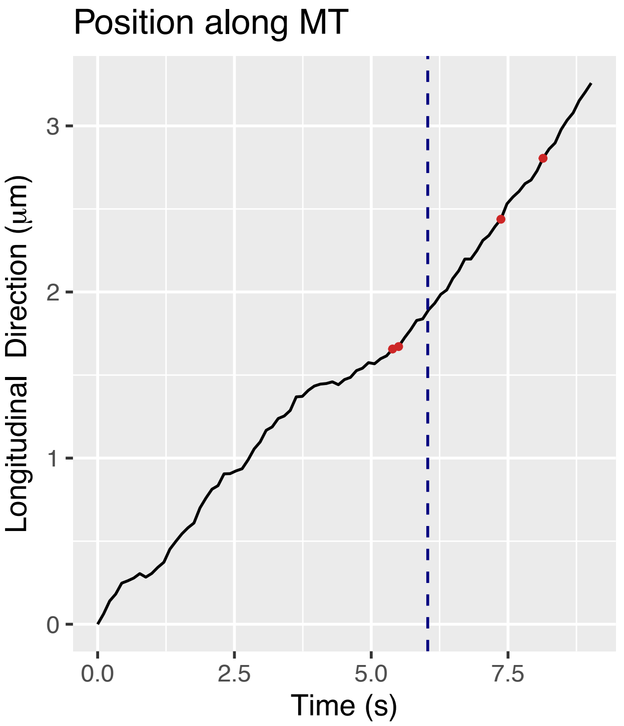

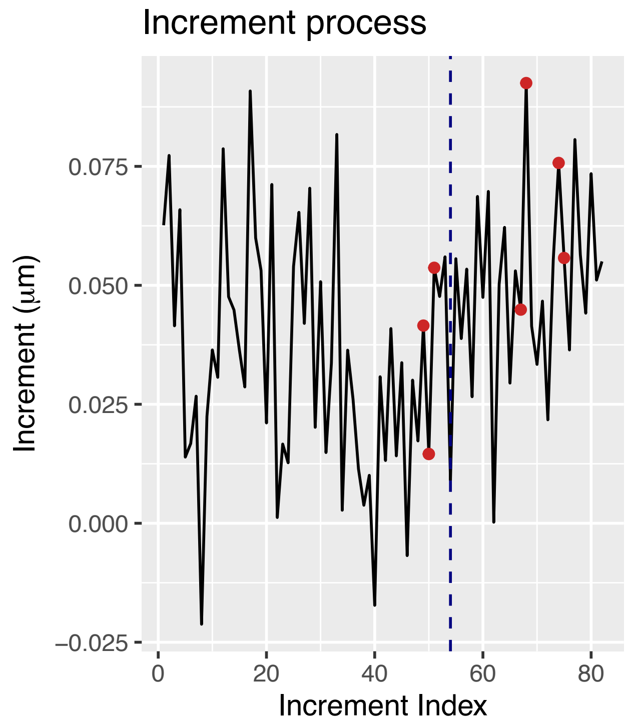

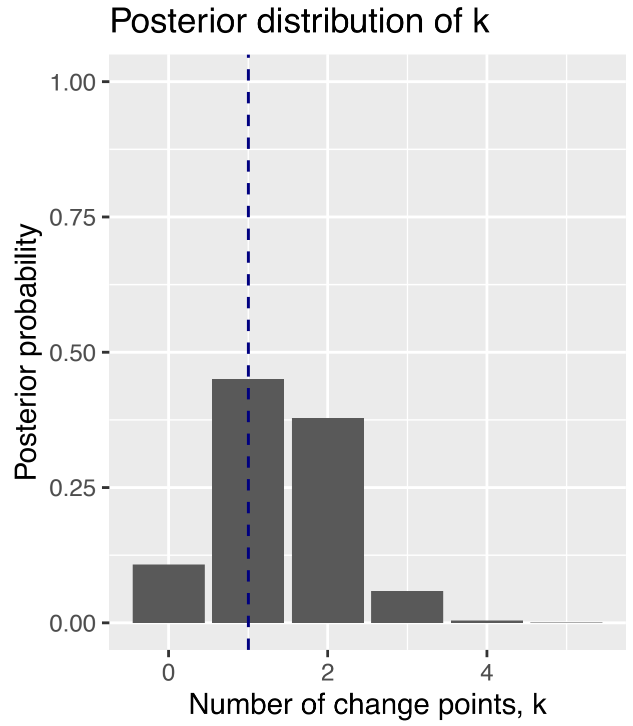

	**State 1**	**State 2**
Change point	6.0324	NA
Velocity	0.3126	0.4541
Std dev	0.0697	NA

Kin1DDB particle: 38

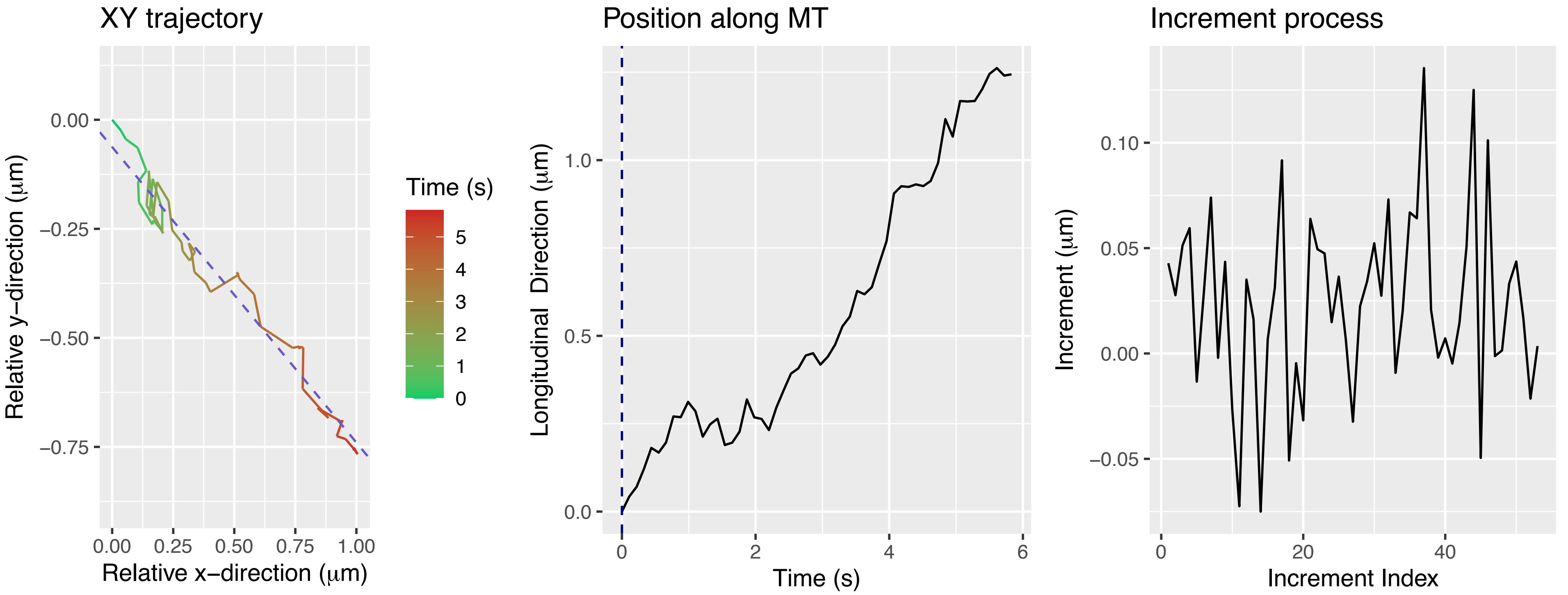

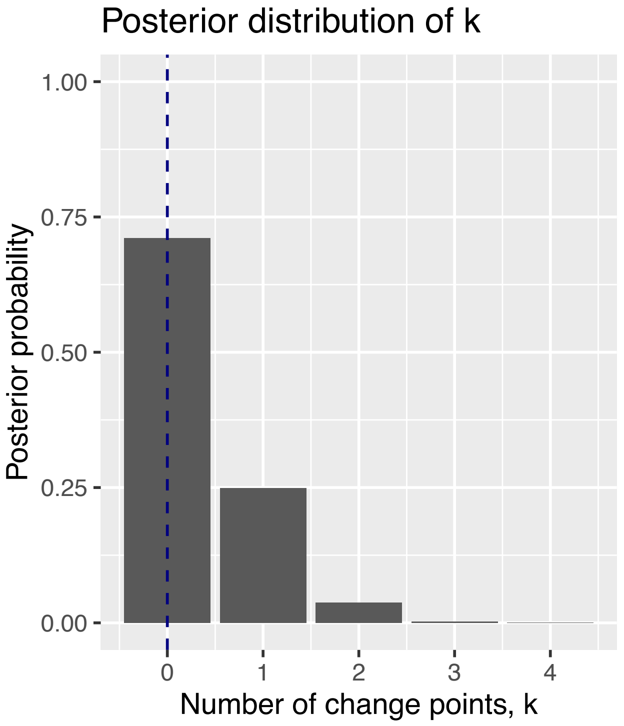

	State 1
Change point	0
Velocity	0.2165
Std dev	0.1312

Kin1DDB particle: 39

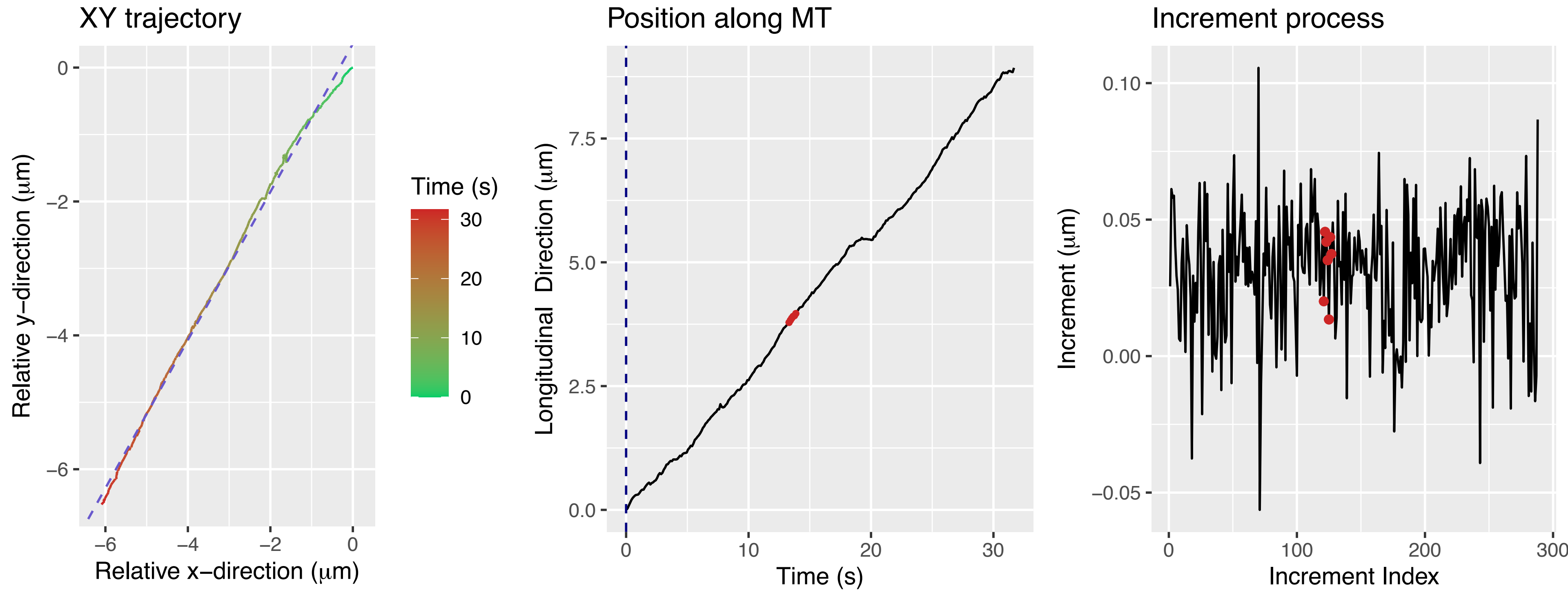

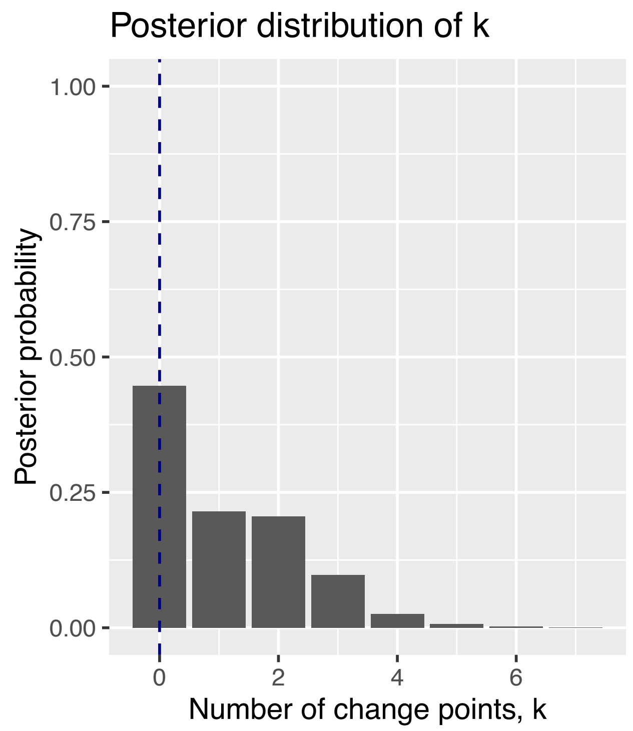

	State 1
Change point	0
Velocity	0.2823
Std dev	0.0689

Kin1DDB particle: 40

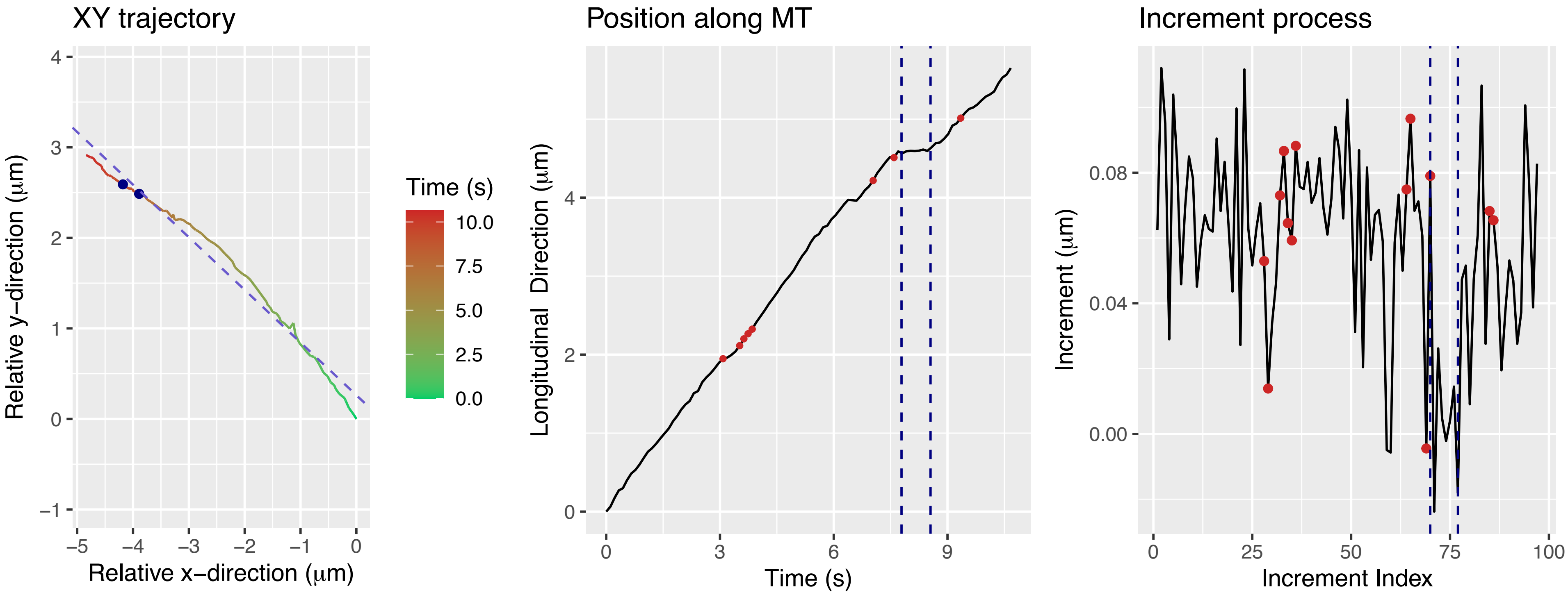

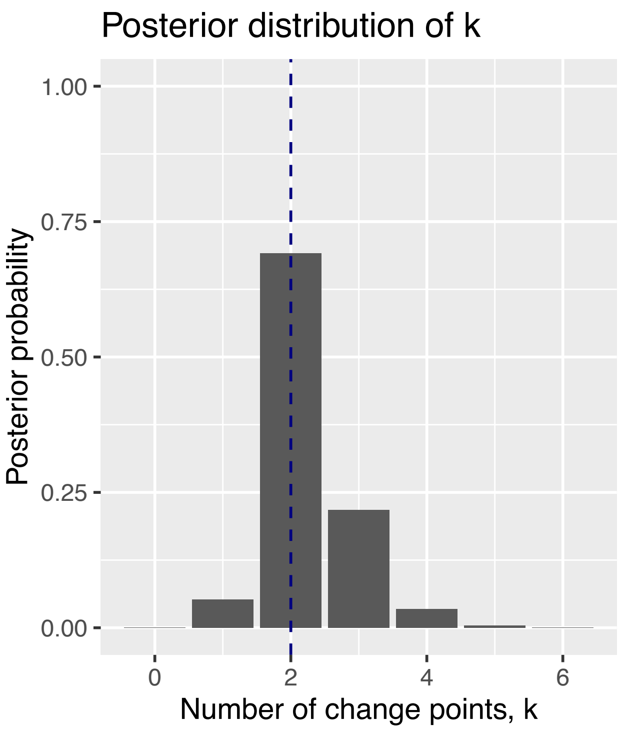

	State 1	State 2	State 3
Change point	7.7881	8.553	NA
Velocity	0.6011	−0.003	0.4867
Std dev	0.0763	NA	NA

Kin1DDB particle: 41

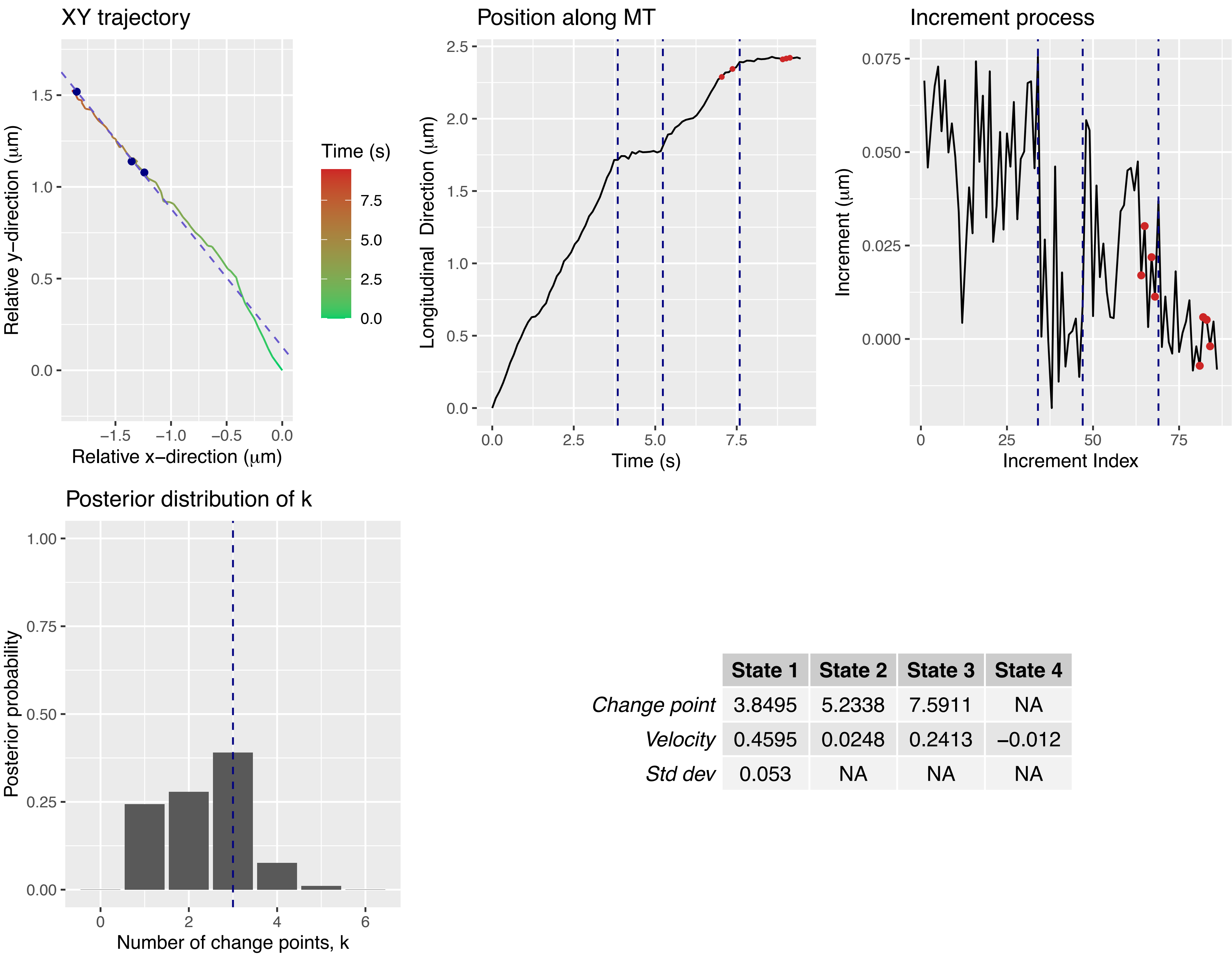

	State 1	State 2	State 3	State 4
Change point	3.8495	5.2338	7.5911	NA
Velocity	0.4595	0.0248	0.2413	−0.012
Std dev	0.053	NA	NA	NA

Kin1DDB particle: 42

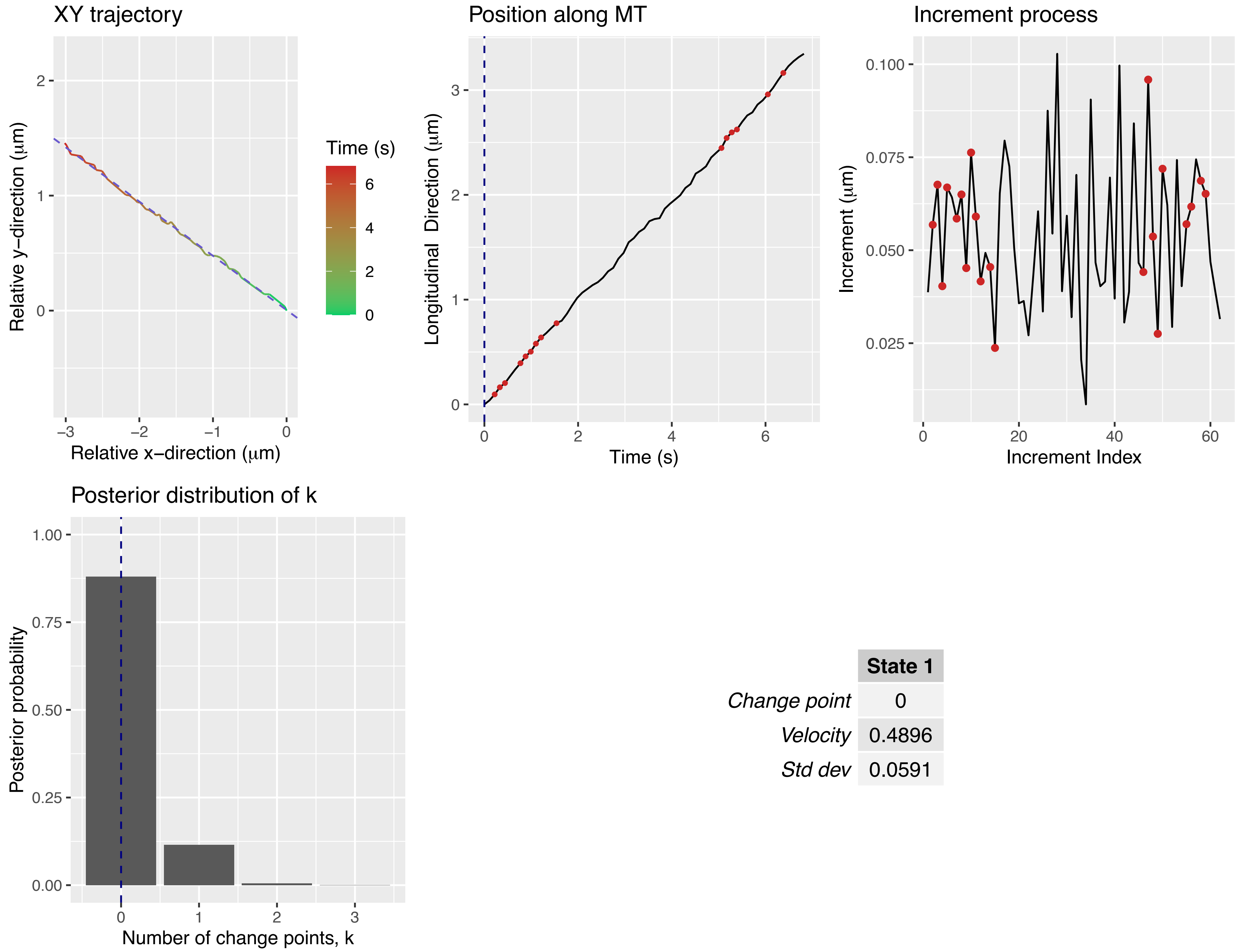

	State 1
Change point	0
Velocity	0.4896
Std dev	0.0591

Kin1DDB particle: 44

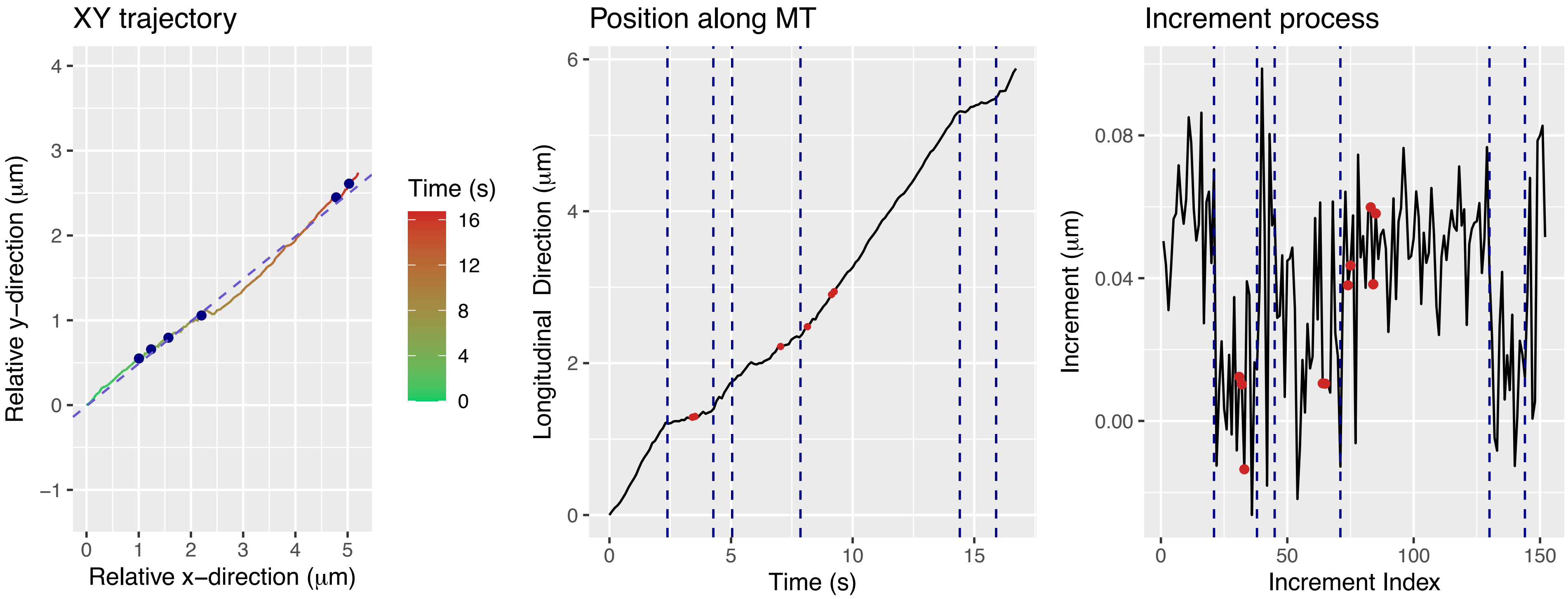

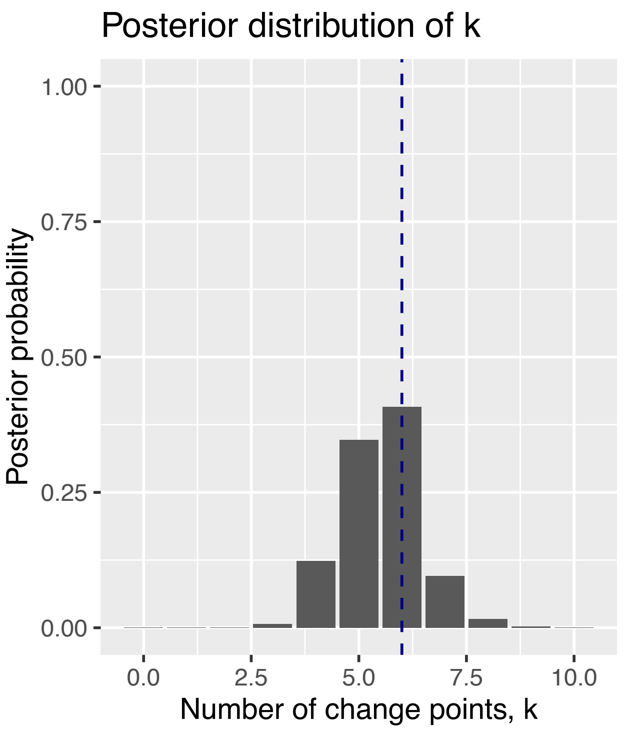

	State 1	State 2	State 3	State 4	State 5	State 6	State 7
Change point	2.3848	4.2724	5.0461	7.8539	14.4049	15.8988	NA
Velocity	0.5259	0.0786	0.4915	0.2031	0.4541	0.0843	0.4928
Std dev	0.0588	NA	NA	NA	NA	NA	NA

Kin1DDB particle: 45

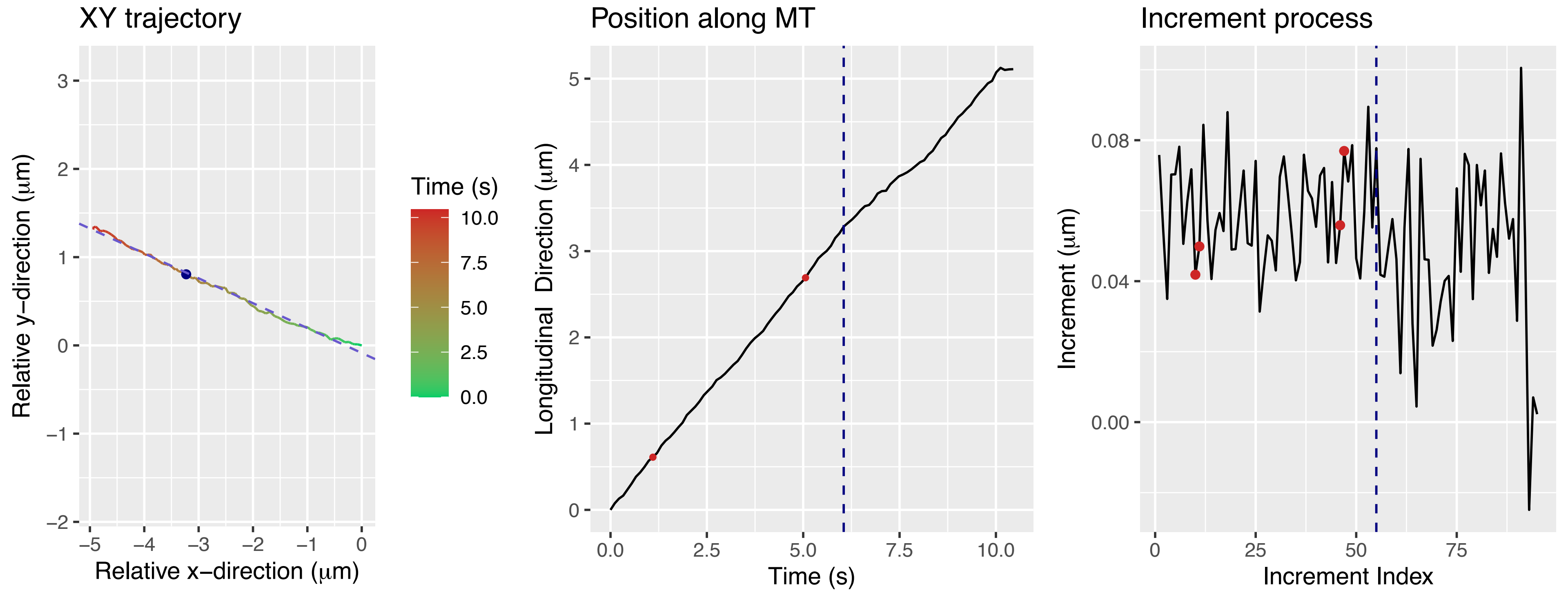

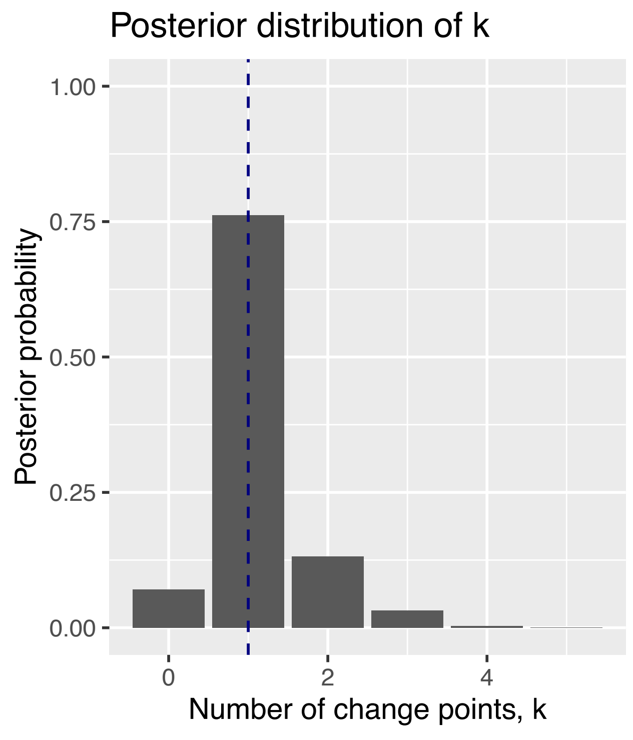

	State 1	State 2
Change point	6.0509	NA
Velocity	0.5414	0.4123
Std dev	0.0577	NA

Kin1DDB particle: 46

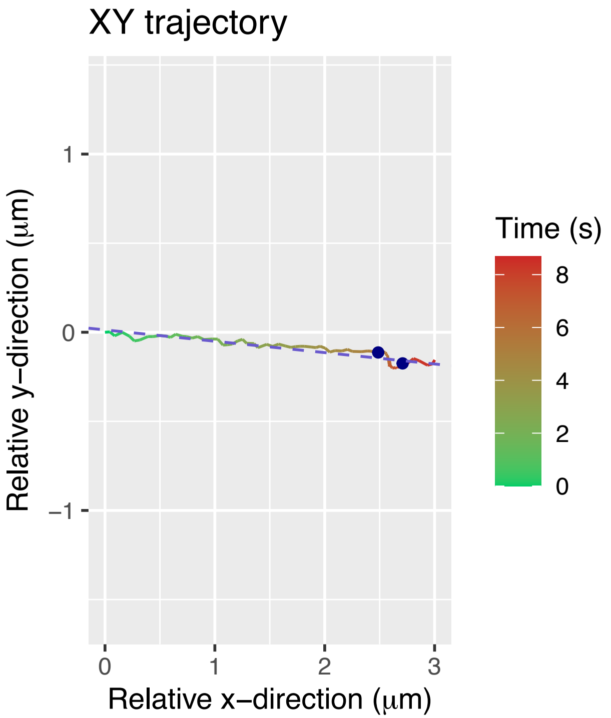

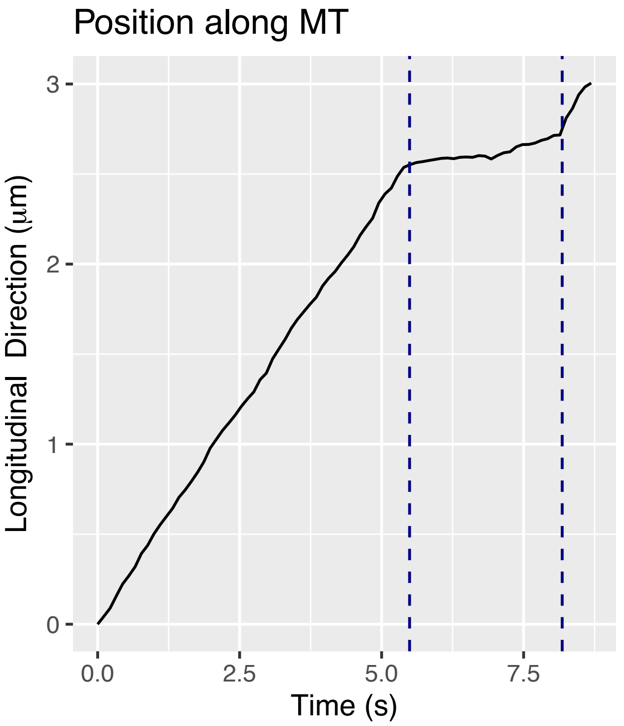

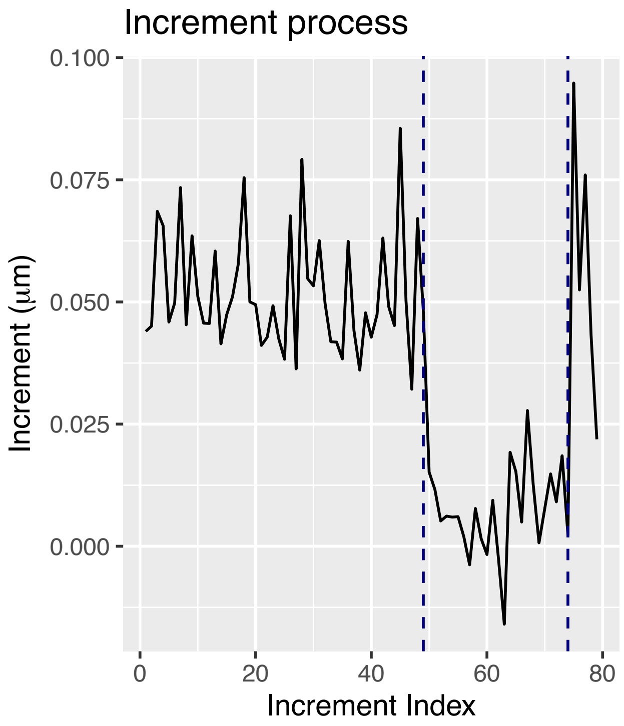

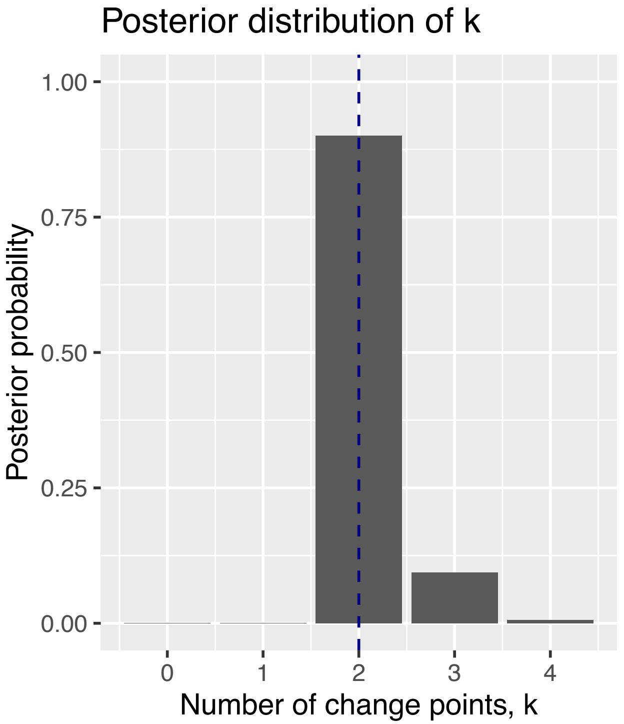

	State 1	State 2	State 3
Change point	5.4921	8.1793	NA
Velocity	0.4695	0.0621	0.5386
Std dev	0.0436	NA	NA

Kin1DDB particle: 47

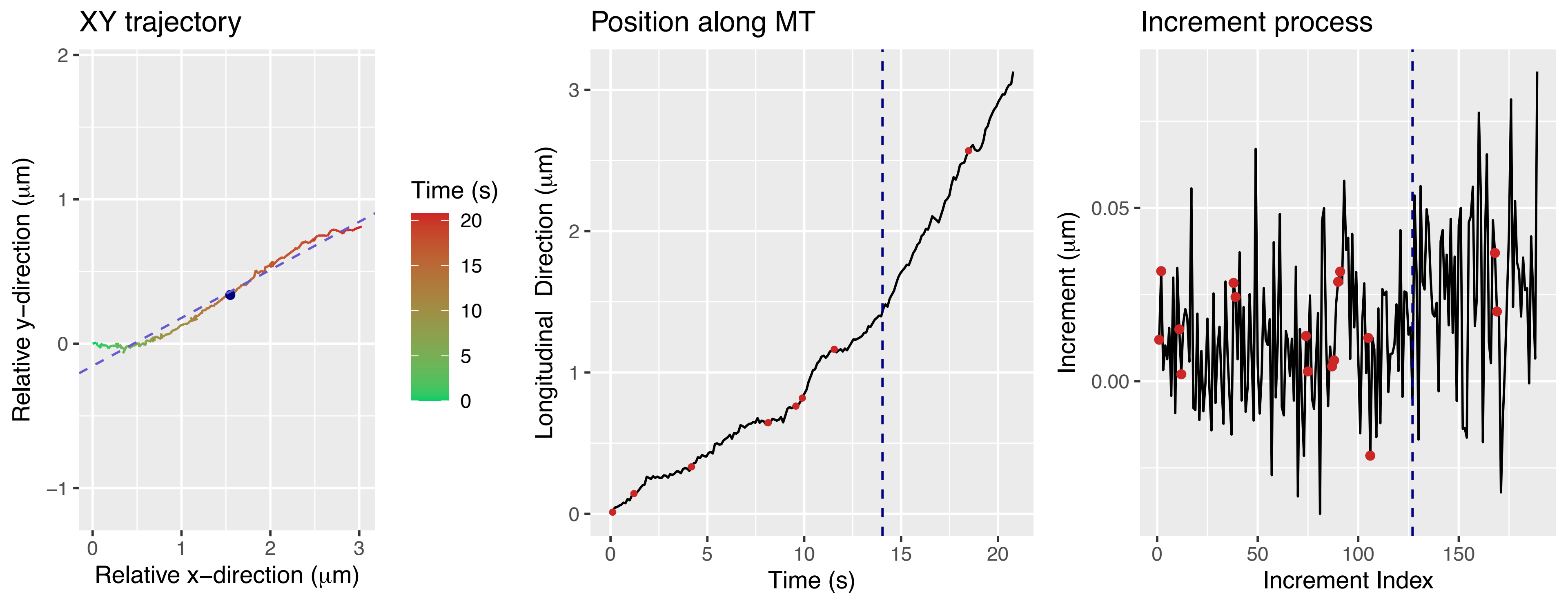

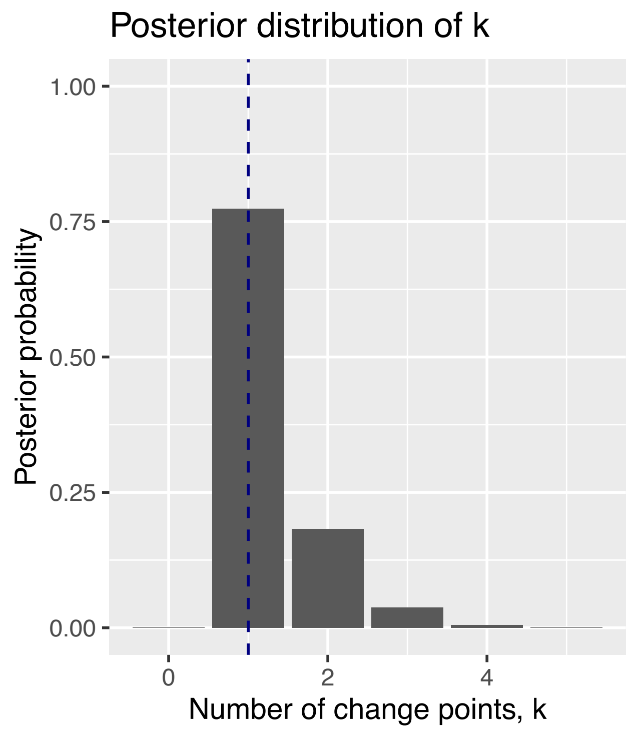

	State 1	State 2
Change point	14.0393	NA
Velocity	0.0974	0.2489
Std dev	0.0627	NA

Kin1DDB particle: 48

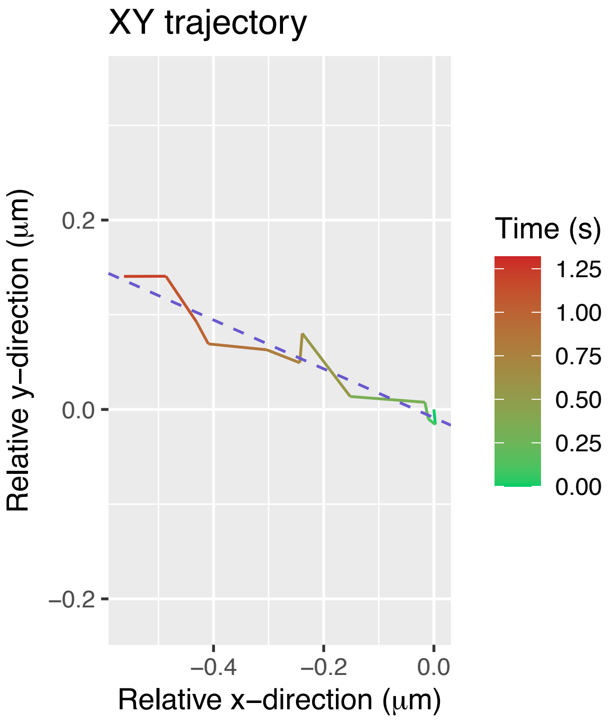

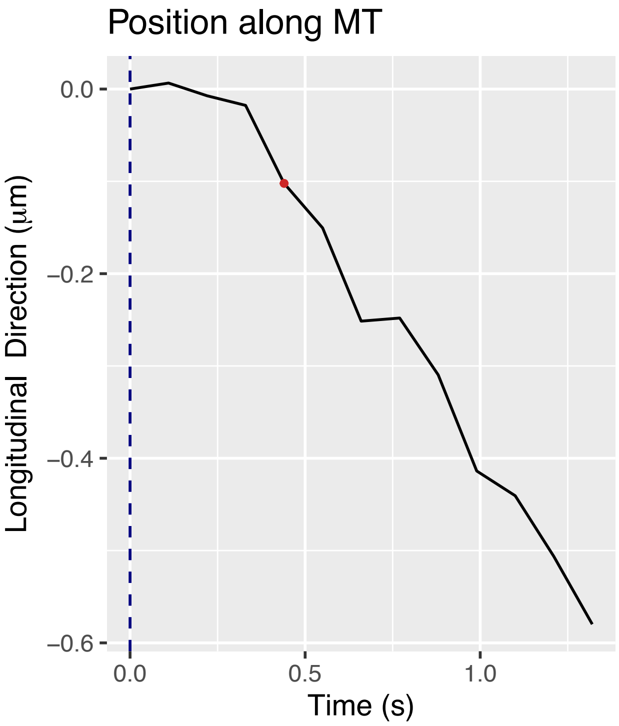

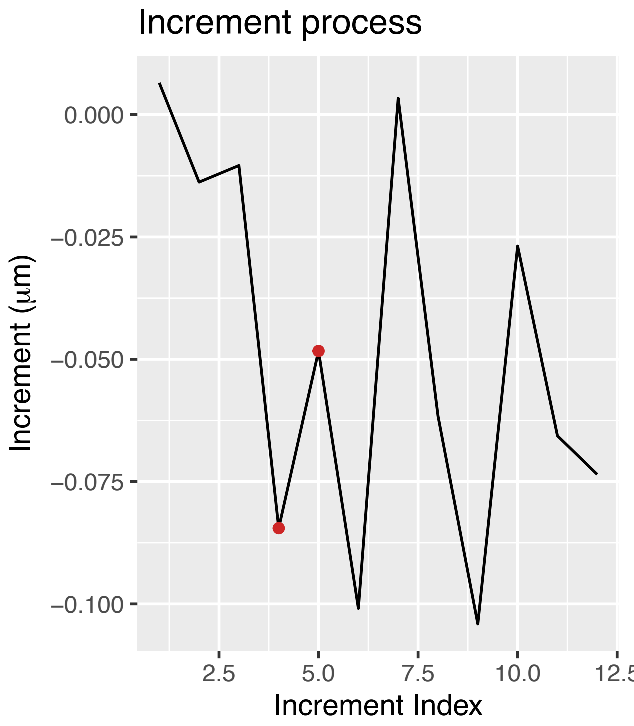

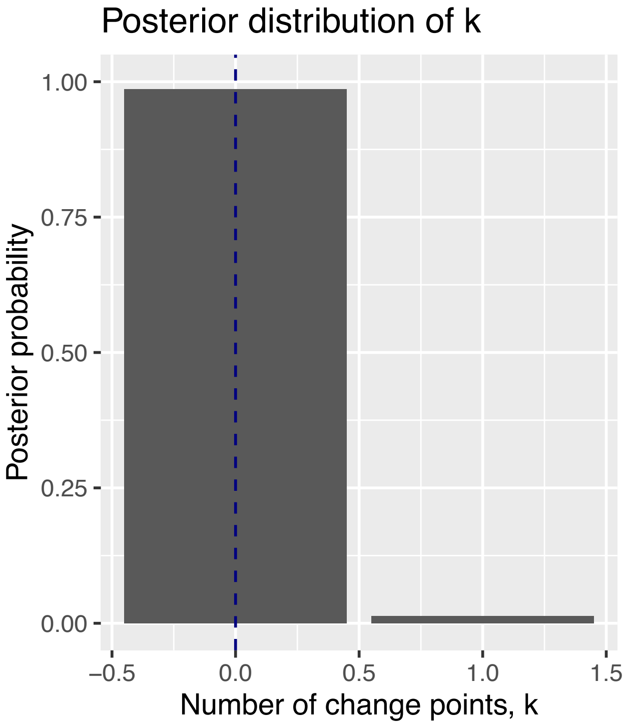

	State 1
Change point	0
Velocity	−0.4422
Std dev	0.1161

Kin1DDB particle: 49

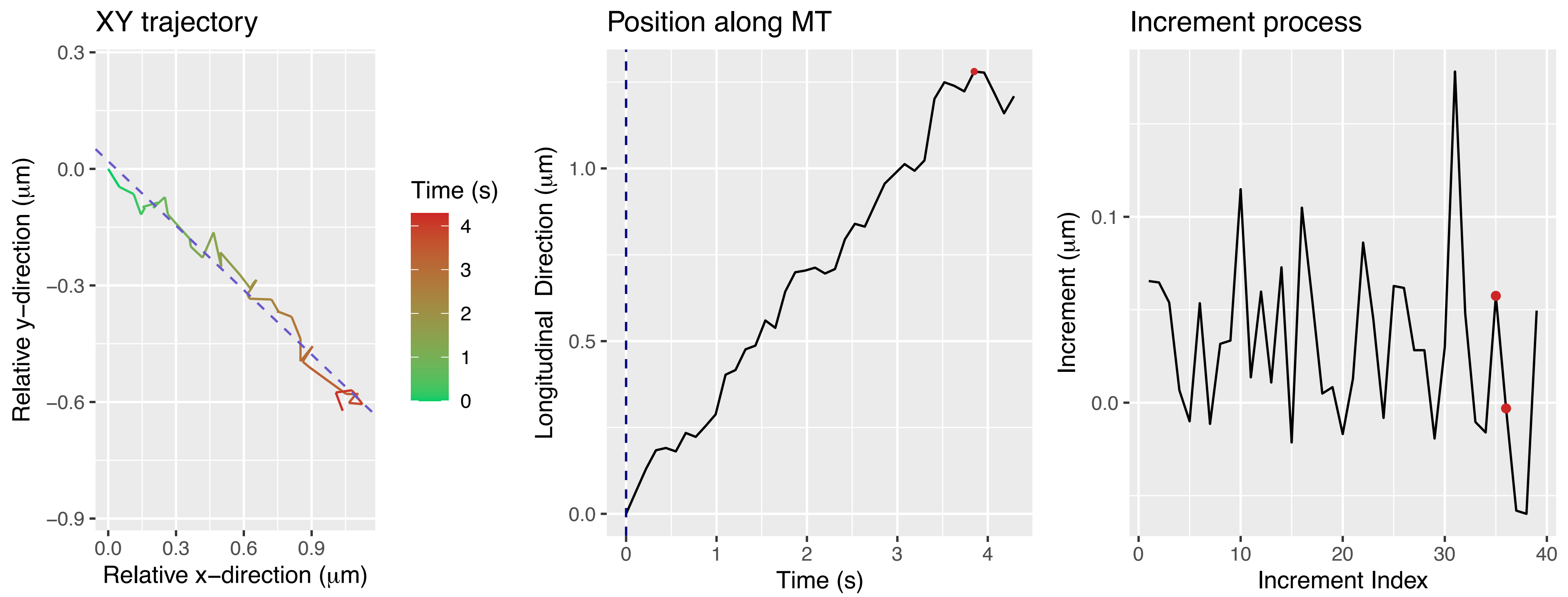

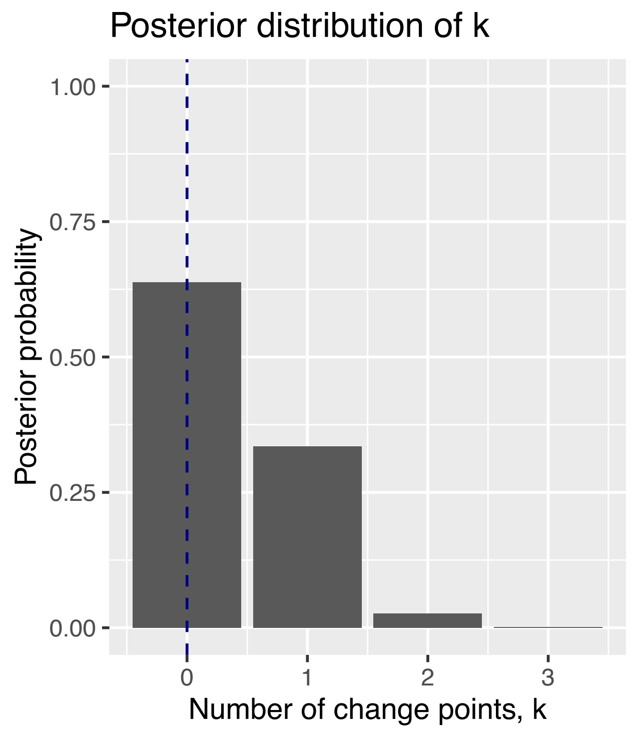

	State 1
Change point	0
Velocity	0.2831
Std dev	0.1426

Kin1DDB particle: 50

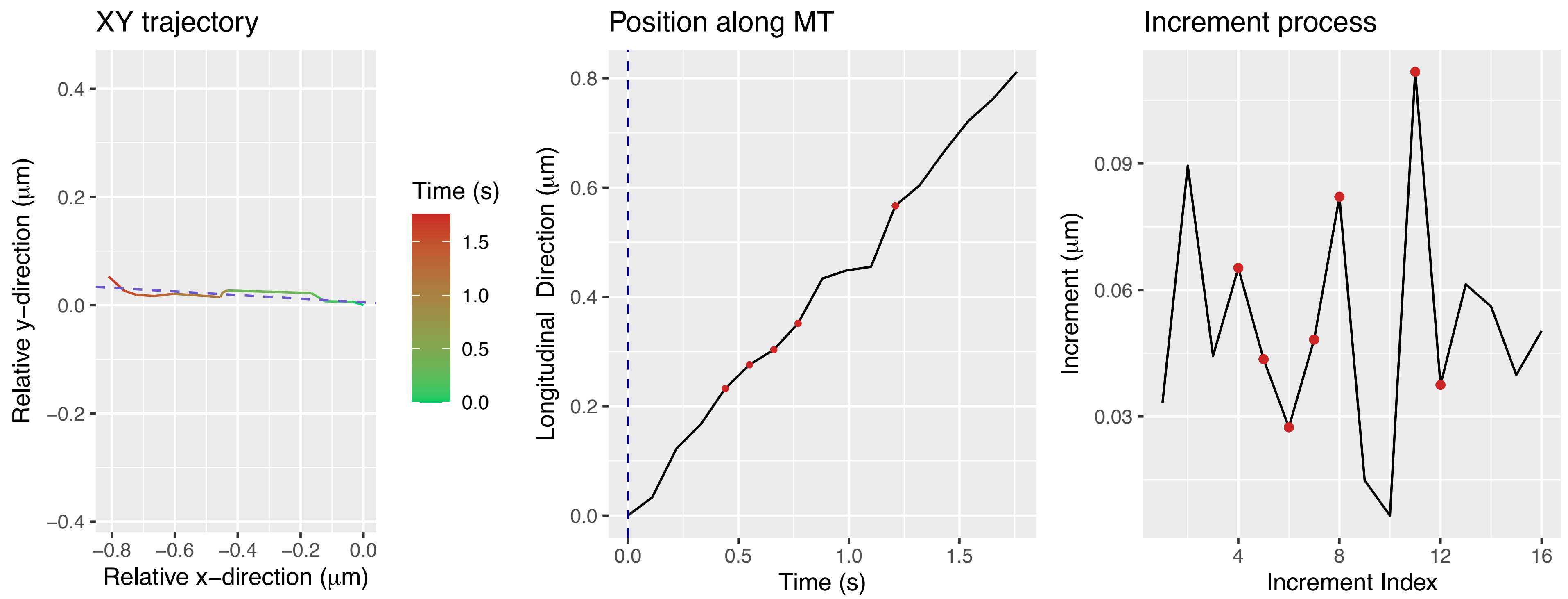

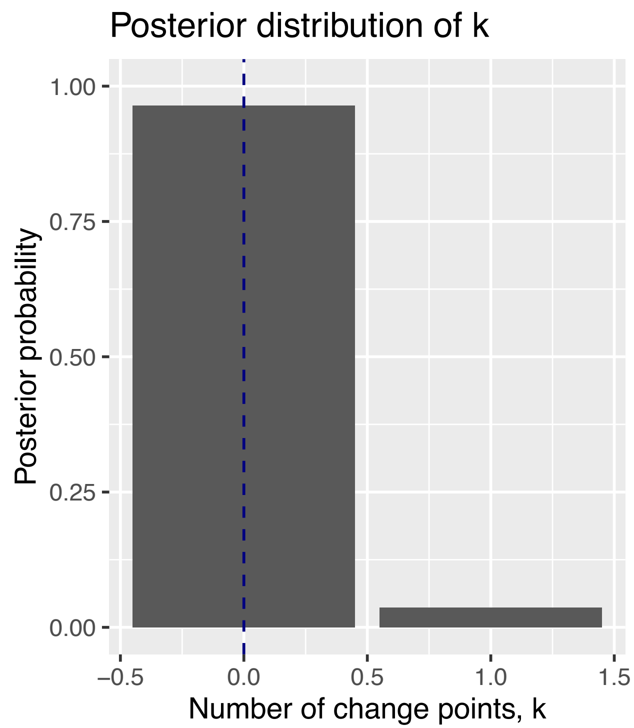

	**State 1**
Change point	0
Velocity	0.4611
Std dev	0.075

Kin1DDB particle: 51

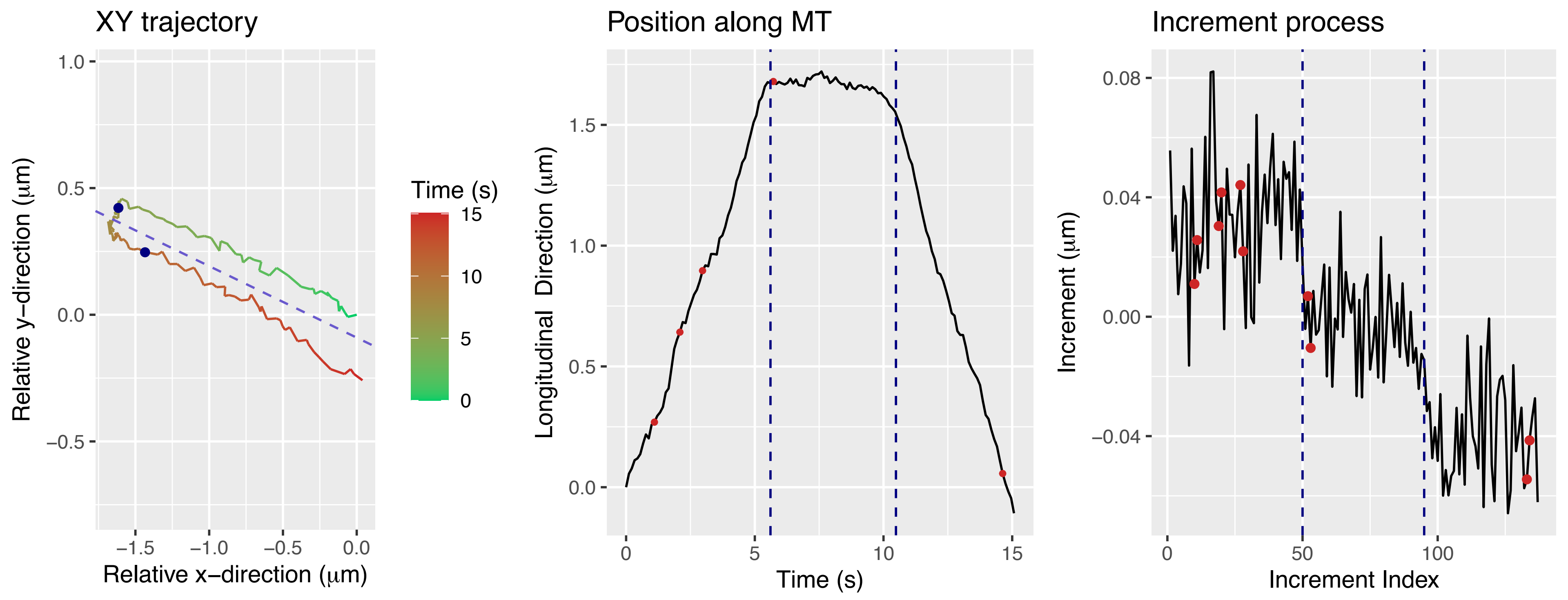

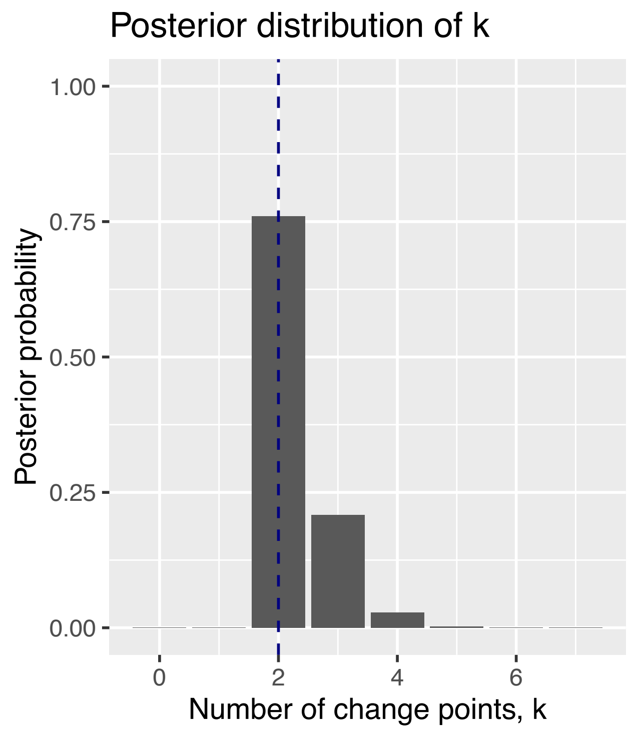

	State 1	State 2	State 3
Change point	5.6084	10.4838	NA
Velocity	0.3064	−0.0211	−0.352
Std dev	0.0563	NA	NA

Kin1DDB particle: 53

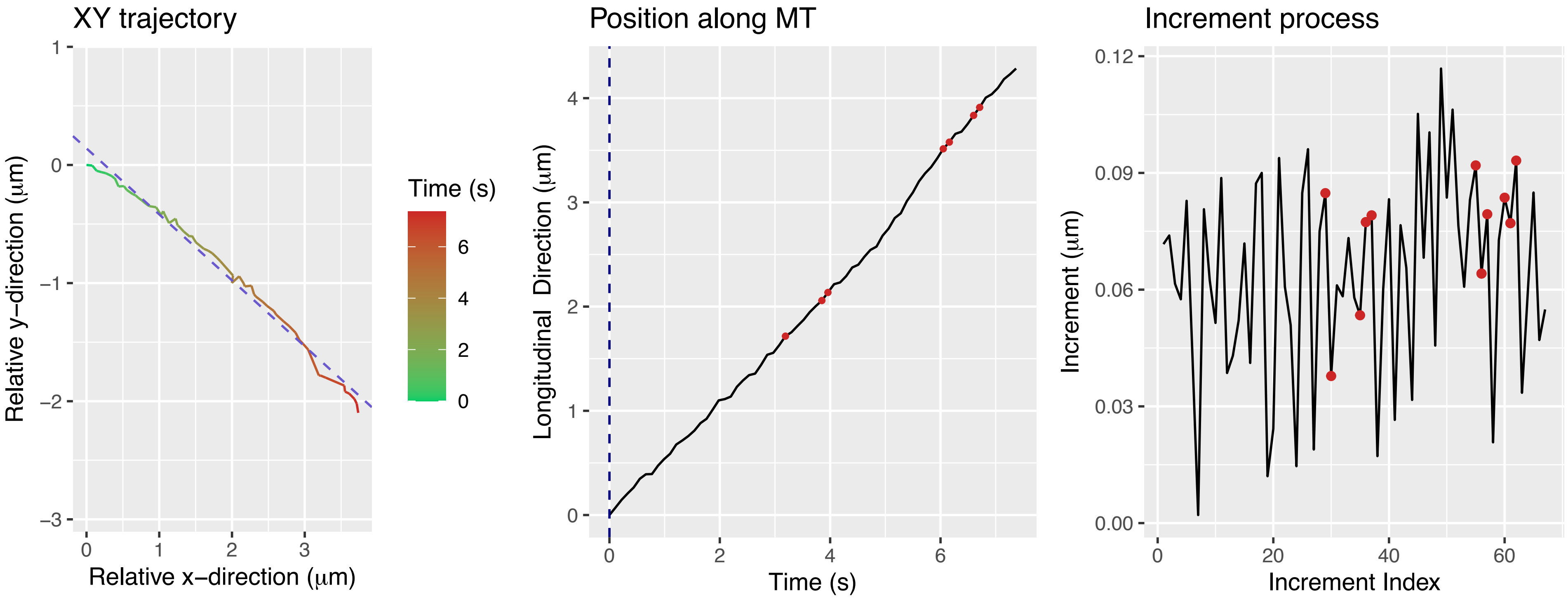

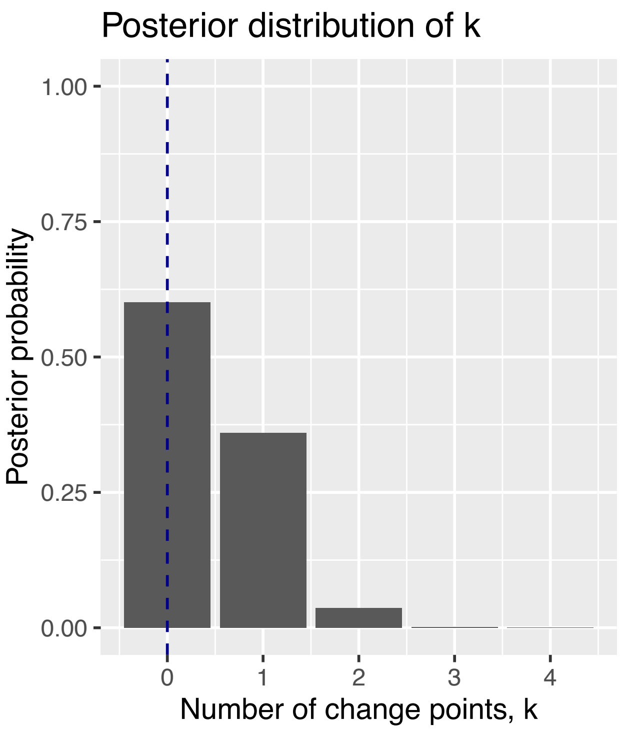

	State 1
Change point	0
Velocity	0.5817
Std dev	0.0743

Kin1DDB particle: 54

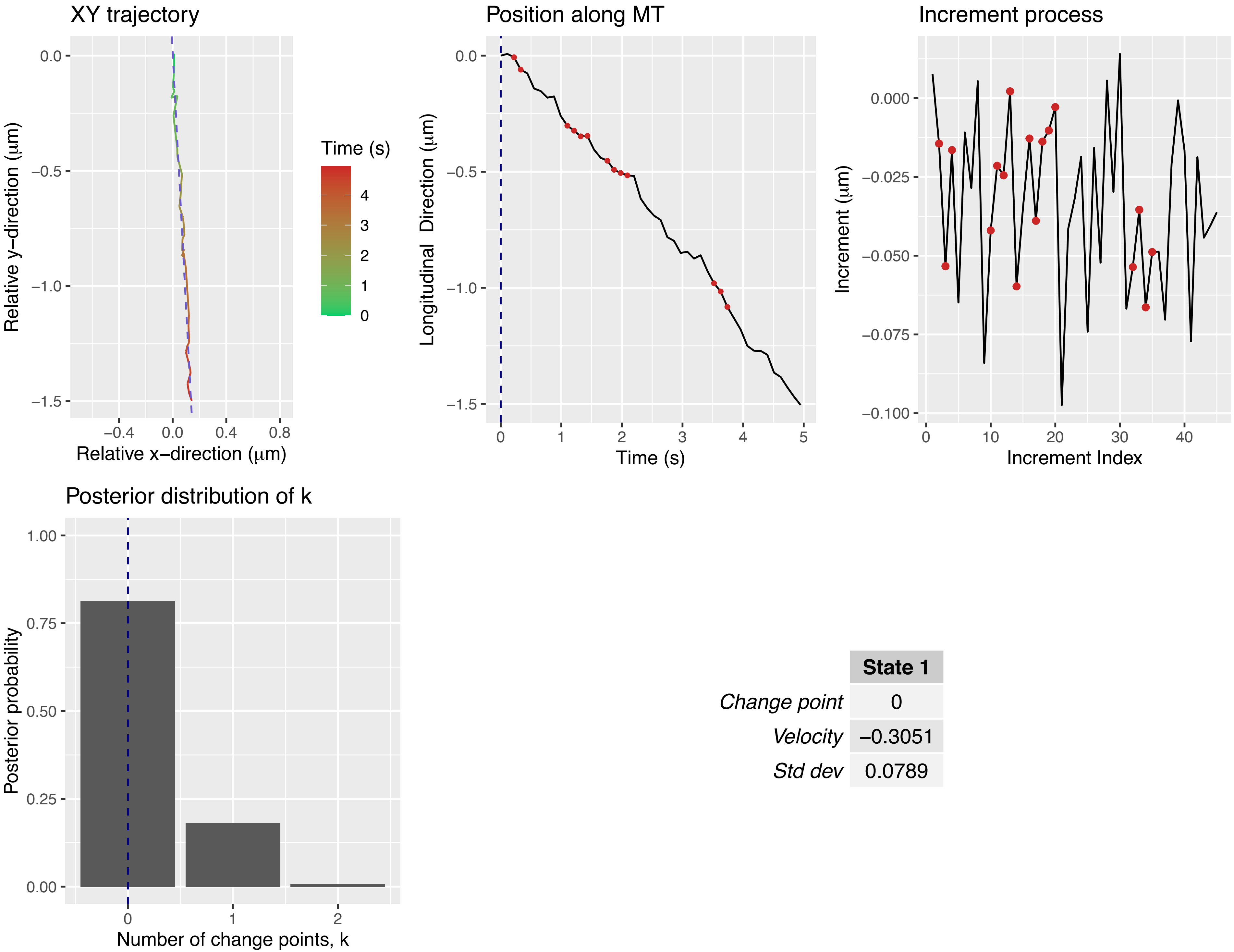

	State 1
Change point	0
Velocity	−0.3051
Std dev	0.0789

Kin1DDB particle: 56

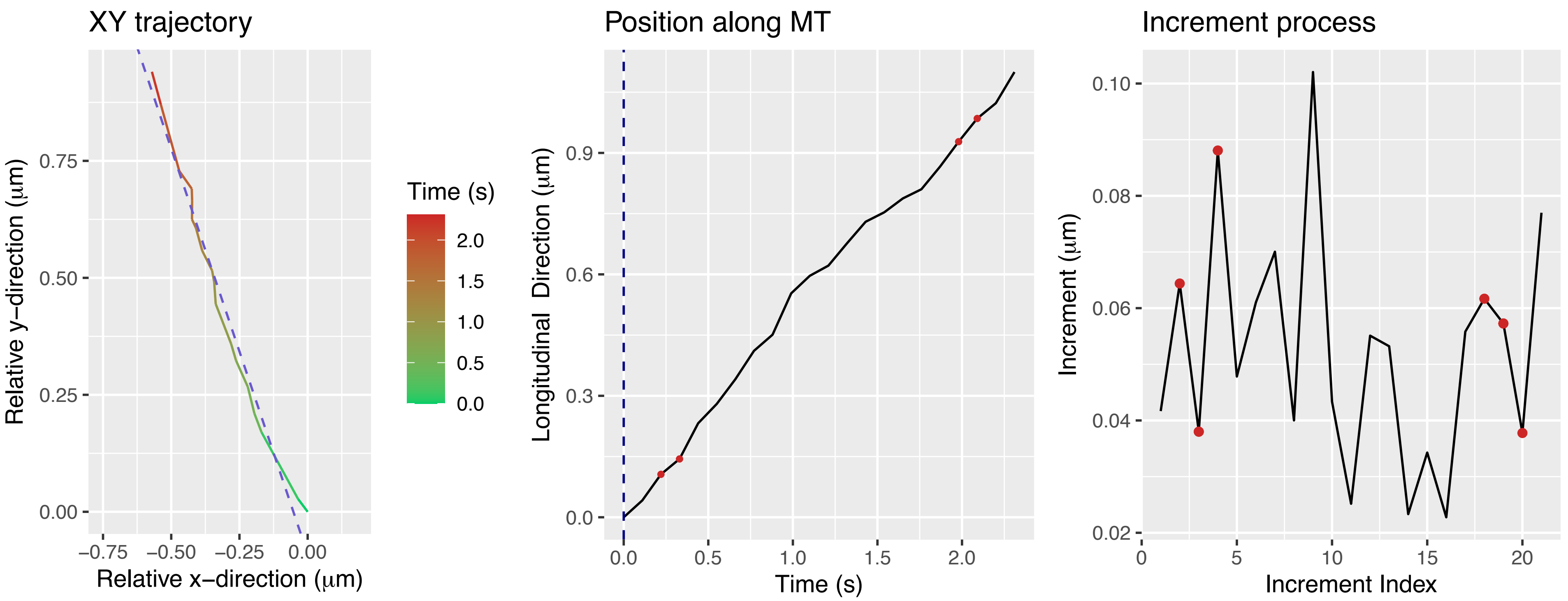

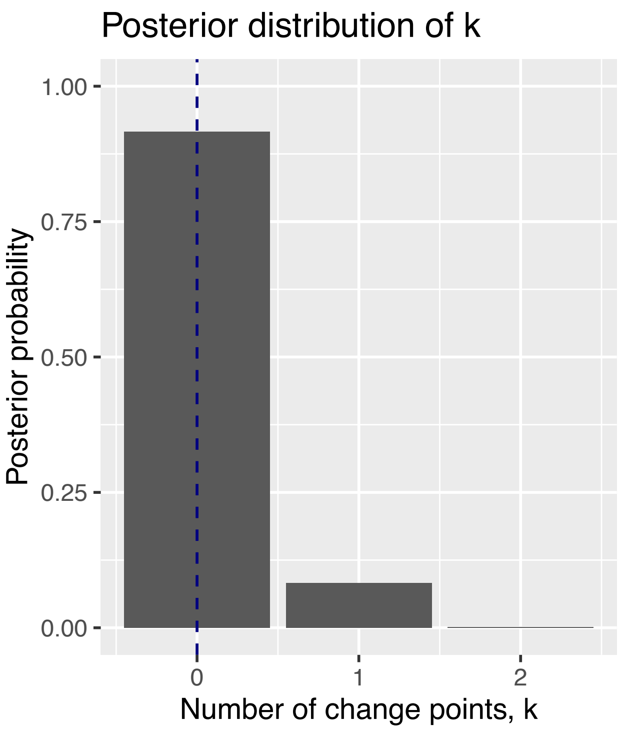

	**State 1**
Change point	0
Velocity	0.4767
Std dev	0.0562

Kin1DDB particle: 58

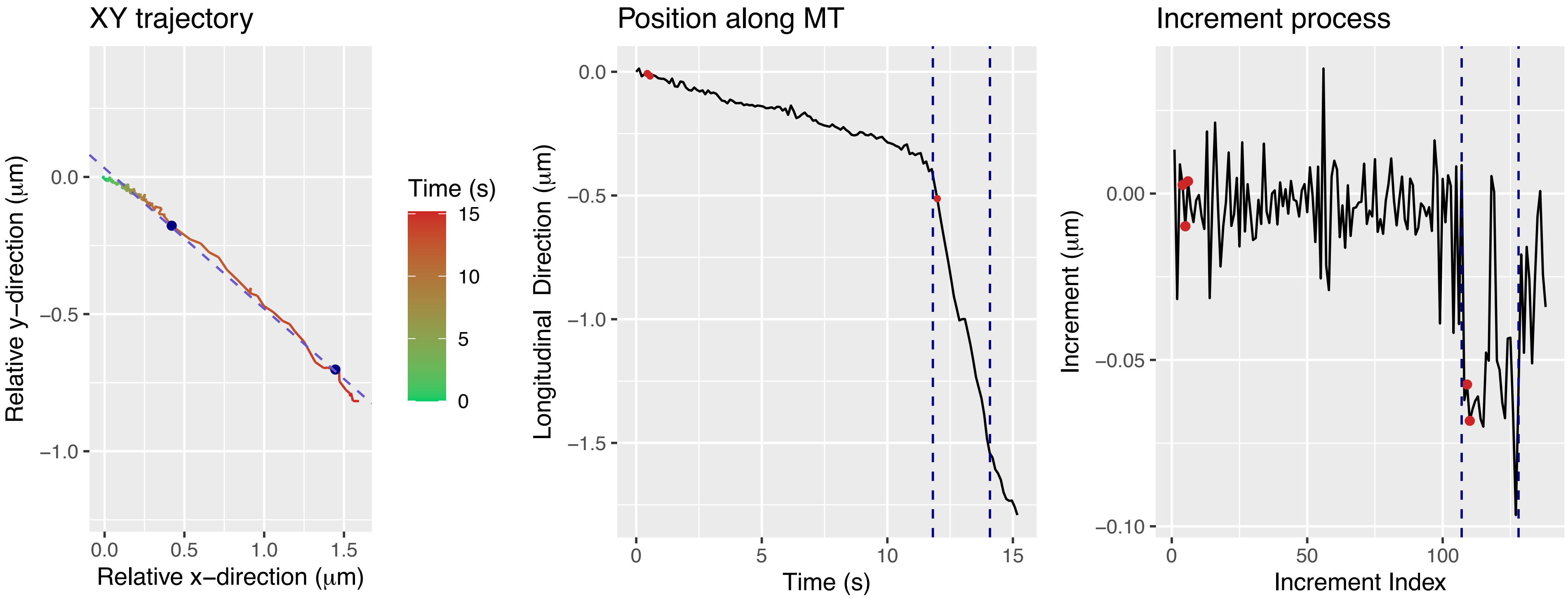

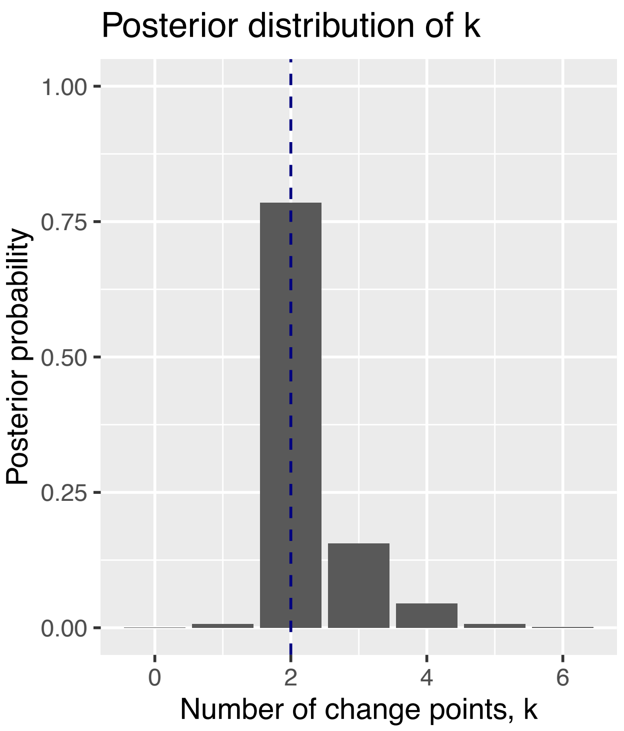

	State 1	State 2	State 3
Change point	11.8131	14.0864	NA
Velocity	−0.0333	−0.4921	−0.2293
Std dev	0.0462	NA	NA

Kin1DDB particle: 62

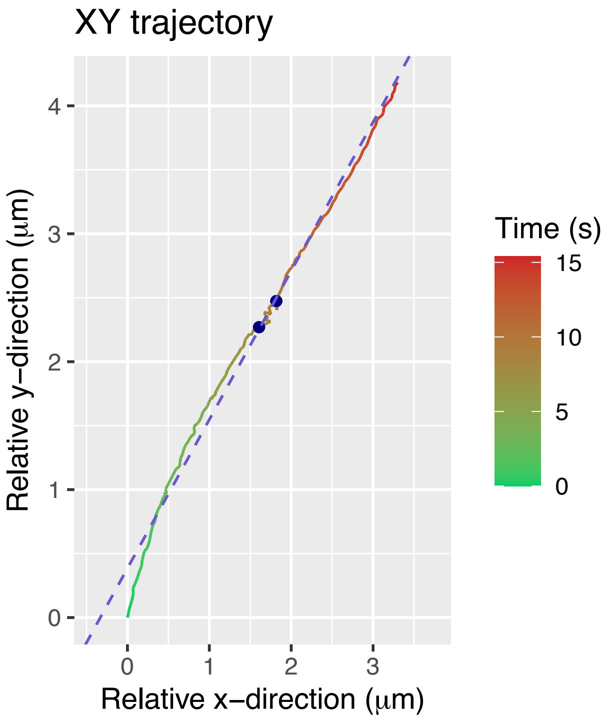

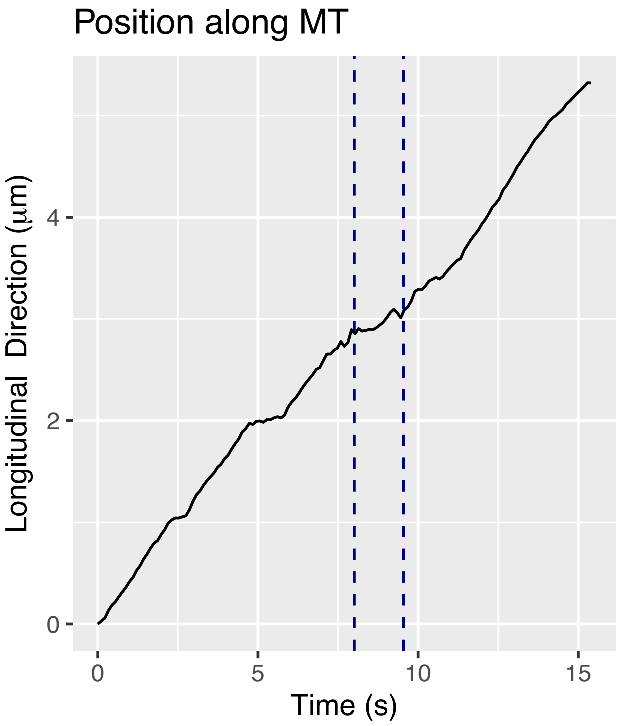

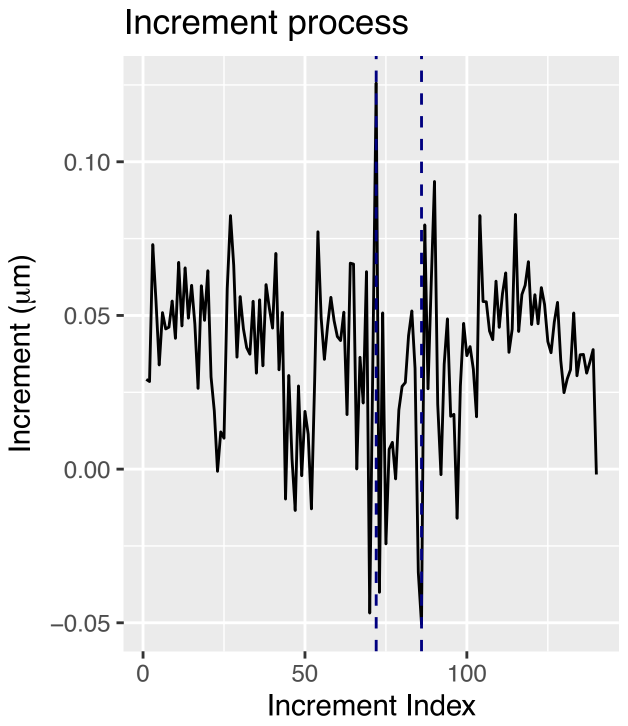

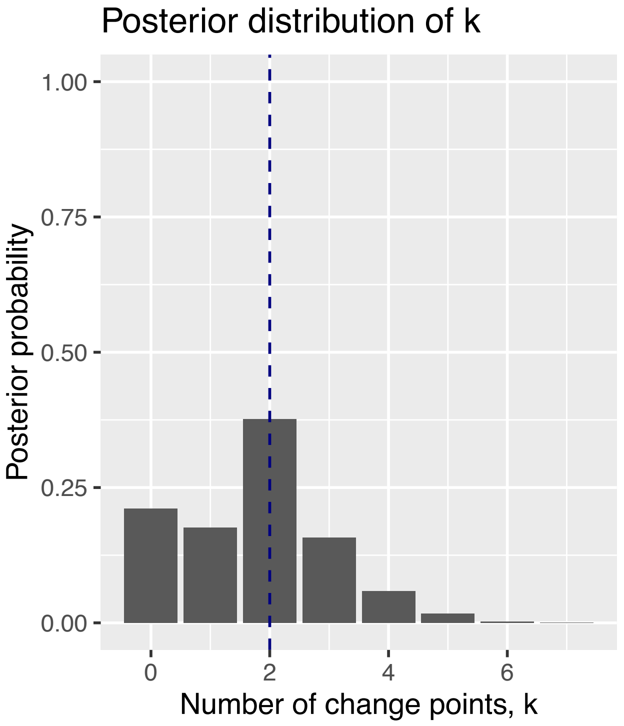

	**State 1**	**State 2**	**State 3**
Change point	8.0063	9.5453	NA
Velocity	0.3654	0.0735	0.3905
Std dev	0.0758	NA	NA

Kin1DDB particle: 63

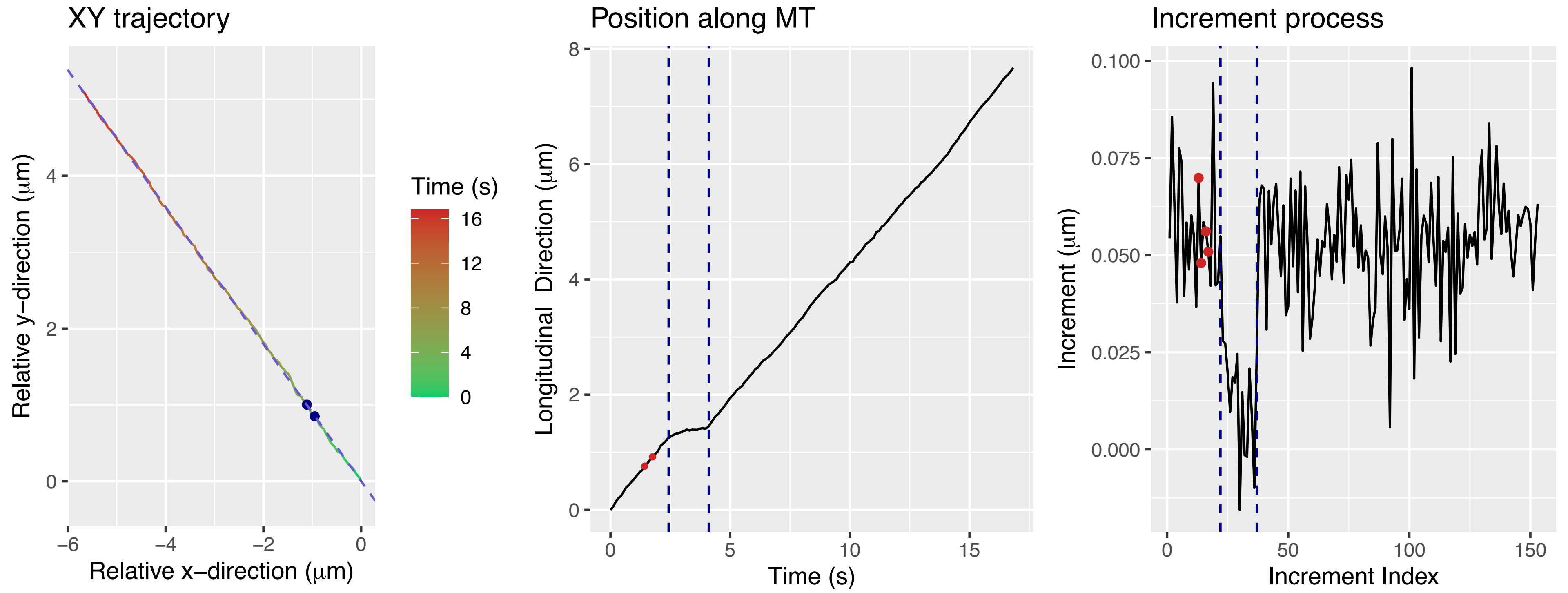

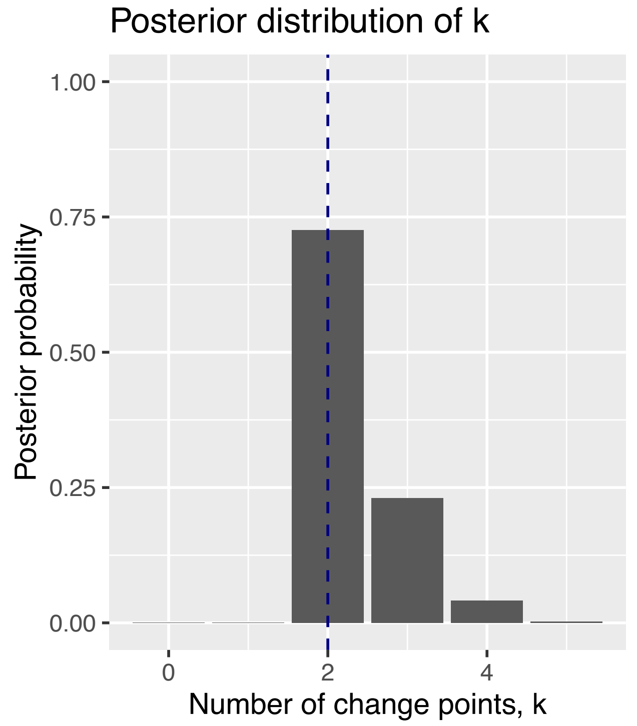

	State 1	State 2	State 3
Change point	2.4289	4.105	NA
Velocity	0.5181	0.0995	0.491
Std dev	0.0454	NA	NA

Kin1DDB particle: 64

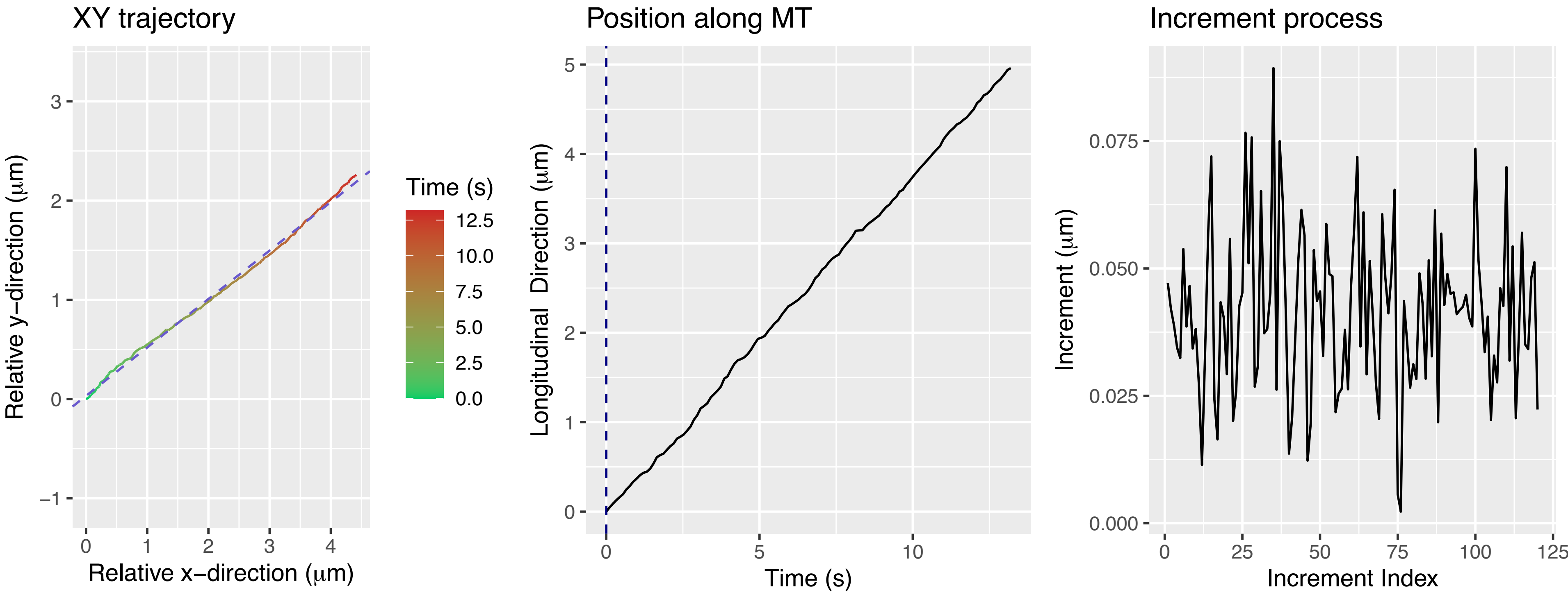

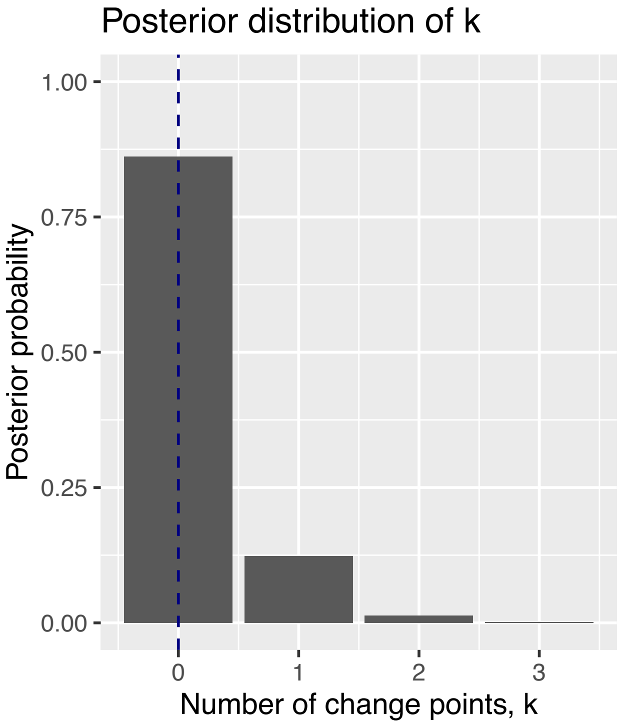

	State 1
Change point	0
Velocity	0.3765
Std dev	0.046

Kin1DDB particle: 65

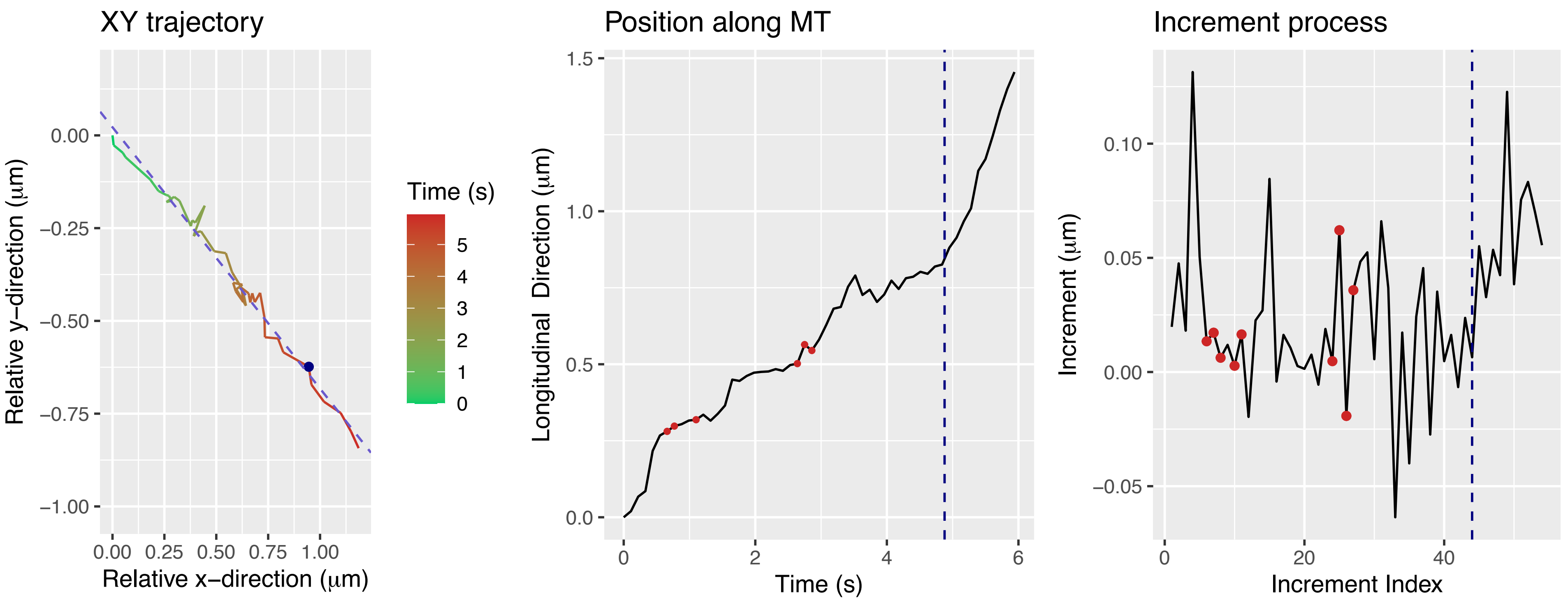

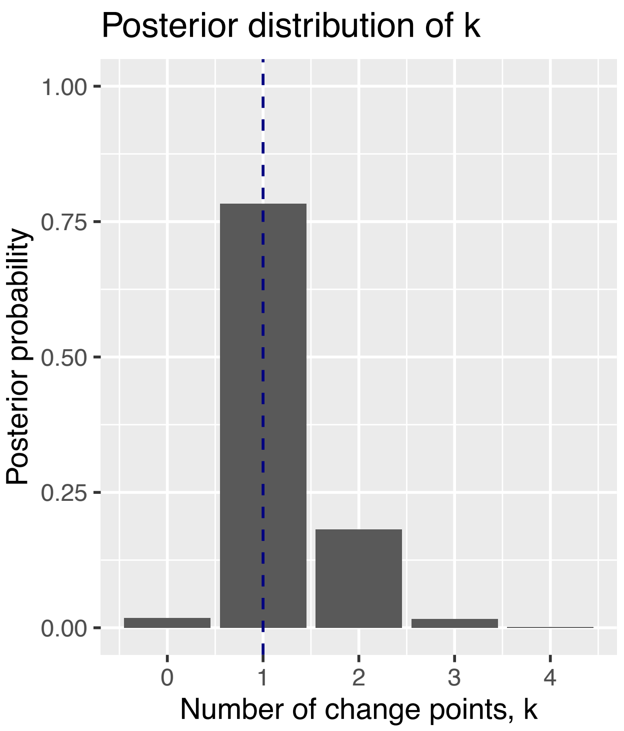

	State 1	State 2
Change point	4.8771	NA
Velocity	0.1691	0.5635
Std dev	0.0951	NA

Kin1DDB particle: 66

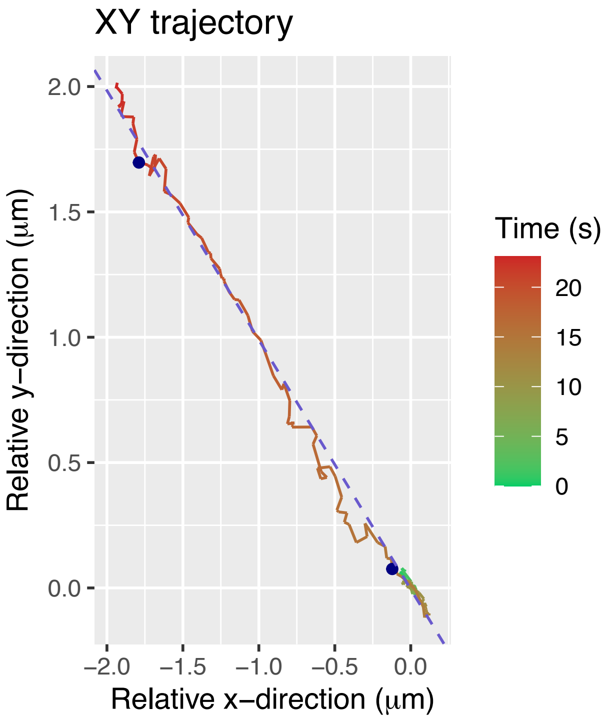

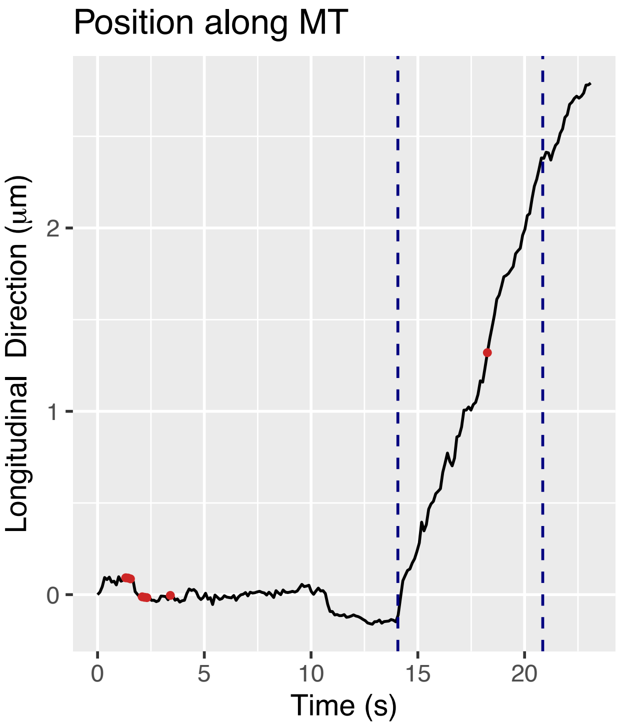

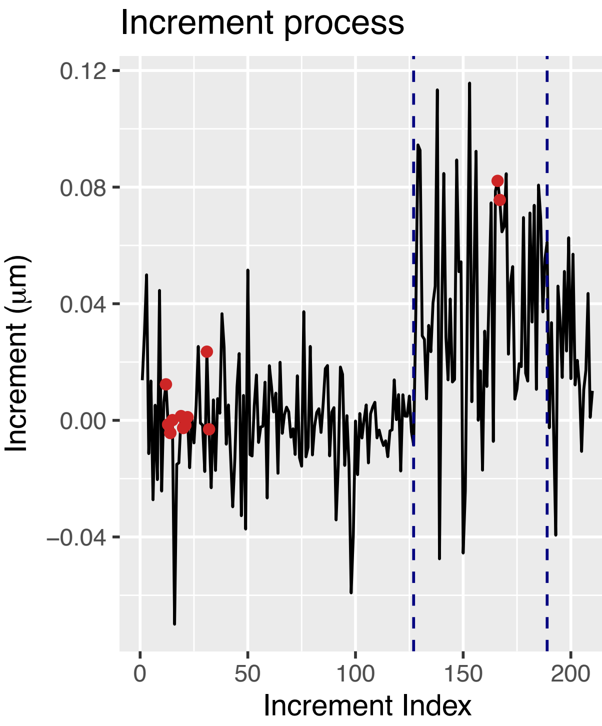

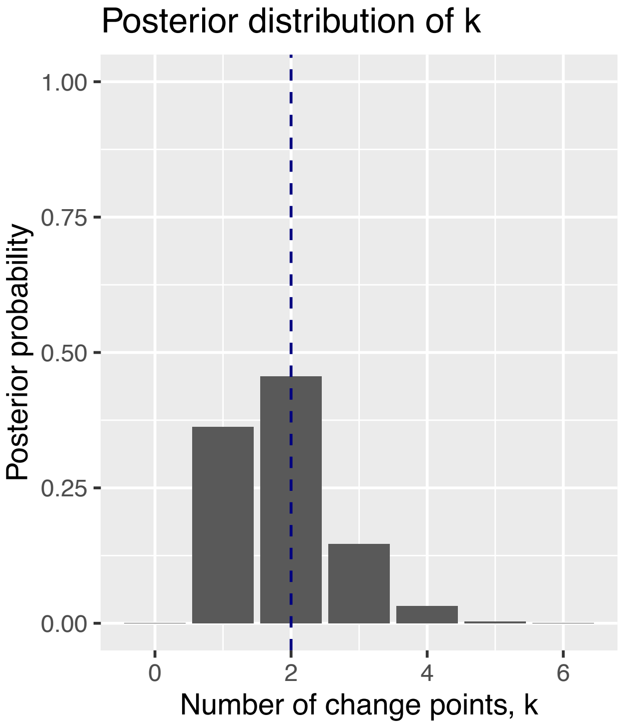

	State 1	State 2	State 3
Change point	14.0651	20.85	NA
Velocity	−0.0058	0.3711	0.1816
Std dev	0.0762	NA	NA

Kin1DDB particle: 67

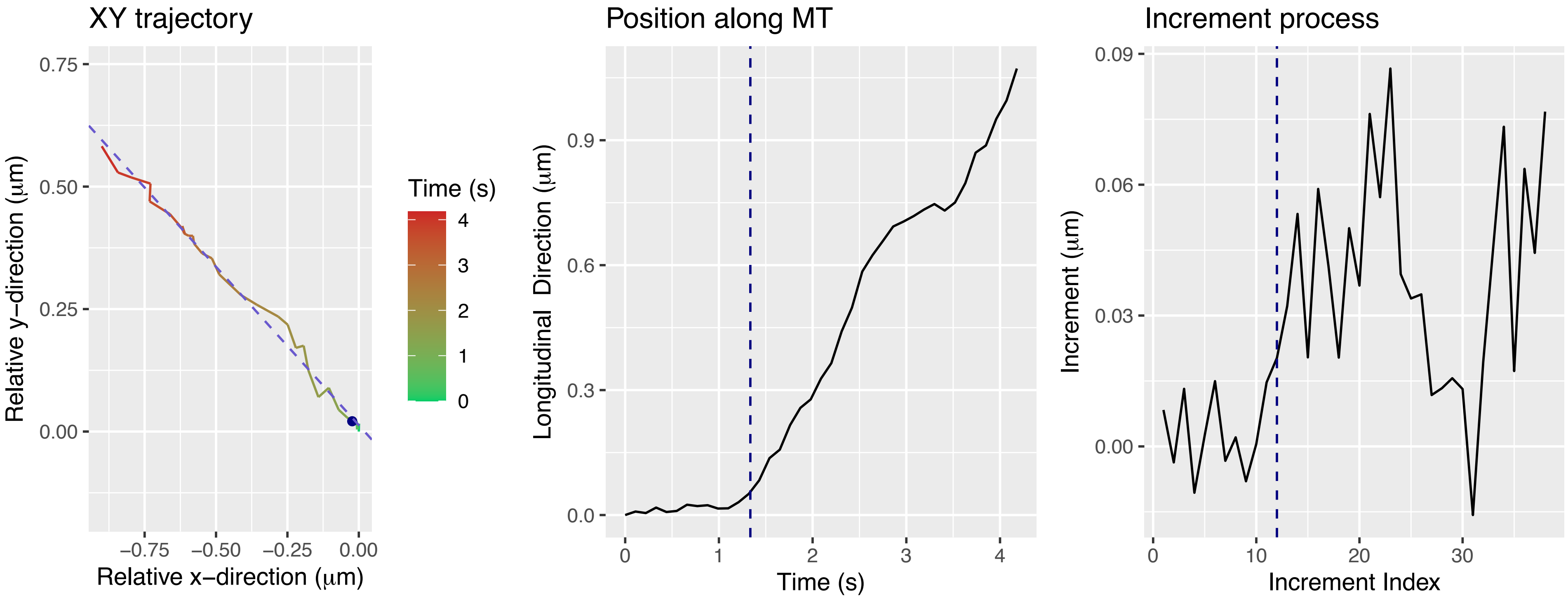

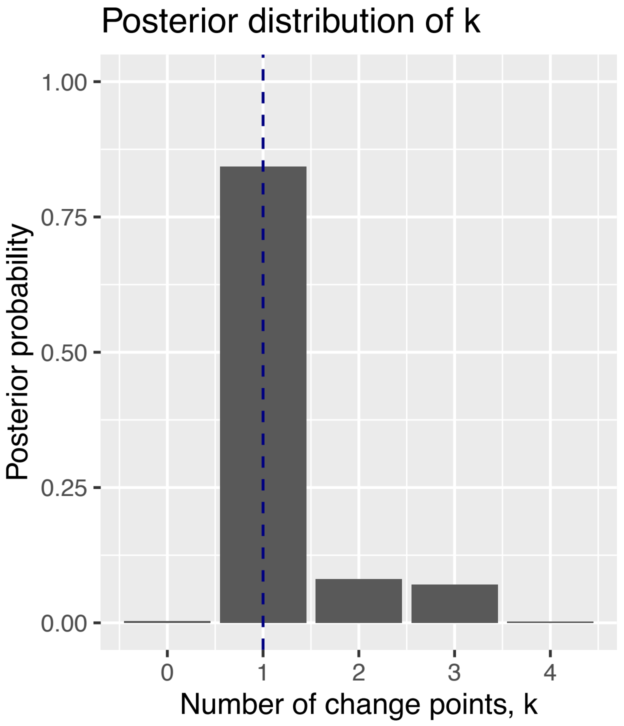

	State 1	State 2
Change point	1.3355	NA
Velocity	0.0265	0.3608
Std dev	0.0649	NA

Kin1DDB particle: 68

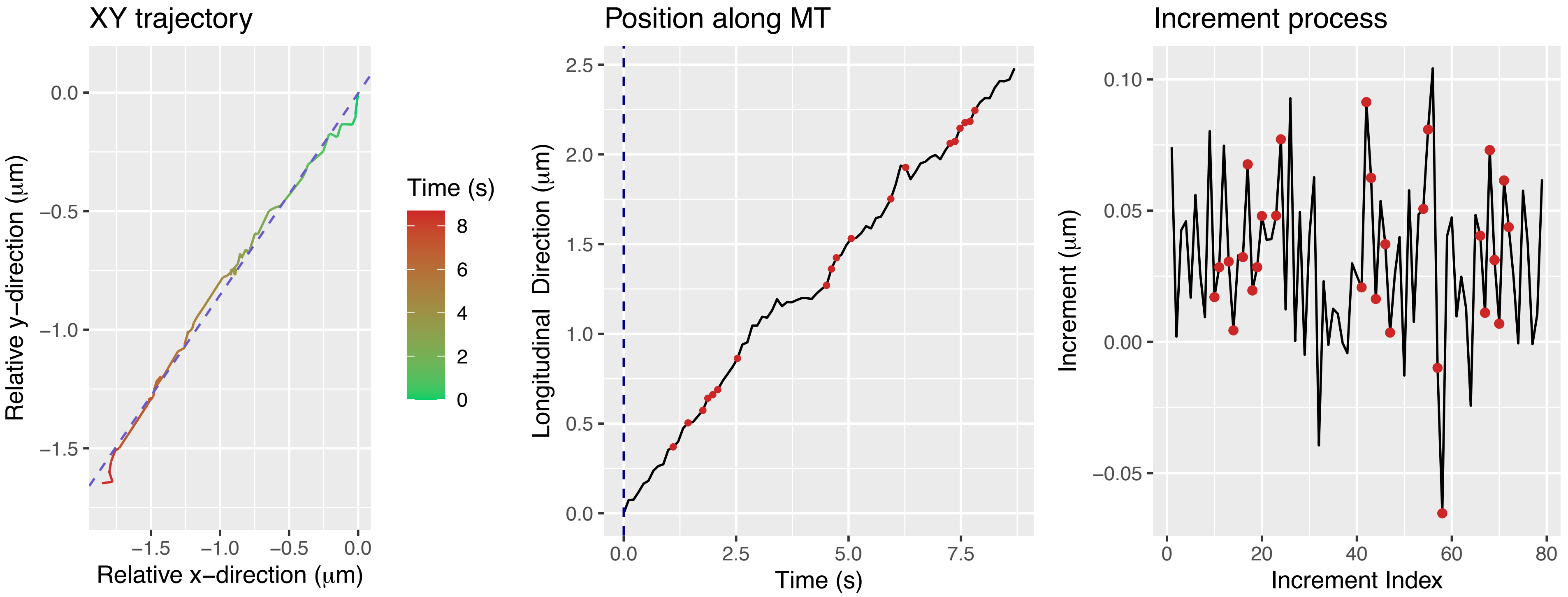

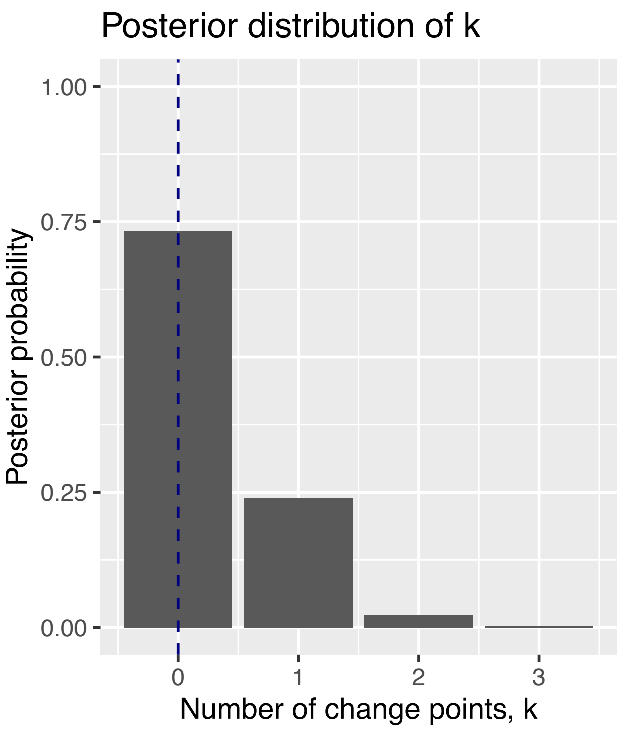

	State 1
Change point	0
Velocity	0.2841
Std dev	0.0916

Kin1DDB particle: 69

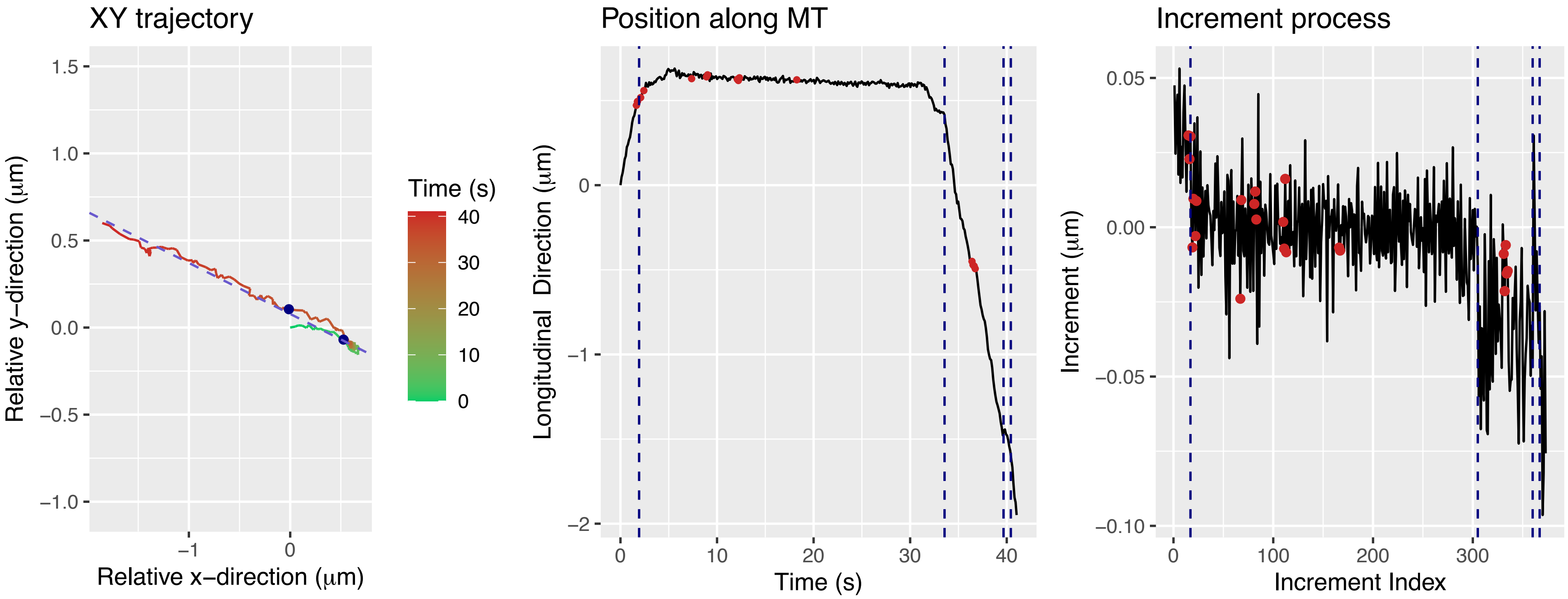

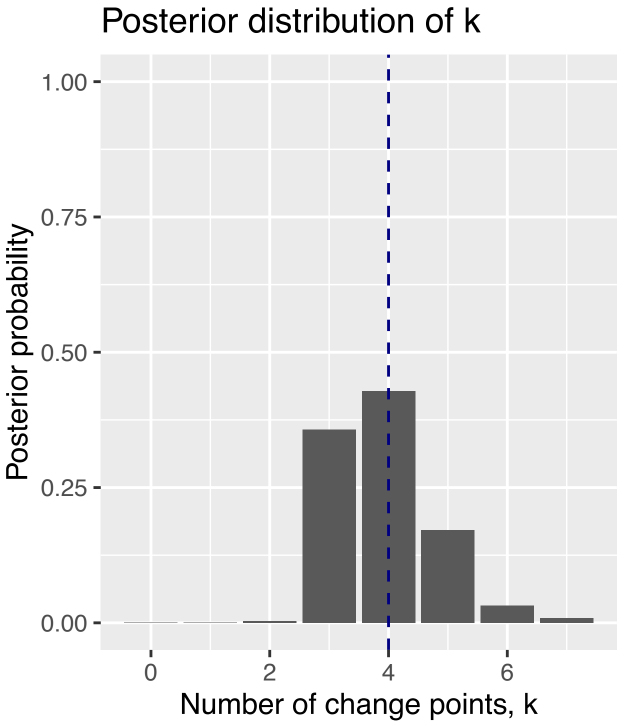

	State 1	State 2	State 3	State 4	State 5
Change point	1.9296	33.5619	39.669	40.4255	NA
Velocity	0.2802	−0.0061	−0.3014	−0.1108	−0.5948
Std dev	0.0455	NA	NA	NA	NA

Kin1DDB particle: 70

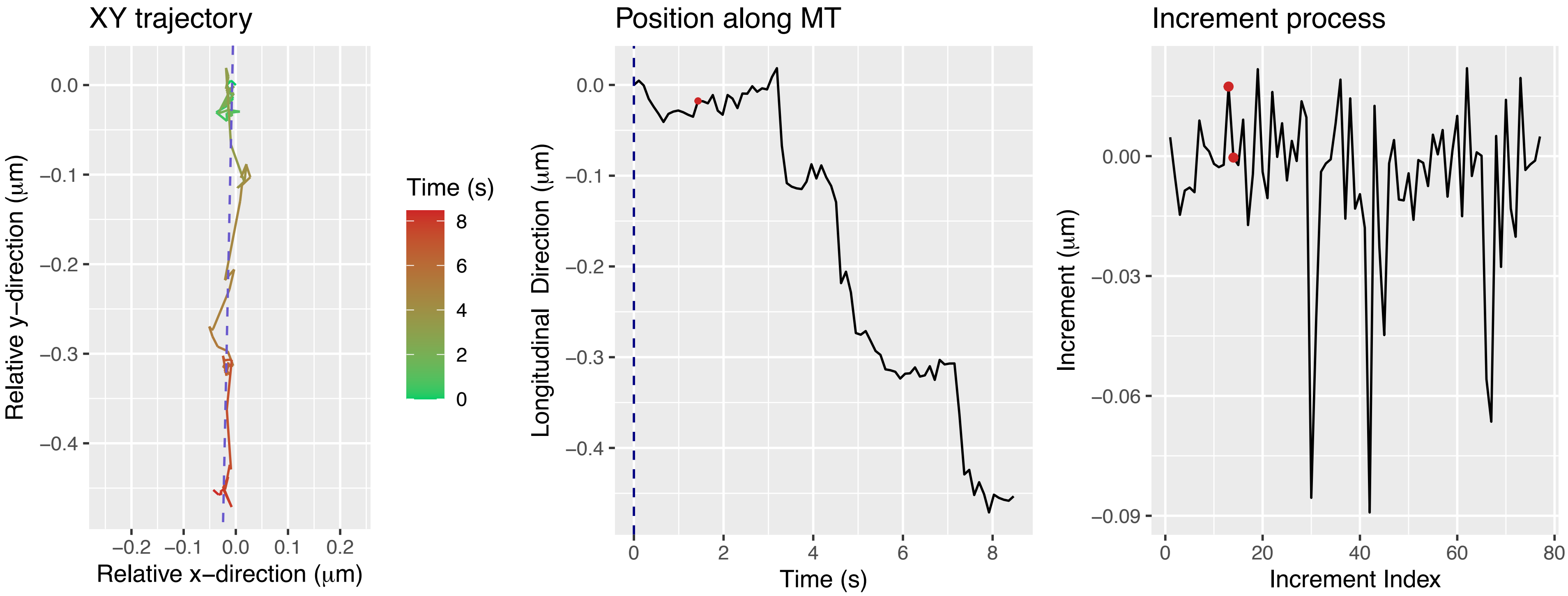

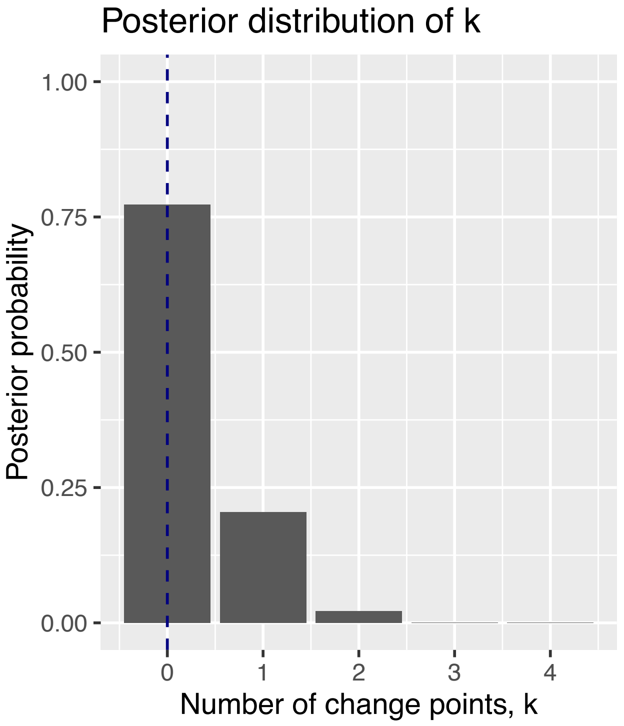

	State 1
Change point	0
Velocity	−0.0535
Std dev	0.0599

Kin1DDB particle: 71

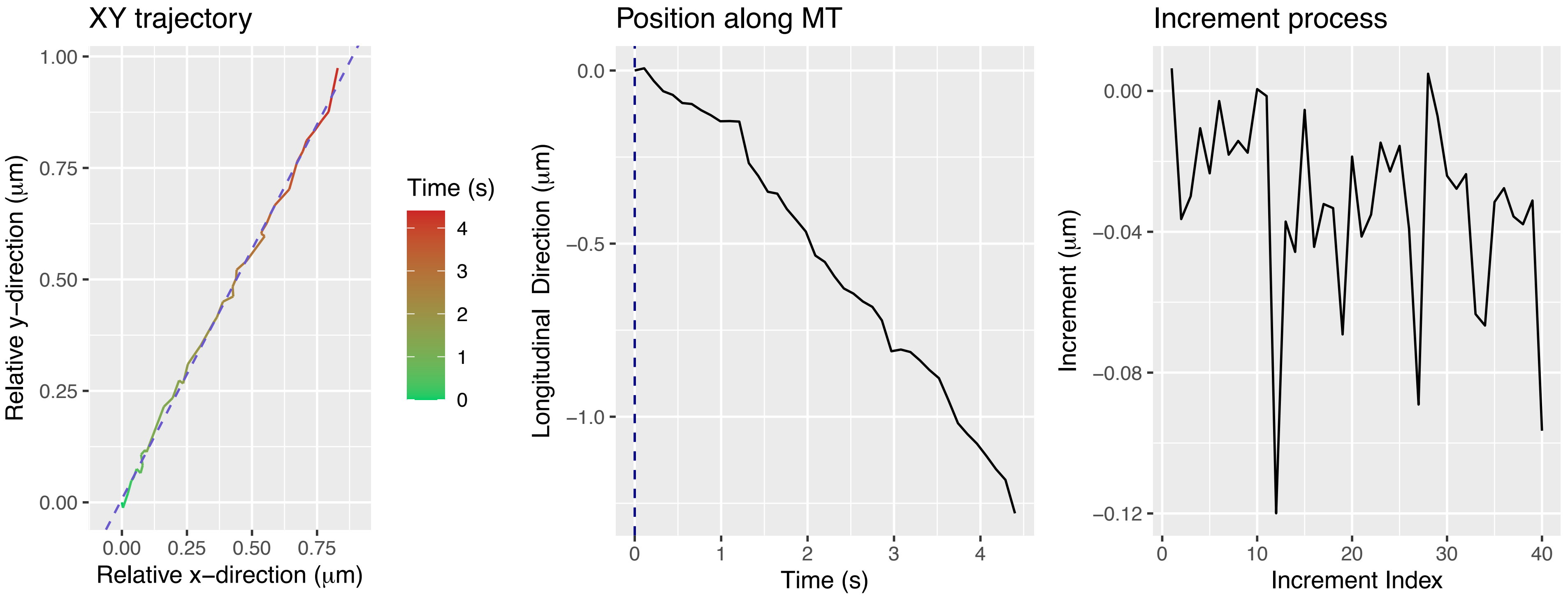

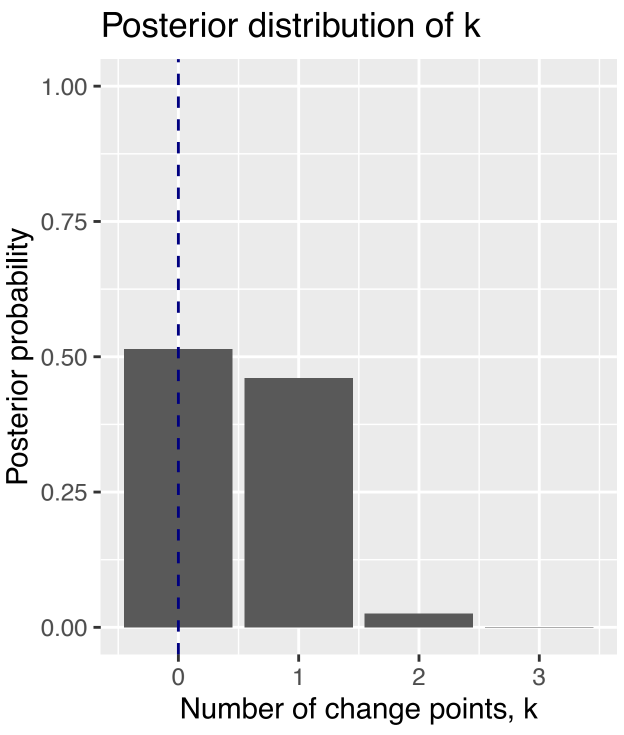

	State 1
Change point	0
Velocity	−0.291
Std dev	0.0785

Kin1DDB particle: 72

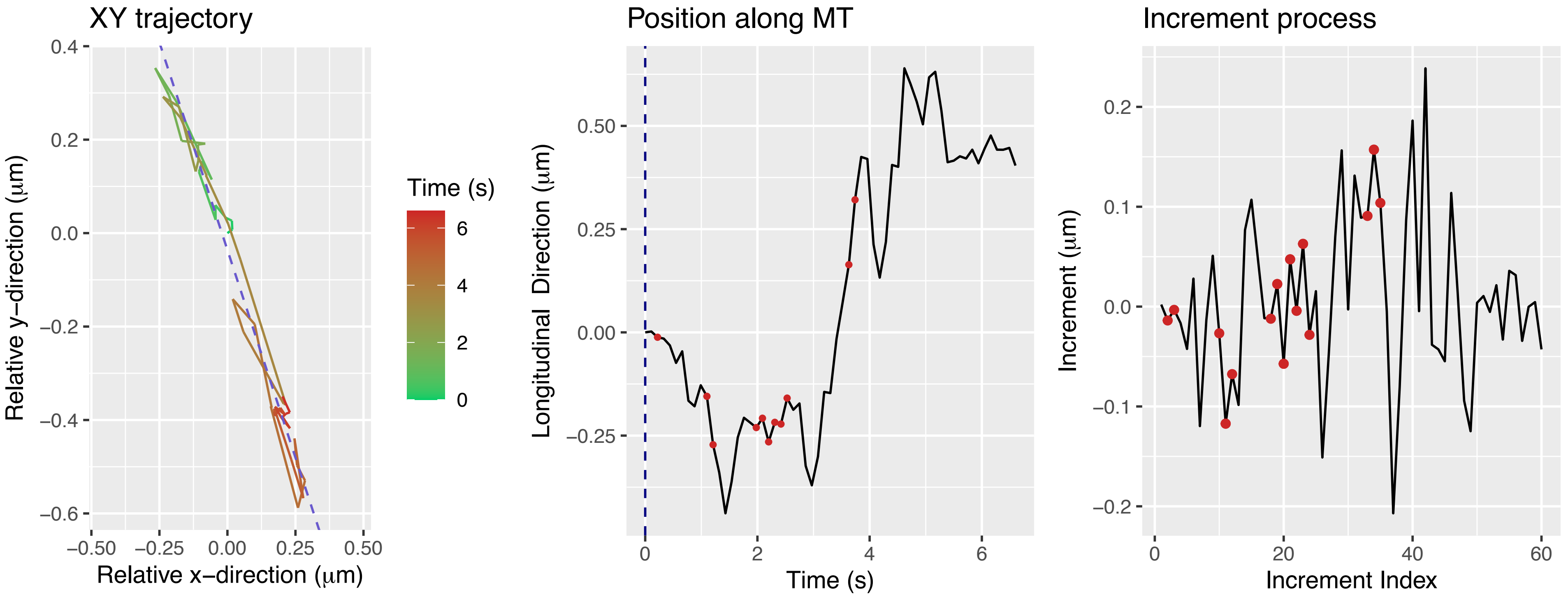

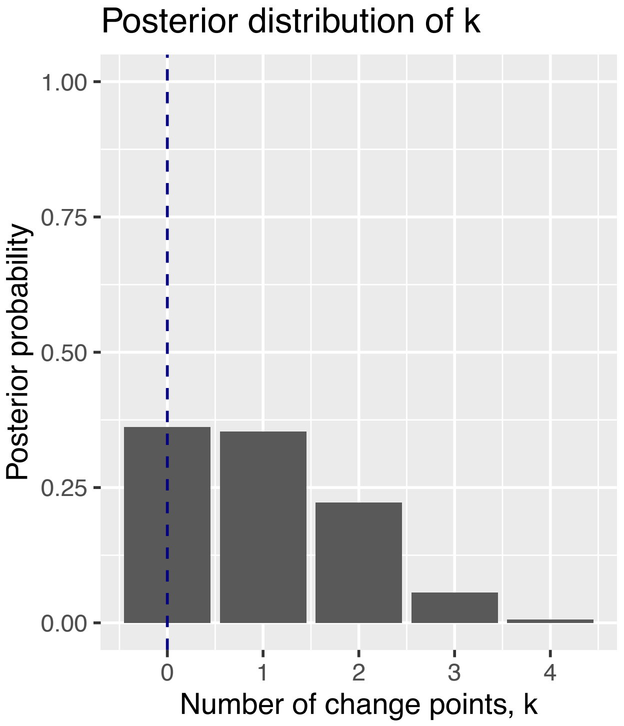

	State 1
Change point	0
Velocity	0.0584
Std dev	0.2496

Kin1DDB particle: 73

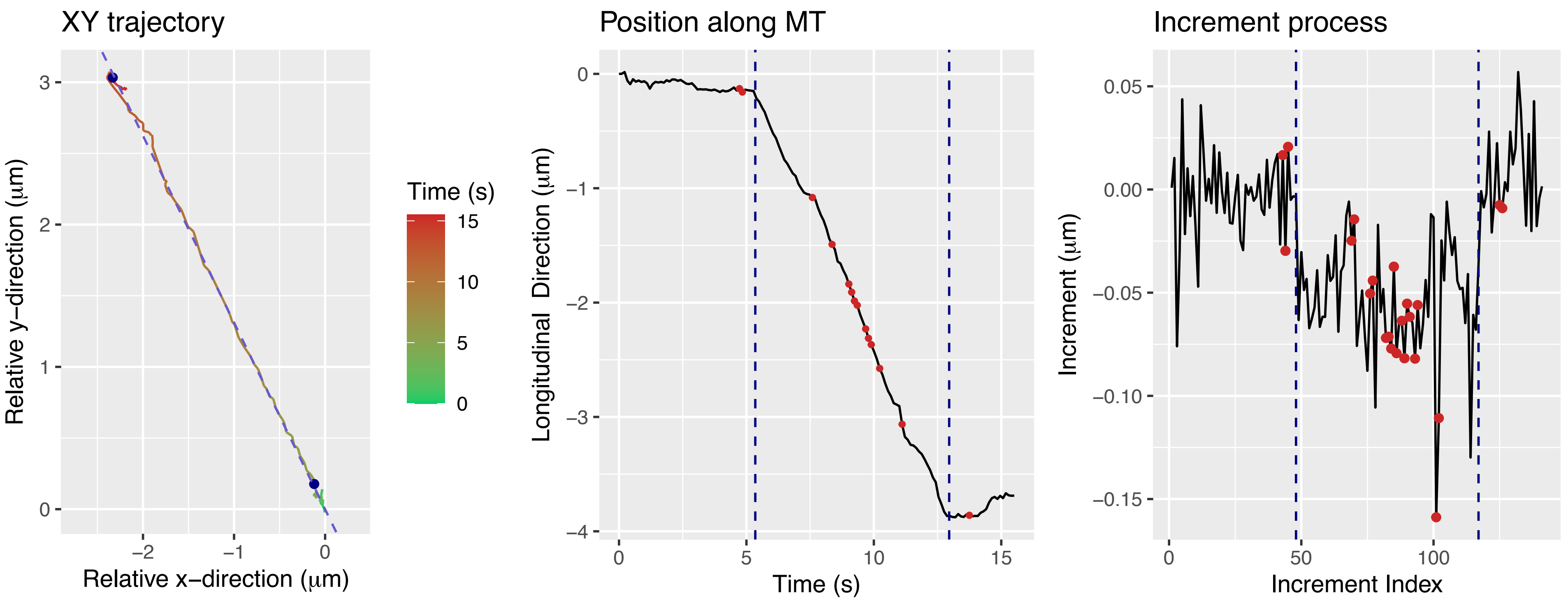

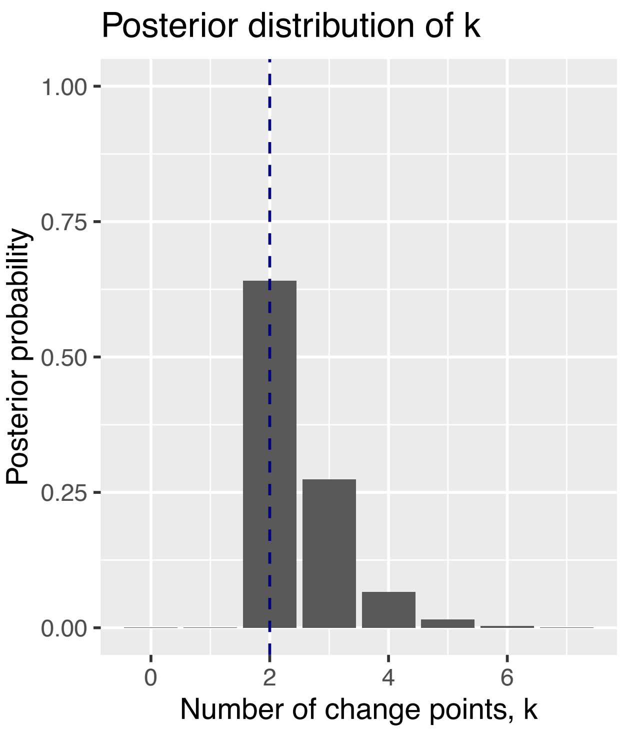

	State 1	State 2	State 3
Change point	5.3476	12.9561	NA
Velocity	−0.027	−0.4837	0.0626
Std dev	0.0738	NA	NA

Kin1DDB particle: 74

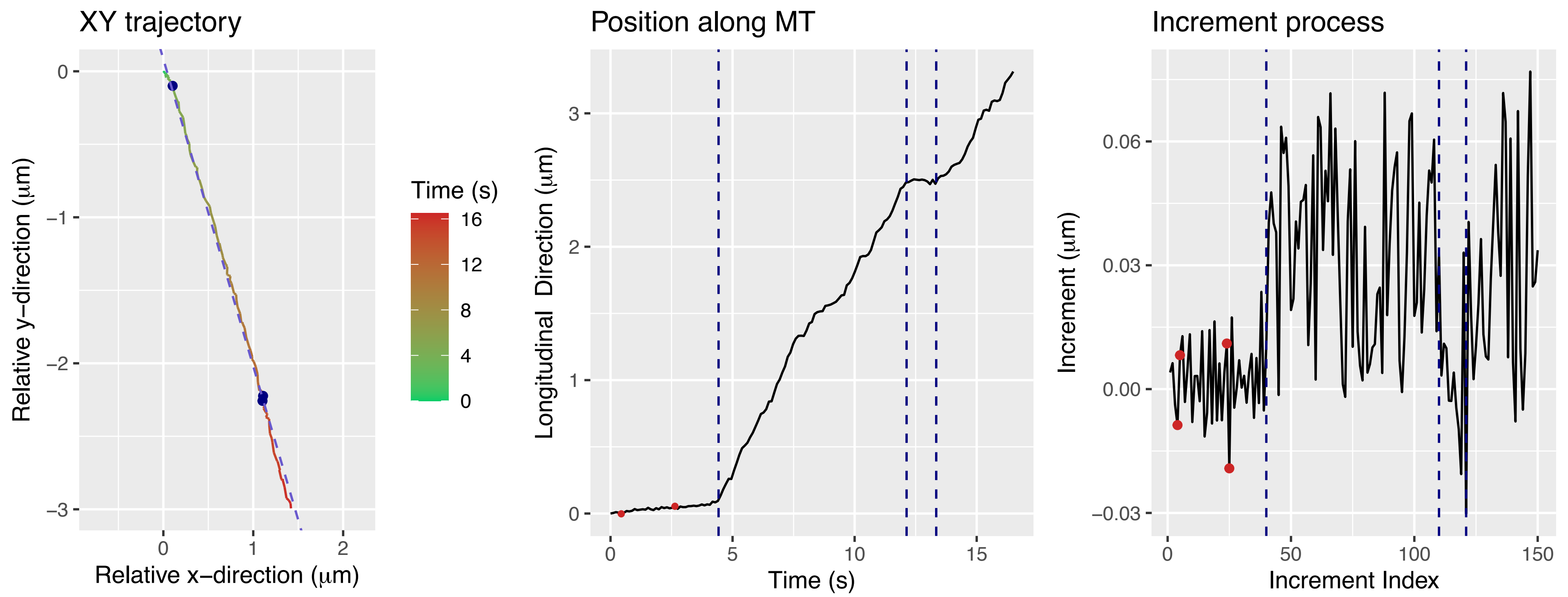

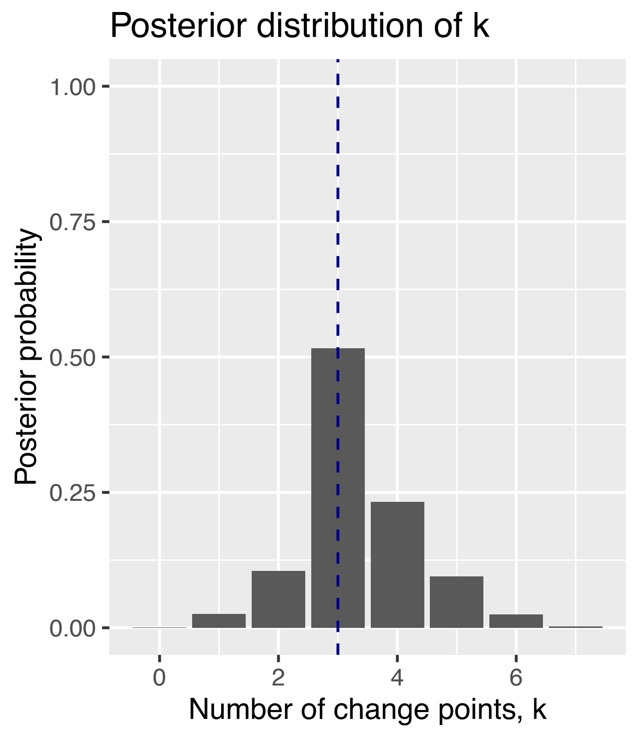

	State 1	State 2	State 3	State 4
Change point	4.4257	12.1287	13.3425	NA
Velocity	0.0035	0.3037	0.0152	0.2789
Std dev	0.0588	NA	NA	NA

Kin1DDB particle: 75

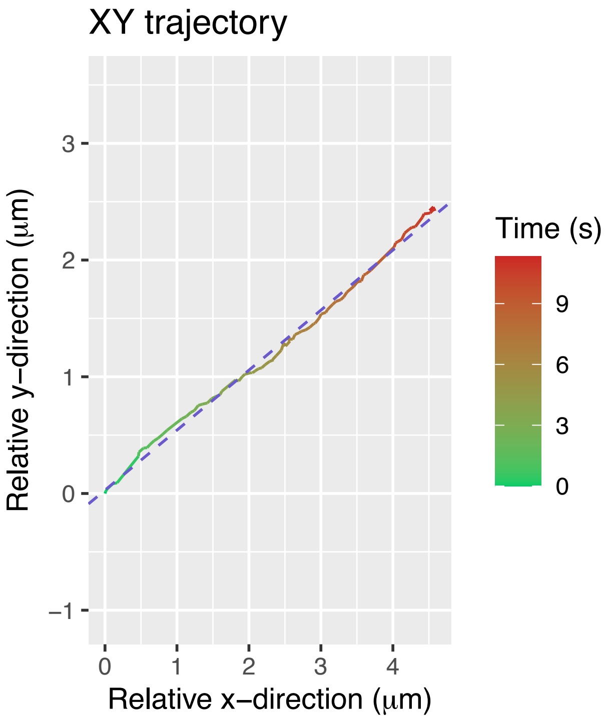

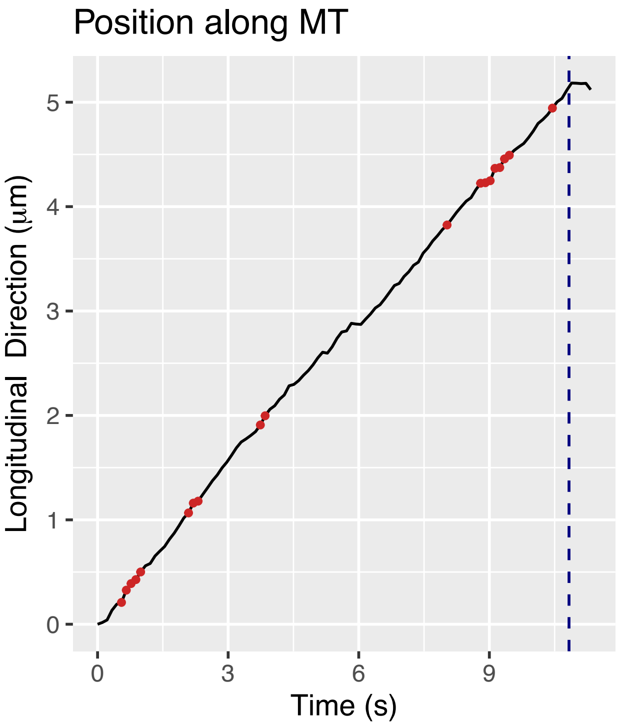

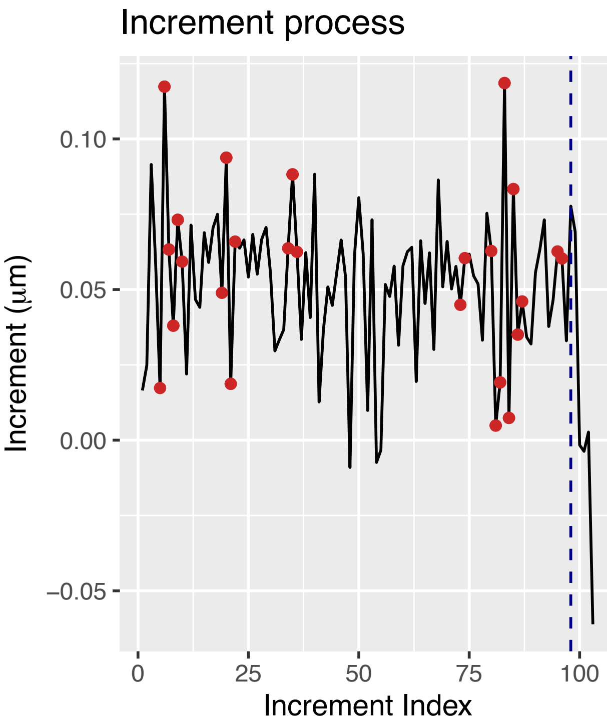

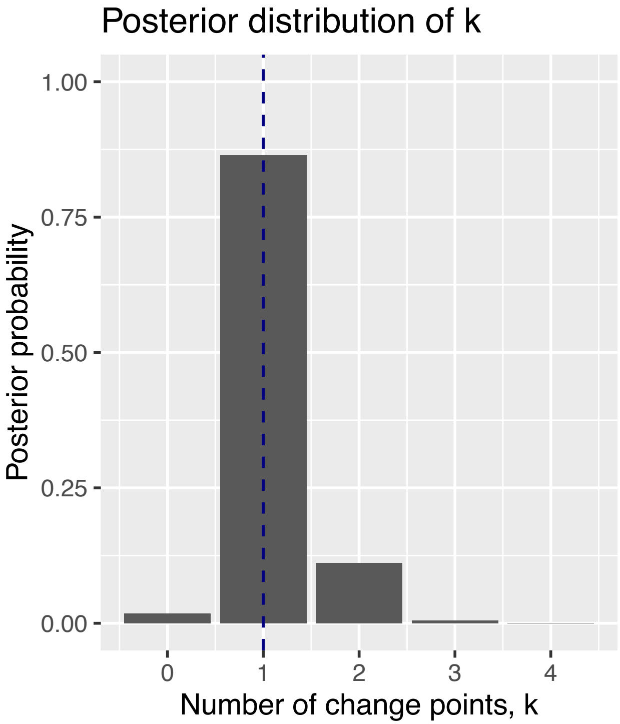

	State 1	State 2
Change point	10.8303	NA
Velocity	0.4717	0.0052
Std dev	0.0759	NA

Kin1DDB particle: 76

XY trajectory

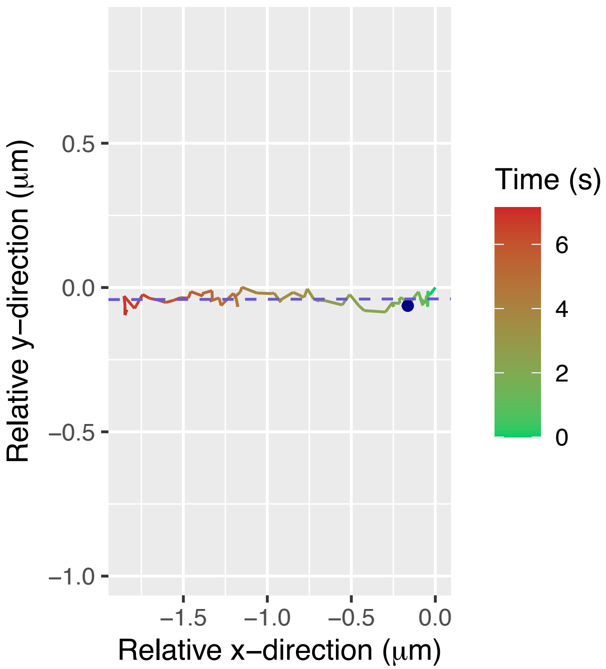

Position along MT

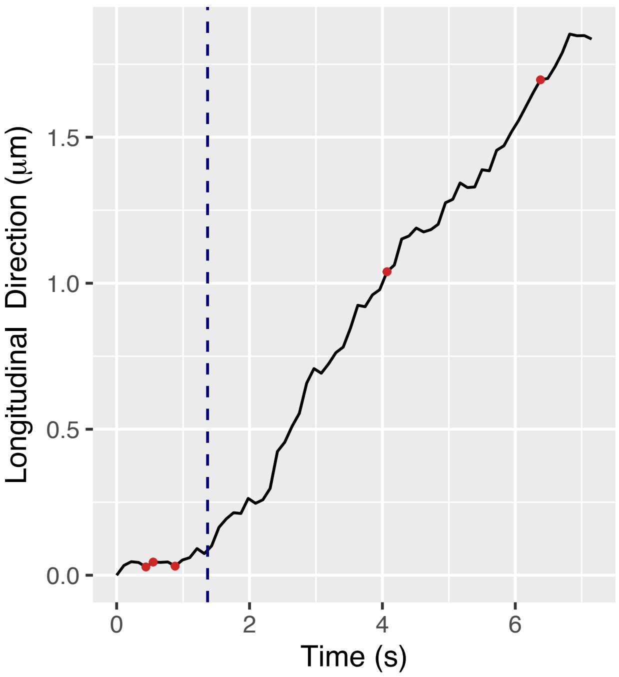

Increment process

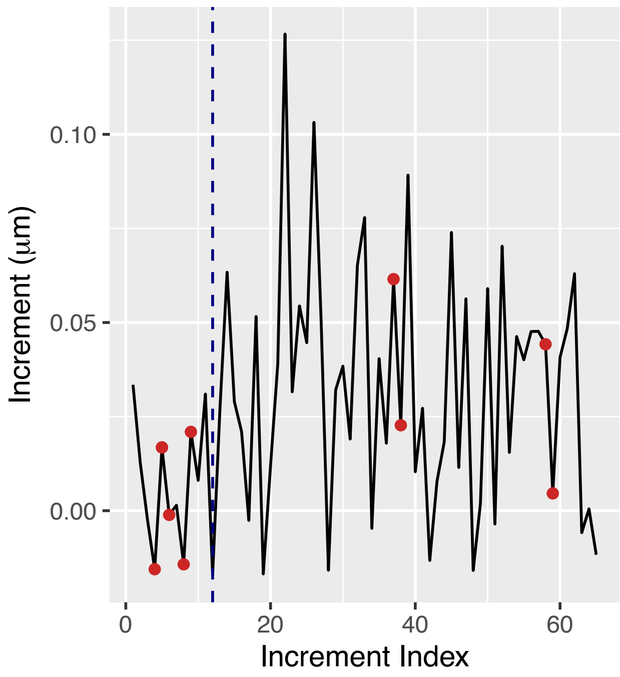

Posterior distribution of k

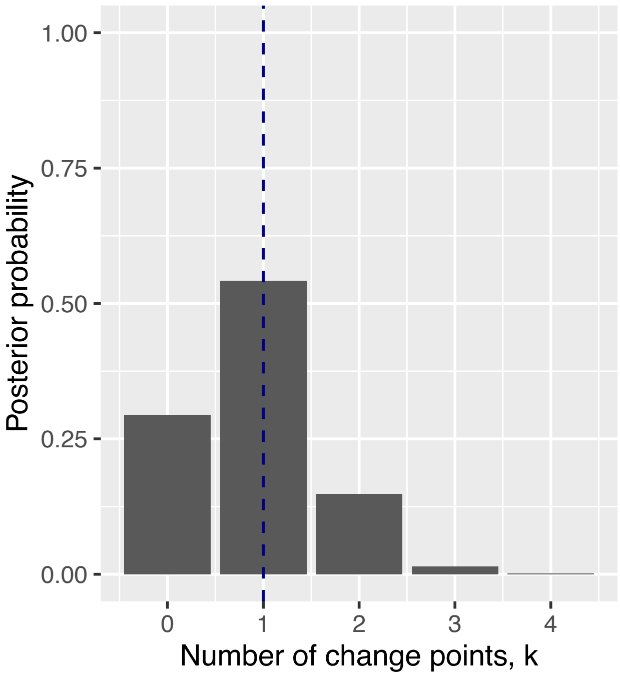

	State 1	State 2
Change point	1.3685	NA
Velocity	0.0718	0.3036
Std dev	0.0908	NA

Kin1DDB particle: 77

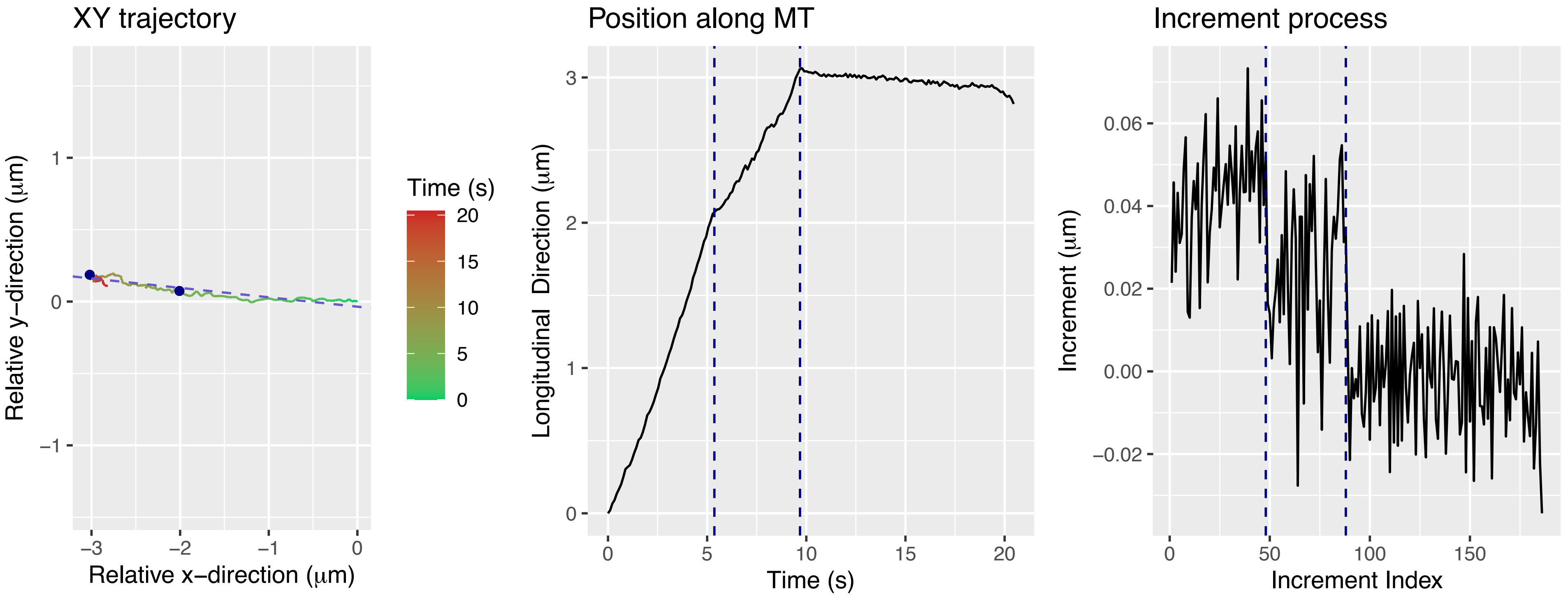

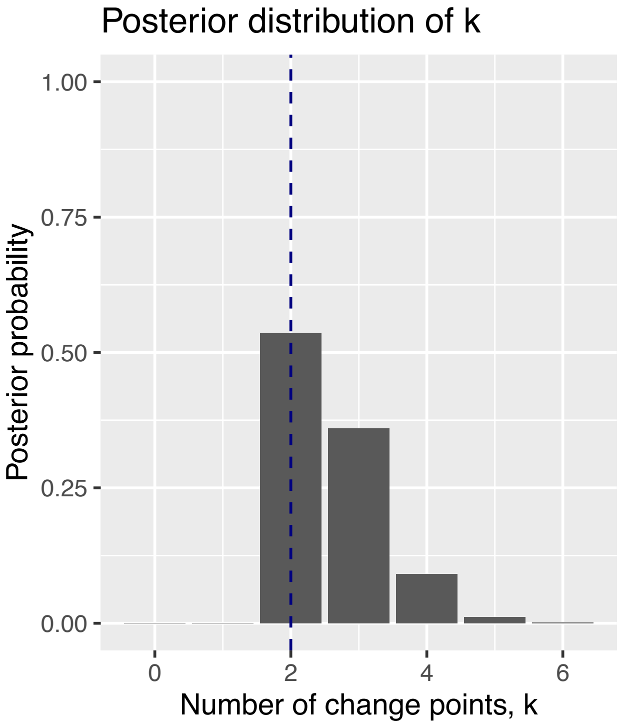

	State 1	State 2	State 3
Change point	5.3631	9.6821	NA
Velocity	0.3879	0.2271	−0.0186
Std dev	0.0453	NA	NA

Kin1DDB particle: 78

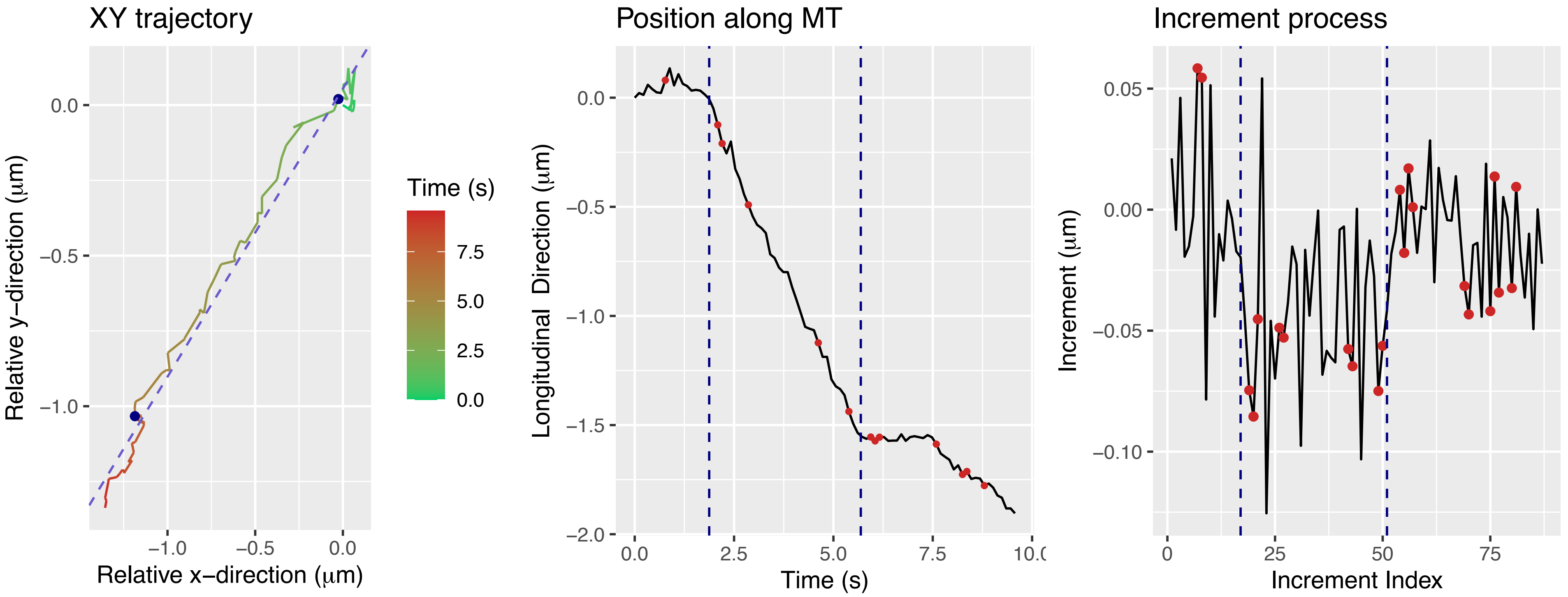

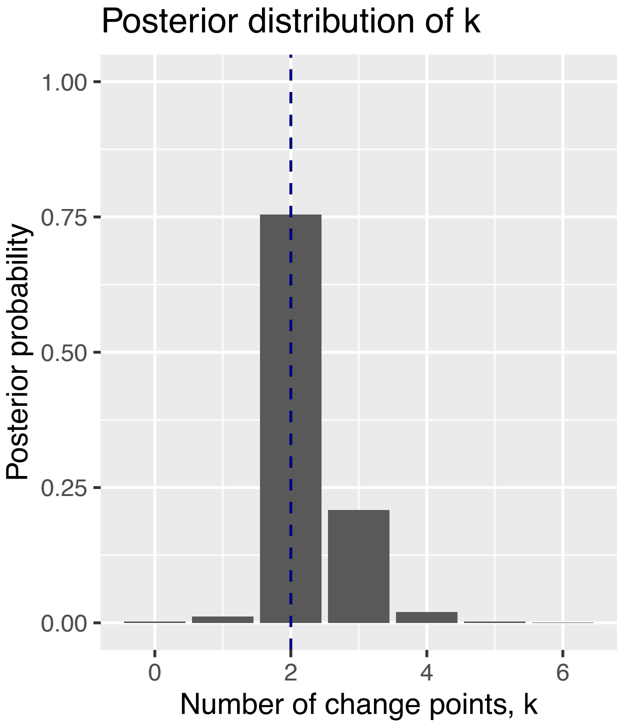

	State 1	State 2	State 3
Change point	1.8744	5.6893	NA
Velocity	0.0134	−0.4113	−0.0774
Std dev	0.0894	NA	NA

Kin1DDB particle: 79

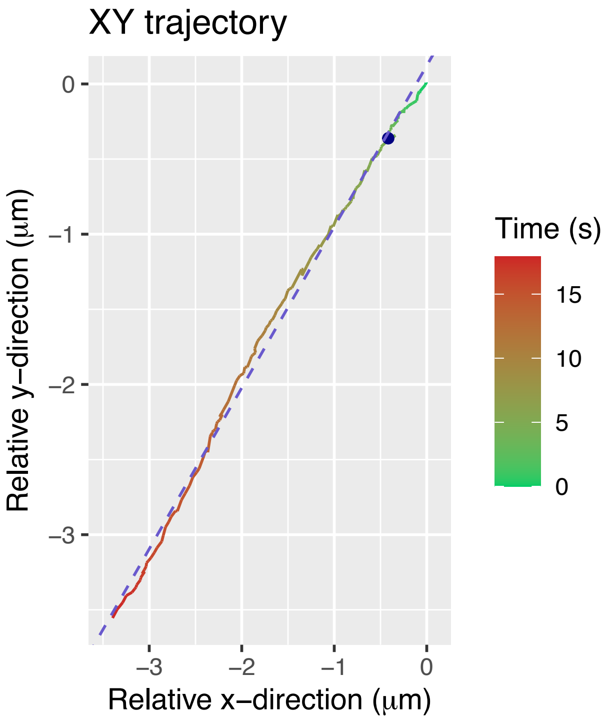

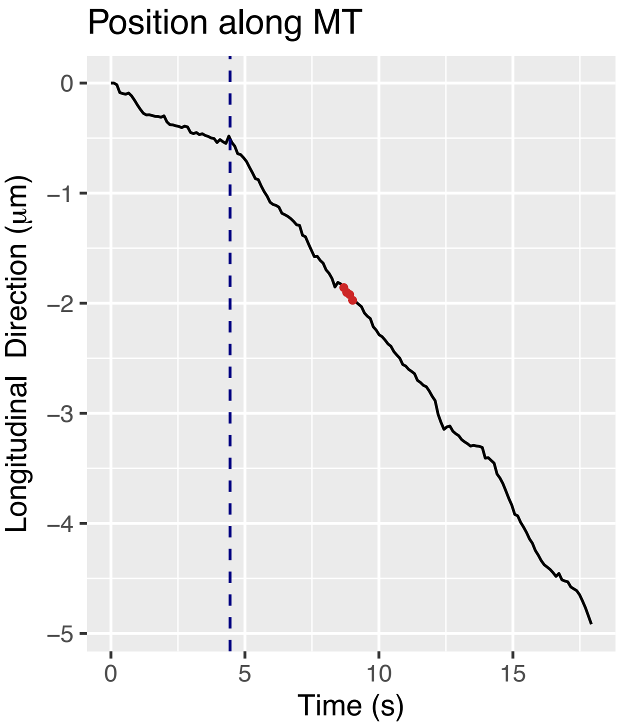

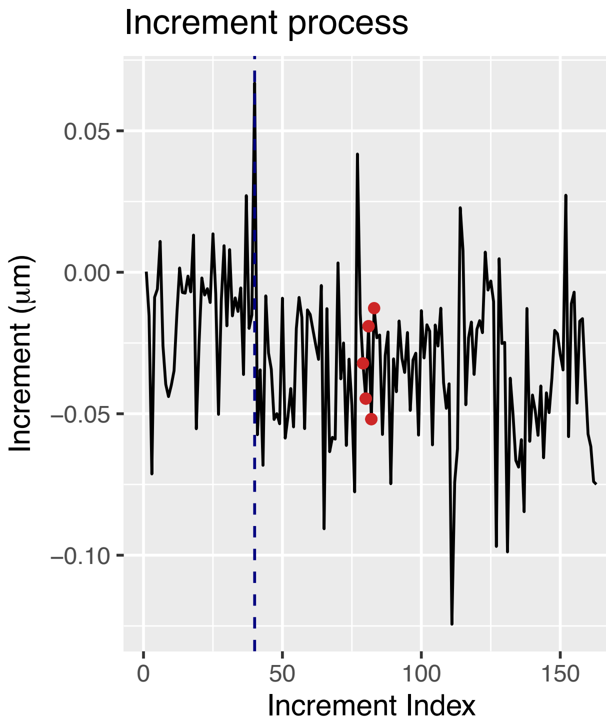

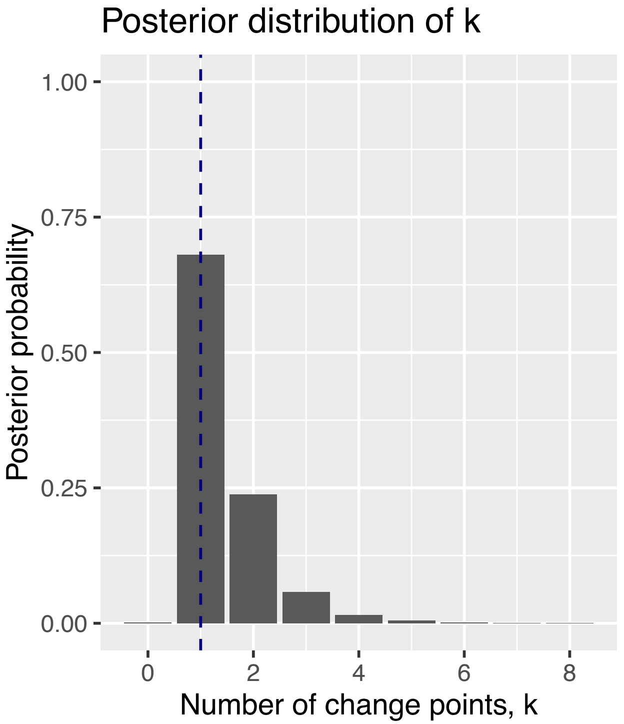

	State 1	State 2
Change point	4.4457	NA
Velocity	−0.1125	−0.328
Std dev	0.0772	NA

Kin1DDB particle: 80

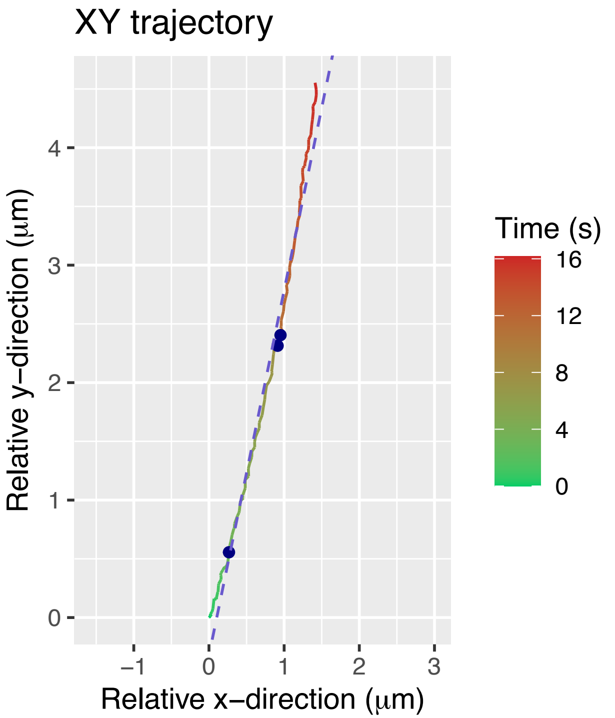

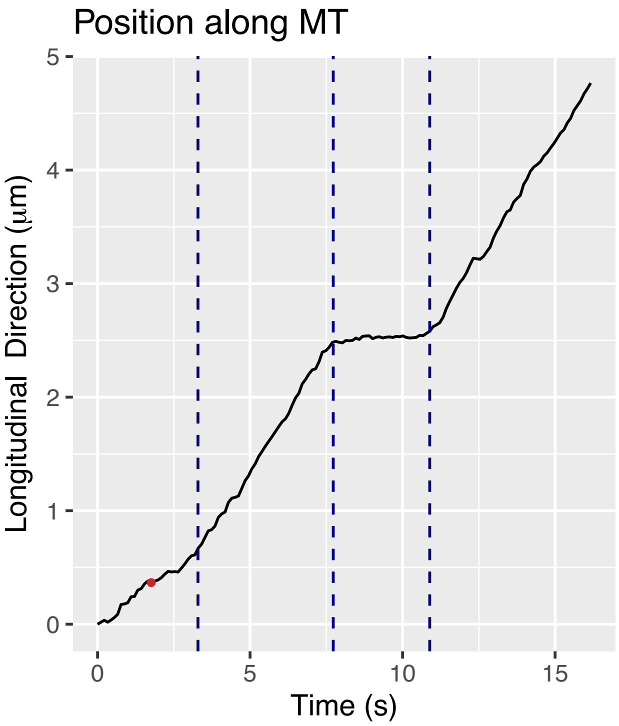

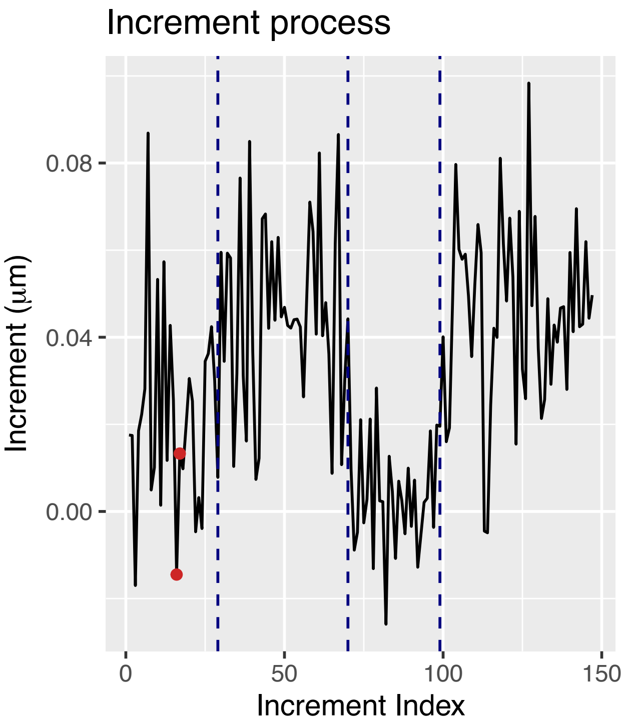

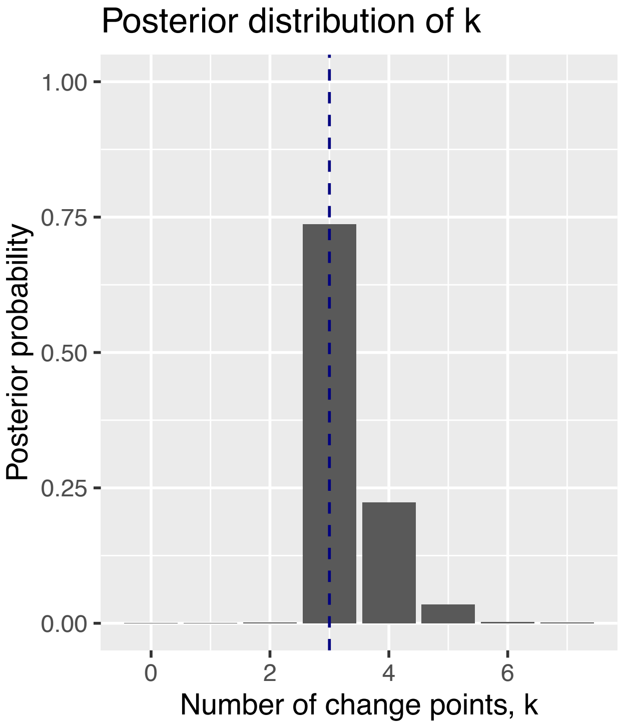

	State 1	State 2	State 3	State 4
Change point	3.2916	7.7273	10.8902	NA
Velocity	0.2018	0.4053	0.0256	0.4165
Std dev	0.0592	NA	NA	NA

Kin1DDB particle: 81

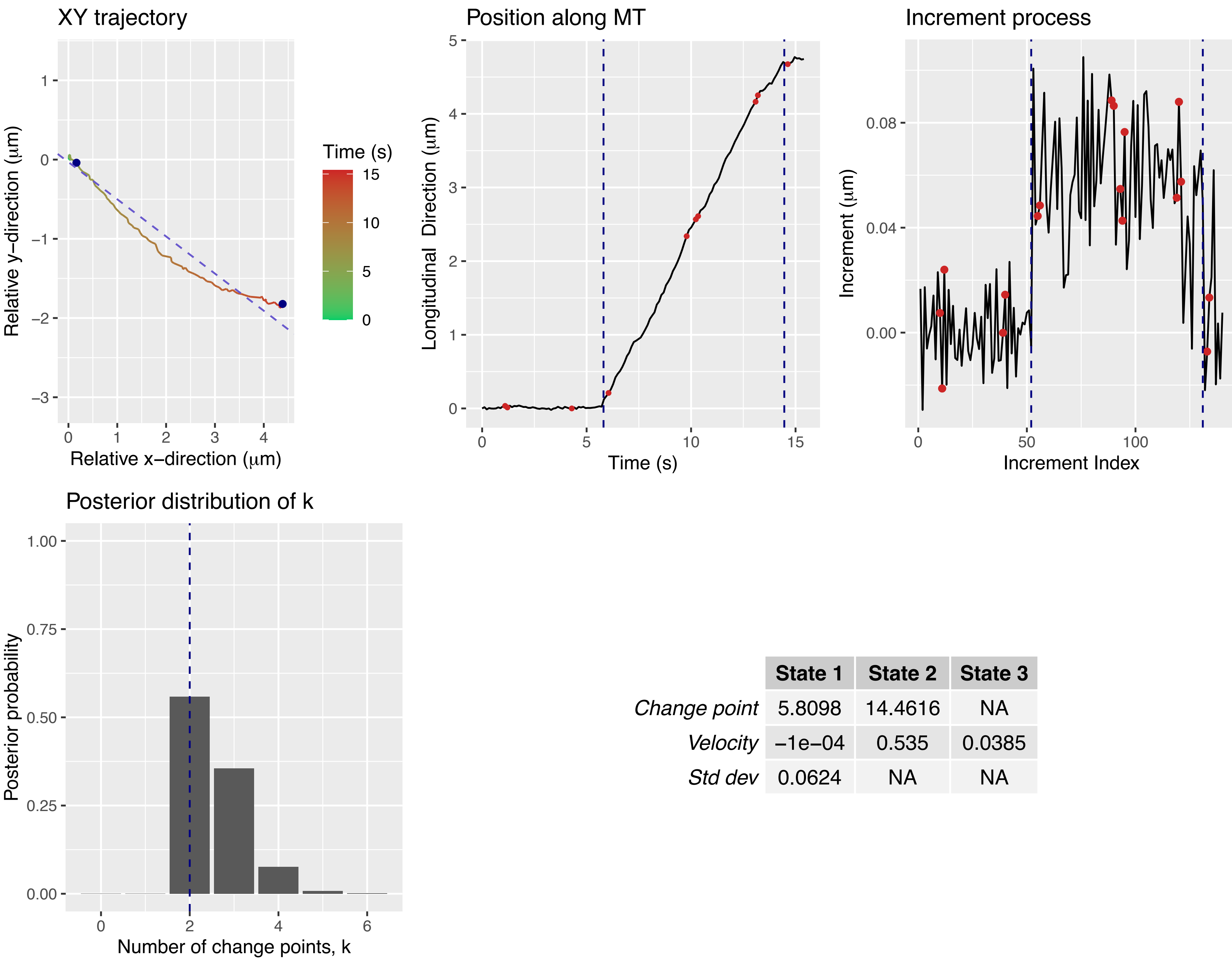

	State 1	State 2	State 3
Change point	5.8098	14.4616	NA
Velocity	−1e−04	0.535	0.0385
Std dev	0.0624	NA	NA

Kin1DDB particle: 82

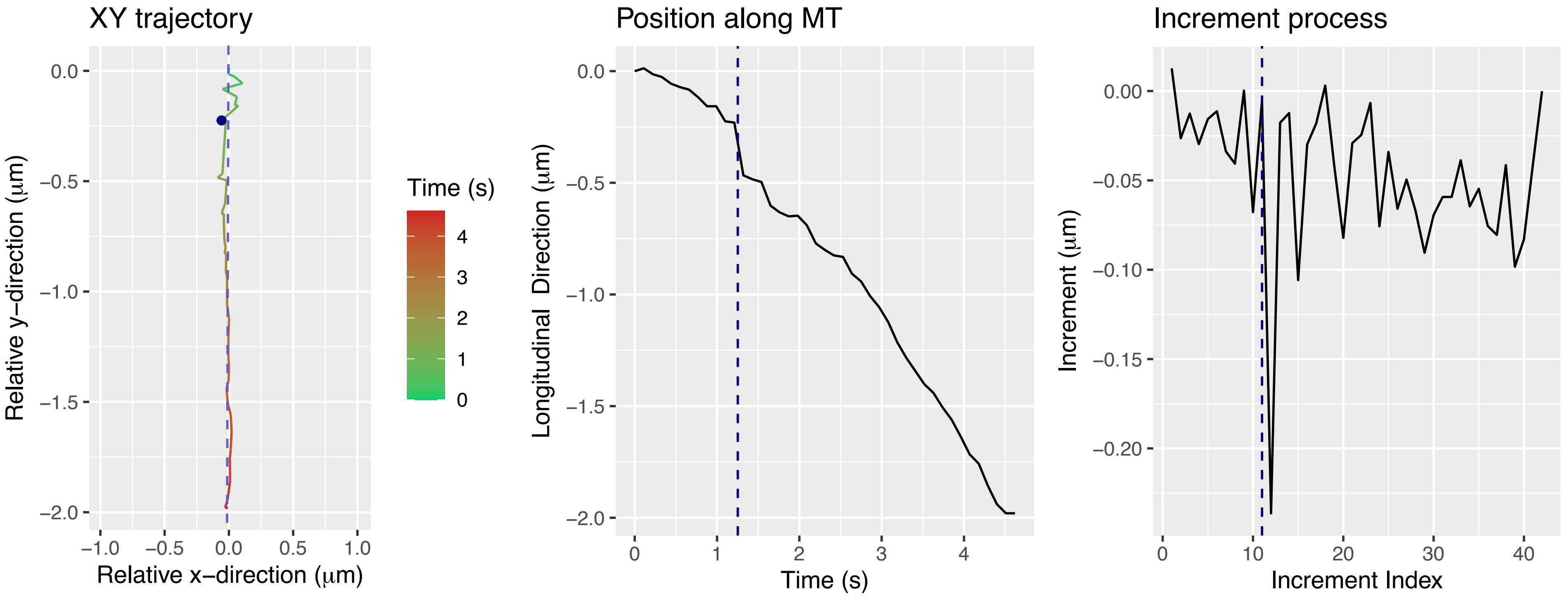

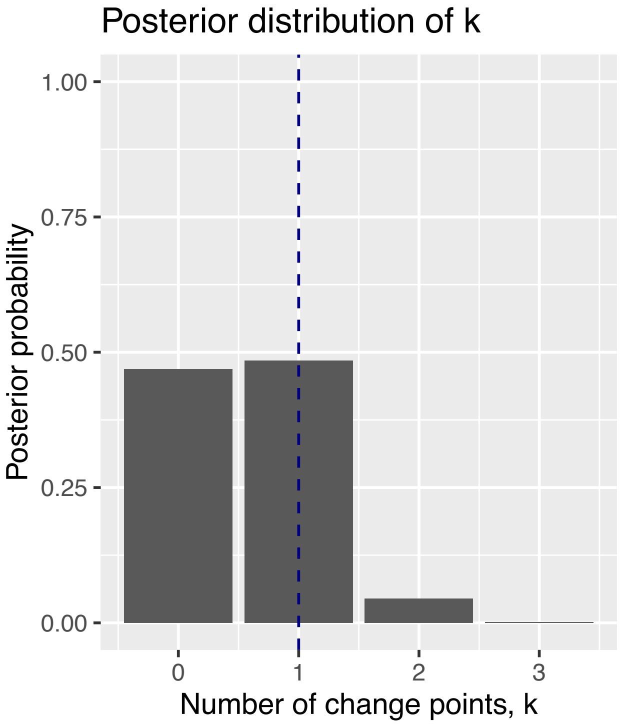

	**State 1**	**State 2**
Change point	1.252	NA
Velocity	−0.1957	−0.5089
Std dev	0.1203	NA

Kin1DDB particle: 83

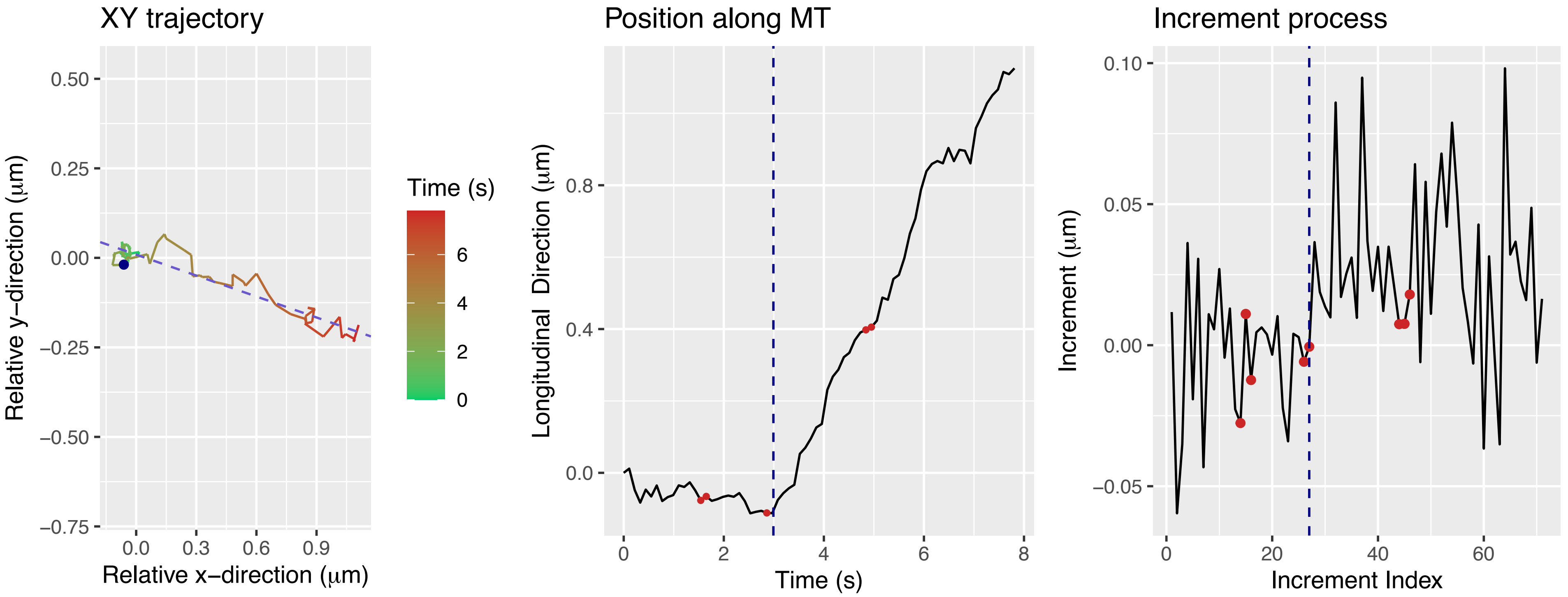

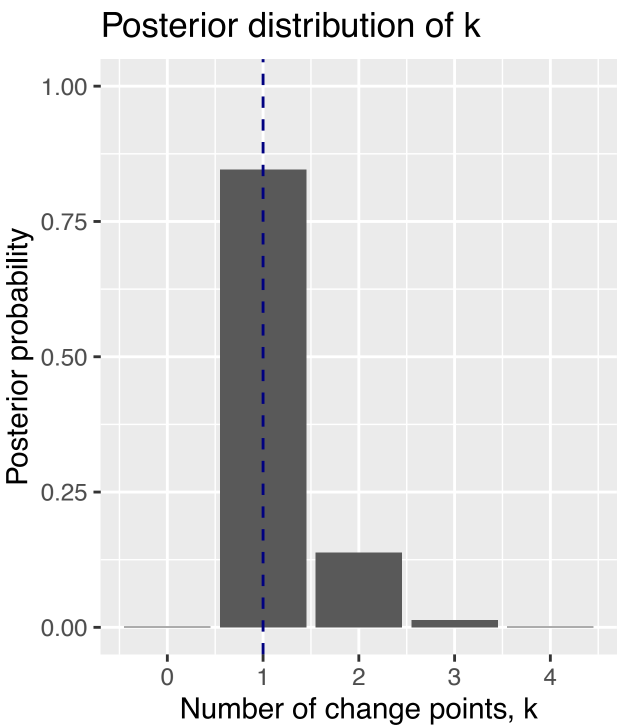

	State 1	State 2
Change point	2.9913	NA
Velocity	−0.0237	0.2559
Std dev	0.0816	NA

Kin1DDB particle: 84

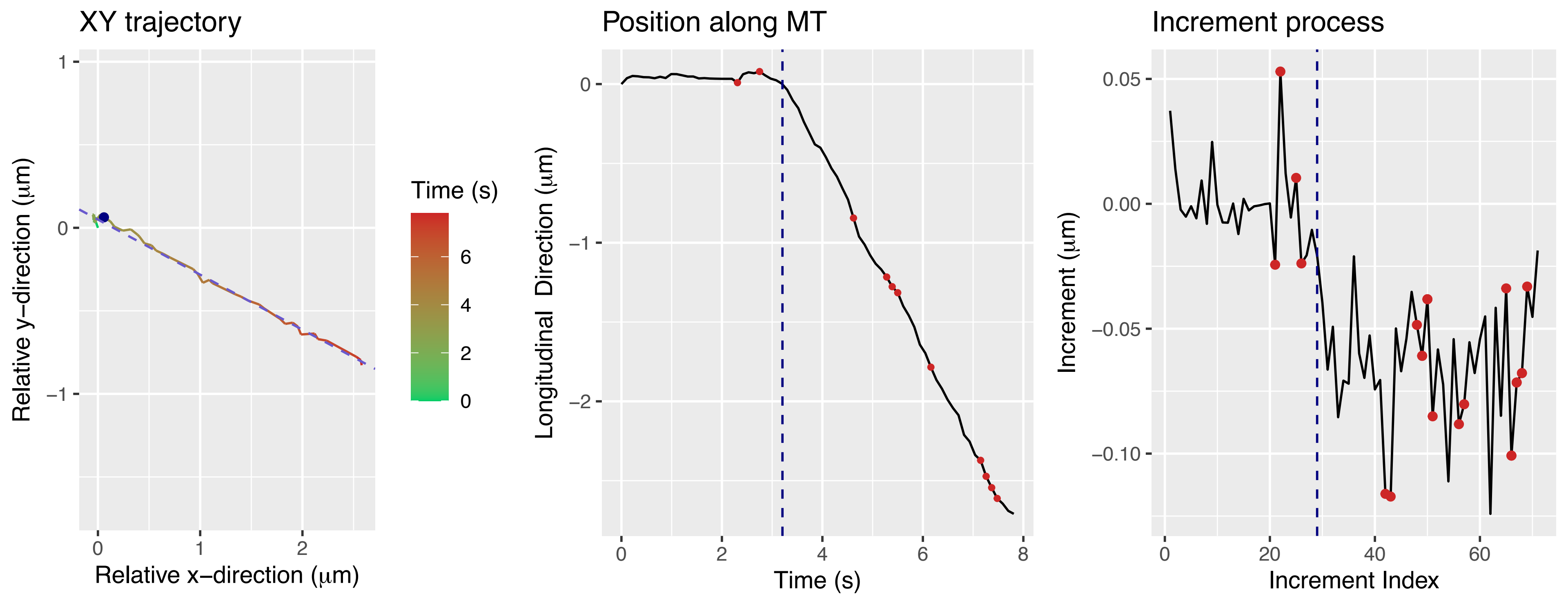

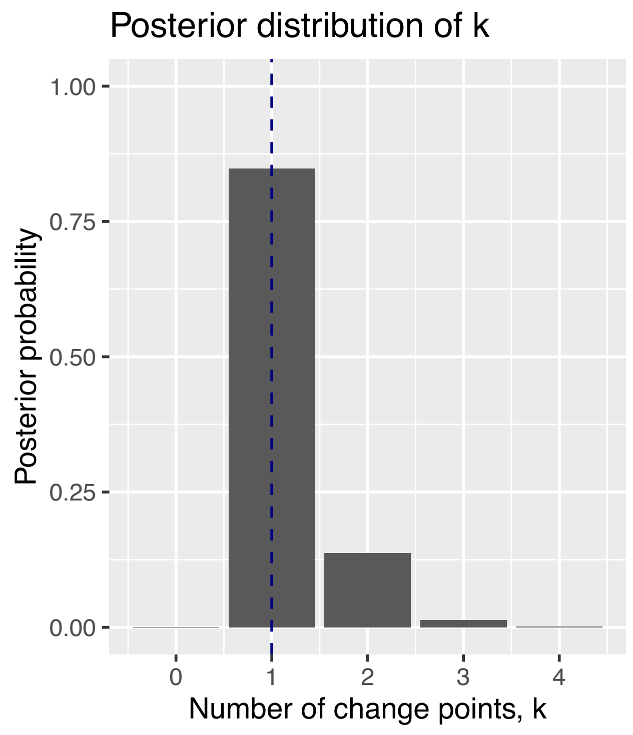

	State 1	State 2
Change point	3.2047	NA
Velocity	0.001	−0.5898
Std dev	0.0693	NA

Kin1DDB particle: 85

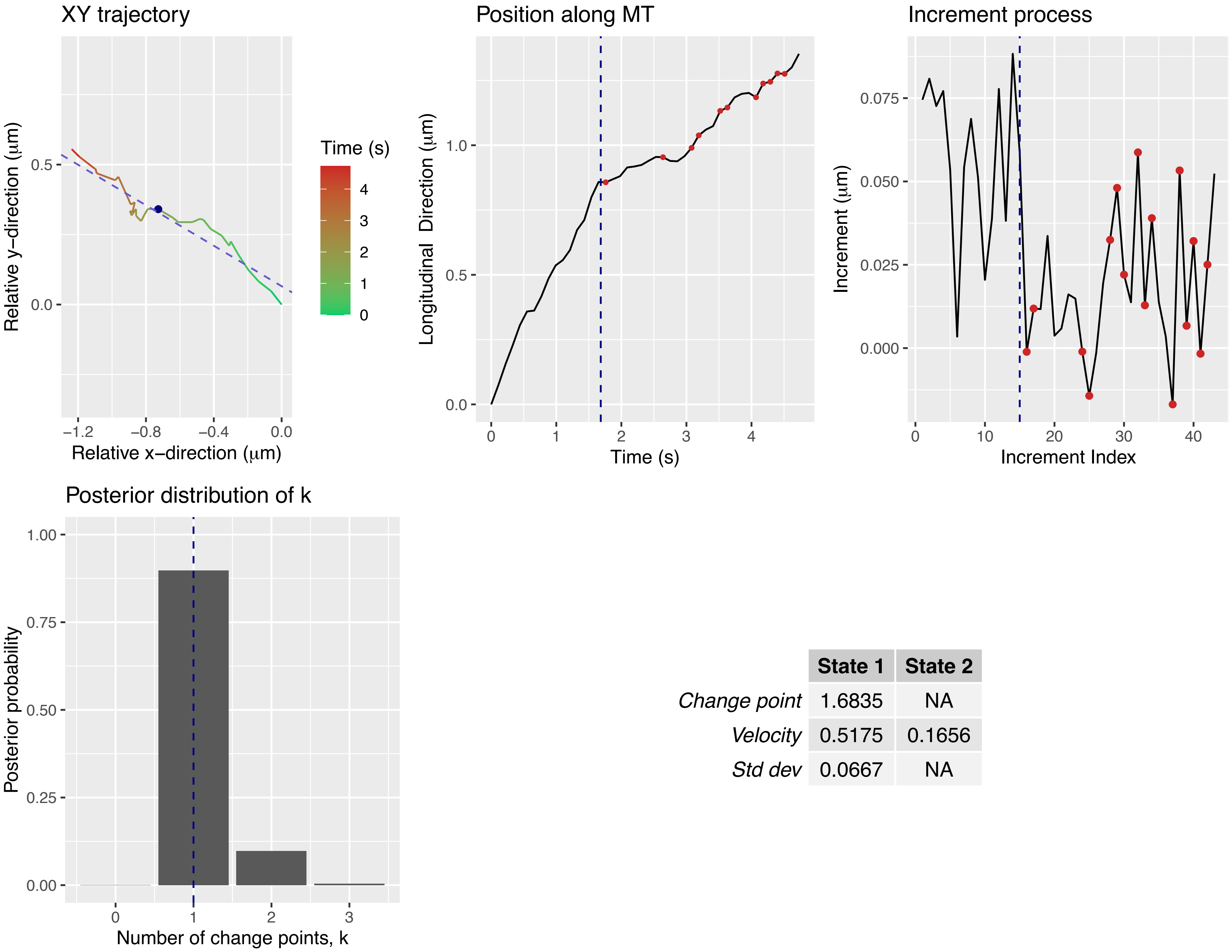

	State 1	State 2
Change point	1.6835	NA
Velocity	0.5175	0.1656
Std dev	0.0667	NA

Kin1DDB particle: 86

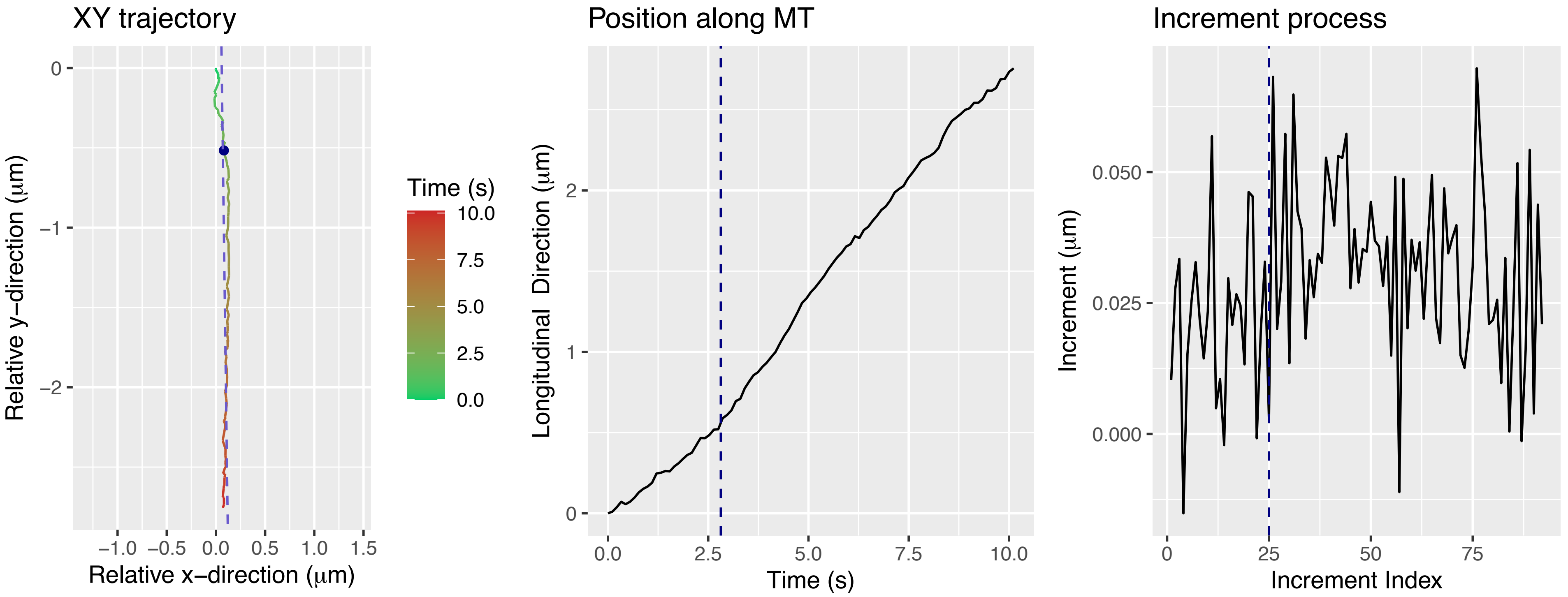

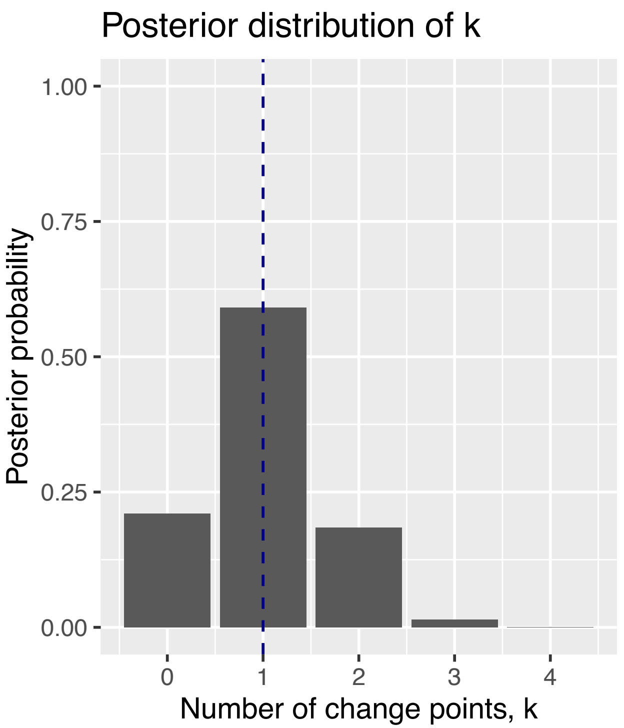

	State 1	State 2
Change point	2.8135	NA
Velocity	0.1872	0.3021
Std dev	0.0482	NA

Kin1DDB particle: 87

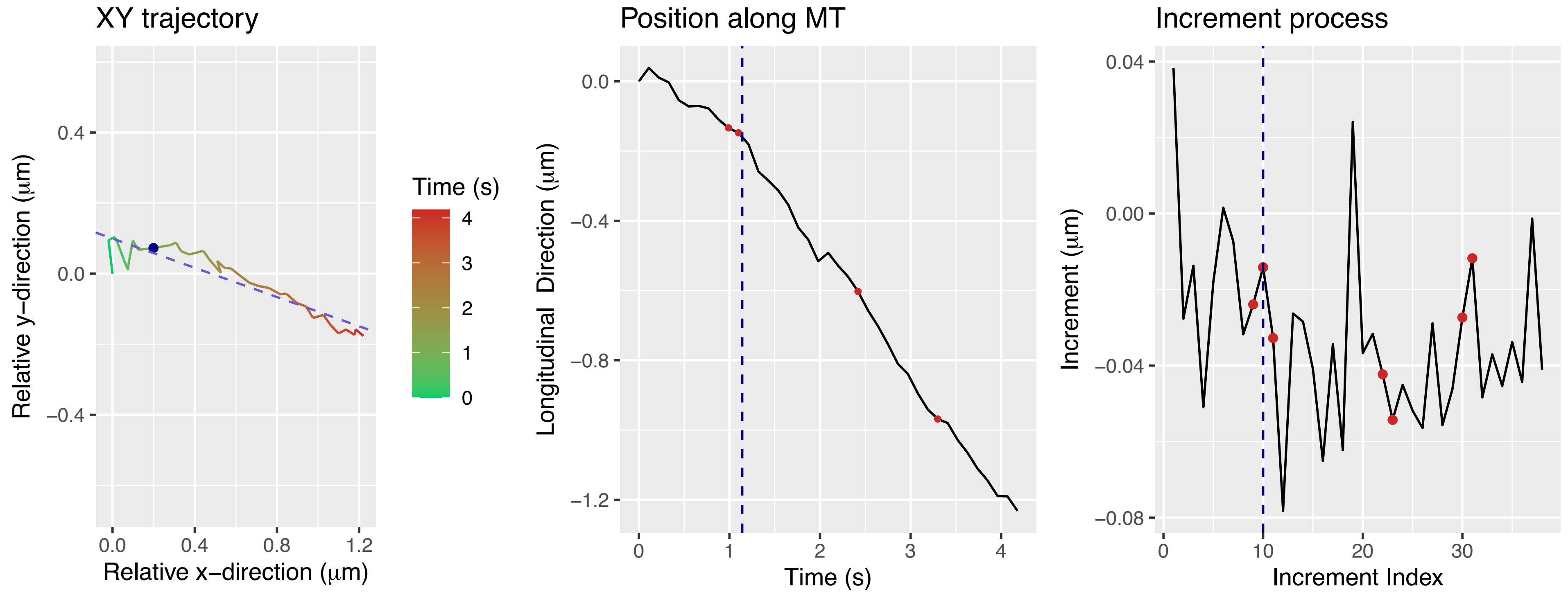

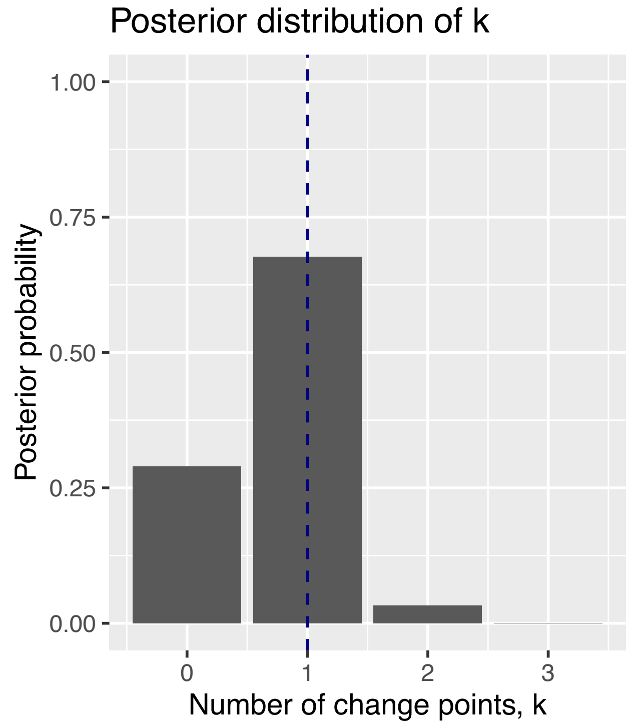

	State 1	State 2
Change point	1.1413	NA
Velocity	−0.1397	−0.3551
Std dev	0.0623	NA

Kin1DDB particle: 88

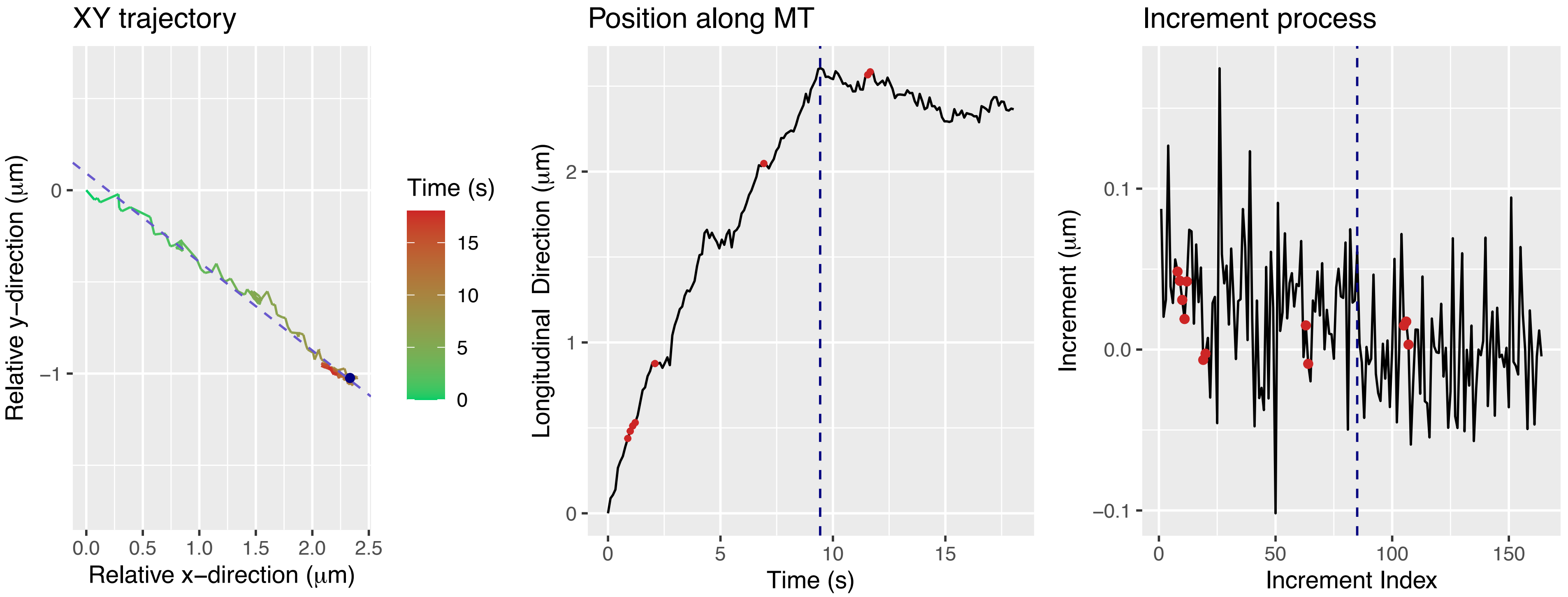

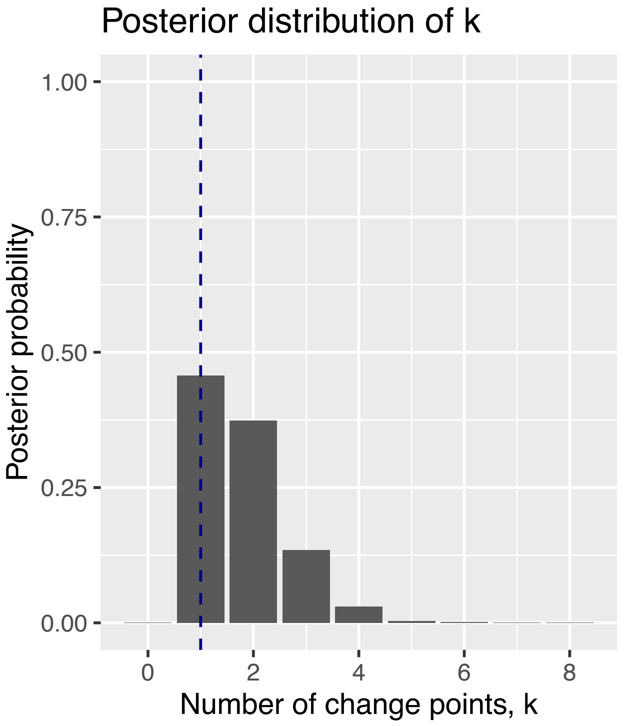

	State 1	State 2
Change point	9.4333	NA
Velocity	0.2723	−0.0246
Std dev	0.1113	NA

Kin1DDB particle: 89

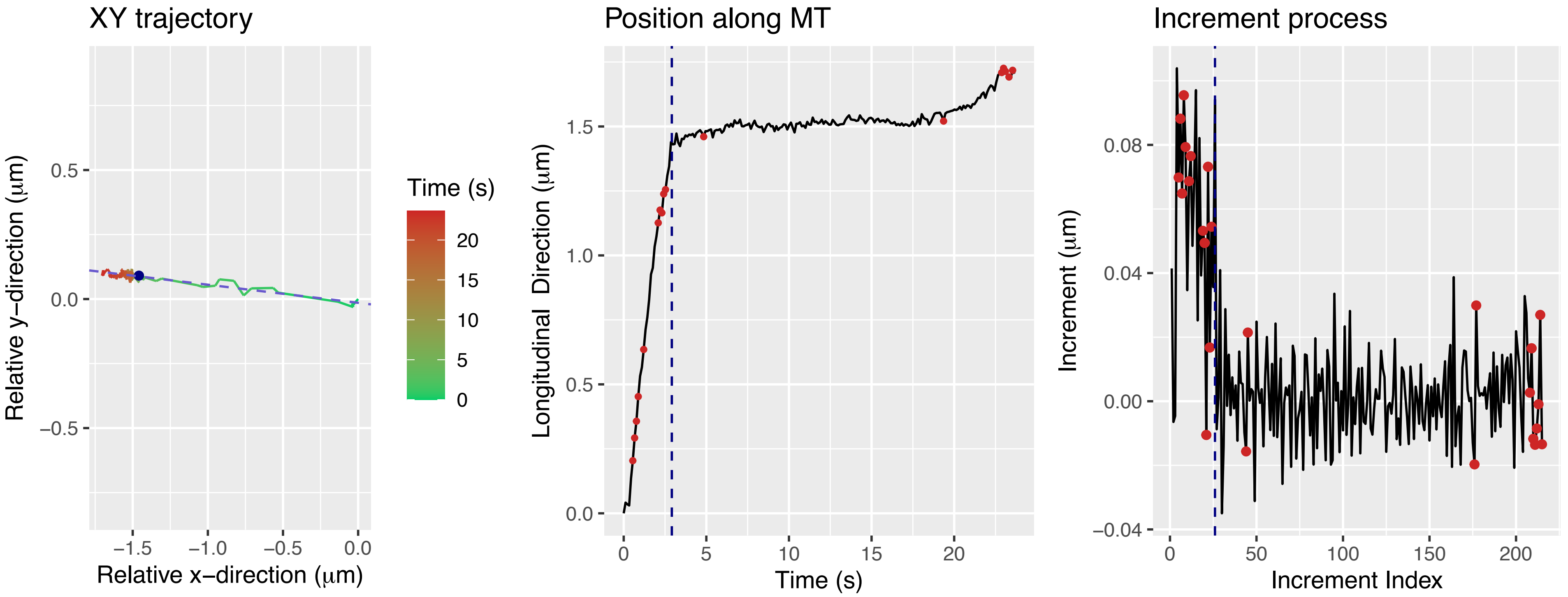

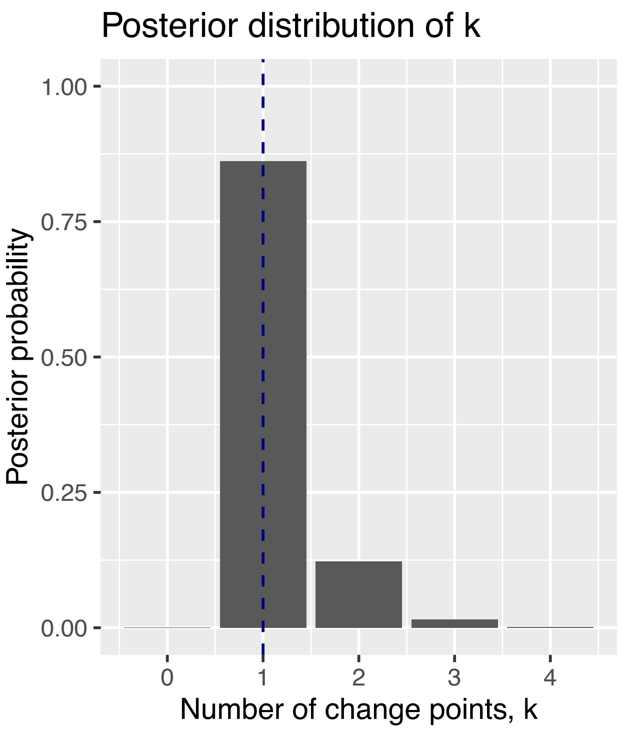

	State 1	State 2
Change point	2.9115	NA
Velocity	0.5042	0.013
Std dev	0.0515	NA

Kin1DDB particle: 90

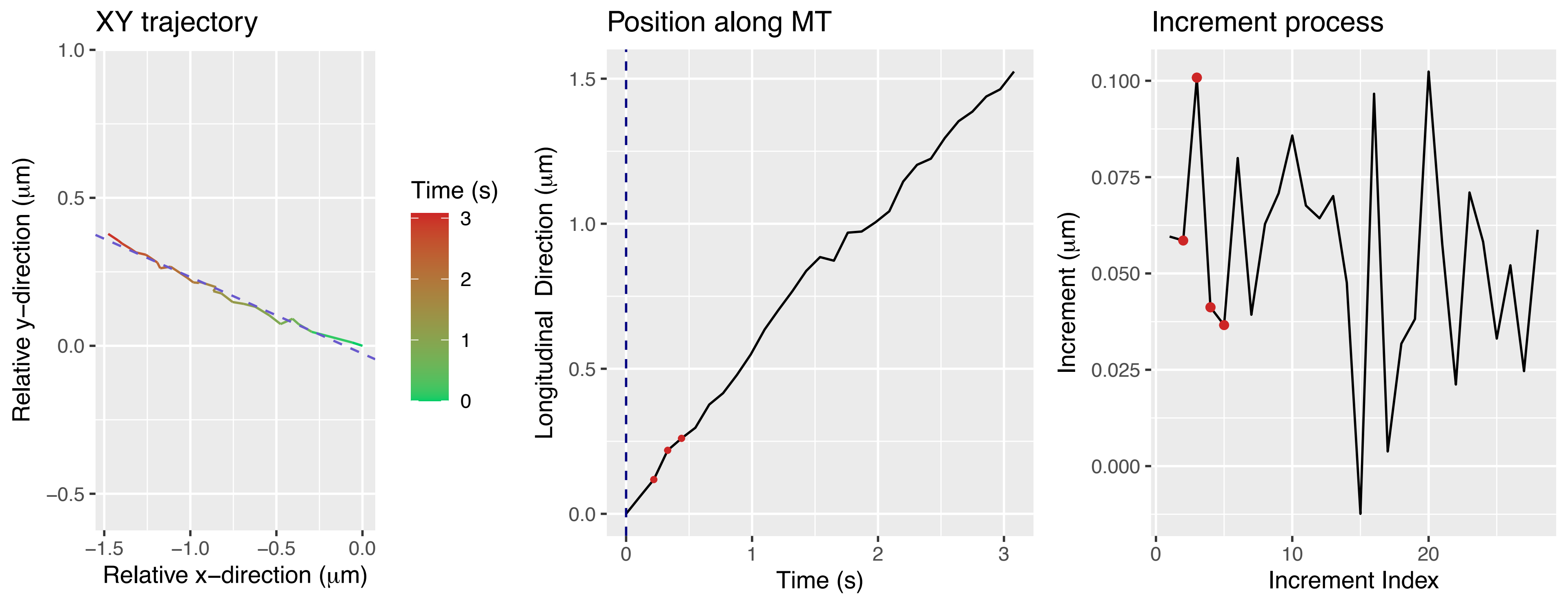

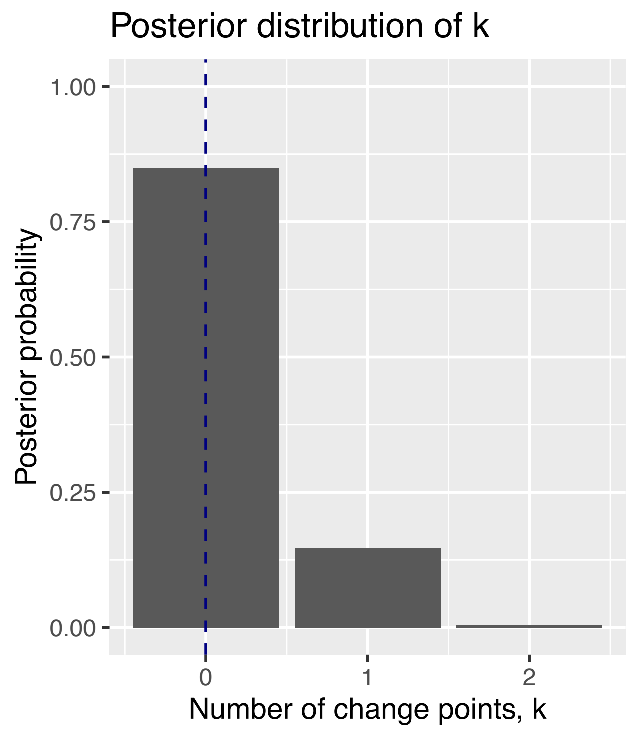

	**State 1**
Change point	0
Velocity	0.4965
Std dev	0.0787

Kin1DDB particle: 91

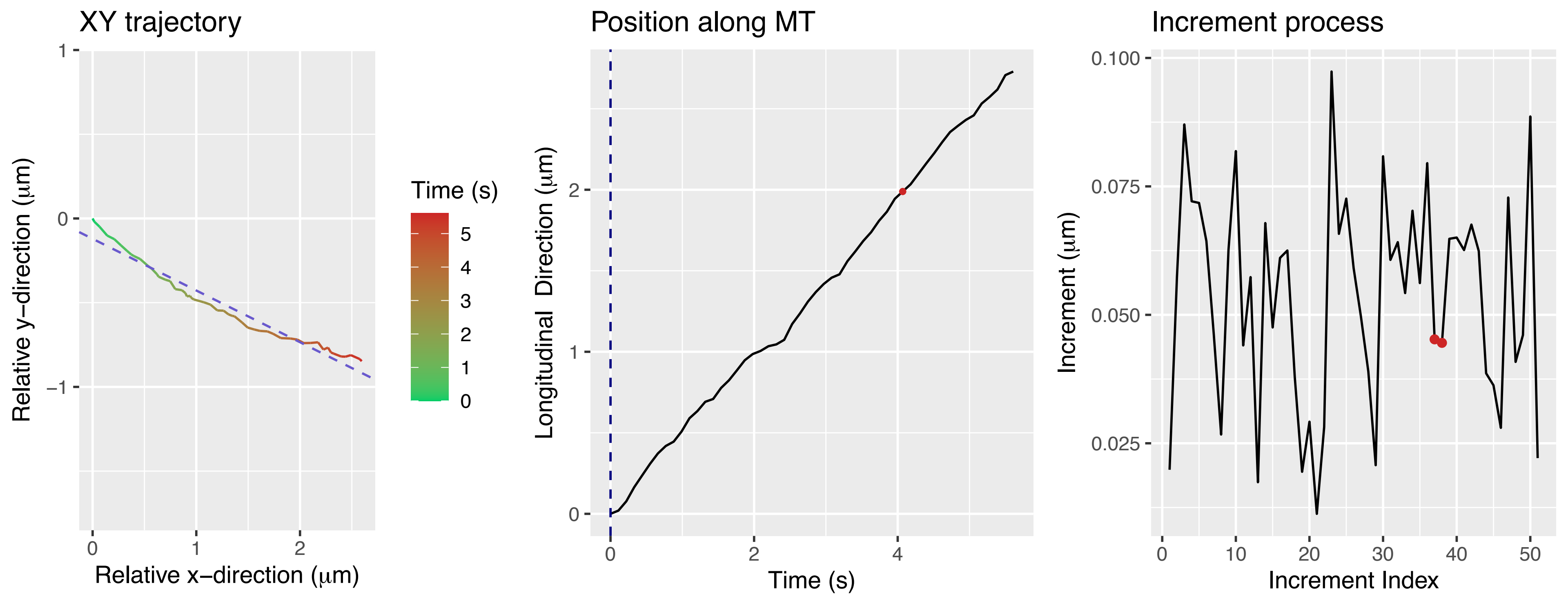

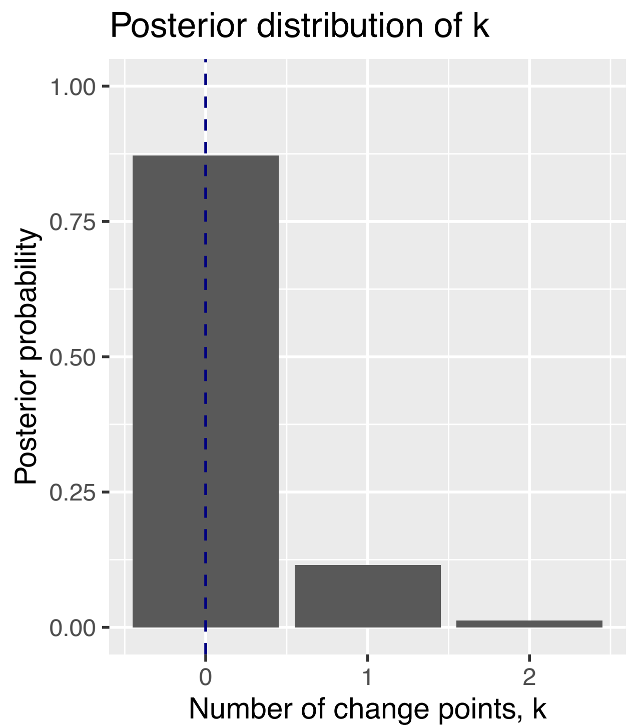

	State 1
Change point	0
Velocity	0.4861
Std dev	0.0589

Kin1DDB particle: 92

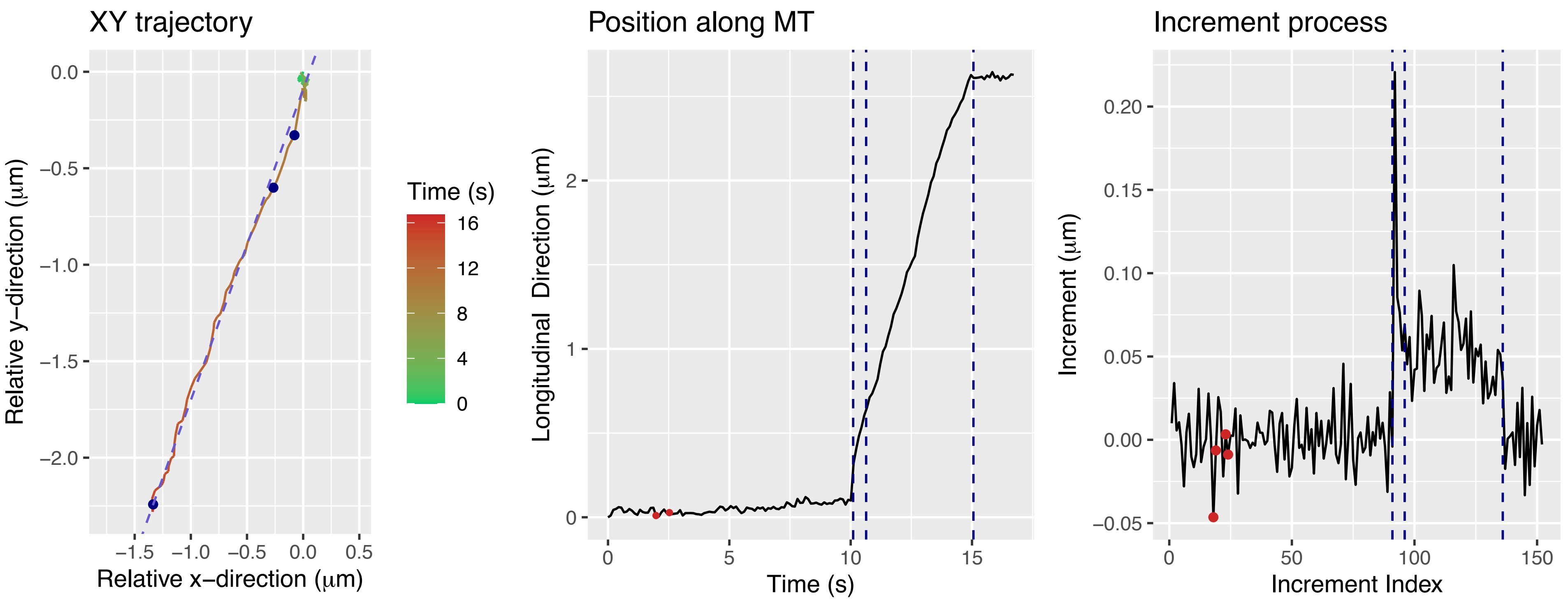

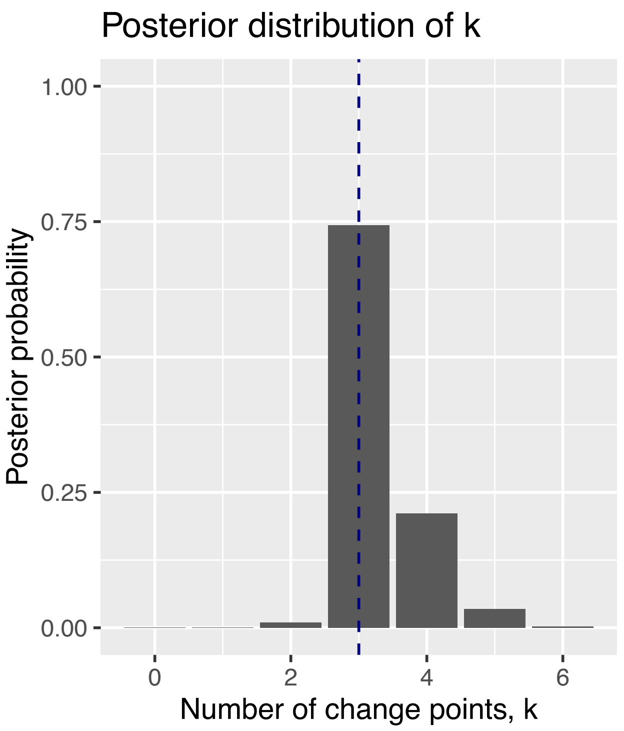

	State 1	State 2	State 3	State 4
Change point	10.1024	10.6381	15.0585	NA
Velocity	0.0075	0.9308	0.4518	−5e−04
Std dev	0.0622	NA	NA	NA

Kin1DDB particle: 93

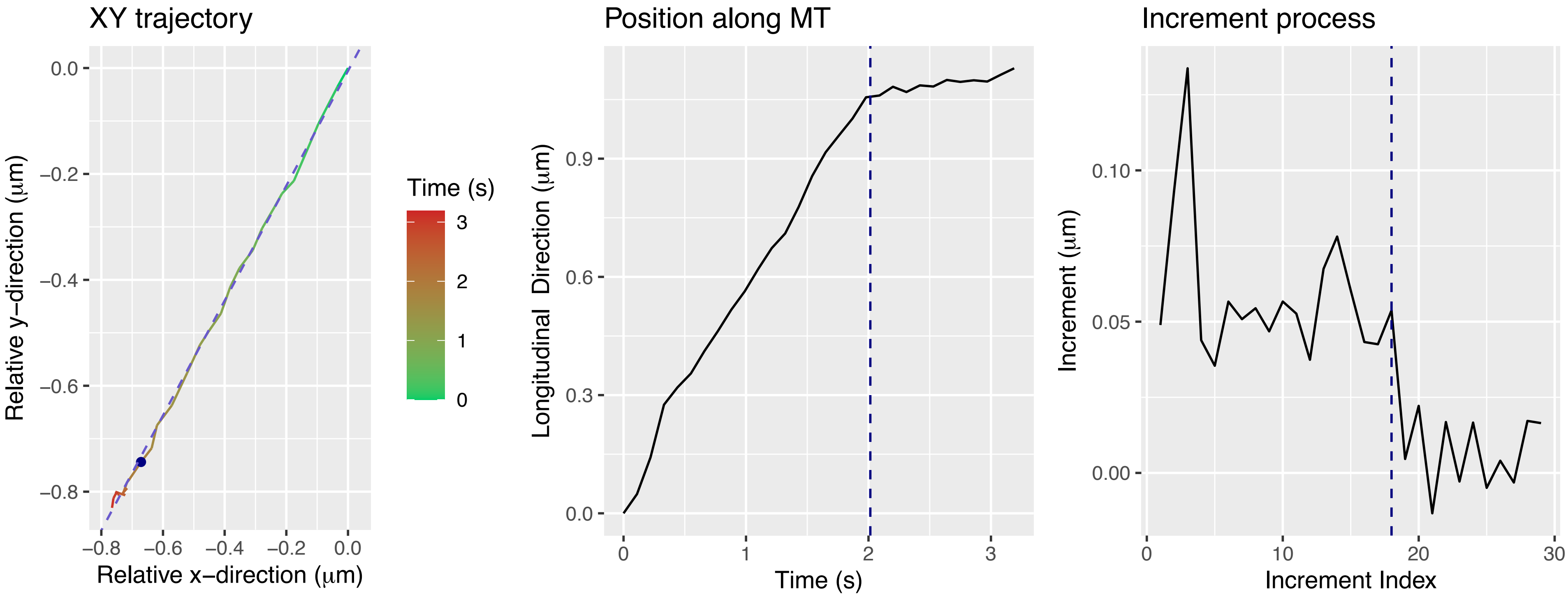

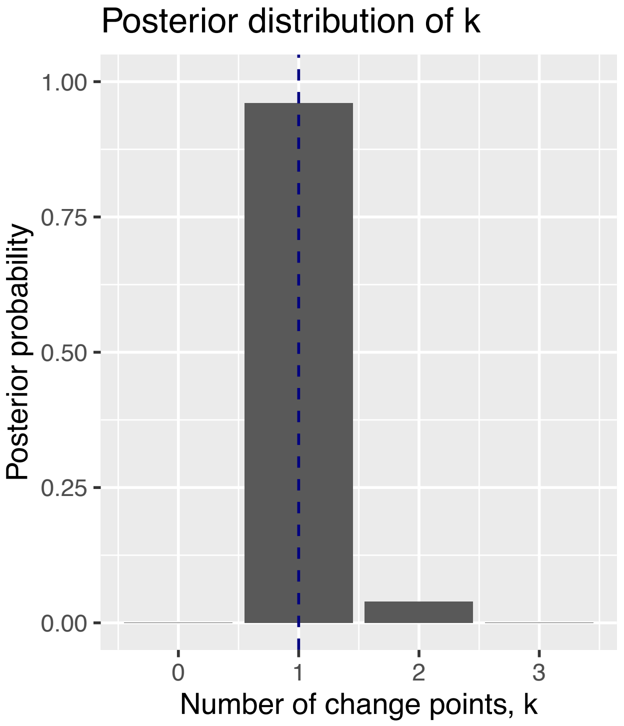

	State 1	State 2
Change point	2.0149	NA
Velocity	0.5355	0.0589
Std dev	0.0678	NA

Kin1DDB particle: 94

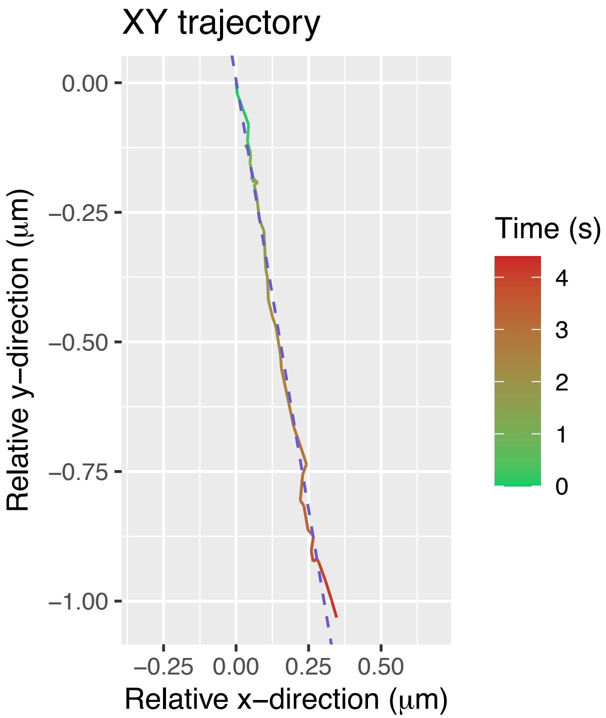

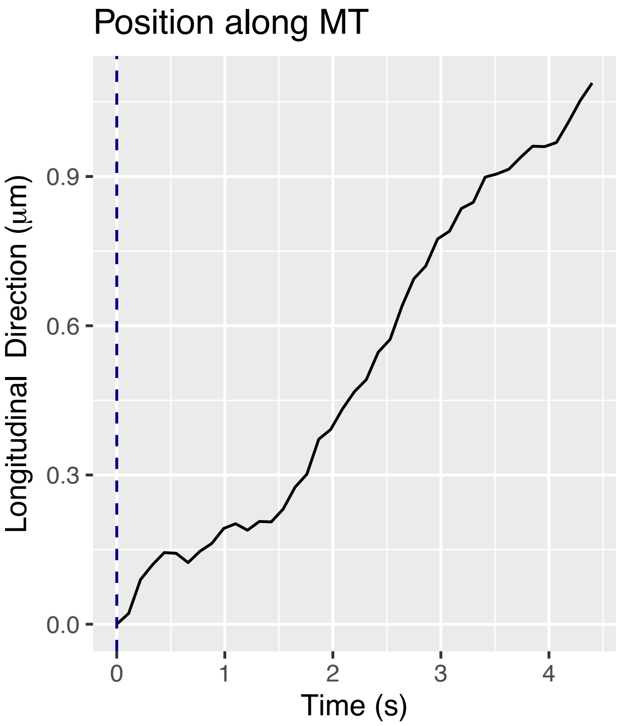

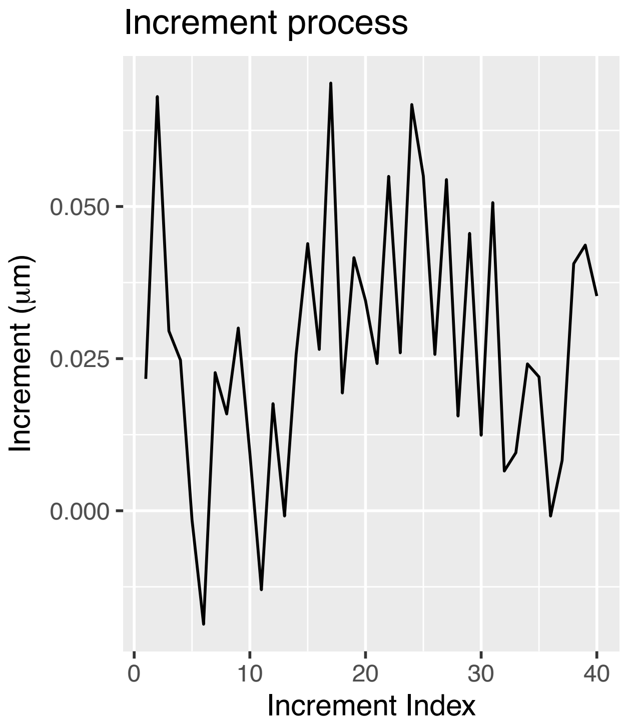

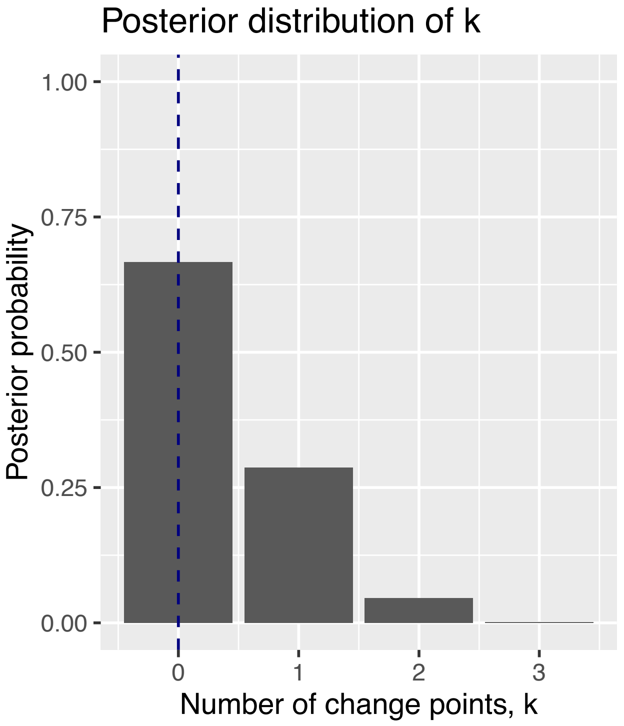

	**State 1**
Change point	0
Velocity	0.247
Std dev	0.0601

Kin1DDB particle: 95

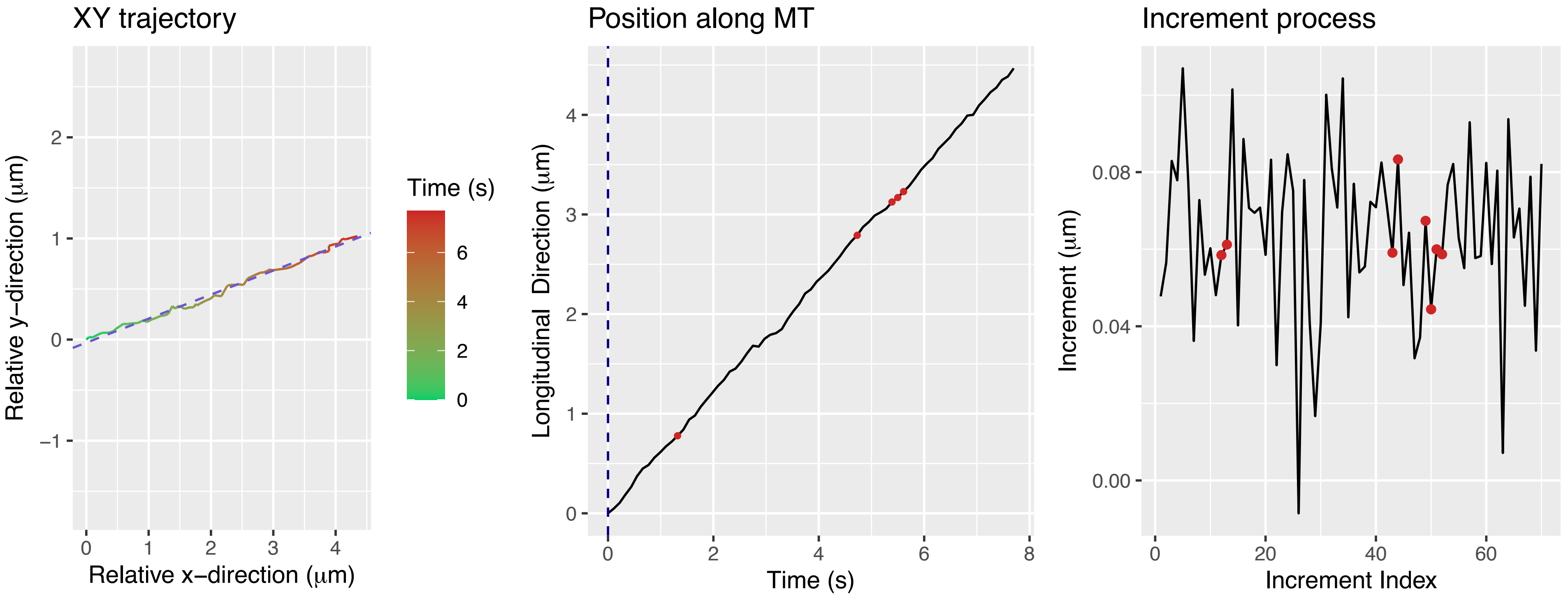

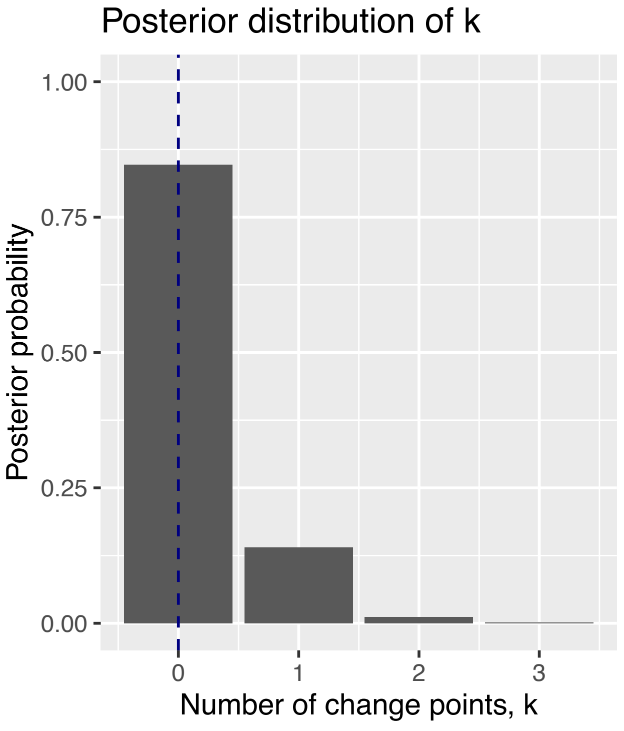

	State 1
Change point	0
Velocity	0.5794
Std dev	0.0643

Kin1DDB particle: 96

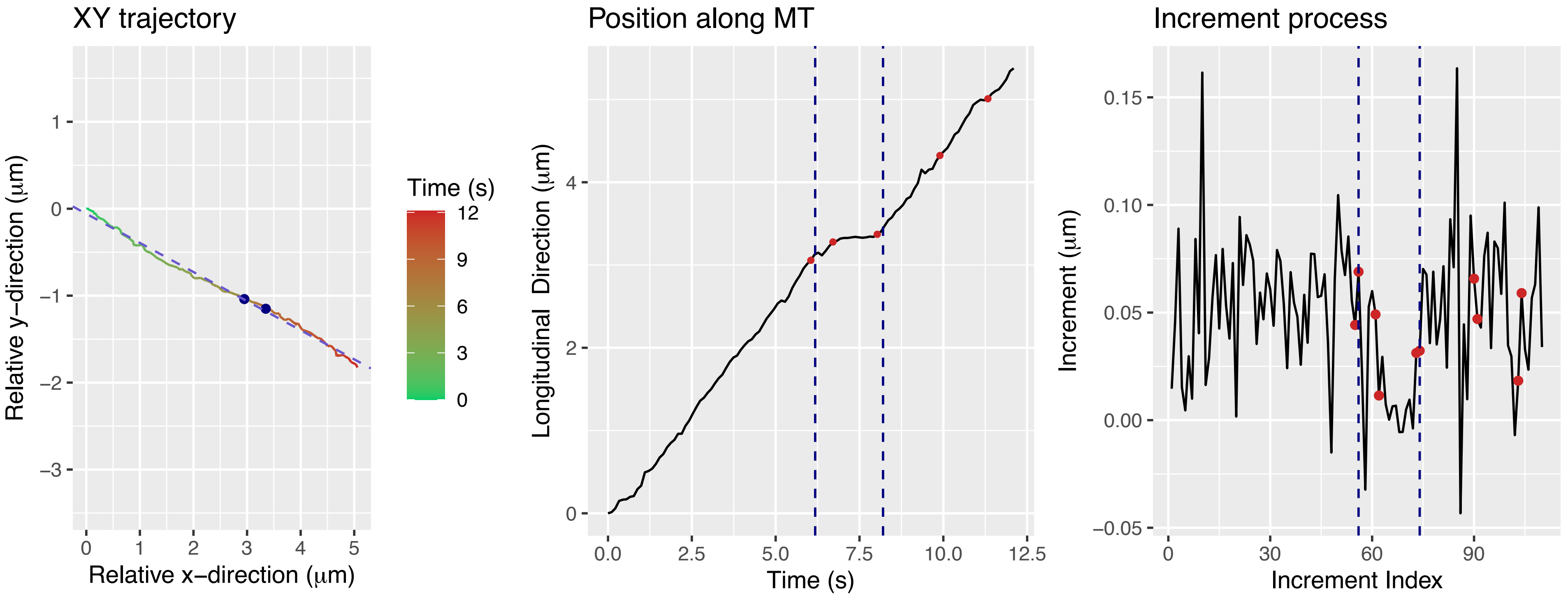

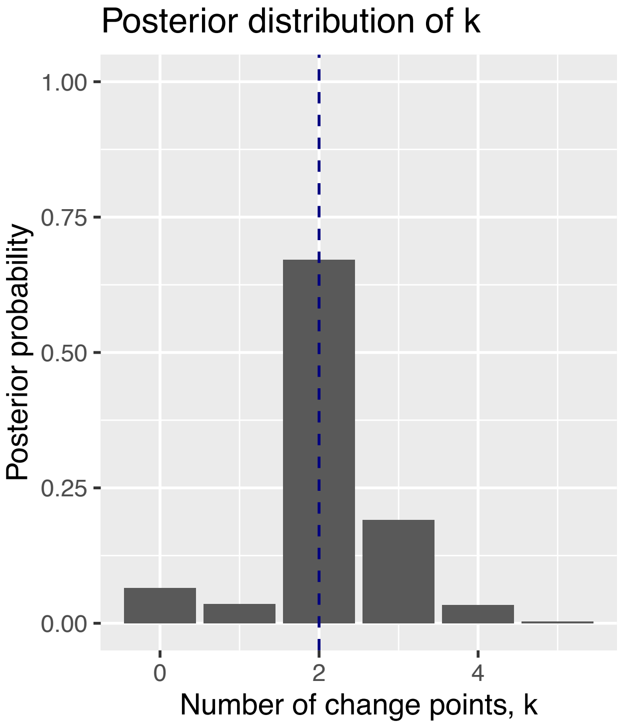

	State 1	State 2	State 3
Change point	6.1787	8.202	NA
Velocity	0.5044	0.1484	0.5053
Std dev	0.0949	NA	NA

Kin1DDB particle: 97

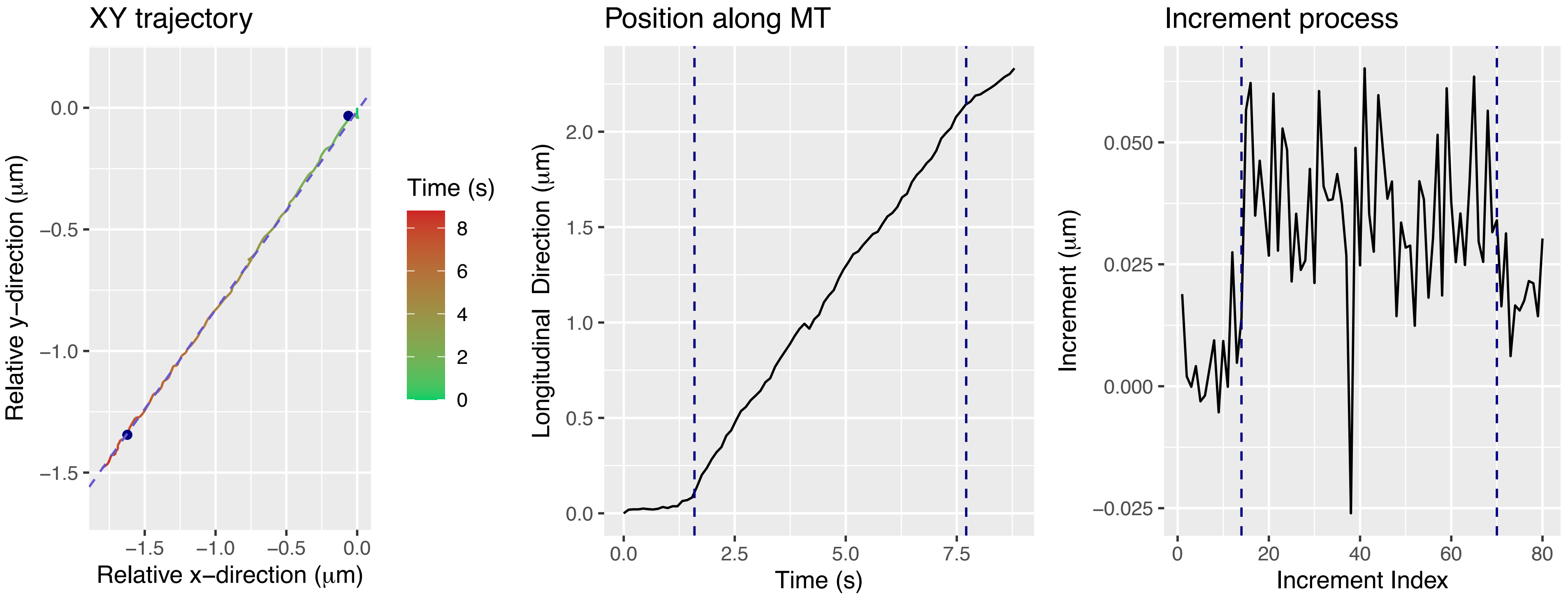

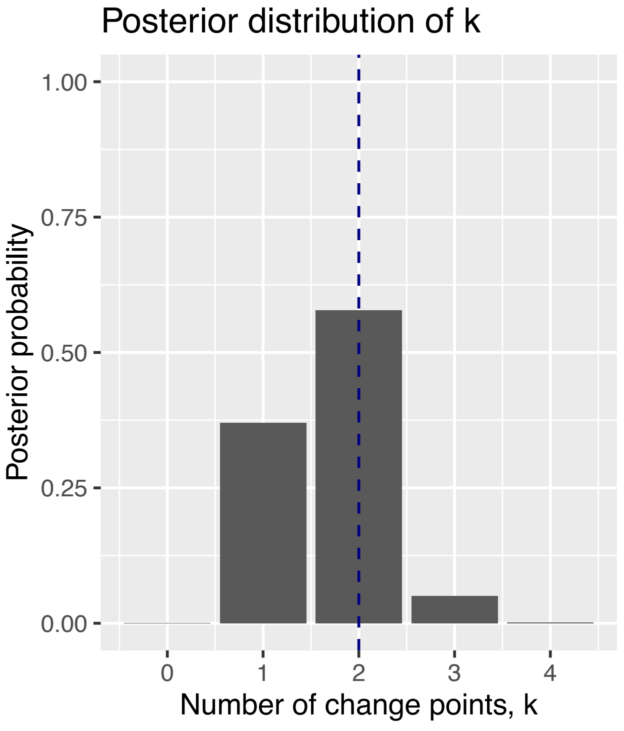

	State 1	State 2	State 3
Change point	1.5933	7.7128	NA
Velocity	0.0612	0.3376	0.1593
Std dev	0.0448	NA	NA

Kin1DDB particle: 98

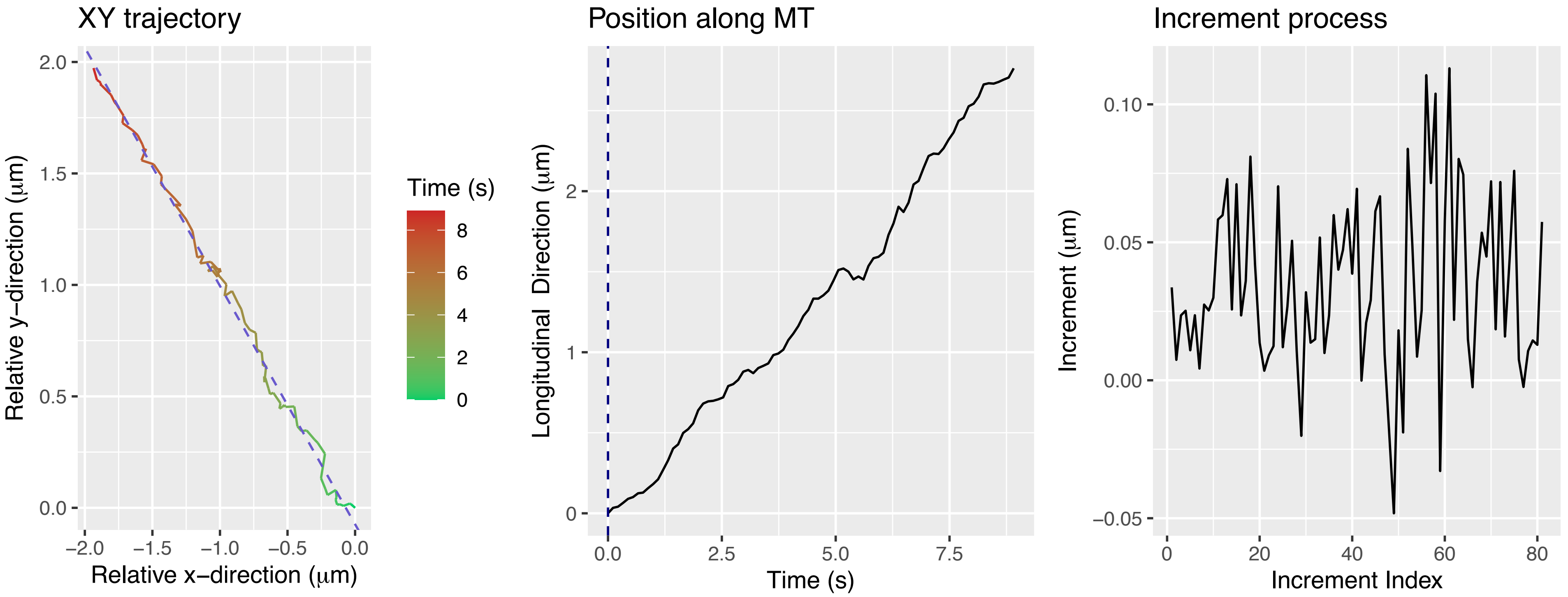

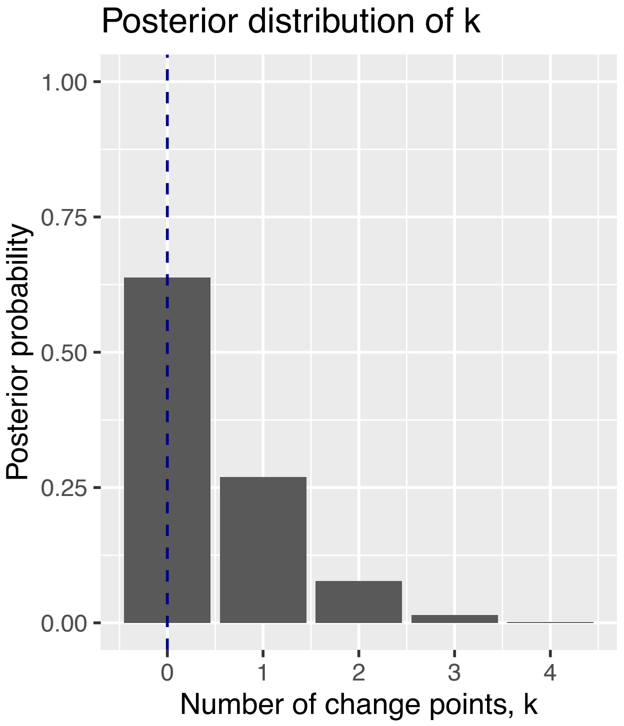

	**State 1**
Change point	0
Velocity	0.3112
Std dev	0.0954

Kin1DDB particle: 99

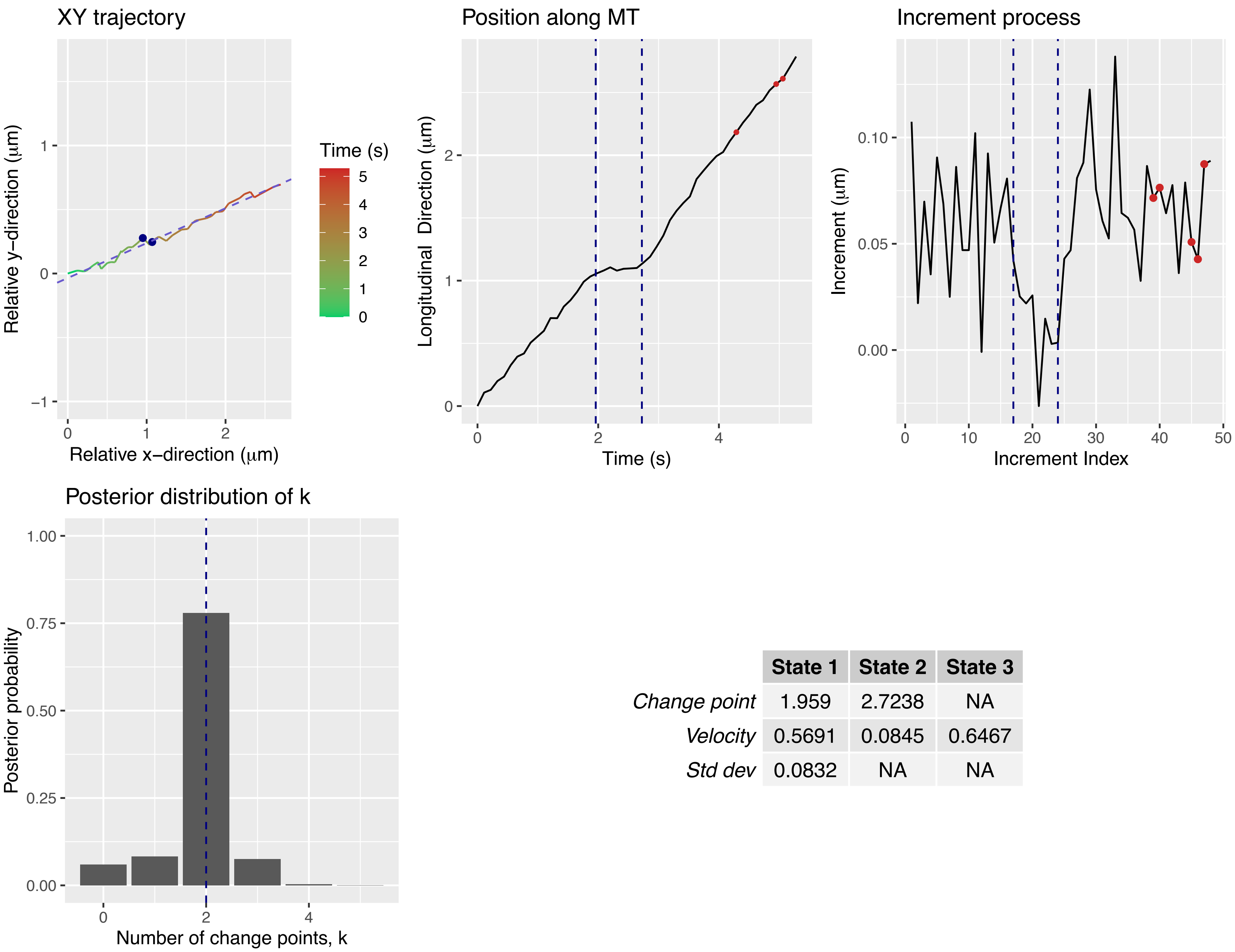

	State 1	State 2	State 3
Change point	1.959	2.7238	NA
Velocity	0.5691	0.0845	0.6467
Std dev	0.0832	NA	NA

Kin1DDB particle: 100

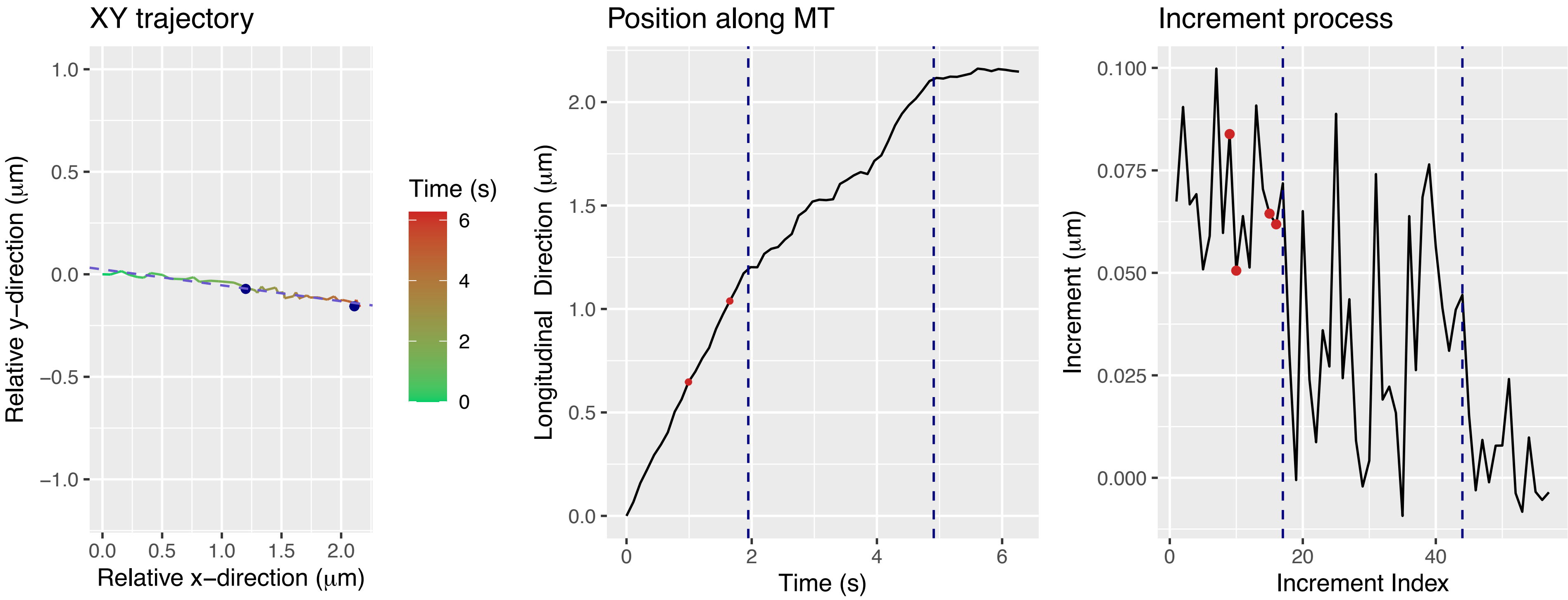

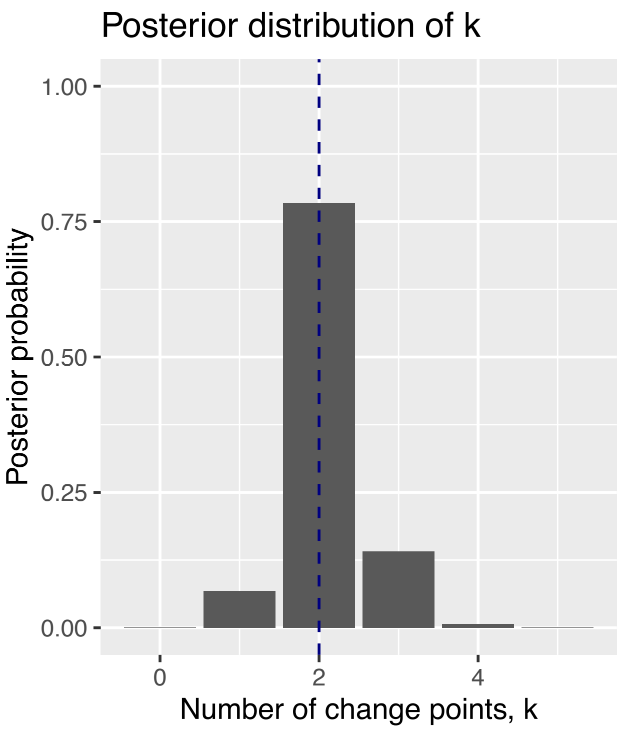

	State 1	State 2	State 3
Change point	1.9451	4.9073	NA
Velocity	0.6227	0.3142	0.011
Std dev	0.0657	NA	NA

Kin1DDB particle: 101

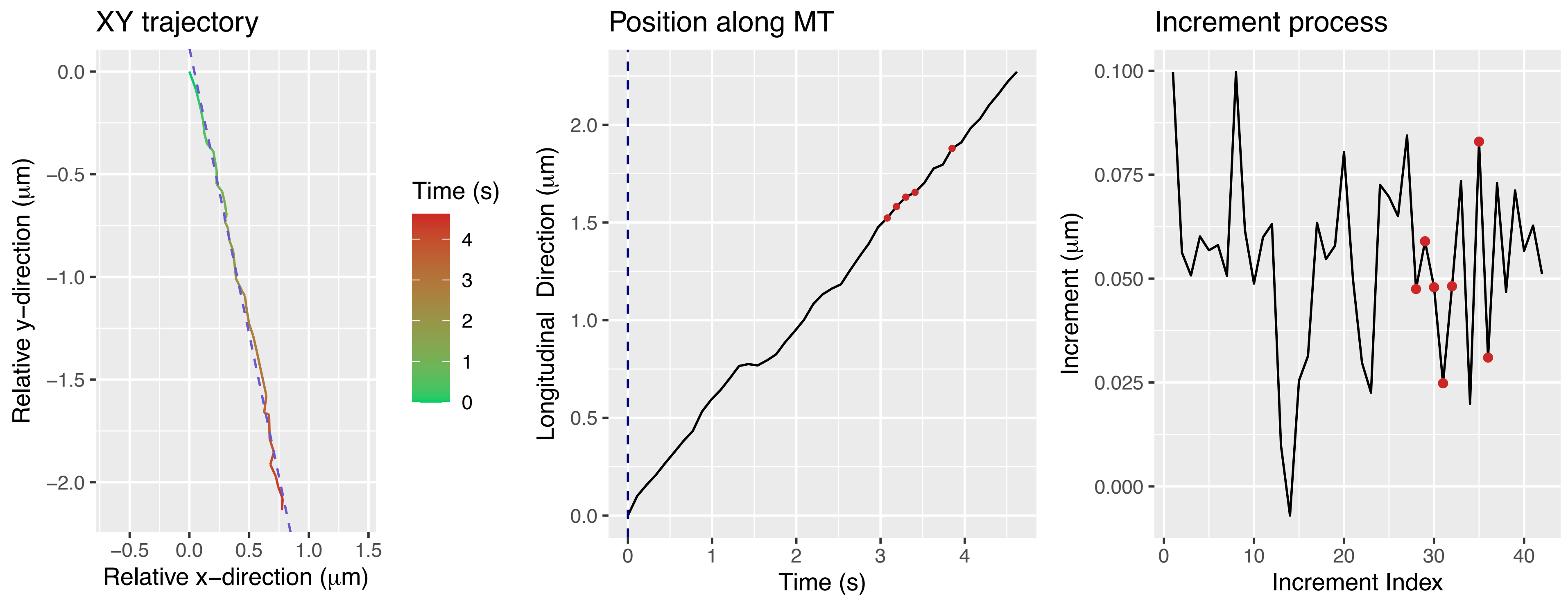

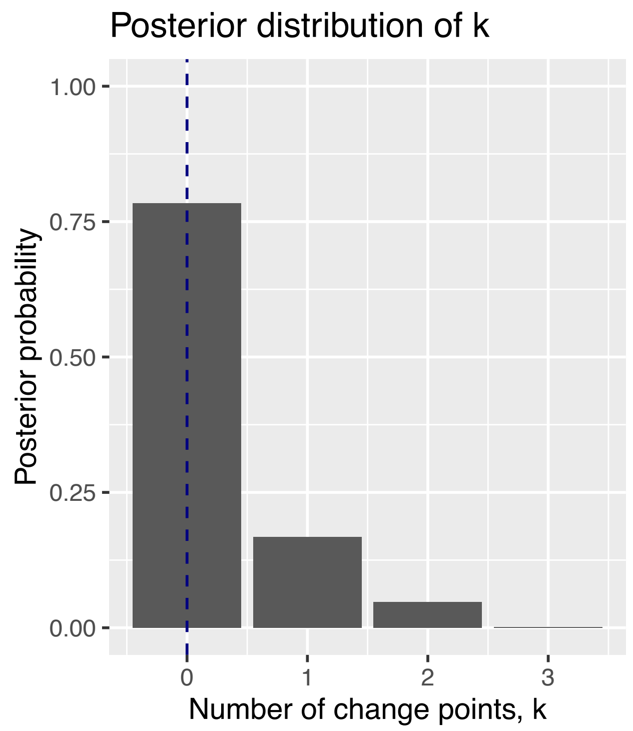

	**State 1**
Change point	0
Velocity	0.4917
Std dev	0.0632
